# Organocatalytic stereoselective cyanosilylation of small ketones

**DOI:** 10.1038/s41586-022-04531-5

**Published:** 2022-05-04

**Authors:** Hui Zhou, Yu Zhou, Han Yong Bae, Markus Leutzsch, Yihang Li, Chandra Kanta De, Gui-Juan Cheng, Benjamin List

**Affiliations:** 1 Max-Planck-Institut für Kohlenforschung, Mülheim an der Ruhr, Germany; 2 Warshel Institute for Computational Biology, School of Life and Health Sciences, The Chinese University of Hong Kong, Shenzhen, Shenzhen, China; 3 Present Address: Department of Chemistry, Sungkyunkwan University, Suwon, Republic of Korea

**Keywords:** Asymmetric catalysis, Immobilized enzymes

## Abstract

Enzymatic stereoselectivity has typically been unrivalled by most chemical catalysts, especially in the conversion of small substrates. According to the ‘lock-and-key theory’^1,2^, enzymes have confined active sites to accommodate their specific reacting substrates, a feature that is typically absent from chemical catalysts. An interesting case in this context is the formation of cyanohydrins from ketones and HCN, as this reaction can be catalysed by various classes of catalysts, including biological, inorganic and organic ones^3–7^. We now report the development of broadly applicable confined organocatalysts for the highly enantioselective cyanosilylation of aromatic and aliphatic ketones, including the challenging 2-butanone. The selectivity (98:2 enantiomeric ratio (e.r.)) obtained towards its pharmaceutically relevant product is unmatched by any other catalyst class, including engineered biocatalysts. Our results indicate that confined chemical catalysts can be designed that are as selective as enzymes in converting small, unbiased substrates, while still providing a broad scope.

## Abstract (web summary)

The development of confined organocatalysts for the enantioselective cyanosilylation of small, unbiased substrates, including 2-butanone, is shown to lead to catalysts that are as selective as enzymes, with excellent levels of control.

## Main

**Fig. 1:**
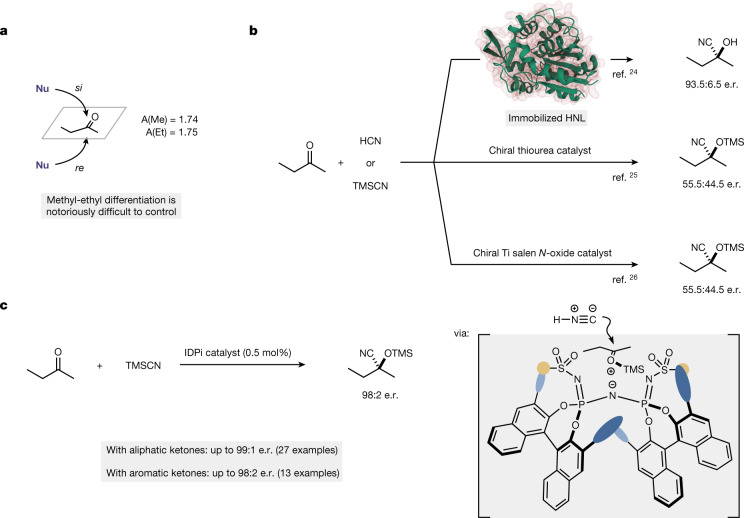
Catalytic asymmetric cyanosilylation of carbonyl compounds. **a**, Enantiofacial differentiation in 2-butanone. **b**, Previously reported asymmetric cyanations of 2-butanone with an enzyme, an organocatalyst and a transition-metal catalyst. **c**, This work: an IDPi-catalysed, highly enantioselective silylcyanation of 2-butanone and a broad scope of other ketones.

The amount of discrimination that enzymes show when operating on their substrates is unique. Frequently, only a single substrate is even accepted. Regioselectivities, diastereoselectivities and enantioselectivities typically achieved by enzymes have served as an inspiration for chemists in their aim to create perfect catalysts. This quest is particularly formidable when it comes to small substrates, which are notoriously difficult to handle in enantioselective processes. An example of this category concerns the catalytic enantiofacial differentiation of 2-butanone. Given the similar steric bulk of the ketone substituents, for example, as expressed with their corresponding A values (Me 1.74, Et 1.75)^8,9^, such a stereoselective process represents a daunting task (Fig. 1a). Although enzymes and an iridium complex have been described that reduce 2-butanone to the corresponding alcohol with high enantioselectivity^10,11^, nucleophilic additions of carbon nucleophiles generally give poor results^12–14^. This also applies for the synthesis of the cyanohydrin that is produced in the addition of HCN to 2-butanone. Notably, the hydrolysis product of this particular cyanohydrin, 2-hydroxy-2-methyl-butyric acid, is a privileged pharmacophore contained in three marketed drugs, beclobrate, clinofibrate and paramethadione^15–17^. Furthermore, the (*S*)-enantiomer is used in the preparation of a COX-2 inhibitor and PPAR agonist, whereas the (*R*)-enantiomer builds the skeletons or side chain of several biologically active natural products^18–23^. To the best of our knowledge, until now, only hydroxynitrile lyases (HNL) delivered satisfactory results in the asymmetric hydrocyanation of 2-butanone, with a maximum enantioselectivity of 93.5:6.5 (87% enantiomeric excess), when the engineered enzyme *Lu*HNL was used^24^. Previously developed chiral thiourea organocatalysts and a chiral salen titanium complex gave only poor amounts of enantiocontrol with this important substrate^25,26^ (Fig. 1b). We were inspired by recent studies on using strong and confined acid catalysts to control difficult substrates and reactions. For example, we have shown that the acetaldehyde enolsilane only reacts once with another aldehyde, with high enantioselectivity, in the presence of one such confined imidodiphosphorimidate (IDPi) catalyst^27^. Further encouragement came from our studies on Diels–Alder and hydroarylation reactions, in which we noticed that IDPi catalysts can differentiate between ethyl and methyl groups, although with moderate enantioselectivities^28,29^. With these results, demonstrating the control that confined catalysts can show, and the underlying catalysis principle of silylium ion asymmetric counteranion directed catalysis (*Si*-ACDC)^30,31^, we reasoned that a tailored, strong and perhaps even more confined silylium-IDPi organocatalyst could accomplish the targeted highly enantioselective cyanosilylation of 2-butanone (Fig. 1c). We suggested that a critical silyl oxocarbenium cation may ion pair with its confined IDPi counteranion in such a way that only one of the two enantiofaces would be exposed towards attack by the cyanide sp-nucleophile.

**Fig. 2:**
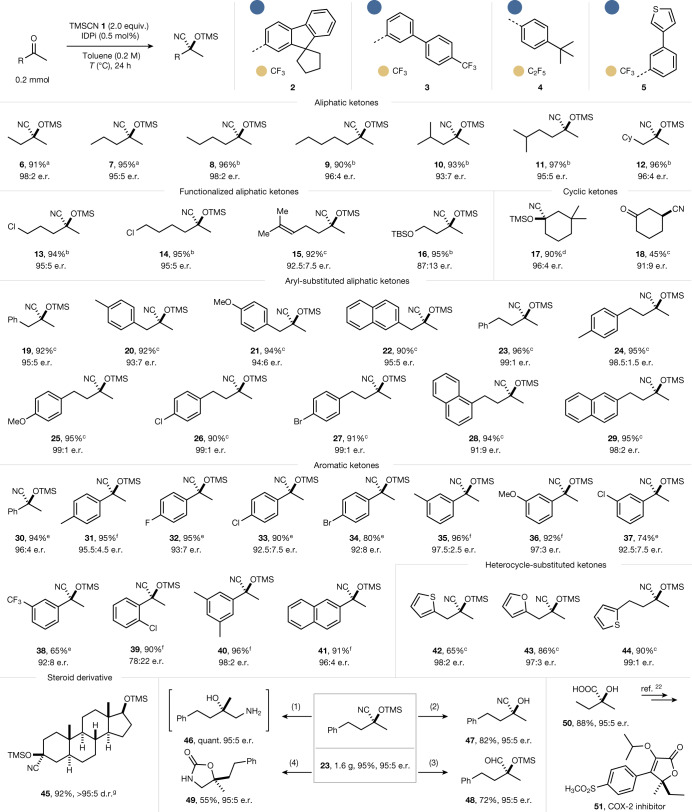
Substrate scope of the catalytic asymmetric cyanosilylation of ketones. Performed with TMSCN **1** (2.0 equiv.) and IDPi (0.5 mol%) in toluene (0.2 M) at rt for 0.5 h, then different ketones (0.2 mmol) were added at the indicated temperature for 24 h. Isolated yields after chromatographic purification. The e.r. was determined by high-performance liquid chromatography or gas chromatography analysis. ^a^With catalyst **2** at −100 °C. ^b^With catalyst **3** at −100 °C. ^c^With catalyst **4** at −80 °C. ^d^With catalyst **2** at −80 °C. ^e^With catalyst **5** (5.0 mol%) in Et_2_O at −100 °C for 5 days. ^f^With catalyst **5** (2.5 mol%) in Et_2_O at −100 °C for 5 days. ^g^With catalyst **2** at 25 °C for 12 h. Applications and functionalizations. Performed with TMSCN **1** (2.0 equiv.) and IDPi **4** (0.5 mol%) in Et_2_O (0.4 M) at rt for 3.0 h, followed by the addition of 4-phenylbutan-2-one (1.0 g, 6.7 mmol) at −60 °C for 24 h. (1) LiAlH_4_ (2.0 equiv.), Et_2_O, rt, 1 h. (2) TFA, DCM, rt, 6 h. (3) DIBAL-H (1.5 equiv.), Et_2_O, rt, 12 h. (4) One-pot synthesis: LiAlH_4_ (2.0 equiv.), Et_2_O, rt, 1 h, then carbonyldiimidazole (1.0 equiv.), THF, rt, 12 h. Synthesis of the key intermediate **50** for COX-2 inhibitor: product **6** (95:5 e.r.) made from gram-scale synthesis was treated with HCl (12.0 M), 80 °C, 3 h.

Indeed, after extensive catalyst evaluation (see Supplementary Information Tables S1–S6), IDPi **2** was found to be a particularly promising motif and afforded product **6** in quantitative yield with an e.r. of 98:2, which constitutes the highest enantiofacial selectivity ever obtained with 2-butanone. Additionally, IDPis **3**–**5** also emerged as privileged catalysts. Using catalysts **2**–**5**, the scope of ketones was explored (Fig. 2). Aliphatic ketones bearing a methyl group and a longer *n*-alkyl group were tested at the outset. Our IDPi catalysts were found to be competent, affording the corresponding cyanohydrin silyl ethers in 90–97% yield, with e.r. values ranging from 93:7 to 98:2, independent of the length of the alkyl chain (products **6**–**12**). Chlorinated ketones were also found to be suitable substrates, furnishing the corresponding silylcyanohydrins **13** and **14** in 94% and 95% yield with 95:5 e.r., respectively. A substrate bearing an alkenyl substituent was also well tolerated, providing product **15** with an e.r. of 92.5:7.5 in 92% yield. Moreover, ketone bearing a protected hydroxy substituent readily gave the desired product **16** in 95% isolated yield with an e.r. of 87:13. In addition, a cyclic ketone could also be successfully used and product **17** was obtained in high yield and with high enantioselectivity when 3,3-dimethyl cyclohexanone was subjected to the reaction conditions. Remarkably, 1,4-addition product **18** was observed as the main product with an e.r. of 91:9 in 45% yield when we reacted 2-cyclohexenone. By contrast, 1,2-adducts were exclusively obtained in moderate e.r. when conjugated acyclic ketones were subjected to the reaction conditions (see Supplementary Information Fig. S6). Aryl-substituted aliphatic ketones reacted similarly and neither electronic effects nor the substitution pattern on the aromatic group notably influenced the enantioselectivity (91:9–99:1 e.r.) (products **19**–**29**).

Subsequently, we explored the reactions of aromatic ketones with both electron-donating (Me, OMe) and electron-withdrawing (F, Cl, Br, CF_3_) groups in different positions on the phenyl ring, delivering the desired products **30**–**41** with moderate to high yields and high enantioselectivities (up to 98:2 e.r.), although higher catalyst loading was required. Cyanosilylations of ketones bearing furyl and thienyl groups, which are moderately basic heterocyclic substituents, also proceeded smoothly, delivering the corresponding products **42**–**44** in moderate to good yields and with high enantioselectivities. To our delight, steroid derivative **45** could also be obtained in 92% yield with a remarkable diastereoselectivity (>95:5 d.r.). It is noteworthy that a shorter reaction time could be observed when the reaction was performed under anhydrous conditions, on pre-drying at room temperature (rt) (see Supplementary Information Figs. S1–S5).

To demonstrate the synthetic utility of our reaction, we performed the cyanosilylation of 4-phenylbutan-2-one on a gram scale, furnishing product **23** in 95% yield with 95:5 e.r. Enantioenriched tertiary cyanohydrins are synthetically important building blocks, which can be easily transformed into natural products and biologically or pharmaceutically active compounds^32^. We investigated the synthetic potential of cyanohydrin precursor **23**. The corresponding amino alcohol **46**, free cyanohydrin **47**, aldehyde **48** and oxazoline **49** could be prepared in good to high yields without erosion of enantiopurity under simple, mild and concise conditions. Moreover, silylcyanohydrin **6**, which we also made on a gram scale, can be easily and efficiently converted into 2-hydroxy-2-methylbutyric acid **50**, which is an important intermediate in the synthesis of COX-2 inhibitor **51**, an effective anti-inflammatory drug^22^, as well as—potentially—various other enantiopure pharmaceuticals, applied as racemates at present^33,34^.

**Fig. 3:**
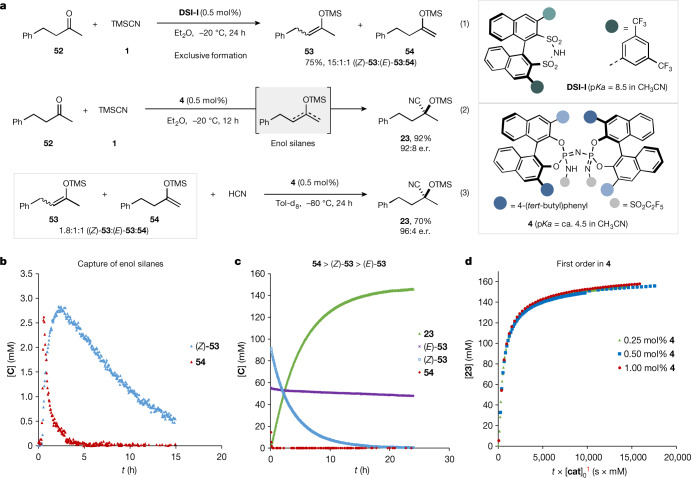
Mechanistic studies on the catalytic asymmetric cyanosilylation of ketones with TMSCN. **a**, Reactivity comparison between **DSI-I** and confined IDPi **4** catalyst (eq. 1 and eq. 2); reaction of enol silane mixture and HCN catalysed by IDPi **4** (eq. 3). **b**, Enol silane formation captured by ^1^H NMR in the reaction of 4-phenylbutan-2-one **52** and TMSCN **1** catalysed by IDPi **4**. **c**, Reaction profile of different enol silanes with HCN. **d**, Reaction order determination of the reaction components by variable time normalization analysis following the method of Burés: concentration plots obtained from ^1^H NMR showing the formation of silylcyanohydrin **23** with a timescale normalized to a first-order dependence on catalyst concentration.

Enol silanes were exclusively obtained under disulfonimide (DSI) catalysis, in accordance with our previously established silicon–hydrogen exchange reaction^35^. For example, enolsilanes were formed in 75% yield and a ratio of 15:1:1 ((*Z*)-**53**:(*E*)-**53**:**54**) from 4-phenylbutan-2-one (Fig. 3a, eq. 1). Instead, the cyanosilylation of ketone **52** enabled by IDPi **4** furnished the desired silyl cyanohydrin product **23** in 92% yield with 92:8 e.r. (Fig. 3a, eq. 2). Unexpectedly, we found that, even under these conditions, using IDPi **4** as the catalyst, the corresponding enol silanes (*Z*)-**53** and **54** could clearly be detected during the reaction by ^1^H NMR spectroscopy (Fig. 3b). A further control experiment in which an enol silane mixture was directly reacted with HCN and 0.5 mol% of catalyst **4** was performed next in toluene-d_8_ at −80 °C for 24 h, affording silylcyanohydrin **23** in 70% yield with 96:4 e.r. (Fig. 3a, eq. 3). Towards a deeper understanding of the reaction mechanism, we monitored the reaction progress by ^1^H NMR spectroscopy. As shown in Fig. 3c, enol silane **54** readily reacted with HCN and was found to be fully consumed within 10 min. The reaction of the (*Z*)-enol silane reached completion within 20 h, whereas the corresponding (*E*)-enol silane, which is not actually generated during the IDPi-catalysed silyl cyanation, hardly reacted with HCN under the standard conditions, leading to an incomplete consumption of the starting material. We also analysed the reaction by variable time normalization analysis with kinetic data obtained from ^1^H NMR (Fig. 3d). When following the procedures described by Burés^36^, we found that the overall reaction is first order in catalyst^37^.

**Fig. 4:**
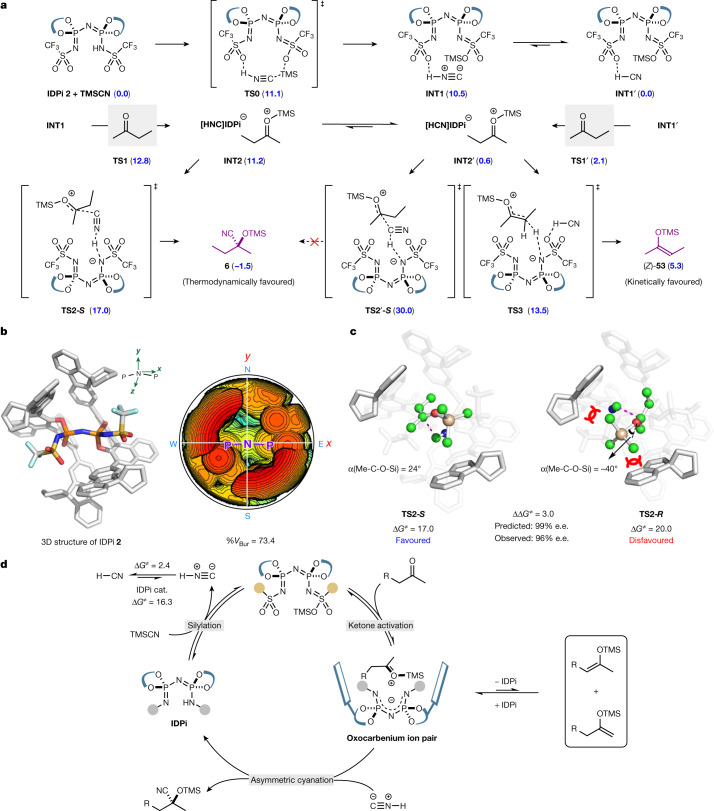
Computational studies and proposed mechanism. **a**, Possible reaction pathways of the deprotosilylation and cyanosilylation of 2-butanone with TMSCN **1** catalysed by IDPi **2**. Calculated relative free energies are indicated in parentheses and in kcal mol^−1^. **b**, Steric map of IDPi on the basis of the DFT-optimized structure of **TS2-*S***. The steric map is viewed down the *z* axis; the orientation of IDPi is indicated in the left panel. The red and blue zones indicate the more-hindered and less-hindered zones in the catalytic pocket, respectively. %*V*_Bur_, percentage of buried volume. **c**, Optimized transition state structures **TS2-*S*** and **TS2-*R***. Their activation free energies are in kcal mol^−1^. **d**, Proposed reaction mechanism.

Density functional theory (DFT) calculations^38^ were performed to study the mechanism of the deprotosilylation and cyanosilylation of 2-butanone with TMSCN catalysed by IDPi **2**. As shown in Fig. 4a, the IDPi catalyst first undergoes a reversible in situ silylation with TMSCN to generate the active catalyst **INT1** ([X-TMS][HNC], X = IDPi^−^), which has also been detected by NMR studies (see Supplementary Information Figs. S17–S20). Notably, this process affords the hydrogen isocyanide (HNC) instead of HCN (Δ*G*^≠^_TS0_ = 11.1 versus Δ*G*^≠^_TS0′_ = 38.4 kcal mol^−1^ (ref. ^39^) (Fig. 4a and Fig. S22a). The ketone substrate was then activated by the silylated catalyst (Δ*G*^≠^_TS1_ = 12.8, Fig. S22b) to form an oxocarbenium intermediate **INT2**, in which a cationic species, [TMS-2-butanone]^+^, ion pairs with the counteranion [X-HNC]^−^. Under the catalysis of IDPi, ΗΝC readily interconverts with HCN (Δ*G*^≠^_HNC→HCN_ = 2.4 and Δ*G*^≠^_HCN→HNC_ = 16.3 kcal mol^−1^, Fig. 4d and Fig. S22c), which enables the interconversions of **INT1** and **INT2** with their HCN-containing counterpart intermediates, that is **INT1′** and **INT2′**, respectively. Although the less stable tautomer HNC leads to higher energies of **INT1** and **INT2**, it is much more reactive than HCN towards the nucleophilic attack to the oxocarbenium intermediate. The calculated activation free energy for the transition state (**TS2-*S***) of cyanation with HNC is 17.0 kcal mol^−1^, which is much lower than that with HCN (30.0 kcal mol^−1^ for **TS2′-*S***) and is able to be overcome under the reaction condition. Once the activation barrier is surmounted, the silylated cyanohydrin products could be generated with the concomitant release of energy (ΔΔ*G*(**6**-**INT2**) = −12.7 kcal mol^−1^). In an alternative pathway, the enol silane product (*Z*)-**53** is readily formed by means of a facile deprotonation process (**TS3**), in line with NMR detection of enol silanes (Fig. 3a, eq. 2 and Fig. 3b). More importantly, the deprotonation step is endogonic and reversible, thus enol silane can be readily reprotonated to afford oxocarbenium intermediate **INT2′** for further conversions—for example, cyanation—which is in agreement with the experiments (Fig. 3a, eq. 2 and eq. 3). Therefore, the computational results support that the formation of enol silane is fast and reversible, and the enol silane could be reprotonated and converted to the thermodynamically favoured silylcyanohydrin product **6**.

Additionally, we computationally investigated the origin of enantioselectivity. The results indicate that **TS2-*R*** is 3.0 kcal mol^−1^ higher than **TS2-*S***, which matches the experimentally observed enantioselectivity. As depicted in Fig. 4b, the 3D structure and steric map of (*S*, *S*)-IDPi **2** shows that the N–P–N–P–N bonding, the sterically demanding Ar substituents and Tf groups constitute a confined and deep chiral pocket (the percentage of buried volume is as high as 73.4). In the lowest-energy transition states (TSs) that lead to *R* and *S* products, the oxocarbenium ion intermediate accommodates within the chiral pocket in two different orientations. In **TS2-*S***, the small methyl group is placed in the south-western quadrant and the TMS group occupies the less-hindered central region, orienting outward along the *z* axis (Fig. 4c, left), whereas **TS2-*R*** orients the bulky TMS group in the crowded south-western quadrant (Fig. 4c, right), which distorts the TMS to destroy its coplanar arrangement with the carbonyl group (α(Me-C-O-Si) = −40°). As a result, the overlapping between the p orbitals of oxygen and the carbonyl carbon is diminished, resulting in a higher energy of 1.6 kcal mol^−1^ compared with that in **TS2-*S*** (see Supplementary Information Fig. S24). Therefore, we speculate that the enantiocontrol of cyanation was enabled by the highly confined structure and steric bias of IDPi. The strong dependence of reaction outcome on the percentage of buried volume of IDPi catalysts is also supported by further DFT studies (see Supplementary Information Figs. S23, S25–S28).

On the basis of the accumulated experimental, spectroscopic and computational data, we can now propose a mechanism for the cyanosilylation of ketones enabled by IDPi catalysts. Accordingly, the initial silylation of the catalyst with TMSCN generates the silylated catalyst, which—in turn—activates the ketone to furnish a silyloxy carbenium intermediate, which can be readily deprotonated by the chiral anion of the catalyst to provide the corresponding enol silanes under the reaction conditions (Fig. 4d). We suggest this process to be merely an off-cycle phenomenon, as the enol silane can be readily reprotonated by the acidic IDPi species, affording the oxocarbenium ion intermediate, which is then captured by HNC to provide the final product, simultaneously regenerating the IDPi catalyst. The entire process features a kinetically favoured enol silane formation from the Si–H exchange reaction and the thermodynamically favoured silyl cyanohydrin formation from reaction of the silyloxocarbenium ion with HNC.

In conclusion, we show that properly designed chiral and confined acids can catalyse an asymmetric cyanosilylation reaction of both aromatic and aliphatic ketones, including the highly challenging 2-butanone. Our work can serve as an encouragement for chemists to create catalysts that rival the remarkable and sometimes extreme selectivities observed with enzymes. We also anticipate that our method could be of use in the synthesis of natural products and pharmaceuticals.

## Methods

Reaction procedure for the gram-scale catalytic asymmetric cyanosilylation of 4-phenylbutan-2-one with TMSCN **1** in the presence of 0.5 mol% IDPi **4** catalyst. TMSCN **1** (1.7 ml, 13.4 mmol, 2.0 equiv.) was placed in a 25-ml flame-dried Schlenk flask equipped with a Teflon-coated magnetic stirring bar. A solution of IDPi **4** (52.6 mg, 0.034 mmol, 0.005 equiv.) in diethyl ether (0.4 M, 16.8 ml) was added at rt. After stirring for 3.0 h at rt, the reaction mixture was cooled to −60 °C for 0.5 h, and 4-phenylbutan-2-one (1.0 g, 6.7 mmol, 1.0 equiv.) was slowly added. The resultant mixture was stirred at −60 °C for 24 h until the ketone was fully consumed (monitored by thin-layer chromatography). On completion, the reaction mixture was treated with three drops of triethylamine. Organic volatiles were evaporated in vacuo and the crude mixture was purified by column chromatography with neutral aluminium oxide (activated by 20 wt% water and washed by triethylamine) to afford the desired cyanohydrin silyl ether **23** (1.6 g, 95% yield, 95:5 e.r.).

## Online content

Any methods, additional references, Nature Research reporting summaries, source data, extended data, supplementary information, acknowledgements, peer review information; details of author contributions and competing interests; and statements of data and code availability are available at 10.1038/s41586-022-04531-5.

## Supplementary information

Supplementary Information This file contains Supplementary Sections 1–13 — see contents page for details.

## Data Availability

The experimental procedures and analytical data supporting the findings of this study are available in the manuscript and its Supplementary Information file. Raw and unprocessed NMR data are available from the corresponding author on reasonable request.
