# Supplementary material for: Organocatalytic stereoselective cyanosilylation of small ketones
Source: Nature. 2022 May 4;605(7908):84–9. doi: 10.1038/s41586-022-04531-5 (PMC9068509; doi:10.1038/s41586-022-04531-5)
Supplement: Supplementary file 1 — This file contains Supplementary Sections 1–13 — see contents page for details. [file 41586_2022_4531_MOESM1_ESM.pdf]

nature portfolio

https://doi.org/10.1038/s41586-022-04531-5

Supplementary information

Organocatalytic stereoselective cyanosilylation of small ketones

In the format provided by the authors and unedited

Supplementary Information

Organocatalytic stereoselective cyanosilylation of small ketones

Hui Zhou,[1] Yu Zhou,[2] Han Yong Bae,[1, 3] Markus Leutzsch,[1] Yihang Li,[1] Chandra Kanta De,[1] Gui-Juan Cheng*[2], and Benjamin List*[1]

[1]Max-Planck-Institut für Kohlenforschung, Kaiser-Wilhelm-Platz 1, D-45470 Mülheim an der Ruhr, Germany.

[2]Warshel Institute for Computational Biology, School of Life and Health Sciences, The Chinese University of Hong Kong, Shenzhen, Shenzhen 518172, China.

[3]Current address: Department of Chemistry, Sungkyunkwan University 2066, Seobu-ro, Jangan-gu, Suwon, 16419 (Republic of Korea).

*Email: list@kofo.mpg.de, chengguijuan@cuhk.edu.cn

Content

1. General considerations S2

2. Reaction time studies with *in situ* FT-IR spectroscopy S4

3. Development of suitable reaction conditions and catalyst identification S7

4. Substrate scope for the cyanosilylation of ketones with TMSCN S15

5. Limitations of the method S28

6. Gram-scale reaction and derivatizations of the cyanohydrin silyl ether **23** S29

7. Mechanistic studies S33

8. Characterization and study of the equilibrium between catalyst **4**-H and **4**-TMS S41

9. Preparation and characterization of imidodiphosphorimidates (IDPis) S44

10. Computational studies S49

11. References S157

12. Copies of NMR spectra S159

13. Copies of HPLC and GC traces S227

1. General considerations

Chemicals

Unless otherwise indicated, starting materials were obtained from Sigma-Aldrich, ABCR-GmbH, TCI, or Acros Co. Ltd. Moreover, all commercially available reagents were purified prior to use either by recrystallization, or by distillation under reduced pressure. For example, ketones were obtained either from commercial suppliers and used after chromatographic purification and recrystallization, or synthesized according to literature procedure.[1,2] The TMSCN **1** was obtained from commercial suppliers, and used after careful distillation. The chiral imidodiphosphorimidates (IDPis) **2**–**5** were synthesized according to literature procedures.[3,4]

Caution! TMSCN is toxic. The distillation should be conducted in a well-ventilated fume hood (65 ºC, 70 mbar).

Solvents

Solvents (Et_2O, THF, 1,4-Dioxane, Cyclohexane, CH_2Cl_2, $CHCl_3$, Benzene and Toluene) were dried by distillation from an appropriate drying agent in the technical department of the Max-Planck-Institut für Kohlenforschung and received in Schlenk flasks under argon. In addition, more solvents (MTBE, CH_3CN and Mesitylene) were purchased from commercial suppliers and dried over molecular sieves.

Inert Gas

Dry argon was purchased from Air Liquide with >99.5% purity.

Thin Layer Chromatography

Thin-layer chromatography (TLC) was performed using silica gel (Polygram SIL G/UV_{254}, 0.2 mm, with fluorescent indicator; Macherey-Nagel) or Al_2O_3 (with fluorescent indicator UV_{254}, 0.25 mm) pre-coated plastic sheets which was visualized with a UV lamp (254 nm) and/or phosphomolybdic acid (PMA) and/or 4-anisaldehyde solution and/or basic $KMnO_4$. PMA stain preparation: PMA (20 g) in EtOH (200 mL). 4-Anisaldehyde stain preparation: 4-Anisaldehyde (15 g) and conc. sulfuric acid (2.5 mL) in EtOH (250 mL). Basic $KMnO_4$ stain preparation: $KMnO_4$ (3 g), K_2CO_3 (20 g), and NaOH (5% aq., 5 mL) in water (300 mL).

Column Chromatography

Column chromatography (CC) was carried out using Merck silica gel (60 Å, 230−400 mesh, particle size 0.040−0.063 mm) or neutral Al_2O_3 (Brockmann I) purchased from Acros and activated by 20 wt % water with technical grade solvents. Elution was accelerated using compressed argon. All reported yields, unless otherwise specified, refer to spectroscopically and chromatographically pure compounds.

Nomenclature

Nomenclature follows the suggestions proposed by the computer program ChemBioDraw (12.0.3.1216) of CBD/cambridgesoft.

In situ FT-IR Spectroscopy

In situ FT-IR spectra were recorded using a METTLER TOLEDO ReactIR® 15.

Nuclear Magnetic Resonance Spectroscopy

^{1}H, ^{13}C, ^{19}F, ^{31}P Nuclear magnetic resonance (NMR) spectra for compound characterization were recorded on Bruker AVIII-500 MHz, NMR spectrometer in a suitable deuterated solvent. The solvent employed and the respective measuring frequency are indicated for each experiment. Chemical shifts are reported with tetramethylsilane (TMS) serving as a universal reference of all nuclides. The resonance multiplicity is described as s (singlet), d (doublet), t (triplet), q (quadruplet), m (multiplet), and b (broad). All spectra were recorded at 298 K, processed with MestReNova 10.0.2 suits of program, and coupling constants are reported as observed. The residual deuterated solvent signal relative to tetramethylsilane was used as the internal reference in ^{1}H NMR spectra (e.g. $CDCl_3$ = 7.26 ppm, CD_2Cl_2 = 5.32 ppm), and in ^{13}C NMR spectra (e.g. $CDCl_3$ = 77.2 ppm, CD_2Cl_2 = 53.8 ppm).[5] Signals are reported as follows: chemical shift δ in ppm (multiplicity, coupling constant *J* in Hz, number of protons). All X-nuclei spectra were acquired proton decoupled unless otherwise noted. Kinetic NMR measurements were performed at Bruker AVIII- 300/500 MHz WB NMR spectrometer. The temperature of low temperature experiments was calibrated against a 4% MeOH in MeOD-d_4 sample.[6] Further details are mentioned in the corresponding section.

Mass Spectrometry

Electrospray ionization (ESI) mass spectrometry was conducted on a Bruker ESQ 3000 spectrometer. High resolution mass spectra were determined on a Bruker APEX III FTMS (7 T magnet). The ionization method and mode of detection employed is indicated for the respective experiment and all masses are reported in atomic units per elementary charge (*m/z*) with an intensity normalized to the most intense peak.

Specific Rotations

Specific rotations ($[\boldsymbol{\alpha}]_{\mathbf{D}}^{\mathbf{T}}$) were measured with a Rudolph RA Autopol IV Automatic Polarimeter at the indicated temperature with a sodium lamp (sodium D line, λ = 589 nm). Measurements were performed in an acid resistant 1 mL cell (50 mm length) with concentrations (g/(100 mL)) reported in the corresponding solvent.

High Performance Liquid Chromatography

High performance liquid chromatography (HPLC) was performed on a Shimadzu LC-20AD liquid chromatograph SIL-20AC auto sampler, CMB-20A using Daicel columns with a chiral stationary phase. All solvents used were HPLC-grade solvents purchased from Sigma-Aldrich. The column employed and the respective solvent mixture are indicated for each experiment.

Gas Chromatography

Gas chromatography (GC) analyses on a chiral stationary phase were performed on HP 6890 and 5890 series instruments (split-mode capillary injection system, flame ionization detector (FID), hydrogen carrier gas). The conditions employed are described in detail for the individual experiments.

Abbreviations

e.r. = enantiomeric ratio, d.r. = diastereomeric ratio, TLC = thin layer chromatography, THF = tetrahydrofuran, MTBE = methyl *tert*-butyl ether, CH_3CN = acetonitrile, TMS = $SiMe_3$, Tf = SO_2CF_3, MOM = methoxymethyl ether.

2. Reaction time studies with *in situ* FT-IR spectroscopy

General procedure for the reaction monitoring by *in situ* FT-IR spectroscopy

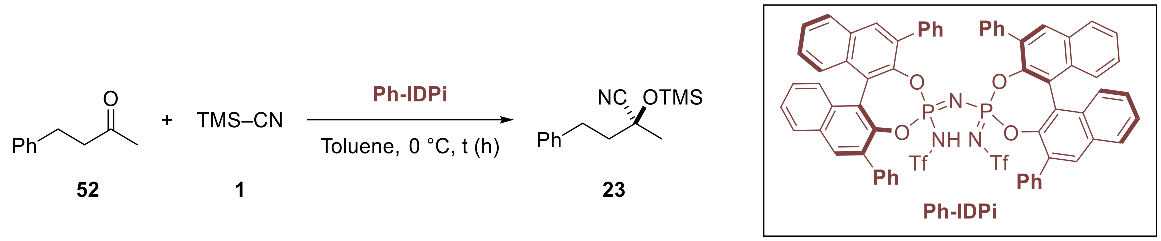

Stock solution of Ph-IDPi: Ph-IDPi (24.9 mg, 0.02 mmol) in toluene (0.25 mL, 0.08 M).

(1). TMSCN **1** (75 µL, 0.6 mmol, 1.5 equiv.), toluene (2 mL, 0.2 M), and Ph-IDPi were placed in a flask, equipped with a teflon-coated magnetic stirring bar and thermometer under argon. A probe rod of ReactIR 15 (Mettler Toledo) was dipped into the solution and the resultant solution was lowered to 0 °C for 10 min. The scan started when ketone **52** (60 µL, 0.4 mmol, 1.0 equiv.) was added to the reaction mixture. The stretching vibration absorption of the carbonyl group (1722 cm^{-1}) of ketone was monitored. Reaction with 0.5 mol% catalyst (~0.3 h dormant period, **Figure S1**); Reaction with 0.25 mol% catalyst (~3.4 h dormant period, **Figure S2**); Reaction with 0.125 mol% catalyst (~29 h dormant period, **Figure S3**)

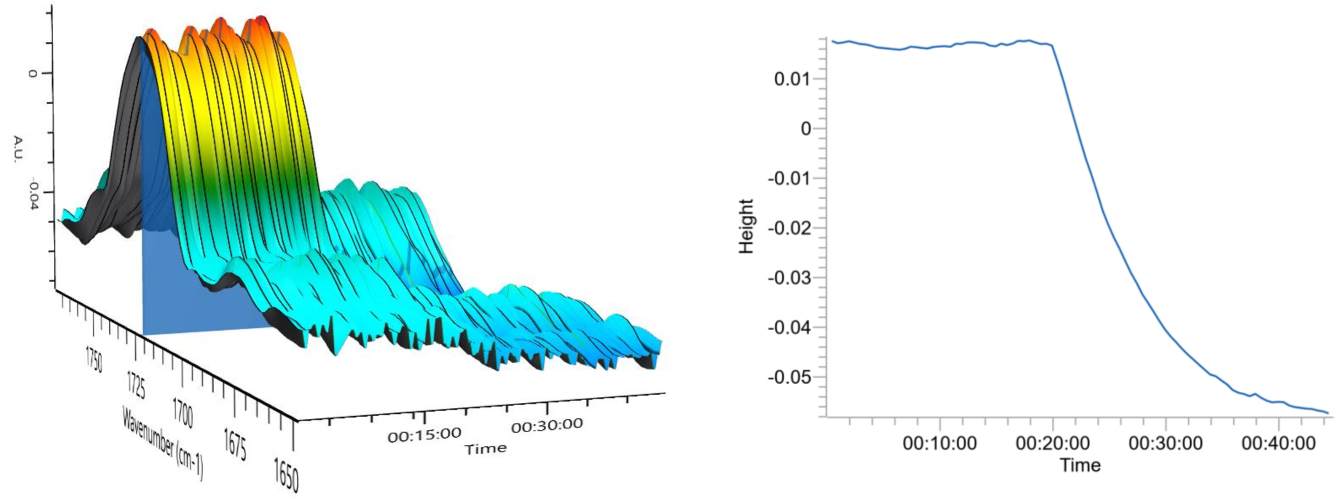

Figure S1. Reaction enabled by 0.5 mol% IDPi catalyst at 0 °C. (left: 3D spectrum); (right: trend of the reaction)

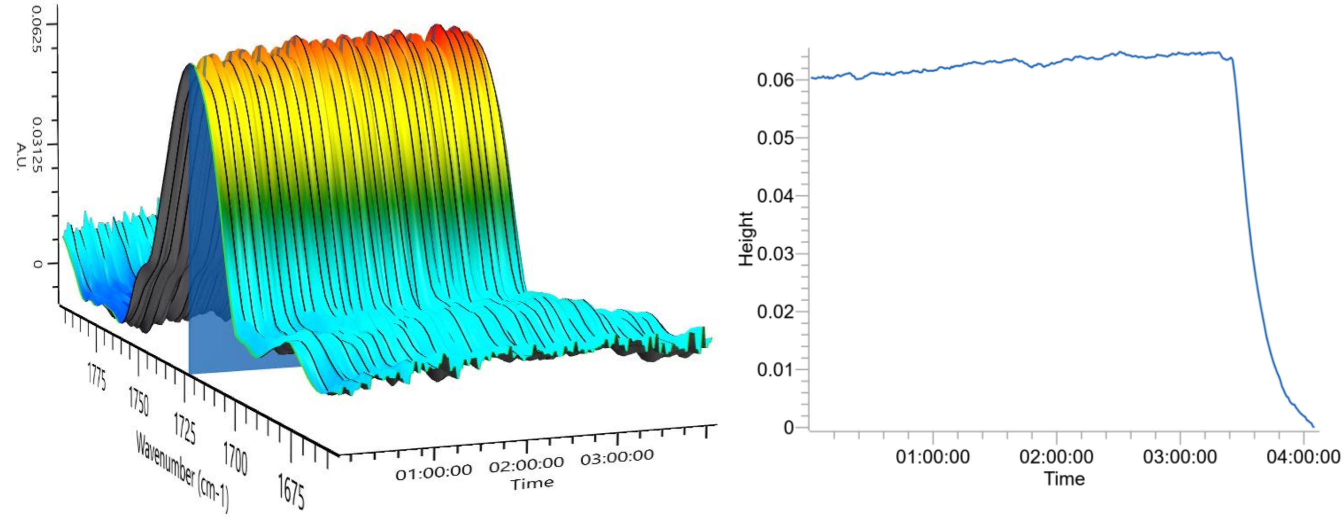

Figure S2. Reaction enabled by 0.25 mol% IDPi catalyst at 0 °C. (left: 3D spectrum); (right: trend of the reaction)

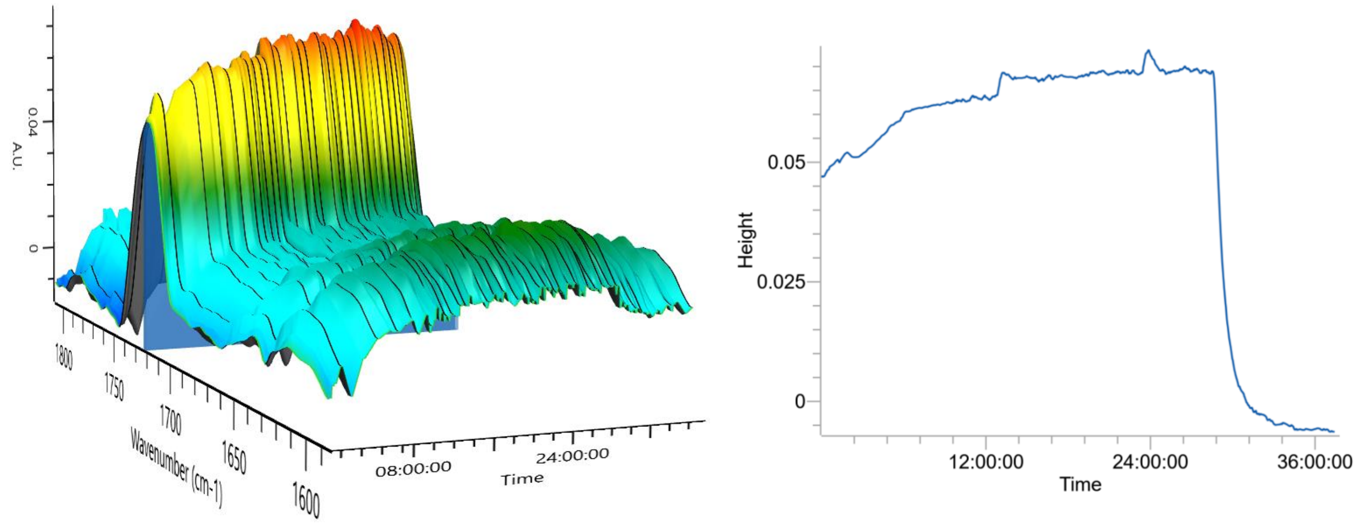

Figure S3. Reaction enabled by 0.125 mol% IDPi catalyst at at 0 °C. (left: 3D spectrum); (right: trend of the reaction)

(2). Reaction with 0.125 mol% cat at room temperature: TMSCN **1** (75 μL, 0.6 mmol, 1.5 equiv.), toluene (2 mL, 0.2 M), and Ph-IDPi (6.25 μL, 0.00125 equiv.) were placed in a flask, equipped with a teflon-coated magnetic stirring bar and thermometer under argon. A probe rod of ReactIR 15 (Mettler Toledo) was dipped into the solution at rt. The scan started when ketone **52** (60 μL, 0.4 mmol, 1.0 equiv.) was added to the reaction mixture. The stretching vibration absorption of the carbonyl group (1722 cm^{-1}) of ketone was monitored. (~3.4 h dormant period, **Figure S4**)

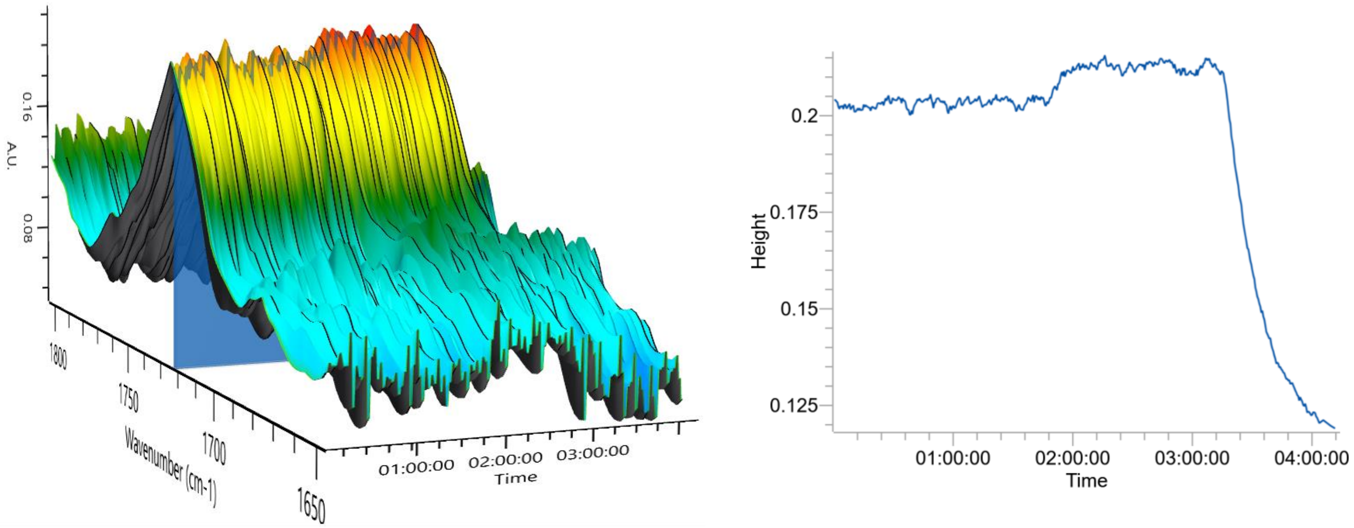

Figure S4. Reaction enabled by 0.125 mol% IDPi catalyst at rt. (left: 3D spectrum); (right: trend of the reaction)

(3). Reaction with 0.125 mol% cat pre-dried at room temperature: TMSCN **1** (75 μL, 0.6 mmol, 1.5 equiv.), toluene (2 mL, 0.2 M), and Ph-IDPi (6.25 μL, 0.00125 equiv.) were placed in a flask, equipped with a teflon-coated magnetic stirring bar and thermometer under argon. A probe rod of ReactIR 15 (Mettler Toledo) was dipped into the solution at rt and the scan started. After ~3 h, the reaction solution was lowered to 0 °C for 10 min, the ketone **52** (60 μL, 0.4 mmol, 1.0 equiv.) was added to the reaction mixture. The stretching vibration absorption of the carbonyl group (1722 cm^{-1}) of ketone was monitored. In this case, no dormant period was observed at 0 °C

and the ketone starting material was consumed significantly before the first data point of ketone was acquired. (the whole dormant period was shortened from ~29 h to ~3 h and the reaction time was shortened from ~38 h to ~4.5 h, **Figure S5**)

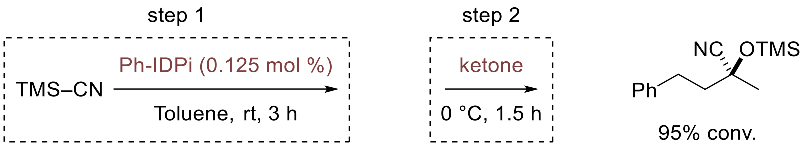

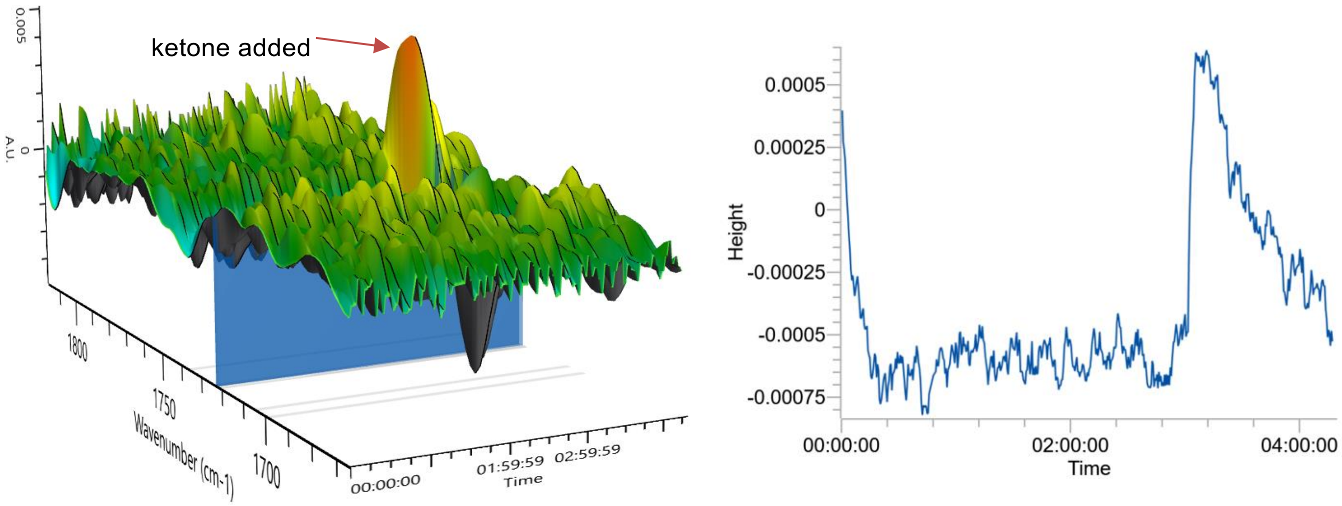

Figure S5. Reaction catalyzed by 0.125 mol% catalyst that was pre-dried at rt. (left: 3D spectrum); (right: trend of the reaction)

Conclusions: from in-situ IR experiments, we confirmed that dormant period mainly depends on the catalyst loading, temperature, and the water content. Due to variations in the water content of different solvent batches, the induced dormant periods typically were not identical. Pre-drying protocol at rt could shorten reaction time significantly especially when the catalyst loading is low and under cryogenic conditions. In general, pre-drying at rt worked very well for all reactions performed in this study.

3. Development of suitable reaction conditions and catalyst identification.

(1): General procedure for the optimization of the catalytic cyanosilylation of 4-phenylbutan-2-one 52 with TMSCN 1.

TMSCN **1** (12.5 µL, 0.1 mmol, 2.0 equiv.) was placed in a GC vial, which was equipped with a teflon-coated magnetic stirring bar. Acid catalysts (0.01 equiv.) and diethyl ether (0.2 M, 0.25 mL) were added, and the resultant solution was stirred at rt for 0.5 h and at –20 ºC for 10 min. Ketone **52** (7.5 µL, 0.05 mmol, 1.0 equiv.) was slowly added and the reaction mixture was stirred for an additional 12 h at the indicated temperature. After 12 h, the reaction was monitored by TLC (5% ethyl acetate in hexanes as eluent).

Table S1. Initial screening with commonly used Brønsted acid catalysts:

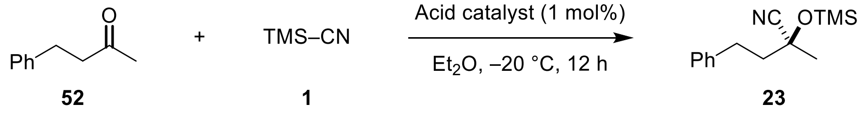

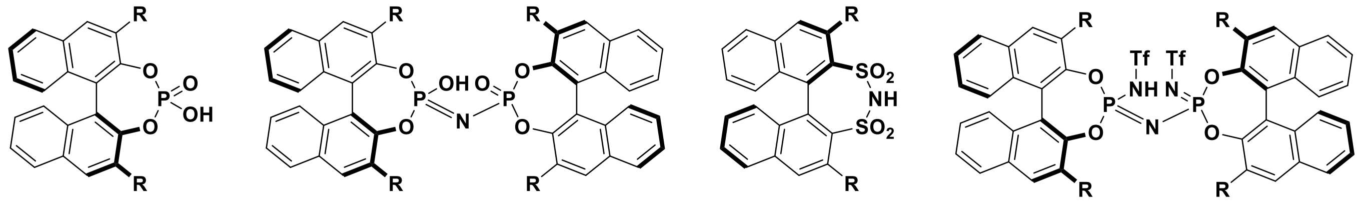

(S)-Phosphoric Acid
R = Ph
No Reaction

(S, S)-Imidodiphosphoric Acid
R = 2-isopropyl-5-methylphenyl
No Reaction

(R)-Disulfonimide
R = 3,5-$(CF_3)_2$-C_6H_3
No desired product

(S, S)-Imidodiphosphorimidate
R = Ph
Full Conv.

TMSCN **1** (12.5 µL, 0.1 mmol, 2.0 equiv.) was placed in a GC vial, which was equipped with a teflon-coated magnetic stirring bar. IDPis (0.005 equiv.) and solvent (0.2 M, 0.25 mL) were added, afterwards the resultant solution was stirred at rt for 0.5 h and at the indicated temperature for 10 min. Ketone **52** (7.5 µL, 0.05 mmol, 1.0 equiv.) was slowly added and the reaction mixture was stirred for an additional 24 h at the indicated temperature. After the ketone was fully consumed, as monitored by TLC (5% ethyl acetate in hexanes as eluent), the reaction mixture was treated with one drop of triethylamine via pipet. The enantiomeric ratio (e.r.) was determined by chiral HPLC analysis after purification by prep. TLC with aluminium oxide plates. (**Table S2**)

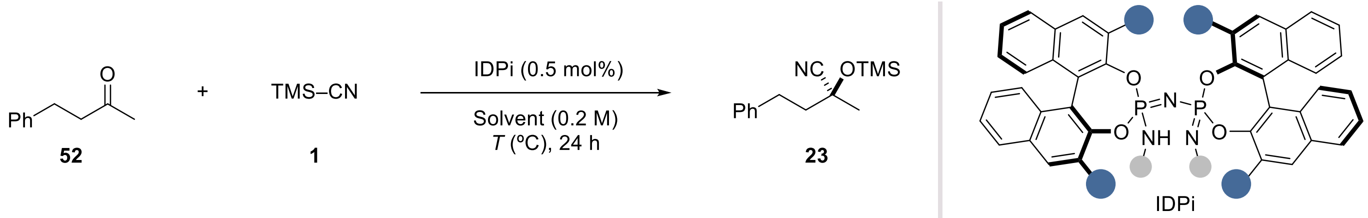

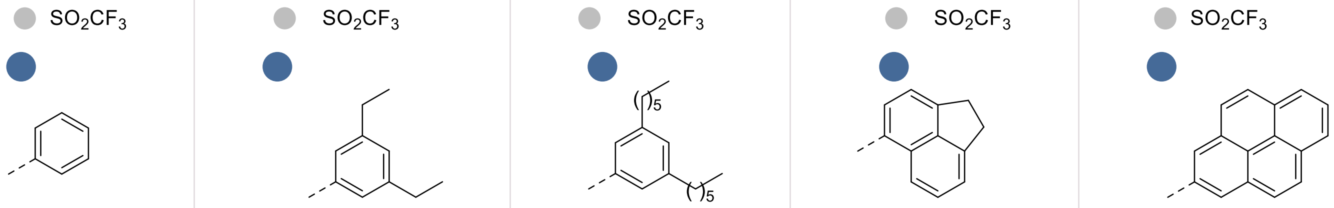

cat. S1 | cat. S2 | cat. S3 | cat. S4 | cat. S5

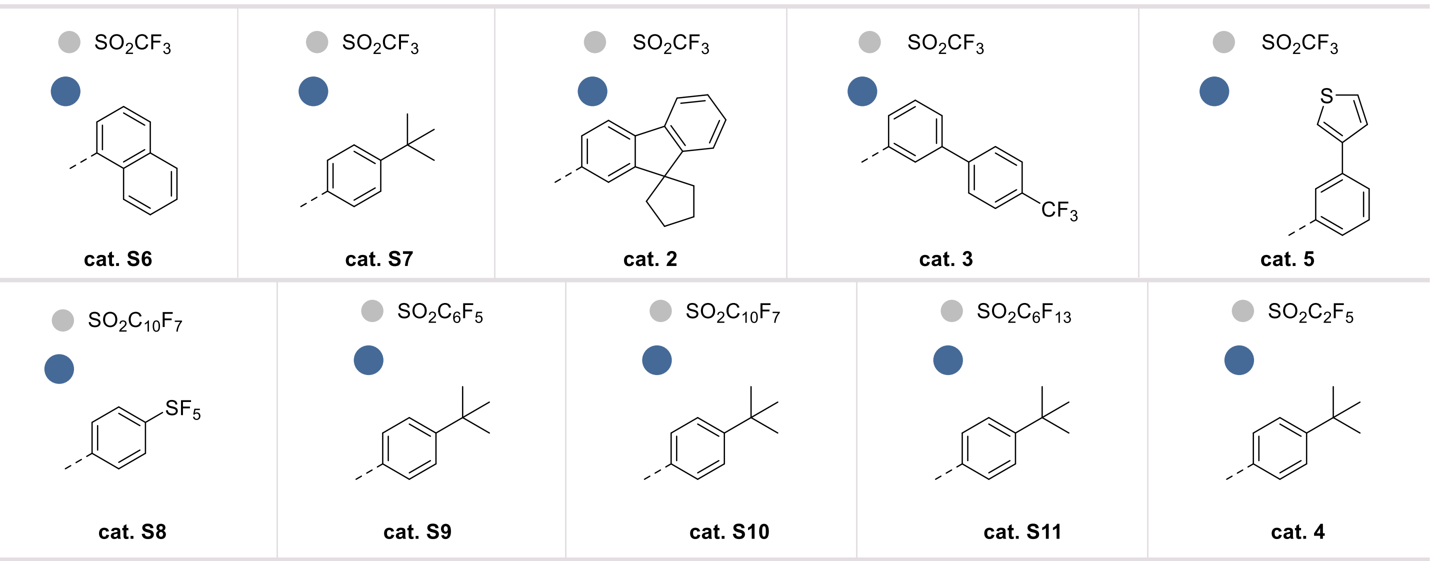

entry[a]	catalyst	solvent	T (ºC)	conv. (%)[b]	e.r.[c]
1	**cat. S1**	Et_2O	–80	>95	54:46
2	**cat. S2**	Et_2O	–80	>95	69:31
3	**cat. S3**	Et_2O	–80	>95	67:33
4	**cat. S4**	Et_2O	–80	80	73:27
5	**cat. S5**	Et_2O	25	NR	--
6	**cat. S6**	Et_2O	–80	>95	78:22
7	**cat. S6**	THF	–80	NR	--
8	**cat. S6**	CH_2Cl_2	–80	>95	54:46
9	**cat. S6**	Toluene	–80	>95	85:15
10	**cat. S7**	Toluene	–80	>95	76:24
11	**cat. 2**	Toluene	–80	>95	88:12
12	**cat. 3**	Toluene	–80	>95	74:26
13	**cat. 5**	Toluene	–80	>95	81:19
14	**cat. S8**	Toluene	–40	>95	85:15
15	**cat. S9**	Toluene	–80	NR	--
16	**cat. S10**	Toluene	–80	NR	--
17	**cat. S11**	Toluene	–80	>95	94:6
18	**cat. 4**	Toluene	–80	>95	99:1

[a]Reactions were conducted with ketone **52** (0.05 mmol), TMSCN **1** (2.0 equiv.), and IDPi catalysts (0.5 mol%) in different solvents (0.2 M) at the indicated temperature. [b]All conversions were determined by crude GC analysis or indicated by TLC analysis. [c]The enantiomeric ratio (e.r.) was determined by chiral HPLC analysis.

(2): General procedure for the optimization of the catalytic cyanosilylation of 2-butanone with TMSCN 1.

TMSCN **1** (12.5 µL, 0.1 mmol, 2.0 equiv.) was placed in a GC vial, which was equipped with a teflon-coated magnetic stirring bar. IDPi catalysts (0.005 equiv.) and solvent (0.2 M, 0.25 mL) were added, afterwards the resultant solution was stirred at rt for 0.5 h and at the indicated temperature for 10 min. 2-Butanone (4.5 µL, 0.05 mmol, 1.0 equiv.) was slowly added and the reaction mixture was stirred for an additional 12 h at the indicated temperature. After the ketone was fully consumed, as monitored by TLC, the reaction mixture was treated with one drop of triethylamine via pipet. Volatiles were removed in *vacuo* and the conversions of **6** were determined by GC

spectroscopy or indicated by TLC analysis. The enantiomeric ratio (e.r.) was determined by chiral GC analysis after purification by prep. TLC with aluminium oxide plates (visualized by treatment with basic $KMnO_4$ or 4-anisaldehyde or PMA solution). (**Table S3**)

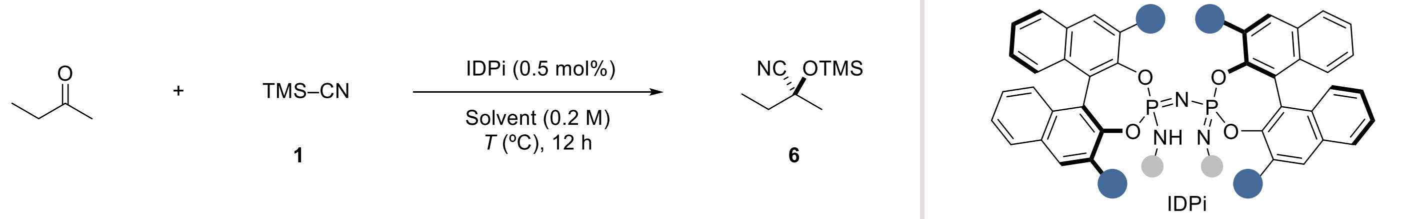

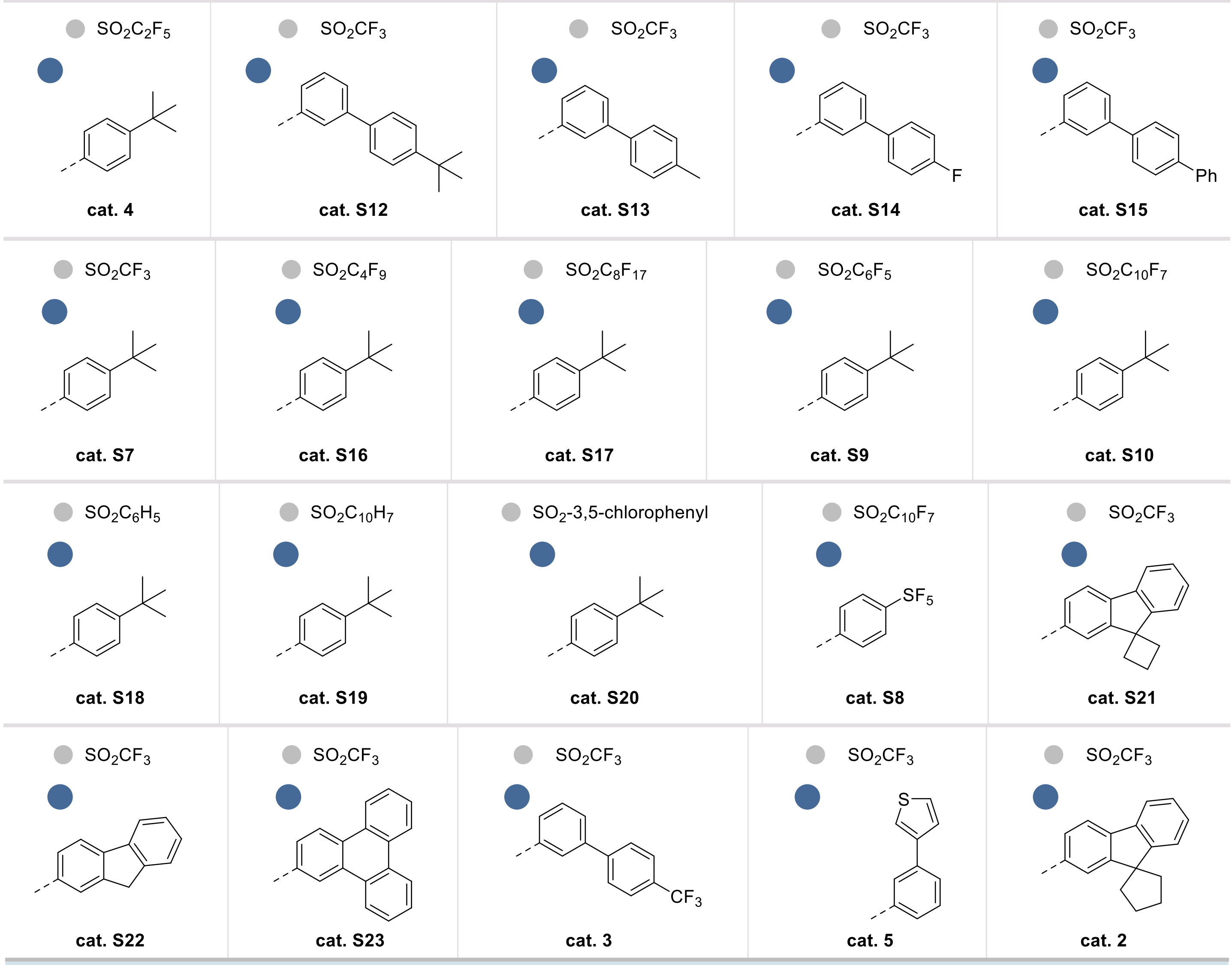

entry[a]	catalyst	solvent	T (ºC)	conv. (%)[b]	e.r.[c]
1	**cat. 4**	Toluene	–80	>95	89:11
2	**cat. 4**	Et_2O	–100	>95	87:13
3	**cat. S12**	Toluene	-80	NR	--
4	**cat. S13**	Toluene	–100	>95	84:16
5	**cat. S14**	Toluene	–100	>95	82:18
6	**cat. S15**	Toluene	–100	>95	78:22
7	**cat. S7**	Toluene	–100	>95	85:15
8	**cat. S16**	Toluene	–100	>95	85:15

9	**cat. S17**	Toluene	–100	>95	86:14
10	**cat. S9**	Toluene	–40	>95	81:19
11	**cat. S10**	Toluene	–40	>95	84:16
12	**cat. S18**	Toluene	–100	NR	--
13	**cat. S19**	Toluene	–100	NR	--
14	**cat. S20**	Toluene	–100	NR	--
15	**cat. S8**	Et_2O	–40	>95	89:11
16	**cat. S21**	Toluene	–100	>95	92:8
17	**cat. S22**	Toluene	–100	>95	65:35
18	**cat. S23**	Toluene	–100	>95	75:25
19	**cat. 3**	Et_2O	–40	>95	71:29
20	**cat. 5**	Et_2O	–40	>95	75:25
21	**cat. 2**	Et_2O	–40	93	94.5:5.5
22	**cat. 2**	Toluene	–40	>95	95:5
23	**cat. 2**	THF	–40	NR	--
24	**cat. 2**	CH_2Cl_2	–40	>95	88:12
25	**cat. 2**	Methylcyclohexane	–40	>95	96:4
26	**cat. 2**	Toluene	–80	>95	96:4
27	**cat. 2**	Toluene	–100	>95	98:2

[a]Reactions were conducted with 2-butanone (0.05 mmol), TMSCN **1** (2.0 equiv.), and IDPi catalysts (0.5 mol%) in different solvents (0.2 M) at the indicated temperature. [b]All conversions were determined by crude GC analysis or indicated by TLC analysis. [c]The enantiomeric ratio (e.r.) was determined by chiral GC analysis.

(3): General procedure for the optimization of the catalytic cyanosilylation of 5-methylhexan-2-one with TMSCN 1.

TMSCN **1** (12.5 µL, 0.1 mmol, 2.0 equiv.) was placed in a GC vial, which was equipped with a teflon-coated magnetic stirring bar. IDPi catalysts (0.005 equiv.) and solvent (0.2 M, 0.25 mL) were added, afterwards the resultant solution was stirred at rt for 0.5 h and at the indicated temperature for 10 min. 5-Methylhexan-2-one (7.0 µL, 0.05 mmol, 1.0 equiv.) was slowly added and the reaction mixture was stirred for an additional 24 h at the indicated temperature. After the ketone was fully consumed, as monitored by TLC, the reaction mixture was treated with one drop of triethylamine via pipet. The conversions were determined by GC spectroscopy or indicated by TLC analysis. The enantiomeric ratio (e.r.) was determined by chiral GC analysis after purification by prep. TLC with aluminium oxide plates (visualized by treatment with basic $KMnO_4$ or 4-anisaldehyde or PMA solution). (**Table S4**)

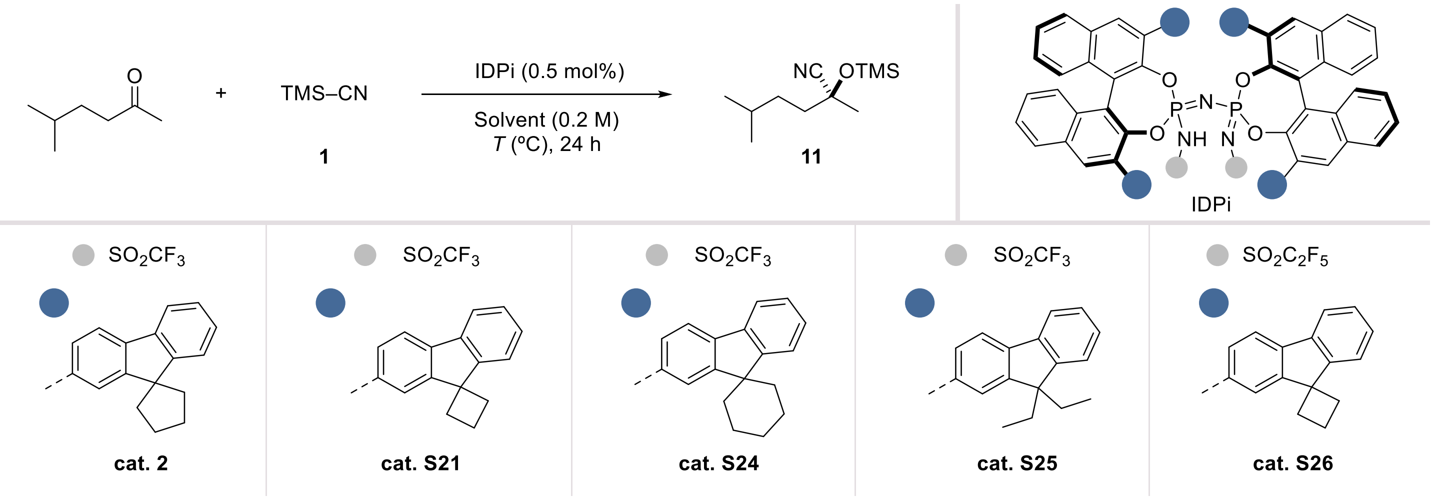

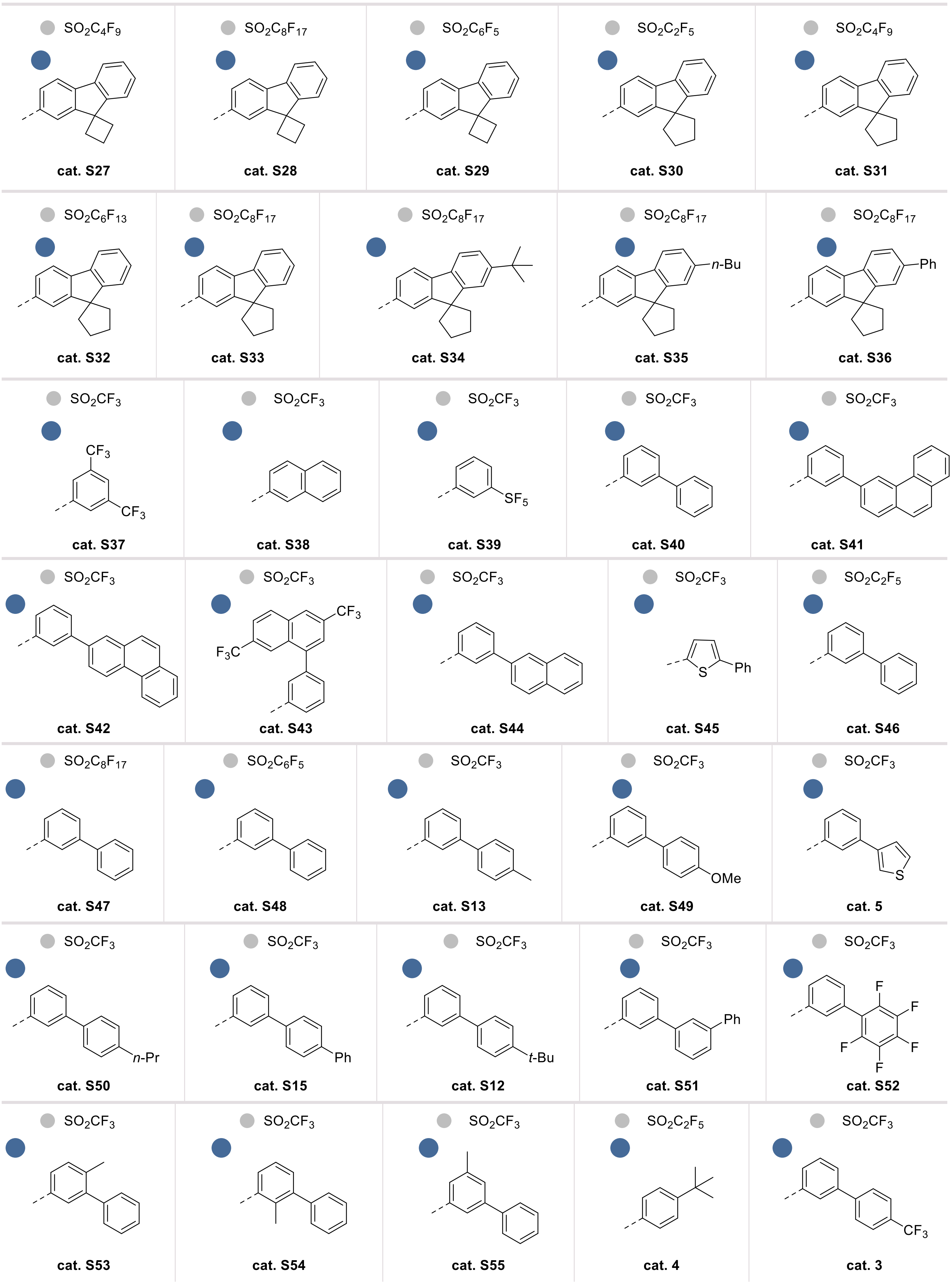

SO2C4F9
cat. S27
SO2C8F17
cat. S28
SO2C6F5
cat. S29
SO2C2F5
cat. S30
SO2C4F9
cat. S31
SO2C6F13
cat. S32
SO2C8F17
cat. S33
SO2C8F17
cat. S34
SO2C8F17
n-Bu
cat. S35
SO2C8F17
Ph
cat. S36
SO2CF3
CF3
CF3
cat. S37
SO2CF3
cat. S38
SO2CF3
SF5
cat. S39
SO2CF3
cat. S40
SO2CF3
cat. S41
SO2CF3
cat. S42
SO2CF3
CF3
F3C
cat. S43
SO2CF3
cat. S44
SO2CF3
S
Ph
cat. S45
SO2C2F5
cat. S46
SO2C8F17
cat. S47
SO2C6F5
cat. S48
SO2CF3
cat. S13
SO2CF3
OMe
cat. S49
SO2CF3
S
cat. 5
SO2CF3
n-Pr
cat. S50
SO2CF3
Ph
cat. S15
SO2CF3
t-Bu
cat. S12
SO2CF3
Ph
cat. S51
SO2CF3
F
F
F
F
F
cat. S52
SO2CF3
cat. S53
SO2CF3
cat. S54
SO2CF3
cat. S55
SO2C2F5
cat. 4
SO2CF3
CF3
cat. 3

entry[a]	catalyst	solvent	T (ºC)	conv. (%)[b]	e.r.[c]
1	**cat. 2**	Toluene	–80	>95	73:27
2	**cat. S21**	Toluene	–80	>95	84:16
3	**cat. S24**	Toluene	–80	NR	52:48
4	**cat. S25**	Toluene	–80	>95	50.5:49.5
5	**cat. S26**	Toluene	–80	>95	75:25
6	**cat. S27**	Toluene	–80	>95	81:19
7	**cat. S28**	Toluene	–80	>95	76:24
8	**cat. S29**	Toluene	–40	NR	--
9	**cat. S30**	Toluene	–40	>95	71:29
10	**cat. S31**	Toluene	–80	>95	62.5:37.5
11	**cat. S32**	Toluene	–80	>95	55:45
12	**cat. S33**	Toluene	–80	>95	76:24
13	**cat. S34**	Toluene	–80	>95	73:27
14	**cat. S35**	Toluene	–80	>95	78:22
15	**cat. S36**	Toluene	–80	>95	67:33
16	**cat. S37**	Toluene	–80	>95	70:30
17	**cat. S38**	Toluene	–80	>95	75:25
18	**cat. S39**	Toluene	–80	>95	64:36
19	**cat. S40**	Et_2O	–80	>95	71:29
20	**cat. S40**	Toluene	–80	>95	79:21
21	**cat. S40**	THF	–80	NR	--
22	**cat. S41**	Toluene	–80	>95	77:23
23	**cat. S42**	Toluene	–80	>95	72:28
24	**cat. S43**	Toluene	–80	>95	64:36
25	**cat. S44**	Toluene	–80	>95	79:21
26	**cat. S45**	Toluene	–80	>95	71:29
27	**cat. S46**	Toluene	–80	>95	72:28
28	**cat. S47**	Toluene	–10	NR	--
29	**cat. S48**	Toluene	–40	<50	76:24
30	**cat. S13**	Toluene	–80	>95	78:22
31	**cat. S49**	Toluene	–40	>95	72:28
32	**cat. 5**	Toluene	–80	>95	78:22
33	**cat. S50**	Toluene	–40	>95	69:31
34	**cat. S15**	Toluene	–80	>95	85:15
35	**cat. S12**	Toluene	–40	>95	78:22
36	**cat. S51**	Toluene	–40	>95	66.5:33.5
37	**cat. S52**	Toluene	–80	>95	74:26
38	**cat. S53**	Toluene	–40	>95	67:33
39	**cat. S54**	Toluene	–80	>95	86:14
40	**cat. S55**	Toluene	–40	>95	78:22
41	**cat. 4**	Toluene	–80	>95	51:49
42	**cat. 3**	Toluene	–80	>95	91:9
43	**cat. 3**	Toluene	–100	>95	95:5

[a]Reactions were conducted with 5-methylhexan-2-one (0.05 mmol), TMSCN **1** (2.0 equiv.), and IDPi catalysts (0.5 mol%) in different solvents (0.2 M) at the indicated temperature. [b]All conversions were determined by crude GC analysis or indicated by TLC analysis. [c]The enantiomeric ratio (e.r.) was determined by chiral GC analysis.

(4): General procedure for the optimization of the catalytic cyanosilylation of acetophenone with TMSCN 1.

TMSCN **1** (12.5 µL, 0.1 mmol, 2.0 equiv.) was placed in a GC vial, which was equipped with a teflon-coated magnetic stirring bar. IDPi catalysts (0.01 equiv.) and solvent (0.2 M, 0.25 mL) were added, afterwards the resultant solution was stirred at rt for 0.5 h and at the indicated temperature for 10 min. Acetophenone (6.0 µL, 0.05 mmol, 1.0 equiv.) was slowly added and the reaction mixture was stirred for an additional 24 h at the indicated temperature. After 48 h, the reaction mixture was treated with one drop of triethylamine via pipet. The conversions were determined by TLC or crude ^{1}H NMR analysis. The enantiomeric ratio (e.r.) was determined by chiral HPLC analysis after purification by prep. TLC with aluminium oxide plates. (**Table S5**)

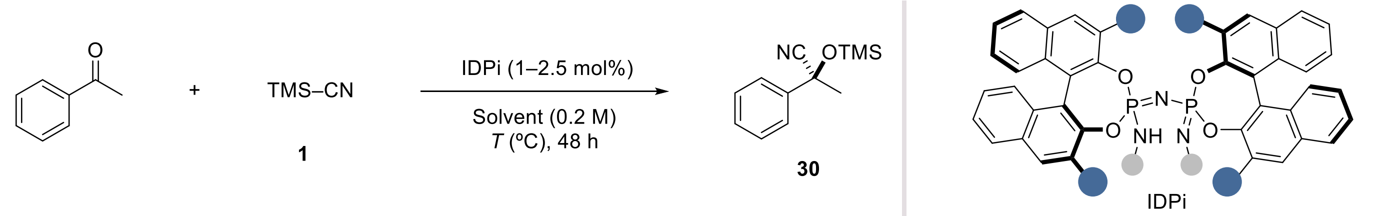

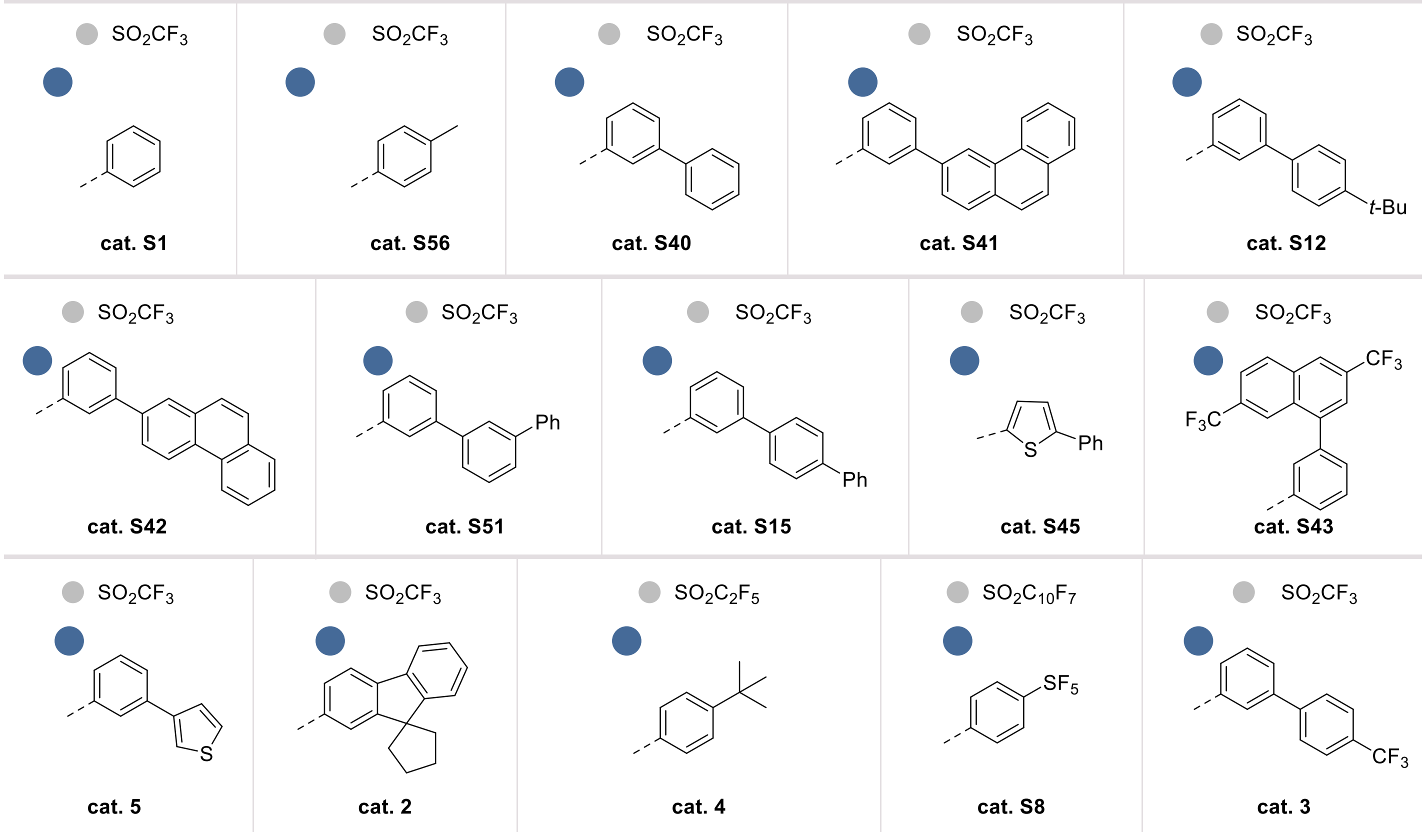

entry[a]	catalyst	solvent	T (ºC)	conv. (%)[b]	e.r.[c]
1	**cat. S1**	Toluene	–20	>95	50:50
2	**cat. S56**	Toluene	–20	>95	55:45
3	**cat. S40**	Toluene	–20	>95	66:34
4	**cat. S41**	Toluene	–80	>95	68:32
5	**cat. S12**	Toluene	–40	>95	66:34
6	**cat. S42**	Toluene	–80	>95	84:16
7	**cat. S51**	Toluene	–80	>95	85:15
8	**cat. S15**	Toluene	–80	>95	68:32
9	**cat. S45**	Toluene	–80	>95	65:35
10	**cat. S43**	Toluene	–80	>95	77.5:22.5
11	**cat. 5**	Toluene	–80	>95	89:11
12	**cat. 5**	Toluene	–100	80[e]	91.5:8.5

13	**cat. 5**	Et_2O	−100	83[e]	96:4
14[d]	**cat. 5**	Et_2O	−100	>95	96:4
15	**cat. 2**	Et_2O	−80	39[e]	68:32
16	**cat. 4**	Et_2O	−80	38[e]	62:38
17	**cat. S8**	Et_2O	−40	70[e]	52:48
18	**cat. 3**	Et_2O	−100	49[e]	64:36

[a]Reactions were conducted with acetophenone (0.05 mmol), TMSCN **1** (2.0 equiv.), and IDPi catalysts (1 mol%) in different solvents (0.2 M) at the indicated temperature. [b]Conversions were determined by crude GC analysis or indicated by TLC analysis. [c]The enantiomeric ratio (e.r.) was determined by chiral HPLC analysis. [d]With 5 mol% catalyst. [e]With 2.5 mol% catalysts and NMR yields determined by crude ^1H NMR analysis using CH_2Br_2 as internal standard.

(5): General procedure for the optimization of the catalytic cyanosilylation of 3,3-dimethylcyclohexan-1-one with TMSCN 1.

TMSCN **1** (12.5 µL, 0.1 mmol, 2.0 equiv.) was placed in a GC vial, which was equipped with a teflon-coated magnetic stirring bar. IDPi catalysts (0.025 equiv.) and toluene (0.2 M, 0.25 mL) were added, afterwards the resultant solution was stirred at rt for 0.5 h and at −80 °C for 10 min. 3,3-Dimethylcyclohexan-1-one (7.0 µL, 0.05 mmol, 1.0 equiv.) was slowly added and the reaction mixture was stirred for an additional 24 h at the indicated temperature. The reaction mixture was treated with one drop of triethylamine via pipet. The conversions were determined by GC or TLC analysis. The enantiomeric ratio (e.r.) was determined by chiral GC analysis. (**Table S6**)

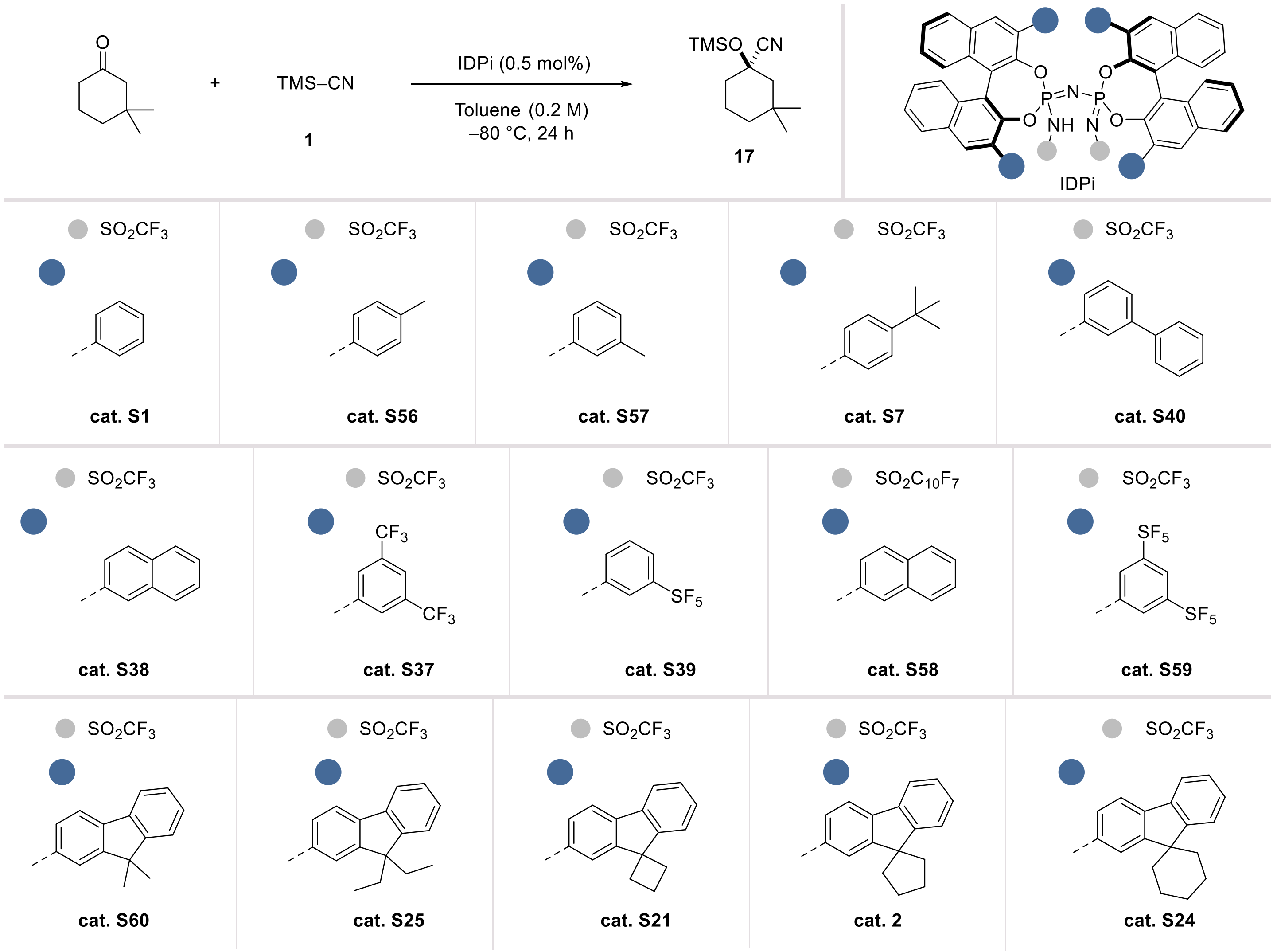

entry[a]	catalyst	solvent	T (°C)	conv. (%)[b]	e.r.[c]
1	**cat. S1**	Toluene	–80	>95	51:49
2	**cat. S56**	Toluene	–80	>95	52:48
3	**cat. S57**	Toluene	–80	>95	54:46
4	**cat. S7**	Et_2O	–40	>95	51:49
5	**cat. S40**	Et_2O	–40	>95	58:42
6	**cat. S38**	Et_2O	–40	>95	57:43
7	**cat. S37**	Et_2O	–40	>95	58:42
8	**cat. S39**	Toluene	–80	>95	60:40
9	**cat. S58**	Toluene	–80	>95	79:21
10	**cat. S59**	Toluene	–80	>95	57:43
11	**cat. S60**	Toluene	–80	>95	94:6
12	**cat. S25**	Toluene	–80	>95	60:40
13	**cat. S21**	Toluene	–80	>95	90:10
14	**cat. 2**	Toluene	–80	>95	96:4
15	**cat. S24**	Toluene	–80	>95	93:7

[a]Reactions were conducted with 3,3-dimethylcyclohexan-1-one (0.05 mmol), TMSCN **1** (2.0 equiv.), and IDPi catalysts (0.5 mol%) in toluene (0.2 M) at –80 °C. [b]All conversions were determined by crude GC analysis or indicated by TLC analysis. [c]The enantiomeric ratio (e.r.) was determined by chiral GC analysis.

Note: In all cases, unless otherwise mentioned, the reactions were quite clean and full conversion of starting ketones was observed. In some cases, the opposite enantiomer was obtained as major product; to unify the reported results, the depicted e.r. did not reflect the HPLC traces.

4. Substrate scope for the cyanosilylation of ketones with TMSCN

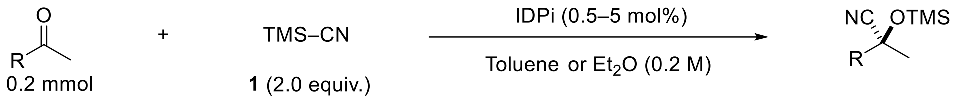

General procedure for the catalytic asymmetric cyanosilylation of ketones with TMSCN.

TMSCN **1** (50.0 μL, 0.4 mmol, 2.0 equiv.) was placed in a GC vial equipped with a teflon-coated magnetic stirring bar. IDPi **2–5** (0.005 –0.05 equiv.) in toluene or diethylether (1 mL, 0.2 M) was added at rt and stirred for 0.5 h. The resultant mixture was cooled to the indicated temperature (–80 °C to –100 °C) for 10 min, subsequently ketones (0.2 mmol, 1.0 equiv.) were slowly added, and the reaction was stirred for 24 h or 5 d at the indicated temperature. The reactions were treated with one drop of triethylamine, and directly purified by column chromatography with diethyl ether and pentane as eluent.

Note: the desired cyanohydrin trimethylsilyl ether products were purified by column chromatography using neutral Al_2O_3 (Brockmann I) activated by 20 wt % water and washed by triethylamine.

Analytical data of products

(*S*)-2-methyl-2-((trimethylsilyl)oxy)butanenitrile 6

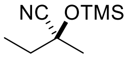

The titled product was purified by column chromatography to afford **6** as colorless oil (31.2 mg, 91% yield).

^{1}H NMR (501 MHz, CD_2Cl_2) δ 1.78–1.72 (m, 2H), δ 1.55 (s, 3H), 1.04 (t, J = 7.4 Hz, 3H), 0.23 (s, 9H).

^{13}C NMR (126 MHz, CD_2Cl_2) δ 122.4, 70.8, 36.8, 28.6, 8.8, 1.3.

R_f = 0.54 (Hexanes).

ESI-HRMS (*m/z*): calculated for $C_8H_{17}N_1O_1Si_1Na_1$ ($[M+Na]^+$): 194.0972, found: 194.0970.

GC (30.0 m BGB 176, injection temperature: 220 °C, 40 °C iso 35 min, 0.6 bar H_2): t_{R1} = 29.9 min (major), t_{R2} = 28.5 min (minor), e.r. = 98:2.

$[\alpha]_D^{25}$ = –13.8 (*c* 0.42, $CHCl_3$).

2-methyl-2-((trimethylsilyl)oxy)pentanenitrile 7

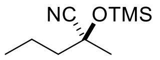

The titled product was purified by column chromatography to afford **7** as colorless oil (35.2 mg, 95% yield).

^1H NMR (501 MHz, CD_2Cl_2) δ 1.75–1.64 (m, 2H), 1.56 (s, 3H), 1.52–1.44 (m, 2H), 0.96 (t, *J* = 7.3 Hz, 3H), 0.22 (s, 9H).

^{13}C NMR (126 MHz, CD_2Cl_2) δ 122.6, 70.2, 45.9, 29.2, 18.1, 14.0, 1.4.

R_f = 0.37 (Ethyl acetate/hexanes = 1:19).

EI-HRMS (*m/z*): calculated for $C_9H_{19}N_1O_1Si_1$ $[M^{+\bullet}]$: 185.1230, found: 185.1227.

GC (30.0 m BGB 176, injection temperature: 220 °C, 60 °C iso 60 min, 0.5 bar H_2): t_{R1} = 70.1 min (major), t_{R2} = 67.2 min (minor), e.r. = 95:5.

$[\alpha]_D^{25}$ = –10.8 (*c* 0.50, $CHCl_3$). Lit[7] (80% *ee*) $[\alpha]_D^{22}$ = –0.9 (*c* 1.6, CH_2Cl_2).

2-methyl-2-((trimethylsilyl)oxy)hexanenitrile 8

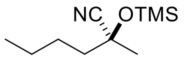

The titled product was purified by column chromatography to afford **8** as colorless oil (38.3 mg, 96% yield).

^1H NMR (501 MHz, CD_2Cl_2) δ 1.71 (ddd, *J* = 10.7, 8.6, 5.1 Hz, 2H), 1.56 (s, 3H), 1.54–1.41 (m, 2H), 1.40–1.31 (m, 2H), 0.93 (t, *J* = 7.2 Hz, 3H), 0.23 (s, 9H).

^{13}C NMR (126 MHz, CD_2Cl_2) δ 122.6, 70.2, 43.5, 29.1, 26.8, 22.9, 14.1, 1.4.

R_f = 0.43 (Ethyl acetate/hexanes = 1:19).

EI-HRMS (*m/z*): calculated for $C_{10}H_{21}N_1O_1Si_1$ $[M^{+\bullet}]$: 199.1386, found: 199.1384.

GC (30.0 m BGB 176, injection temperature: 220 °C, 80 °C iso 140 min, 0.5 bar H_2): t_{R1} = 86.4 min (major), t_{R2} = 83.3 min (minor), e.r. = 98:2.

$[\alpha]_D^{25}$ = –15.6 (*c* 0.87, $CHCl_3$). Lit[8] (92% *ee*) $[\alpha]_D^{25}$ = –13.9 (*c* 0.95, $CHCl_3$).

2-methyl-2-((trimethylsilyl)oxy)heptanenitrile 9

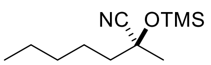

The titled product was purified by column chromatography to afford **9** as colorless oil (38.4 mg, 90% yield).

^1H NMR (501 MHz, CD_2Cl_2) δ 1.76–1.64 (m, 2H), 1.56 (s, 3H), 1.54–1.39 (m, 2H), 1.33 (dt, *J* = 7.0, 3.4 Hz, 4H), 0.91 (t, *J* = 7.0 Hz, 3H), 0.23 (s, 9H).

^{13}C NMR (126 MHz, CD_2Cl_2) δ 122.7, 70.3, 43.7, 31.9, 29.1, 24.4, 22.9, 14.1, 1.4.

R_f = 0.68 (Ethyl acetate/hexanes = 1:9).

ESI-HRMS (*m/z*): calculated for $C_{11}H_{23}N_1O_1Si_1Na_1$ ($[M+Na]^+$): 236.1441, found: 236.1443.

GC (30.0 m BGB 176, injection temperature: 220 °C, 80 °C iso 80 min, 0.5 bar H_2): t_{R1} = 64.6 min (major), t_{R2} = 62.7 min (minor), e.r. = 96:4.

$[\alpha]_D^{25}$ = −14.8 (*c* 1.08, $CHCl_3$). Lit[9] (51% *ee*) $[\alpha]_D^{25}$ = −8.2 (*c* 1.86, CH_2Cl_2).

2,4-dimethyl-2-((trimethylsilyl)oxy)pentanenitrile 10

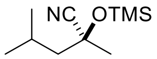

The titled product was purified by column chromatography to afford **10** as colorless oil (37.1 mg, 93% yield).

^{1}H NMR (501 MHz, CD_2Cl_2) δ 1.93 (dp, *J* = 13.1, 6.5 Hz, 1H), 1.72–1.59 (m, 2H), 1.58 (s, 3H), 1.00 (dd, *J* = 6.7, 4.5 Hz, 6H), 0.23 (s, 9H).

^{13}C NMR (126 MHz, CD_2Cl_2) δ 123.0, 69.7, 51.9, 30.0, 25.4, 24.0, 23.9, 1.4.

R_f = 0.56 (Ethyl acetate/hexanes = 1:19).

ESI-HRMS (*m/z*): calculated for $C_{10}H_{21}N_1O_1Si_1Na_1$ ($[M+Na]^+$): 222.1285, found: 222.1284.

GC (30.0 m G-TA, injection temperature: 220 °C, 65 °C iso 20 min, 6 °C/min, 180 °C iso 3 min, 0.5 bar H_2): t_{R1} = 14.9 min (major), t_{R2} = 12.7 min (minor), e.r. = 93:7.

$[\alpha]_D^{25}$ = −10.2 (*c* 1.0, $CHCl_3$).

2,5-dimethyl-2-((trimethylsilyl)oxy)hexanenitrile 11

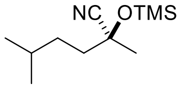

The titled product was purified by column chromatography to afford **11** as colorless oil (41.4 mg, 97% yield).

^{1}H NMR (501 MHz, CD_2Cl_2) δ 1.76–1.65 (m, 2H), 1.56 (s, 3H), 1.40 (ddd, *J* = 11.7, 6.6, 5.1 Hz, 1H), 1.37–1.27 (m, 2H), 0.91 (d, *J* = 6.6 Hz, 6H), 0.23 (s, 9H).

^{13}C NMR (126 MHz, CD_2Cl_2) δ 122.6, 70.4, 41.7, 33.6, 29.1, 28.4, 22.7, 22.6, 1.4.

R_f = 0.32 (Ethyl acetate/hexanes = 1:19).

ESI-HRMS (*m/z*): calculated for $C_{11}H_{23}N_1O_1Si_1Na_1$ ($[M+Na]^+$): 236.1441, found: 236.1439.

GC (25.0 m Hydrodex-gamma TBDAc-CD, injection temperature: 220 °C, 65 °C iso 40 min, 8 °C/min, 230 °C iso 3 min, 0.5 bar H_2): t_{R1} = 31.9 min (major), t_{R2} = 28.9 min (minor), e.r. = 95:5.

$[\alpha]_D^{25}$ = −13.6 (*c* 1.04, $CHCl_3$). Lit[8] (92% *ee*)$[\alpha]_D^{25}$ = −12.5 (*c* 0.95, $CHCl_3$).

3-cyclohexyl-2-methyl-2-((trimethylsilyl)oxy)propanenitrile 12

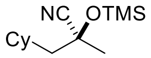

The titled product was purified by column chromatography to afford **12** as colorless oil (45.9 mg, 96% yield).

^{1}H NMR (501 MHz, CD_2Cl_2) δ 1.86–1.80 (m, 2H), 1.73–1.59 (m, 6H), 1.57 (s, 3H), 1.35–1.24 (m, 2H), 1.22–1.13 (m, 1H), 1.08–0.93 (m, 2H), 0.23 (s, 9H).

^{13}C NMR (126 MHz, CD_2Cl_2) δ 123.1, 69.7, 50.7, 34.8, 34.7, 34.6, 30.0, 26.6, 1.4.

R_f = 0.31 (Ethyl acetate/hexanes = 1:19).

EI-HRMS (*m/z*): calculated for $C_{13}H_{25}N_1O_1Si_1$ $[M^{+\bullet}]$: 239.1700, found: 239.1696.

GC (30.0 m BGB 176, injection temperature: 220 °C, 90 °C iso 100 min, 6 °C/min, 230 °C iso 3 min, 0.5 bar H_2): t_{R1} = 90.1 min (major), t_{R2} = 88.7 min (minor), e.r. = 96:4.

$[\alpha]_D^{25}$ = –15.0 (*c* 1.05, $CHCl_3$).

5-chloro-2-methyl-2-((trimethylsilyl)oxy)pentanenitrile 13

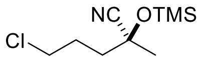

The titled product was purified by column chromatography to afford **13** as colorless oil (41.3 mg, 94% yield).

^{1}H NMR (501 MHz, CD_2Cl_2) δ 3.61 (ddd, *J* = 6.8, 6.0, 2.6 Hz, 2H), 2.10–1.91 (m, 2H), 1.90–1.84 (m, 2H), 1.60 (s, 3H), 0.24 (s, 9H).

^{13}C NMR (126 MHz, CD_2Cl_2) δ 122.2, 69.7, 45.1, 41.1, 29.2, 28.0, 1.3.

R$_f$ = 0.32 (Ethyl acetate/hexanes = 1:19).

ESI-HRMS (*m/z*): calculated for $C_9H_{18}Cl_1N_1O_1Si_1Na_1$ ($[M+Na]^+$): 242.0738, found: 242.0740.

GC (30.0 m G-TA, injection temperature: 220 °C, 75 °C iso 80 min, 0.5 bar H_2): t_{R1} = 61.5 min (major), t_{R2} = 60.0 min (minor), e.r. = 95:5.

$[\alpha]_D^{25}$ = –15.9 (*c* 1.02, $CHCl_3$).

6-chloro-2-methyl-2-((trimethylsilyl)oxy)hexanenitrile 14

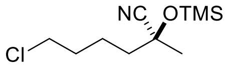

The titled product was purified by column chromatography to afford **14** as colorless oil (44.4 mg, 95% yield).

^{1}H NMR (501 MHz, CD_2Cl_2) δ 3.57 (t, *J* = 6.6 Hz, 2H), 1.86–1.79 (m, 2H), 1.77–1.71 (m, 2H), 1.71–1.59 (m, 2H), 1.58 (s, 3H), 0.23 (s, 9H).

^{13}C NMR (126 MHz, CD_2Cl_2) δ 122.4, 70.0, 45.2, 42.9, 32.6, 29.1, 22.2, 1.3.

R$_f$ = 0.43 (Ethyl acetate/hexanes = 1:19).

ESI-HRMS (*m/z*): calculated for $C_{10}H_{20}Cl_1N_1O_1Si_1Na_1$ ($[M+Na]^+$): 256.0895, found: 256.0896.

GC (30.0 m G-TA, injection temperature: 220 °C, 100 °C iso 30 min, 8 °C/min, 180 °C iso 3 min, 0.5 bar H_2): t_{R1} = 24.4 min (major), t_{R2} = 22.5 min (minor), e.r. = 95:5.

$[\alpha]_D^{25}$ = –16.1 (*c* 0.51, $CHCl_3$).

2,6-dimethyl-2-((trimethylsilyl)oxy)hept-5-enenitrile 15

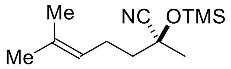

The titled product was purified by column chromatography to afford **15** as colorless oil (41.5 mg, 92% yield).

^{1}H NMR (501 MHz, CD_2Cl_2) δ 5.14–5.08 (m, 1H), 2.28–2.05 (m, 2H), 1.73 (ddd, *J* = 10.4, 6.1, 3.9 Hz, 2H), 1.69 (d, *J* = 1.5 Hz, 3H), 1.63 (d, *J* = 1.3 Hz, 3H), 1.57 (s, 3H), 0.23 (s, 9H).

^{13}C NMR δ 133.1, 123.0, 122.5, 70.0, 43.7, 29.2, 25.8, 23.5, 17.8, 1.4.

R$_f$ = 0.54 (Ethyl acetate/hexanes = 1:19).

ESI-HRMS (*m/z*): calculated for $C_{12}H_{23}N_1O_1Si_1Na_1$ ($[M+Na]^+$): 248.1441, found: 248.1442.

GC (30.0 m BGB 176, injection temperature: 220 °C, 85 °C iso 55 min, 8 °C/min, 220 °C iso 3 min, 0.5 bar H_2): t_{R1} = 46.7 min (major), t_{R2} = 48.0 min (minor), e.r. = 92.5:7.5.

$[\alpha]_D^{25}$ = 10.8 (*c* 1.0, $CHCl_3$). Lit[8] (90% *ee*) $[\alpha]_D^{25}$ = –14.4 (*c* 0.89, $CHCl_3$).

4-((*tert*-butyldimethylsilyl)oxy)-2-methyl-2-((trimethylsilyl)oxy)butanenitrile 16

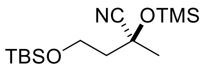

The titled product was purified by column chromatography to afford **16** as colorless oil (57.3 mg, 95% yield).

^{1}H NMR (501 MHz, CD_2Cl_2) δ 3.82 (td, *J* = 6.7, 2.6 Hz, 2H), 1.97 (t, *J* = 6.6 Hz, 2H), 1.61 (s, 3H), 0.90 (s, 9H), 0.23 (s, 9H), 0.07 (s, 6H).

^{13}C NMR (126 MHz, CD_2Cl_2) δ 122.4, 68.4, 59.0, 46.0, 29.8, 26.0, 18.5, 1.4, –5.3, –5.4.

R_f = 0.44 (Ethyl acetate/hexanes = 1:19).

CI-HRMS (*m/z*): calculated for $C_{14}H_{32}N_1O_2Si_2$ ($[M+H]^+$): 302.1966, found: 302.1968.

GC (25.0 m Hydrodex-gamma TBDAc-CD, injection temperature: 220 °C, 95 °C iso 60 min, 8 °C/min, 230 °C iso 3 min, 0.5 bar H_2): t_{R1} = 52.2 min (major), t_{R2} = 50.2 min (minor), e.r. = 87:13.

$[\alpha]_D^{25}$ = –3.8 (*c* 1.64, $CHCl_3$).

3,3-dimethyl-1-((trimethylsilyl)oxy)cyclohexane-1-carbonitrile 17

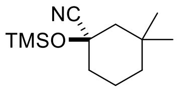

The titled product was purified by column chromatography to afford **17** as colorless oil (40.6 mg, 90% yield).

^{1}H NMR (501 MHz, CD_2Cl_2) δ 1.97–1.93 (m, 1H), 1.78 (dt, *J* = 13.6, 1.3 Hz, 1H), 1.69 (s, 2H), 1.59 (d, *J* = 13.6 Hz, 2H), 1.28 (ddd, *J* = 17.8, 7.9, 3.9 Hz, 2H), 1.02 (s, 3H), 0.99 (s, 3H), 0.23 (s, 9H).

^{13}C NMR (126 MHz, CD_2Cl_2) δ 123.5, 69.4, 50.7, 39.7, 38.3, 31.6, 30.8, 29.3, 19.0, 1.4.

R_f = 0.85 (Diethyl Ether/*i*-hexane = 1:15).

ESI-HRMS (*m/z*): calculated for $C_{12}H_{23}N_1O_1Si_1Na_1$ $[M+Na^+]$: 248.1443, found: 248.1441.

GC (30.0 m BGB 176, injection temperature: 220 °C, 70 °C iso 120 min, 25 °C/min, 220 °C iso 2 min, 0.5 bar H_2): t_{R1} = 111.7 min (major), t_{R2} = 115.6 min (minor), e.r. = 96:4.

$[\alpha]_D^{25}$ = 3.4 (*c* 1.11, $CHCl_3$).

3-oxocyclohexane-1-carbonitrile 18

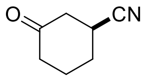

The titled product was purified by column chromatography with 5% ethyl acetate in hexanes as eluent to afford **18** as colorless oil (11.1 mg, 45% yield).

^{1}H NMR (501 MHz, CD_2Cl_2) δ 3.06 (tdd, *J* = 8.9, 5.3, 3.8 Hz, 1H), 2.68–2.61 (m, 1H), 2.56 (ddd, *J* = 14.6, 9.0, 0.7 Hz, 1H), 2.40–2.35 (m, 2H), 2.18–2.07 (m, 2H), 2.05–1.95 (m, 2H).

^{13}C NMR (126 MHz, CD_2Cl_2) δ 205.8, 120.8, 43.7, 41.1, 29.2, 28.5, 24.2.

R_f = 0.30 (Ethyl acetate/hexanes = 1:1).

EI-HRMS (*m/z*): calculated for $C_7H_9N_1O_1$ $[M^{+\bullet}]$: 123.0679, found: 123.0681.

GC (30.0 m BGB 176, injection temperature: 220 °C, 110 °C iso 65 min, 8 °C/min, 240 °C iso 3 min, 0.5 bar H_2): t_{R1} = 56.0 min (major), t_{R2} = 53.3 min (minor), e.r. = 91:9.

Exclusively 1,2-addition was observed with unsaturated conjugated acyclic ketones and TMSCN under standard reaction conditions:

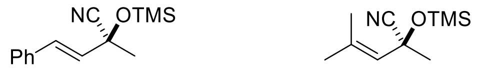

with **4**, >95% conv. 75:25 e.r.

with **2**, >95% conv. 51:49 e.r.

Figure S6. Products derived from conjugated ketones catalyzed by IDPis **2** and **4** under standard conditions.

2-methyl-3-phenyl-2-((trimethylsilyl)oxy)propanenitrile 19

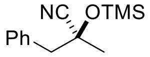

The titled product was purified by column chromatography to afford **19** as colorless oil (42.9 mg, 92% yield).

^{1}H NMR (501 MHz, CD_2Cl_2) δ 7.34–7.29 (m, 5H), 3.04–2.96 (m, 2H), 1.58 (s, 3H), 0.15 (s, 9H).

^{13}C NMR (126 MHz, CD_2Cl_2) δ 135.4, 131.2, 128.5, 127.7, 122.1, 70.4, 49.5, 29.1, 1.1.

R_f = 0.43 (Ethyl acetate/hexanes = 1:19).

ESI-HRMS (*m/z*): calculated for $C_{13}H_{19}N_1O_1Si_1Na_1$ [M+Na$^+$]: 256.1128, found: 256.1126.

HPLC (OJ-3R, MeOH : Water = 75:25, 1.0 mL/min, 298 K, 220 nm): t_{R1} = 10.6 min (major), t_{R2} = 11.7 min (minor), e.r. = 95:5.

$[\boldsymbol{\alpha}]_{\mathbf{D}}^{\mathbf{25}}$ = 3.4 (*c* 1.0, $CHCl_3$).

2-methyl-3-(p-tolyl)-2-((trimethylsilyl)oxy)propanenitrile 20

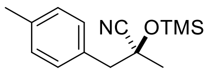

The titled product was purified by column chromatography to afford **20** as colorless oil (45.5 mg, 92% yield).

^{1}H NMR (501 MHz, $CDCl_3$) δ 7.18 (d, *J* = 8.2 Hz, 2H), 7.13 (d, *J* = 7.6 Hz, 2H), 2.99 (d, *J* = 13.5 Hz, 1H), 2.93 (d, *J* = 13.5 Hz, 1H), 2.34 (s, 3H), 1.55 (s, 3H), 0.16 (s, 9H).

^{13}C NMR (126 MHz, CD_2Cl_2) δ 137.1, 131.7, 130.7, 129.0, 121.9, 70.1, 48.9, 28.9, 21.3, 1.2.

R_f = 0.42 (Ethyl acetate/hexanes = 1:19).

EI-HRMS (*m/z*): calculated for $C_{14}H_{21}N_1O_1Si_1$ [$M^{+\bullet}$]: 247.1387, found: 247.1383.

HPLC (OD-3, 100% heptane, 0.5 mL/min, 298 K, 220 nm): t_{R1} = 10.7 min (major), t_{R2} = 12.4 min (minor), e.r. = 93:7.

$[\boldsymbol{\alpha}]_{\mathbf{D}}^{\mathbf{25}}$ = 3.1 (*c* 0.26, $CHCl_3$).

3-(4-methoxyphenyl)-2-methyl-2-((trimethylsilyl)oxy)propanenitrile 21

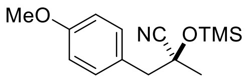

The titled product was purified by column chromatography to afford **21** as colorless oil (49.5 mg, 94% yield).

^{1}H NMR (501 MHz, CD_2Cl_2) δ 7.22 (d, *J* = 8.6 Hz, 2H), 6.87 (d, *J* = 8.7 Hz, 2H), 3.79 (s, 3H), 2.94 (q, *J* = 13.6 Hz, 2H), 1.56 (s, 3H), 0.16 (s, 9H).

^{13}C NMR (126 MHz, CD_2Cl_2) δ 159.5, 132.1, 127.4, 122.2, 113.8, 70.6, 55.6, 48.6, 28.9, 1.2.

R_f = 0.56 (Ethyl acetate/hexanes = 1:9).

EI-HRMS (*m/z*): calculated for $C_{14}H_{21}N_1O_2Si_1$ [$M^{+\bullet}$]: 263.1336, found: 263.1332.

HPLC (OJ-3R, MeOH : Water = 75:25, 1.0 mL/min, 298 K, 220 nm): t_{R1} = 16.3 min (major), t_{R2} = 13.8 min (minor), e.r. = 94:6.

$[\boldsymbol{\alpha}]_{\mathbf{D}}^{\mathbf{25}}$ = 3.4 (*c* 0.64, $CHCl_3$). Lit[8] (91% *ee*)$[\boldsymbol{\alpha}]_{\mathbf{D}}^{\mathbf{25}}$ = –6.7 (*c* 1.05, $CHCl_3$).

2-methyl-3-(naphthalen-2-yl)-2-((trimethylsilyl)oxy)propanenitrile 22

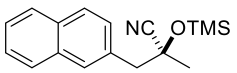

The titled product was purified by column chromatography to afford **22** as colorless oil (51.0 mg, 90% yield).

^{1}H NMR (501 MHz, $CDCl_3$) δ 7.85–7.79 (m, 3H), 7.77–7.74 (m, 1H), 7.51–7.45 (m, 2H), 7.43 (dd, *J* = 8.4, 1.7 Hz, 1H), 3.23–3.12 (m, 2H), 1.62 (s, 3H), 0.15 (s, 9H).

^{13}C NMR (126 MHz, $CDCl_3$) δ 133.4, 132.8, 132.4, 129.7, 128.9, 127.9, 127.8, 127.7, 126.2, 126.0, 121.9, 70.0, 49.4, 29.0, 1.3.

R_f = 0.41 (Ethyl acetate/hexanes = 1:19).

EI-HRMS (*m/z*): calculated for $C_{17}H_{21}N_1O_1Si_1$ [$M^{+\bullet}$]: 283.1387, found: 283.1384.

HPLC (OJ-3, isopropanol : heptane = 0.5:99.5, 0.5 mL/min, 298 K, 254 nm): t_{R1} = 26.7 min (major), t_{R2}= 22.4 min (minor), e.r. = 95:5.

$[\alpha]_D^{25}$ = 3.9 (*c* 0.56, $CHCl_3$).

2-methyl-4-phenyl-2-((trimethylsilyl)oxy)butanenitrile 23

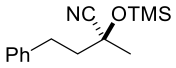

The titled product was purified by column chromatography to afford **23** as colorless oil (47.5 mg, 96% yield).

^{1}H NMR (501 MHz, CD_2Cl_2) δ 7.30 (dd, *J* = 8.1, 7.0 Hz, 2H), 7.21 (ddt, *J* = 14.3, 6.6, 1.5 Hz, 3H), 2.94–2.71 (m, 2H), 2.08–1.98 (m, 2H), 1.64 (s, 3H), 0.27 (s, 9H).

^{13}C NMR (126 MHz, CD_2Cl_2) δ 141.4, 128.9, 128.8, 126.5, 122.3, 69.9, 45.6, 31.1, 29.3, 1.4.

R_f = 0.43 (Ethyl acetate/hexanes = 1:19).

ESI-HRMS (*m/z*): calculated for $C_{14}H_{21}N_1O_1Si_1Na_1$ [$M+Na^+$]: 270.1285, found: 270.1281.

HPLC (IA, 100% heptane, 0.5 mL/min, 298 K, 220 nm): t_{R1} = 18.4 min (major), t_{R2}= 17.3 min (minor), e.r. = 99:1.

$[\alpha]_D^{25}$ = 12.4 (*c* 0.98, $CHCl_3$). Lit[8] (94% *ee*)$[\alpha]_D^{25}$ = –13.3 (*c* 1.1, $CHCl_3$).

2-methyl-4-(p-tolyl)-2-((trimethylsilyl)oxy)butanenitrile 24

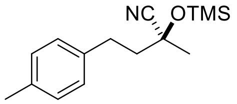

The titled product was purified by column chromatography to afford **24** as colorless oil (49.7 mg, 95% yield).

^{1}H NMR (501 MHz, $CDCl_3$) δ 7.11 (d, *J* = 1.6 Hz, 4H), 2.83 (ddd, *J* = 13.6, 11.2, 5.9 Hz, 1H), 2.75 (ddd, *J* = 13.6, 11.1, 6.1 Hz, 1H), 2.33 (s, 3H), 2.06–1.93 (m, 2H), 1.63 (s, 3H), 0.27 (s, 9H).

^{13}C NMR (126 MHz, $CDCl_3$) δ 137.8, 135.8, 129.4, 128.4, 122.1, 69.5, 45.5, 30.4, 29.2, 21.1, 1.5.

R*f* = 0.28 (Ethyl acetate/hexanes = 1:19).

EI-HRMS (*m/z*): calculated for $C_{15}H_{23}N_1O_1Si_1$ [$M^{+\bullet}$]: 261.1543, found: 261.1541.

HPLC (IA, 100% heptane, 0.5 mL/min, 298 K, 220 nm): t_{R1} = 18.5 min (major), t_{R2} = 17.5 min (minor), e.r. = 98.5:1.5.

$[\alpha]_D^{25}$ = 12.5 (*c* 1.12, $CHCl_3$).

4-(4-methoxyphenyl)-2-methyl-2-((trimethylsilyl)oxy)butanenitrile 25

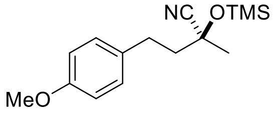

The titled product was purified by column chromatography to afford **25** as colorless oil (52.7 mg, 95% yield).

^{1}H NMR (501 MHz, $CDCl_3$) δ 7.12 (d, *J* = 8.6 Hz, 2H), 6.84 (d, *J* = 8.6 Hz, 2H), 3.79 (s, 3H), 2.81 (ddd, *J* = 13.7, 11.2, 5.9 Hz, 1H), 2.72 (ddd, *J* = 13.7, 11.1, 6.0 Hz, 1H), 1.99 (ddd, *J* = 11.0, 6.5, 5.7 Hz, 2H), 1.62 (s, 3H), 0.27 (s, 9H).

^{13}C NMR (126 MHz, $CDCl_3$) δ 158.2, 132.9, 129.4, 122.1, 114.1, 69.5, 55.4, 45.6, 30.0, 29.2, 1.5.

R_f = 0.31 (Ethyl acetate/hexanes = 1:19).

EI-HRMS (*m/z*): calculated for $C_{15}H_{23}N_1O_2Si_1$ [$M^{+\bullet}$]: 277.1492, found: 277.1491.

HPLC (AS-3, isopropanol : heptane = 0.5:99.5, 0.5 mL/min, 298 K, 254 nm): t_{R1} = 8.0 min (major), t_{R2} = 10.4 min (minor), e.r. = 99:1.

$[\alpha]_D^{25}$ = 9.8 (*c* 0.82, $CHCl_3$).

4-(4-chlorophenyl)-2-methyl-2-((trimethylsilyl)oxy)butanenitrile 26

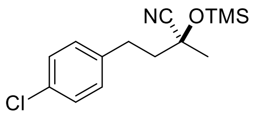

The titled product was purified by column chromatography to afford **26** as colorless oil (50.7 mg, 90% yield).

^{1}H NMR (501 MHz, $CDCl_3$) δ 7.26 (d, *J* = 8.4 Hz, 2H), 7.13 (d, *J* = 8.4 Hz, 2H), 2.85 (ddd, *J* = 13.8, 10.8, 6.3 Hz, 1H), 2.76 (ddd, *J* = 13.7, 10.6, 6.4 Hz, 1H), 2.05–1.92 (m, 2H), 1.63 (s, 3H), 0.26 (s, 9H).

^{13}C NMR (126 MHz, $CDCl_3$) δ 139.3, 132.1, 129.9, 128.8, 121.9, 69.4, 45.3, 30.3, 29.2, 1.5.

R_f = 0.28 (Ethyl acetate/hexanes = 1:19).

ESI-HRMS (*m/z*): calculated for $C_{14}H_{20}N_1O_1Cl_1Si_1Na_1$ [$M+Na^+$]: 304.0895, found: 304.0891.

HPLC (OD-3, 100% heptane, 0.5 mL/min, 298 K, 220 nm): t_{R1} = 15.4 min (major), t_{R2} = 17.1 min (minor), e.r. = 99:1.

$[\alpha]_D^{25}$ = 12.1 (*c* 1.04, $CHCl_3$).

4-(4-bromophenyl)-2-methyl-2-((trimethylsilyl)oxy)butanenitrile 27

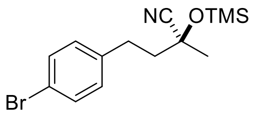

The titled product was purified by column chromatography to afford **27** as colorless oil (59.4 mg, 91% yield).

^{1}H NMR (501 MHz, $CDCl_3$) δ 7.41 (d, *J* = 8.4 Hz, 2H), 7.08 (d, *J* = 8.4 Hz, 2H), 2.83 (ddd, *J* = 13.8, 10.7, 6.3 Hz, 1H), 2.74 (ddd, *J* = 13.7, 10.6, 6.4 Hz, 1H), 2.05–1.93 (m, 2H), 1.62 (s, 3H), 0.26 (s, 9H).

^{13}C NMR (126 MHz, $CDCl_3$) δ 139.8, 131.7, 130.3, 121.9, 120.1, 69.4, 45.2, 30.3, 29.2, 1.4.

R_f = 0.28 (Ethyl acetate/hexanes = 1:19).

ESI-HRMS (*m/z*): calculated for $C_{14}H_{20}N_1O_1Si_1Br_1Na_1$ [$M+Na^+$]: 348.0390, found: 348.0388.

HPLC (OD-3, 100% heptane, 0.5 mL/min, 298 K, 220 nm): t_{R1} = 17.1 min (major), t_{R2} = 19.5 min (minor), e.r. = 99:1.

$[\alpha]_D^{25}$ = 10.2 (*c* 1.0, $CHCl_3$).

2-methyl-4-(naphthalen-1-yl)-2-((trimethylsilyl)oxy)butanenitrile 28

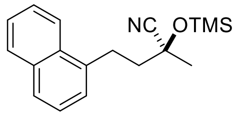

The titled product was purified by column chromatography to afford **28** as colorless oil (55.9 mg, 94% yield).

^{1}H NMR (501 MHz, $CDCl_3$) δ 8.07–8.06 (m, 1H), 7.87 (dd, *J* = 7.8, 1.5 Hz, 1H), 7.80–7.71 (m, 1H), 7.60–7.47 (m, 2H), 7.45–7.33 (m, 2H), 3.42–3.32 (m, 1H), 3.32–3.21 (m, 1H), 2.14 (tt, *J* = 6.7, 1.8 Hz, 2H), 1.69 (d, *J* = 4.4 Hz, 3H), 0.33 (s, 9H).

^{13}C NMR (126 MHz, $CDCl_3$) δ 137.0, 134.1, 131.8, 129.0, 127.2, 126.2, 126.2, 125.7, 125.7, 123.6, 122.1, 69.6, 44.6, 29.2, 28.1, 1.5.

R_f = 0.39 (Ethyl acetate/hexanes = 1:19).

ESI-HRMS (*m/z*): calculated for $C_{18}H_{23}N_1O_1Si_1Na_1$ [M+Na$^+$]: 320.1441, found: 320.1443.

HPLC (OJ-3, isopropanol : heptane = 0.5:99.5, 0.5 mL/min, 298 K, 220 nm): t_{R1} = 10.2 min (major), t_{R2} = 18.0 min (minor), e.r. = 91:9.

$[\alpha]_D^{25}$ = 6.0 (*c* 0.96, $CHCl_3$). Lit[8] (91% *ee*) $[\alpha]_D^{25}$ = –8.9 (*c* 1.09, $CHCl_3$).

2-methyl-4-(naphthalen-2-yl)-2-((trimethylsilyl)oxy)butanenitrile 29

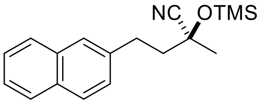

The titled product was purified by column chromatography to afford **29** as colorless oil (56.5 mg, 95% yield).

^{1}H NMR (501 MHz, $CDCl_3$) δ 7.82–7.76 (m, 3H), 7.67–7.64 (m, 1H), 7.50–7.41 (m, 2H), 7.35 (dd, *J* = 8.4, 1.8 Hz, 1H), 3.05 (ddd, *J* = 13.7, 11.1, 6.0 Hz, 1H), 2.96 (ddd, *J* = 13.7, 10.9, 6.1 Hz, 1H), 2.23–2.04 (m, 2H), 1.67 (s, 3H), 0.29 (s, 9H).

^{13}C NMR (126 MHz, $CDCl_3$) δ 138.4, 133.8, 132.2, 128.3, 127.8, 127.6, 127.2, 126.6, 126.2, 125.5, 122.1, 69.6, 45.3, 31.1, 29.2, 1.5.

R_f = 0.39 (Ethyl acetate/hexanes = 1:19).

ESI-HRMS (*m/z*): calculated for $C_{18}H_{23}N_1O_1Si_1Na_1$ [M+Na$^+$]: 320.1441, found: 320.1443.

HPLC (IB-3, isopropanol : heptane = 0.5:99.5, 0.5 mL/min, 298 K, 220 nm): t_{R1} = 7.5 min (major), t_{R2} = 8.0 min (minor), e.r. = 98:2.

$[\alpha]_D^{25}$ = 12.4 (*c* 0.68, $CHCl_3$). Lit[8] (92% *ee*) $[\alpha]_D^{25}$ = –10.8 (*c* 1.15, $CHCl_3$).

(*S*)-2-phenyl-2-((trimethylsilyl)oxy)propanenitrile 30

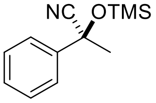

The titled product was purified by column chromatography to afford **30** as colorless oil (41.4 mg, 94% yield).

^{1}H NMR (501 MHz, CD_2Cl_2) δ 7.56–7.54 (m, 2H), 7.44–7.39 (m, 2H), 7.38–7.34 (m, 1H), 1.86 (s, 3H), 0.18 (s, 9H).

^{13}C NMR (126 MHz, CD_2Cl_2) δ 142.5, 129.0, 129.0, 125.1, 122.1, 72.0, 33.6, 1.1.

R_f = 0.32 (Ethyl acetate/hexanes = 1:19).

ESI-HRMS (*m/z*): calculated for $C_{12}H_{17}N_1O_1Si_1Na_1$ [M+Na$^+$]: 242.0972, found: 242.0971.

HPLC (OD-3, 100% heptane, 0.5 mL/min, 298 K, 220 nm): t_{R1} = 8.4 min (major), t_{R2} = 7.8 min (minor), e.r. = 96:4.

$[\alpha]_D^{25}$ = –18.8 (*c* 1.03, $CHCl_3$).

The absolute configuration was determined to be *S* by comparison with the previously reported optical rotation [lit[10] (93% *ee*) $[\alpha]_D^{24}$ = 21.9 (*c* 1.18, $CHCl_3$, *R* enantiomer)].

2-(p-tolyl)-2-((trimethylsilyl)oxy)propanenitrile 31

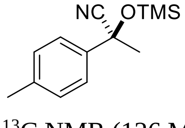

The titled product was purified by column chromatography to afford **31** as colorless oil (44.3 mg, 95% yield).

^{1}H NMR (501 MHz, CD_2Cl_2) δ 7.43 (d, *J* = 8.3 Hz, 2H), 7.24–7.19 (m, 2H), 2.36 (s, 3H), 1.84 (s, 3H), 0.16 (s, 9H).

^{13}C NMR (126 MHz, CD_2Cl_2) δ 139.6, 139.1, 129.6, 125.0, 122.2, 71.9, 33.5, 21.2, 1.1.

R_f = 0.32 (Ethyl acetate/hexanes = 1:19).

ESI-HRMS (*m/z*): calculated for $C_{13}H_{19}N_1O_1Na_1Si_1$ [M+Na$^+$]: 256.1128, found: 256.1127.

HPLC (OJ-3R, MeOH : Water = 75:25, 1.0 mL/min, 298 K, 220 nm): t_{R1} = 18.2 min (major), t_{R2} = 11.5 min (minor), e.r. = 95.5:4.5.

$[\alpha]_D^{25}$ = –20.6 (*c* 0.93, $CHCl_3$). Lit[11] (86% *ee*) $[\alpha]_D^{24}$ = –17.6 (c 1.0, $CHCl_3$).

2-(4-fluorophenyl)-2-((trimethylsilyl)oxy)propanenitrile 32

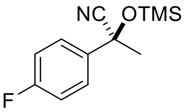

The titled product was purified by column chromatography to afford **32** as colorless oil (45.0 mg, 95% yield).

^{1}H NMR (501 MHz, $CDCl_3$) δ 7.54–7.51 (m, 2H), 7.10–7.06 (m, 2H), 1.84 (s, 3H), 0.18 (s, 9H).

^{13}C NMR (126 MHz, $CDCl_3$) δ 162.8 (d, *J* = 247.8 Hz), 138.1 (d, *J* = 3.3 Hz), 126.6 (d, *J* = 8.5 Hz), 121.6, 115.7 (d, *J* = 21.6 Hz), 71.2, 33.7, 1.2.

^{19}F NMR (471 MHz, $CDCl_3$) δ –113.3.

R_f = 0.32 (Ethyl acetate/hexanes = 1:19).

ESI-HRMS (*m/z*): calculated for $C_{12}H_{16}F_1N_1O_1Si_1Na_1$ [M+Na$^+$]: 260.0877, found: 260.0876.

HPLC (OJ-3R, MeOH : Water = 75:25, 1.0 mL/min, 298 K, 220 nm): t_{R1} = 11.4 min (major), t_{R2} = 8.1 min (minor), e.r. = 93:7.

$[\alpha]_D^{25}$ = –12.8 (*c* 0.14, $CHCl_3$). Lit[7] (92% *ee*) $[\alpha]_D^{22}$ = 17.6 (*c* 2.7, CH_2Cl_2).

2-(4-chlorophenyl)-2-((trimethylsilyl)oxy)propanenitrile 33

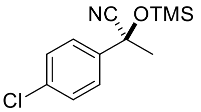

The titled product was purified by column chromatography to afford **33** as colorless oil (45.7 mg, 90% yield).

^{1}H NMR (501 MHz, $CDCl_3$) δ 7.49–7.47 (m, 2H), 7.40–7.34 (m, 2H), 1.83 (s, 3H), 0.19 (s, 9H).

^{13}C NMR (126 MHz, $CDCl_3$) δ 140.9, 134.8, 129.0, 126.2, 121.4, 71.2, 33.7, 1.2.

R_f = 0.32 (Ethyl acetate/hexanes = 1:19).

ESI-HRMS (*m/z*): calculated for $C_{12}H_{16}Cl_1N_1O_1Si_1Na_1$ [M+Na$^+$]: 276.0582, found: 276.0580.

HPLC (OJ-3R, MeOH : Water = 75:25, 1.0 mL/min, 298 K, 220 nm): t_{R1} = 16.3 min (major), t_{R2} = 12.5 min (minor), e.r. = 92.5:7.5.

$[\alpha]_D^{25}$ = –13.0 (*c* 0.36, $CHCl_3$). Lit[8] (92% *ee*) $[\alpha]_D^{25}$ = –20.3 (*c* 1.08, $CHCl_3$).

2-(4-bromophenyl)-2-((trimethylsilyl)oxy)propanenitrile 34

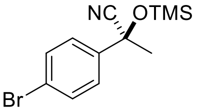

The titled product was purified by column chromatography to afford **34** as colorless oil (47.5 mg, 80% yield).

^{1}H NMR (501 MHz, $CDCl_3$) δ 7.54–7.52 (m, 2H), 7.44–7.40 (m, 2H), 1.83 (s, 3H), 0.19 (s, 9H).

^{13}C NMR (126 MHz, $CDCl_3$) δ 141.4, 132.0, 126.5, 122.9, 121.3, 71.3, 33.7, 1.2.

R_f = 0.52 (Ethyl acetate/hexanes = 1:19).

ESI-HRMS (*m/z*): calculated for $C_{12}H_{16}N_1O_1Br_1Si_1Na_1$ [M+Na$^+$]: 320.0077, found: 320.0076.

HPLC (OJ-3R, MeOH : Water = 75:25, 1.0 mL/min, 298 K, 220 nm): t_{R1} = 20.5 min (major), t_{R2} = 15.6 min (minor), e.r. = 92:8.

$[\alpha]_D^{25}$ = –14.8 (*c* 0.35, $CHCl_3$). Lit[12] (93% *ee*) $[\alpha]_D^{25}$ = –20.1 (*c* 1.0, $CHCl_3$).

2-(m-tolyl)-2-((trimethylsilyl)oxy)propanenitrile 35

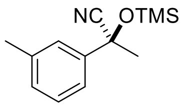

The titled product was purified by column chromatography to afford **35** as colorless oil (44.8 mg, 96% yield).

^{1}H NMR (501 MHz, $CDCl_3$) δ 7.35–7.32 (m, 2H), 7.30–7.26 (m, 1H), 7.18–7.14 (m, 1H), 2.41–2.36 (m, 3H), 1.85 (s, 3H), 0.17 (s, 9H).

^{13}C NMR (126 MHz, $CDCl_3$) δ 142.1, 138.5, 129.5, 128.7, 125.4, 121.9, 121.8, 71.8, 33.7, 21.7, 1.2.

R_f = 0.34 (Ethyl acetate/hexanes = 1:19).

ESI-HRMS (*m/z*): calculated for $C_{13}H_{19}N_1O_1Si_1Na_1$ [M+Na$^+$]: 256.1128, found: 256.1128.

HPLC (OJ-3R, MeOH : Water = 75:25, 1.0 mL/min, 298 K, 220 nm): t_{R1} = 10.1 min (major), t_{R2} = 9.0 min (minor), e.r. = 97.5:2.5.

$[\alpha]_D^{25}$ = –20.8 (*c* 0.94, $CHCl_3$).

2-(3-methoxyphenyl)-2-((trimethylsilyl)oxy)propanenitrile 36

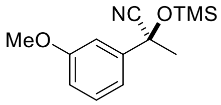

The titled product was purified by column chromatography to afford **36** as colorless oil (45.9 mg, 92% yield).

^{1}H NMR (501 MHz, $CDCl_3$) δ 7.31 (t, *J* = 8.0 Hz, 1H), 7.13 (ddd, *J* = 7.8, 1.8, 0.9 Hz, 1H), 7.08 (t, *J* = 2.2 Hz, 1H), 6.88 (ddd, *J* = 8.2, 2.6, 0.9 Hz, 1H), 3.83 (s, 3H), 1.85 (s, 3H), 0.19 (s, 9H).

^{13}C NMR (126 MHz, $CDCl_3$) δ 159.9, 143.8, 129.9, 121.7, 117.1, 114.0, 110.7, 71.7, 55.5, 33.7, 1.2.

R_f = 0.32 (Ethyl acetate/hexanes = 1:19).

EI-HRMS (*m/z*): calculated for $C_{13}H_{19}N_1O_2Si_1$ [$M^{+\bullet}$]: 249.1180, found: 249.1177.

HPLC (OD-3, 100% heptane, 0.5 mL/min, 298 K, 220 nm): t_{R1} = 15.5 min (major), t_{R2} = 12.1 min (minor), e.r. = 97:3.

$[\alpha]_D^{25}$ = –21.7 (*c* 0.94, $CHCl_3$). Lit[12] (97% *ee*) $[\alpha]_D^{25}$ = –21.8 (*c* 1.0, $CHCl_3$).

2-(3-chlorophenyl)-2-((trimethylsilyl)oxy)propanenitrile 37

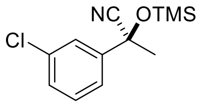

The titled product was purified by column chromatography to afford **37** as colorless oil (37.5 mg, 74% yield).

^{1}H NMR (501 MHz, CD_2Cl_2) δ 7.55 (dt, *J* = 2.5, 1.0 Hz, 1H), 7.46 (ddd, *J* = 6.4, 2.8, 1.9 Hz, 1H), 7.37–7.35 (m, 2H), 1.84 (s, 3H), 0.21 (s, 9H).

^{13}C NMR (126 MHz, CD_2Cl_2) δ 144.7, 134.9, 130.5, 129.2, 125.4, 123.4, 121.6, 71.5, 33.6, 1.1.

R_f = 0.34 (Ethyl acetate/hexanes = 1:19).

ESI-HRMS (*m/z*): calculated for $C_{12}H_{16}N_1O_1Na_1Cl_1Si_1$ [M+Na$^+$]: 276.0582, found: 276.0582.

HPLC (OD-3, 100% heptane, 0.5 mL/min, 298 K, 220 nm): t_{R1} = 8.0 min (major), t_{R2} = 7.2 min (minor), e.r. = 92.5:7.5.

$[\alpha]_D^{25}$ = –18.6 (*c* 0.28, $CHCl_3$). Lit[8] (89% *ee*) $[\alpha]_D^{25}$ = –20.4 (*c* 1.27, $CHCl_3$).

2-(3-(trifluoromethyl)phenyl)-2-((trimethylsilyl)oxy)propanenitrile 38

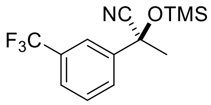

The titled product was purified by column chromatography to afford **38** as colorless oil (37.3 mg, 65% yield).

^{1}H NMR (501 MHz, $CDCl_3$) δ 7.79 (d, *J* = 1.8 Hz, 1H), 7.75 (dt, *J* = 7.8, 1.4 Hz, 1H), 7.65–7.61 (m, 1H), 7.54 (t, *J* = 7.8 Hz, 1H), 1.87 (s, 3H), 0.21 (s, 9H).

^{13}C NMR (126 MHz, $CDCl_3$) δ 143.5, 131.4 (q, *J* = 32.8 Hz), 129.4, 128.2, 125.7 (q, *J* = 3.7 Hz), 123.9 (q, *J* = 273.2 Hz), 121.7 (q, *J* = 4.0 Hz), 121.2, 71.2, 33.7, 1.2.

^{19}F NMR (471 MHz, $CDCl_3$) δ –62.8.

R_f = 0.51 (Ethyl acetate/hexanes = 1:19).

ESI-HRMS (*m/z*): calculated for $C_{13}H_{16}N_1O_1F_3Si_1Na_1$ [M+Na$^+$]: 310.0845, found: 310.0847.

HPLC (OD-3, 100% heptane, 0.5 mL/min, 298 K, 220 nm): t_{R1} = 7.2 min (major), t_{R2} = 6.6 min (minor), e.r. = 92:8 (e.r. = 95:5 at –110 °C).

$[\alpha]_D^{25}$ = –22.5 (*c* 0.16, $CHCl_3$, 90% *ee*).

2-(2-chlorophenyl)-2-((trimethylsilyl)oxy)propanenitrile 39

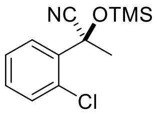

The titled product was purified by column chromatography to afford **39** as colorless oil (45.7 mg, 90% yield).

^{1}H NMR (501 MHz, $CDCl_3$) δ 7.72–7.69 (m, 1H), 7.43–7.40 (m, 1H), 7.35–7.28 (m, 2H), 2.00 (s, 3H), 0.29 (s, 9H).

^{13}C NMR (126 MHz, $CDCl_3$) δ 138.2, 131.7, 131.4, 130.1, 127.2, 127.1, 120.6, 70.4, 30.0, 1.3.

R_f = 0.45 (Ethyl acetate/hexanes = 1:19).

ESI-HRMS (*m/z*): calculated for $C_{12}H_{16}N_1O_1Cl_1Si_1Na_1$ [M+Na$^+$]: 276.0582, found: 276.0582.

HPLC (OD-3, 100% heptane, 0.5 mL/min, 298 K, 220 nm): t_{R1} = 10.4 min (major), t_{R2} = 9.0 min (minor), e.r. = 78:22.

2-(3,5-dimethylphenyl)-2-((trimethylsilyl)oxy)propanenitrile 40

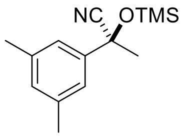

The titled product was purified by column chromatography to afford **40** as colorless oil (47.5 mg, 96% yield).

^{1}H NMR (501 MHz, $CDCl_3$) δ 7.14 (dd, *J* = 1.7, 0.9 Hz, 2H), 6.97 (dt, *J* = 1.6, 0.8 Hz, 1H), 2.34 (d, *J* = 0.7 Hz, 6H), 1.83 (s, 3H), 0.17 (s, 9H).

^{13}C NMR (126 MHz, $CDCl_3$) δ 142.0, 138.4, 130.4, 122.5, 122.0, 71.8, 33.7, 21.5, 1.3.

R_f = 0.58 (Ethyl acetate/hexanes = 1:19).

ESI-HRMS (*m/z*): calculated for $C_{14}H_{21}N_1O_1Si_1Na_1$ [M+Na$^+$]: 270.1285, found: 270.1282.

GC (30.0 m G-TA, injection temperature: 220 °C, 80 °C iso 110 min, 8 °C/min, 180 °C iso 3 min, 0.5 bar H_2): t_{R1} = 96.5 min (major), t_{R2} = 91.6 min (minor), e.r. = 98:2.

$[\alpha]_D^{25}$ = –16.0 (*c* 1.05, $CHCl_3$).

2-(naphthalen-2-yl)-2-((trimethylsilyl)oxy)propanenitrile 41

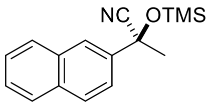

The titled product was purified by column chromatography to afford **41** as colorless oil (49.0 mg, 91% yield).

^{1}H NMR (501 MHz, $CDCl_3$) δ 8.04 (d, *J* = 2.0 Hz, 1H), 7.88 (ddd, *J* = 15.9, 7.7, 3.0 Hz, 3H), 7.66–7.59 (m, 1H), 7.58–7.50 (m, 2H), 1.94 (d, *J* = 1.2 Hz, 3H), 0.20 (s, 9H).

^{13}C NMR (126 MHz, $CDCl_3$) δ 139.4, 133.4, 133.0, 128.9, 128.5, 127.8, 126.9, 126.8, 123.9, 122.5, 121.8, 72.0, 33.7, 1.3.

R_f = 0.39 (Ethyl acetate/hexanes = 1:19).

EI-HRMS (*m/z*): calculated for $C_{16}H_{19}N_1O_1Si_1$ [$M^{+\bullet}$]: 269.1230, found: 269.1229.

HPLC (OD-3, 100% heptane, 0.5 mL/min, 298 K, 254 nm): t_{R1} = 15.5 min (major), t_{R2} = 13.8 min (minor), e.r. = 96:4.

$[\alpha]_D^{25}$ = –11.6 (*c* 1.47, $CHCl_3$). Lit[8] (90% *ee*) $[\alpha]_D^{25}$ = –11.3 (*c* 1.15, $CHCl_3$).

2-methyl-3-(thiophen-2-yl)-2-((trimethylsilyl)oxy)propanenitrile 42

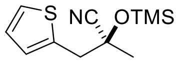

The titled product was purified by column chromatography to afford **42** as colorless oil (31.1 mg, 65% yield).

^{1}H NMR (501 MHz, CD_2Cl_2) δ 7.25 (dd, *J* = 4.6, 1.8 Hz, 1H), 7.02–6.96 (m, 2H), 3.29–3.18 (m, 2H), 1.60 (s, 3H), 0.21 (s, 9H).

^{13}C NMR (126 MHz, CD_2Cl_2) δ 136.5, 128.3, 126.9, 125.8, 121.9, 70.0, 43.9, 28.6, 1.2.

R$_f$ = 0.35 (Ethyl acetate/hexanes = 1:19).

EI-HRMS (*m/z*): calculated for $C_{11}H_{17}N_1O_1S_1Si_1$ [$M^{+\bullet}$]: 239.0795, found: 239.0791.

GC (30.0 m G-TA, injection temperature: 220 °C, 90 °C iso 60 min, 8 °C/min, 180 °C iso 3 min, 0.5 bar H_2): t_{R1} = 51.3 min (major), t_{R2} = 53.8 min (minor), e.r. = 98:2.

$[\alpha]_D^{25}$ = 18.0 (*c* 1.13, $CHCl_3$).

3-(furan-2-yl)-2-methyl-2-((trimethylsilyl)oxy)propanenitrile 43

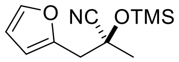

The titled product was purified by column chromatography to afford **43** as colorless oil (38.4 mg, 86% yield).

^{1}H NMR (501 MHz, CD_2Cl_2) δ 7.40 (dd, *J* = 1.9, 0.8 Hz, 1H), 6.37 (dd, *J* = 3.2, 1.9 Hz, 1H), 6.28 (dd, *J* = 3.3, 0.8 Hz, 1H), 3.15 – 3.01 (m, 2H), 1.58 (s, 3H), 0.20 (s, 9H).

^{13}C NMR (126 MHz, CD_2Cl_2) δ 149.8, 142.6, 121.9, 110.9, 109.5, 69.6, 42.2, 28.9, 1.2.

R$_f$ = 0.41 (Ethyl acetate/hexanes = 1:19).

EI-HRMS (*m/z*): calculated for $C_{11}H_{17}N_1O_2Si_1$ [$M^{+\bullet}$]: 223.1023, found: 223.1019.

GC (30.0 m G-TA, injection temperature: 220 °C, 70 °C iso 62 min, 8 °C/min, 180 °C iso 3 min, 0.5 bar H_2): t_{R1} = 50.9 min (major), t_{R2} = 57.5 min (minor), e.r. = 97:3.

$[\alpha]_D^{25}$ = 4.5 (*c* 1.5, $CHCl_3$).

2-methyl-4-(thiophen-2-yl)-2-((trimethylsilyl)oxy)butanenitrile 44

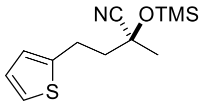

The titled product was purified by column chromatography to afford **44** as colorless oil (45.8 mg, 90% yield).

^{1}H NMR (501 MHz, CD_2Cl_2) δ 7.15 (dd, *J* = 5.2, 1.2 Hz, 1H), 6.93 (dd, *J* = 5.2, 3.4 Hz, 1H), 6.86–6.83 (m, 1H), 3.16–2.96 (m, 2H), 2.14–2.07 (m, 2H), 1.63 (s, 3H), 0.26 (s, 9H).

^{13}C NMR (126 MHz, CD_2Cl_2) δ 144.0, 127.2, 124.9, 123.7, 122.1, 69.6, 45.6, 29.2, 25.2, 1.4.

R$_f$ = 0.45 (Ethyl acetate/hexanes = 1:19).

CI-HRMS (*m/z*): calculated for $C_{12}H_{20}N_1O_1S_1Si_1$ ($[M+H]^+$): 254.1029, found: 254.1027.

HPLC (IA, 100% heptane, 0.5 mL/min, 298 K, 220 nm): t_{R1} = 20.0 min (major), t_{R2} = 18.4 min (minor), e.r. = 99:1.

$[\alpha]_D^{25}$ = 13.3 (*c* 1.28, $CHCl_3$).

(3*S*,5*S*,8*R*,9*S*,10*S*,13*S*,14*S*,17*S*)-10,13-dimethyl-3,17-bis((trimethylsilyl)oxy)hexadecahydro-1*H*-cyclopenta[*a*]phenanthrene-3-carbonitrile 45

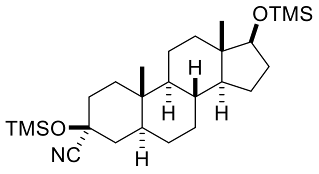

The titled product was purified by column chromatography to afford **45** as white solid (85.0 mg, 92% yield).

^{1}H NMR (501 MHz, CD_2Cl_2) δ 3.58–3.52 (m, 1H), 2.05–1.97 (m, 1H), 1.88 (dtd, *J* = 13.3, 9.3, 5.9 Hz, 1H), 1.79–1.66 (m, 5H), 1.56–1.51 (m, 2H), 1.46–1.35 (m, 3H), 1.34–1.16 (m, 6H), 1.03–0.85 (m, 3H), 0.82 (s, 3H), 0.77–0.70 (m, 1H), 0.68 (s, 3H), 0.23 (s, 9H), 0.07 (s, 9H).

^{13}C NMR (126 MHz, CD_2Cl_2) δ 122.6, 82.1, 72.7, 54.5, 51.0, 44.1, 43.5, 42.0, 37.4, 36.2, 36.1, 35.9, 35.8, 31.8, 31.2, 28.4, 23.8, 21.2, 12.4, 11.6, 1.6, 0.3.

R_f = 0.44 (Ethyl acetate/hexanes = 1:19).

ESI-HRMS (*m/z*): calculated for $C_{26}H_{47}N_1O_2Si_2Na_1$ [M+Na$^+$]: 484.3038, found: 484.3037.

$[\alpha]_D^{25}$ = 8.4 (*c* 1.0, $CHCl_3$).

m.p. = 140–144 ºC.

5. Limitations of the method:

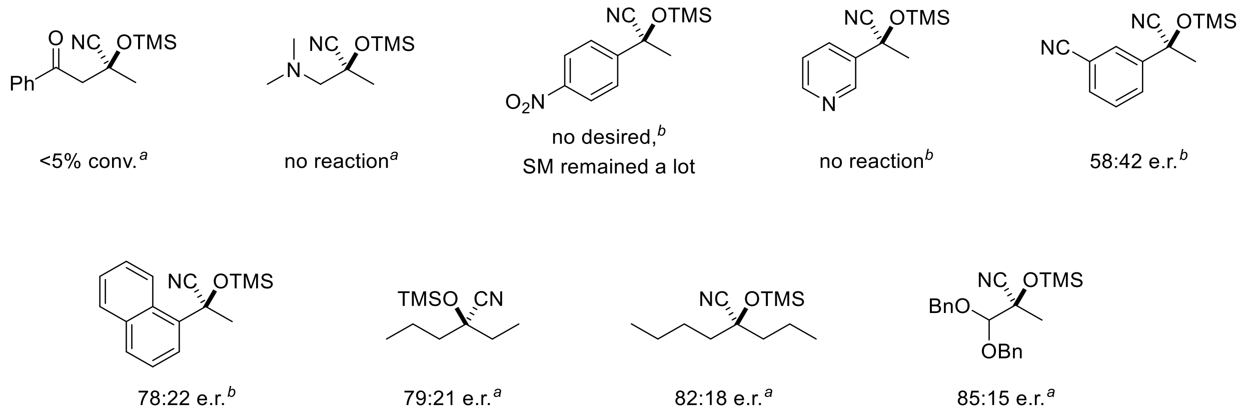

Figure S7. [a]Catalyzed by IDPi **2** under standard conditions. [b]Catalyzed by IDPi **5** under standard conditions.

6. Gram-scale reaction and derivatizations of the cyanohydrin silyl ether 23

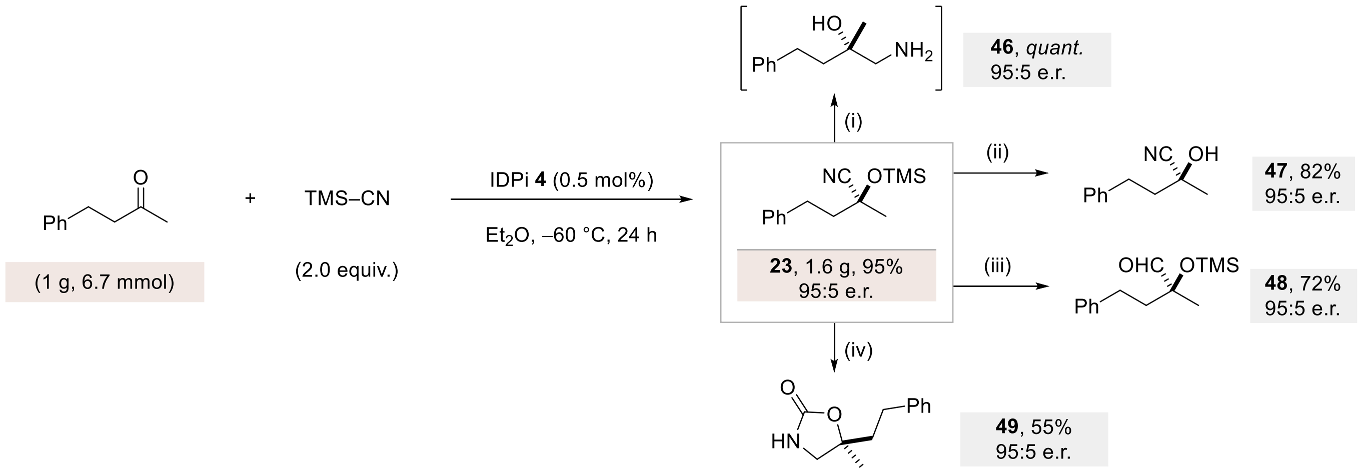

Procedure for the gram scale catalytic cyanosilylation of 4-phenylbutan-2-one with TMSCN 1.

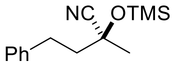

TMSCN **1** (1.7 mL, 13.4 mmol, 2.0 equiv.) was placed in a 25 mL flame-dried Schlenk flask equipped with a Teflon-coated magnetic stirring bar. A solution of IDPi **4** (52.6 mg, 0.034 mmol, 0.005 equiv.) in diethylether (0.4 M, 16.8 mL) was added at rt. After stirring for 3.0 h at rt, the reaction mixture was cooled to –60 °C for 0.5 h, and 4-phenylbutan-2-one **52** (1.0 g, 6.7 mmol, 1.0 equiv.) was slowly added. The resultant mixture was stirred at –60 °C for 24 h until the ketone was fully consumed (monitored by TLC). Upon completion, the reaction mixture was treated with three drops of triethylamine. Volatiles were removed in *vacuo* and the residue was purified by column chromatography with neutral aluminium oxide (activated by 20 wt % water and washed by 50 mL triethylamine followed by 200 mL pentane) padded in 8 cm height and 3 cm width, using 500 mL 5% diethylether in pentane as eluent to afford the product **23** in 95% isolated yield (fraction of test tubes (16 cm height and 1.5 cm width) 1–16 was collected). The enantiomeric ratio (e.r.) was determined by HPLC analysis. (1.6 g, 95:5 e.r.).

i. Preparation of 1-amino-2-methyl-4-phenylbutan-2-ol 46

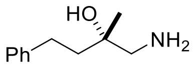

The titled compound was synthesized as an intermediate to the corresponding 4-bromobenzenesulfonate according to a known method with minor modification.[8]

To a flame-dried Schlenk tube were sequentially added $LiAlH_4$ (1.0 M in diethyl ether, 1.0 mL), anhydrous THF (1.0 mL) and Et_2O (5.0 mL). After the resulting solution was cooled down to 0 °C by an ice bath, a solution of **23** (123.7 mg, 0.5 mmol) in Et_2O (5.0 mL) was added dropwise during a period of 5 min. After the mixture was slowly warmed to rt and stirred for an additional 1 h, full conversion of **23** was observed and wet Et_2O (10 wt% water in Et_2O, 5 mL) was slowly added at 0 °C. $NaSO_4$ was added and the reaction was stirred at rt for 5 min. Then we filtered and removed the solvent under *vacuo*, and the obtained amino alcohol **46** was dissolved in 2 mL anhydrous DCM followed by the addition of triethylamine (0.21 mL, 1.5 mmol) and 4-bromobenzene-1-sulfonyl chloride (255.6 mg, 1 mmol). The reaction was stirred at rt for 24 h, and quenched by NH_4Cl (sat.). After work-up, the derivative of **46** could be obtained as white solid by column chromatography with hexanes/EtOAc as eluent. (118 mg, 59% yield, 95:5 e.r.)

4-bromo-N-(2-hydroxy-2-methyl-4-phenylbutyl)benzenesulfonamide

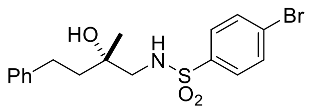

^{1}H NMR (501 MHz, $CDCl_3$) δ 7.73–7.66 (m, 2H), 7.65–7.60 (m, 2H), 7.30–7.22 (m, 2H), 7.21–7.15 (m, 1H), 7.15–7.11 (m, 2H), 5.09 (t, J = 6.5 Hz, 1H), 2.93 (dd, J = 12.6, 6.3 Hz, 1H), 2.87 (dd, J = 12.6, 6.6 Hz, 1H), 2.68–2.58 (m, 2H), 1.84 (s, 1H), 1.81–1.74 (m, 2H), 1.24 (s, 3H).

^{13}C NMR (126 MHz, $CDCl_3$) δ 141.7, 138.9, 132.6, 128.7, 128.7, 128.4, 127.8, 126.2, 72.3, 52.3, 41.6, 30.2, 24.8.

R_f = 0.38 (Ethyl acetate/hexanes = 2:3).

ESI-HRMS (*m/z*): calculated for $C_{17}H_{20}N_1O_3S_1Br_1Na_1$ [M+Na$^+$]: 420.0240, found: 420.0240.

HPLC (IC-3, isopropanol: heptane = 15:85, 1.0 mL/min, 298 K, 254 nm): t_{R1} = 13.1 min (major), t_{R2} = 15.6 min (minor), e.r. = 95:5.

m.p. = 110–112 °C.

$[\alpha]_D^{25}$ = –3.7 (c 1.02, $CHCl_3$).

ii. Preparation of 2-hydroxy-2-methyl-4-phenylbutanenitrile 47

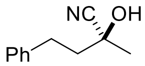

According to a known procedure with modification[13]

To a GC vial equipped with a stirring bar, compound **23** (49.5 mg, 0.2 mmol) and 5 mL TFA solution in DCM (DCM/TFA 4:1, v/v) were added to split the silyl ether, and the reaction mixture was stirred at rt for 6 h. When the starting material was fully converted indicated by TLC (with 5–20% ethyl acetate in hexanes as eluent), the crude mixture was concentrated and purified by column chromatography with silica gel to provide **47** as colorless liquid. (28.7 mg, 82%)

^{1}H NMR (501 MHz, $CDCl_3$) δ 7.32 (dd, J = 8.2, 6.9 Hz, 2H), 7.25–7.21 (m, 3H), 2.98–2.81 (m, 2H), 2.65 (s, 1H), 2.13–2.04 (m, 2H), 1.66 (s, 3H).

^{13}C NMR (126 MHz, $CDCl_3$) δ 140.2, 128.9, 128.5, 126.6, 121.8, 68.8, 43.5, 30.9, 28.2.

R_f = 0.41 (Ethyl acetate/hexanes = 1:4).

ESI-HRMS (*m/z*): calculated for $C_{11}H_{13}N_1O_1Na_1$ [M+Na$^+$]: 198.0889, found: 198.0891.

The e.r. of **47** was determined by converting to the corresponding ester:

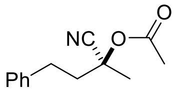

To a flame-dried flask equipped with a stirring bar, compound **47** (44 mg, 0.25 mmol) and 2 mL DCM were added, followed by the addition of acetyl chloride (43 μL, 0.6 mmol) and pyridine (81 μL, 1.0 mmol), and the reaction mixture was stirred at rt for 24 h. When the starting material was fully converted indicated by TLC (with 10% ethyl acetate in hexanes as eluent), the crude mixture was quenched by $NaHCO_3$ solution and extracted by DCM. Then the combined mixture was concentrated and purified by column chromatography with silica gel to provide the ester as yellow liquid. (40.2 mg, 74%)

^{1}H NMR (501 MHz, $CDCl_3$) δ 7.34–7.28 (m, 2H), 7.22 (ddd, J = 8.2, 6.9, 1.7 Hz, 3H), 2.95–2.79 (m, 2H), 2.31 (ddd, J = 14.0, 11.4, 5.5 Hz, 1H), 2.21 (ddd, J = 14.0, 11.6, 5.4 Hz, 1H), 2.08 (s, 3H), 1.80 (s, 3H).

^{13}C NMR (126 MHz, $CDCl_3$) δ 169.0, 140.0, 128.8, 128.5, 126.6, 118.6, 71.7, 41.5, 30.4, 24.7, 21.2.

R_f = 0.19 (Ethyl acetate/hexanes = 1:9).

ESI-HRMS (*m/z*): calculated for $C_{13}H_{15}N_1O_2Na_1$ [M+Na$^+$]: 240.0995, found: 240.0996.

HPLC (IE-3, isopropanol: heptane = 1:99, 1.0 mL/min, 298 K, 220 nm): t_{R1} = 15.4 min (major), t_{R2} = 17.6 min (minor), e.r. = 95:5.

$[\alpha]_D^{25}$ = 37.3 (*c* 1.42, $CHCl_3$).

iii. Preparation of 2-methyl-4-phenyl-2-((trimethylsilyl)oxy)butanal 48

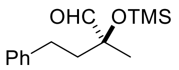

According to known methods with modification[8,14]

To a flame-dried Schlenk tube, **23** (61.8 mg, 0.25 mmol) and anhydrous Et_2O (1.0 mL) were added stepwise. After the resulting mixture was cooled down to –30 °C, diisobutylaluminum hydride (1.0 M in cyclohexane, 0.38 mL) was added dropwise during a period of 5 min. Then the reaction was warmed up to rt and stirred for 12 h. When full consumption of **23** was observed indicated by TLC analysis with Al_2O_3 TLC plate, 2 mL NH_4Cl (sat.) was slowly added to quench the reaction. After work-up, the residue was purified by column chromatography, affording **48** as colorless oil. (45.1 mg, 72%)

^{1}H NMR (501 MHz, CD_2Cl_2) δ 9.55 (s, 1H), 7.27 (dd, *J* = 8.1, 7.0 Hz, 2H), 7.21–7.13 (m, 3H), 2.70 (ddd, *J* = 13.7, 11.8, 4.9 Hz, 1H), 2.56 (ddd, *J* = 13.7, 12.1, 5.3 Hz, 1H), 2.02–1.91 (m, 1H), 1.83 (ddd, *J* = 13.8, 12.0, 4.9 Hz, 1H), 1.34 (s, 3H), 0.20 (s, 9H).

^{13}C NMR (126 MHz, CD_2Cl_2) δ 204.8, 142.4, 128.8, 128.7, 126.3, 81.1, 41.2, 30.1, 23.3, 2.4.

R_f = 0.35 (Ethyl acetate/hexanes = 1:19).

ESI-HRMS (*m/z*): calculated for $C_{14}H_{22}O_2Si_1Na_1$ [M+Na$^+$]: 273.1281, found: 273.1280.

HPLC (OD-3, isopropanol: heptane = 0.5:99.5, 1.0 mL/min, 298 K, 190 nm): t_{R1} = 5.0 min (major), t_{R2} = 4.1 min (minor), e.r. = 95:5.

$[\alpha]_D^{25}$ = 11.5 (*c* 0.78, $CHCl_3$).

iv. Preparation of 5-methyl-5-phenethyloxazolidin-2-one 49

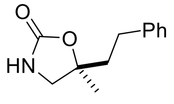

According to a known procedure with modification[8]

Step 1: To a flame-dried Schlenk tube were sequentially added $LiAlH_4$ (1.0 M in diethyl ether, 1.0 mL), anhydrous THF (1.0 mL) and Et_2O (5.0 mL). After the resulting solution was cooled down to 0 °C by an ice bath, a solution of **23** (123.7 mg, 0.5 mmol) in Et_2O (5.0 mL) was added dropwise during a period of 5 min. After the mixture was slowly warmed to rt and stirred for an additional 1 h, full conversion of **23** was observed and wet Et_2O (10 wt% water in Et_2O, 5 mL) was slowly added at 0 °C. $NaSO_4$ was added and the reaction was stirred at rt for 5 min. Then we filtered and removed the solvent under *vacuo*, and the crude amino alcohol **46** could be obtained and directly used in the next step without further purification.

Step 2: The product obtained from the above-mentioned procedure was dissolved in anhydrous THF (4.0 mL), and followed by the addition of 1,1'-carbonyldiimidazole (81.0 mg, 0.5 mmol). The resultant mixture was vigorously stirred at rt till TLC indicated the completion of the reaction. The solvent was removed under reduced pressure and the residue was directly subjected to column chromatography to afford **49** as a colorless oil in 55% yield in two steps, using hexanes/EtOAc as eluent. (56.4 mg, 55%)

^{1}H NMR (501 MHz, $CDCl_3$) δ 7.29 (t, *J* = 7.5 Hz, 2H), 7.23–7.15 (m, 3H), 5.48 (s, 1H), 3.44 (d, *J* = 8.4 Hz, 1H), 3.32 (d, *J* = 8.4 Hz, 1H), 2.83–2.67 (m, 2H), 2.05–1.98 (m, 2H), 1.52 (s, 3H).

^{13}C NMR (126 MHz, $CDCl_3$) δ 159.3, 141.1, 128.7, 128.4, 126.3, 82.9, 51.3, 42.3, 29.9, 25.8.

R_f = 0.22 (Ethyl acetate/hexanes = 1:1).

EI-HRMS (*m/z*): calculated for $C_{12}H_{15}N_1O_2$ [M$^{+•}$]: 205.1097, found: 205.1095.

HPLC (AD-3, isopropanol: heptane = 5:95, 1.0 mL/min, 298 K, 190 nm): t_{R1} = 9.5 min (major), t_{R2} = 10.6 min (minor), e.r. = 95:5.

$[\alpha]_D^{25}$ = –1.6 (c 0.64, $CHCl_3$).

Formal synthesis of COX-2 inhibitor

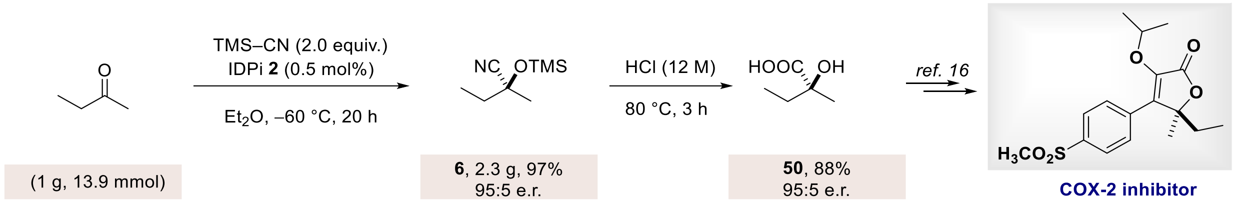

Step 1. TMSCN **1** (3.5 mL, 27.8 mmol, 2.0 equiv.) was placed in a 100 mL round-bottom flask equipped with a teflon-coated magnetic stirring bar. IDPi **2** (125.7 mg, 0.069 mmol, 0.005 equiv.) and diethylether (0.4 M, 34.8 mL) were added, afterwards the resultant solution was stirred at rt for 3 h and at –60 °C for 0.5 h. 2-Butanone (1.0 g, 13.9 mmol, 1.0 equiv.) was slowly added and the reaction mixture was stirred for an additional 20 h at the indicated temperature. After the ketone was fully consumed, as monitored by TLC, the reaction mixture was treated with three drop of triethylamine via pipet. Volatiles were removed in *vacuo* and the residue was purified by column chromatography with neutral aluminium oxide (activated by 20 wt % water and washed by trimethylamine followed by 200 mL pentane) padded in 13 cm height and 3 cm width, using 250 mL 5% diethylether in pentane as eluent to afford the product **6** in 97% isolated yield (fraction of test tubes (16 cm height and 1.5 cm width) 3–8 was collected). The enantiomeric ratio (e.r.) was determined by chiral GC analysis. (2.3 g, 95:5 e.r.).

Step 2. A solution of **6** (50.0 mg, 0.29 mmol) in conc. HCl (2 mL) was stirred at 80 °C for 3 h. After cooling, the solution was concentrated in *vacuo* and the residue was treated with water (5 mL) and ethyl acetate (2 mL). The aqueous phase was extracted with ethyl acetate (3 ×2 mL), and the combined solution was dried over sodium sulfate and concentrated in *vacuo*. Purification by column chromatography provided **50** as off white solid. (30.0 mg, 88%, 95:5 e.r.) [15]

Characterization of (*S*)-2-hydroxy-2-methylbutanoic acid 50

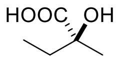

^{1}H NMR (501 MHz, $CDCl_3$) δ 1.85 (dd, J = 14.2, 7.3 Hz, 1H), 1.72 (dd, J = 14.0, 7.4 Hz, 1H), 1.47 (s, 3H), 0.94 (t, J = 7.5 Hz, 3H).

^{13}C NMR (126 MHz, $CDCl_3$) δ 182.2, 75.2, 33.1, 25.6, 8.0.

R_f = 0.32 (Ethyl acetate/hexanes = 1:4).

CI-HRMS (*m/z*): calculated for $C_5H_{11}O_3$ [M+H$^+$]: 119.0702, found: 119.0704.

HPLC (IA, isopropanol: heptane:TFA = 2:98:0.1, 1.0 mL/min, 298 K, 220 nm): t_{R1} = 9.8 min (major), t_{R2} = 8.7 min (minor), e.r. = 95:5.

$[\alpha]_D^{25}$ = 7.8 (c 0.87, $CHCl_3$). Lit[16]: (>99% *ee*), for (*S*)-product, $[\alpha]_D^{25}$ = 9.1 (c 1.6, $CHCl_3$). Meanwhile, the absolute configuration of cyanohydrin silyl ether **6** was further confirmed to be *S* by the HPLC traces comparison between the known (*R*)-2-hydroxy-2-methylbutanoic acid (left) and the derived product from **6** (right).

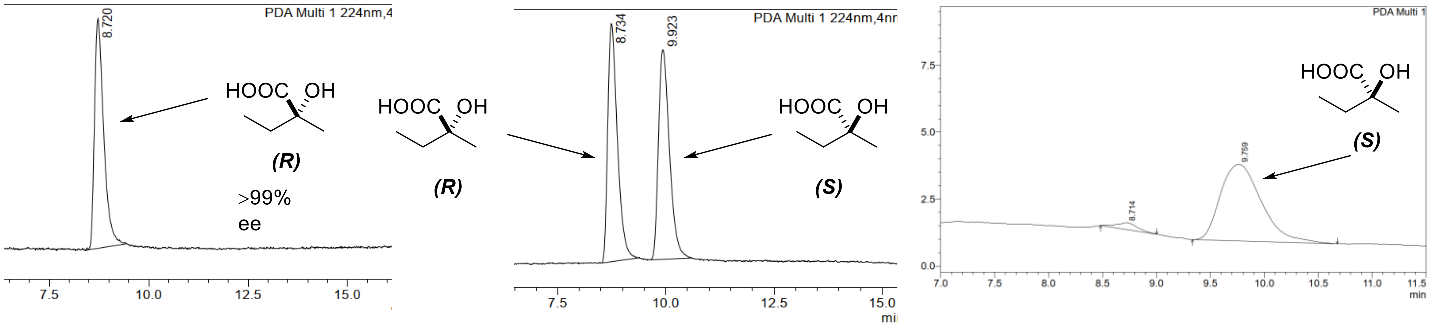

7. Mechanistic studies

(1). Procedure of reactivity comparison of reactions catalyzed by DSI-I and confined IDPi catalyst **4**:

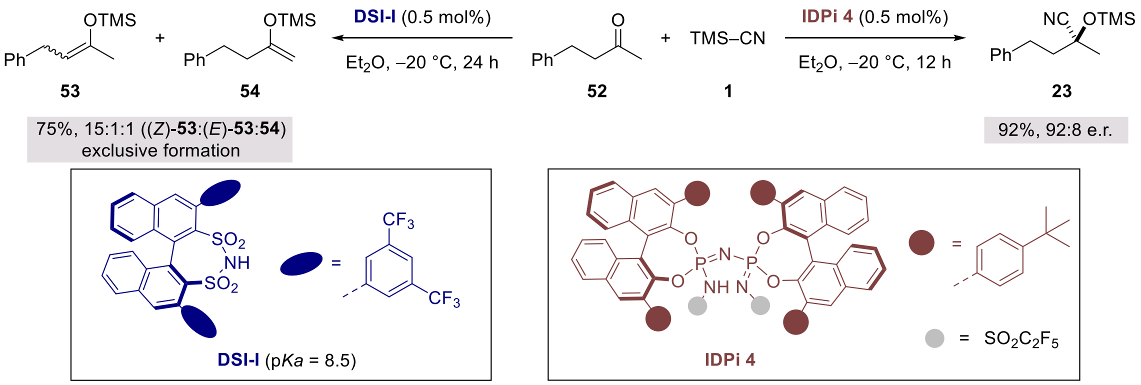

Reaction with DSI-I: TMSCN **1** (0.4 mmol, 2.0 equiv.) was placed in a GC vial, which was equipped with a teflon-coated magnetic stirring bar. DSI-I (0.005 equiv.) and Et_2O (0.2 M, 1.0 mL) were added, afterwards the resultant solution was stirred at rt for 5 min then lowered to –20 °C for 10 min. Ketone **52** (30.0 µL, 0.2 mmol, 1.0 equiv.) was slowly added and the reaction mixture was stirred for an additional 24 h at the indicated temperature. Then the reaction mixture was treated with one drop of triethylamine via pipet. Volatiles were removed in *vacuo* and the residue was purified by column chromatography. The species of the mixture were confirmed by GC-MS analysis and the ratio of the products were determined by NMR analysis.

Reaction with IDPi **4**: TMSCN **1** (0.4 mmol, 2.0 equiv.) was placed in a GC vial, which was equipped with a teflon-coated magnetic stirring bar. IDPi **4** (0.005 equiv.) and Et_2O (0.2 M, 1.0 mL) were added, and the resultant solution was stirred at rt for 30 min then lowered to –20 °C for 10 min. Ketone **52** (30.0 µL, 0.2 mmol, 1.0 equiv.) was slowly added and the reaction mixture was stirred for an additional 12 h at the indicated temperature. After the ketone was fully consumed, as monitored by TLC, the reaction mixture was treated with one drop of triethylamine via pipet. Volatiles were removed in *vacuo* and the residue was purified by column chromatography. The enantiomeric ratio (e.r.) was determined by HPLC analysis.

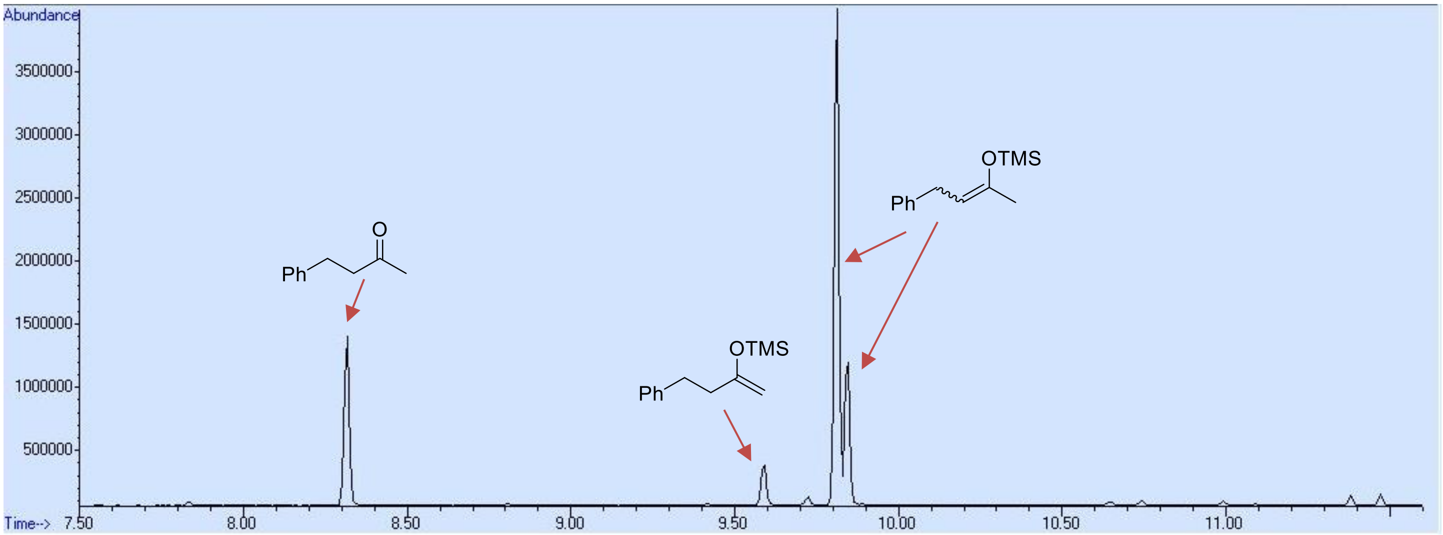

Figure S8. GC spectrum of the reaction catalyzed by DSI-I.

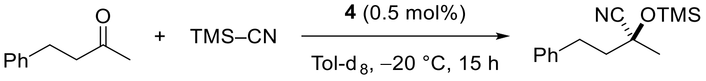

52 **1** **23**

Procedure: TMSCN **1** (18.8 µL, 0.15 mmol, 1.5 equiv.), tol-d_8 (0.5 mL, 0.2 M), and IDPi **4** (0.005 equiv.) were placed in a NMR tube. The resultant solution was kept at rt for 1 h. Ketone **52** (15 µL, 0.1 mmol, 1.0 equiv.) was added to the reaction mixture at –78 °C (dry ice), and after quick mixing, the sample was transferred to a 5mm BBFO NMR probe precooled to –20 °C in the Bruker AVIII 300WB NMR spectrometer. The reaction was monitored at the indicated temperature and enol silanes were captured by ^{1}H NMR analysis. The NMR data was imported with the *Reaction Monitoring Plugin* into MNOVA 14.1.2 and processed therein (phase correction, baseline correction).

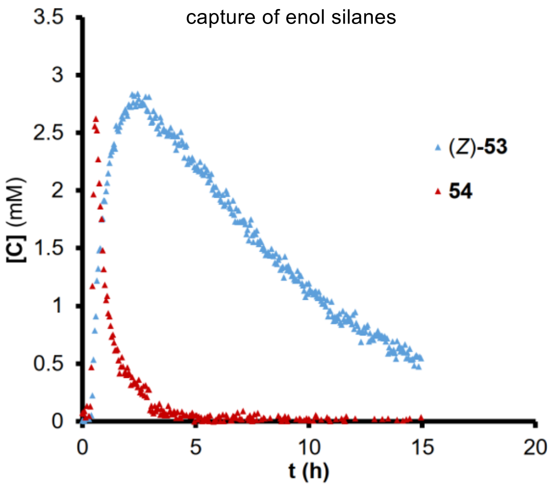

Figure S9. Enol silanes formation captured by ^{1}H NMR in the reaction of 4-phenylbutan-2-one and TMSCN catalyzed by IDPi **4**.

(2). Reaction of silyl enol ethers mixture with HCN catalyzed by catalyst IDPi **4**:

In-situ HCN synthesis monitored by ^{1}H NMR: MeOH (0.4 mmol), TMSCN (0.5 mmol), tol-d_8 (0.5 mL, 1.0 M), and **4** (0.5 mol%) were placed in a NMR tube at rt and the reaction was monitored by ^{1}H NMR.

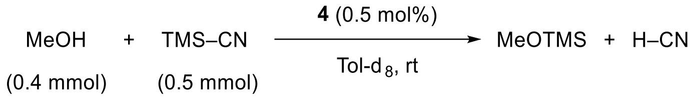

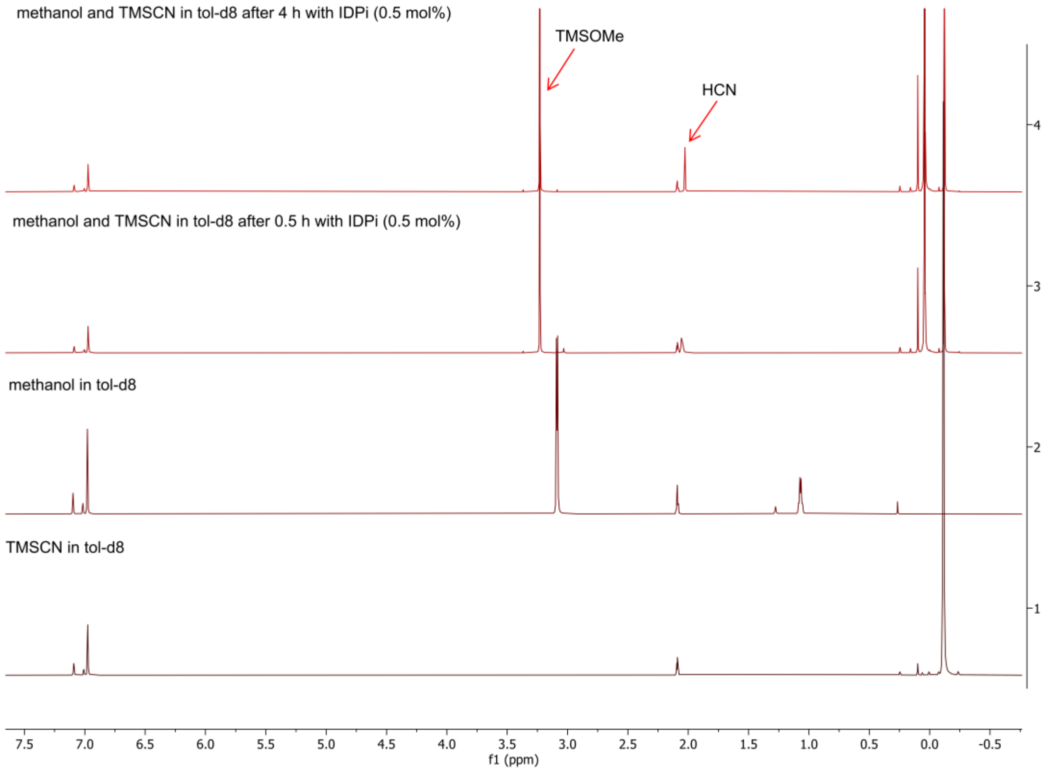

Figure S10. *In-situ* synthesis of HCN from the reaction of methanol and TMSCN.

Note: The comparison experiment was also performed without IDPi catalyst, in which the formation of HCN occurred with a slower rate.

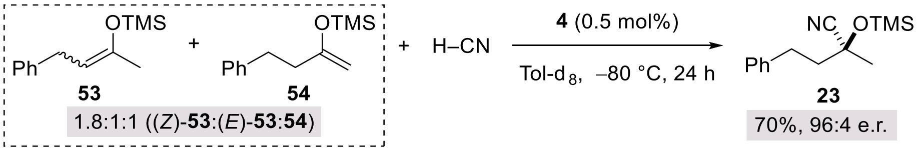

Procedure: To the mixture of HCN solution (0.4 M in tol-d_8 prepared according to the procedure shown above) in a GC vial that was equipped with a teflon-coated magnetic stirring bar, silyl enol ether mixture **53** and **54** (0.2 mmol) was added at –80 °C and the reaction mixture was stirred for an additional 24 h at the indicated temperature. Then the reaction mixture was treated with one drop of triethylamine via pipet and the volatiles were removed in *vacuo* and the residue was purified by column chromatography. The e.r. was determined to be 96:4.

In order to get a deeper understanding of this unusual low yield, we monitored the reaction with ^{1}H NMR shown below:

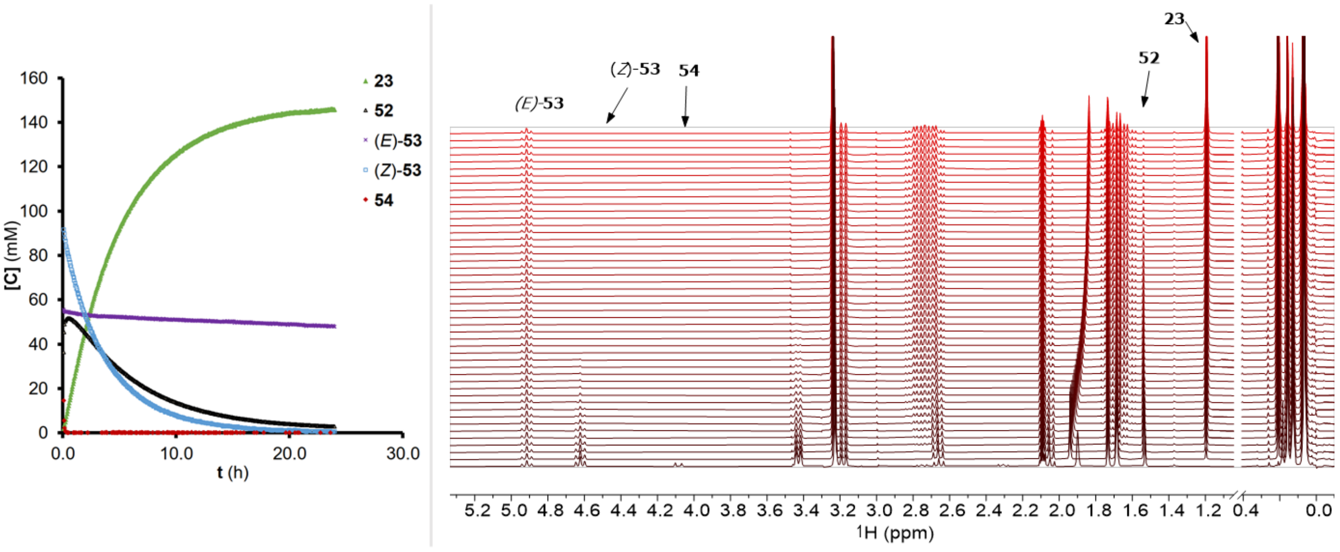

Figure S11. Reaction plots of the reaction of silyl enol ethers **53**, **54** and HCN.

Results: Enol silane **54** reacted with HCN in the fastest manner, and was soon found to be fully consumed within 10 min. The reaction of *Z*-enol silane was completed in 20 h, while the *E*-enol silane hardly reacted with HCN under the standard conditions which well explained the incomplete consumption of the starting material.

Kinetic studies

The kinetic studies were performed with NMR spectroscopy.

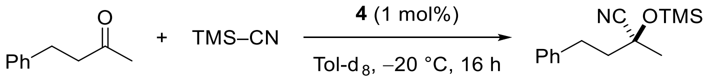

52 **1** **23**

Sample preparation:

Stock solution of TMSCN: TMSCN 1 (251 µL, 2.0 mmol) in tol-d$_8$ (1.0 mL, 2 M).

Stock solution of 4: 4 (7.8 mg, 0.005 mmol) in tol-d$_8$ (0.25 mL, 0.02 M).

Procedure: TMSCN **1** (75 µL, 0.15 mmol, 1.5 equiv.), tol-d$_8$ (0.45 mL, 0.2 M), and **4** (50 µL, 0.01 equiv.) were placed in a NMR tube. The resultant solution was kept at rt for 1 h. Ketone **52** (15 µL, 0.1 mmol, 1.0 equiv.) was added to the reaction mixture at –78 °C (dry ice), and after quick mixing, the sample was transferred to a 5mm BBFO NMR probe precooled to –20 °C in the Bruker AVIII 300WB NMR spectrometer. The reaction was monitored at the indicated temperature until the starting material was fully consumed. The NMR data was imported with the *Reaction Monitoring Plugin* into MNOVA 14.1.2 and processed therein (phase correction, baseline correction). The concentration graphs were generated by integration of signals with no overlap and referenced to the initial concentration of the ketone (162.6 mM).

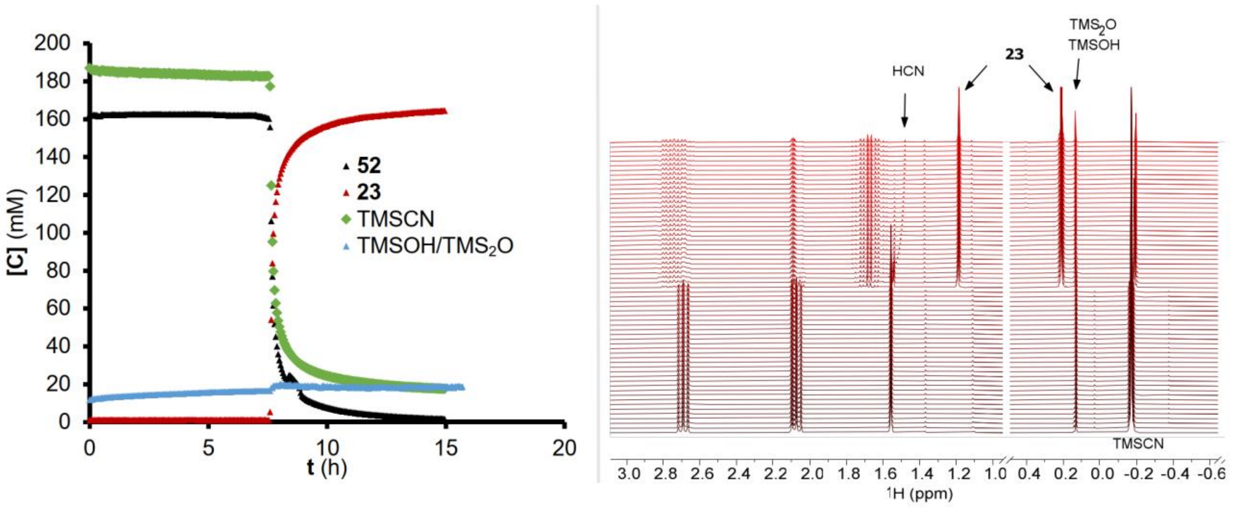

Figure S12. *left*: NMR concentration profile showing the dormant period of 7.5 h and the reaction time of 7 h; *right*: ^{1}H NMR at different time points during the reaction showing the product formation.

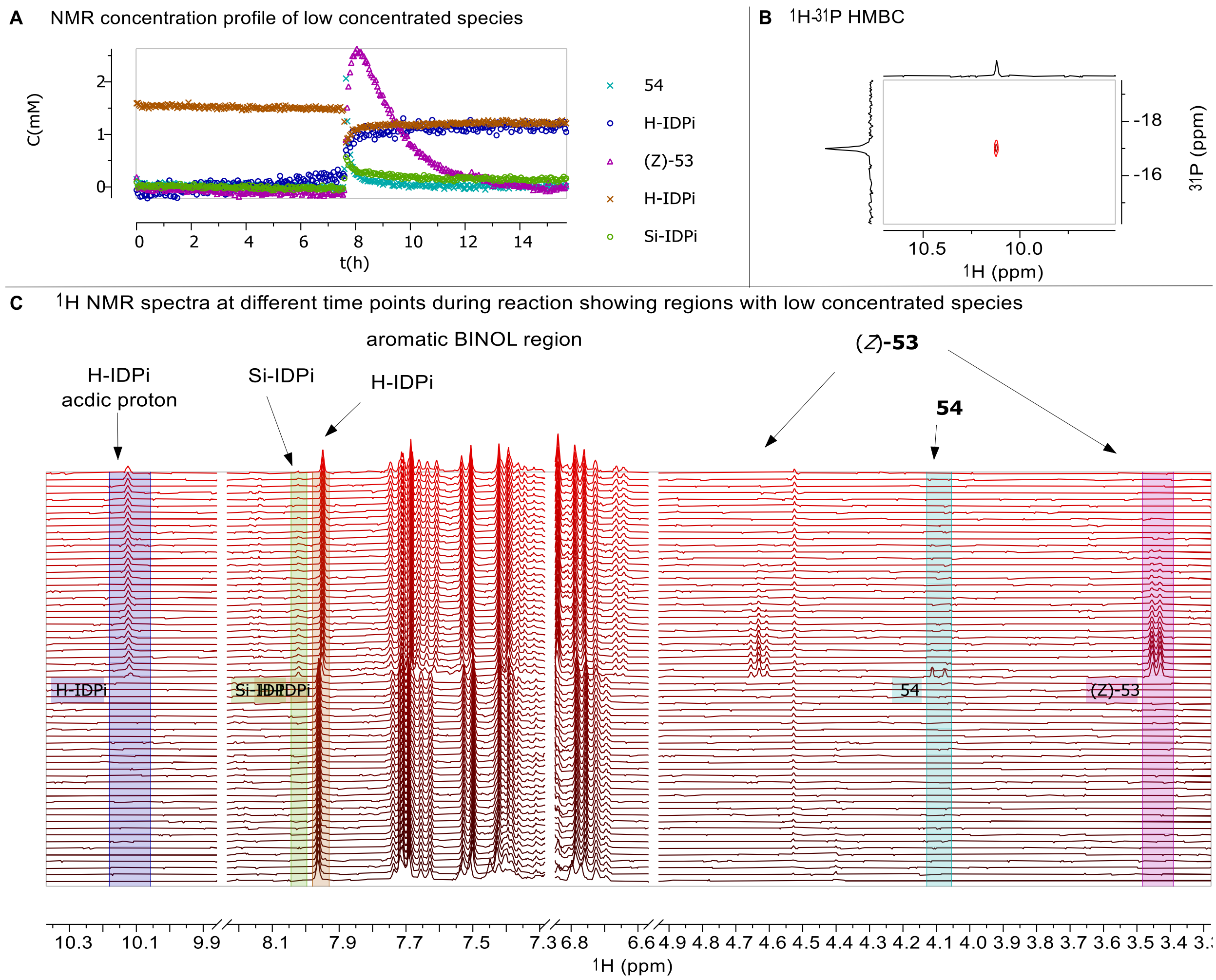

Figure S13. A: NMR concentration profile low concentrated species present during the reaction; **B**: ^{1}H-^{31}P HMBC spectrum showing the coupling of the acidic proton at $\delta_H = 10.15$ ppm with the ^{31}P signal ($\delta_P = -17.0$ ppm) of **4**-H; **C**: ^{1}H NMR at different time points during the reaction showing the regions of species in low concentration (catalyst, enol silanes).

Results: During the first 7.5 h of NMR monitoring, no product formation was observed. Instead, only the conversion of TMSCN with the concomitant formation of HCN, TMSOH, and TMSOTMS occurred. After that, the reaction initiated and was fully converted within another 7 h (see **Figure S12**). Besides the main species, small amounts of (*Z*)-**53** and **54** were observed during the reaction, which were also fully consumed after the reaction completed. Interestingly, two different catalyst species (4-**H** and 4-**TMS**) can be observed in parallel during the reaction.

Determination of the reaction order of IDPi catalyst 4.

Sample preparation:

Stock solution of TMSCN: TMSCN 1 (251 µL, 2.0 mmol) in tol-d$_8$ (1.0 mL, 2 M).

Stock solution of 4: 4 (7.8 mg, 0.005 mmol) in tol-d$_8$ (0.25 mL, 0.02 M).

Procedure: TMSCN **1** (75 µL, 0.15 mmol, 1.5 equiv.), tol-d$_8$ (0.45–0.475 mL, 0.2 M), and **4** were placed in a NMR tube. Three different samples were prepared from the stock solutions with 0.25, 0.5 and 1 mol% of catalyst **4** at –20°C in tol-d$_8$, and the resultant solution was kept at rt for 1 h. Ketone **52** (15 µL, 0.1 mmol, 1.0 equiv.) was added to the reaction mixture at –78 °C (dry ice), and after quick mixing, the sample was transferred to a 5mm BBFO NMR probe precooled to –20 °C in the Bruker AVIII 300WB NMR spectrometer. The reaction mixture was monitored at the indicated temperature until the starting material was fully consumed. The NMR data was imported with the *Reaction Monitoring Plugin* into MNOVA 14.1.2 and processed therein (phase correction, baseline correction). The concentration graphs were generated by integration of signals with no overlap and referenced to the initial concentration of the ketone (162.6 mM). *Note: The individual reaction showed differences in the dormant period and t=0 was set to the point where the reaction started.*

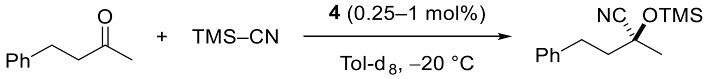

52 **1** **23**

The catalyst order was determined by time normalization analysis reported by the Bures group.[17–21] When comparing conversion plots normalized to different catalyst orders, the curves were found to overlap nicely when a first order with respect to the catalyst was assumed, implying no significant influence from off-cycle equilibrium, synergistic effects or the presence of different catalyst species in solution.

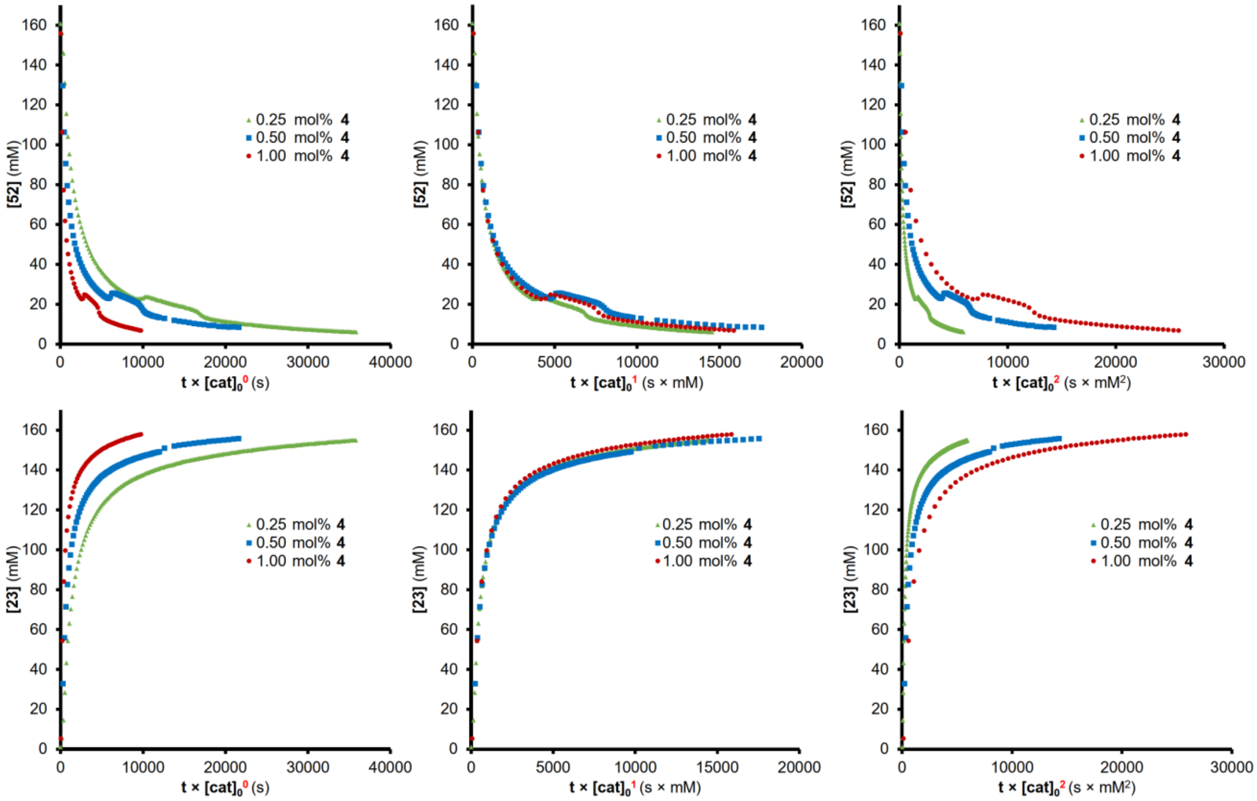

Figure S14. Conversion plots obtained from NMR measurements with time scales normalized to different catalyst orders: the first row shows the consumption of **52**, zero-order reaction (left); first-order reaction (middle); second-order reaction (right). The second row indicated by the formation of **23**, zero-order reaction (left); first-order reaction (middle); second-order reaction (right).

Determination of the reaction order of TMSCN 1

Sample preparation:

Stock solution of TMSCN: TMSCN 1 (251 µL, 2.0 mmol) in tol-d$_8$ (1.0 mL, 2 M).

Stock solution of 4: 4 (7.8 mg, 0.005 mmol) in tol-d$_8$ (0.25 mL, 0.02 M).

Procedure: TMSCN **1**, tol-d$_8$ (0.27–0.45 mL, 0.2 M), and **4** (0.25 mol%) were placed in a NMR tube. Three different samples were prepared from the stock solutions with 1.5, 2.3 and 3 equiv. of TMSCN **1** at –20°C in tol-d$_8$, and the resultant solution was kept at rt for 1 h. Ketone **52** (15 µL, 0.1 mmol, 1.0 equiv.) was added to the reaction mixture at –78 °C (dry ice), and after quick mixing, the sample was transferred to a 5 mm BBFO NMR probe precooled to –20 °C in the Bruker AVIII 300WB NMR spectrometer. The reaction mixture was monitored at the indicated temperature until the starting material was fully consumed. The NMR data was imported with the *Reaction Monitoring Plugin* into MNOVA 14.1.2 and processed therein (phase correction, baseline correction). The concentration graphs were generated by integration of signals with no overlap and referenced to the initial concentration of the ketone (162.6 mM). *Note: The individual reaction showed differences in the dormant period and t=0 was set to the point where the reaction started.*

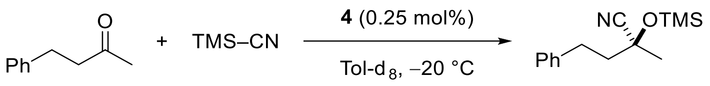

52 **1** **23**

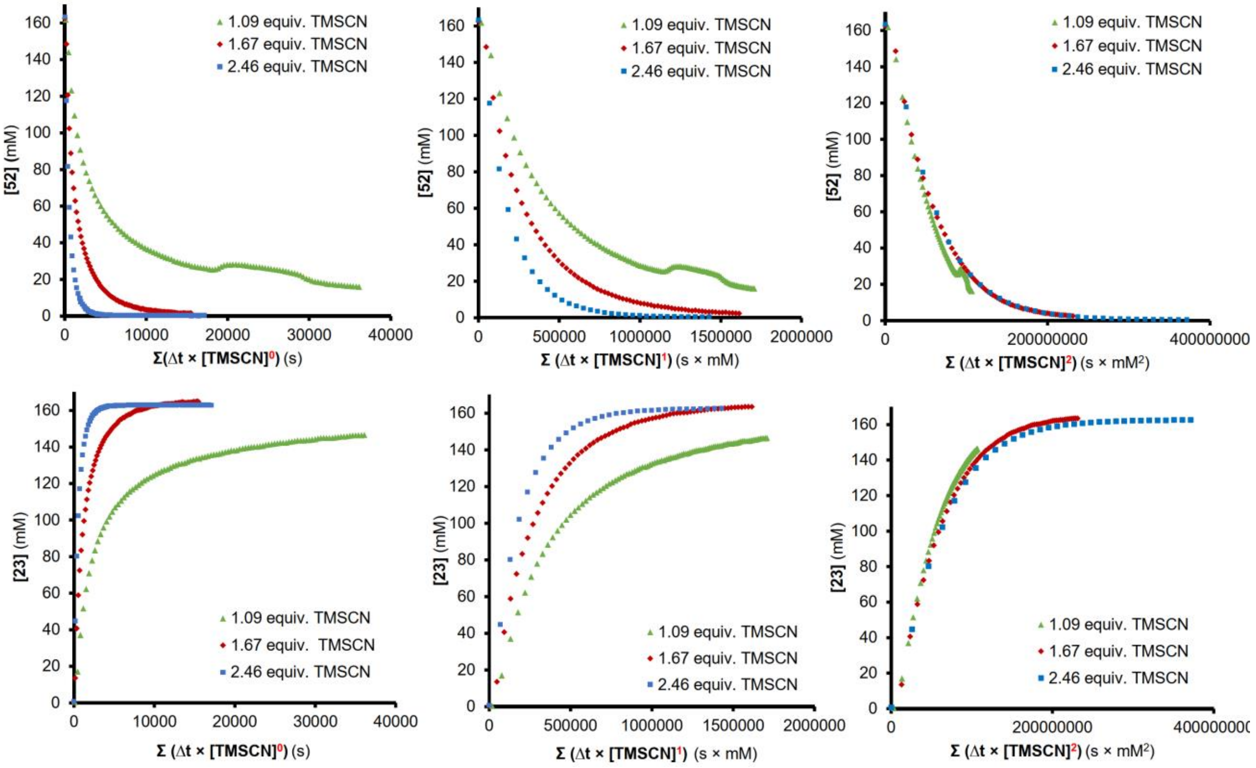

Figure S15. Conversion plots obtained from NMR measurements with time scales normalized to different orders of TMSCN **1**. The first row shows the consumption of **52**, zero-order reaction (left); first-order reaction (middle); second-order reaction (right). The second row shows the formation of **23**, zero-order reaction (left); first-order reaction (middle); second-order reaction (right).

The order of the TMSCN **1** was also determined by variable time normalization analysis (VTNA). Reaction plots normalized to different reaction order of TMSCN can be found in **Figure S15**. By assuming different orders the best overlap was found for a second order dependence on TMSCN **1.** This result is in agreement with previous kinetic studies for the DSI catalyzed cyanosilylation of aldehydes.[22]

Determination of the reaction order of ketone 52

Sample preparation:

Stock solution of TMSCN: TMSCN 1 (251 µL, 2.0 mmol) in tol-d$_8$ (1.0 mL, 2 M).

Stock solution of 4: 4 (7.8 mg, 0.005 mmol) in tol-d$_8$ (0.25 mL, 0.02 M).

Procedure: TMSCN **1** (100 µL, 0.2 mmol, 2.0 equiv.), tol-d$_8$ (0.45 mL, 0.2 M), and **4** (0.5 mol%) were placed in a NMR tube. The resultant solution was kept at rt for 1 h. Three different samples were prepared with 0.25, 0.5 and 1 equiv. of Ketone **52** at –78 °C (dry ice), and after quick mixing, the sample was transferred to a 5 mm BBFO NMR probe precooled to –20 °C in the Bruker AVIII 300WB NMR spectrometer. The reaction mixture was monitored at the indicated temperature until the starting material was fully consumed.

The NMR data was imported with the *Reaction Monitoring Plugin* into MNOVA 14.1.2 and processed therein (phase correction, baseline correction). The concentration graphs were generated by integration of signals with no overlap and referenced to the initial concentration of the ketone (162.6 mM, 81.3 mM and 40.65 mM). *Note: The individual reaction showed differences in the dormant period and t=0 was set to the point where the reaction started.*

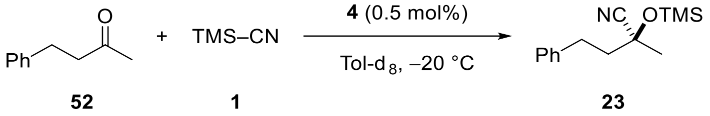

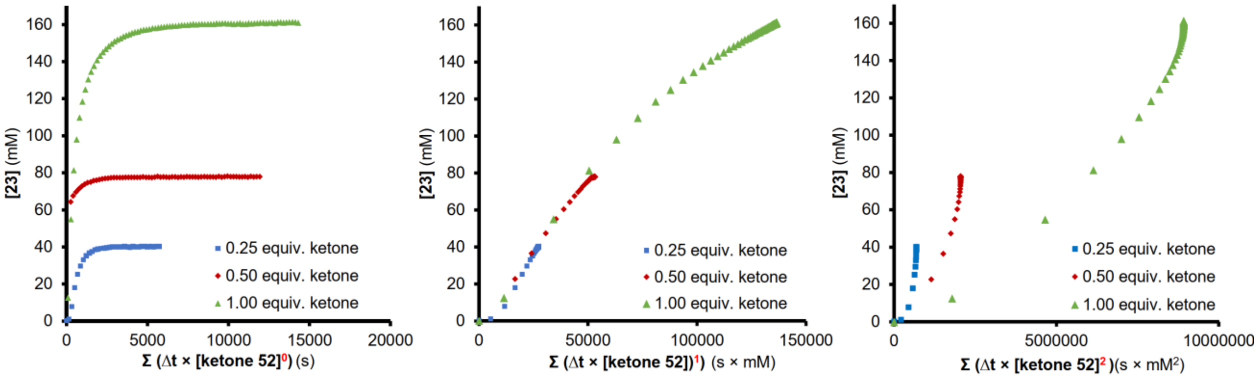

Figure S16. Conversion plots obtained from NMR measurements with time scales normalized to different orders of ketone **52**. The graphs show the formation of **23**, normalized to a zero-order reaction (left); first-order reaction (middle); second-order reaction (right).

The order of the ketone **52** was also determined by variable time normalization analysis (VTNA). Reaction plots normalized to different reaction order of ketone can be found in **Figure S16**. By assuming different ketone orders the best overlap was found for a first order dependence on ketone **52**. This result is in contrast to our previous kinetic studies for the DSI catalyzed cyanosilylation of aldehydes, where the reaction was found to be nearly zeroth order in concentration of the aldehyde. In our reaction system the result suggests, that one molecular of ketone is involved in the turnover-limiting step.

8. Characterization and study of the equilibrium between catalyst 4-H and 4-TMS

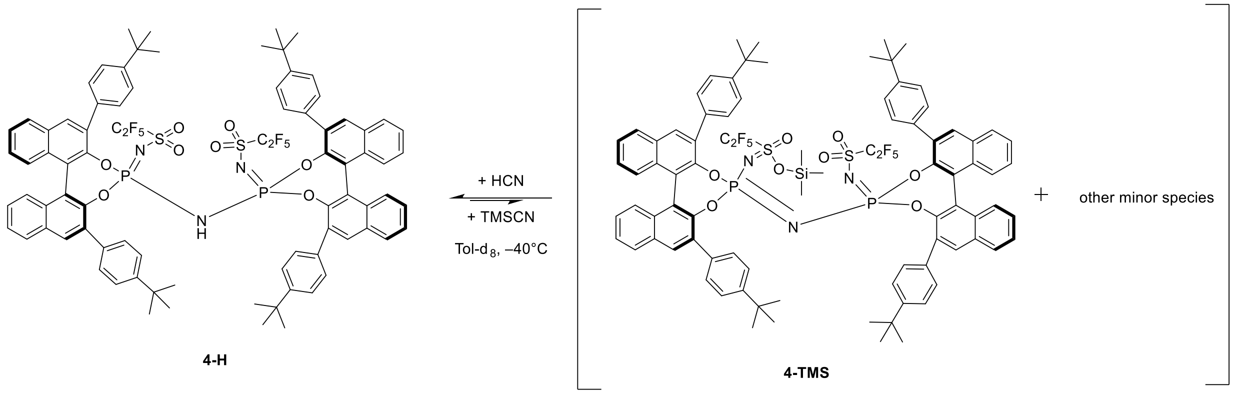

To a NMR tube containing IDPi **4** (10 mg) in tol-d_8, TMSCN **1** (5.0–15.0 equiv.) was added at room temperature and the resultant mixture were characterized at –40 °C. ^{31}P NMR spectra in presence of different equivalents of TMSCN (**Figure S17**) and as well as ^{1}H NMR spectra (**Figure S18**) showed the change of the ratio between the protonated IDPi **4**-H and silylated IDPi **4**-TMS. Additionally,

EXSY cross peaks in ^{31}P-^{31}P NOESY (**Figure S19**) and ^{1}H-^{1}H-ROESY (**Figure S20**) spectra showed the chemical interconversion of the two species on a 200 ms timescale. The ^{31}P-^{31}P NOESY data also revealed additional minor species. The observed reversible equilibrium explains the observed higher order of TMSCN: The addition of higher amounts of TMSCN influences the rate of product formation, but also increases the relative amount of active catalyst species **4**-TMS in solution. This reversible equilibrium has not been considered previously, but might also explain the kinetic data of the DSI catalyzed cyanosilylation of aldehydes.[22]

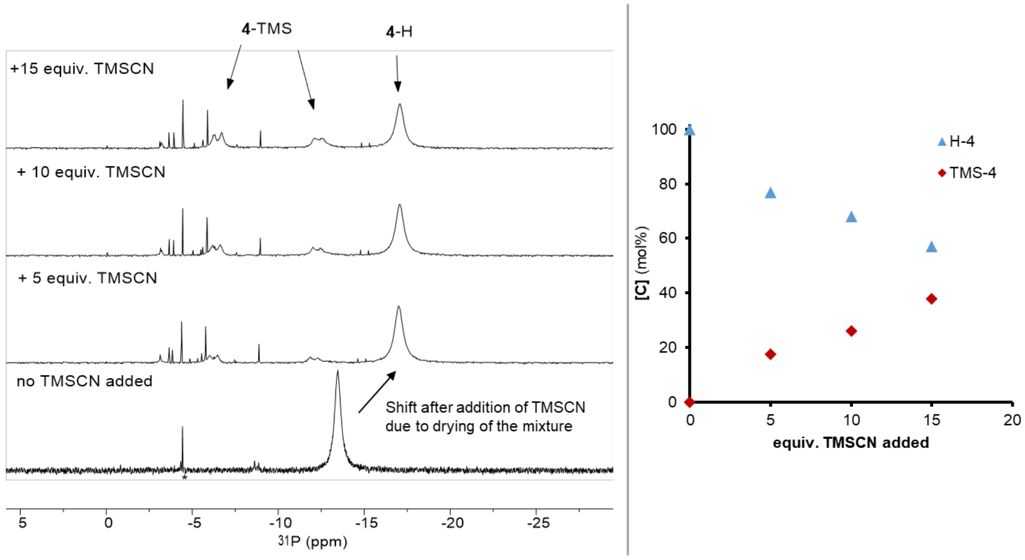

Figure S17. left: ^{31}P NMR spectra of catalyst **4** with different equivalents of TMSCN added; right: graph showing changes in the composition of the two main catalyst species present in the mixture in presence of different equivalents of TMSCN.

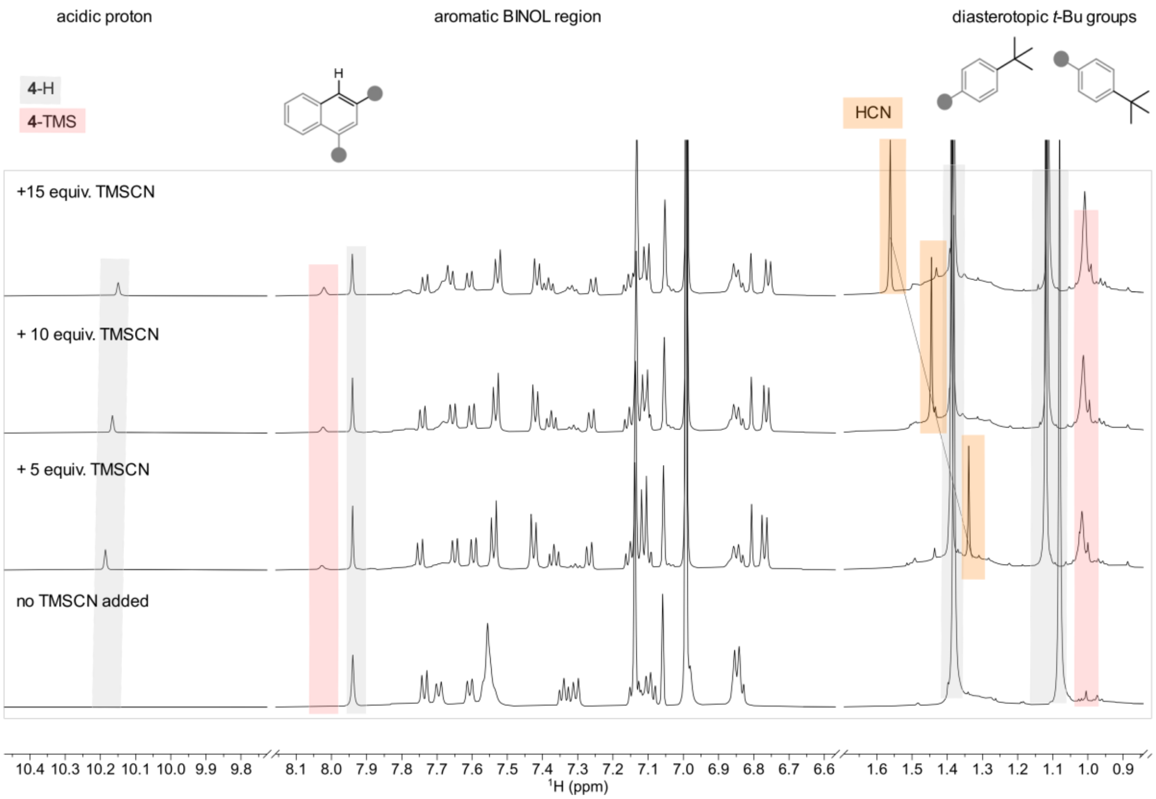

Figure S18. ^{1}H NMR spectra showing the signals of catalyst **4** with different equivalents of TMSCN. Regions with the excess of TMSCN and regions without signals are removed for clarity.

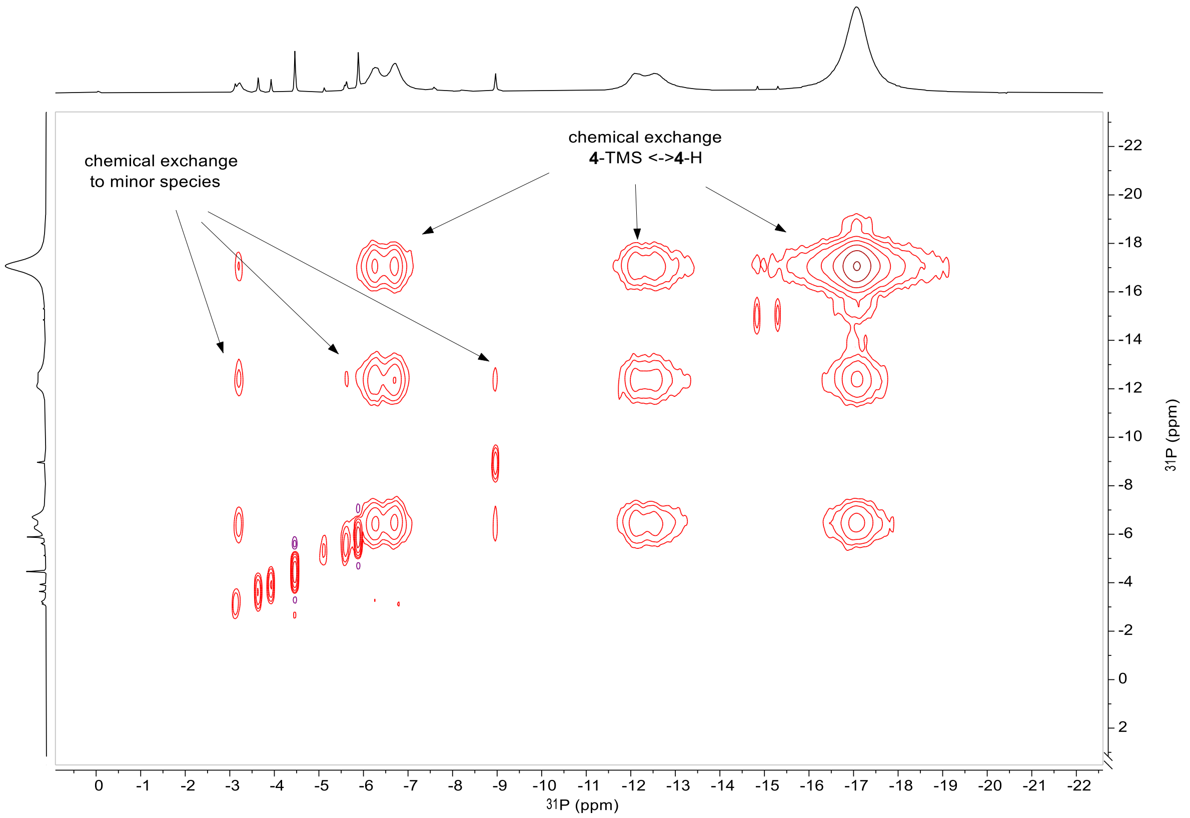

Figure S19. ^{31}P - ^{31}P NOESY spectra showing the chemical interconversion of the catalyst species present in solution (mixing time = 200ms).

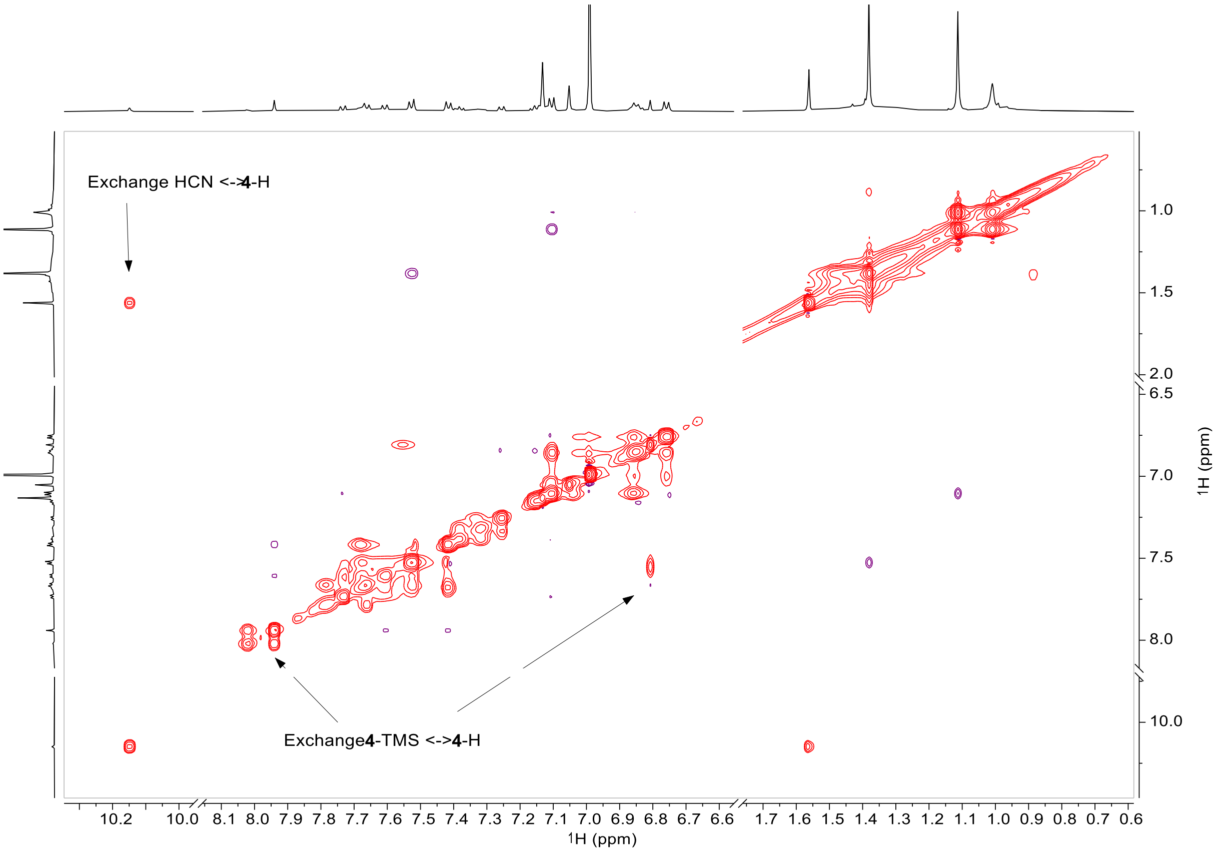

Figure S20. ^{1}H - ^{1}H EASY-ROESY spectrum (τ_{SL} = 200ms), red cross peaks show chemical interconversion of showing the chemical interconversion of the catalyst species present in solution (EXSY), blue cross peaks show spatial proximity of different protons (ROE).

9. Preparation and characterization of imidodiphosphorimidates (IDPis)[3-4]

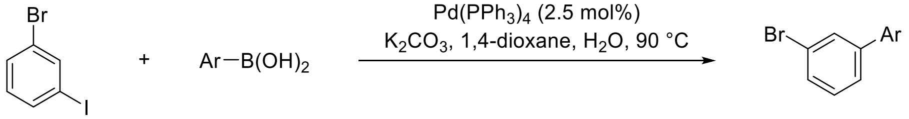

Procedure for 3-Ar bromide synthesis using Suzuki coupling strategy:

In a flame dried 2-neck round-bottom flask with a condenser, 1-bromo-3-iodobenzene (0.51 mL, 4.0 mmol, 1.0 equiv.), Ar-B(OH)$_2$ (4 mmol), and tetrakis(triphenylphosphine)palladium (115.6 mg, 0.1 mmol, 0.025 equiv.) were dissolved in 1,4-dioxane (10 mL). After degassing the reaction mixture with argon for 10–15 min, a degassed aqueous solution of K_2CO_3 (2.0 M, 4.0 mL) was added. The resultant mixture was then transferred into a microwave reactor and stirred at 90 °C for 2 h. After cooling the reaction system to room temperature, the mixture was further purified by column chromatography to afford Ar-Br (66.5% yield for 3-bromo-4'-(trifluoromethyl)-1,1'-biphenyl, 85% yield for 3-(3-bromophenyl)thiophene, respectively).

3-bromo-4'-(trifluoromethyl)-1,1'-biphenyl

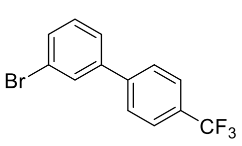

^1H NMR (501 MHz, $CDCl_3$) δ 7.74 (t, *J* = 1.9 Hz, 1H), 7.71 (d, *J* = 8.3 Hz, 2H), 7.66 (d, *J* = 8.2 Hz, 2H), 7.53 (ddt, *J* = 9.6, 7.9, 1.6 Hz, 2H), 7.34 (t, *J* = 7.9 Hz, 1H).

^{13}C NMR (126 MHz, $CDCl_3$) δ 143.4, 142.0, 131.3, 130.6, 130.5, 130.3, 130.0, 127.6, 126.1, 126.0, 125.4, 123.3.

^{19}F NMR (471 MHz, $CDCl_3$) δ –62.5.

R_f = 0.62 (hexanes).

EI-HRMS (*m*/*z*): calculated for $C_{13}H_8F_3Br_1$ [$M^{+\bullet}$]: 299.9756, found: 299.9762.

3-(3-bromophenyl)thiophene

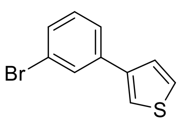

^1H NMR (501 MHz, $CDCl_3$) δ 7.74 (t, *J* = 1.9 Hz, 1H), 7.52 (ddd, *J* = 7.7, 1.7, 1.0 Hz, 1H), 7.46 (dd, *J* = 2.9, 1.3 Hz, 1H), 7.44–7.38 (m, 2H), 7.36 (dd, *J* = 5.0, 1.4 Hz, 1H), 7.26 (t, *J* = 7.9 Hz, 1H).

^{13}C NMR (126 MHz, $CDCl_3$) δ 141.0, 138.0, 130.4, 130.2, 129.6, 126.7, 126.3, 125.2, 123.1, 121.3.

R_f = 0.36 (hexanes).

EI-HRMS (*m*/*z*): calculated for $C_{10}H_7S_1Br_1$ [$M^{+\bullet}$]: 237.9446, found: 237.9449.

Representative procedure for 3,3'-disubstituted BINOL synthesis using Suzuki coupling strategy and the subsequent hydrolysis:

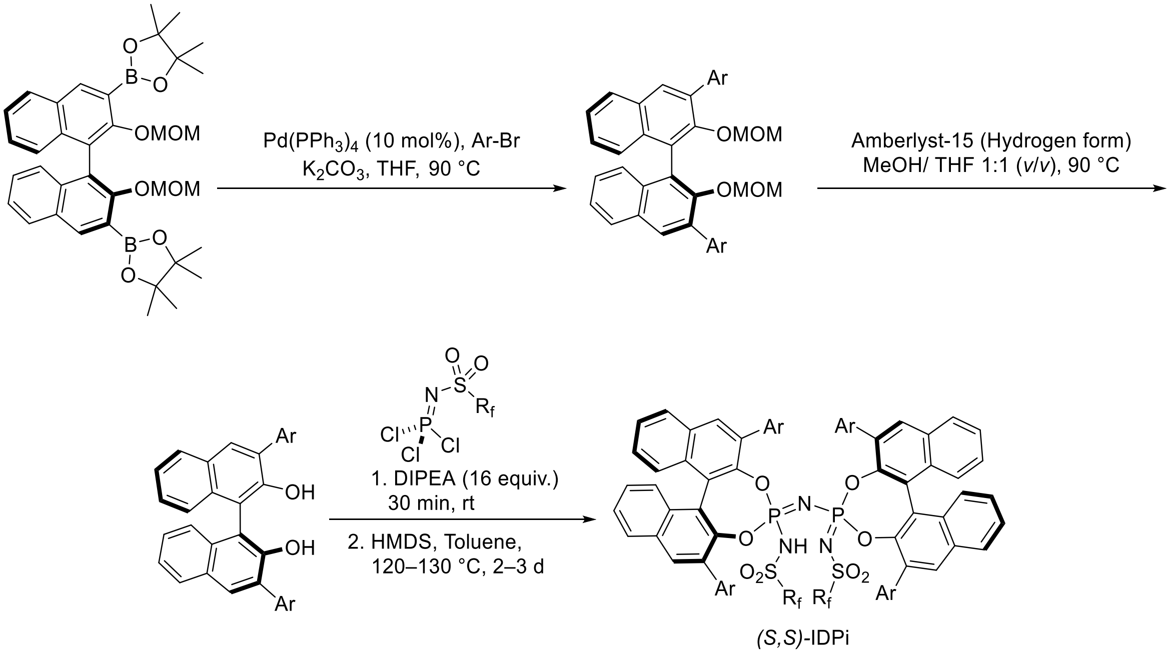

In a flame dried 2-neck round-bottom flask with a condenser, (*S*)-2,2'-(2,2'-bis(methoxymethoxy)-1,1'-binaphthyl-3,3'-diyl)bis(4,4,5,5-tetramethyl-1,3,2-dioxaborolane) (500 mg, 0.8 mmol, 1.0 equiv.), 3-bromo-4'-(trifluoromethyl)-1,1'-biphenyl (554 mg, 2.3 equiv.), and tetrakis(triphenylphosphine)palladium (92 mg, 0.08 mmol, 0.1 equiv.) were dissolved in 1,4-dioxane (10 mL). After degassing the reaction mixture with argon for 10–15 min, a degassed aqueous solution of K_2CO_3 (2.0 M, 3.0 mL) was added. The resultant mixture

was then transferred into a microwave reactor and stirred at 90 °C for 2 h. After cooling the reaction system to room temperature, the mixture was further purified by column chromatography to afford (*S*)-2,2'-bis(methoxymethoxy)-3,3'-bis(4'-(trifluoromethyl)-[1,1'-biphenyl]-3-yl)-1,1'-binaphthalene. A solution of this material and Amberlyst®-15 (Hydrogen form) in THF/MeOH (1:1) was then stirred at 90 °C for 12 h. After completion of the reaction (as monitored by TLC analysis), Amberlyst®-15 was removed by filtration, and the mixture was concentrated under reduced pressure. Finally, the crude mixture was purified by column chromatography (ethyl acetate/hexanes 1:20 to 1:4) to afford (*S*)-3,3'-bis(4'-(trifluoromethyl)-[1,1'-biphenyl]-3-yl)-[1,1'-binaphthalene]-2,2'-diol as white solid (overall yield of the two steps: 85 %).

(*S*)-2,2'-bis(methoxymethoxy)-3,3'-bis(4'-(trifluoromethyl)-[1,1'-biphenyl]-3-yl)-1,1'-binaphthalene

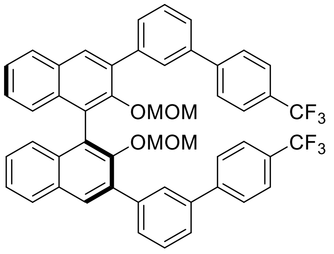

^{1}H NMR (501 MHz, $CDCl_3$) δ 8.08–8.02 (m, 4H), 7.95 (dd, *J* = 8.2, 1.1 Hz, 2H), 7.86 (dt, *J* = 7.4, 1.6 Hz, 2H), 7.82 (d, *J* = 8.1 Hz, 4H), 7.75 (d, *J* = 8.5 Hz, 4H), 7.68–7.57 (m, 4H), 7.47 (ddd, *J* = 8.1, 6.2, 1.8 Hz, 2H), 7.40–7.31 (m, 4H), 4.52 (d, *J* = 5.8 Hz, 2H), 4.48 (d, *J* = 5.8 Hz, 2H), 2.44 (s, 6H).

^{13}C NMR (126 MHz, $CDCl_3$) δ 151.5, 144.8, 140.1, 140.0, 135.2, 133.9, 131.0, 130.8, 129.8, 129.5, 129.1, 128.6, 128.1, 127.7, 126.7, 126.6, 126.4, 126.0, 125.9, 125.9, 125.9, 125.5, 123.4, 98.8, 56.1.

^{19}F NMR (471 MHz, $CDCl_3$) δ –62.3.

R_f = 0.46 (Ethyl acetate/hexanes = 1:10).

(*S*)-3,3'-bis(4'-(trifluoromethyl)-[1,1'-biphenyl]-3-yl)-[1,1'-binaphthalene]-2,2'-diol

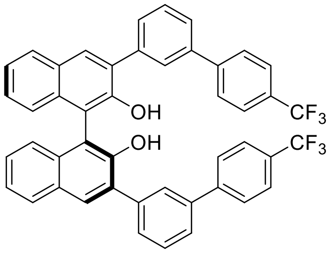

^{1}H NMR (501 MHz, $CDCl_3$) δ 8.12 (s, 2H), 8.00 (t, *J* = 1.8 Hz, 2H), 7.97 (dd, *J* = 8.2, 1.2 Hz, 2H), 7.83–7.75 (m, 6H), 7.72 (d, *J* = 8.3 Hz, 4H), 7.66 (dt, *J* = 7.8, 1.6 Hz, 2H), 7.61 (t, *J* = 7.6 Hz, 2H), 7.44 (ddd, *J* = 8.1, 6.8, 1.3 Hz, 2H), 7.37 (ddd, *J* = 8.3, 6.8, 1.3 Hz, 2H), 7.28 (dd, *J* = 8.5, 1.2 Hz, 2H), 5.41 (s, 2H).

^{13}C NMR (126 MHz, CD_2Cl_2) δ 151.0, 145.3, 140.6, 139.2, 133.9, 132.2, 132.2, 131.1, 130.3, 130.1, 130.1, 129.7, 129.7, 129.3, 129.3, 129.2, 129.2, 128.2, 128.2, 128.1, 128.1, 127.2, 127.2, 126.4, 126.3, 126.3, 126.3, 125.1, 125.1, 124.8, 124.8, 113.4. (due to the low peak intensity, the splitting of carbon NMR is hard to distinguish)

^{19}F NMR (471 MHz, CD_2Cl_2) δ –62.5.

R_f = 0.26 (Ethyl acetate/hexanes = 1:9).

ESI-HRMS (*m/z*): calculated for $C_{46}H_{27}F_6O_2$ ($[M-H]^-$): 725.1921, found: 725.1933.

m.p. = 220–225°C.

$[\alpha]_D^{25}$ = –28.8 (*c* 0.64, $CHCl_3$).

In a flame dried 2-neck round-bottom flask with a condenser, (*S*)-2,2'-(2,2'-bis(methoxymethoxy)-1,1'binaphthyl-3,3'-diyl)bis(4,4,-5,5-tetramethyl-1,3,2-dioxaborolane) (688 mg, 1.1 mmol, 1.0 equiv.), 3-(3-bromophenyl)thiophene (605 mg, 2.3 equiv.), and tetrakis(triphenylphosphine)palladium (126 mg, 0.11 mmol, 0.1 equiv.) were dissolved in 1,4-dioxane (14 mL). After degassing the reaction mixture with argon for 10–15 min, a degassed aqueous solution of K_2CO_3 (2.0 M, 4.1 mL) was added. The resultant mixture was then transferred into a microwave reactor and stirred at 90 °C for 2 h. After cooling the reaction to room temperature, the mixture was further purified by column chromatography to afford (*S*)-3,3'-((2,2'-bis(methoxymethoxy)-[1,1'-binaphthalene]-3,3'-diyl)bis(3,1-phenylene))dithiophene. A solution of this material and Amberlyst®-15 (Hydrogen form) in THF/MeOH (1:1) was then stirred at 90 °C for 12 h. After the completion of the reaction (as monitored by TLC analysis), Amberlyst®-15 was removed by filtration, and the mixture

was concentrated under reduced pressure. Finally, the crude mixture was purified by column chromatography (Ethyl acetate/hexanes 1:20 to 1:4) to afford (*S*)-3,3'-bis(3-(thiophen-3-yl)phenyl)-[1,1'-binaphthalene]-2,2'-diol as a white solid (overall yield of the two steps: 72 %).

(*S*)-3,3'-((2,2'-bis(methoxymethoxy)-[1,1'-binaphthalene]-3,3'-diyl)bis(3,1-phenylene))dithiophene

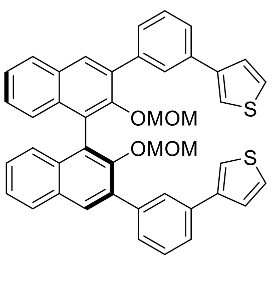

^{1}H NMR (501 MHz, $CDCl_3$) δ 8.08–8.02 (m, 4H), 7.97–7.91 (m, 2H), 7.75 (dt, *J* = 7.6, 1.4 Hz, 2H), 7.66 (ddd, *J* = 7.7, 1.9, 1.2 Hz, 2H), 7.58 (dd, *J* = 2.9, 1.4 Hz, 2H), 7.56–7.50 (m, 4H), 7.49–7.41 (m, 4H), 7.40–7.30 (m, 4H), 4.52 (d, *J* = 5.8 Hz, 2H), 4.49 (d, *J* = 5.8 Hz, 2H), 2.45 (s, 6H).

^{13}C NMR (126 MHz, $CDCl_3$) δ 151.5, 142.3, 139.8, 136.1, 135.5, 133.8, 131.0, 130.7, 128.9, 128.7, 128.0, 127.8, 126.7, 126.6, 126.5, 126.4, 125.5, 125.4, 120.7, 98.7, 56.1.

R_f = 0.32 (Ethyl acetate/hexanes = 1:10).

(*S*)-3,3'-bis(3-(thiophen-3-yl)phenyl)-[1,1'-binaphthalene]-2,2'-diol

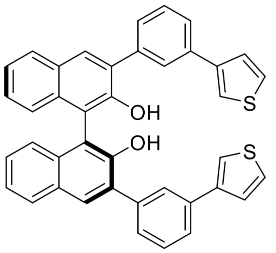

^{1}H NMR (501 MHz, $CDCl_3$) δ 8.08 (s, 2H), 7.97 (t, *J* = 1.8 Hz, 2H), 7.96–7.92 (m, 2H), 7.66 (ddt, *J* = 13.1, 7.7, 1.4 Hz, 4H), 7.56–7.49 (m, 4H), 7.46 (dd, *J* = 5.0, 1.4 Hz, 2H), 7.44–7.38 (m, 4H), 7.35 (ddd, *J* = 8.3, 6.8, 1.3 Hz, 2H), 7.29–7.23 (m, 2H), 5.40 (s, 2H).

^{13}C NMR (126 MHz, $CDCl_3$) δ 150.3, 142.3, 138.1, 136.4, 133.2, 131.6, 130.7, 129.6, 129.1, 128.6, 128.5, 128.0, 127.6, 126.6, 126.4, 126.0, 124.6, 124.5, 120.8, 112.6.

R_f = 0.22 (Ethyl acetate/hexanes = 1:9).

ESI-HRMS (*m*/*z*): calculated for $C_{40}H_{25}O_2S_2$ ([M-H]$^-$): 601.1302, found: 601.1309.

m.p. = 286–288°C.

$[\alpha]_D^{25}$ = –35.3 (*c* 0.34, $CHCl_3$).

The synthesis of phosphorimidoyl trichlorides R_f-**1–3.**

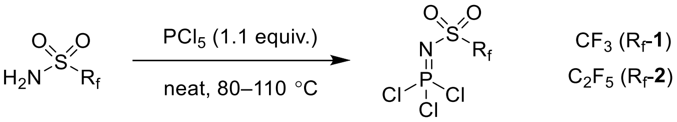

The syntheses of ((trifluormethyl)sulfonyl)phosphorimidoyl trichloride[23] R_f-**1** and ((perfluoroethyl)sulfonyl)phosphorimidoyl trichloride[24] R_f-**2** have been described previously.

General procedure for the preparation of compounds **3**, **4** and **5**. To a mixture of the 3,3'-disubstituted BINOL (2.1 equiv.) and ((trifluormethyl)sulfonyl)phosphorimidoyl trichloride (2.1 equiv.) in toluene (0.1 M) was added diisopropylethylamine (16 equiv.) at room temperature under an argon atmosphere. After stirring for 10–30 min, HMDS (1.0 equiv.) was added. After an additional 10 min at room temperature, the reaction mixture was sealed and heated to 120–130 °C for 2–3 d. The mixture was diluted with ethyl acetate and insoluble solid was filtered off through a short pad of Celite. Then the solvent was removed under reduced pressure and the crude residue was purified by column chromatography on silica gel using ethyl acetate/hexanes mixtures. After evaporation of solvent, the collected solid was stirred in a biphasic solution (DCM/6 M HCl) for 15 min and extracted with DCM. Azeotropic removal of water using toluene gave catalysts **3** (yield: 60%), **4** (yield: 48%) and **5** (yield: 82%) as white solid in their acidic form.

*Note: catalysts **2**[4] was previously reported, and the 3,3'-disubstituted BINOL for **4** is known.[25]*

(*S,S*)-IDPi-**3**:

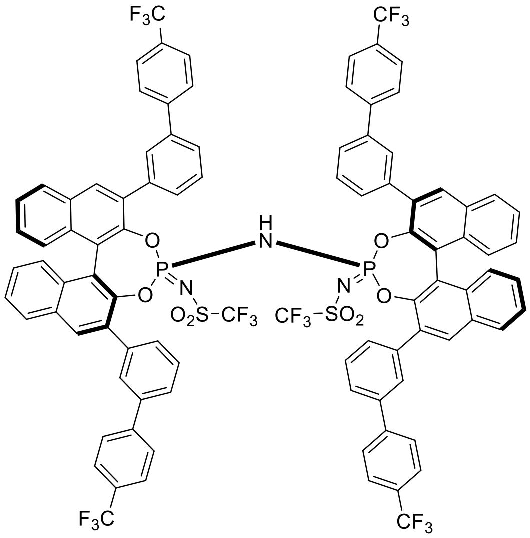

^{1}H NMR (501 MHz, CD_2Cl_2) δ 8.17 (s, 2H), 8.08–7.98 (m, 4H), 7.89 (ddd, *J* = 8.1, 6.7, 1.1 Hz, 2H), 7.79–7.73 (m, 6H), 7.71–7.63 (m, 6H), 7.60 (ddd, *J* = 8.1, 6.7, 1.2 Hz, 2H), 7.52 (s, 12H), 7.42–7.34 (m, 4H), 7.31 (dt, *J* = 5.0, 2.3 Hz, 4H), 7.21–7.14 (m, 4H), 7.09 (t, *J* = 7.7 Hz, 2H), 7.04–6.98 (m, 2H), 6.53 (d, *J* = 7.8 Hz, 2H), 5.71 (s, 1H).

^{13}C NMR (126 MHz, CD_2Cl_2) δ 145.1, 144.3, 143.8 (t, *J* = 5.2 Hz), 143.0 (t, *J* = 5.2 Hz), 141.0, 140.1, 136.7, 136.6, 133.3, 133.0, 132.5, 132.2, 132.1, 131.7, 129.9, 129.8, 129.8, 129.7, 129.6, 129.6, 129.5, 129.4, 129.3, 129.2, 128.9, 128.7, 128.5, 128.2, 128.1, 127.6, 127.5, 127.4, 127.3, 127.2, 127.1, 126.0, 126.0, 126.0, 125.9, 125.7, 125.7, 125.7, 125.7, 123.8, 123.7, 123.6, 122.1, 120.6, 118.0.

^{19}F NMR (471 MHz, CD_2Cl_2) δ –62.7, –62.8, –78.7.

^{31}P NMR (203 MHz, CD_2Cl_2) δ –16.8.

R_f = 0.24 (Ethyl acetate/hexanes = 1:4).

ESI-HRMS (*m/z*): calculated for $C_{94}H_{52}F_{18}N_3O_8P_2S_2$ ($[M-H]^-$): 1818.2389, found: 1818.2409.

m.p. = 252–276 °C.

$[\boldsymbol{\alpha}]_{\mathbf{D}}^{\mathbf{25}}$ = 225.0 (*c* 0.20, $CHCl_3$).

(*S,S*)-IDPi-**4**:

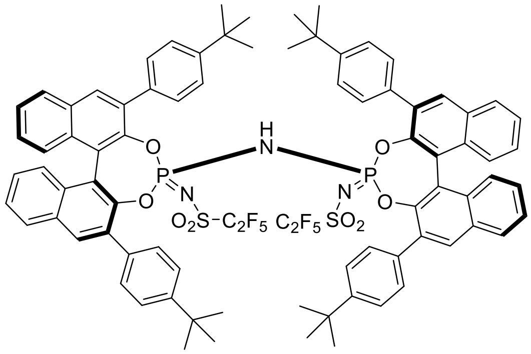

^{1}H NMR (501 MHz, CD_2Cl_2) δ 8.06 (s, 2H), 7.94 (dd, *J* = 9.9, 8.5 Hz, 4H), 7.72 (ddd, *J* = 8.2, 5.6, 2.3 Hz, 2H), 7.57–7.50 (m, 4H), 7.46 (ddd, *J* = 8.1, 5.7, 2.1 Hz, 2H), 7.28–7.19 (m, 8H), 7.16 (d, *J* = 8.2 Hz, 4H), 7.04–6.93 (m, 6H), 6.58–6.51 (m, 4H), 5.75 (s, 1H), 1.19 (s, 18H), 1.02 (s, 18H).

^{13}C NMR (126 MHz, CD_2Cl_2) δ 151.5, 151.3, 144.3, 144.2, 144.2, 143.3, 133.5, 133.4, 133.1, 132.8, 132.6, 132.3, 132.3, 132.0, 132.0, 131.6, 130.0, 129.5, 129.4, 129.1, 127.6, 127.5, 127.3, 127.1, 127.0, 126.8, 125.8, 125.0, 123.6, 122.4, 34.8, 34.7, 31.3, 31.2.

^{19}F NMR (471 MHz, CD_2Cl_2) δ –79.0, –116.2.

^{31}P NMR (203 MHz, CD_2Cl_2) δ –17.0.

R_f = 0.32 (Ethyl acetate/hexanes = 1:4).

ESI-HRMS (*m/z*): calculated for $C_{84}H_{72}N_3O_8F_{10}P_2S_2$ ($[M-H]^-$): 1566.4082, found: 1566.4095.

m.p. = 208–225 °C.

$[\boldsymbol{\alpha}]_{\mathbf{D}}^{\mathbf{25}}$ = 289.2 (*c* 0.26, $CHCl_3$).

(*S,S*)-IDPi-**5**:

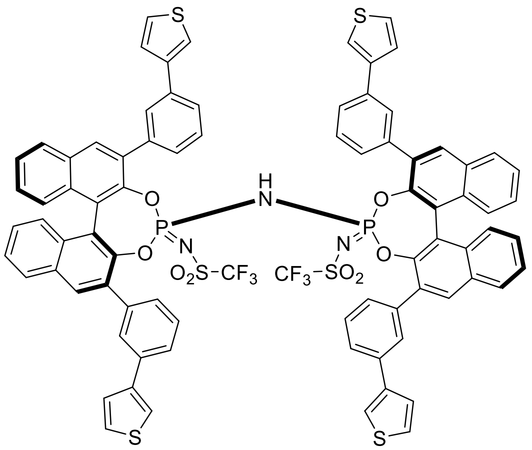

^{1}H NMR (501 MHz, CD_2Cl_2) δ 8.17 (s, 2H), 8.08 (dd, J = 10.3, 8.2 Hz, 4H), 7.85 (dd, J = 8.0, 6.2 Hz, 2H), 7.75 (d, J = 2.0 Hz, 2H), 7.70–7.57 (m, 6H), 7.56–7.46 (m, 6H), 7.46–7.24 (m, 18H), 6.95 (d, J = 7.8 Hz, 2H), 6.85 (q, J = 7.9 Hz, 4H), 6.04 (s, 1H), 5.89 (d, J = 7.7 Hz, 2H).

^{13}C NMR (126 MHz, CD_2Cl_2) δ 143.8, 143.2, 142.6, 141.8, 136.9, 136.5, 136.3, 135.9, 133.9, 133.5, 132.5, 132.4, 132.3, 132.3, 132.1, 131.6, 129.4, 129.1, 129.1, 128.7, 128.6, 128.5, 128.3, 128.1, 127.8, 127.5, 127.4, 127.4, 127.2, 127.0, 127.0, 126.7, 126.4, 126.4, 123.7, 122.3, 121.2, 121.1, 117.9.

^{19}F NMR (471 MHz, CD_2Cl_2) δ –78.7.

^{31}P NMR (203 MHz, CD_2Cl_2) δ –16.7.

R_f = 0.19 (Ethyl acetate/hexanes = 1:4).

ESI-HRMS (*m/z*): calculated for $C_{82}H_{48}F_6N_3O_8P_2S_6$ ($[M-H]^-$): 1570.1151, found: 1570.1140.

m.p. = 220–229°C.

$[\alpha]_D^{25}$ = 231.2 (*c* 0.16, $CHCl_3$).

10. Computational studies

Computational methods

To identify the thermally accessible intermediates and transition states (TSs) among the thousands of plausible structures for IDPi catalyst system, we applied a filtration strategy which is similar to the protocol reported in a recent study on IDPi.[26] Candidates structures were generated from extensive conformational sampling and filtered by a hierarchy of levels of theory, as detailed in the following sections. All calculations were conducted with Gaussian[27] software unless otherwise noted.

Step 1. Generation of initial guess structures

We first generate a set of initial guess structures of catalysts, reaction intermediates and TSs for the subsequent MD simulations. The initial guess structures of the chiral catalyst HX (i.e IDPi) were built based on the structures reported in the literature[26] or taken from the X-ray crystal structures. The initial guess structures of silylated catalysts **INT1** and **INT1’** were generated by placing the TMS cation and HNC or HCN to different N or O atoms of the anion catalysts in different relative orientations. The initial guess structures of ion pairs **INT2** and **INT2’** were produced by placing the anion catalysts and silylated substrate (in their different conformations), respectively, in different relative orientations. The initial guess structures of TSs are constructed based on the optimized structures of intermediates.

Step 2. Conformational sampling

Staring from the initial guess structures obtained in the first step, molecular dynamics (MD) simulations with xTB 6.3[28] employing GFN0-xTB method were performed to sample the conformational space of catalysts, intermediates, and TSs. Restrains were added to keep the HCN (or HNC) and substrate to be attached with catalyst for MD of silylated catalysts and ion pairs. For the MD simulations of TSs, the chemical bonds that are about to form or break were constrained to specific values. The redundant conformations for which their RMSD is less than 0.2 Å and energy difference is less than 0.2 kcal/mol were removed. The remained conformers were submitted

for further optimizations (restrain optimization for TSs) at the GFN2-xTB[29] level and then filtered based on the RMSD and energy difference criteria.

According to a previous study on IDPi,[26] the lowest-energy conformer of IDPi catalyst keeps a C2 symmetry. Thus, we also applied Molclus[30] software to systematically generate a variety of conformations in C2 symmetry as complementary to the MD simulation method.

Step 3. Geometry optimizations and frequency calculations

Candidate structures that obtained from the second step from the two sampling methods were further optimized (with restrains for TSs) using the PBE[31] functional including Grimme's D3 dispersion correction[32] in conjunction with a double-ζ basis set (def2-SVP)[33] and then filtrated based on the same criteria listed above. Among the structures left after redundancy elimination for catalysts and intermediates, we selected the conformers that are within 10.0 kcal/mol of energy difference with the lowest-energy conformer for further frequency calculations to obtain their thermal corrections. For TSs, the conformations that are within 8.0 kcal/mol of energy difference with the lowest-energy conformer and those with remarkably different structural features were submitted to fully relaxed TS optimization and frequency calculations at the PBE-D3/def2-SVP level.

Step 4. Single-point energies in solvent

For low-energy structures identified in the previous step, single point energy was computed at the B3LYP-D3/def2-TZVP level and solvent effects were introduced with the SMD model.[34]

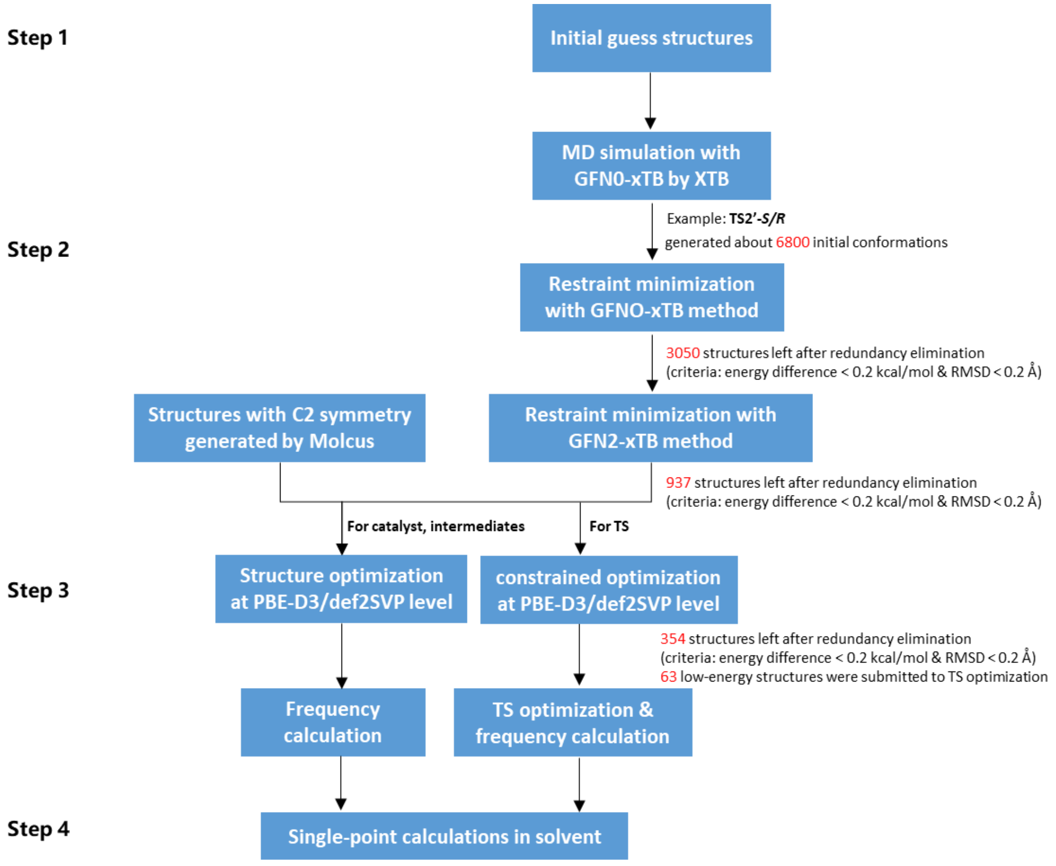

Figure S21. Work flow of conformational sampling and filtration.

Computational results

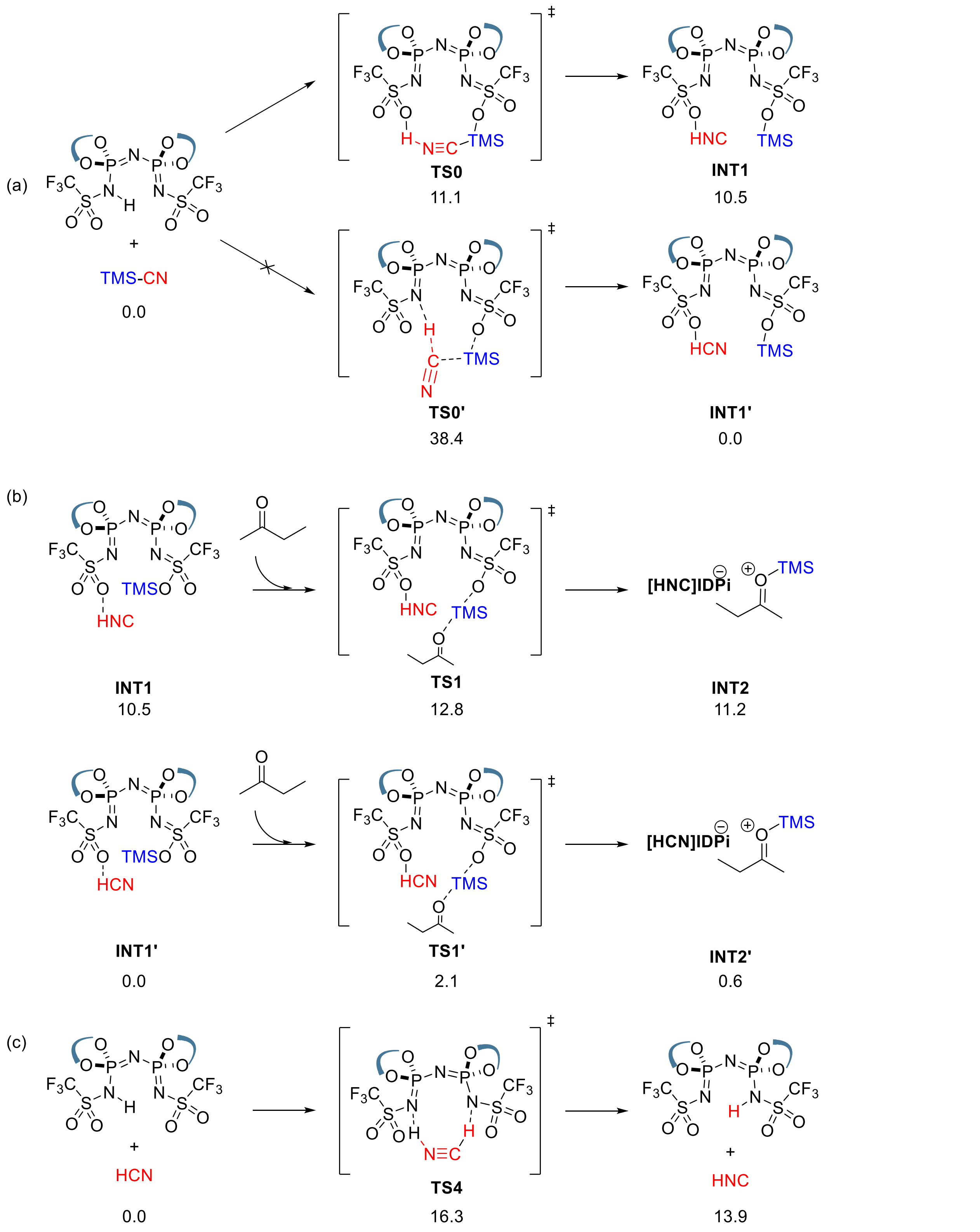

Figure S22. (a) Activation of TMSCN **(b)** transfer of TMS group to the substrate and **(c)** IDPi-catalyzed interconversion of HNC and HCN. Gibbs free energies are in kcal•mol^{-1}.

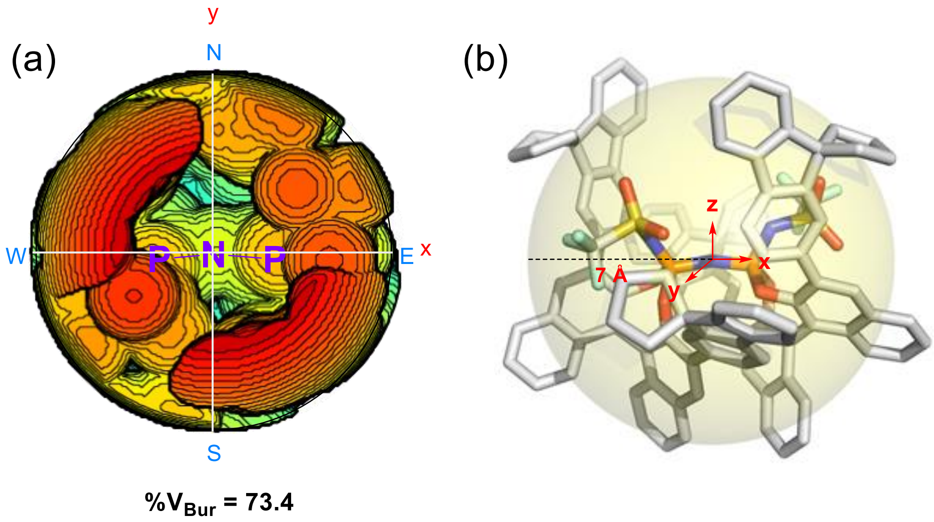

(c)

%V Free	%V Buried	% V Tot/V Ex
26.6	**73.4**	100.0

Quadrant	V f	V b	V t	%V f	%V b
SW	90.9	268.2	359.1	**25.3**	74.7
NW	90.6	268.5	359.1	**25.2**	74.8
NE	134.0	225.0	359.1	37.3	**62.7**
SE	66.8	292.3	359.1	**18.6**	81.4

Figure S23. **a**, steric map of IDPi **2** based on the density functional theory (DFT)-optimized structure of **TS2-*S***. The red and blue zones indicate the more- and less-hindered zones in the catalytic pocket, respectively. **b**, the steric map was calculated using a 7Å sphere around the N atom center to characterize the feature of the whole catalyst. The map is viewed down the z-axis; the orientation of IDPi is indicated in **b**. **c**, the calculated percentage of buried volume and free volume.[35,36]

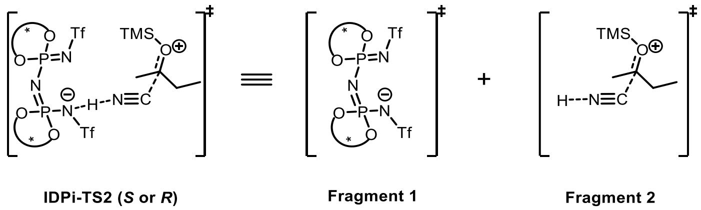

	$\Delta\Delta E_{distortion}$			$\Delta\Delta E_{interaction}$	$\Delta\Delta E$
	**Total**	**Fragment 1**	**Fragment 2**		
**TS2-*S* – TS2-*R***	–3.5	–1.6	–1.9<br>(–1.6 for the [TMS-2-butanone]$^+$ part)	–0.2	–3.7

Figure S24. Distortion/interaction analysis were performed at the level of B3LYP-D3/def2-TZVP/SMD (solvent = diethylether) //PBE-D3/def2-SVP. Electronic energies are in kcal•mol^{-1}. The origin of enantioselectivity was mainly attributed to the distortion energy difference, especially the distortion energy of fragment 2. The distortion energy of fragment 2 for **TS2-*R*** is 1.9 kcal•mol^{-1} higher than that for **TS2-*S*** since the TMS group is tilted from the carbonyl plane (α(Me-C-O-Si) = –40°). The corresponding distortion energy of the [TMS-2-butanone]$^+$ part in fragment 2 for **TS2-*R*** is 1.6 kcal•mol^{-1} higher than that for **TS2-*S***.

To understand the stereocontrol of different IDPi catalysts, IDPi **2**, **5**, and **4**-catalyzed cyanosilylation of substrates 2-butanone and acetophenone were investigated by DFT calculations. Results are shown below:

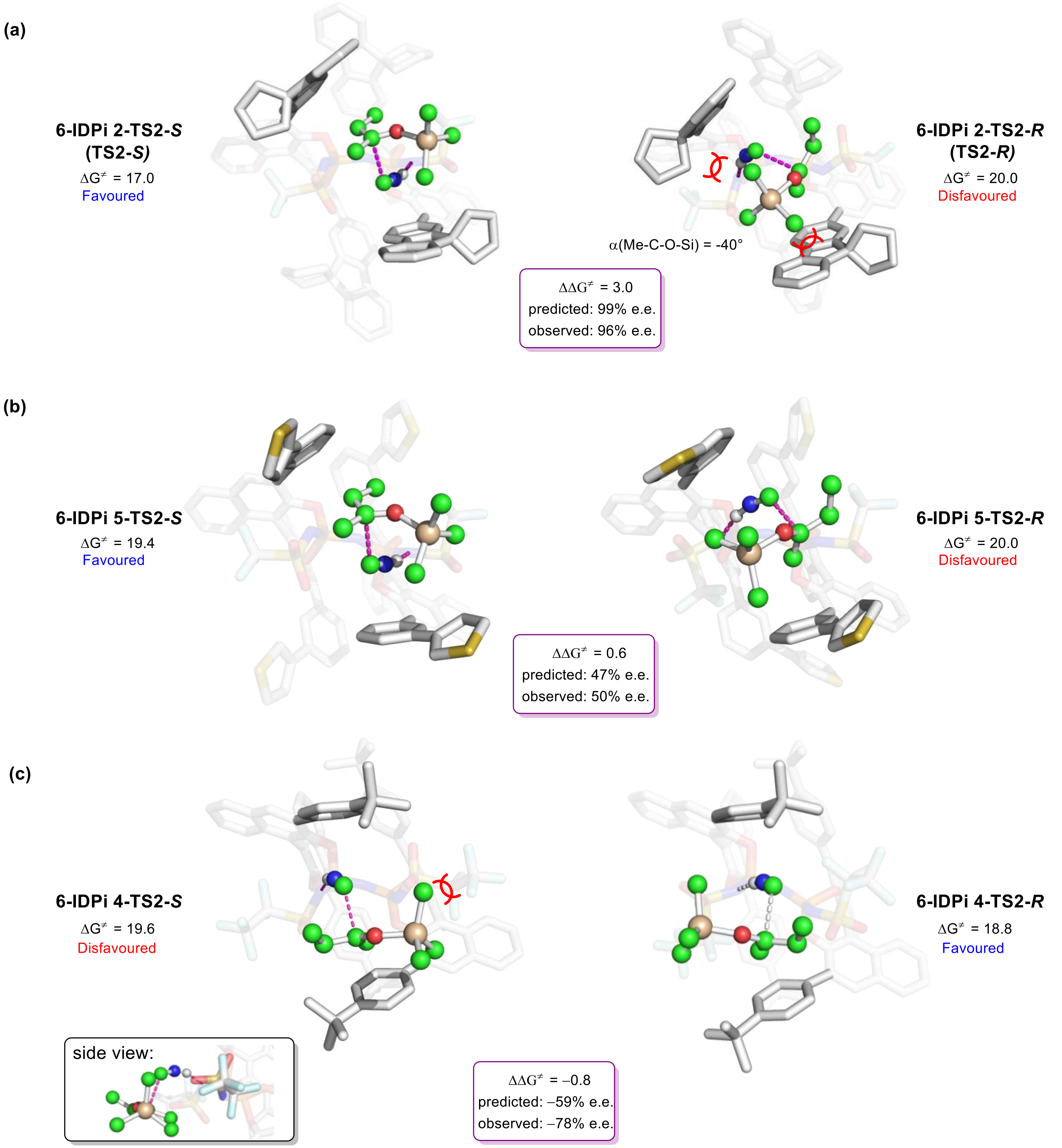

Figure S25. Optimized transition state structures **TS2-*S*** and **TS2-*R*** for IDPi **2**, **5**, and **4**-catalyzed cyanosilylation of 2-butanone. Their activation free energies are in kcal•mol^{-1}. (a) **6**-IDPi **2**-TS-*R* orientates the bulky TMS group in a narrow pocket formed by Ar substituents of catalyst (the crowed south-western quadrant as shown in **Figure S23**). To relieve the steric repulsion with the Ar substituents, the silylated substrate is distorted (α(Me-C-O-Si) = –40°) which leads to a barrier higher than **6**-IDPI **2**-TS2-*S*; (b) In the

case of IDPi **5**, the pocket is too large to differentiate the methyl and ethyl groups and thus the enantioselectivity is poor; (c) In **6**-IDPi **4**-TS2-*S*, the bulky TMS group is in proximity of the outward-facing –C_2F_5 group of the catalyst, which leads to steric repulsion. This unfavorable interaction is avoided in **6**-IDPi **4**-TS2-*R*. Therefore, the *R*-product is favored.

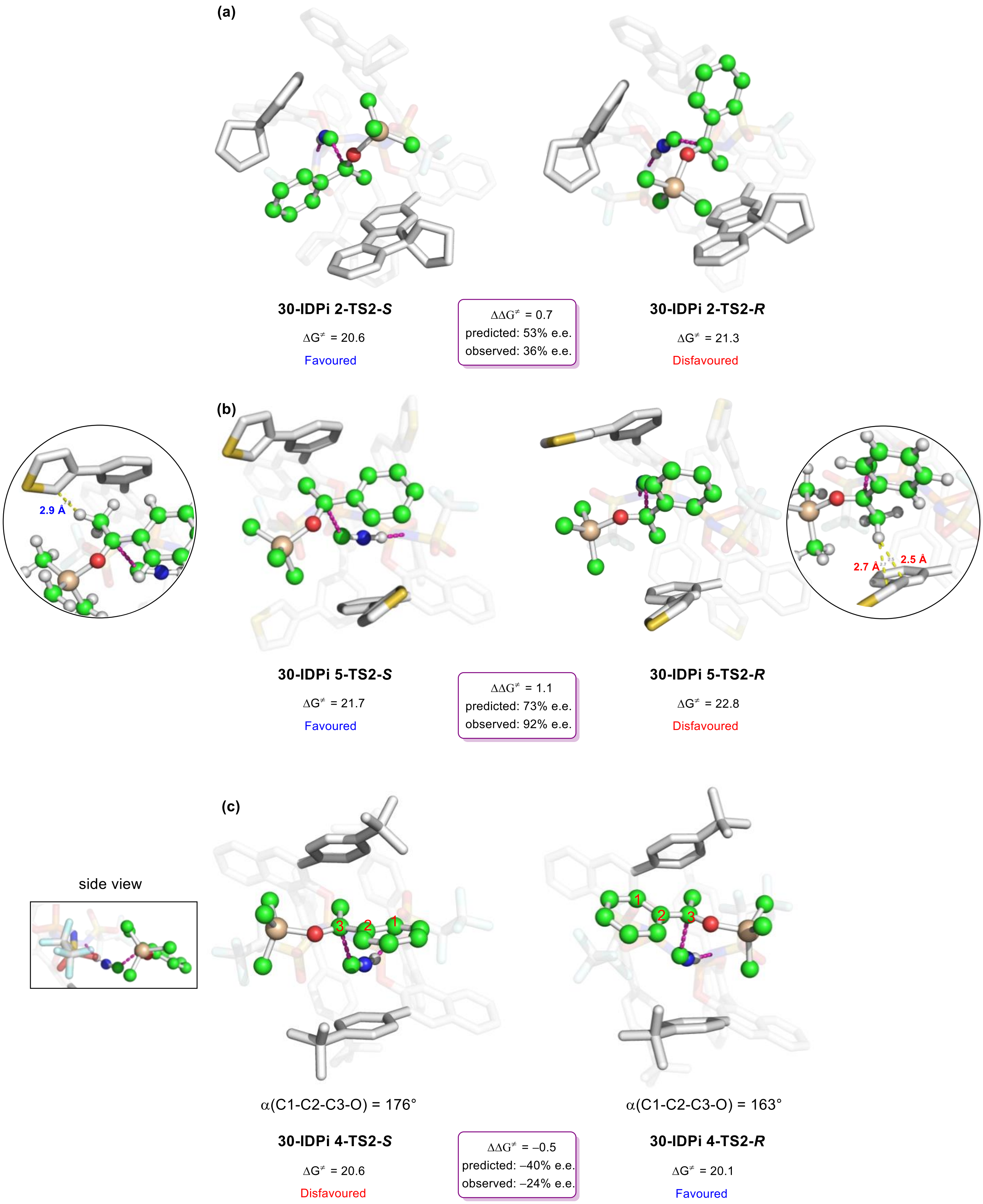

Figure S26. Optimized transition state structures **TS2-*S*** and **TS2-*R*** for IDPi **2, 5,** and **4**-catalyzed cyanosilylation of acetophenone substrate. Their activation free energies are in kcal•mol^{-1}. For acetophenone substrate that possesses two large groups (i.e. phenyl group and TMS group), only the small methyl group can bind the inner pocket formed by the N−P−N−P−N core moiety in all TSs. (a) **30-IDPi 2-TS2-*R*** orientates the bulky TMS group in a narrow pocket of catalyst which leads to a higher barrier. But the e.e. decreases compared with the IDPi **2**-catalyzed reaction of 2-butanone because acetophenone stays at the outer region of the pocket that is farther away from the N1 atom center of the catalyst. In addition, the sterical difference between the phenyl and TMS group becomes smaller compare to that between the ethyl and TMS group; (b) In the case of IDPi **5**, the wide pocket is suitable to accommodate the phenyl-substituted substrates. Acetophenone can bind closer to N1 center which leads to excellent enantioselectivity. The unfavorable TS (**30-IDPi 5-TS2-*R***) suffers from steric hindrance between the methyl group and the Ar substituent (short C H distances of 2.5 and 2.7 Å are detected); (c) In **30-IDPi 4-TS2-*S***, the bulky TMS moiety is close to the outward-facing –C_2F_5 group of the catalyst. While in **30-IDPi 4-TS2-*R***, the planarity of phenyl group and the acyl group is slightly distorted (α(C-C-C-O) = 163°) to avoid repulsions with the outward-facing –C_2F_5 group. Therefore, the *R*-product is slightly favored.

The substrate binding pocket is constituted by the N2−P−N1−P−N3 core moiety, R^1 and R^2 groups. As shown in **Figure S27**, the N2−P−N1−P−N3 core moiety constitutes the bottom of the pocket and determines the size of its inner region. The R^1 group (Ar substituents) and the R^2 (fluoroalkyl sulfonate substituents) set a long wall and a short wall along z axis, respectively, which determines the outer region open for the substrate approaching. The three structural moieties jointly determine the feature of IDPi's substrate binding pocket. Thus, the shape of the binding pocket can be tuned by altering the R^1 and R^2 substituents to achieve good chemo- and enantioselectivities for different substrates.

To understand how the R^1 and R^2 substituents modulate the binding pocket to recognize different substrates, we analyzed the geometry features and steric map of IDPi **2**, **4**, and **5** (**Figure S28**). These three catalysts are installed with different R^1 and (or) R^2 substituents and thus serve as ideal model catalysts for the study. In order to characterize the steric bias and %V buried (the percentage of buried volume) of the region open for the substrate access and binding, we defined an origin point that is 4Å away from the N1 atom and calculated the steric maps using a 5Å sphere around that point.

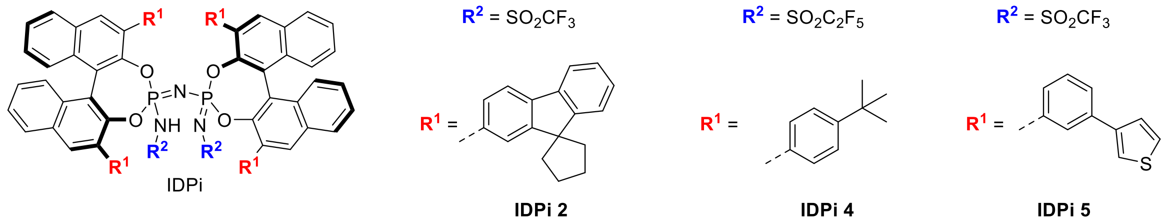

For IDPi **2** and IDPi **5**, the OTf-N2-P-N1-P-N3-OTf core moiety sets a broad bottom with a relatively large inner region as indicated by a large N2-N3 distance (4.7 and 4.8 Å, **Figure S27**). The R^1 substituents of IDPi **2** forms an asymmetric pocket which features with a narrow end (distance between two Ar groups is 7.4 Å) and a wide end (distance between two Ar groups is 10.4 Å). While the R^1 substituents are near parallel to each other in IDPi **5**. Moreover, the steric demanding R^1 substituents of IDPi **2** leads to a more confined pocket with 46.6% buried volume and the R^1 groups shield the north-western and south-eastern quadrants, leading to biased steric. These features enable IDPi **2** to differentiate the methyl and ethyl groups well. By contract, IDPi **5** features a relatively larger pocket (42.9% V buried) and the shielding from the R^1 groups are more distant from the center. Thus, IDPi **5** cannot differentiate the methyl and ethyl groups well, but its large pocket is suitable to accommodate the relatively large aromatic moiety, e.g. acetophenone substrate.

For IDPi **4**, the $SO_2C_2F_5$-N2-P-N1-P-N3-$SO_2C_2F_5$ core moiety sets a narrow bottom with a relatively small inner region as indicated by a small N2-N3 distance (3.7 Å, **Figure S27**). N2 and N3 bring the large $SO_2C_2F_5$ groups to be closer than that in IDPi **2** and **5**, which leads to a narrow pocket with 49.4% buried volume. With such a narrow pocket, IDPi **4** is not suitable to accommodate aromatic ketones, e.g. acetophenone, but it can be used for aryl-substituted aliphatic ketones as the alkyl link introduces conformational flexibility and directs the aryl group farther away from the $SO_2C_2F_5$ group.

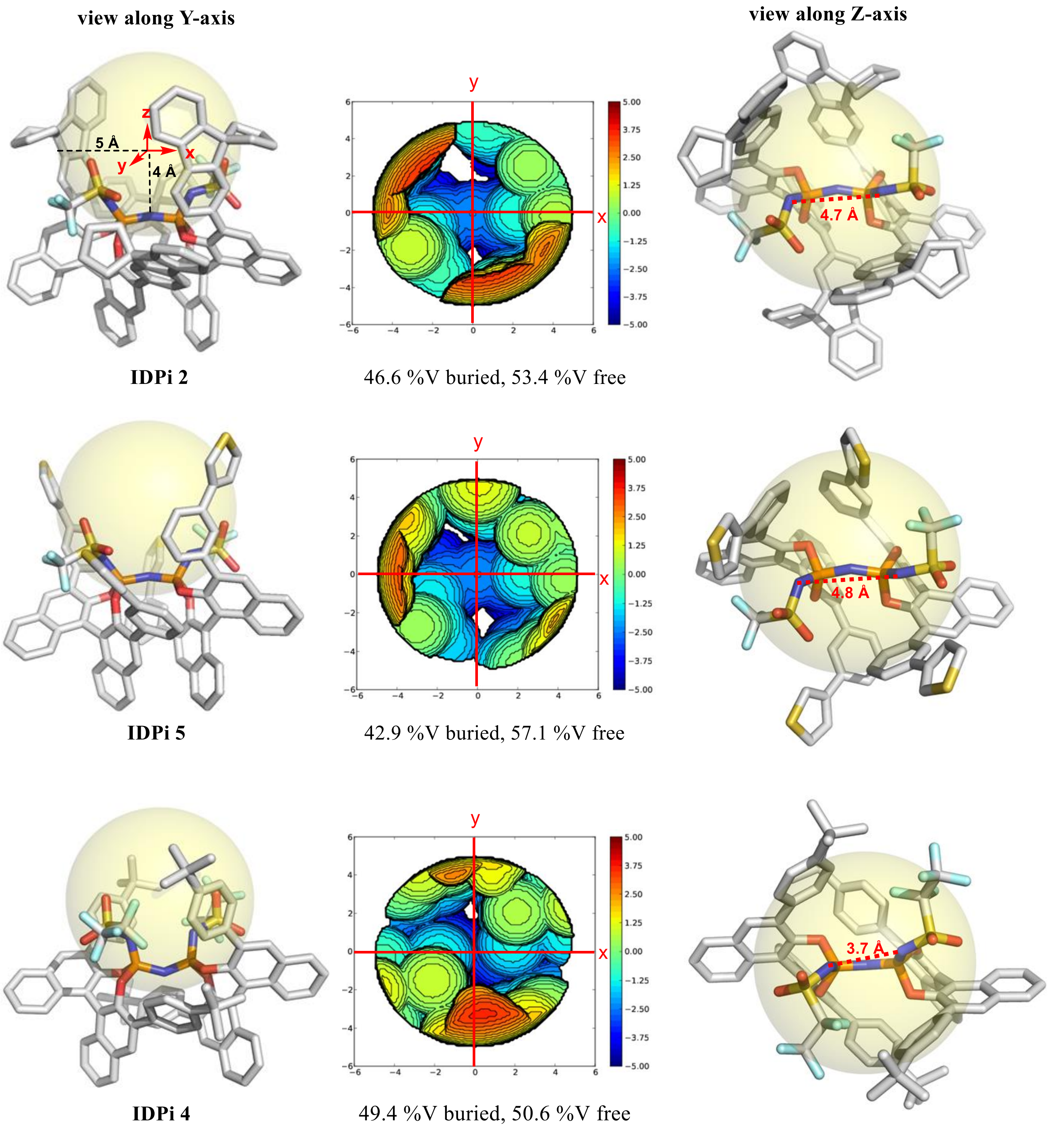

Figure S27. Steric maps of IDPi **2**, IDPi **4**, and IDPi **5** based on the density functional theory (DFT)-optimized lowest-energy TSs. The steric maps are viewed down the z-axis; the orientation of IDPi is indicated in the left panel. The red and blue zones indicate the more- and less-hindered zones in the catalytic pocket, respectively. Note the steric maps were calculated using a 5Å sphere around a point that 4Å away from the N1 atom, which is different from that used in **Figure S23** and **Figure 4b**. We applied a different sphere because the focus here is to analyze the catalytic pocket around the reacting HNC and substrate instead of capturing the feature of the whole catalyst.

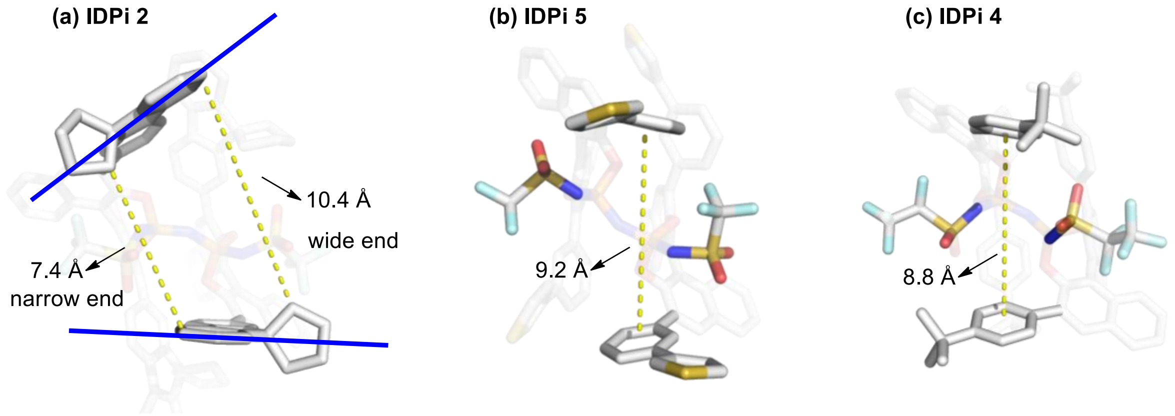

Figure S28. Geometry features of (a) IDPi **2**, (b) **5** and (c) **4**.

Table S7. Energy table for optimized structures.

Listed are the zero-point energy (ZPE), enthalpy correction (H_{corr}), Gibbs free energy correction (G_{corr}) and imaginary frequencies (for transition states) of the optimized structures calculated in the gas-phase at 298.15 K and 1 atm. The single-point energies (E_{sol}) calculated in the diethylether solvent are also presented. All energy data are given in Hartree.

Structures	ZPE	Hcorr	Gcorr	Esol	Imaginary frequency
2-Butanone	0.108704	0.115513	0.079753	-232.5817484	//
TMS-CN	0.115808	0.12644	0.082153	-502.2554904	//
HCN	0.015896	0.019402	-0.003505	-93.46770113	//
HNC	0.014436	0.018585	-0.003439	-93.44552205	//
IDPi **2**	1.611482	1.718813	1.466909	-7087.498243	//
INT1	1.728878	1.847874	1.572411	-7589.760314	//
INT1'	1.729022	1.848047	1.572605	-7589.777367	//
INT2	1.839949	1.966495	1.671159	-7822.359923	//
INT2'	1.838192	1.965133	1.668981	-7822.374715	//
TS0	1.727500	1.845703	1.568478	-7589.755500	-91.50
TS0’	1.725007	1.568214	1.568214	-7589.711752	-726.34
TS1	1.83962	1.965404	1.672656	-7822.35892	-18.34
TS1’	1.839545	1.965491	1.672217	-7822.375543	-15.41
TS2-*S*	1.838003	1.963767	1.672781	-7822.352431	-65.73

TS2-*R*	1.83769	1.963419	1.671753	-7822.346521	-54.21
TS2'-*S*	1.837376	1.962918	1.671686	-7822.330585	-416.79
TS2'-*R*	1.836905	1.962518	1.671181	-7822.327632	-391.77
TS3	1.833908	1.960356	1.665842	-7822.350968	-704.97
TS4	1.622930	1.733066	1.473022	-7180.949655	-584.71
**53***Z*	0.2074	0.222731	0.1665	-641.3621983	//
**6**	0.229221	0.246369	0.18674	-734.8644512	//
1_Ph	0.134317	0.143192	0.101643	-385.0657904	//
IDPi **4**	1.370375	1.470131	1.235637	-6491.016315	//
IDPi **5**	1.09257	1.183142	0.961307	-7593.566708	//
**6**-IDPi **4**-TS2-*S*	1.596298	1.714631	1.439424	-7225.864281	-90.31
**6**-IDPi **4**-TS2-*R*	1.596065	1.714458	1.441063	-7225.867167	-40.69
**30**-IDPi **4**-TS2-*S*	1.621299	1.741242	1.460917	-7378.346264	-92.74
**30**-IDPi **4**-TS2-*R*	1.621351	1.741301	1.463184	-7378.349265	-49.70
**6**-IDPi **5**-TS2-*S*	1.319286	1.427655	1.17057	-8328.42018	-80.78
**6**-IDPi **5**-TS2-*R*	1.320082	1.428055	1.172117	-8328.420933	-109.70
**30**-IDPi **5**-TS2-*S*	1.344857	1.454863	1.193614	-8480.901866	-27.64
**30**-IDPi **5**-TS2-*R*	1.344568	1.454278	1.193493	-8480.900099	-145.42
**30**-IDPi **2** TS2-*S*	1.863335	1.990487	1.695841	-7974.831807	-74.68
**30**-IDPi **2**-TS2-*R*	1.86359	1.99082	1.696543	-7974.831489	-16.13

Table S8. Cartesian coordinates (in Å) of related structure.

2-Butanone

O	0.405228	1.384472	-0.000485
C	0.528278	0.169609	-0.000567
C	-0.684019	-0.760462	-0.001249
H	-0.587652	-1.439466	0.876808
H	-0.588874	-1.435894	-0.882221
C	-2.019138	-0.024599	0.001036
H	-2.109605	0.632039	-0.886509
H	-2.869530	-0.734382	0.000161
H	-2.108584	0.628436	0.891361
C	1.894441	-0.499358	0.000640
H	2.688280	0.269485	-0.004442
H	2.009386	-1.147853	0.894535
H	2.007383	-1.159287	-0.884971

TMS-CN

Si	0.311239	-0.000056	0.000044
C	0.859287	1.667996	0.686881
H	0.478331	2.501079	0.062686
H	0.489182	1.814388	1.721446
H	1.967286	1.728185	0.704413
C	0.859921	-0.238667	-1.787769
H	0.487043	0.582402	-2.432377
H	0.482356	-1.197371	-2.196575
H	1.967963	-0.249656	-1.848505
C	0.860719	-1.428550	1.100713
H	0.483591	-1.302761	2.135484
H	1.968751	-1.475607	1.140049
H	0.487656	-2.397474	0.712437
C	-1.571513	-0.000612	0.000195
N	-2.745713	-0.000487	0.000030

HCN

C	0.000000	0.000000	0.505970
N	0.000000	0.000000	-0.661300
H	0.000000	0.000000	1.593277

HNC

C	-0.002303	-0.743976	0.000000
N	-0.002303	0.433361	0.000000
H	0.029939	1.430324	0.000000

IDPi 2

P	-0.776561	-0.602649	1.286809
N	-0.134560	-0.839767	-0.154529
P	0.667707	-0.389038	-1.403779
N	0.564889	1.249981	-1.823659
S	-0.191823	2.018276	-3.132135
O	-0.564380	3.362457	-2.671743
O	-1.123848	1.114527	-3.820200
C	1.220065	2.241571	-4.391011
F	1.989481	3.280902	-4.041800
F	1.958832	1.131983	-4.458944
F	0.659157	2.485445	-5.574584
O	2.229018	-0.799296	-1.189813
C	3.078732	-0.874473	-2.303182
C	2.862298	-1.875168	-3.254813
C	3.676153	-1.863054	-4.444685
C	3.458033	-2.759171	-5.534266
C	4.255366	-2.712066	-6.668923
C	5.310734	-1.767244	-6.771790
C	5.536170	-0.868631	-5.740595
C	4.728495	-0.881402	-4.564240
C	4.936861	0.058719	-3.514991

C	4.128854	0.089462	-2.385214	C	-3.144328	-1.519579	2.104196
H	5.755211	0.789903	-3.603770	C	-2.832282	-2.797257	2.589250
H	6.341393	-0.121131	-5.815832	C	-3.617636	-3.328491	3.678427
H	5.939681	-1.742976	-7.674620	C	-3.264680	-4.523936	4.374750
H	4.063232	-3.409527	-7.498333	C	-4.052814	-5.008880	5.409199
H	2.637988	-3.487992	-5.472485	C	-5.234487	-4.324491	5.800230
C	1.799135	-2.901225	-3.041126	C	-5.590585	-3.146221	5.163463
C	0.477293	-2.500762	-2.835731	C	-4.793367	-2.613360	4.106048
C	-0.630420	-3.390533	-2.642829	C	-5.124260	-1.383502	3.477718
C	-0.313945	-4.751878	-2.690084	C	-4.314636	-0.796990	2.507303
C	1.014060	-5.235838	-2.823961	H	-6.031024	-0.849605	3.800652
C	1.300368	-6.634581	-2.764142	H	-6.491143	-2.592327	5.471700
C	2.605693	-7.098488	-2.813966	H	-5.854847	-4.722684	6.617478
C	3.682418	-6.178248	-2.928961	H	-3.754002	-5.928419	5.935140
C	3.437933	-4.814724	-3.017894	H	-2.347731	-5.058114	4.089969
C	2.105260	-4.306356	-2.982189	C	-1.725088	-3.605498	2.000478
H	4.277266	-4.110310	-3.102848	C	-0.435371	-3.078610	1.945021
H	4.718616	-6.548409	-2.945684	C	0.715754	-3.806693	1.502617
H	2.811872	-8.178015	-2.755497	C	0.488027	-5.083383	0.994301
H	0.458783	-7.337282	-2.660971	C	-0.813740	-5.651947	0.929828
H	-1.121043	-5.491168	-2.585746	C	-1.023339	-6.939285	0.348244
O	0.248242	-1.110856	-2.793322	C	-2.292997	-7.491254	0.270890
N	-0.584472	0.821032	2.011292	C	-3.406958	-6.772848	0.781948
S	0.788098	1.651845	1.798200	C	-3.235107	-5.524756	1.365054
O	2.025311	0.867357	1.998300	C	-1.943462	-4.925692	1.459474
O	0.741333	2.533862	0.582089	H	-4.107657	-4.974436	1.740887
C	0.648886	2.875817	3.255597	H	-4.416475	-7.205065	0.709315
F	1.230356	4.024685	2.907466	H	-2.440820	-8.481330	-0.186601
F	-0.631588	3.107345	3.569366	H	-0.149846	-7.480778	-0.047304
F	1.277427	2.354541	4.314885	H	1.342803	-5.682223	0.644846
O	-2.361898	-0.946687	1.097505	O	-0.262095	-1.749602	2.343972

C	3.671129	6.336313	3.196546	H	3.431124	7.257662	3.749427
C	4.192207	5.225189	3.886249	C	-8.317237	-0.017028	-1.781741
H	4.358050	5.286940	4.972874	C	-8.336596	-1.236760	-1.079778
C	4.496006	4.037710	3.200475	H	-9.272094	-1.585925	-0.616158
H	4.898460	3.165634	3.739382	C	-7.174018	-2.019886	-0.985484
C	4.267930	3.978754	1.815449	H	-7.191181	-2.984769	-0.455026
C	4.425994	2.876492	0.861241	C	-5.993554	-1.558376	-1.591806
C	4.842854	1.550295	1.049752	C	-4.674133	-2.183550	-1.717764
H	5.188483	1.191873	2.030034	C	-4.177261	-3.416564	-1.266556
C	4.759855	0.657154	-0.023778	H	-4.798791	-4.096919	-0.665418
H	5.026645	-0.396471	0.124748	C	-2.862883	-3.777080	-1.580609
C	4.305631	1.082490	-1.289690	H	-2.477426	-4.730004	-1.196190
C	3.954579	2.435915	-1.490730	C	-2.016710	-2.937224	-2.351402
H	3.606522	2.759927	-2.480111	C	-2.535447	-1.694979	-2.801278
C	3.998409	3.324834	-0.413747	H	-1.923599	-1.020235	-3.410574
C	3.749369	5.099618	1.117381	C	-3.839854	-1.323444	-2.473729
C	3.451144	6.279265	1.806737	C	-5.961084	-0.308653	-2.269202
H	3.040923	7.152774	1.274584	C	-7.130552	0.452615	-2.378315
C	3.591177	4.794620	-0.367887	H	-7.133845	1.406870	-2.926039
C	2.149579	5.056618	-0.943398	C	-4.552446	-0.013845	-2.779002
C	4.519969	5.697056	-1.261430	C	-4.489191	0.498217	-4.252248
C	2.293625	6.140264	-2.027081	C	-3.912166	1.163427	-1.966979
H	1.435380	5.329194	-0.145019	C	-4.329405	2.044378	-4.191429
H	1.759544	4.124998	-1.391508	H	-5.390679	0.177301	-4.811317
C	3.724420	5.948801	-2.548628	H	-3.619677	0.039231	-4.762057
H	5.514425	5.235066	-1.419745	C	-4.374628	2.427168	-2.696050
H	4.686826	6.662121	-0.738198	H	-2.811264	1.065830	-2.034454
H	1.515189	6.052926	-2.811964	H	-4.183730	1.125352	-0.896328
H	2.196951	7.153802	-1.579524	H	-3.347975	2.329861	-4.619467
H	3.770949	5.060986	-3.215366	H	-5.104553	2.574237	-4.781602
H	4.107732	6.812152	-3.130448	H	-3.743280	3.306677	-2.457455

H	-5.403430	2.692958	-2.377547	H	-1.374530	5.866512	2.953637
H	-9.240800	0.576401	-1.868289	H	-2.502621	6.796759	1.947253
C	-5.607434	6.774885	-0.957643	H	-5.523389	7.783004	-1.392074
C	-6.799948	6.045507	-1.115594	C	8.054508	-0.231838	3.555148
H	-7.641899	6.487729	-1.670232	C	8.250321	-0.828508	2.295285
C	-6.918516	4.754759	-0.575544	H	9.216366	-0.711148	1.780914
H	-7.845774	4.175034	-0.706450	C	7.221422	-1.565106	1.686203
C	-5.829271	4.208725	0.123634	H	7.374125	-2.020937	0.695534
C	-5.656924	2.886256	0.732230	C	5.990577	-1.693325	2.352780
C	-6.519504	1.781412	0.803577	C	4.732548	-2.331237	1.945521
H	-7.535045	1.823317	0.384104	C	4.373482	-3.025525	0.776790
C	-6.051592	0.593708	1.380017	H	5.104698	-3.196706	-0.028808
H	-6.704600	-0.291332	1.397175	C	3.057353	-3.484503	0.630783
C	-4.733083	0.490416	1.889397	H	2.765050	-4.001485	-0.295243
C	-3.878150	1.617263	1.833290	C	2.087197	-3.258507	1.636521
H	-2.856429	1.555441	2.235748	C	2.463018	-2.557584	2.809619
C	-4.344049	2.803420	1.259647	H	1.718742	-2.375584	3.597224
C	-4.623454	4.943202	0.291692	C	3.771580	-2.106584	2.964359
C	-4.515738	6.228881	-0.254736	C	5.794213	-1.096815	3.626528
H	-3.589170	6.812251	-0.153290	C	6.825702	-0.368278	4.228869
C	-3.605151	4.121082	1.080389	H	6.678616	0.101918	5.214587
C	-2.216438	3.992969	0.397235	C	4.387489	-1.363006	4.146075
C	-3.221027	4.785192	2.439900	C	4.399519	-2.241710	5.455471
C	-1.412593	5.242553	0.818866	C	3.585896	-0.076710	4.568056
H	-2.287363	3.865505	-0.699884	C	3.265429	-1.688313	6.328417
H	-1.723437	3.083251	0.789758	H	5.369638	-2.093071	5.974612
C	-2.089998	5.780095	2.111016	H	4.313806	-3.322188	5.226619
H	-2.845676	3.986236	3.109777	C	3.364842	-0.177196	6.086072
H	-4.096480	5.247957	2.937664	H	2.618121	-0.063522	4.034745
H	-0.350978	4.975994	0.982849	H	4.105006	0.850803	4.258308
H	-1.416790	6.008854	0.016778	H	2.282034	-2.066022	5.972284

H	3.360250	-1.980516	7.394373
H	2.473960	0.392095	6.421116
H	4.239944	0.229322	6.639968
H	8.868438	0.348801	4.015785
H	0.667120	1.886967	-0.972827

INT1

P	0.432227	-1.299917	0.300709
N	-0.113817	0.101413	-0.023832
P	-0.457257	1.645684	-0.061631
N	-0.131706	2.281269	-1.500074
S	0.117200	3.865447	-1.724024
O	1.389850	4.390260	-1.181504
O	-1.122762	4.676976	-1.511114
C	0.353227	3.858676	-3.603495
F	0.252993	5.120369	-4.029267
F	1.569814	3.384034	-3.914977
F	-0.577127	3.109862	-4.216209
O	-2.013732	1.679768	0.473418
C	-2.612524	2.913267	0.753071
C	-2.159729	3.649427	1.852283
C	-2.690607	4.977660	2.051928
C	-2.153230	5.889699	3.007503
C	-2.683410	7.163174	3.154253
C	-3.782915	7.585142	2.362107
C	-4.318881	6.729100	1.414333
C	-3.784880	5.420172	1.223683
C	-4.302822	4.548017	0.228391
C	-3.731245	3.309491	-0.048757
H	-5.160462	4.882663	-0.375124
H	-5.151743	7.054940	0.772065
H	-4.186275	8.601458	2.482587
H	-2.231942	7.860513	3.875468
H	-1.287651	5.586206	3.612389
C	-1.176052	3.075251	2.815509
C	0.065052	2.602486	2.384950
C	1.127578	2.206267	3.263882
C	0.837284	2.208318	4.627709
C	-0.442123	2.565255	5.133378
C	-0.722668	2.509375	6.532267
C	-1.982519	2.817880	7.022237
C	-3.014931	3.199937	6.124342
C	-2.764419	3.298584	4.762311
C	-1.475344	2.999413	4.225992
H	-3.569951	3.599488	4.078470
H	-4.020636	3.425355	6.510356
H	-2.186002	2.764635	8.102727
H	0.085655	2.202372	7.213310
H	1.626682	1.941046	5.345876
O	0.331934	2.588175	1.021715
N	0.472731	-2.323685	-0.998751
S	-0.164304	-3.724859	-1.129911
O	0.581487	-4.797663	-0.210782
O	-1.626662	-3.927978	-1.110723
C	0.489266	-4.289369	-2.819639
F	1.820737	-4.289454	-2.812776
F	0.035945	-5.529873	-3.028592
F	0.033802	-3.476940	-3.770905
O	1.938222	-1.172679	0.930734
C	2.540734	-2.302359	1.492897
C	2.010153	-2.831072	2.675645
C	2.575728	-4.052863	3.194465
C	1.995023	-4.771656	4.283104

C	2.567744	-5.945611	4.753278	H	-8.069364	-0.800862	-3.519955
C	3.755470	-6.455501	4.163944	C	-5.928024	-0.475816	-3.769036
C	4.334063	-5.792500	3.092088	C	-5.585845	0.583543	-2.812436
C	3.758877	-4.592881	2.572197	C	-6.365525	1.262992	-1.863959
C	4.321837	-3.925121	1.451231	H	-7.442679	1.062336	-1.765894
C	3.734589	-2.799226	0.875764	C	-5.734900	2.156561	-0.988866
H	5.242675	-4.326547	1.001386	H	-6.324949	2.645314	-0.198938
H	5.243681	-6.188193	2.613912	C	-4.340794	2.400905	-1.054137
H	4.206842	-7.381594	4.550804	C	-3.574119	1.747753	-2.047832
H	2.096704	-6.484487	5.589253	H	-2.501947	1.967835	-2.141699
H	1.078089	-4.387301	4.751341	C	-4.192797	0.833473	-2.901375
C	0.915795	-2.132153	3.409245	C	-4.737260	-0.882455	-4.427276
C	-0.291702	-1.834961	2.771844	C	-4.774025	-1.920069	-5.365109
C	-1.423210	-1.239948	3.419019	H	-3.854724	-2.249063	-5.875605
C	-1.259796	-0.932948	4.771762	C	-3.545934	-0.054380	-3.956539
C	-0.043055	-1.149962	5.470209	C	-2.359518	-0.920134	-3.382656
C	0.088901	-0.785910	6.844675	C	-2.884525	0.769971	-5.123551
C	1.293817	-0.942849	7.511084	C	-1.081928	-0.342680	-4.006843
C	2.418837	-1.471146	6.822637	H	-2.493546	-1.977894	-3.691275
C	2.313610	-1.865769	5.495905	H	-2.344540	-0.909130	-2.276372
C	1.081139	-1.737165	4.786479	C	-1.542199	0.081810	-5.406570
H	3.194389	-2.266992	4.976475	H	-2.697982	1.803302	-4.769232
H	3.383156	-1.568224	7.343955	H	-3.551628	0.840383	-6.004830
H	1.385467	-0.648851	8.567749	H	-0.745554	0.546355	-3.433562
H	-0.785508	-0.356116	7.355863	H	-0.243760	-1.065577	-4.002295
H	-2.107278	-0.505658	5.327488	H	-0.828923	0.746450	-5.934527
O	-0.397114	-2.180319	1.417613	H	-1.688875	-0.815953	-6.047501
C	-6.002407	-2.548600	-5.648235	H	-6.040184	-3.365813	-6.385003
C	-7.181327	-2.144713	-4.993397	C	8.834310	1.705387	-0.104756
H	-8.132454	-2.649834	-5.222420	C	8.829313	0.840143	1.005482
C	-7.152567	-1.106663	-4.046699	H	9.749415	0.304685	1.286304

C	7.656448	0.660121	1.756705	H	8.721608	1.758756	-5.105452
H	7.648354	-0.014979	2.626842	C	7.561920	0.602870	-3.676063
C	6.495503	1.359178	1.385681	H	8.376256	0.523187	-2.940258
C	5.159807	1.372992	1.989049	C	6.309192	0.023242	-3.414047
C	4.608145	0.665907	3.071962	C	5.843736	-0.731821	-2.249021
H	5.199690	-0.082926	3.621888	C	6.503266	-1.086488	-1.063127
C	3.283394	0.922646	3.447557	H	7.550995	-0.801374	-0.890015
H	2.845157	0.359812	4.283318	C	5.786936	-1.764769	-0.070910
C	2.489187	1.881003	2.767116	H	6.280764	-2.006079	0.882325
C	3.051826	2.569036	1.662617	C	4.419429	-2.093088	-0.240454
H	2.466588	3.319221	1.114328	C	3.769548	-1.741395	-1.448020
C	4.364991	2.305375	1.278894	H	2.717001	-2.013530	-1.598466
C	6.497928	2.245483	0.271136	C	4.480742	-1.070058	-2.445138
C	7.671467	2.408862	-0.477035	C	5.236827	0.133248	-4.340218
H	7.697650	3.076789	-1.349561	C	5.445183	0.805063	-5.553152
C	5.122827	2.890277	0.099603	H	4.646336	0.885336	-6.303310
C	5.142398	4.443443	0.020583	C	3.982740	-0.553565	-3.789475
C	4.435271	2.535464	-1.254193	C	2.760345	0.403720	-3.671608
C	5.417160	4.788902	-1.461396	C	3.415631	-1.651249	-4.741524
H	5.869646	4.890894	0.727269	C	2.182173	0.473313	-5.091014
H	4.134366	4.806944	0.307353	H	3.024669	1.390564	-3.253029
C	4.987933	3.539464	-2.283409	H	2.022936	-0.052540	-2.976311
H	3.344884	2.693876	-1.128902	C	2.442012	-0.926400	-5.708503
H	4.595094	1.476155	-1.524659	H	2.866346	-2.388993	-4.123731
H	4.857905	5.697388	-1.761201	H	4.226173	-2.203596	-5.256944
H	6.490512	5.019602	-1.622512	H	1.114358	0.754851	-5.081600
H	4.218082	3.781429	-3.042803	H	2.697632	1.265307	-5.670294
H	5.846976	3.104795	-2.834794	H	1.500296	-1.503731	-5.814646
H	9.759287	1.840584	-0.686820	H	2.867947	-0.844386	-6.729359
C	6.700407	1.386159	-5.816979	H	6.860489	1.920521	-6.766040
C	7.749075	1.292912	-4.883890	C	-8.930210	-1.226871	-0.411370

C	-8.917960	-0.118481	0.456067
H	-9.811816	0.518184	0.542904
C	-7.770226	0.180185	1.207505
H	-7.753996	1.049998	1.882375
C	-6.641187	-0.646239	1.081545
C	-5.329997	-0.570844	1.731653
C	-4.787681	0.351214	2.642762
H	-5.357804	1.236487	2.964008
C	-3.498768	0.135123	3.142463
H	-3.070831	0.864032	3.844004
C	-2.723779	-0.985899	2.748420
C	-3.263581	-1.875848	1.787042
H	-2.679884	-2.739940	1.444945
C	-4.551993	-1.670481	1.294476
C	-6.653792	-1.777262	0.219059
C	-7.800643	-2.059360	-0.534578
H	-7.830493	-2.914855	-1.224033
C	-5.307019	-2.498926	0.267402
C	-5.398897	-4.017318	0.590469
C	-4.585504	-2.523605	-1.110411
C	-5.667442	-4.718634	-0.759695
H	-6.155956	-4.237380	1.369615
H	-4.413862	-4.335897	0.993723
C	-5.128749	-3.756366	-1.858177
H	-3.502260	-2.643182	-0.923473
H	-4.723699	-1.574044	-1.654631
H	-5.183765	-5.716578	-0.794585
H	-6.753486	-4.902040	-0.893720
H	-4.331580	-4.221466	-2.472178
H	-5.931521	-3.466901	-2.566076
H	-9.834062	-1.448893	-0.999756

Si	-0.171089	-6.175872	0.671078
C	1.352919	-7.143683	1.144226
H	2.021387	-6.548691	1.795860
H	1.059776	-8.059255	1.698221
H	1.920838	-7.451507	0.243654
C	-1.105897	-5.439760	2.112314
H	-1.464249	-6.249360	2.781191
H	-0.458296	-4.765160	2.705107
H	-1.987530	-4.867295	1.762104
C	-1.287802	-7.096084	-0.520524
H	-1.720381	-7.974925	0.002071
H	-2.120944	-6.454099	-0.867930
H	-0.731833	-7.465456	-1.404599
C	-1.038633	8.232869	0.191351
N	-1.060248	7.163578	-0.311758
H	-1.070698	6.223681	-0.756535

INT1'

P	-0.435458	1.293021	0.334462
N	0.118737	-0.099816	-0.007312
P	0.460584	-1.642728	-0.101376
N	0.122387	-2.232333	-1.553945
S	-0.088785	-3.819653	-1.826750
O	-1.344745	-4.383505	-1.280663
O	1.167856	-4.606101	-1.663010
C	-0.367747	-3.750828	-3.698548
F	-0.279038	-4.999107	-4.167314
F	-1.589381	-3.265194	-3.973869
F	0.551873	-2.985355	-4.308680
O	2.020973	-1.689884	0.427561
C	2.623423	-2.924793	0.685527
C	2.176549	-3.681342	1.773476

C	2.715338	-5.009929	1.951322
C	2.185173	-5.940419	2.893495
C	2.725242	-7.211896	3.022565
C	3.826885	-7.614471	2.223693
C	4.354850	-6.741111	1.286850
C	3.811763	-5.432981	1.115544
C	4.323973	-4.541339	0.134157
C	3.745087	-3.301929	-0.122116
H	5.183852	-4.860411	-0.474604
H	5.190310	-7.051112	0.640009
H	4.239050	-8.628879	2.330076
H	2.279720	-7.922117	3.734893
H	1.318422	-5.651856	3.504027
C	1.191664	-3.128720	2.747585
C	-0.051186	-2.652576	2.325734
C	-1.114889	-2.276940	3.212549
C	-0.824623	-2.304939	4.576110
C	0.456184	-2.666636	5.074836
C	0.736299	-2.637415	6.474599
C	1.997742	-2.948939	6.958563
C	3.032339	-3.306904	6.053281
C	2.782206	-3.380038	4.689561
C	1.491342	-3.077909	4.159131
H	3.589600	-3.662285	3.999969
H	4.039456	-3.533855	6.434712
H	2.200932	-2.916066	8.039924
H	-0.073559	-2.347818	7.161371
H	-1.614945	-2.054665	5.299427
O	-0.318539	-2.614984	0.963942
N	-0.478901	2.339551	-0.947825
S	0.151995	3.745983	-1.048753
O	-0.602460	4.796835	-0.110815
O	1.613338	3.956166	-1.019621
C	-0.498038	4.341945	-2.728994
F	-1.829531	4.360480	-2.717797
F	-0.028422	5.578900	-2.921980
F	-0.057007	3.536745	-3.692986
O	-1.941898	1.151763	0.961711
C	-2.546330	2.266035	1.551028
C	-2.015416	2.770129	2.744502
C	-2.584829	3.977401	3.292156
C	-2.003369	4.674153	4.394648
C	-2.579836	5.834479	4.893293
C	-3.771958	6.352012	4.319674
C	-4.351181	5.710852	3.234958
C	-3.772303	4.526040	2.686071
C	-4.335451	3.881302	1.551855
C	-3.744161	2.771944	0.949181
H	-5.259598	4.288219	1.113848
H	-5.264170	6.113239	2.768967
H	-4.226205	7.266968	4.729027
H	-2.108255	6.356565	5.739563
H	-1.082595	4.283981	4.850470
C	-0.916671	2.059435	3.459820
C	0.290467	1.782383	2.812828
C	1.426040	1.177426	3.443585
C	1.267642	0.840154	4.789779
C	0.051797	1.036422	5.496065
C	-0.075028	0.642592	6.862784
C	-1.279326	0.778626	7.534933
C	-2.408970	1.314771	6.860290
C	-2.308789	1.738600	5.542181

C	-1.077049	1.632528	4.827958
H	-3.192725	2.145978	5.032952
H	-3.372677	1.394983	7.385597
H	-1.366880	0.461881	8.585365
H	0.802961	0.207104	7.362897
H	2.118386	0.404437	5.333874
O	0.389826	2.158536	1.466555
C	5.988587	2.676182	-5.604481
C	7.169373	2.264713	-4.957708
H	8.118238	2.778415	-5.176852
C	7.145247	1.208236	-4.031490
H	8.063403	0.896132	-3.510865
C	5.923586	0.566524	-3.766423
C	5.586371	-0.513165	-2.831019
C	6.369349	-1.207610	-1.896247
H	7.445445	-1.003444	-1.794084
C	5.743001	-2.121708	-1.039555
H	6.335431	-2.623033	-0.259327
C	4.350107	-2.371618	-1.110101
C	3.579984	-1.701996	-2.090124
H	2.508802	-1.925038	-2.187643
C	4.194404	-0.767538	-2.924677
C	4.730911	0.981037	-4.416160
C	4.762985	2.037003	-5.333465
H	3.842001	2.371749	-5.837104
C	3.543108	0.139359	-3.960852
C	2.354922	0.989348	-3.367396
C	2.882422	-0.663158	-5.143293
C	1.078177	0.419796	-4.000055
H	2.484499	2.053542	-3.655243
H	2.342532	0.955671	-2.261534
C	1.536228	0.024536	-5.409006
H	2.701385	-1.704558	-4.810335
H	3.547843	-0.711997	-6.027331
H	0.746472	-0.481353	-3.443458
H	0.237812	1.139696	-3.979059
H	0.824552	-0.633754	-5.946898
H	1.676710	0.934763	-6.033662
H	6.022720	3.507748	-6.325209
C	-8.826992	-1.755028	-0.139474
C	-8.828296	-0.918090	0.992249
H	-9.752614	-0.397746	1.287270
C	-7.656194	-0.747271	1.746766
H	-7.652520	-0.094285	2.633636
C	-6.489847	-1.426771	1.357022
C	-5.153344	-1.443439	1.958392
C	-4.605826	-0.756285	3.056192
H	-5.202355	-0.024348	3.623241
C	-3.278440	-1.010485	3.424074
H	-2.842821	-0.461630	4.270496
C	-2.478073	-1.948287	2.722431
C	-3.036264	-2.614393	1.602201
H	-2.445684	-3.346434	1.035498
C	-4.351468	-2.351538	1.225268
C	-6.485473	-2.284397	0.220194
C	-7.658617	-2.438975	-0.530480
H	-7.680790	-3.085025	-1.419482
C	-5.104699	-2.912018	0.031254
C	-5.110146	-4.462311	-0.090041
C	-4.421167	-2.514365	-1.312922
C	-5.384472	-4.769489	-1.580623
H	-5.831872	-4.935679	0.605346

H	-4.098040	-4.823924	0.184559	H	-3.028873	-1.298273	-3.289025
C	-4.967092	-3.494345	-2.368513	H	-2.033267	0.142467	-2.979268
H	-3.329132	-2.666767	-1.193371	C	-2.455304	1.086998	-5.680035
H	-4.590618	-1.449281	-1.553648	H	-2.904570	2.510193	-4.065586
H	-4.817868	-5.664516	-1.906040	H	-4.258247	2.330097	-5.206555
H	-6.456097	-5.004855	-1.746403	H	-1.130694	-0.616295	-5.109058
H	-4.196676	-3.709109	-3.135381	H	-2.718762	-1.103770	-5.705345
H	-5.830488	-3.051861	-2.906839	H	-1.513777	1.669568	-5.754720
H	-9.751394	-1.883465	-0.723980	H	-2.868203	1.038034	-6.708291
C	-6.712666	-1.245959	-5.843772	H	-6.874411	-1.751763	-6.808090
C	-7.756202	-1.190339	-4.901940	C	8.924856	1.275711	-0.401657
H	-8.726303	-1.657190	-5.131912	C	8.918745	0.149171	0.442129
C	-7.567353	-0.535694	-3.674803	H	9.816002	-0.484446	0.515302
H	-8.378007	-0.484574	-2.932383	C	7.773013	-0.171284	1.187639
C	-6.317881	0.046336	-3.402737	H	7.762071	-1.055155	1.844130
C	-5.852226	0.772374	-2.219485	C	6.639473	0.651517	1.079751
C	-6.508226	1.087598	-1.020566	C	5.329671	0.556716	1.730166
H	-7.552348	0.788072	-0.850054	C	4.793153	-0.385814	2.623616
C	-5.792524	1.744932	-0.013917	H	5.367404	-1.275464	2.924792
H	-6.282951	1.955569	0.948316	C	3.505038	-0.184357	3.131459
C	-4.428877	2.089488	-0.181657	H	3.081585	-0.928696	3.819397
C	-3.782389	1.777394	-1.401736	C	2.725068	0.942014	2.763119
H	-2.732439	2.061265	-1.549163	C	3.258596	1.852789	1.817905
C	-4.493646	1.128752	-2.413790	H	2.671101	2.721768	1.495223
C	-5.250075	-0.027288	-4.337613	C	4.546147	1.661816	1.317322
C	-5.460563	-0.662912	-5.569506	C	6.645747	1.800333	0.241023
H	-4.665753	-0.712683	-6.326657	C	7.790748	2.104475	-0.506884
C	-3.998434	0.653173	-3.773977	H	7.815664	2.974327	-1.178414
C	-2.770621	-0.300143	-3.683438	C	5.294930	2.513197	0.304424
C	-3.441805	1.778070	-4.700245	C	5.377580	4.026276	0.653354
C	-2.197509	-0.331000	-5.105630	C	4.571052	2.556414	-1.071825

C	5.636619	4.753413	-0.685157
H	6.135971	4.237735	1.433590
H	4.392000	4.331255	1.065527
C	5.105776	3.804886	-1.799224
H	3.487397	2.665929	-0.881298
H	4.714600	1.617079	-1.632189
H	5.142195	5.746562	-0.702007
H	6.720320	4.950899	-0.817824
H	4.305940	4.273937	-2.406707
H	5.911196	3.532087	-2.510713
H	9.827264	1.514983	-0.985484
Si	0.142384	6.161487	0.798095
C	-1.387433	7.107041	1.297145
H	-2.050198	6.492509	1.936280
H	-1.099601	8.011814	1.871291
H	-1.959772	7.430985	0.405067
C	1.088057	5.401988	2.219958
H	1.436339	6.198961	2.908989
H	0.449344	4.704408	2.795651
H	1.976469	4.849704	1.854849
C	1.246086	7.116423	-0.378102
H	1.681208	7.982126	0.163970
H	2.077819	6.486923	-0.750888
H	0.680699	7.508084	-1.246525
C	1.060113	-7.350195	-0.368472
N	1.068350	-8.398548	0.147197
H	1.055119	-6.359872	-0.850638

INT2

P	0.844118	-0.953700	-0.657118
N	-0.062778	-1.018017	0.619995
P	-1.232187	-0.404546	1.480837
N	-0.996422	1.015712	2.183071
S	-0.806588	1.441936	3.711921
O	0.091559	2.629873	3.777572
O	-0.595643	0.337995	4.676765
C	-2.503402	2.129176	4.190706
F	-2.488146	2.483143	5.480308
F	-2.804791	3.215557	3.448414
F	-3.448465	1.199263	3.997744
O	-2.545183	-0.363956	0.481110
C	-3.791897	-0.171532	1.070004
C	-4.361788	-1.233882	1.785002
C	-5.594525	-0.992203	2.493717
C	-6.187962	-1.954943	3.364048
C	-7.385514	-1.690721	4.013998
C	-8.049826	-0.449200	3.827665
C	-7.483113	0.520825	3.015859
C	-6.243410	0.288516	2.347343
C	-5.641933	1.294749	1.544585
C	-4.418207	1.107233	0.906341
H	-6.171435	2.251339	1.415945
H	-7.976514	1.496356	2.881749
H	-9.003741	-0.254148	4.340848
H	-7.819591	-2.448166	4.684234
H	-5.678501	-2.914873	3.524747
C	-3.679611	-2.564255	1.815206
C	-2.344753	-2.649279	2.225998
C	-1.589194	-3.867460	2.267471
C	-2.277067	-5.034398	1.937189
C	-3.629165	-5.025691	1.502955
C	-4.288229	-6.238388	1.136556
C	-5.581315	-6.228374	0.637027

C	-6.264088	-4.993261	0.478198	H	2.142432	-6.333809	-5.983554
C	-5.658797	-3.798819	0.847173	H	0.899197	-5.285661	-4.120544
C	-4.340138	-3.774630	1.391629	C	0.501172	-3.871609	-1.947635
H	-6.202821	-2.853764	0.722465	C	-0.386769	-2.796716	-2.041281
H	-7.282878	-4.979405	0.062032	C	-1.808685	-2.950502	-2.175053
H	-6.075882	-7.170440	0.355378	C	-2.313617	-4.234804	-1.970929
H	-3.735968	-7.184571	1.245186	C	-1.474193	-5.349122	-1.693684
H	-1.751399	-5.999573	1.988128	C	-2.018890	-6.643098	-1.437592
O	-1.719460	-1.474625	2.626021	C	-1.193233	-7.727959	-1.183474
N	1.428961	0.519804	-1.000113	C	0.217988	-7.561877	-1.186139
S	2.325337	0.961223	-2.232018	C	0.780339	-6.321610	-1.456706
O	3.430825	1.868203	-1.817381	C	-0.041850	-5.184611	-1.715114
O	2.650019	-0.119266	-3.216420	H	1.872720	-6.198161	-1.454650
C	1.166551	2.107095	-3.194105	H	0.870786	-8.421814	-0.972537
F	0.071014	1.452425	-3.599622	H	-1.628466	-8.718021	-0.978281
F	0.798558	3.147426	-2.425560	H	-3.113374	-6.757614	-1.433418
F	1.821472	2.581021	-4.268137	H	-3.395926	-4.410206	-2.064166
O	1.999239	-2.077227	-0.348923	O	0.138291	-1.503746	-2.048601
C	2.673475	-2.742227	-1.367314	C	-0.890327	8.017731	-2.342077
C	1.962044	-3.646257	-2.158254	C	-0.610016	6.972471	-3.243258
C	2.642750	-4.285953	-3.259115	H	0.029143	7.164617	-4.119187
C	1.980712	-5.117736	-4.211655	C	-1.143721	5.689025	-3.038419
C	2.679410	-5.702849	-5.258878	H	-0.915426	4.872069	-3.739218
C	4.074355	-5.485389	-5.411039	C	-1.959025	5.463559	-1.915365
C	4.743664	-4.663889	-4.518022	C	-2.593405	4.240150	-1.413571
C	4.052816	-4.039145	-3.436588	C	-2.565149	2.921083	-1.891658
C	4.724902	-3.149597	-2.557186	H	-2.062996	2.666885	-2.835494
C	4.068508	-2.464472	-1.536684	C	-3.153821	1.905410	-1.130371
H	5.802280	-2.983816	-2.706405	H	-3.110977	0.872571	-1.492453
H	5.818846	-4.460175	-4.640097	C	-3.798495	2.187030	0.098560
H	4.616393	-5.953673	-6.246530	C	-3.856127	3.529086	0.549427

H	-4.322410	3.749598	1.520611
C	-3.249291	4.542448	-0.193240
C	-2.239801	6.516028	-1.004665
C	-1.710061	7.793157	-1.219250
H	-1.923345	8.615393	-0.517093
C	-3.117061	6.026253	0.142492
C	-2.505392	6.288884	1.572179
C	-4.510914	6.757328	0.189009
C	-3.445136	7.290399	2.266298
H	-1.457077	6.641756	1.508131
H	-2.483728	5.338297	2.139986
C	-4.820737	6.950035	1.679163
H	-5.285670	6.195272	-0.368209
H	-4.412470	7.749673	-0.298925
H	-3.401228	7.222946	3.372309
H	-3.173606	8.333619	1.991554
H	-5.197327	6.002000	2.122063
H	-5.591413	7.725884	1.864644
H	-0.465822	9.018344	-2.517260
C	6.549444	-2.810250	4.205671
C	6.497169	-3.812157	3.218766
H	7.431966	-4.244769	2.830684
C	5.262287	-4.262890	2.725503
H	5.222464	-5.043024	1.949597
C	4.080378	-3.691426	3.227136
C	2.674253	-3.929772	2.881916
C	2.078360	-4.789036	1.944344
H	2.688216	-5.455375	1.314710
C	0.688257	-4.759197	1.786934
H	0.226643	-5.390553	1.015423
C	-0.130819	-3.890516	2.552864
C	0.480201	-3.045928	3.513888
H	-0.130727	-2.360586	4.117508
C	1.867958	-3.063641	3.663310
C	4.129771	-2.673567	4.217080
C	5.365528	-2.238957	4.711471
H	5.418167	-1.457709	5.486059
C	2.727984	-2.169162	4.547601
C	2.388127	-2.198938	6.084227
C	2.546740	-0.660088	4.154875
C	1.973336	-0.763715	6.472426
H	3.289423	-2.516330	6.647335
H	1.599569	-2.945601	6.303353
C	2.694815	0.133963	5.456302
H	1.515843	-0.522455	3.777013
H	3.237244	-0.358254	3.345909
H	0.881173	-0.628551	6.337779
H	2.213224	-0.521203	7.527878
H	2.244714	1.144547	5.375392
H	3.766853	0.259635	5.727028
H	7.525009	-2.468499	4.584516
C	8.929888	3.632253	1.638037
C	8.181183	3.129503	2.720006
H	8.310261	3.567019	3.721965
C	7.272810	2.073282	2.531152
H	6.689893	1.677376	3.377563
C	7.126351	1.525453	1.244552
C	6.263675	0.448532	0.748215
C	5.325368	-0.363426	1.404351
H	5.171903	-0.285332	2.490694
C	4.593685	-1.304165	0.667905
H	3.867243	-1.943447	1.184825

C	4.794602	-1.453692	-0.726761
C	5.751858	-0.631322	-1.374049
H	5.881546	-0.711298	-2.461751
C	6.486878	0.301572	-0.644021
C	7.880981	2.031040	0.151686
C	8.782654	3.082723	0.348434
H	9.375841	3.478421	-0.491873
C	7.578200	1.255513	-1.125372
C	7.151153	2.119837	-2.373634
C	8.839077	0.472083	-1.637264
C	8.091654	1.728345	-3.538583
H	7.216648	3.202885	-2.142565
H	6.091765	1.904504	-2.619571
C	8.638896	0.348070	-3.149990
H	8.966300	-0.491828	-1.105751
H	9.743065	1.084365	-1.431139
H	7.576297	1.720680	-4.519480
H	8.931803	2.452654	-3.609289
H	7.892556	-0.437810	-3.390820
H	9.569842	0.078438	-3.688704
H	9.636996	4.459942	1.801834
C	-7.753302	2.614545	-4.361819
C	-6.569059	3.020789	-5.006028
H	-6.564713	3.942486	-5.607891
C	-5.393327	2.263308	-4.882141
H	-4.465011	2.586278	-5.378153
C	-5.420084	1.094333	-4.102720
C	-4.361085	0.141718	-3.759154
C	-3.008323	0.093233	-4.127977
H	-2.587600	0.826055	-4.833514
C	-2.184049	-0.896650	-3.580256
H	-1.129434	-0.933081	-3.880197
C	-2.682579	-1.845564	-2.649443
C	-4.056168	-1.789496	-2.292422
H	-4.452237	-2.499987	-1.549927
C	-4.889503	-0.821492	-2.859290
C	-6.610940	0.685888	-3.450479
C	-7.780330	1.441893	-3.583128
H	-8.710701	1.132181	-3.080473
C	-6.389920	-0.601469	-2.665160
C	-6.842734	-0.529717	-1.164704
C	-7.240686	-1.790215	-3.261639
C	-8.052316	-1.469647	-1.027355
H	-7.050378	0.510261	-0.850215
H	-6.014727	-0.887082	-0.521476
C	-7.753582	-2.575346	-2.048712
H	-6.652656	-2.396971	-3.977712
H	-8.099269	-1.365464	-3.822042
H	-8.192473	-1.835979	0.011102
H	-8.990189	-0.943258	-1.310473
H	-6.953812	-3.242329	-1.662531
H	-8.628222	-3.215280	-2.286204
H	-8.665667	3.221947	-4.465481
C	5.836656	-0.420561	-5.423511
N	4.839861	-0.312163	-4.797645
H	3.976347	-0.218193	-4.214126
Si	1.595756	3.631789	1.472241
C	-0.093195	3.651614	0.707061
H	-0.223960	4.605823	0.161275
H	-0.220986	2.815828	-0.004222
H	-0.872718	3.558187	1.481285
C	2.488151	2.018225	1.758218

H	3.333415	2.130904	2.464733
H	1.763759	1.310910	2.199188
H	2.843101	1.571479	0.810744
C	1.912273	4.995226	2.719410
H	1.585106	5.979678	2.328944
H	1.315568	4.756193	3.622339
H	2.978873	5.056573	3.014541
C	3.547947	4.368874	-0.635125
O	2.439133	4.372482	-0.039535
C	3.590014	4.960672	-2.001991
H	2.663919	5.518469	-2.229268
H	4.495071	5.584078	-2.141028
H	3.680394	4.098898	-2.699135
C	4.807882	3.886931	0.002092
H	4.587288	3.063600	0.704229
H	5.474411	3.485178	-0.783083
C	5.503726	5.051796	0.741232
H	4.862086	5.461185	1.546198
H	6.438893	4.675197	1.196993
H	5.765869	5.880456	0.053068

INT2'

P	1.235779	1.030427	0.900639
N	0.692264	0.876723	-0.558064
P	-0.348379	0.568475	-1.694482
N	-0.517307	-0.988713	-2.047925
S	-0.343603	-1.855178	-3.383379
O	0.324123	-3.143658	-3.031696
O	0.116944	-1.127225	-4.585286
C	-2.116081	-2.399867	-3.763635
F	-2.666867	-2.974947	-2.678928
F	-2.861950	-1.352442	-4.132985
F	-2.085468	-3.297572	-4.759354
O	-1.757765	1.267085	-1.222606
C	-2.766481	1.480090	-2.157962
C	-2.574955	2.487423	-3.113888
C	-3.571349	2.662934	-4.138436
C	-3.392525	3.531886	-5.255793
C	-4.391829	3.687009	-6.206297
C	-5.620141	2.983271	-6.083884
C	-5.813879	2.110441	-5.024901
C	-4.797401	1.911600	-4.041234
C	-4.965394	0.981088	-2.982684
C	-3.962787	0.696181	-2.050806
H	-5.929279	0.456623	-2.907374
H	-6.752605	1.541711	-4.932604
H	-6.408953	3.121207	-6.838999
H	-4.228493	4.356594	-7.064379
H	-2.444044	4.076313	-5.363710
C	-1.368609	3.363383	-3.045059
C	-0.093272	2.793073	-3.008702
C	1.121531	3.549041	-2.912101
C	0.991237	4.936324	-2.870966
C	-0.272529	5.584670	-2.880320
C	-0.365863	7.006804	-2.797718
C	-1.597358	7.639623	-2.732555
C	-2.788281	6.865910	-2.743922
C	-2.731702	5.483038	-2.853487
C	-1.480844	4.799370	-2.944939
H	-3.663754	4.902197	-2.857804
H	-3.766296	7.364451	-2.665522
H	-1.654935	8.736581	-2.661154
H	0.567522	7.589270	-2.765184

H	1.897384	5.558848	-2.825079	C	1.075955	7.502379	0.289978
O	-0.005383	1.401582	-3.066761	C	2.336595	8.079335	0.241308
N	1.397705	-0.280456	1.849727	C	3.471758	7.346203	0.680957
S	0.337919	-0.774082	2.925755	C	3.327474	6.058466	1.179507
O	-1.078300	-0.464631	2.611246	C	2.044292	5.437710	1.243311
O	0.652542	-2.190854	3.305232	H	4.207934	5.494259	1.520541
C	0.752930	0.144720	4.549722	H	4.471858	7.802843	0.628073
F	0.051371	-0.428610	5.541918	H	2.461003	9.104723	-0.139247
F	2.064032	0.024438	4.810691	H	0.190497	8.058519	-0.053164
F	0.436441	1.442791	4.493791	H	-1.272571	6.167523	0.537916
O	2.736122	1.652238	0.693617	O	0.455963	2.151253	1.835616
C	3.353807	2.090932	1.865317	C	-6.689757	-5.597015	2.829400
C	2.953883	3.320317	2.384948	C	-5.840351	-4.819724	3.639475
C	3.519030	3.745116	3.642312	H	-5.685761	-5.089114	4.695542
C	3.123839	4.945814	4.304312	C	-5.180182	-3.697064	3.113923
C	3.682185	5.307972	5.522366	H	-4.520411	-3.096689	3.756732
C	4.660800	4.486436	6.141574	C	-5.382083	-3.366551	1.763170
C	5.050343	3.301535	5.536544	C	-4.842549	-2.270735	0.952257
C	4.490761	2.892925	4.288972	C	-3.919513	-1.257869	1.249884
C	4.859795	1.655133	3.688211	H	-3.454080	-1.181285	2.241532
C	4.310510	1.229810	2.484167	C	-3.589163	-0.316257	0.266685
H	5.601168	1.016711	4.193384	H	-2.894034	0.485373	0.534270
H	5.796337	2.648070	6.015594	C	-4.174631	-0.357795	-1.025296
H	5.099379	4.786032	7.105545	C	-5.063496	-1.425925	-1.323296
H	3.358632	6.238059	6.013962	H	-5.473856	-1.523907	-2.339692
H	2.360493	5.585462	3.840759	C	-5.395042	-2.362840	-0.349810
C	1.863407	4.092660	1.716454	C	-6.240441	-4.146145	0.944062
C	0.597576	3.510689	1.581877	C	-6.893753	-5.263288	1.475845
C	-0.579719	4.249023	1.235267	H	-7.561676	-5.877568	0.850216
C	-0.394285	5.567718	0.822546	C	-6.314520	-3.573021	-0.468561
C	0.894184	6.170912	0.770759	C	-5.840771	-4.590716	-1.573934

C	-7.777985	-3.188112	-0.907057	C	4.421090	-0.139511	-5.684344
C	-6.847665	-4.452962	-2.726270	C	3.611126	-1.380511	-3.695627
H	-5.885805	-5.619169	-1.159459	C	3.248282	-1.062856	-6.046657
H	-4.791910	-4.404301	-1.876656	H	5.366172	-0.555708	-6.091572
C	-8.172363	-4.198868	-1.995173	H	4.323429	0.889720	-6.082246
H	-7.766624	-2.163305	-1.332578	C	3.331851	-2.166794	-4.986445
H	-8.470080	-3.167964	-0.042343	H	2.649382	-1.005128	-3.292595
H	-6.593061	-3.573037	-3.357777	H	4.090232	-1.976069	-2.893448
H	-6.866395	-5.338181	-3.394500	H	2.276583	-0.537282	-5.935567
H	-8.985471	-3.831590	-2.654361	H	3.303419	-1.439455	-7.088402
H	-8.529356	-5.145257	-1.532590	H	2.401567	-2.763990	-4.915438
H	-7.200532	-6.473893	3.256906	H	4.170996	-2.861342	-5.214250
C	8.141127	-1.108631	-3.158866	H	8.901475	-1.889757	-3.312973
C	8.432295	-0.005151	-2.334334	C	6.047618	-6.661972	0.106117
H	9.417161	0.067206	-1.848424	C	6.483058	-5.823043	-0.937084
C	7.474290	1.000068	-2.122117	H	6.960658	-6.262252	-1.826465
H	7.702005	1.856566	-1.468950	C	6.316993	-4.429627	-0.850483
C	6.217066	0.884583	-2.740796	H	6.661475	-3.768489	-1.661472
C	5.018247	1.728683	-2.660227	C	5.707812	-3.890695	0.296791
C	4.739869	2.895740	-1.927750	C	5.411403	-2.501727	0.663856
H	5.498929	3.348189	-1.270605	C	5.612628	-1.303991	-0.039516
C	3.462068	3.463974	-2.010322	H	6.064499	-1.301528	-1.041518
H	3.223449	4.340230	-1.388532	C	5.228529	-0.097717	0.559708
C	2.455436	2.903437	-2.837382	H	5.364671	0.845828	0.013999
C	2.752008	1.730717	-3.575814	C	4.674535	-0.070610	1.859837
H	1.986157	1.276465	-4.218154	C	4.459406	-1.284989	2.553172
C	4.011809	1.145421	-3.469798	H	3.967068	-1.261377	3.536528
C	5.925183	-0.223833	-3.581078	C	4.813067	-2.494941	1.949526
C	6.886374	-1.219391	-3.790404	C	5.270475	-4.734110	1.352696
H	6.666371	-2.084221	-4.436939	C	5.440061	-6.120032	1.256989
C	4.499935	-0.145731	-4.113095	H	5.108601	-6.784420	2.071416

C	4.632456	-3.918345	2.472186
C	3.110064	-4.279654	2.697874
C	5.284045	-4.151760	3.880981
C	3.017844	-4.903571	4.099333
H	2.725069	-4.942781	1.897203
H	2.506266	-3.352726	2.678050
C	4.114803	-4.166647	4.877183
H	6.059550	-3.392636	4.103488
H	5.787598	-5.141247	3.887521
H	2.007024	-4.791804	4.541228
H	3.248327	-5.991552	4.065873
H	3.778845	-3.130385	5.100108
H	4.376456	-4.642116	5.844358
H	6.187536	-7.750995	0.025234
C	-7.498423	-0.202055	2.845956
C	-7.655359	0.329863	1.552676
H	-8.485615	-0.016757	0.917912
C	-6.745799	1.278084	1.056701
H	-6.846419	1.669468	0.032470
C	-5.671501	1.675092	1.869814
C	-4.503453	2.508957	1.567175
C	-4.156916	3.232638	0.415019
H	-4.854931	3.314424	-0.432226
C	-2.883915	3.812692	0.333749
H	-2.589548	4.355936	-0.573508
C	-1.935159	3.652034	1.368666
C	-2.312869	2.958617	2.549048
H	-1.588650	2.840944	3.368776
C	-3.596502	2.420022	2.656806
C	-5.509045	1.139005	3.174535
C	-6.427918	0.205371	3.665458
H	-6.302743	-0.239107	4.665245
C	-4.242058	1.684998	3.831135
C	-4.583320	2.701447	4.995252
C	-3.366390	0.595582	4.538215
C	-4.330688	1.927921	6.295511
H	-5.607962	3.110870	4.896709
H	-3.879693	3.558006	4.933785
C	-3.078675	1.108278	5.959119
H	-2.454613	0.368843	3.961164
H	-3.939441	-0.351694	4.598475
H	-4.213500	2.592859	7.175978
H	-5.181818	1.244869	6.509596
H	-2.184367	1.769839	5.958162
H	-2.872444	0.283470	6.670723
H	-8.205343	-0.961643	3.213200
Si	1.711355	-5.970253	-1.790185
C	3.091633	-5.028325	-2.616141
H	3.929640	-4.798580	-1.929770
H	2.692256	-4.095584	-3.056935
H	3.495296	-5.650414	-3.442617
C	2.291580	-7.318958	-0.629640
H	2.780944	-8.129659	-1.208047
H	3.042286	-6.917838	0.081346
H	1.448517	-7.758467	-0.060345
C	0.346203	-6.471181	-2.965398
H	-0.034731	-5.568447	-3.484674
H	0.728834	-7.184698	-3.723963
H	-0.490796	-6.952358	-2.421470
O	0.788027	-4.914181	-0.668999
C	0.731678	-3.712896	-0.244510
C	-0.553301	-3.269857	0.336749

H	-0.329621	-2.970295	1.387905
H	-0.773755	-2.294598	-0.165288
C	-1.714682	-4.247387	0.236182
H	-1.507271	-5.187132	0.784742
H	-1.918048	-4.501279	-0.821717
H	-2.628447	-3.790119	0.658358
C	1.884535	-2.788995	-0.308495
H	1.915423	-2.133054	0.585544
H	2.847595	-3.303129	-0.460995
H	1.691536	-2.109682	-1.167306
C	-1.788136	-2.924962	5.014334
N	-2.801659	-3.028190	5.585899
H	-0.848315	-2.783132	4.459459

TS0

P	1.075555	-0.727607	-1.058805
N	0.336117	-0.465446	0.310705
P	-0.873412	-0.137302	1.228427
N	-0.931749	1.363485	1.857487
S	-0.481920	1.924436	3.238307
O	0.090220	3.355676	3.013595
O	0.291877	1.084383	4.170750
C	-2.073577	2.379509	4.171346
F	-1.758948	2.720134	5.420863
F	-2.711647	3.394903	3.577161
F	-2.858825	1.298975	4.176906
O	-2.269172	-0.379517	0.409042
C	-3.436149	-0.238218	1.165945
C	-3.782921	-1.280359	2.031265
C	-4.920611	-1.091392	2.896369
C	-5.297246	-2.034731	3.898297
C	-6.410596	-1.821382	4.699048
C	-7.201128	-0.652081	4.540013
C	-6.842423	0.301200	3.599810
C	-5.694233	0.121856	2.770660
C	-5.300493	1.119370	1.836883
C	-4.171445	0.982755	1.032276
H	-5.914415	2.028527	1.744871
H	-7.435129	1.221903	3.482111
H	-8.087764	-0.498551	5.173692
H	-6.679359	-2.562915	5.466620
H	-4.688221	-2.938614	4.035750
C	-2.950873	-2.521978	2.052838
C	-1.571283	-2.430894	2.276410
C	-0.684883	-3.555771	2.289247
C	-1.265579	-4.809330	2.098346
C	-2.654460	-4.978202	1.857091
C	-3.209633	-6.277213	1.650546
C	-4.554183	-6.441694	1.356757
C	-5.395269	-5.302456	1.248367
C	-4.889056	-4.027236	1.462592
C	-3.516756	-3.823355	1.797056
H	-5.554619	-3.158732	1.376587
H	-6.458577	-5.428124	0.993864
H	-4.969302	-7.448621	1.198113
H	-2.537656	-7.146578	1.714333
H	-0.626638	-5.704551	2.129863
O	-1.027641	-1.163781	2.503502
N	1.663190	0.615839	-1.723520
S	2.166469	0.806840	-3.238385
O	3.293010	1.796636	-3.262989
O	2.301364	-0.399286	-4.076858
C	0.743656	1.816265	-3.981181

F	-0.401531	1.109654	-3.943230	H	-0.761382	-8.803412	-0.674576
F	0.569482	2.952132	-3.285794	H	-2.464076	-7.018689	-1.094715
F	1.026457	2.119081	-5.252199	H	-3.023096	-4.732274	-1.771912
O	2.189072	-1.851045	-0.632600	O	0.182882	-1.506725	-2.211946
C	2.889572	-2.502291	-1.644936	C	-1.416432	7.949540	-2.791510
C	2.218085	-3.495608	-2.353898	C	-1.174046	6.889024	-3.685271
C	2.899694	-4.126170	-3.457516	H	-0.656714	7.085986	-4.636718
C	2.273916	-5.071794	-4.323317	C	-1.581175	5.582393	-3.371094
C	2.972019	-5.653760	-5.372509	H	-1.375946	4.751449	-4.062263
C	4.331137	-5.318603	-5.609968	C	-2.234538	5.353114	-2.148043
C	4.961162	-4.383262	-4.804142	C	-2.742426	4.116604	-1.545577
C	4.269384	-3.754729	-3.725265	C	-2.728445	2.789800	-2.003243
C	4.901500	-2.759105	-2.930608	H	-2.324415	2.532486	-2.992463
C	4.239006	-2.100999	-1.897920	C	-3.207195	1.773832	-1.168240
H	5.948227	-2.495619	-3.147414	H	-3.179573	0.736737	-1.521463
H	6.008199	-4.097919	-4.992021	C	-3.719480	2.061606	0.120064
H	4.876308	-5.790143	-6.441749	C	-3.762057	3.409447	0.556078
H	2.463975	-6.376256	-6.029319	H	-4.125326	3.636880	1.568645
H	1.219595	-5.333370	-4.160192	C	-3.270132	4.424360	-0.265147
C	0.800861	-3.828235	-2.021661	C	-2.469841	6.418795	-1.241094
C	-0.191105	-2.840946	-2.071066	C	-2.065483	7.718574	-1.563596
C	-1.598423	-3.138780	-2.062754	H	-2.242802	8.553410	-0.866361
C	-1.958173	-4.455153	-1.776163	C	-3.144143	5.916373	0.031032
C	-0.995839	-5.473027	-1.534733	C	-2.305483	6.210068	1.337485
C	-1.389435	-6.799958	-1.186807	C	-4.528994	6.603101	0.310509
C	-0.443639	-7.786529	-0.951594	C	-3.152422	7.176073	2.184820
C	0.939675	-7.483245	-1.065154	H	-1.302397	6.608887	1.089220
C	1.354188	-6.209967	-1.432255	H	-2.143828	5.264448	1.891450
C	0.406317	-5.171958	-1.681246	C	-4.594810	6.789338	1.832716
H	2.426629	-5.983449	-1.516609	H	-5.368885	6.018700	-0.113851
H	1.689428	-8.263121	-0.862725	H	-4.541943	7.597492	-0.182839

H	-2.922806	7.109269	3.267817	H	4.144574	0.386890	4.035445
H	-2.966466	8.227953	1.875698	H	1.297702	-1.041028	6.187077
H	-4.862095	5.831618	2.331252	H	2.274405	-0.859377	7.673498
H	-5.348502	7.540678	2.145376	H	2.309760	1.209574	5.923869
H	-1.089433	8.967758	-3.052697	H	3.904241	0.587568	6.461250
C	7.409272	-2.073814	4.129393	H	8.369043	-1.690437	4.508501
C	7.400003	-3.025249	3.091609	C	7.910183	4.830078	1.141720
H	8.352475	-3.377333	2.666925	C	7.316507	4.180611	2.240567
C	6.186823	-3.520149	2.587570	H	7.354729	4.648874	3.236202
H	6.180656	-4.252497	1.765624	C	6.674471	2.942413	2.075292
C	4.982224	-3.046830	3.135035	H	6.208061	2.433238	2.933448
C	3.586141	-3.328806	2.781771	C	6.631510	2.367912	0.793381
C	3.029625	-4.182520	1.815738	C	6.004833	1.132221	0.315695
H	3.670275	-4.794610	1.162120	C	5.292549	0.136021	1.001618
C	1.638059	-4.229533	1.673403	H	5.173610	0.171092	2.094238
H	1.198829	-4.873912	0.899371	C	4.727879	-0.918673	0.277656
C	0.784300	-3.427431	2.469925	H	4.161388	-1.691854	0.810734
C	1.357876	-2.556929	3.431371	C	4.871008	-1.000583	-1.129101
H	0.709193	-1.916581	4.044925	C	5.623158	-0.008597	-1.806731
C	2.744601	-2.521949	3.592298	H	5.703715	-0.052035	-2.903798
C	4.989659	-2.088758	4.180994	C	6.190878	1.045746	-1.088034
C	6.202834	-1.603299	4.681339	C	7.230071	3.020152	-0.315323
H	6.218054	-0.854319	5.489551	C	7.873291	4.250026	-0.140998
C	3.571831	-1.700727	4.579132	H	8.339336	4.767247	-0.995160
C	3.234134	-2.055716	6.074070	C	7.051971	2.202829	-1.590722
C	3.291901	-0.154186	4.490187	C	6.425077	3.000139	-2.792799
C	2.334330	-0.914796	6.567171	C	8.435396	1.694935	-2.157517
H	4.177497	-2.062264	6.659647	C	7.535570	3.129763	-3.848766
H	2.785184	-3.064359	6.165929	H	6.010905	3.971883	-2.461319
C	2.970869	0.320256	5.918336	H	5.580038	2.419158	-3.210957
H	2.416236	0.017094	3.837672	C	8.326044	1.826438	-3.682074

H	8.671442	0.671843	-1.804638
H	9.240254	2.362771	-1.785869
H	7.137698	3.284635	-4.872301
H	8.195366	3.995581	-3.619173
H	7.743800	0.977357	-4.102672
H	9.313448	1.830083	-4.187828
H	8.405448	5.802684	1.286101
C	-8.247655	1.735889	-3.833352
C	-7.191019	2.185230	-4.648182
H	-7.343243	3.051820	-5.309618
C	-5.944030	1.540680	-4.618972
H	-5.115035	1.897351	-5.249481
C	-5.770507	0.440979	-3.761637
C	-4.595224	-0.391303	-3.491757
C	-3.299377	-0.356747	-4.028270
H	-3.035401	0.353799	-4.826242
C	-2.329424	-1.234779	-3.530795
H	-1.321071	-1.211449	-3.961309
C	-2.623136	-2.147750	-2.486243
C	-3.942133	-2.179967	-1.959819
H	-4.177938	-2.869802	-1.134138
C	-4.922049	-1.324671	-2.472274
C	-6.832122	-0.011168	-2.938144
C	-8.073751	0.632618	-2.976041
H	-8.906555	0.289411	-2.341111
C	-6.401529	-1.210374	-2.101427
C	-6.669013	-1.052226	-0.563597
C	-7.211590	-2.506322	-2.496060
C	-7.761421	-2.071941	-0.202135
H	-6.930872	-0.010187	-0.300120
H	-5.737212	-1.285868	-0.012623
C	-7.497174	-3.229762	-1.174297
H	-6.666577	-3.120441	-3.239375
H	-8.168326	-2.197766	-2.966117
H	-7.737424	-2.363630	0.868784
H	-8.770600	-1.646453	-0.395469
H	-6.599525	-3.798816	-0.851771
H	-8.335354	-3.952775	-1.248520
H	-9.218241	2.254577	-3.864686
C	3.233238	4.338922	-0.412866
N	3.403429	3.616201	-1.327004
H	3.418400	2.898161	-2.104632
Si	1.358311	3.828379	1.687347
C	0.296806	3.778588	0.144443
H	0.446284	4.682993	-0.475564
H	0.538290	2.890986	-0.471897
H	-0.770424	3.715759	0.428235
C	2.610890	2.463288	1.990404
H	2.677094	2.256598	3.075881
H	2.314884	1.531386	1.467201
H	3.614682	2.745763	1.622714
C	1.739535	5.505632	2.426485
H	2.484412	6.022283	1.792698
H	0.819127	6.121190	2.480139
H	2.149152	5.395247	3.450136

TS0'

P	0.793628	0.368624	1.516475
N	-0.262197	0.262076	0.363673
P	-0.877473	0.591480	-1.059552
N	-1.436672	-0.647004	-1.928142
S	-0.660166	-1.909147	-2.475230
O	-1.217448	-3.194261	-1.874355

O	0.812266	-1.806018	-2.577593
C	-1.275517	-2.015737	-4.261012
F	-0.897735	-0.904052	-4.901401
F	-0.714708	-3.081070	-4.843714
F	-2.609975	-2.131988	-4.319058
O	-2.148789	1.579563	-0.784192
C	-2.766112	2.100247	-1.932192
C	-2.116739	3.130973	-2.609250
C	-2.673547	3.591910	-3.859993
C	-2.024659	4.549183	-4.697237
C	-2.592298	4.951766	-5.897994
C	-3.837067	4.418899	-6.325439
C	-4.479859	3.465809	-5.551245
C	-3.916185	3.018205	-4.318865
C	-4.547542	2.003620	-3.547089
C	-3.998074	1.520375	-2.364158
H	-5.502674	1.582282	-3.897120
H	-5.434851	3.027116	-5.880468
H	-4.280502	4.752040	-7.276053
H	-2.067737	5.687286	-6.526672
H	-1.054910	4.963045	-4.390604
C	-0.815327	3.665623	-2.118349
C	0.287546	2.814992	-1.990049
C	1.634363	3.288200	-1.872402
C	1.804586	4.662948	-1.702363
C	0.708321	5.567702	-1.663705
C	0.900826	6.968542	-1.470685
C	-0.178112	7.838785	-1.430616
C	-1.499695	7.340763	-1.587398
C	-1.718504	5.989386	-1.817573
C	-0.627782	5.070683	-1.873694
H	-2.741585	5.609215	-1.951809
H	-2.354925	8.031525	-1.535034
H	-0.013449	8.915697	-1.273064
H	1.926926	7.341972	-1.339948
H	2.824772	5.069230	-1.630354
O	0.094210	1.438213	-2.086991
N	0.680065	-0.952822	2.521006
S	1.534616	-1.187540	3.957495
O	1.734135	-2.636655	4.103987
O	2.626221	-0.218194	4.131082
C	0.264333	-0.720344	5.297583
F	0.045590	0.604132	5.283849
F	-0.893826	-1.358259	5.105818
F	0.780521	-1.070662	6.476654
O	2.249693	0.643285	0.821760
C	3.372776	0.933764	1.612243
C	3.431466	2.146239	2.305042
C	4.505249	2.324751	3.254969
C	4.554429	3.418615	4.170763
C	5.600756	3.553955	5.072211
C	6.656018	2.604542	5.103065
C	6.622281	1.511675	4.251517
C	5.547229	1.330036	3.330365
C	5.465635	0.167075	2.517811
C	4.379725	-0.076020	1.684130
H	6.265379	-0.586819	2.581583
H	7.416623	0.749639	4.285620
H	7.485717	2.727043	5.815737
H	5.606431	4.401346	5.774550
H	3.740129	4.155555	4.172856
C	2.398699	3.203736	2.108345

C	1.037278	2.914666	2.241797
C	-0.012539	3.886832	2.130809
C	0.390489	5.205367	1.915978
C	1.751305	5.572998	1.747088
C	2.121860	6.932826	1.519354
C	3.441214	7.283513	1.279802
C	4.443470	6.277766	1.248969
C	4.120263	4.952125	1.504871
C	2.776475	4.560852	1.788381
H	4.911647	4.190644	1.497811
H	5.486945	6.550037	1.029525
H	3.711914	8.335198	1.100288
H	1.328927	7.695861	1.515777
H	-0.370763	5.998289	1.876080
O	0.668152	1.607418	2.571683
C	-7.775294	-5.264513	0.876142
C	-7.836618	-4.262610	1.863551
H	-8.307239	-4.482725	2.834072
C	-7.301134	-2.987154	1.620440
H	-7.347584	-2.199905	2.389622
C	-6.703708	-2.732458	0.374259
C	-6.058522	-1.524886	-0.150532
C	-5.832781	-0.273173	0.443133
H	-6.199246	-0.050655	1.455522
C	-5.122306	0.698350	-0.274009
H	-4.914822	1.673176	0.187052
C	-4.679772	0.449850	-1.591487
C	-4.931298	-0.806383	-2.190971
H	-4.551619	-1.000535	-3.204398
C	-5.600144	-1.795034	-1.464965
C	-6.636756	-3.741026	-0.621607
C	-7.174729	-5.008188	-0.371678
H	-7.128260	-5.800907	-1.135753
C	-5.913750	-3.233722	-1.865860
C	-4.608243	-4.058233	-2.199441
C	-6.770651	-3.338783	-3.177634
C	-4.869677	-4.754975	-3.545202
H	-4.350930	-4.759683	-1.382408
H	-3.751866	-3.365520	-2.301132
C	-5.792456	-3.773224	-4.278799
H	-7.304478	-2.392525	-3.394582
H	-7.544414	-4.122747	-3.039517
H	-3.932056	-4.979225	-4.093696
H	-5.399680	-5.720178	-3.389635
H	-5.200967	-2.904679	-4.640801
H	-6.302475	-4.211365	-5.161061
H	-8.198645	-6.259483	1.083072
C	7.869577	-1.577600	-4.740495
C	8.387125	-0.892218	-3.624694
H	9.435559	-1.048287	-3.327716
C	7.576209	-0.015074	-2.886969
H	7.980238	0.517824	-2.012313
C	6.239515	0.165132	-3.281386
C	5.171828	0.996220	-2.715825
C	5.169121	1.893445	-1.634714
H	6.073383	2.047854	-1.026340
C	3.995269	2.598556	-1.338315
H	3.984940	3.312777	-0.504124
C	2.808894	2.402329	-2.083639
C	2.809261	1.452325	-3.138471
H	1.890266	1.276513	-3.714750
C	3.986429	0.778580	-3.467235

C	5.715263	-0.527129	-4.403371
C	6.530502	-1.397308	-5.135201
H	6.132155	-1.941553	-6.006730
C	4.245172	-0.191216	-4.619875
C	3.958588	0.472520	-6.016018
C	3.303093	-1.456050	-4.610694
C	2.578889	-0.051334	-6.436492
H	4.722240	0.120239	-6.741264
H	4.034864	1.576867	-5.971259
C	2.630650	-1.518655	-5.995026
H	2.533510	-1.338384	-3.826034
H	3.873592	-2.374589	-4.369751
H	1.772132	0.479176	-5.885304
H	2.376676	0.084578	-7.518726
H	1.637047	-2.009138	-5.959394
H	3.256501	-2.097636	-6.709114
H	8.517809	-2.263771	-5.307024
C	3.291218	-8.001456	-0.885731
C	3.139057	-7.186272	-2.023842
H	2.860332	-7.641349	-2.986786
C	3.337430	-5.798605	-1.938905
H	3.212937	-5.158158	-2.826106
C	3.689135	-5.240906	-0.697603
C	3.915352	-3.848302	-0.302985
C	3.809672	-2.666203	-1.051376
H	3.571518	-2.695733	-2.122724
C	3.952808	-1.434326	-0.405561
H	3.845221	-0.504267	-0.977652
C	4.226738	-1.373564	0.980033
C	4.345321	-2.572587	1.726527
H	4.493747	-2.503228	2.813271
C	4.190960	-3.808136	1.089396
C	3.835513	-6.058146	0.452027
C	3.640720	-7.440606	0.357504
H	3.751370	-8.087270	1.242868
C	4.208368	-5.223156	1.670145
C	3.274324	-5.407506	2.904393
C	5.593943	-5.610388	2.270359
C	4.117297	-5.026791	4.148287
H	2.971695	-6.474003	2.950618
H	2.346050	-4.809435	2.820813
C	5.598481	-4.995977	3.679660
H	6.435713	-5.280409	1.628928
H	5.641154	-6.719069	2.330875
H	3.795263	-4.047243	4.553115
H	3.961427	-5.762077	4.963368
H	5.971351	-3.951951	3.633921
H	6.280697	-5.538547	4.365042
H	3.130289	-9.087471	-0.967917
C	-7.882028	1.215945	3.616360
C	-7.982320	2.265905	2.685227
H	-8.971150	2.574027	2.312957
C	-6.828326	2.926005	2.234292
H	-6.904564	3.752033	1.510400
C	-5.575080	2.512607	2.716997
C	-4.227799	3.009522	2.418888
C	-3.771109	4.010279	1.543176
H	-4.475741	4.582418	0.919734
C	-2.396002	4.257537	1.448122
H	-2.035274	5.006148	0.727618
C	-1.455391	3.542780	2.235134
C	-1.934123	2.536582	3.112305

H	-1.233116	1.979887	3.743933
C	-3.298903	2.260121	3.181548
C	-5.464942	1.435902	3.641183
C	-6.626312	0.797743	4.099446
H	-6.572912	-0.022075	4.829788
C	-3.995989	1.142682	3.942602
C	-3.653142	0.991986	5.449841
C	-3.563953	-0.257348	3.397111
C	-3.956241	-0.485585	5.812462
H	-4.198193	1.726273	6.075647
H	-2.570446	1.197502	5.574359
C	-4.012428	-1.263569	4.466524
H	-2.457696	-0.258608	3.309414
H	-3.969090	-0.455767	2.387403
H	-3.174691	-0.890759	6.485303
H	-4.913359	-0.574442	6.366297
H	-3.374344	-2.169311	4.458507
H	-5.044673	-1.610251	4.253376
H	-8.794575	0.713392	3.971997
C	-0.916053	-3.103626	2.542045
N	-1.459966	-3.873476	3.255029
H	-0.087086	-1.922027	2.375136
Si	-1.152284	-3.437248	0.305334
C	-2.588875	-2.229581	0.479863
H	-3.294755	-2.685263	1.201424
H	-2.277643	-1.250739	0.882924
H	-3.108802	-2.068286	-0.480075
C	0.716271	-3.266504	0.149804
H	1.077588	-2.240458	-0.038583
H	1.216203	-3.645656	1.060513
H	1.034078	-3.886652	-0.709545
C	-1.691985	-5.233851	0.246209
H	-0.930337	-5.881248	0.723001
H	-2.655738	-5.374129	0.772351
H	-1.806694	-5.542576	-0.811837

TS1

P	0.850379	-0.955443	-0.682415
N	-0.059827	-1.047504	0.593185
P	-1.222953	-0.428468	1.457148
N	-0.993191	1.003974	2.139613
S	-0.754036	1.444978	3.653159
O	0.156757	2.630584	3.678143
O	-0.517672	0.359492	4.631454
C	-2.432115	2.151186	4.172246
F	-2.379162	2.516819	5.457220
F	-2.748058	3.231252	3.427669
F	-3.385027	1.223473	4.012647
O	-2.548351	-0.397784	0.476475
C	-3.787308	-0.208374	1.083539
C	-4.340450	-1.273770	1.806780
C	-5.566719	-1.041586	2.528998
C	-6.143897	-2.009196	3.404765
C	-7.336133	-1.753537	4.067805
C	-8.010894	-0.516448	3.889336
C	-7.460279	0.457671	3.071392
C	-6.226274	0.234372	2.389225
C	-5.640861	1.244760	1.579868
C	-4.422611	1.067106	0.927600
H	-6.179425	2.197021	1.457168
H	-7.962288	1.429541	2.942793
H	-8.960449	-0.328258	4.413055

H	-7.757965	-2.514351	4.742035	C	2.726142	-5.659734	-5.312112
H	-5.626145	-2.965880	3.558538	C	4.117378	-5.422404	-5.468465
C	-3.646410	-2.598222	1.828886	C	4.779186	-4.597115	-4.573313
C	-2.306931	-2.671336	2.226565	C	4.084423	-3.987796	-3.485635
C	-1.538092	-3.881245	2.254264	C	4.748820	-3.095927	-2.602498
C	-2.217106	-5.053374	1.922968	C	4.087768	-2.426666	-1.574605
C	-3.572928	-5.056601	1.500796	H	5.823632	-2.916046	-2.753980
C	-4.221768	-6.273649	1.130395	H	5.851111	-4.378550	-4.698262
C	-5.519112	-6.273883	0.641974	H	4.662394	-5.878626	-6.308678
C	-6.217232	-5.045292	0.499589	H	2.195031	-6.294211	-6.038044
C	-5.622046	-3.847228	0.873070	H	0.944857	-5.272659	-4.164568
C	-4.298699	-3.812800	1.405308	C	0.534316	-3.868211	-1.986422
H	-6.177338	-2.907272	0.760599	C	-0.363546	-2.801102	-2.072878
H	-7.239726	-5.039406	0.092401	C	-1.785078	-2.965737	-2.197092
H	-6.005485	-7.219089	0.356602	C	-2.277872	-4.254745	-1.993067
H	-3.658278	-7.214526	1.226652	C	-1.427229	-5.362466	-1.723369
H	-1.681068	-6.013304	1.963246	C	-1.959515	-6.660965	-1.464085
O	-1.689155	-1.488878	2.619635	C	-1.123293	-7.739113	-1.215880
N	1.411948	0.528366	-1.007302	C	0.286478	-7.561642	-1.228434
S	2.314421	0.996590	-2.224685	C	0.836885	-6.316665	-1.502091
O	3.413814	1.900221	-1.788413	C	0.003703	-5.186243	-1.753886
O	2.647976	-0.065432	-3.226323	H	1.928198	-6.184097	-1.507635
C	1.148239	2.150273	-3.167858	H	0.947663	-8.416413	-1.019961
F	0.064524	1.488767	-3.595093	H	-1.549034	-8.732721	-1.007945
F	0.760806	3.167056	-2.378654	H	-3.052997	-6.784566	-1.452588
F	1.802643	2.656940	-4.227095	H	-3.359348	-4.439054	-2.078692
O	2.016792	-2.068795	-0.381417	O	0.150297	-1.503672	-2.076976
C	2.696284	-2.720888	-1.404672	C	-1.031263	8.025903	-2.367249
C	1.992697	-3.627760	-2.199329	C	-0.720454	6.977754	-3.254932
C	2.678127	-4.253841	-3.304929	H	-0.081343	7.175596	-4.129580
C	2.023653	-5.089623	-4.259155	C	-1.222877	5.683865	-3.037561

H	-0.970649	4.864670	-3.727512	C	5.318185	-4.257007	2.675273
C	-2.038086	5.451463	-1.915887	H	5.278028	-5.026768	1.889108
C	-2.647156	4.219281	-1.405272	C	4.136427	-3.696574	3.189841
C	-2.593594	2.898455	-1.875484	C	2.729209	-3.930285	2.845506
H	-2.084460	2.648352	-2.816600	C	2.131821	-4.780387	1.900799
C	-3.167928	1.876924	-1.111285	H	2.740791	-5.439128	1.262403
H	-3.108372	0.843967	-1.470030	C	0.741088	-4.752975	1.749548
C	-3.819570	2.153126	0.115320	H	0.278058	-5.379322	0.974817
C	-3.901780	3.497005	0.558443	C	-0.077028	-3.893856	2.526929
H	-4.373394	3.715247	1.527758	C	0.536136	-3.053606	3.490731
C	-3.314318	4.517513	-0.189972	H	-0.075170	-2.373848	4.100283
C	-2.349218	6.506542	-1.018526	C	1.924488	-3.071584	3.637508
C	-1.850374	7.793927	-1.245440	C	4.187660	-2.692853	4.193122
H	-2.086923	8.618716	-0.553806	C	5.423517	-2.255700	4.684293
C	-3.221096	6.008114	0.129122	H	5.475087	-1.480006	5.464921
C	-2.628609	6.307116	1.559078	C	2.787477	-2.199650	4.542551
C	-4.634499	6.701941	0.154748	C	2.450271	-2.296570	6.077598
C	-3.603377	7.287607	2.235085	C	2.600687	-0.674150	4.212952
H	-1.591489	6.691655	1.497403	C	1.907331	-0.915323	6.489087
H	-2.580596	5.364842	2.138834	H	3.381357	-2.527888	6.634932
C	-4.963441	6.901757	1.639754	H	1.742052	-3.123369	6.283164
H	-5.388678	6.113801	-0.403844	C	2.637721	0.061265	5.557508
H	-4.557795	7.691440	-0.342715	H	1.600780	-0.532287	3.761866
H	-3.567393	7.232653	3.342093	H	3.344480	-0.319307	3.475841
H	-3.359866	8.335533	1.952302	H	0.819794	-0.847439	6.282768
H	-5.317039	5.948108	2.089594	H	2.057568	-0.700907	7.567033
H	-5.757630	7.657179	1.809783	H	2.147356	1.054003	5.496716
H	-0.630678	9.034631	-2.551902	H	3.686145	0.216945	5.897263
C	6.607161	-2.814770	4.164918	H	7.583474	-2.471232	4.540172
C	6.553546	-3.806607	3.167639	C	8.876112	3.695512	1.663277
H	7.488145	-4.230030	2.769085	C	8.138908	3.167438	2.741087

H	8.266473	3.592366	3.748658
C	7.244296	2.101615	2.541044
H	6.670533	1.685729	3.384060
C	7.099844	1.569911	1.247538
C	6.250614	0.487688	0.739760
C	5.325730	-0.345797	1.387774
H	5.174751	-0.285022	2.475507
C	4.603956	-1.286105	0.641411
H	3.886292	-1.939840	1.152376
C	4.803771	-1.416087	-0.755353
C	5.748320	-0.573109	-1.394551
H	5.876665	-0.638412	-2.483412
C	6.471070	0.362080	-0.654976
C	7.842441	2.101360	0.158885
C	8.730421	3.162484	0.366646
H	9.314061	3.578255	-0.470632
C	7.543119	1.341605	-1.128384
C	7.088705	2.221137	-2.356586
C	8.812434	0.590207	-1.665481
C	8.025880	1.868239	-3.536568
H	7.135850	3.300835	-2.106034
H	6.031058	1.991066	-2.596460
C	8.601170	0.491073	-3.178572
H	8.961160	-0.381155	-1.153433
H	9.707511	1.214176	-1.455373
H	7.502296	1.869696	-4.513154
H	8.852249	2.608841	-3.601006
H	7.866973	-0.303461	-3.427847
H	9.531885	0.248171	-3.730275
H	9.572906	4.530151	1.835854
C	-7.786593	2.576586	-4.283565
C	-6.609670	2.999011	-4.930797
H	-6.615935	3.927941	-5.521410
C	-5.427825	2.248482	-4.824053
H	-4.505123	2.584048	-5.322140
C	-5.441150	1.070129	-4.058601
C	-4.373035	0.121283	-3.733740
C	-3.023172	0.085927	-4.114327
H	-2.613568	0.828678	-4.816011
C	-2.187208	-0.903113	-3.583047
H	-1.134798	-0.928116	-3.891857
C	-2.671197	-1.864439	-2.657188
C	-4.042364	-1.821897	-2.288753
H	-4.426857	-2.541949	-1.549328
C	-4.887421	-0.854786	-2.839531
C	-6.624355	0.645681	-3.402711
C	-7.800006	1.394744	-3.518514
H	-8.724654	1.072561	-3.013141
C	-6.388423	-0.648916	-2.633873
C	-6.835228	-0.600226	-1.130428
C	-7.230702	-1.838245	-3.240901
C	-8.030201	-1.559917	-0.998277
H	-7.057391	0.432932	-0.803643
H	-5.998644	-0.951716	-0.495062
C	-7.721235	-2.647911	-2.035101
H	-6.643058	-2.426151	-3.972839
H	-8.100527	-1.415131	-3.785064
H	-8.159816	-1.941228	0.036131
H	-8.977239	-1.043960	-1.269754
H	-6.907755	-3.306335	-1.662778
H	-8.587037	-3.299029	-2.274589
H	-8.703928	3.178617	-4.373898

C	5.825009	-0.332949	-5.449336
N	4.831957	-0.231363	-4.816385
H	3.971132	-0.145269	-4.227524
Si	1.524914	3.592499	1.550050
C	-0.154682	3.699554	0.763309
H	-0.237694	4.662950	0.224150
H	-0.299195	2.877670	0.038748
H	-0.953900	3.627966	1.518378
C	2.419828	1.960085	1.728559
H	3.135893	1.992289	2.572362
H	1.676394	1.169611	1.931604
H	2.946136	1.670498	0.799933
C	1.941246	4.937882	2.791462
H	1.666098	5.937891	2.400239
H	1.361211	4.745339	3.714690
H	3.018730	4.936432	3.052290
C	3.492733	4.402951	-0.545117
O	2.375284	4.361485	0.027218
C	3.541437	5.000156	-1.911116
H	2.608216	5.542794	-2.146029
H	4.436286	5.639325	-2.041945
H	3.649610	4.141690	-2.609711
C	4.759307	3.955397	0.108096
H	4.547579	3.164948	0.849772
H	5.418952	3.517517	-0.664467
C	5.465856	5.151054	0.784358
H	4.827628	5.609111	1.565612
H	6.397545	4.790430	1.259930
H	5.737039	5.938957	0.053412

TS1'

P	0.861433	-0.935523	-0.684171
N	-0.042526	-1.010342	0.597980
P	-1.216289	-0.407276	1.456814
N	-1.014656	1.033967	2.130265
S	-0.773344	1.486049	3.640265
O	0.130994	2.676603	3.656819
O	-0.529413	0.408458	4.625607
C	-2.455228	2.185100	4.156102
F	-2.402566	2.563714	5.437464
F	-2.780813	3.255689	3.401828
F	-3.401665	1.248954	4.007513
O	-2.543041	-0.407024	0.476981
C	-3.783793	-0.232022	1.084225
C	-4.320072	-1.300311	1.815773
C	-5.549136	-1.081348	2.537441
C	-6.111444	-2.051431	3.420104
C	-7.307196	-1.809167	4.081895
C	-8.000442	-0.583572	3.895361
C	-7.464534	0.393199	3.070823
C	-6.227561	0.183822	2.389639
C	-5.657509	1.197592	1.573472
C	-4.437615	1.033104	0.920918
H	-6.209783	2.141254	1.445308
H	-7.980978	1.356623	2.935986
H	-8.952586	-0.406088	4.418126
H	-7.717365	-2.571668	4.761405
H	-5.579371	-2.999188	3.580151
C	-3.604733	-2.613232	1.847564
C	-2.264060	-2.661393	2.244884
C	-1.475825	-3.858635	2.281352
C	-2.135284	-5.044141	1.958235

C	-3.491348	-5.072553	1.537478	H	5.851373	-2.886736	-2.715800
C	-4.121069	-6.302519	1.176940	H	5.893102	-4.344389	-4.660869
C	-5.418471	-6.327219	0.689298	H	4.721491	-5.863608	-6.266894
C	-6.136087	-5.111015	0.537702	H	2.257948	-6.302020	-5.998408
C	-5.559903	-3.900909	0.902036	H	0.993997	-5.282564	-4.132665
C	-4.237183	-3.841377	1.433458	C	0.566599	-3.870313	-1.960419
H	-6.129837	-2.970623	0.782491	C	-0.342731	-2.812967	-2.051747
H	-7.158663	-5.124388	0.130877	C	-1.762331	-2.995040	-2.176346
H	-5.889795	-7.282266	0.411513	C	-2.240920	-4.288600	-1.967798
H	-3.542520	-7.233474	1.279764	C	-1.378332	-5.385475	-1.692185
H	-1.583665	-5.994943	2.004950	C	-1.896335	-6.688720	-1.428274
O	-1.665245	-1.466522	2.627967	C	-1.048572	-7.756030	-1.172602
N	1.424309	0.538925	-1.033965	C	0.359026	-7.562240	-1.181785
S	2.294483	0.990814	-2.287635	C	0.895822	-6.312377	-1.460419
O	3.403073	1.898564	-1.877928	C	0.050463	-5.192891	-1.720669
O	2.595507	-0.068509	-3.292537	H	1.985571	-6.167312	-1.463781
C	1.098115	2.146469	-3.190546	H	1.029487	-8.408184	-0.966956
F	0.014929	1.477456	-3.610419	H	-1.463469	-8.753456	-0.961041
F	0.709028	3.147929	-2.382049	H	-2.988342	-6.824551	-1.418790
F	1.724779	2.679382	-4.255138	H	-3.320098	-4.485484	-2.054305
O	2.026391	-2.048790	-0.367214	O	0.154657	-1.510864	-2.065717
C	2.715758	-2.698833	-1.383373	C	-1.146523	8.011364	-2.432880
C	2.022618	-3.617106	-2.174323	C	-0.817450	6.958974	-3.308924
C	2.716128	-4.244418	-3.274001	H	-0.178387	7.157455	-4.183478
C	2.071382	-5.090552	-4.225736	C	-1.300682	5.659851	-3.079601
C	2.781604	-5.659358	-5.274244	H	-1.033094	4.837178	-3.759625
C	4.170945	-5.409674	-5.429025	C	-2.116020	5.427022	-1.958076
C	4.823901	-4.575487	-4.535571	C	-2.708652	4.191414	-1.436980
C	4.121082	-3.968934	-3.451343	C	-2.636467	2.867969	-1.896822
C	4.776477	-3.069419	-2.568953	H	-2.123408	2.617934	-2.835695
C	4.106116	-2.395964	-1.549449	C	-3.196632	1.844679	-1.124733

H	-3.123184	0.809899	-1.475520	C	0.820008	-4.692533	1.785486
C	-3.850495	2.121547	0.100447	H	0.370359	-5.327403	1.009716
C	-3.951150	3.467502	0.533452	C	-0.015293	-3.845204	2.557368
H	-4.422891	3.685383	1.502664	C	0.579416	-2.992507	3.521980
C	-3.380852	4.490798	-0.224576	H	-0.046287	-2.320926	4.125935
C	-2.444712	6.486064	-1.071946	C	1.967075	-2.988727	3.677014
C	-1.964638	7.778391	-1.310535	C	4.221205	-2.577555	4.246330
H	-2.214626	8.606178	-0.627196	C	5.447531	-2.120777	4.743549
C	-3.312974	5.986592	0.077742	H	5.482864	-1.343557	5.523634
C	-2.737880	6.313272	1.509004	C	2.812186	-2.104853	4.587742
C	-4.738408	6.652735	0.085134	C	2.465698	-2.211073	6.120057
C	-3.766427	7.233078	2.193869	C	2.605845	-0.580581	4.261585
H	-1.727408	6.762862	1.445852	C	1.880072	-0.845968	6.525008
H	-2.627539	5.372988	2.082968	H	3.399285	-2.412951	6.684821
C	-5.101663	6.811877	1.566495	H	1.781291	-3.058768	6.321133
H	-5.468408	6.064390	-0.504642	C	2.604005	0.150039	5.609456
H	-4.668006	7.655390	-0.386733	H	1.613389	-0.452857	3.790703
H	-3.746084	7.149205	3.299420	H	3.358476	-0.209775	3.541336
H	-3.562009	8.297486	1.944304	H	0.795172	-0.804421	6.298232
H	-5.431938	5.838393	1.990414	H	2.004531	-0.631005	7.606143
H	-5.923765	7.537027	1.735797	H	2.093293	1.132063	5.543568
H	-0.760422	9.024020	-2.626682	H	3.642843	0.326756	5.967941
C	6.642392	-2.661720	4.230712	H	7.611329	-2.302736	4.610642
C	6.609248	-3.655020	3.233952	C	8.846104	3.780329	1.657010
H	7.552317	-4.064056	2.840333	C	8.099885	3.264197	2.734358
C	5.383588	-4.124811	2.735609	H	8.215115	3.703750	3.737116
H	5.359398	-4.895238	1.949429	C	7.211769	2.191966	2.540032
C	4.190602	-3.582572	3.243716	H	6.631198	1.785170	3.382798
C	2.788901	-3.837130	2.891297	C	7.082686	1.641976	1.252552
C	2.210203	-4.697552	1.944266	C	6.242379	0.549860	0.751214
H	2.833413	-5.347109	1.310225	C	5.310788	-0.275624	1.399672

H	5.146975	-0.200323	2.484584
C	4.596988	-1.225614	0.657958
H	3.872152	-1.870695	1.169480
C	4.812837	-1.375575	-0.734418
C	5.766062	-0.541711	-1.372579
H	5.915620	-0.626507	-2.457168
C	6.479147	0.404796	-0.638906
C	7.834141	2.161549	0.164208
C	8.715921	3.228896	0.366453
H	9.306373	3.635829	-0.470392
C	7.551094	1.382907	-1.115612
C	7.100182	2.241855	-2.359179
C	8.829044	0.633808	-1.632715
C	8.027457	1.854080	-3.537729
H	7.164717	3.325396	-2.130429
H	6.036910	2.023632	-2.585738
C	8.621447	0.493857	-3.143145
H	8.988955	-0.322743	-1.096688
H	9.716184	1.273787	-1.437007
H	7.493603	1.818919	-4.507945
H	8.845164	2.600051	-3.637548
H	7.902407	-0.318264	-3.376713
H	9.557821	0.252938	-3.686185
H	9.537487	4.620344	1.825105
C	-7.832972	2.461208	-4.288864
C	-6.662135	2.894099	-4.940193
H	-6.680547	3.819267	-5.536444
C	-5.470973	2.158866	-4.830341
H	-4.553056	2.502657	-5.331634
C	-5.468726	0.985273	-4.057585
C	-4.388581	0.051627	-3.728502

C	-3.038568	0.031043	-4.109687
H	-2.638571	0.775288	-4.815297
C	-2.189599	-0.944055	-3.573110
H	-1.136708	-0.956156	-3.881071
C	-2.661171	-1.906306	-2.641814
C	-4.032410	-1.878559	-2.272478
H	-4.406968	-2.598995	-1.528455
C	-4.889944	-0.925284	-2.827900
C	-6.645838	0.550317	-3.397547
C	-7.830776	1.284105	-3.516415
H	-8.750711	0.953694	-3.007715
C	-6.393036	-0.736531	-2.621180
C	-6.837890	-0.685137	-1.117008
C	-7.220840	-1.940018	-3.219515
C	-8.013660	-1.667239	-0.974539
H	-7.080097	0.345828	-0.797646
H	-5.992942	-1.014490	-0.480957
C	-7.689796	-2.753896	-2.008120
H	-6.629548	-2.520232	-3.954665
H	-8.101346	-1.531570	-3.757745
H	-8.130724	-2.046296	0.062189
H	-8.971803	-1.170760	-1.243329
H	-6.862481	-3.395740	-1.637205
H	-8.544669	-3.421834	-2.240485
H	-8.757784	3.051308	-4.381689
C	5.025357	-0.511002	-4.912924
N	6.073264	-0.708823	-5.391692
H	4.060946	-0.314270	-4.410439
Si	1.488255	3.622040	1.503032
C	-0.200844	3.730960	0.737098
H	-0.287708	4.691577	0.193311

H	-0.359203	2.905355	0.019842
H	-0.989118	3.666527	1.504304
C	2.379908	1.989262	1.691664
H	3.094411	2.026822	2.536493
H	1.635776	1.200050	1.897115
H	2.906444	1.693596	0.765227
C	1.921141	4.974666	2.731003
H	1.645269	5.973478	2.337334
H	1.349420	4.788846	3.660896
H	3.000991	4.971057	2.981728
C	3.443353	4.409062	-0.609985
O	2.327323	4.371366	-0.034197
C	3.486935	4.994280	-1.981051
H	2.555265	5.540076	-2.214662
H	4.384902	5.626280	-2.124114
H	3.583840	4.127299	-2.670941
C	4.712156	3.968534	0.044018
H	4.502270	3.187486	0.796130
H	5.367870	3.520104	-0.725704
C	5.423441	5.171220	0.702425
H	4.788924	5.641213	1.479559
H	6.355689	4.814801	1.180097
H	5.694388	5.948774	-0.039598

TS2-*S*

P	0.979085	-0.332229	-1.543403
N	-0.169652	-0.559293	-0.503552
P	-0.807574	-1.035494	0.857228
N	-1.505903	0.068561	1.797407
S	-0.899394	0.889578	3.032080
O	-1.729425	2.098834	3.269907
O	0.576911	1.014704	3.044877
C	-1.308937	-0.205686	4.532240
F	-0.673643	-1.386620	4.460469
F	-0.924900	0.426801	5.652877
F	-2.634252	-0.437322	4.601683
O	-1.997167	-2.085899	0.415992
C	-2.593134	-2.788569	1.464591
C	-1.887366	-3.860078	2.010829
C	-2.431954	-4.537209	3.162852
C	-1.735618	-5.568339	3.862584
C	-2.297186	-6.185212	4.971942
C	-3.582326	-5.803096	5.439933
C	-4.274444	-4.787481	4.799186
C	-3.720145	-4.123482	3.664142
C	-4.402833	-3.046740	3.035326
C	-3.866573	-2.357541	1.951726
H	-5.386862	-2.742389	3.424615
H	-5.263178	-4.466898	5.163368
H	-4.019107	-6.304287	6.317098
H	-1.736903	-6.974307	5.496273
H	-0.736103	-5.868982	3.520660
C	-0.545491	-4.222635	1.474829
C	0.490280	-3.281231	1.499244
C	1.866261	-3.637911	1.309749
C	2.129680	-4.940775	0.886787
C	1.100216	-5.899626	0.683316
C	1.387352	-7.218019	0.218775
C	0.373088	-8.144445	0.028188
C	-0.974871	-7.788084	0.301991
C	-1.283976	-6.523556	0.784840
C	-0.262103	-5.549369	0.995985
H	-2.328537	-6.253901	0.998317

H	-1.779656	-8.519895	0.133897
H	0.609321	-9.154964	-0.339050
H	2.433469	-7.478183	-0.000969
H	3.174909	-5.252510	0.739976
O	0.197125	-1.956521	1.804136
N	0.996216	1.207050	-2.071965
S	1.650392	1.866320	-3.413707
O	1.849045	3.309508	-3.147134
O	2.697405	1.076027	-4.086531
C	0.186606	1.837738	-4.615373
F	-0.165132	0.568931	-4.893275
F	-0.877893	2.472800	-4.082392
F	0.528868	2.452572	-5.751440
O	2.398747	-0.796776	-0.857135
C	3.530141	-0.706863	-1.674906
C	3.706124	-1.689660	-2.651176
C	4.798516	-1.544976	-3.578701
C	4.978665	-2.401163	-4.705265
C	6.051649	-2.230874	-5.568795
C	6.998957	-1.196039	-5.347455
C	6.835763	-0.328025	-4.279139
C	5.732154	-0.462766	-3.383584
C	5.517814	0.470457	-2.333657
C	4.409731	0.407511	-1.491167
H	6.236927	1.294130	-2.205999
H	7.547583	0.495979	-4.113433
H	7.850191	-1.074510	-6.034597
H	6.164531	-2.899392	-6.435890
H	4.247251	-3.199054	-4.893954
C	2.754043	-2.834858	-2.721851
C	1.374978	-2.610559	-2.821367
C	0.403194	-3.662578	-2.888693
C	0.889396	-4.969568	-2.899350
C	2.272033	-5.270406	-2.781734
C	2.734241	-6.620892	-2.764170
C	4.073792	-6.914269	-2.561005
C	5.003552	-5.859951	-2.356113
C	4.590250	-4.535532	-2.402118
C	3.223508	-4.196869	-2.642494
H	5.323066	-3.731137	-2.251794
H	6.062018	-6.092469	-2.164105
H	4.416212	-7.960262	-2.542997
H	1.995587	-7.426927	-2.891785
H	0.176710	-5.803057	-2.989931
O	0.909128	-1.295999	-2.875808
C	-7.115260	5.105751	-0.004800
C	-7.013610	4.392901	-1.215077
H	-7.225649	4.903713	-2.167143
C	-6.644169	3.037191	-1.218186
H	-6.557974	2.480157	-2.164280
C	-6.372571	2.405884	0.008172
C	-5.867610	1.059504	0.303085
C	-5.470431	0.021141	-0.556066
H	-5.642551	0.078939	-1.641664
C	-4.812334	-1.091561	-0.017044
H	-4.457309	-1.887875	-0.683135
C	-4.576204	-1.192400	1.371662
C	-5.024642	-0.165767	2.234460
H	-4.781946	-0.232485	3.302822
C	-5.666572	0.954950	1.705573
C	-6.475260	3.122318	1.231770
C	-6.851990	4.470335	1.225268

H	-6.939135	5.034487	2.167942
C	-6.136730	2.223816	2.415035
C	-5.098773	2.780884	3.433954
C	-7.373202	1.966842	3.333201
C	-5.407516	2.098553	4.794088
H	-5.236106	3.879407	3.514549
H	-4.057958	2.596173	3.107047
C	-6.779286	1.391574	4.627364
H	-8.126367	1.313161	2.849490
H	-7.861612	2.945622	3.530771
H	-4.608725	1.380773	5.068082
H	-5.435893	2.850607	5.608128
H	-6.642483	0.295701	4.521005
H	-7.451671	1.539614	5.496362
H	-7.404998	6.167820	-0.019846
C	7.719934	1.170350	4.973190
C	8.270873	0.744660	3.749447
H	9.288726	1.058777	3.471370
C	7.530893	-0.072794	2.880096
H	7.959394	-0.400202	1.920123
C	6.231582	-0.457082	3.252343
C	5.234810	-1.278969	2.558774
C	5.287972	-1.967429	1.335200
H	6.188385	-1.929106	0.703101
C	4.170748	-2.703264	0.919281
H	4.203163	-3.249075	-0.034060
C	2.982178	-2.733808	1.686573
C	2.926079	-2.001933	2.900789
H	2.002641	-2.002936	3.496403
C	4.053326	-1.309768	3.345720
C	5.673472	-0.026939	4.484033
C	6.418406	0.784642	5.346369
H	5.992523	1.125974	6.303744
C	4.255410	-0.555821	4.659048
C	4.113663	-1.512058	5.901921
C	3.179730	0.562493	4.928208
C	2.715698	-1.233200	6.469377
H	4.878663	-1.229313	6.655572
H	4.296452	-2.570857	5.631586
C	2.595731	0.288992	6.326379
H	2.373932	0.489010	4.174824
H	3.620214	1.574025	4.831137
H	1.934082	-1.729826	5.854180
H	2.591692	-1.597723	7.509930
H	1.556708	0.661596	6.425307
H	3.204870	0.785229	7.113886
H	8.311235	1.814167	5.642575
C	2.538285	7.521567	2.582220
C	2.161434	6.459432	3.427171
H	1.661741	6.675330	4.384294
C	2.416291	5.128222	3.059385
H	2.106807	4.293245	3.706654
C	3.053502	4.877811	1.831710
C	3.418241	3.616567	1.184366
C	3.249444	2.294022	1.618557
H	2.811121	2.073727	2.599630
C	3.588080	1.243861	0.760167
H	3.436515	0.211404	1.094906
C	4.102484	1.499716	-0.534045
C	4.286963	2.843229	-0.952982
H	4.625060	3.032562	-1.980508
C	3.953936	3.898627	-0.098876

C	3.409916	5.943007	0.965892	C	-3.041446	-2.253211	-3.728688
C	3.161699	7.266970	1.345124	C	-5.290106	-1.598784	-4.040318
H	3.439203	8.104967	0.685066	C	-6.504914	-0.983579	-4.365705
C	4.011516	5.412027	-0.328888	H	-6.537806	-0.142727	-5.076931
C	3.250120	5.879390	-1.604761	C	-3.883278	-1.250221	-4.514093
C	5.448327	5.927236	-0.631547	C	-3.722541	-1.382520	-6.078480
C	4.242540	5.707348	-2.776516	C	-3.495296	0.243325	-4.217704
H	2.988347	6.950806	-1.470285	C	-3.010168	-0.102222	-6.545317
H	2.308924	5.317695	-1.751386	H	-4.730452	-1.443155	-6.537983
C	5.659875	5.661160	-2.135306	H	-3.187681	-2.312420	-6.353944
H	6.209413	5.453146	0.019803	C	-3.526910	0.965764	-5.572379
H	5.472242	7.018659	-0.423672	H	-2.469469	0.272845	-3.802674
H	4.014264	4.776144	-3.332246	H	-4.158386	0.695638	-3.455348
H	4.143381	6.535617	-3.507253	H	-1.911215	-0.204160	-6.429615
H	6.122122	4.663873	-2.283860	H	-3.207014	0.132930	-7.611223
H	6.359029	6.394805	-2.585277	H	-2.924126	1.896080	-5.569521
H	2.337405	8.560079	2.888497	H	-4.569166	1.248956	-5.839023
C	-7.692621	-1.448747	-3.768518	H	-8.651493	-0.965953	-4.012165
C	-7.664245	-2.524696	-2.861423	C	0.040998	3.635973	0.730272
H	-8.600801	-2.874333	-2.400894	N	0.528568	2.952598	-0.094266
C	-6.450942	-3.152216	-2.536531	H	0.825262	2.278084	-0.880010
H	-6.429625	-3.987146	-1.819314	C	-2.457509	4.167171	0.301287
C	-5.264022	-2.683549	-3.125752	O	-2.194231	4.796291	-0.781976
C	-3.867299	-3.094916	-2.939691	C	-2.617394	4.864049	1.607040
C	-3.294219	-4.093015	-2.134746	H	-2.061221	5.815466	1.643354
H	-3.919675	-4.738935	-1.499062	H	-3.706440	5.065331	1.730038
C	-1.902578	-4.243605	-2.128643	H	-2.295506	4.192808	2.426350
H	-1.445725	-4.997253	-1.471479	C	-2.849454	2.747427	0.167013
C	-1.062933	-3.414493	-2.914085	H	-2.351187	2.177670	0.980636
C	-1.653087	-2.404757	-3.715649	H	-3.918949	2.718371	0.483646
H	-1.014563	-1.753960	-4.328047	C	-2.660015	2.110803	-1.198250

H	-1.607956	2.176836	-1.532725
H	-2.930749	1.041041	-1.145376
H	-3.293366	2.596304	-1.966813
Si	-1.447703	6.320160	-1.356242
C	-0.255475	6.984833	-0.077061
H	0.420305	7.713843	-0.570905
H	-0.758117	7.506247	0.760957
H	0.380300	6.178584	0.342012
C	-2.949619	7.409577	-1.641913
H	-3.448964	7.665194	-0.685222
H	-2.650080	8.358036	-2.134019
H	-3.688371	6.902124	-2.294611
C	-0.629060	5.717783	-2.919964
H	-0.099361	6.549921	-3.427819
H	0.114645	4.921511	-2.709529
H	-1.378865	5.299870	-3.620995

TS2-*R*

P	0.570911	-1.275291	-0.856813
N	0.013082	-0.605110	0.449620
P	-1.096413	0.160676	1.267306
N	-0.757013	1.660480	1.736060
S	-0.427561	2.264741	3.181788
O	0.137484	3.629535	3.014910
O	0.252829	1.322940	4.105205
C	-2.083604	2.621029	4.041504
F	-1.822155	2.998073	5.299527
F	-2.737371	3.618519	3.419650
F	-2.863564	1.533183	4.063604
O	-2.461197	0.154339	0.344348
C	-3.580005	0.701736	0.973839
C	-4.270146	-0.096233	1.892489
C	-5.354784	0.502169	2.631438
C	-6.047168	-0.172974	3.680280
C	-7.094387	0.437456	4.356884
C	-7.499413	1.756731	4.022044
C	-6.819787	2.456538	3.036993
C	-5.726239	1.865702	2.335888
C	-4.987295	2.608739	1.375256
C	-3.899509	2.069454	0.694723
H	-5.280401	3.650828	1.175396
H	-7.105021	3.490359	2.786703
H	-8.338142	2.226771	4.557723
H	-7.610136	-0.104736	5.164027
H	-5.737413	-1.189547	3.958085
C	-3.819743	-1.502377	2.118825
C	-2.479217	-1.762861	2.440747
C	-1.948927	-3.079744	2.650451
C	-2.858208	-4.135870	2.578893
C	-4.226681	-3.946509	2.249514
C	-5.120168	-5.056108	2.152023
C	-6.433171	-4.885277	1.740887
C	-6.904359	-3.586515	1.410252
C	-6.071076	-2.482928	1.532534
C	-4.718704	-2.619681	1.971887
H	-6.456669	-1.485683	1.283514
H	-7.939133	-3.448577	1.060293
H	-7.107153	-5.751855	1.661101
H	-4.732875	-6.058837	2.388693
H	-2.505686	-5.160106	2.771768
O	-1.610637	-0.687696	2.585918
N	1.627593	-0.387035	-1.728874
S	1.379379	0.436882	-3.109756

O	-0.039675	0.600746	-3.495322	C	-1.365880	-5.350275	-1.040505
O	2.290036	1.605022	-3.126585	H	0.347928	-6.669971	-0.752721
C	2.082544	-0.759617	-4.402557	H	-1.006169	-8.592703	0.062930
F	1.493064	-1.957374	-4.285680	H	-3.511700	-8.409420	0.207459
F	1.855048	-0.270340	-5.626606	H	-4.642119	-6.265649	-0.408105
F	3.409842	-0.905274	-4.231448	H	-4.526906	-3.973713	-1.274146
O	1.370298	-2.605350	-0.324087	O	-0.525032	-1.857563	-1.939863
C	1.788610	-3.525341	-1.289486	C	1.490171	7.463388	-2.848509
C	0.818802	-4.331295	-1.884722	C	1.458321	6.331670	-3.685463
C	1.210726	-5.208846	-2.961585	H	2.099671	6.296699	-4.579698
C	0.277772	-5.981357	-3.717037	C	0.620248	5.243036	-3.387220
C	0.701602	-6.803883	-4.751578	H	0.616047	4.345012	-4.024110
C	2.078804	-6.897050	-5.084767	C	-0.198230	5.312710	-2.246325
C	3.008210	-6.140728	-4.388426	C	-1.173210	4.360142	-1.705406
C	2.604407	-5.273795	-3.328877	C	-1.579653	3.094559	-2.158104
C	3.547824	-4.464310	-2.640589	H	-1.157600	2.643997	-3.067414
C	3.176710	-3.580443	-1.630308	C	-2.507591	2.369711	-1.400836
H	4.611505	-4.540424	-2.914324	H	-2.816138	1.376563	-1.749899
H	4.077312	-6.187969	-4.648836	C	-3.033082	2.886824	-0.191039
H	2.402864	-7.558108	-5.902926	C	-2.646682	4.182850	0.229628
H	-0.039321	-7.384372	-5.322103	H	-3.021254	4.576971	1.185219
H	-0.792728	-5.913277	-3.481522	C	-1.724226	4.910483	-0.521549
C	-0.615327	-4.212201	-1.496526	C	-0.159872	6.447906	-1.392122
C	-1.282093	-2.994639	-1.664241	C	0.687063	7.521380	-1.691309
C	-2.711291	-2.874014	-1.665597	H	0.723846	8.407890	-1.037692
C	-3.427315	-4.007774	-1.273500	C	-1.133453	6.285696	-0.227483
C	-2.796002	-5.229661	-0.915378	C	-0.433759	6.370370	1.182597
C	-3.548560	-6.361383	-0.479246	C	-2.229883	7.415804	-0.177180
C	-2.918478	-7.548270	-0.136449	C	-1.321996	7.286137	2.040580
C	-1.503960	-7.654529	-0.225794	H	0.570419	6.829264	1.060355
C	-0.746025	-6.583921	-0.680287	H	-0.293035	5.370772	1.637322

C	-1.868459	8.298828	1.027002	H	1.508525	-2.429316	6.454250
H	-3.218354	6.941744	-0.005587	C	3.028195	0.375246	5.311391
H	-2.298158	7.965870	-1.136333	H	1.989119	-0.237156	3.516007
H	-2.157185	6.698310	2.480498	H	3.753614	-0.456026	3.370653
H	-0.772625	7.747076	2.886420	H	1.031260	-0.039948	6.017735
H	-2.729075	8.890665	1.400706	H	2.212124	0.066302	7.358273
H	-1.069627	9.022530	0.751654	H	2.753080	1.425461	5.091163
H	2.149876	8.308794	-3.098237	H	4.070228	0.367389	5.702576
C	6.270054	-3.553500	4.533199	H	7.301212	-3.386149	4.880505
C	5.974676	-4.679755	3.741792	C	9.123975	1.844243	0.779497
H	6.777325	-5.383916	3.474664	C	8.584906	1.302105	1.961403
C	4.666186	-4.909264	3.287027	H	8.938871	1.665350	2.938596
H	4.440255	-5.786392	2.661096	C	7.599622	0.302057	1.903283
C	3.656271	-3.992728	3.628360	H	7.174465	-0.123687	2.825758
C	2.232967	-3.944205	3.271077	C	7.161440	-0.145332	0.645337
C	1.439062	-4.797679	2.486821	C	6.140719	-1.132439	0.281485
H	1.868210	-5.690269	2.005357	C	5.254175	-1.879714	1.073390
C	0.087067	-4.486537	2.298823	H	5.308814	-1.853497	2.171455
H	-0.522225	-5.131351	1.649322	C	4.266092	-2.648499	0.445921
C	-0.502071	-3.336446	2.885394	H	3.542759	-3.203538	1.056876
C	0.313520	-2.478722	3.668734	C	4.189756	-2.727407	-0.964349
H	-0.112956	-1.575927	4.126335	C	5.116739	-2.004271	-1.752435
C	1.664455	-2.781006	3.850326	H	5.036406	-2.042907	-2.848427
C	3.952406	-2.855561	4.424980	C	6.064344	-1.191690	-1.131660
C	5.258194	-2.638091	4.881515	C	7.695220	0.407726	-0.549136
H	5.497015	-1.759102	5.501401	C	8.679251	1.400177	-0.481466
C	2.713924	-1.991273	4.626488	H	9.098040	1.840986	-1.400183
C	2.353752	-1.782717	6.146313	C	7.043154	-0.220531	-1.779020
C	2.900426	-0.526106	4.074438	C	6.332167	0.839547	-2.711903
C	2.072599	-0.281492	6.314145	C	8.075600	-0.927245	-2.730991
H	3.226552	-2.078390	6.764509	C	6.820817	0.549296	-4.141143

H	6.639162	1.859053	-2.399735
H	5.230381	0.796314	-2.620166
C	8.241660	0.010234	-3.935300
H	7.641432	-1.890399	-3.071902
H	9.022179	-1.165404	-2.207214
H	6.183990	-0.233524	-4.607815
H	6.769552	1.439540	-4.800812
H	8.660795	-0.501011	-4.826064
H	8.931702	0.846170	-3.684734
H	9.895327	2.627449	0.840700
C	-8.284831	2.689168	-4.609201
C	-7.124413	2.843461	-5.389132
H	-7.125008	3.546229	-6.236507
C	-5.968186	2.102665	-5.096545
H	-5.060305	2.216402	-5.708787
C	-5.989163	1.210638	-4.011599
C	-4.956396	0.300610	-3.507012
C	-3.651922	0.028563	-3.951561
H	-3.217014	0.570546	-4.805008
C	-2.896566	-0.958644	-3.305216
H	-1.884598	-1.168357	-3.671980
C	-3.422139	-1.685788	-2.205333
C	-4.719079	-1.361190	-1.734356
H	-5.122962	-1.889163	-0.858183
C	-5.479524	-0.390493	-2.385985
C	-7.154440	1.063377	-3.209014
C	-8.305335	1.800477	-3.516442
H	-9.223795	1.694042	-2.920710
C	-6.893843	0.077246	-2.070260
C	-7.006290	0.762202	-0.676897
C	-7.937313	-1.068520	-1.938263
C	-8.496372	0.705878	-0.295279
H	-6.583568	1.783964	-0.684077
H	-6.397559	0.176496	0.040540
C	-9.084924	-0.514854	-1.060052
H	-7.443350	-1.919904	-1.424863
H	-8.272248	-1.439816	-2.927126
H	-8.624699	0.630424	0.803763
H	-9.008404	1.640706	-0.601897
H	-9.466701	-1.294919	-0.369788
H	-9.950792	-0.209791	-1.682718
H	-9.187922	3.269362	-4.853975
Si	4.834946	5.025595	-0.737448
C	4.162823	4.234494	-2.287744
H	3.148347	4.598951	-2.541160
H	4.140250	3.128442	-2.243520
H	4.836925	4.521477	-3.123485
C	4.555791	6.875779	-0.665245
H	4.867067	7.294980	0.312302
H	5.141986	7.380680	-1.460717
H	3.484903	7.108798	-0.835842
C	6.574306	4.478168	-0.321017
H	7.276373	4.747629	-1.136920
H	6.931979	4.946100	0.617498
H	6.604832	3.377141	-0.190434
O	3.968667	4.473854	0.724340
C	3.073199	3.625057	1.074013
C	3.120957	3.156062	2.484504
H	2.293550	3.717838	2.989203
H	2.737378	2.115620	2.509026
C	4.463562	3.302424	3.184362
H	4.804239	4.356037	3.198559

H	5.232334	2.698495	2.659429
H	4.389264	2.946221	4.229071
C	1.875686	3.384375	0.222382
H	1.239607	4.292368	0.322532
H	2.121700	3.274147	-0.848864
H	1.270507	2.530193	0.570515
C	4.233127	1.547149	0.227098
N	3.601655	0.747990	-0.360744
H	2.871212	0.176176	-0.925256

TS2'-*S*

P	0.724267	-0.546899	-1.527606
N	-0.298635	-0.288094	-0.369513
P	-0.845061	-0.775139	1.053023
N	-1.405616	0.385214	2.007513
S	-0.700345	1.102080	3.258198
O	-1.436122	2.348137	3.585530
O	0.780877	1.146291	3.182150
C	-1.078088	-0.015804	4.749131
F	-0.519510	-1.229706	4.607617
F	-0.576483	0.563096	5.850419
F	-2.404006	-0.164632	4.898132
O	-2.084060	-1.792481	0.707757
C	-2.685788	-2.333153	1.854747
C	-2.019105	-3.366068	2.511363
C	-2.547158	-3.831572	3.772187
C	-1.870723	-4.781413	4.595316
C	-2.406950	-5.186938	5.809315
C	-3.646761	-4.664641	6.263425
C	-4.315459	-3.718678	5.502375
C	-3.783607	-3.267513	4.257579
C	-4.431457	-2.245949	3.508300
C	-3.900467	-1.748232	2.324596
H	-5.368571	-1.814069	3.892128
H	-5.265209	-3.286079	5.854055
H	-4.065171	-4.999010	7.224923
H	-1.861042	-5.915366	6.428014
H	-0.903918	-5.185383	4.266575
C	-0.733028	-3.902312	1.982004
C	0.375190	-3.062286	1.818441
C	1.710405	-3.555748	1.646858
C	1.856091	-4.928586	1.448230
C	0.748015	-5.818813	1.438566
C	0.915177	-7.216595	1.204673
C	-0.173773	-8.075197	1.200885
C	-1.479113	-7.566358	1.437411
C	-1.671070	-6.216976	1.701828
C	-0.569949	-5.308380	1.718216
H	-2.682756	-5.831176	1.893126
H	-2.343992	-8.246776	1.417477
H	-0.029968	-9.150042	1.011878
H	1.929566	-7.596736	1.013365
H	2.867502	-5.344907	1.326427
O	0.225275	-1.684834	1.919344
N	0.789088	0.784288	-2.528345
S	1.443366	0.867574	-4.100597
O	2.126073	2.158250	-4.237615
O	2.056947	-0.402244	-4.509187
C	-0.149058	1.000272	-5.139409
F	-0.740918	-0.206405	-5.194748
F	-1.002226	1.882119	-4.615083
F	0.193840	1.379167	-6.370503
O	2.164896	-0.961852	-0.879546

C	3.229214	-1.125751	-1.778885	C	-7.193403	5.384010	-0.694488
C	3.264735	-2.284441	-2.555213	C	-7.449849	4.390188	-1.658450
C	4.265586	-2.372757	-3.590237	H	-7.955769	4.660642	-2.598021
C	4.284831	-3.413823	-4.565060	C	-7.062445	3.059138	-1.432155
C	5.265409	-3.458254	-5.545874	H	-7.255696	2.281206	-2.187808
C	6.278174	-2.464300	-5.600834	C	-6.412109	2.738758	-0.228615
C	6.267074	-1.418544	-4.691673	C	-5.850868	1.470990	0.252152
C	5.257462	-1.329509	-3.686018	C	-5.802707	0.198262	-0.340006
C	5.191341	-0.210139	-2.813035	H	-6.274260	0.001828	-1.314657
C	4.170887	-0.054589	-1.876479	C	-5.138638	-0.836827	0.333827
H	5.958887	0.573912	-2.900629	H	-5.074534	-1.831895	-0.125866
H	7.026796	-0.622937	-4.743275	C	-4.557483	-0.625094	1.601784
H	7.056332	-2.514881	-6.377511	C	-4.617901	0.656317	2.197017
H	5.251820	-4.266956	-6.292290	H	-4.117170	0.826270	3.160978
H	3.500067	-4.181823	-4.545576	C	-5.251475	1.699587	1.517700
C	2.251850	-3.358961	-2.350981	C	-6.155720	3.737465	0.746260
C	0.879916	-3.078271	-2.371575	C	-6.547325	5.060654	0.514437
C	-0.143076	-4.077449	-2.251608	H	-6.349526	5.844891	1.263173
C	0.283455	-5.396692	-2.106842	C	-5.411140	3.156300	1.943135
C	1.655715	-5.751780	-2.036049	C	-4.034107	3.867343	2.228447
C	2.054824	-7.112144	-1.868732	C	-6.196130	3.307601	3.298363
C	3.391549	-7.453090	-1.735128	C	-4.180749	4.575072	3.584895
C	4.382941	-6.436321	-1.755669	H	-3.751598	4.544406	1.399529
C	4.028208	-5.108426	-1.950418	H	-3.237362	3.104119	2.319421
C	2.662879	-4.724891	-2.120319	C	-5.132696	3.649231	4.352117
H	4.810008	-4.337676	-1.977428	H	-6.789877	2.401661	3.530845
H	5.442749	-6.701124	-1.622338	H	-6.911953	4.151225	3.206322
H	3.685417	-8.505262	-1.600839	H	-3.204539	4.716098	4.091478
H	1.271305	-7.883781	-1.826275	H	-4.646115	5.578718	3.462711
H	-0.467731	-6.198204	-2.047735	H	-4.581920	2.735273	4.664201
O	0.449180	-1.763310	-2.579970	H	-5.562148	4.104472	5.267932

H	-7.500455	6.423352	-0.888252
C	8.214864	1.134473	4.199837
C	8.632930	0.486532	3.021662
H	9.656603	0.642495	2.647888
C	7.753617	-0.352718	2.318929
H	8.078981	-0.855161	1.394757
C	6.450037	-0.533401	2.812074
C	5.329765	-1.331292	2.303830
C	5.236171	-2.195555	1.200938
H	6.090498	-2.340604	0.522010
C	4.037504	-2.885133	0.978540
H	3.958582	-3.580814	0.132795
C	2.910130	-2.693005	1.810898
C	2.998279	-1.774723	2.889774
H	2.121696	-1.596816	3.527333
C	4.209468	-1.130096	3.153975
C	6.026517	0.119576	3.997894
C	6.909225	0.952982	4.693542
H	6.588346	1.467886	5.613521
C	4.576340	-0.213188	4.322075
C	4.403612	-0.944090	5.701574
C	3.649876	1.052501	4.456531
C	3.041690	-0.478085	6.234252
H	5.205046	-0.598427	6.388690
H	4.507120	-2.042996	5.603377
C	3.027128	1.011854	5.865951
H	2.844553	0.995232	3.703913
H	4.218109	1.983828	4.264311
H	2.212738	-1.005123	5.714648
H	2.915230	-0.669858	7.319587
H	2.011689	1.455163	5.879997
H	3.652362	1.578918	6.589844
H	8.915185	1.792051	4.737670
C	3.646324	7.728587	1.282941
C	3.286094	6.850723	2.323624
H	3.002960	7.257009	3.306943
C	3.288231	5.460879	2.117632
H	3.014232	4.770867	2.931418
C	3.650801	4.965444	0.852232
C	3.739020	3.592348	0.349911
C	3.492335	2.369992	0.993975
H	3.178080	2.337692	2.045874
C	3.643565	1.176481	0.279857
H	3.469673	0.221422	0.791245
C	4.024796	1.192668	-1.084706
C	4.259774	2.435453	-1.726164
H	4.489317	2.440271	-2.799426
C	4.130698	3.630909	-1.014373
C	4.007710	5.847923	-0.200610
C	4.010513	7.230512	0.017206
H	4.284257	7.926858	-0.791442
C	4.345219	5.075878	-1.468855
C	3.499534	5.482061	-2.713078
C	5.791135	5.333093	-1.990344
C	4.311118	5.012676	-3.941747
H	3.409413	6.589165	-2.709982
H	2.471770	5.071994	-2.686517
C	5.781610	4.856849	-3.456410
H	6.558759	4.839645	-1.360932
H	5.980019	6.426676	-1.940776
H	3.907206	4.054816	-4.326168
H	4.221872	5.739015	-4.774434

H	6.104416	3.797025	-3.516625	H	-5.140120	0.252088	-6.454122
H	6.501434	5.431830	-4.073503	H	-3.585327	1.863408	-4.625746
H	3.642635	8.815053	1.461010	H	-5.246031	1.309659	-4.342093
C	-8.076517	-1.445571	-3.386674	H	-9.004629	-0.946252	-3.704552
C	-8.130270	-2.469060	-2.422948	C	0.054105	3.390580	-2.162888
H	-9.098588	-2.759617	-1.987851	N	0.158279	4.361474	-2.829730
C	-6.957329	-3.126956	-2.019354	H	0.457286	1.843020	-2.189149
H	-6.999324	-3.933354	-1.270866	C	-1.139639	3.522941	-0.238960
C	-5.729963	-2.737104	-2.581576	O	-1.558220	4.751022	-0.206159
C	-4.369749	-3.235046	-2.345966	C	0.020975	3.135818	0.617293
C	-3.867574	-4.215662	-1.472593	H	0.884795	3.807206	0.461246
H	-4.537926	-4.774059	-0.800831	H	-0.304848	3.232023	1.674874
C	-2.488369	-4.459334	-1.443655	H	0.336530	2.096924	0.436374
H	-2.086625	-5.194489	-0.731523	C	-2.192030	2.537559	-0.655303
C	-1.592266	-3.752390	-2.285572	H	-1.713057	1.601450	-0.992776
C	-2.113942	-2.770098	-3.162421	H	-2.690850	2.263307	0.307727
H	-1.441449	-2.216495	-3.829375	C	-3.208221	3.080648	-1.652245
C	-3.482377	-2.507845	-3.176108	H	-2.708298	3.332163	-2.609191
C	-5.666924	-1.686250	-3.538964	H	-3.994719	2.330057	-1.842848
C	-6.846859	-1.051748	-3.950772	H	-3.704626	3.993465	-1.272679
H	-6.827250	-0.254836	-4.708472	Si	-0.986463	6.308386	0.420592
C	-4.216842	-1.417824	-3.938424	C	0.412570	6.863044	-0.680674
C	-3.967630	-1.349621	-5.471020	H	0.639271	7.931361	-0.484875
C	-3.736363	-0.005216	-3.477948	H	1.336406	6.284946	-0.487378
C	-4.195559	0.128929	-5.885797	H	0.150556	6.729130	-1.748952
H	-4.606042	-2.068065	-6.022114	C	-0.517648	6.084788	2.224087
H	-2.915031	-1.639650	-5.662289	H	0.480082	5.617445	2.331634
C	-4.219124	0.957416	-4.571207	H	-0.471821	7.082513	2.708994
H	-2.628563	-0.017567	-3.436702	H	-1.261632	5.470994	2.770687
H	-4.093271	0.237683	-2.459108	C	-2.550247	7.322048	0.209624
H	-3.385197	0.469115	-6.561965	H	-2.382425	8.371905	0.526139

H	-2.866983	7.325895	-0.852773
H	-3.381407	6.906998	0.815108

TS2'-*R*

P	0.556000	-0.983729	-0.839065
N	-0.048827	-0.565114	0.535809
P	-1.228662	0.089471	1.377455
N	-0.987583	1.574833	1.913910
S	-0.655044	2.129082	3.386879
O	-0.032074	3.469849	3.272084
O	-0.053819	1.126628	4.299121
C	-2.343202	2.487696	4.172042
F	-2.152558	2.819059	5.453938
F	-2.958768	3.509304	3.548757
F	-3.127387	1.402596	4.109562
O	-2.555851	0.021674	0.406717
C	-3.728137	0.453697	1.033883
C	-4.369820	-0.429153	1.907904
C	-5.499515	0.062930	2.656745
C	-6.160692	-0.707632	3.658568
C	-7.254096	-0.200512	4.347099
C	-7.737649	1.106149	4.072107
C	-7.092931	1.896602	3.133212
C	-5.956154	1.412588	2.418868
C	-5.265006	2.242437	1.493404
C	-4.144811	1.800667	0.795603
H	-5.628483	3.268265	1.327921
H	-7.442731	2.920635	2.929016
H	-8.611221	1.494684	4.617216
H	-7.745054	-0.814732	5.117149
H	-5.788926	-1.715315	3.889275
C	-3.832204	-1.814102	2.064588
C	-2.481792	-2.003051	2.390801
C	-1.862000	-3.290320	2.513530
C	-2.688615	-4.400931	2.344069
C	-4.063159	-4.285134	2.006289
C	-4.871453	-5.445662	1.806469
C	-6.190202	-5.338186	1.392231
C	-6.753408	-4.054324	1.163138
C	-6.003538	-2.906634	1.381691
C	-4.647654	-2.979187	1.824931
H	-6.458875	-1.923446	1.206072
H	-7.793953	-3.964314	0.815057
H	-6.798609	-6.241748	1.234818
H	-4.414544	-6.433279	1.973377
H	-2.261623	-5.408291	2.460598
O	-1.691872	-0.880581	2.622367
N	1.335788	0.249678	-1.661325
S	1.139895	0.839415	-3.223165
O	-0.212215	0.611565	-3.752917
O	1.761688	2.172680	-3.251331
C	2.295852	-0.291996	-4.221566
F	1.786920	-1.529048	-4.263389
F	2.393562	0.195935	-5.460510
F	3.503203	-0.320120	-3.649951
O	1.603506	-2.174406	-0.454060
C	2.127484	-3.026078	-1.432371
C	1.250147	-3.889861	-2.089994
C	1.750763	-4.680429	-3.188377
C	0.909173	-5.472502	-4.025667
C	1.436829	-6.207030	-5.078571
C	2.832012	-6.191374	-5.343963
C	3.674181	-5.417206	-4.561737

C	3.161605	-4.635432	-3.482586	C	-2.008472	3.043269	-2.113645
C	4.013113	-3.815389	-2.696642	H	-1.615756	2.654984	-3.064889
C	3.538884	-2.988434	-1.677530	C	-2.849722	2.237883	-1.335357
H	5.094925	-3.840011	-2.896658	H	-3.115004	1.237003	-1.699511
H	4.755525	-5.382264	-4.767529	C	-3.349640	2.688768	-0.088377
H	3.239099	-6.784103	-6.177184	C	-3.014776	3.988106	0.359807
H	0.765612	-6.802291	-5.716086	H	-3.369715	4.329445	1.342386
H	-0.173604	-5.486970	-3.841063	C	-2.165906	4.788058	-0.403721
C	-0.193504	-3.966670	-1.723349	C	-0.726016	6.437274	-1.277893
C	-1.012858	-2.841899	-1.824928	C	0.056230	7.561874	-1.565135
C	-2.445335	-2.896028	-1.817916	H	0.098048	8.412577	-0.866011
C	-3.011760	-4.123749	-1.469911	C	-1.617769	6.169731	-0.068537
C	-2.227527	-5.272974	-1.177288	C	-0.835949	6.206836	1.302579
C	-2.830584	-6.502991	-0.775798	C	-2.752072	7.247210	0.112414
C	-2.056732	-7.620634	-0.502094	C	-1.696116	7.049112	2.258113
C	-0.642979	-7.554953	-0.630005	H	0.146494	6.699115	1.145023
C	-0.027613	-6.382441	-1.046611	H	-0.636656	5.193526	1.702364
C	-0.795867	-5.214380	-1.331352	C	-2.342571	8.086247	1.332131
H	1.066715	-6.337085	-1.140465	H	-3.707050	6.724220	0.327066
H	-0.030755	-8.439479	-0.397822	H	-2.907817	7.838371	-0.811373
H	-2.534728	-8.559800	-0.184160	H	-2.479068	6.411338	2.723634
H	-3.925798	-6.539134	-0.677177	H	-1.106473	7.490279	3.086962
H	-4.107490	-4.222501	-1.457070	H	-3.196236	8.628033	1.788526
O	-0.397896	-1.595075	-2.016216	H	-1.589097	8.850723	1.040215
C	0.791354	7.600482	-2.766820	H	1.404887	8.483679	-3.002565
C	0.753650	6.516996	-3.664248	C	6.355484	-3.211959	4.424806
H	1.342243	6.557936	-4.593539	C	6.171864	-4.252754	3.494936
C	-0.018328	5.377691	-3.378974	H	7.041254	-4.828728	3.143107
H	-0.023232	4.518051	-4.066039	C	4.890690	-4.556139	3.007543
C	-0.764528	5.346671	-2.188416	H	4.750748	-5.363157	2.271864
C	-1.653648	4.317438	-1.638498	C	3.795370	-3.799345	3.457888

C	2.373121	-3.850980	3.098044	C	7.351692	0.334890	1.029860
C	1.664125	-4.675548	2.208633	C	6.327378	-0.600195	0.558777
H	2.174958	-5.460900	1.629830	C	5.266522	-1.225098	1.230325
C	0.288636	-4.471559	2.044122	H	5.152440	-1.134404	2.320128
H	-0.260182	-5.093088	1.323183	C	4.338176	-1.974139	0.497226
C	-0.403510	-3.454020	2.748687	H	3.504240	-2.454602	1.024716
C	0.323334	-2.626852	3.641915	C	4.475939	-2.140112	-0.903332
H	-0.186523	-1.823916	4.191818	C	5.576348	-1.534995	-1.562136
C	1.694295	-2.828142	3.808898	H	5.674072	-1.625888	-2.655236
C	3.978455	-2.746789	4.393055	C	6.483987	-0.765105	-0.839571
C	5.258396	-2.456266	4.880808	C	8.128215	0.745820	-0.086033
H	5.411969	-1.641432	5.606202	C	9.149283	1.687134	0.082787
C	2.662131	-2.032487	4.674974	H	9.753380	2.022826	-0.775025
C	2.280159	-1.986416	6.201372	C	7.682619	0.021434	-1.354158
C	2.708772	-0.518220	4.251931	C	7.366419	0.977325	-2.564659
C	1.887612	-0.528310	6.498409	C	8.810584	-0.930335	-1.910901
H	3.168724	-2.272520	6.801281	C	8.156940	0.430920	-3.766073
H	1.482930	-2.717215	6.441990	H	7.711433	2.000979	-2.312251
C	2.785498	0.284292	5.556936	H	6.276936	1.054742	-2.732309
H	1.763189	-0.259519	3.738855	C	9.398961	-0.200234	-3.124697
H	3.535763	-0.318122	3.544920	H	8.340922	-1.879435	-2.244866
H	0.828825	-0.342496	6.221916	H	9.552645	-1.190737	-1.130715
H	2.004130	-0.262608	7.569023	H	7.570667	-0.358263	-4.287360
H	2.437141	1.327814	5.423115	H	8.390309	1.209926	-4.520503
H	3.828289	0.316422	5.944785	H	9.963084	-0.873370	-3.802731
H	7.366931	-2.983382	4.794338	H	10.102816	0.595632	-2.795226
C	9.394810	2.214145	1.366023	H	10.192993	2.959715	1.505019
C	8.627472	1.800152	2.471005	C	-8.620231	2.091548	-4.588144
H	8.832056	2.223256	3.466615	C	-7.497769	2.364625	-5.390948
C	7.600214	0.855575	2.311108	H	-7.587109	3.065412	-6.235166
H	6.994096	0.533531	3.172325	C	-6.267085	1.743105	-5.126183

H	-5.389257	1.947901	-5.758257
C	-6.175539	0.851350	-4.044638
C	-5.048241	0.044616	-3.567590
C	-3.735572	-0.096427	-4.046607
H	-3.378830	0.485475	-4.909865
C	-2.873672	-1.009015	-3.425554
H	-1.858728	-1.128187	-3.823218
C	-3.296911	-1.785949	-2.316969
C	-4.608292	-1.597480	-1.812866
H	-4.938531	-2.169463	-0.933162
C	-5.475419	-0.701278	-2.440014
C	-7.302073	0.586760	-3.218059
C	-8.528270	1.203115	-3.499160
H	-9.419311	1.001988	-2.886446
C	-6.919181	-0.367432	-2.087262
C	-7.050647	0.317484	-0.694736
C	-7.853762	-1.599383	-1.922110
C	-8.518418	0.134918	-0.271421
H	-6.719540	1.372395	-0.721146
H	-6.374095	-0.206931	0.008742
C	-9.011454	-1.149549	-0.996961
H	-7.270658	-2.409106	-1.436571
H	-8.194536	-1.991317	-2.900886
H	-8.612218	0.071683	0.831935
H	-9.120689	1.012924	-0.581901
H	-9.279487	-1.953514	-0.280743
H	-9.932422	-0.945608	-1.580481
H	-9.582358	2.577367	-4.812442
Si	4.255842	5.114812	-0.772267
C	3.706600	4.669642	-2.500801
H	2.637539	4.897956	-2.673058
H	3.880108	3.598593	-2.715639
H	4.301482	5.276192	-3.216558
C	3.741386	6.850970	-0.276695
H	3.962070	7.049938	0.791109
H	4.285168	7.600862	-0.887874
H	2.655286	6.994193	-0.453230
C	6.058822	4.716428	-0.455061
H	6.717887	5.274987	-1.151280
H	6.353632	4.964214	0.584112
H	6.217866	3.629384	-0.613587
O	3.456872	4.122478	0.445082
C	2.598100	3.162391	0.607312
C	2.724770	2.429206	1.913002
H	2.049556	3.004336	2.594033
H	2.243247	1.436517	1.817007
C	4.136936	2.349859	2.478255
H	4.594585	3.354881	2.551097
H	4.786140	1.732269	1.826370
H	4.123061	1.905944	3.490921
C	1.266036	3.273429	-0.062202
H	0.745195	4.125903	0.423819
H	1.354788	3.493314	-1.140587
H	0.644365	2.379399	0.104633
C	3.483965	1.577791	-0.665568
N	4.628423	1.628426	-0.954415
H	2.184718	0.815450	-1.123045

TS3

P	1.204293	0.828084	1.042920
N	0.675947	0.552820	-0.420117
P	-0.287558	0.553511	-1.640622

N	-0.428089	-0.968161	-2.283645
S	-0.246482	-1.517337	-3.843708
O	0.201748	-2.919993	-3.739123
O	0.433453	-0.558967	-4.730872
C	-2.033742	-1.610570	-4.473658
F	-2.807035	-2.250831	-3.588594
F	-2.502346	-0.374135	-4.670925
F	-2.031576	-2.278919	-5.630465
O	-1.715787	1.179898	-1.153661
C	-2.716169	1.460162	-2.087756
C	-2.506381	2.515471	-2.985214
C	-3.476188	2.726966	-4.029329
C	-3.269143	3.637054	-5.108172
C	-4.240084	3.819734	-6.082913
C	-5.467633	3.106802	-6.021794
C	-5.688893	2.197462	-4.999571
C	-4.700711	1.968220	-3.994195
C	-4.897682	1.003388	-2.972235
C	-3.917950	0.679661	-2.028484
H	-5.866231	0.483889	-2.935118
H	-6.627288	1.622694	-4.954680
H	-6.234050	3.267524	-6.795168
H	-4.054585	4.518769	-6.912454
H	-2.320882	4.189212	-5.168898
C	-1.323748	3.413773	-2.835418
C	-0.032615	2.884870	-2.781531
C	1.158386	3.665968	-2.627456
C	0.986156	5.046388	-2.539469
C	-0.295775	5.657880	-2.560544
C	-0.431496	7.073487	-2.436542
C	-1.681617	7.669523	-2.384343
C	-2.849381	6.864258	-2.451202
C	-2.750960	5.487908	-2.603846
C	-1.479418	4.842310	-2.683549
H	-3.666305	4.883537	-2.654753
H	-3.842775	7.332877	-2.383186
H	-1.772020	8.761606	-2.280629
H	0.483785	7.679877	-2.361124
H	1.873138	5.691175	-2.449714
O	0.109609	1.497140	-2.904550
N	1.430388	-0.451535	1.995795
S	0.353092	-1.080817	2.988675
O	-1.060588	-0.810371	2.618302
O	0.728692	-2.484809	3.313695
C	0.661126	-0.184964	4.642510
F	-0.064218	-0.790751	5.596131
F	1.962354	-0.264311	4.960505
F	0.306216	1.106298	4.583622
O	2.659065	1.535733	0.818620
C	3.261788	1.955832	2.010692
C	2.807909	3.145915	2.572315
C	3.334088	3.536537	3.858171
C	2.879238	4.688191	4.567088
C	3.400866	5.014209	5.811476
C	4.401018	4.204045	6.410631
C	4.848205	3.064946	5.759292
C	4.326813	2.693119	4.484013
C	4.753916	1.498582	3.836311
C	4.240718	1.103247	2.606269
H	5.510487	0.866671	4.326827
H	5.610737	2.419107	6.222247
H	4.810052	4.474966	7.395825

H	3.030616	5.906016	6.339676
H	2.097452	5.317772	4.120930
C	1.708242	3.911330	1.913031
C	0.461513	3.305053	1.717036
C	-0.727644	4.037712	1.395695
C	-0.569400	5.382594	1.065189
C	0.702867	6.020355	1.075984
C	0.857063	7.385243	0.688830
C	2.102746	7.995529	0.700831
C	3.249385	7.262464	1.109478
C	3.130052	5.941739	1.520785
C	1.862143	5.286606	1.521873
H	4.019293	5.378891	1.840413
H	4.238359	7.745476	1.103550
H	2.206415	9.047179	0.392600
H	-0.037045	7.940887	0.368548
H	-1.459157	5.978051	0.808437
O	0.333179	1.933381	1.907875
C	-6.909561	-5.627481	2.669673
C	-6.038388	-4.902714	3.505286
H	-5.909577	-5.199278	4.557459
C	-5.322306	-3.801093	3.010021
H	-4.642230	-3.245142	3.671298
C	-5.491028	-3.437918	1.663175
C	-4.905163	-2.344208	0.883606
C	-3.956492	-1.367912	1.215675
H	-3.490248	-1.340529	2.208850
C	-3.594086	-0.405687	0.263727
H	-2.884872	0.371391	0.564453
C	-4.163707	-0.398389	-1.036342
C	-5.074199	-1.438096	-1.373520

H	-5.474533	-1.501148	-2.396533
C	-5.444746	-2.388061	-0.427067
C	-6.367199	-4.166833	0.817662
C	-7.076947	-5.263373	1.319197
H	-7.759737	-5.837912	0.672267
C	-6.386131	-3.575998	-0.588916
C	-5.881912	-4.593688	-1.681945
C	-7.823463	-3.166441	-1.084002
C	-6.824932	-4.415726	-2.880643
H	-5.977765	-5.624681	-1.282858
H	-4.812132	-4.436133	-1.923921
C	-8.179599	-4.148966	-2.211336
H	-7.785430	-2.132013	-1.484023
H	-8.553445	-3.159929	-0.250829
H	-6.522339	-3.531430	-3.484431
H	-6.827125	-5.289656	-3.563782
H	-8.952255	-3.754854	-2.902850
H	-8.575584	-5.097149	-1.785629
H	-7.464443	-6.488176	3.074410
C	8.318177	-0.764241	-2.949853
C	8.544007	0.280482	-2.033377
H	9.502727	0.335799	-1.495776
C	7.553539	1.246751	-1.793773
H	7.729033	2.054976	-1.067183
C	6.329869	1.151216	-2.479151
C	5.104372	1.955018	-2.387407
C	4.767189	3.058867	-1.585171
H	5.492271	3.487411	-0.875800
C	3.474526	3.592770	-1.667448
H	3.191692	4.420828	-0.999896
C	2.508936	3.054531	-2.555058

C	2.863989	1.946369	-3.365133
H	2.131721	1.512517	-4.058857
C	4.142654	1.400044	-3.269397
C	6.103855	0.102617	-3.409995
C	7.097306	-0.854547	-3.646495
H	6.927035	-1.675430	-4.361755
C	4.705503	0.190468	-4.005890
C	4.720892	0.374220	-5.568575
C	3.838769	-1.109538	-3.785458
C	3.556756	-0.475466	-6.094592
H	5.679982	-0.021250	-5.963790
H	4.668413	1.443194	-5.854957
C	3.587842	-1.706725	-5.181500
H	2.871938	-0.822441	-3.326182
H	4.332627	-1.810921	-3.084941
H	2.588622	0.050503	-5.960551
H	3.654518	-0.718480	-7.172374
H	2.651526	-2.297567	-5.223185
H	4.424520	-2.379118	-5.473165
H	9.102172	-1.517654	-3.121744
C	6.130135	-6.641884	-0.100195
C	6.485246	-5.756142	-1.135087
H	6.913456	-6.152341	-2.068766
C	6.299625	-4.371169	-0.984942
H	6.583348	-3.674237	-1.789640
C	5.750567	-3.888739	0.215986
C	5.436947	-2.523116	0.647552
C	5.571322	-1.298816	-0.024854
H	5.986902	-1.251304	-1.041499
C	5.166644	-0.125545	0.623060
H	5.248472	0.837650	0.102382
C	4.652954	-0.158880	1.938972
C	4.513729	-1.399048	2.605644
H	4.045541	-1.421320	3.600654
C	4.896688	-2.576955	1.958278
C	5.392718	-4.779155	1.262187
C	5.582952	-6.156656	1.104127
H	5.306006	-6.858404	1.907366
C	4.807848	-4.021434	2.448887
C	3.328157	-4.450565	2.801526
C	5.593108	-4.254401	3.785877
C	3.352023	-4.957769	4.256061
H	2.948599	-5.212201	2.091541
H	2.655390	-3.576243	2.721038
C	4.528451	-4.203423	4.889654
H	6.418946	-3.526223	3.910736
H	6.050049	-5.266586	3.762643
H	2.386186	-4.786733	4.772840
H	3.555055	-6.050826	4.287422
H	4.235655	-3.151878	5.099741
H	4.876452	-4.642039	5.847253
H	6.280410	-7.724356	-0.233108
C	-7.603931	-0.520839	2.883581
C	-7.785197	0.070366	1.619671
H	-8.626253	-0.248229	0.984601
C	-6.886027	1.042626	1.151527
H	-7.007007	1.480322	0.148361
C	-5.799169	1.406543	1.963340
C	-4.639210	2.258672	1.681058
C	-4.307041	3.031024	0.556395
H	-5.015610	3.149240	-0.277732
C	-3.036073	3.617970	0.485101

H	-2.755805	4.205204	-0.398981
C	-2.074606	3.416383	1.501136
C	-2.436454	2.670787	2.653892
H	-1.703336	2.519370	3.460005
C	-3.717292	2.125542	2.753303
C	-5.612553	0.810208	3.239000
C	-6.519369	-0.149330	3.701501
H	-6.373499	-0.641733	4.675532
C	-4.337437	1.331245	3.900048
C	-4.665586	2.283059	5.119111
C	-3.437141	0.216216	4.534663
C	-4.413297	1.434766	6.371291
H	-5.687094	2.706018	5.048311
H	-3.954232	3.135540	5.102768
C	-3.159855	0.638315	5.988125
H	-2.518242	0.049794	3.946877
H	-3.986075	-0.746640	4.528925
H	-4.296362	2.047101	7.289139
H	-5.264922	0.741115	6.545295
H	-2.265994	1.298358	6.036092
H	-2.957178	-0.231397	6.644675
H	-8.299768	-1.301756	3.226156
Si	1.760597	-5.723714	-2.196015
C	3.131004	-4.733416	-3.007653
H	3.865463	-4.347870	-2.274400
H	2.699146	-3.889882	-3.579285
H	3.678402	-5.387988	-3.717877
C	2.416822	-6.944910	-0.927973
H	3.037818	-7.721335	-1.420642
H	3.062472	-6.433493	-0.185078
H	1.587993	-7.449263	-0.391709
C	0.626965	-6.505576	-3.468956
H	0.210535	-5.711154	-4.121153
H	1.171537	-7.234754	-4.103043
H	-0.216812	-7.031005	-2.978171
O	0.652650	-4.700889	-1.327391
C	0.685475	-3.559072	-0.661888
C	-0.526356	-2.928287	-0.379232
H	-0.499255	-2.170068	0.422790
H	-0.379846	-1.906361	-1.442236
C	-1.854016	-3.589232	-0.661558
H	-2.193522	-4.202479	0.200144
H	-1.784336	-4.249379	-1.547284
H	-2.641361	-2.834697	-0.844851
C	2.014497	-2.946823	-0.339379
H	1.930617	-2.179883	0.450627
H	2.734672	-3.719947	-0.009307
H	2.443299	-2.466545	-1.244811
C	-1.815708	-3.303055	4.825862
N	-2.837156	-3.329415	5.391813
H	-0.863443	-3.226008	4.280532

TS4

P	0.562776	0.275946	-1.198535
N	-0.436971	0.121463	0.003975
P	-0.896863	-0.452348	1.387861
N	-1.124188	0.801222	2.447980
S	-1.461535	0.730039	4.092383
O	-1.661020	-0.658276	4.533847
O	-2.406887	1.813353	4.400870
C	0.203338	1.319673	4.802600
F	0.109967	1.340775	6.133145
F	1.191033	0.490095	4.434994

F	0.470676	2.547087	4.345296	H	2.699068	-4.878327	0.680642
O	-2.243200	-1.307770	1.060763	O	0.079407	-1.463316	2.219401
C	-2.835898	-2.118160	2.038614	N	0.516965	1.805376	-1.784483
C	-2.136514	-3.234553	2.496110	S	0.895830	2.383694	-3.300726
C	-2.699782	-3.989904	3.588398	O	1.393463	3.759938	-3.136466
C	-1.989183	-5.030474	4.258221	O	1.573804	1.397238	-4.154624
C	-2.568094	-5.728442	5.308792	C	-0.852823	2.584491	-4.014601
C	-3.887192	-5.425428	5.738963	F	-1.392623	1.373009	-4.238213
C	-4.597681	-4.408305	5.121076	F	-1.628847	3.256672	-3.153126
C	-4.026636	-3.661439	4.047759	F	-0.782119	3.252062	-5.167211
C	-4.739812	-2.601650	3.423757	O	2.039416	-0.196490	-0.677906
C	-4.169541	-1.799310	2.439530	C	3.039644	-0.015804	-1.647086
H	-5.768140	-2.384507	3.751057	C	3.122578	-0.949068	-2.680503
H	-5.614014	-4.150129	5.457224	C	4.050297	-0.685329	-3.750684
H	-4.336606	-5.989506	6.570272	C	4.148726	-1.509647	-4.909965
H	-1.995411	-6.518747	5.817588	C	5.064414	-1.225621	-5.913368
H	-0.962953	-5.268158	3.945221	C	5.923910	-0.100015	-5.808538
C	-0.850973	-3.655169	1.868771	C	5.827821	0.742662	-4.712090
C	0.250381	-2.798996	1.826752	C	4.886745	0.489114	-3.669315
C	1.574797	-3.216932	1.470042	C	4.759721	1.378757	-2.566685
C	1.700815	-4.508259	0.958291	C	3.842734	1.160542	-1.542360
C	0.592221	-5.387861	0.827910	H	5.411189	2.264877	-2.520259
C	0.744059	-6.690023	0.262458	H	6.470775	1.633060	-4.632677
C	-0.340223	-7.545031	0.139629	H	6.651791	0.111085	-6.606576
C	-1.623543	-7.129761	0.586209	H	5.119157	-1.874591	-6.800563
C	-1.798508	-5.880316	1.165289	H	3.480908	-2.376244	-5.009211
C	-0.703129	-4.976682	1.308394	C	2.227796	-2.143727	-2.658160
H	-2.796798	-5.565858	1.498636	C	0.840006	-1.987915	-2.549213
H	-2.486842	-7.802775	0.472662	C	-0.095694	-3.075799	-2.524517
H	-0.210614	-8.543701	-0.304716	C	0.449315	-4.356495	-2.634554
H	1.743079	-6.995941	-0.083383	C	1.846938	-4.594836	-2.699827

C	2.364991	-5.925173	-2.749506
C	3.730698	-6.162414	-2.734560
C	4.635531	-5.068832	-2.674633
C	4.163879	-3.763285	-2.659900
C	2.763971	-3.482489	-2.684969
H	4.877905	-2.930383	-2.621328
H	5.720329	-5.253599	-2.644368
H	4.115393	-7.193135	-2.763869
H	1.650571	-6.762051	-2.783093
H	-0.225601	-5.224480	-2.656497
O	0.315514	-0.692162	-2.485427
C	-7.849164	4.755712	-1.368040
C	-8.651524	3.628755	-1.628969
H	-9.536089	3.726843	-2.277031
C	-8.330536	2.382150	-1.067349
H	-8.949377	1.495377	-1.273059
C	-7.192890	2.281141	-0.248526
C	-6.596317	1.121525	0.421098
C	-6.997022	-0.221546	0.468933
H	-7.911083	-0.559990	-0.039117
C	-6.186098	-1.144986	1.140067
H	-6.468613	-2.208284	1.152126
C	-4.978692	-0.748914	1.762989
C	-4.596699	0.612958	1.728766
H	-3.685949	0.943552	2.243523
C	-5.403696	1.539874	1.065374
C	-6.380053	3.414496	0.015345
C	-6.713546	4.654751	-0.541663
H	-6.097066	5.545260	-0.344472
C	-5.191943	3.040509	0.899445
C	-3.815271	3.414574	0.248284
C	-5.180912	3.824431	2.274662
C	-3.394126	4.728599	0.916846
H	-3.874044	3.463028	-0.856219
H	-3.076376	2.624222	0.495075
C	-3.798259	4.497338	2.377160
H	-5.391497	3.146281	3.124352
H	-5.987293	4.585530	2.260048
H	-2.317930	4.951549	0.780977
H	-3.962736	5.582791	0.488343
H	-3.074479	3.807130	2.860259
H	-3.819109	5.423932	2.986177
H	-8.112135	5.727067	-1.814573
C	9.297272	-0.074647	2.596626
C	8.532949	0.336828	3.703663
H	9.009902	0.906388	4.515908
C	7.166835	0.023123	3.778855
H	6.567236	0.340998	4.645740
C	6.578776	-0.704578	2.731013
C	5.204746	-1.185107	2.560208
C	4.072810	-1.081194	3.386026
H	4.118740	-0.546595	4.347183
C	2.877388	-1.692777	2.987671
H	2.005599	-1.645870	3.652121
C	2.784014	-2.405807	1.765701
C	3.921179	-2.466881	0.922490
H	3.850594	-2.990250	-0.043108
C	5.119284	-1.871389	1.322209
C	7.343490	-1.112163	1.603691
C	8.707614	-0.798659	1.542404
H	9.323957	-1.108731	0.686114
C	6.457344	-1.842475	0.594468

C	6.397517	-1.096603	-0.770947	C	3.037495	5.880876	0.305589
C	6.979547	-3.243625	0.162984	C	1.828378	6.276426	-0.628422
C	7.605766	-1.597663	-1.584686	C	4.260458	6.663811	-0.305060
H	6.370968	0.001395	-0.640132	C	2.454003	6.933020	-1.866768
H	5.449542	-1.376662	-1.270938	H	1.191492	7.010011	-0.092190
C	7.965423	-2.994811	-1.001378	H	1.191193	5.404759	-0.866739
H	6.111049	-3.834913	-0.194819	C	3.661925	7.680827	-1.289934
H	7.423168	-3.800890	1.011841	H	4.903143	5.946836	-0.856821
H	7.367902	-1.632202	-2.667667	H	4.890084	7.119437	0.484425
H	8.461525	-0.899683	-1.481897	H	2.786797	6.152368	-2.584384
H	7.892140	-3.798417	-1.762844	H	1.741325	7.586580	-2.410128
H	9.014431	-3.013693	-0.641754	H	4.390831	8.018667	-2.054988
H	10.368862	0.173451	2.551545	H	3.316228	8.587445	-0.745758
C	2.267114	7.507196	3.749579	H	2.049200	8.477382	4.222211
C	2.307902	6.341523	4.537743	C	-8.333924	-1.284269	-2.720805
H	2.117960	6.408306	5.620056	C	-8.209951	-2.519760	-2.057900
C	2.584279	5.094926	3.953712	H	-9.113543	-3.071248	-1.755721
H	2.604222	4.182658	4.568642	C	-6.940080	-3.056216	-1.789032
C	2.821611	5.031904	2.569873	H	-6.840062	-4.028479	-1.281729
C	3.132586	3.895198	1.696732	C	-5.800236	-2.341591	-2.194639
C	3.268775	2.523594	1.971077	C	-4.377249	-2.686171	-2.110178
H	3.161304	2.133121	2.994351	C	-3.707714	-3.790313	-1.555342
C	3.521374	1.639441	0.915411	H	-4.257970	-4.579399	-1.019584
H	3.621969	0.566373	1.123761	C	-2.316569	-3.878499	-1.685624
C	3.647966	2.107228	-0.416237	H	-1.796509	-4.731904	-1.230114
C	3.534643	3.493721	-0.678530	C	-1.563006	-2.888808	-2.369865
H	3.580658	3.852286	-1.717732	C	-2.252211	-1.771316	-2.905581
C	3.270779	4.376329	0.369860	H	-1.703245	-0.994429	-3.450330
C	2.771428	6.204694	1.771686	C	-3.635028	-1.669969	-2.760384
C	2.494346	7.442806	2.361586	C	-5.921236	-1.087928	-2.856119
H	2.448944	8.359041	1.751324	C	-7.192249	-0.561434	-3.118982

H	-7.311666	0.401127	-3.636743
C	-4.540828	-0.529332	-3.195633
C	-4.388658	-0.061033	-4.670708
C	-4.216534	0.779306	-2.406374
C	-4.819401	1.428270	-4.704782
H	-4.964270	-0.704982	-5.364889
H	-3.321915	-0.153677	-4.955487
C	-4.853901	1.910977	-3.227964
H	-3.113951	0.893740	-2.373132
H	-4.568466	0.726992	-1.358820
H	-4.105964	2.021908	-5.312030
H	-5.810081	1.553954	-5.187865
H	-4.316152	2.869063	-3.084863
H	-5.894933	2.089276	-2.889945
H	-9.334906	-0.878248	-2.934829
C	-0.444877	3.055712	1.226728
N	-0.037814	3.322950	0.154956
H	0.267744	2.728154	-0.877207
H	-0.904866	1.902113	2.039768

53*Z*

Si	-1.256041	-0.181299	0.025209
C	-1.222265	-0.146863	1.911430
H	-0.604418	-0.979375	2.305426
H	-0.794406	0.802315	2.293748
H	-2.245551	-0.248871	2.328577
C	-1.931683	-1.807735	-0.631412
H	-1.863564	-1.839182	-1.737892
H	-2.996030	-1.941992	-0.348591
H	-1.355893	-2.666096	-0.229961
C	-2.251366	1.265820	-0.667614
H	-2.167566	1.300813	-1.773172
H	-1.906160	2.240271	-0.267789
H	-3.326002	1.155804	-0.411483
O	0.344863	-0.142452	-0.565953
C	1.426446	0.606423	-0.173420
C	2.623137	0.007864	0.044024
H	3.468428	0.665380	0.304489
C	2.879421	-1.464377	-0.061583
H	3.250923	-1.887920	0.897750
H	1.957064	-2.006937	-0.345696
C	1.201552	2.091486	-0.069542
H	0.784153	2.493548	-1.017520
H	2.143349	2.626314	0.155493
H	0.471749	2.339762	0.732657
H	3.658140	-1.691722	-0.822630

6

Si	-1.586396	-0.141784	-0.102404
C	-1.755219	1.553945	-0.907281
H	-1.369253	2.370833	-0.264599
H	-1.207746	1.601004	-1.869788
H	-2.827565	1.762335	-1.105881
C	-2.452629	-0.180241	1.576123
H	-3.549019	-0.058633	1.450547
H	-2.100057	0.627724	2.248346
H	-2.274883	-1.150458	2.083661
C	-2.255993	-1.502217	-1.212687
H	-3.345821	-1.379989	-1.380637
H	-2.088270	-2.498623	-0.755723
H	-1.749848	-1.488678	-2.199056
O	0.056282	-0.585472	0.097977
C	1.196549	0.160952	0.478507
C	2.373828	-0.839938	0.612754

H	2.108653	-1.504419	1.462642
H	3.279963	-0.272605	0.913522
C	2.637063	-1.663491	-0.645325
H	3.455133	-2.391188	-0.476020
H	1.727387	-2.222742	-0.938258
C	0.982265	0.920302	1.802823
H	0.704011	0.192928	2.590473
H	0.173259	1.670523	1.708154
H	1.905408	1.448698	2.111386
C	1.528634	1.148533	-0.592274
N	1.780223	1.913111	-1.443829
H	2.929378	-1.016810	-1.497973

1_Ph

O	2.223035	-1.316498	0.000140
C	1.709375	-0.203223	0.000004
C	0.210084	-0.049209	-0.000019
C	-0.572985	-1.224324	0.000014
C	-1.969907	-1.149089	-0.000019
C	-2.605801	0.105521	-0.000092
C	-1.837767	1.281220	-0.000128
C	-0.437048	1.205581	-0.000087
H	0.150967	2.135396	-0.000112
H	-2.333843	2.263930	-0.000188
H	-3.705148	0.167058	-0.000120
H	-2.571154	-2.071396	0.000012
H	-0.044279	-2.189639	0.000060
C	2.566469	1.052515	0.000124
H	2.356835	1.678068	0.892372
H	2.357114	1.678026	-0.892219
H	3.630712	0.756595	0.000290

IDPi **4**

P	-0.952258	-0.367551	0.503389
N	0.128696	-0.332962	-0.615244
P	1.301070	0.469458	-1.341937
N	1.056385	1.899468	-2.020013
S	-0.314409	2.634546	-2.389699
O	-0.112250	4.075340	-2.604815
O	-1.484659	2.208153	-1.542403
C	-0.674587	1.933460	-4.161209
F	0.030049	0.791736	-4.319576
F	-0.252224	2.849359	-5.048474
O	2.536646	0.558708	-0.268196
C	3.799836	0.872971	-0.776585
C	4.525969	-0.135224	-1.419500
C	5.806163	0.207381	-1.989066
C	6.570622	-0.698754	-2.783969
C	7.806832	-0.333618	-3.298648
C	8.341013	0.958157	-3.047825
C	7.607494	1.876153	-2.312756
C	6.323556	1.540491	-1.785966
C	5.527078	2.511551	-1.117898
C	4.250098	2.223918	-0.643459
H	5.920349	3.533611	-1.004901
H	7.996432	2.891408	-2.136962
H	9.326324	1.233604	-3.453519
H	8.373824	-1.050342	-3.912051
H	6.165613	-1.697759	-2.995875
C	3.929471	-1.497155	-1.549223
C	2.643136	-1.637361	-2.086194
C	1.970149	-2.894002	-2.230398
C	2.659730	-4.028765	-1.808763
C	3.951586	-3.959578	-1.221168

C	4.599692	-5.134585	-0.731796	H	-6.135244	-5.659245	4.630986
C	5.824661	-5.060518	-0.087139	H	-3.681580	-6.171136	4.855315
C	6.451123	-3.798228	0.093115	H	-2.015146	-4.965353	3.482356
C	5.858300	-2.640237	-0.390652	C	-1.103362	-3.360523	1.584487
C	4.603499	-2.680520	-1.072216	C	-0.149002	-2.380506	1.878392
H	6.349430	-1.670403	-0.233660	C	1.242356	-2.648670	2.088631
H	7.412573	-3.735322	0.624900	C	1.664274	-3.957383	1.861694
H	6.308330	-5.973243	0.292702	C	0.776323	-4.977827	1.426362
H	4.094414	-6.103838	-0.867418	C	1.256151	-6.281779	1.096781
H	2.181236	-5.014472	-1.911645	C	0.394404	-7.260440	0.626053
O	1.967096	-0.487083	-2.498900	C	-0.988362	-6.972285	0.472069
N	-1.501543	1.158337	0.953939	C	-1.490610	-5.722403	0.806423
S	-2.436184	1.580552	2.304411	C	-0.631658	-4.692357	1.295321
O	-3.186487	2.794399	1.966384	H	-2.559697	-5.507055	0.672049
O	-3.035032	0.372374	2.889730	H	-1.666983	-7.743819	0.078141
C	-1.052218	2.156476	3.532362	H	0.779056	-8.257490	0.363034
F	0.147888	1.767918	3.068257	H	2.332566	-6.485537	1.208226
F	-1.100573	3.500946	3.587716	H	2.724403	-4.211406	2.012048
O	-2.147322	-1.316737	-0.051521	O	-0.565902	-1.033200	1.943599
C	-3.042579	-1.969866	0.803423	C	1.316056	5.232324	0.442864
C	-2.557075	-3.023007	1.580718	C	1.524591	4.099863	1.262672
C	-3.482663	-3.726155	2.436566	H	0.890879	3.948868	2.145820
C	-3.081826	-4.729189	3.369475	C	2.496123	3.137622	0.968721
C	-4.017825	-5.404374	4.140899	H	2.593493	2.251054	1.611714
C	-5.401836	-5.109961	4.021367	C	3.326689	3.283836	-0.165854
C	-5.820969	-4.113254	3.154480	C	3.173785	4.448129	-0.945783
C	-4.882347	-3.384812	2.362879	H	3.787216	4.573260	-1.851031
C	-5.296533	-2.289611	1.558571	C	2.188243	5.398098	-0.650108
C	-4.401830	-1.517914	0.819365	C	-2.156460	-3.200821	-3.586587
H	-6.361724	-2.013141	1.559218	C	-1.660820	-3.956144	-2.500419
H	-6.887718	-3.851262	3.075748	H	-2.329199	-4.633340	-1.946958

C	-0.332207	-3.856981	-2.082542	F	-0.309686	2.114501	5.774342
H	0.000695	-4.435900	-1.210870	F	-2.463178	1.939173	5.430098
C	0.575861	-2.991976	-2.731518	C	-3.625808	-3.354330	-4.021072
C	0.095386	-2.247771	-3.828130	C	-3.835454	-4.796590	-4.543140
H	0.771992	-1.573125	-4.367819	H	-4.887630	-4.944014	-4.865062
C	-1.241162	-2.350088	-4.240524	H	-3.176282	-5.004636	-5.410376
C	-5.478237	2.454184	-0.598842	H	-3.611709	-5.550567	-3.761763
C	-4.648392	1.619387	-1.369430	C	-4.008532	-2.366588	-5.139345
H	-4.208004	1.990912	-2.299380	H	-5.078959	-2.490562	-5.400670
C	-4.319214	0.319186	-0.962974	H	-3.857155	-1.314139	-4.828482
H	-3.637973	-0.269615	-1.588810	H	-3.421397	-2.539275	-6.064217
C	-4.817646	-0.213039	0.244258	C	-4.558896	-3.106147	-2.812972
C	-5.698284	0.604711	0.995599	H	-5.619235	-3.243499	-3.109736
H	-6.075005	0.243697	1.964046	H	-4.355141	-3.799861	-1.973202
C	-6.024233	1.896947	0.579078	H	-4.446811	-2.074412	-2.427766
C	4.069053	0.388915	3.420155	C	0.132739	6.176017	0.722398
C	2.753981	0.335806	3.927572	C	0.100025	7.365829	-0.253747
H	2.414716	1.070468	4.670652	H	-0.020764	7.026478	-1.302054
C	1.838603	-0.637464	3.503602	H	-0.760938	8.022183	-0.014152
H	0.831070	-0.645990	3.936547	H	1.019985	7.982226	-0.187380
C	2.193706	-1.602248	2.539726	C	-1.181219	5.374015	0.547801
C	3.514933	-1.569507	2.045399	H	-2.061029	6.032265	0.706029
H	3.826709	-2.295542	1.283899	H	-1.245812	4.943723	-0.470619
C	4.425279	-0.600431	2.475514	H	-1.261946	4.545296	1.276179
H	-1.546704	1.818520	0.123091	C	0.221519	6.719718	2.166950
C	-2.165781	1.613809	-4.439954	H	0.192050	5.905800	2.918244
C	-1.255784	1.590663	4.968972	H	1.157956	7.293850	2.321966
F	-2.926282	2.701939	-4.234521	H	-0.634720	7.394026	2.376598
F	-2.314280	1.223121	-5.721775	C	-5.748000	3.922934	-0.967358
F	-2.591727	0.611770	-3.650328	C	-4.955832	4.357726	-2.215219
F	-1.133582	0.256834	4.983822	H	-3.863217	4.233452	-2.074058

H	-5.250387	3.783334	-3.116569
H	-5.149980	5.428659	-2.427153
C	-5.314787	4.824702	0.213987
H	-5.476123	5.892915	-0.040772
H	-5.890991	4.608385	1.135501
H	-4.243336	4.675635	0.452233
C	-7.257825	4.111869	-1.241924
H	-7.872997	3.837983	-0.361076
H	-7.477610	5.171208	-1.490454
H	-7.588508	3.483010	-2.093709
C	5.103453	1.426865	3.894719
C	6.137441	0.698365	4.787888
H	6.662816	-0.102228	4.228720
H	6.901970	1.412047	5.160337
H	5.646100	0.229835	5.664903
C	5.827829	2.066649	2.688453
H	5.117933	2.616812	2.040747
H	6.592721	2.787564	3.043854
H	6.347223	1.319303	2.056986
C	4.449723	2.558976	4.710458
H	3.984488	2.187496	5.645645
H	5.217173	3.304804	5.000226
H	3.673477	3.088323	4.120532
H	5.437786	-0.611894	2.045026
H	2.072975	6.254823	-1.326460
H	-6.677312	2.504474	1.224157
H	-1.564913	-1.737552	-5.092815

IDPi **5**

P	0.978964	-0.833276	-0.261331
N	-0.513196	-0.816155	0.165550
P	-1.588291	0.244494	0.708271
N	-1.029862	1.628125	1.285004
S	-1.027830	2.188496	2.802560
O	-0.075002	3.314416	2.897505
O	-1.008233	1.134345	3.845581
C	-2.730564	2.998403	3.024052
F	-2.796086	3.508232	4.258500
F	-2.907262	3.989034	2.133296
F	-3.705742	2.092532	2.867067
O	-2.580539	0.448811	-0.579591
C	-3.762877	1.145392	-0.311453
C	-4.778595	0.502428	0.401720
C	-5.915600	1.290822	0.802282
C	-6.936446	0.783127	1.660060
C	-8.004998	1.578829	2.048058
C	-8.106785	2.921093	1.595482
C	-7.121389	3.451252	0.776701
C	-6.001051	2.666694	0.370312
C	-4.965778	3.223019	-0.437057
C	-3.838254	2.489669	-0.780854
H	-5.052568	4.266193	-0.777763
H	-7.180439	4.496713	0.435664
H	-8.960845	3.541745	1.906486
H	-8.775350	1.167950	2.718287
H	-6.860113	-0.250481	2.026108
C	-4.642203	-0.951178	0.712475
C	-3.500582	-1.424408	1.369884
C	-3.233543	-2.810787	1.613670
C	-4.229733	-3.710399	1.234383
C	-5.409843	-3.305317	0.553598
C	-6.372692	-4.263635	0.113828
C	-7.471720	-3.878396	-0.637464

C	-7.644571	-2.512161	-0.985774
C	-6.739347	-1.552174	-0.554431
C	-5.612127	-1.910119	0.245742
H	-6.882495	-0.500872	-0.838339
H	-8.502035	-2.210556	-1.606423
H	-8.201481	-4.628737	-0.977492
H	-6.212919	-5.322895	0.369699
H	-4.089398	-4.781605	1.444524
O	-2.560132	-0.491762	1.803371
N	1.794167	0.623598	-0.589694
S	2.159290	1.334981	-2.095309
O	1.216430	0.848687	-3.106928
O	2.442709	2.747366	-1.822558
C	3.842139	0.551581	-2.561921
F	3.846915	-0.764854	-2.365734
F	4.033012	0.815916	-3.856861
F	4.818379	1.115066	-1.839639
O	1.839018	-1.614833	0.877879
C	3.159599	-1.957444	0.551486
C	3.363272	-3.038443	-0.309420
C	4.715642	-3.342480	-0.703306
C	5.034890	-4.347974	-1.664227
C	6.352196	-4.608983	-2.014388
C	7.415697	-3.875952	-1.423795
C	7.138469	-2.870307	-0.511159
C	5.794393	-2.563388	-0.140467
C	5.504799	-1.475990	0.730832
C	4.200693	-1.128282	1.073091
H	6.338519	-0.874266	1.124291
H	7.953565	-2.279018	-0.065449
H	8.456721	-4.097697	-1.703568
H	6.573115	-5.387625	-2.760156
H	4.221123	-4.916302	-2.135287
C	2.181379	-3.771785	-0.853609
C	1.171228	-3.063910	-1.512734
C	-0.016280	-3.653494	-2.041193
C	-0.141767	-5.033474	-1.908807
C	0.831481	-5.821074	-1.233890
C	0.644322	-7.224420	-1.050150
C	1.546215	-7.974096	-0.310777
C	2.673294	-7.343630	0.281706
C	2.896231	-5.985234	0.104622
C	2.000580	-5.188843	-0.670103
H	3.763773	-5.506195	0.579010
H	3.372828	-7.935516	0.891140
H	1.383882	-9.053248	-0.168966
H	-0.245546	-7.697883	-1.493425
H	-1.036353	-5.527132	-2.318643
O	1.299309	-1.665096	-1.620009
C	-0.485116	3.895542	-3.083816
C	-1.444623	3.063412	-3.676240
H	-1.315963	2.733980	-4.717208
C	-2.546484	2.612420	-2.937434
H	-3.276008	1.927086	-3.394000
C	-2.709302	3.025602	-1.598436
C	-1.757259	3.882503	-1.020669
H	-1.888223	4.199870	0.021250
C	-0.619354	4.313271	-1.740232
C	0.547364	-4.339161	3.019396
C	-0.155565	-4.985151	1.996815
H	0.266114	-5.885949	1.528178
C	-1.379453	-4.475564	1.550728

H	-1.899204	-4.969719	0.716613
C	-1.930094	-3.309151	2.135681
C	-1.216737	-2.678439	3.177512
H	-1.642871	-1.785887	3.653845
C	0.025918	-3.177887	3.634750
C	3.097395	2.592221	2.940524
C	2.524595	1.395129	3.392280
H	1.742002	1.423439	4.162919
C	2.901214	0.164320	2.837138
H	2.428363	-0.759849	3.197865
C	3.869097	0.123048	1.806685
C	4.468418	1.330794	1.387944
H	5.224532	1.309603	0.590260
C	4.090926	2.576364	1.935824
C	-3.185091	-1.294021	-3.740408
C	-3.450974	-2.191446	-2.699183
H	-4.471252	-2.279290	-2.297307
C	-2.419466	-2.962286	-2.148493
H	-2.626936	-3.640746	-1.307998
C	-1.101123	-2.827682	-2.637925
C	-0.845782	-1.919275	-3.683034
H	0.177579	-1.832617	-4.073508
C	-1.877907	-1.147545	-4.261977
H	1.860832	1.303983	0.186077
H	0.406658	4.188377	-3.657361
C	1.306195	6.039063	-1.764770
C	0.677143	5.104533	0.285022
C	2.197986	6.663732	-0.919013
H	1.267814	6.233640	-2.845436
H	0.214470	4.481241	1.062264
H	2.974965	7.396967	-1.165988
S	1.975353	6.152739	0.722079
C	0.416071	5.136307	-1.082508
H	2.734521	3.551105	3.337631
C	5.045465	4.948650	2.280457
C	4.987831	4.085262	0.107343
C	5.591723	6.000890	1.576656
H	4.912203	4.953523	3.371006
H	4.746371	3.457879	-0.758281
H	5.962145	6.958802	1.961880
C	4.697034	3.832051	1.443099
S	5.673433	5.652986	-0.119420
H	1.526022	-4.734849	3.327079
C	0.519719	-1.223374	5.183775
C	1.859431	-3.118580	5.457031
H	-0.196059	-0.486639	4.795372
C	2.400205	-2.289690	6.416405
H	2.217695	-4.143739	5.293599
H	3.218329	-2.502998	7.115175
C	0.769919	-2.515742	4.728991
S	1.589445	-0.758712	6.455088
H	-4.006813	-0.683239	-4.142238
C	-0.347348	0.254567	-5.732971
C	-2.619526	0.306711	-6.265179
H	0.599204	0.103168	-5.203013
C	-2.119076	1.158736	-7.225797
H	-3.684608	0.046353	-6.203259
H	-2.662959	1.679818	-8.023042
C	-1.607561	-0.224085	-5.385797
S	-0.399423	1.330421	-7.081679

6-IDPi **4**-TS2-*S*

P	-0.811324	1.010039	-0.623267

N	0.652547	0.914421	-0.029382	C	7.426349	3.965845	-0.592140
P	1.618109	-0.142641	0.623744	C	7.690100	2.586985	-0.807413
N	1.063572	-1.542648	1.260042	C	6.808812	1.619428	-0.343937
S	0.618563	-1.685130	2.821666	C	5.616550	1.979993	0.353240
O	-0.198766	-2.919131	2.938578	H	7.023832	0.557537	-0.522673
O	0.149071	-0.431031	3.453513	H	8.597527	2.280596	-1.349733
C	2.265806	-2.111842	3.722584	H	8.133352	4.724749	-0.960219
F	3.121227	-2.668777	2.842452	H	6.050780	5.417627	0.235194
F	2.796134	-0.975246	4.212215	H	3.937843	4.858510	1.322502
O	2.728899	-0.494340	-0.527552	O	2.534960	0.537357	1.801780
C	3.886755	-1.165012	-0.133461	N	-1.707603	-0.306895	-0.884493
C	4.837917	-0.452946	0.606378	S	-1.917081	-1.019183	-2.309394
C	5.970645	-1.168811	1.130219	O	-0.800805	-0.885194	-3.274135
C	6.912646	-0.577228	2.023418	O	-2.510149	-2.363859	-2.070010
C	7.991574	-1.304640	2.506099	C	-3.366927	-0.050565	-3.109473
C	8.183162	-2.656945	2.114714	F	-2.898963	1.084096	-3.662648
C	7.274947	-3.266038	1.262735	F	-4.267635	0.265237	-2.145160
C	6.143614	-2.553118	0.762612	O	-1.593580	2.013675	0.417810
C	5.190714	-3.180546	-0.084884	C	-2.800674	2.561387	-0.001582
C	4.038511	-2.533660	-0.533784	C	-2.763815	3.568927	-0.972546
H	5.379340	-4.218325	-0.398074	C	-4.010171	4.048017	-1.507156
H	7.405011	-4.318790	0.966378	C	-4.079359	4.961015	-2.600989
H	9.046384	-3.221762	2.498428	C	-5.302921	5.399154	-3.087829
H	8.701139	-0.831052	3.201569	C	-6.516070	4.947938	-2.501978
H	6.767116	0.466326	2.336645	C	-6.482768	4.046533	-1.449248
C	4.654660	1.014505	0.822021	C	-5.242284	3.562945	-0.934178
C	3.477788	1.484855	1.408546	C	-5.200307	2.620276	0.129068
C	3.162467	2.866793	1.599082	C	-4.006502	2.081158	0.608063
C	4.123770	3.783520	1.175570	H	-6.151783	2.317004	0.591470
C	5.336873	3.383202	0.549178	H	-7.417933	3.679358	-0.997737
C	6.272758	4.351036	0.073150	H	-7.480349	5.307141	-2.892582

H	-5.333156	6.097296	-3.938177
H	-3.144914	5.308361	-3.064182
C	-1.442290	4.112746	-1.410378
C	-0.474246	3.240741	-1.912763
C	0.844699	3.633336	-2.304910
C	1.158278	4.983294	-2.154923
C	0.237322	5.925128	-1.616649
C	0.612253	7.288182	-1.414810
C	-0.265304	8.196452	-0.842803
C	-1.563802	7.774032	-0.452391
C	-1.965545	6.459226	-0.646896
C	-1.088930	5.498532	-1.234869
H	-2.970183	6.145687	-0.333392
H	-2.256764	8.491245	0.013232
H	0.043111	9.241233	-0.685959
H	1.624587	7.602542	-1.714138
H	2.154853	5.338017	-2.459689
O	-0.810672	1.896044	-2.007758
C	1.185645	-4.635211	-3.088374
C	1.379827	-3.244900	-3.197614
H	0.792792	-2.650800	-3.908680
C	2.287629	-2.557631	-2.382093
H	2.390205	-1.475098	-2.522990
C	3.049030	-3.230794	-1.397922
C	2.861192	-4.630403	-1.291590
H	3.403978	-5.195439	-0.518511
C	1.964420	-5.311063	-2.121452
C	-0.584103	4.310151	3.288986
C	0.018849	4.922807	2.166579
H	-0.452411	5.796702	1.691493
C	1.212181	4.440540	1.622303
H	1.627811	4.930548	0.729029
C	1.867435	3.314679	2.172458
C	1.251619	2.673633	3.268242
H	1.699899	1.774165	3.709456
C	0.056625	3.168088	3.811554
C	-4.060046	-0.748668	3.936012
C	-2.987008	0.146643	3.744679
H	-2.129541	0.141051	4.431635
C	-2.949367	1.043369	2.674207
H	-2.081429	1.707585	2.584875
C	-3.995924	1.095108	1.721225
C	-5.072100	0.198683	1.905468
H	-5.892254	0.177581	1.171590
C	-5.106881	-0.693322	2.989670
C	3.820394	0.871407	-3.883274
C	4.160316	1.893559	-2.967513
H	5.203665	2.018683	-2.640577
C	3.197283	2.763370	-2.447494
H	3.503525	3.523690	-1.713054
C	1.837070	2.654965	-2.817955
C	1.483187	1.610776	-3.699783
H	0.435551	1.459260	-3.990339
C	2.456862	0.746101	-4.219468
Si	-5.147796	-4.443758	-0.930935
C	-4.316229	-4.893130	-2.536260
H	-3.417903	-4.263994	-2.682609
H	-4.026158	-5.962023	-2.547560
H	-5.012114	-4.701394	-3.378646
C	-5.681980	-2.658978	-0.741539
H	-6.061095	-2.453557	0.279460
H	-6.510072	-2.470608	-1.456597

H	-4.869337	-1.950004	-0.979383	H	1.647765	-6.968543	-4.487345
C	-6.535305	-5.621451	-0.466078	H	0.295534	-6.810704	-5.657579
H	-7.361061	-5.543136	-1.203427	H	1.618548	-5.594650	-5.636639
H	-6.950542	-5.387258	0.535105	C	-0.746999	-4.432594	-4.740133
H	-6.180780	-6.671745	-0.460606	H	-0.191648	-3.804622	-5.465675
O	-3.960183	-4.884307	0.335150	H	-1.496166	-5.013170	-5.316233
C	-3.093700	-4.310461	1.092552	H	-1.290543	-3.753472	-4.054033
C	-2.495427	-5.170241	2.165214	C	-0.694209	-6.331890	-3.113881
H	-3.164539	-4.975653	3.042345	H	-1.236465	-5.759818	-2.334678
H	-1.521275	-4.734067	2.461633	H	-1.439286	-6.850157	-3.753031
C	-2.422444	-6.660213	1.859456	H	-0.097941	-7.114371	-2.603102
H	-3.417460	-7.078231	1.611584	C	-4.054114	-1.707137	5.140700
H	-1.754145	-6.829455	0.990951	C	-2.690307	-2.429621	5.242339
H	-2.016415	-7.214036	2.727432	H	-2.472412	-3.021113	4.332451
C	-2.992361	-2.832206	1.180211	H	-1.840845	-1.730967	5.366503
H	-3.911230	-2.450871	1.678592	H	-2.686093	-3.117683	6.112912
H	-2.929410	-2.361416	0.182007	C	-5.160475	-2.773596	5.036346
H	-2.124963	-2.531406	1.790291	H	-5.100501	-3.468082	5.898608
C	-0.993617	-4.379833	-0.178320	H	-6.176149	-2.328677	5.042943
N	-0.097413	-3.644895	0.016934	H	-5.058495	-3.379133	4.110938
H	0.524843	-2.852555	0.371713	C	-4.287778	-0.871977	6.423312
C	2.034832	-3.098274	4.905937	H	-5.262777	-0.344778	6.383961
C	-4.092313	-0.872338	-4.219549	H	-4.281827	-1.524670	7.321392
F	-4.878760	-1.826456	-3.684156	H	-3.495625	-0.107524	6.552058
F	-4.872747	-0.050641	-4.946721	C	4.920442	-0.019717	-4.491283
F	-3.201930	-1.453218	-5.037554	C	5.883728	0.881103	-5.302286
F	3.151492	-3.160068	5.657209	H	6.689715	0.271160	-5.761698
F	1.755065	-4.328416	4.458623	H	5.344665	1.410535	-6.114190
F	1.025211	-2.662830	5.682439	H	6.362605	1.646908	-4.659605
C	0.184266	-5.396386	-3.976776	C	5.711747	-0.728539	-3.368483
C	0.983544	-6.241436	-4.997507	H	6.537284	-1.331107	-3.801161

H	6.159397	-0.006408	-2.658147
H	5.061111	-1.411380	-2.789028
C	4.346516	-1.097057	-5.430808
H	5.171625	-1.715328	-5.839156
H	3.651175	-1.778090	-4.899752
H	3.807795	-0.652525	-6.292274
C	-1.863389	4.910886	3.902490
C	-2.978289	4.981989	2.834170
H	-3.888213	5.457273	3.256116
H	-2.664905	5.574901	1.953166
H	-3.257899	3.972889	2.474850
C	-2.383263	4.085665	5.094949
H	-3.301150	4.554702	5.504304
H	-2.645005	3.050026	4.798283
H	-1.641416	4.035028	5.917694
C	-1.542968	6.341838	4.399189
H	-1.203163	6.995302	3.570788
H	-2.444944	6.806797	4.849693
H	-0.742797	6.325700	5.167021
H	2.123027	-0.042261	-4.907636
H	-5.971836	-1.366186	3.081346
H	-0.372151	2.634599	4.670916
H	1.849378	-6.396968	-1.984545

6-IDPi **4**-TS2-*R*

P	1.300271	-0.183132	1.023954
N	0.570059	0.954535	0.202740
P	-0.659024	1.212331	-0.734222
N	-1.596768	-0.065540	-1.121032
S	-2.257941	-0.390064	-2.565313
O	-2.648253	0.782002	-3.370981
O	-3.217595	-1.507498	-2.377066
C	-0.765701	-1.221189	-3.481427
F	0.389770	-0.823404	-2.913223
F	-0.887887	-2.566423	-3.337459
O	-1.559419	2.366373	0.019221
C	-2.557662	2.966067	-0.749729
C	-2.161732	3.914532	-1.695683
C	-3.154158	4.483217	-2.565448
C	-2.830342	5.366941	-3.637278
C	-3.823972	5.897799	-4.447597
C	-5.188713	5.571501	-4.224177
C	-5.533445	4.698252	-3.204451
C	-4.535230	4.121792	-2.362173
C	-4.874850	3.196131	-1.339607
C	-3.918429	2.577035	-0.533420
H	-5.938853	2.965792	-1.179677
H	-6.586594	4.424114	-3.034639
H	-5.968398	6.003313	-4.870003
H	-3.551620	6.572644	-5.273360
H	-1.776141	5.617502	-3.821582
C	-0.719584	4.281319	-1.782865
C	0.230300	3.281972	-2.010588
C	1.638478	3.524390	-2.077748
C	2.060301	4.837521	-1.874302
C	1.153403	5.894989	-1.588274
C	1.618842	7.217198	-1.316246
C	0.734777	8.232133	-0.984207
C	-0.658072	7.961848	-0.912498
C	-1.145158	6.691093	-1.186558
C	-0.263278	5.624949	-1.538872
H	-2.223769	6.493150	-1.121591
H	-1.357803	8.764359	-0.633579

H	1.109398	9.244311	-0.768674	C	5.063812	3.218606	1.844033
H	2.701891	7.411886	-1.363376	C	6.121512	4.148474	1.607135
H	3.135895	5.064812	-1.931456	C	7.384118	3.715914	1.232507
O	-0.217921	1.963920	-2.131706	C	7.638488	2.326713	1.079013
N	0.428290	-1.473880	1.404886	C	6.637142	1.395359	1.318182
S	0.704869	-2.509784	2.611333	C	5.328860	1.805463	1.717766
O	0.102924	-3.821055	2.249823	H	6.846760	0.324218	1.192285
O	2.055480	-2.501985	3.211243	H	8.636184	1.984161	0.764669
C	-0.413275	-1.772074	3.996966	H	8.186706	4.445326	1.044960
F	0.353404	-0.943780	4.740106	H	5.907912	5.223683	1.715071
F	-1.420964	-1.055895	3.452136	H	3.568419	4.744087	2.248859
O	2.663295	-0.536266	0.154326	O	1.938864	0.475120	2.393906
C	3.642839	-1.262038	0.831489	C	-5.285982	-0.215719	2.525982
C	4.426523	-0.592268	1.775697	C	-4.156712	0.601844	2.735864
C	5.349590	-1.354583	2.571702	H	-3.607251	0.550175	3.685263
C	6.098229	-0.784907	3.644239	C	-3.681720	1.479676	1.752898
C	6.978299	-1.556896	4.389436	H	-2.795335	2.088989	1.972344
C	7.153288	-2.936360	4.098397	C	-4.324704	1.587848	0.498149
C	6.424053	-3.524720	3.076920	C	-5.439362	0.747159	0.271793
C	5.499001	-2.763177	2.301388	H	-5.933958	0.757558	-0.711394
C	4.718384	-3.367073	1.279862	C	-5.910420	-0.120013	1.262942
C	3.769984	-2.657527	0.545546	C	4.650567	0.492080	-2.894815
H	4.870240	-4.435934	1.065690	C	3.440541	0.530056	-3.620226
H	6.536866	-4.597645	2.855152	H	3.255960	-0.186753	-4.433149
H	7.857439	-3.537586	4.693534	C	2.446300	1.481608	-3.346012
H	7.540182	-1.097332	5.216904	H	1.524241	1.483648	-3.942751
H	5.961852	0.278918	3.884040	C	2.624151	2.443571	-2.330610
C	4.250740	0.878792	1.945115	C	3.816300	2.387245	-1.575615
C	2.980276	1.385065	2.234733	H	3.963668	3.084757	-0.737409
C	2.681308	2.781637	2.331514	C	4.801121	1.436388	-1.854725
C	3.749003	3.662283	2.155416	C	1.788052	-4.846151	-2.645611

C	2.168809	-3.499234	-2.811014	C	-2.623631	-4.924632	0.211023
H	2.036302	-3.000939	-3.780549	C	-2.290926	-5.594764	1.498681
C	2.736838	-2.757225	-1.765926	H	-1.672023	-6.475823	1.190663
H	3.025074	-1.712820	-1.947539	H	-1.568775	-4.944476	2.034677
C	2.972010	-3.342037	-0.503317	C	-3.474500	-6.007886	2.361296
C	2.508500	-4.662641	-0.301522	H	-4.166195	-6.689787	1.828788
H	2.590758	-5.113692	0.698761	H	-4.040533	-5.103976	2.663771
C	1.933092	-5.392094	-1.348623	H	-3.118698	-6.513793	3.278855
C	-1.277523	4.466586	3.018637	C	-1.555148	-4.268802	-0.588015
C	-0.381707	5.006802	2.069067	H	-0.795761	-3.813438	0.075465
H	-0.664118	5.893933	1.482439	H	-1.038361	-5.064779	-1.169076
C	0.873945	4.439498	1.839906	H	-1.950633	-3.525873	-1.301977
H	1.525279	4.876168	1.067971	C	-3.144049	-2.640392	1.306277
C	1.305464	3.297259	2.552777	N	-2.729756	-1.698373	0.739210
C	0.397485	2.720110	3.466657	H	-2.283061	-0.967911	0.102781
H	0.676384	1.812410	4.019454	C	1.273003	-5.718460	-3.807951
C	-0.861055	3.299586	3.693268	C	1.248375	-4.950968	-5.142924
Si	-4.868011	-4.684579	-1.627091	H	0.572189	-4.075707	-5.103447
C	-3.893264	-4.574290	-3.220648	H	2.257857	-4.592712	-5.428620
H	-3.230533	-5.451765	-3.360615	H	0.889870	-5.617263	-5.953712
H	-3.302775	-3.639814	-3.277024	C	2.209923	-6.940044	-3.964930
H	-4.618004	-4.566366	-4.062457	H	2.233232	-7.565643	-3.050253
C	-6.013054	-6.170351	-1.602160	H	1.873448	-7.582795	-4.805260
H	-6.493853	-6.281927	-0.609560	H	3.248962	-6.614845	-4.175052
H	-6.814112	-6.047779	-2.360211	C	-0.163258	-6.207722	-3.511876
H	-5.467805	-7.107692	-1.833654	H	-0.525914	-6.869893	-4.325660
C	-5.686358	-3.108992	-1.060164	H	-0.217564	-6.785486	-2.566317
H	-6.554173	-2.897229	-1.719301	H	-0.857965	-5.347731	-3.438515
H	-6.048812	-3.204447	-0.017975	C	-5.843840	-1.145717	3.620417
H	-4.982669	-2.257085	-1.125127	C	-6.305739	-2.492903	3.018351
O	-3.793001	-5.145784	-0.263407	H	-6.667745	-3.163972	3.824214

H	-7.141125	-2.371350	2.299695
H	-5.465063	-2.994594	2.497051
C	-4.785631	-1.451069	4.698912
H	-4.480494	-0.544749	5.259257
H	-5.194090	-2.167518	5.440331
H	-3.879638	-1.901564	4.252095
C	-7.052527	-0.437017	4.278339
H	-7.846711	-0.223562	3.534088
H	-7.490028	-1.071736	5.077554
H	-6.747414	0.528046	4.731633
C	5.805206	-0.474188	-3.223818
C	6.999029	0.366785	-3.740326
H	7.343763	1.093368	-2.977166
H	7.856130	-0.291280	-3.995330
H	6.718972	0.938506	-4.648538
C	5.420890	-1.495710	-4.310955
H	5.160883	-1.002733	-5.269658
H	6.276757	-2.172439	-4.509294
H	4.564567	-2.127573	-4.000191
C	6.241109	-1.248623	-1.958242
H	6.546579	-0.569401	-1.138368
H	5.423607	-1.887671	-1.573004
H	7.105097	-1.905284	-2.189935
C	-2.613587	5.179271	3.304813
C	-3.486800	4.407745	4.312539
H	-4.436973	4.954531	4.479586
H	-3.748279	3.395685	3.943595
H	-2.988226	4.302597	5.297488
C	-2.297996	6.574590	3.898027
H	-1.708609	7.193407	3.191954
H	-3.237754	7.120395	4.125606
H	-1.714112	6.484229	4.836583
C	-3.418762	5.356161	1.996457
H	-4.353798	5.920221	2.194204
H	-2.846404	5.913411	1.229057
H	-3.699746	4.378918	1.559309
H	-1.521618	2.823468	4.431486
H	-6.780678	-0.751126	1.031583
H	1.608893	-6.425967	-1.147671
H	5.710803	1.437829	-1.236350
C	-1.016682	-2.857201	4.931653
C	-0.714915	-0.894566	-4.997041
F	-0.056040	-3.673809	5.385845
F	-1.612678	-2.263297	5.987453
F	-1.947939	-3.585814	4.286798
F	0.280245	-1.611686	-5.577657
F	-0.463370	0.402769	-5.203056
F	-1.874148	-1.231682	-5.580342

30-IDPi **4**-TS2-*S*

P	0.185065	1.504139	-0.551784
N	1.308307	0.509700	-0.037512
P	1.389639	-0.942811	0.562454
N	0.043073	-1.698920	1.095672
S	-0.456889	-1.674923	2.642056
O	-1.908884	-1.980641	2.652779
O	0.051076	-0.553393	3.460477
C	0.362030	-3.257251	3.359085
F	0.520972	-4.156514	2.365754
F	1.565986	-2.925791	3.862096
O	2.095850	-1.856750	-0.598757
C	2.594919	-3.108883	-0.239796
C	3.754027	-3.155487	0.543541

C	4.201918	-4.435783	1.023763	O	-0.752162	0.020316	-3.291884
C	5.275536	-4.579504	1.951989	O	-3.121025	-0.031173	-2.313637
C	5.676041	-5.833876	2.390607	C	-2.197430	2.300789	-3.263502
C	5.024771	-7.005132	1.918725	F	-1.073896	2.835206	-3.779346
C	3.965609	-6.897844	1.030840	F	-2.713274	3.146201	-2.339406
C	3.515750	-5.622371	0.573344	O	0.086574	2.651625	0.620729
C	2.410649	-5.502111	-0.312049	C	-0.555404	3.839547	0.281424
C	1.903849	-4.268595	-0.722887	C	0.122418	4.738987	-0.551524
H	1.944318	-6.424650	-0.688990	C	-0.578258	5.910075	-1.007176
H	3.443348	-7.798515	0.671520	C	-0.036730	6.807458	-1.975269
H	5.355511	-7.995004	2.268311	C	-0.747559	7.925933	-2.387558
H	6.501262	-5.920856	3.113706	C	-2.032635	8.203608	-1.849076
H	5.778698	-3.677456	2.327899	C	-2.595342	7.339540	-0.922630
C	4.484423	-1.890028	0.858078	C	-1.900015	6.169755	-0.491075
C	3.806857	-0.842941	1.484119	C	-2.487445	5.259485	0.428451
C	4.383919	0.427760	1.797624	C	-1.864308	4.072774	0.816463
C	5.715458	0.609533	1.428893	H	-3.472510	5.511666	0.849012
C	6.462651	-0.396666	0.753910	H	-3.598470	7.535546	-0.512120
C	7.806413	-0.157668	0.334334	H	-2.583576	9.097805	-2.178204
C	8.520025	-1.117279	-0.367095	H	-0.313670	8.599910	-3.141868
C	7.913193	-2.363903	-0.674923	H	0.953167	6.595183	-2.403145
C	6.613162	-2.634806	-0.269627	C	1.532434	4.448365	-0.953348
C	5.851024	-1.671573	0.456925	C	1.824625	3.236528	-1.582371
H	6.156282	-3.601998	-0.518209	C	3.137520	2.817634	-1.966838
H	8.474543	-3.121309	-1.242917	C	4.176533	3.702633	-1.683473
H	9.551937	-0.913653	-0.691042	C	3.961529	4.942663	-1.018579
H	8.262764	0.816442	0.570767	C	5.055169	5.798179	-0.684744
H	6.208643	1.565584	1.662845	C	4.855162	6.979668	0.012631
O	2.464053	-1.045968	1.795435	C	3.542135	7.357326	0.400346
N	-1.305493	1.015829	-0.934916	C	2.454342	6.556983	0.077886
S	-1.786958	0.621618	-2.417258	C	2.623734	5.334532	-0.638813

H	1.444695	6.860574	0.385153
H	3.383567	8.289329	0.963937
H	5.710662	7.622236	0.270535
H	6.069941	5.491016	-0.983330
H	5.202963	3.433703	-1.976843
O	0.765967	2.370091	-1.821497
C	-1.514078	-4.119729	-3.436583
C	-0.507754	-3.135361	-3.472645
H	-0.575591	-2.290873	-4.168599
C	0.586429	-3.161207	-2.599113
H	1.330147	-2.358724	-2.677622
C	0.731064	-4.184660	-1.633586
C	-0.276891	-5.178920	-1.597832
H	-0.229335	-5.973853	-0.837886
C	-1.360535	-5.150692	-2.481727
C	2.121957	3.618492	3.712679
C	3.010408	3.872777	2.642504
H	3.142195	4.898343	2.266541
C	3.727211	2.844602	2.022991
H	4.376700	3.084107	1.167568
C	3.593255	1.503108	2.447912
C	2.694328	1.239028	3.502720
H	2.533271	0.210442	3.849696
C	1.977747	2.275793	4.115993
C	-3.898632	1.511415	3.711392
C	-2.489048	1.572083	3.650045
H	-1.876576	0.985104	4.349828
C	-1.817824	2.361934	2.711237
H	-0.720757	2.372852	2.729985
C	-2.530257	3.137196	1.762308
C	-3.939310	3.040362	1.789322
H	-4.534211	3.580437	1.037117
C	-4.605223	2.258830	2.745422
C	3.954130	-1.042644	-3.814545
C	4.789660	-0.501432	-2.810224
H	5.677749	-1.056045	-2.471390
C	4.515222	0.732623	-2.213541
H	5.175000	1.098111	-1.412013
C	3.384210	1.492690	-2.590843
C	2.527016	0.943298	-3.568551
H	1.617440	1.476610	-3.873389
C	2.811734	-0.293846	-4.163610
Si	-6.495051	-0.241260	-1.808537
C	-5.965196	-0.784845	-3.511462
H	-4.861263	-0.836514	-3.556632
H	-6.390168	-1.777863	-3.757710
H	-6.308532	-0.049402	-4.266748
C	-5.873069	1.429161	-1.231270
H	-6.168390	1.637006	-0.183823
H	-6.335561	2.204412	-1.877591
H	-4.776556	1.519474	-1.330393
C	-8.354082	-0.378764	-1.551441
H	-8.879466	0.354951	-2.197706
H	-8.634537	-0.165814	-0.499810
H	-8.724623	-1.389996	-1.814613
O	-5.953335	-1.563800	-0.749907
C	-5.141196	-1.803601	0.228036
C	-4.346487	-0.686964	0.821747
H	-5.053189	-0.054451	1.403729
H	-3.886874	-0.060526	0.037007
H	-3.558061	-1.028427	1.513129
C	-3.140217	-2.610772	-0.758803

N	-2.028995	-2.548578	-0.383509
H	-1.105484	-2.305561	0.104083
C	-0.498329	-3.898473	4.488524
C	-3.224307	2.139299	-4.428100
F	-4.478476	2.001996	-3.956387
F	-3.194170	3.235542	-5.209054
F	-2.924570	1.069765	-5.182541
F	0.232653	-4.818328	5.144284
F	-1.581570	-4.507601	3.975620
F	-0.891595	-2.960367	5.367342
C	-2.723648	-4.088671	-4.389791
C	-2.557192	-5.226494	-5.425507
H	-2.512174	-6.219183	-4.932838
H	-3.410578	-5.235426	-6.135974
H	-1.623143	-5.096807	-6.009290
C	-2.824727	-2.741738	-5.134180
H	-1.969282	-2.579792	-5.820422
H	-3.747329	-2.720510	-5.749440
H	-2.858939	-1.886097	-4.431091
C	-4.037940	-4.302407	-3.602574
H	-4.167169	-3.523096	-2.824180
H	-4.905500	-4.259218	-4.294064
H	-4.069062	-5.288167	-3.096583
C	-4.588269	0.699890	4.823802
C	-4.073150	-0.757330	4.831252
H	-4.344835	-1.282531	3.895313
H	-2.972971	-0.824588	4.927224
H	-4.523811	-1.316289	5.677662
C	-6.118705	0.667028	4.656503
H	-6.573415	0.062659	5.467275
H	-6.568378	1.679483	4.706300
H	-6.415580	0.202605	3.693014
C	-4.254772	1.367306	6.180894
H	-4.613553	2.416518	6.207302
H	-4.735985	0.814517	7.014809
H	-3.162397	1.379629	6.366682
C	4.325641	-2.374873	-4.493195
C	5.691299	-2.200225	-5.201022
H	5.988421	-3.142148	-5.708179
H	5.643827	-1.396807	-5.964146
H	6.492782	-1.936399	-4.481907
C	4.442273	-3.494565	-3.434200
H	4.749705	-4.447609	-3.912953
H	5.192401	-3.249667	-2.657531
H	3.474759	-3.667429	-2.925219
C	3.281661	-2.806568	-5.540442
H	3.589794	-3.766481	-6.002515
H	2.282314	-2.963537	-5.086444
H	3.180077	-2.062141	-6.356133
C	1.378310	4.782870	4.394551
C	0.517048	5.535265	3.355146
H	0.008890	6.402156	3.826796
H	1.126764	5.916907	2.513652
H	-0.263471	4.875192	2.930653
C	0.453222	4.299274	5.527397
H	-0.058666	5.167526	5.989884
H	-0.333145	3.612882	5.152570
H	1.016908	3.779271	6.328386
C	2.424133	5.754921	4.992324
H	3.086340	6.175016	4.208633
H	1.919508	6.604198	5.498924
H	3.064935	5.240339	5.737115

H	2.108498	-0.667882	-4.920221	C	1.979438	-7.154703	3.955931
H	-5.704624	2.241676	2.725720	C	0.737033	-7.830488	3.819445
H	1.288971	2.007784	4.928369	C	-0.204511	-7.371396	2.911602
H	-2.122948	-5.940313	-2.399123	C	0.055151	-6.226226	2.099944
C	-5.413785	-3.058101	0.968750	C	-0.916015	-5.729741	1.189608
C	-4.565600	-3.499125	2.012289	C	-0.713244	-4.584287	0.418012
C	-6.566140	-3.815041	0.646730	H	-1.861370	-6.283459	1.086889
C	-4.874942	-4.664613	2.722442	H	-1.177016	-7.878274	2.809550
H	-3.634472	-2.965263	2.245048	H	0.520681	-8.710835	4.443680
C	-6.871513	-4.975635	1.364840	H	2.715844	-7.508761	4.693421
H	-7.220657	-3.472274	-0.166584	H	3.228895	-5.523601	3.289965
C	-6.027927	-5.402876	2.406029	C	2.892502	-3.702394	1.383485
H	-4.197343	-4.998118	3.522407	C	2.924323	-2.340456	1.692300
H	-7.773928	-5.553111	1.113066	C	4.111836	-1.543145	1.670033
H	-6.268442	-6.318669	2.967950	C	5.291220	-2.187049	1.298796
**30**-IDPi **4**-TS2-*R*				C	5.326445	-3.560657	0.930371
P	0.912051	1.072633	-0.929025	C	6.536199	-4.177755	0.489130
N	1.367710	-0.267698	-0.215290	C	6.554864	-5.500949	0.075737
P	0.740148	-1.375347	0.704431	C	5.356745	-6.264075	0.090733
N	-0.770317	-1.144822	1.271062	C	4.166667	-5.699784	0.529756
S	-1.315356	-1.451972	2.768421	C	4.113327	-4.342188	0.967833
O	-0.672705	-2.581649	3.465394	H	3.245577	-6.298470	0.533294
O	-2.795430	-1.354446	2.737123	H	5.369607	-7.310127	-0.251437
C	-0.774189	0.159727	3.689861	H	7.493485	-5.959573	-0.270682
F	0.264373	0.722182	3.039686	H	7.456852	-3.573354	0.471113
F	-1.825630	1.016462	3.672944	H	6.229458	-1.611419	1.282212
O	0.805248	-2.774800	-0.163497	O	1.714087	-1.710323	1.986520
C	0.561317	-3.945033	0.556050	N	-0.668171	1.293490	-1.070268
C	1.584423	-4.419631	1.381439	S	-1.438598	2.291372	-2.072244
C	1.329721	-5.561525	2.214978	O	-2.758968	2.629860	-1.481118
C	2.269506	-6.046933	3.171982	O	-0.640593	3.376027	-2.684465

C	-1.829092	1.115187	-3.545456	H	8.964582	2.757055	-1.887910
F	-0.835213	1.245983	-4.450113	H	7.833452	0.620620	-2.523513
F	-1.864790	-0.168151	-3.121484	H	5.790797	-0.682730	-2.873453
O	1.697447	2.279007	-0.114051	O	1.629177	1.158252	-2.413506
C	1.791373	3.512708	-0.755727	C	-3.837486	-3.445350	-2.405795
C	2.699847	3.647154	-1.810037	C	-2.477296	-3.209181	-2.684819
C	2.718757	4.879638	-2.551139	H	-2.177531	-2.760961	-3.641175
C	3.515357	5.066683	-3.720083	C	-1.465190	-3.522119	-1.766889
C	3.506361	6.273706	-4.404938	H	-0.422706	-3.319766	-2.043963
C	2.700315	7.353333	-3.954617	C	-1.766392	-4.105813	-0.515535
C	1.897122	7.195636	-2.835846	C	-3.134878	-4.304526	-0.212961
C	1.870995	5.962681	-2.117267	H	-3.420559	-4.701050	0.773263
C	1.017734	5.778486	-0.996374	C	-4.136571	-3.987690	-1.135414
C	0.934296	4.567498	-0.313535	C	4.235443	2.694260	2.656941
H	0.393912	6.622593	-0.664691	C	3.470718	1.789422	3.423214
H	1.252458	8.018653	-2.489129	H	2.915611	2.137351	4.306082
H	2.705056	8.307717	-4.502975	C	3.400445	0.425886	3.100281
H	4.124309	6.392448	-5.308088	H	2.801806	-0.245430	3.730432
H	4.134905	4.234300	-4.082315	C	4.099184	-0.093629	1.991186
C	3.589608	2.498631	-2.144216	C	4.834190	0.813633	1.196158
C	3.019265	1.253625	-2.424131	H	5.341687	0.454407	0.288118
C	3.780236	0.066451	-2.671335	C	4.899556	2.170265	1.524699
C	5.168786	0.199717	-2.658844	C	-1.710628	4.474328	3.134654
C	5.816534	1.431257	-2.365657	C	-0.445456	3.853545	3.168820
C	7.240318	1.521234	-2.299050	H	-0.079513	3.390835	4.095014
C	7.866538	2.705689	-1.943037	C	0.386032	3.808253	2.041345
C	7.086785	3.853163	-1.637333	H	1.362876	3.310866	2.115511
C	5.701290	3.804233	-1.709799	C	-0.002323	4.414732	0.828378
C	5.024334	2.604644	-2.086193	C	-1.300849	4.969375	0.762069
H	5.109719	4.697270	-1.466706	H	-1.662626	5.371844	-0.195973
H	7.583199	4.788739	-1.337631	C	-2.131485	4.992993	1.888405

C	2.128518	-3.899997	-3.389415	C	-2.600327	4.629012	4.384103
C	3.231326	-3.686445	-2.532223	C	-1.942075	4.038152	5.644539
H	3.719736	-4.537685	-2.034557	H	-1.756357	2.952417	5.542395
C	3.732318	-2.405714	-2.287423	H	-0.975997	4.531673	5.873512
H	4.576379	-2.285330	-1.591534	H	-2.606573	4.185543	6.520106
C	3.153651	-1.263368	-2.886610	C	-2.856989	6.135095	4.630177
C	2.014987	-1.460553	-3.696879	H	-3.371707	6.613054	3.772845
H	1.508525	-0.598231	-4.152609	H	-3.492879	6.278699	5.528865
C	1.524732	-2.752350	-3.945616	H	-1.902788	6.675833	4.793234
Si	-6.394895	-0.711135	2.512205	C	-3.950451	3.908403	4.164320
C	-5.449028	0.042510	3.942082	H	-4.604014	4.026792	5.053899
H	-5.699366	1.111910	4.091663	H	-4.497937	4.322769	3.293094
H	-4.355361	-0.079196	3.818638	H	-3.792868	2.825161	3.994172
H	-5.744484	-0.502019	4.864208	C	-4.963983	-3.150627	-3.413691
C	-8.244191	-0.736030	2.836584	C	-5.965265	-2.138892	-2.807273
H	-8.800221	-1.100998	1.949777	H	-6.792151	-1.942133	-3.521763
H	-8.471477	-1.412983	3.686029	H	-6.416071	-2.517807	-1.867315
H	-8.624495	0.272935	3.095077	H	-5.469156	-1.176864	-2.572552
C	-5.737603	-2.342526	1.889630	C	-4.419265	-2.561933	-4.728413
H	-5.950157	-3.132556	2.639901	H	-3.733989	-3.266665	-5.241939
H	-6.222063	-2.623514	0.933791	H	-5.257124	-2.347380	-5.422268
H	-4.641506	-2.288544	1.744233	H	-3.875906	-1.612513	-4.561981
O	-6.372584	0.347162	1.077929	C	-5.700310	-4.472920	-3.737342
C	-5.626863	1.196836	0.452071	H	-6.152328	-4.925935	-2.832041
C	-4.454904	1.827172	1.129768	H	-6.517482	-4.292378	-4.466861
H	-3.700147	2.181665	0.402105	H	-5.005018	-5.217612	-4.175597
H	-4.827770	2.704231	1.702785	C	4.414729	4.178868	3.030524
H	-3.974037	1.129530	1.836663	C	5.903473	4.405146	3.393179
C	-4.033519	-0.380072	-0.586349	H	6.569916	4.166966	2.539913
N	-2.955299	-0.695291	-0.246895	H	6.077948	5.464795	3.675167
H	-2.014740	-0.887921	0.226547	H	6.206401	3.765783	4.247352

C	3.551633	4.587154	4.239276
H	3.816370	4.012862	5.150243
H	3.709999	5.660731	4.467122
H	2.469903	4.447507	4.041529
C	4.042277	5.084577	1.834074
H	4.647995	4.854676	0.935874
H	2.977296	4.971000	1.554708
H	4.213975	6.150327	2.091077
C	1.672322	-5.337101	-3.708933
C	0.421557	-5.369506	-4.606955
H	0.134934	-6.420920	-4.811637
H	-0.446742	-4.878182	-4.124004
H	0.602343	-4.879029	-5.584924
C	2.826426	-6.055729	-4.450710
H	3.744154	-6.101718	-3.830521
H	2.535737	-7.096943	-4.704011
H	3.081636	-5.529245	-5.392968
C	1.355577	-6.101021	-2.402239
H	1.075568	-7.150815	-2.628686
H	2.224405	-6.125425	-1.715784
H	0.511428	-5.635517	-1.858207
H	0.651690	-2.852352	-4.606000
H	-5.184696	-4.164986	-0.848649
H	-3.130213	5.448426	1.792986
H	5.484169	2.833941	0.870504
C	-3.183441	1.442784	-4.237984
C	-0.358110	-0.088789	5.163568
F	-3.301498	2.760670	-4.464252
F	-3.248112	0.802386	-5.427430
F	-4.217182	1.027778	-3.488129
F	-0.105330	1.099370	5.768123
F	0.753186	-0.830942	5.232105
F	-1.352346	-0.696247	5.827651
C	-6.196426	1.738800	-0.791233
C	-7.287616	1.075043	-1.406234
C	-5.675238	2.912762	-1.382621
C	-7.829773	1.566481	-2.594621
H	-7.677539	0.155179	-0.949054
C	-6.229926	3.404622	-2.569755
H	-4.812500	3.426267	-0.942554
C	-7.299900	2.732671	-3.180265
H	-8.663216	1.035189	-3.078283
H	-5.801844	4.305094	-3.033924
H	-7.721370	3.112457	-4.123833

6-IDPi **5**-TS2-*S*

P	0.527398	1.409190	-0.958781
N	0.275518	-0.104535	-0.641740
P	0.516399	-1.430278	0.179644
N	-0.775554	-2.166467	0.799667
S	-1.340284	-2.083185	2.295714
O	-2.755795	-2.539575	2.312826
O	-0.970319	-0.852362	3.035492
C	-0.448985	-3.498298	3.204088
F	0.883947	-3.374268	3.121477
F	-0.808365	-3.471673	4.496107
F	-0.804972	-4.683545	2.675425
O	1.189243	-2.474311	-0.900374
C	1.585216	-3.693176	-0.342385
C	2.771456	-3.715735	0.389911
C	3.134647	-4.926606	1.083918
C	4.264471	-5.016362	1.951548
C	4.564432	-6.198034	2.614664

C	3.751296	-7.349725	2.444654
C	2.632596	-7.289399	1.628457
C	2.287550	-6.086102	0.943252
C	1.109662	-6.002086	0.150265
C	0.729432	-4.827338	-0.490230
H	0.470384	-6.892987	0.051207
H	1.981623	-8.169051	1.504282
H	4.002749	-8.282484	2.972021
H	5.437665	-6.239453	3.283400
H	4.896397	-4.130872	2.102907
C	3.583572	-2.474727	0.536517
C	3.021210	-1.338023	1.130291
C	3.810266	-0.227694	1.579549
C	5.150890	-0.209982	1.196405
C	5.740831	-1.256224	0.435845
C	7.098771	-1.189424	0.001536
C	7.667438	-2.226183	-0.723386
C	6.898198	-3.380778	-1.032697
C	5.580270	-3.483827	-0.608913
C	4.962713	-2.429743	0.129679
H	4.987407	-4.374503	-0.861441
H	7.349495	-4.198737	-1.614733
H	8.713472	-2.158219	-1.059286
H	7.681656	-0.288332	0.246270
H	5.788992	0.625644	1.521172
O	1.655984	-1.301951	1.379720
N	-0.876462	2.144842	-1.320936
S	-1.189457	3.490510	-2.195039
O	-2.443625	4.084190	-1.677420
O	-0.026488	4.339663	-2.507391
C	-1.702067	2.730598	-3.853687
F	-0.640564	2.171049	-4.455336
F	-2.643366	1.780572	-3.668885
F	-2.202576	3.686237	-4.641827
O	1.305219	2.105610	0.313627
C	1.536859	3.477458	0.148748
C	2.573700	3.871429	-0.700986
C	2.715951	5.273032	-1.000243
C	3.638166	5.768998	-1.969000
C	3.742954	7.128615	-2.225826
C	2.930779	8.060605	-1.526707
C	2.002947	7.608839	-0.601517
C	1.856473	6.215759	-0.327825
C	0.858495	5.744046	0.567353
C	0.648979	4.387377	0.803131
H	0.212488	6.478157	1.072783
H	1.347769	8.318839	-0.072879
H	3.027273	9.137201	-1.734203
H	4.456258	7.485831	-2.984185
H	4.262836	5.058248	-2.527248
C	3.473505	2.840559	-1.294238
C	2.942807	1.760765	-2.012757
C	3.745275	0.721547	-2.595148
C	5.128131	0.867181	-2.475112
C	5.731926	1.916106	-1.735593
C	7.149339	1.981638	-1.574081
C	7.731531	2.953026	-0.774950
C	6.910571	3.899517	-0.104883
C	5.531962	3.879494	-0.265801
C	4.900574	2.902831	-1.095812
H	4.912331	4.621065	0.256473
H	7.370252	4.657177	0.547745

H	8.824538	2.987928	-0.650260
H	7.768594	1.226616	-2.082865
H	5.787740	0.125592	-2.948197
O	1.563269	1.703559	-2.199991
C	-2.984954	-4.722567	-2.660941
C	-1.795124	-4.351959	-3.302815
H	-1.813931	-4.027446	-4.354604
C	-0.581153	-4.348087	-2.600689
H	0.343583	-4.021021	-3.094550
C	-0.545137	-4.757431	-1.252131
C	-1.745651	-5.125441	-0.615918
H	-1.715377	-5.405656	0.446047
C	-2.980577	-5.095731	-1.296495
C	2.633126	2.693884	4.511419
C	3.407943	3.073462	3.407590
H	3.745609	4.116327	3.306055
C	3.746027	2.136932	2.423796
H	4.355625	2.440362	1.562254
C	3.295406	0.801709	2.522496
C	2.483123	0.438772	3.619094
H	2.128590	-0.596846	3.712771
C	2.155717	1.367518	4.632653
C	-2.696383	3.147699	3.218707
C	-1.450902	2.532669	3.396798
H	-1.331914	1.727606	4.135974
C	-0.356302	2.898818	2.601438
H	0.604800	2.388823	2.732328
C	-0.491187	3.922388	1.637784
C	-1.752623	4.530602	1.465700
H	-1.870537	5.293821	0.684042
C	-2.871152	4.145738	2.233274
C	2.249126	-2.882703	-4.463341
C	3.463332	-2.877353	-3.764920
H	4.052449	-3.802759	-3.673511
C	3.919956	-1.708074	-3.148333
H	4.849367	-1.744116	-2.564656
C	3.186626	-0.499460	-3.241283
C	1.965744	-0.520992	-3.949911
H	1.388357	0.402947	-4.070285
C	1.472462	-1.705453	-4.539529
C	-3.458092	0.626856	1.039858
N	-2.698694	1.252256	0.395240
H	-1.977614	1.710766	-0.274476
C	-5.009840	-1.031692	-0.123346
O	-5.548439	-0.119017	-0.844365
C	-5.627116	-1.516453	1.141496
H	-6.304639	-0.771180	1.590112
H	-6.198150	-2.440263	0.892867
H	-4.827659	-1.809234	1.849899
C	-3.899251	-1.799447	-0.738911
H	-3.159543	-2.022164	0.059601
H	-4.335907	-2.807649	-0.943945
C	-3.257804	-1.205179	-1.981084
H	-2.839617	-0.202793	-1.771058
H	-2.431308	-1.857777	-2.314565
H	-3.977875	-1.105855	-2.816990
Si	-6.529998	1.374758	-0.715874
C	-6.695309	1.896374	1.071051
H	-7.032937	2.954479	1.097739
H	-7.429276	1.286115	1.633341
H	-5.713917	1.856392	1.584823
C	-8.152966	0.849187	-1.498069

H	-8.657954	0.067263	-0.895477
H	-8.837436	1.720299	-1.565440
H	-7.997932	0.458262	-2.523680
C	-5.532681	2.543111	-1.772538
H	-6.062330	3.513398	-1.865687
H	-4.537966	2.751891	-1.331550
H	-5.376299	2.125831	-2.787228
C	-4.612799	5.211135	0.660378
C	-5.208537	4.900454	2.901289
C	-5.899824	5.705552	0.650685
H	-3.980556	5.140843	-0.236410
H	-5.173436	4.663998	3.971766
H	-6.463826	6.118109	-0.195340
S	-6.631424	5.615211	2.223628
C	-4.197690	4.740945	1.959326
H	-3.561297	2.805191	3.806957
C	-5.400033	-5.879128	-1.130953
C	-4.417954	-5.082196	0.839600
H	-5.551722	-6.216778	-2.163589
C	-5.688450	-5.376539	1.287864
H	-3.649311	-4.614543	1.472472
H	-6.098292	-5.244104	2.297055
H	-3.938785	-4.666245	-3.207948
C	-4.235976	-5.370229	-0.562560
S	-6.685497	-6.012483	0.017552
H	2.357936	3.441675	5.269797
C	0.336357	-0.001625	5.760965
C	1.442820	1.562397	7.104803
H	-0.030175	-0.566516	4.892676
C	0.552931	1.034872	8.016373
H	2.183487	2.330942	7.365516
H	0.440415	1.282909	9.078994
C	1.329682	0.973219	5.795068
S	-0.444208	-0.182990	7.290216
C	-0.229165	-2.531366	-6.313392
C	-0.935450	-0.970704	-4.724185
C	-1.548841	-2.372663	-6.684663
H	0.472130	-3.185649	-6.849736
H	-0.964132	-0.314029	-3.848257
H	-2.081232	-2.839983	-7.522299
S	-2.364437	-1.237619	-5.656088
C	0.141203	-1.721065	-5.182345
H	1.880881	-3.813424	-4.921580

6-IDPi **5**-TS2-*R*

P	0.895143	-0.672813	1.011833
N	0.062942	0.579828	0.554232
P	-0.440270	1.667249	-0.449392
N	-1.848457	1.265662	-1.170176
S	-2.391135	1.680963	-2.655282
O	-1.721071	2.840614	-3.272874
O	-3.866290	1.574362	-2.661290
C	-1.821522	0.173681	-3.661598
F	-0.498810	-0.004854	-3.533385
F	-2.442423	-0.947540	-3.223092
F	-2.119103	0.347650	-4.953335
O	-0.602828	3.017535	0.473042
C	-0.946097	4.173431	-0.231814
C	0.064209	4.818333	-0.941467
C	-0.288173	5.948945	-1.762560
C	0.650261	6.626490	-2.596886
C	0.261423	7.702041	-3.383215
C	-1.085172	8.152359	-3.377604

C	-2.027076	7.500743	-2.596756	F	0.468330	-1.568930	4.522595
C	-1.664529	6.385555	-1.784037	F	-1.505088	-2.440511	4.174751
C	-2.638829	5.691483	-1.012951	O	1.958028	-1.018369	-0.208951
C	-2.313588	4.587994	-0.231996	C	2.928088	-1.983912	0.075007
H	-3.683076	6.040000	-1.034030	C	3.997911	-1.649787	0.913168
H	-3.078582	7.828160	-2.598537	C	4.897883	-2.704317	1.310181
H	-1.380614	9.008593	-4.002909	C	5.923011	-2.519923	2.285668
H	1.003368	8.205466	-4.021689	C	6.782730	-3.554460	2.626741
H	1.692809	6.280970	-2.621787	C	6.662021	-4.828091	2.010341
C	1.447122	4.257183	-0.940818	C	5.651535	-5.052614	1.089071
C	1.666539	2.962125	-1.419458	C	4.737083	-4.016795	0.734740
C	2.967288	2.457776	-1.752108	C	3.658177	-4.267292	-0.154540
C	4.062084	3.215245	-1.344757	C	2.730316	-3.285375	-0.477545
C	3.908090	4.461259	-0.673331	H	3.546488	-5.274434	-0.584858
C	5.035012	5.195443	-0.196628	H	5.527223	-6.042598	0.623348
C	4.873386	6.415443	0.443887	H	7.357140	-5.637630	2.280284
C	3.571827	6.957363	0.622141	H	7.560492	-3.386401	3.387345
C	2.455890	6.278754	0.151262	H	6.021882	-1.544048	2.780307
C	2.587511	5.017121	-0.501770	C	4.161741	-0.244086	1.389384
H	1.449565	6.696204	0.299675	C	3.094700	0.414006	2.014921
H	3.446866	7.921184	1.138737	C	3.148857	1.777211	2.470427
H	5.751445	6.966009	0.814665	C	4.379898	2.420922	2.335258
H	6.037853	4.764766	-0.339860	C	5.498524	1.821753	1.702374
H	5.078023	2.859021	-1.574353	C	6.721670	2.540875	1.538404
O	0.580277	2.125912	-1.658574	C	7.789171	1.986578	0.849393
N	-0.048443	-1.879750	1.505305	C	7.667816	0.683645	0.296565
S	0.351429	-3.184979	2.381876	C	6.501640	-0.051005	0.462645
O	-0.593609	-4.280416	2.043966	C	5.390058	0.482789	1.181051
O	1.792179	-3.487684	2.506479	H	6.428673	-1.055549	0.026859
C	-0.177424	-2.676493	4.131283	H	8.507111	0.246379	-0.266259
F	0.109348	-3.673819	4.974782	H	8.724394	2.553405	0.724498

H	6.790644	3.555745	1.959956	C	0.869174	4.680915	3.371575
H	4.494986	3.446703	2.713389	H	0.861318	5.773320	3.234672
O	1.932730	-0.313924	2.239629	C	1.930044	3.931763	2.851093
C	-5.313385	2.535632	2.067266	H	2.719301	4.451226	2.290953
C	-4.166831	3.032308	2.695523	C	1.977665	2.524344	3.011238
H	-4.036828	2.906219	3.780434	C	0.897987	1.903631	3.677133
C	-3.171717	3.675392	1.948064	H	0.909496	0.823056	3.851299
H	-2.257344	4.037077	2.437043	C	-0.208907	2.640380	4.148098
C	-3.342069	3.861020	0.561290	Si	-5.706967	-2.969220	-1.623662
C	-4.500752	3.356366	-0.063456	C	-5.622919	-1.139231	-1.947983
H	-4.581839	3.434176	-1.155976	H	-4.611245	-0.761283	-2.177021
C	-5.489211	2.661590	0.669717	H	-6.015587	-0.562458	-1.087443
C	3.500080	-0.889771	-4.415531	H	-6.266983	-0.901513	-2.820200
C	2.482267	0.046889	-4.640280	C	-4.700455	-4.036531	-2.797227
H	1.828052	-0.051407	-5.519970	H	-4.512270	-5.048526	-2.382812
C	2.287783	1.110922	-3.751402	H	-5.261440	-4.157046	-3.747632
H	1.481198	1.833944	-3.934973	H	-3.725220	-3.569272	-3.036142
C	3.127383	1.262582	-2.623206	C	-7.460385	-3.584541	-1.397532
C	4.154237	0.320061	-2.410996	H	-8.005759	-3.543915	-2.362847
H	4.787255	0.408062	-1.516602	H	-7.485516	-4.629850	-1.029836
C	4.344336	-0.770168	-3.288716	H	-8.001563	-2.938876	-0.676676
C	-0.628052	-4.262958	-2.988238	O	-5.067076	-3.268465	0.026950
C	0.175923	-3.159170	-3.297909	C	-3.904428	-3.164737	0.574477
H	-0.046385	-2.547323	-4.184297	C	-3.782978	-3.689305	1.966082
C	1.249587	-2.806599	-2.469777	H	-3.486030	-4.756529	1.802686
H	1.857823	-1.923966	-2.704772	H	-2.893498	-3.236471	2.437380
C	1.556003	-3.592291	-1.337309	C	-5.032981	-3.588818	2.828868
C	0.737026	-4.697943	-1.029112	H	-5.903127	-4.082092	2.353606
H	0.951217	-5.275557	-0.118636	H	-5.283270	-2.520389	2.988332
C	-0.372818	-5.039714	-1.833783	H	-4.857165	-4.059387	3.815087
C	-0.197112	4.046749	4.023585	C	-2.685102	-2.944152	-0.244566

H	-1.844175	-2.546079	0.354968
H	-2.370617	-3.949505	-0.603287
H	-2.867561	-2.304479	-1.125230
C	-3.955437	-0.806947	1.194117
N	-3.456125	0.054836	0.569573
H	-2.873219	0.656481	-0.129631
C	-1.555311	-6.507169	-0.069876
C	-2.089958	-6.850969	-2.323428
C	-2.525673	-7.480872	0.042185
H	-1.069258	-6.029652	0.793851
H	-2.093506	-6.801840	-3.419186
H	-2.920956	-7.943403	0.955331
C	-7.459274	1.063063	0.561654
C	-7.061576	2.323285	-1.361737
H	-7.369020	0.607757	1.554793
C	-8.159143	1.594224	-1.762811
H	-6.559903	3.047873	-2.015573
H	-8.681512	1.627729	-2.726726
C	5.143812	-3.163757	-3.109853
C	6.718358	-1.521012	-2.578071
H	4.200728	-3.663687	-3.364279
C	7.459565	-2.663190	-2.353615
H	7.117501	-0.504357	-2.462298
H	8.506135	-2.745973	-2.035591
C	-1.883226	0.756094	4.145289
C	-2.195669	2.380105	5.798758
H	-1.518586	0.226085	3.257005
C	-3.263008	1.543626	6.059375
H	-1.971944	3.277500	6.392515
H	-4.015188	1.627990	6.853511
H	-1.046782	4.637555	4.399533
H	3.653574	-1.725047	-5.115394
H	-6.075476	2.024322	2.673406
H	-1.496584	-4.499445	-3.621722
S	-3.130802	-7.966491	-1.509931
S	-8.711333	0.532185	-0.506824
S	-3.303273	0.204356	4.956948
S	6.529502	-4.092848	-2.661492
C	-6.644069	2.036417	-0.012221
C	-1.297410	-6.126076	-1.437783
C	-1.386185	1.932371	4.696558
C	5.373897	-1.796194	-3.008751

30-IDPi **5**-TS2-*S*

P	-1.351391	-1.310682	-0.801215
N	-0.543764	0.030158	-0.711955
P	-0.156806	1.406724	-0.048949
N	1.384564	1.669575	0.323426
S	2.174021	1.605518	1.702410
O	3.642316	1.649811	1.440025
O	1.663206	0.628323	2.695439
C	1.899532	3.309459	2.511426
F	0.596337	3.598835	2.639788
F	2.461656	3.292964	3.729925
F	2.484586	4.273086	1.776186
O	-0.569065	2.524495	-1.185153
C	-0.417334	3.850222	-0.782633
C	-1.401545	4.390045	0.044498
C	-1.217985	5.724302	0.559644
C	-2.098448	6.319960	1.512041
C	-1.879557	7.607780	1.980852
C	-0.769729	8.365908	1.522328
C	0.120955	7.806494	0.619548

C	-0.065150	6.478491	0.130588
C	0.885635	5.873746	-0.736640
C	0.755623	4.562012	-1.184211
H	1.767886	6.456636	-1.043116
H	0.999360	8.374178	0.274287
H	-0.610638	9.388706	1.896424
H	-2.570073	8.041747	2.720014
H	-2.955328	5.742932	1.885320
C	-2.566228	3.557043	0.458737
C	-2.362934	2.354955	1.149658
C	-3.421952	1.671071	1.835463
C	-4.722262	2.111412	1.594332
C	-5.007249	3.221100	0.753037
C	-6.349099	3.618744	0.473140
C	-6.613898	4.719327	-0.328185
C	-5.539418	5.473483	-0.873014
C	-4.223554	5.121256	-0.605400
C	-3.916767	3.988074	0.207082
H	-3.399081	5.702260	-1.041951
H	-5.751210	6.341903	-1.515258
H	-7.653302	5.011227	-0.542875
H	-7.169878	3.022033	0.899740
H	-5.560122	1.612475	2.104050
O	-1.081266	1.832826	1.264579
N	-0.376375	-2.522953	-1.265983
S	-0.702087	-3.971698	-1.946390
O	0.418478	-4.882862	-1.623565
O	-2.100878	-4.428010	-1.850827
C	-0.450696	-3.597002	-3.785486
F	-1.387387	-2.745581	-4.230203
F	0.765167	-3.045897	-3.988539
F	-0.525987	-4.736044	-4.481878
O	-2.114599	-1.552565	0.637757
C	-2.835727	-2.751392	0.695341
C	-4.065989	-2.817403	0.039486
C	-4.717895	-4.099954	-0.044887
C	-5.903277	-4.315300	-0.808236
C	-6.494315	-5.569210	-0.874687
C	-5.928514	-6.671404	-0.181182
C	-4.761120	-6.502253	0.546911
C	-4.118610	-5.230699	0.623337
C	-2.888720	-5.071301	1.321162
C	-2.217873	-3.854676	1.359608
H	-2.445882	-5.942201	1.828727
H	-4.298343	-7.355009	1.067978
H	-6.408076	-7.660530	-0.237106
H	-7.404265	-5.710813	-1.477616
H	-6.342320	-3.474482	-1.362652
C	-4.633468	-1.584632	-0.581052
C	-3.878601	-0.840497	-1.498092
C	-4.345075	0.365847	-2.123397
C	-5.653456	0.749968	-1.827241
C	-6.462735	0.062187	-0.886373
C	-7.770724	0.531973	-0.557697
C	-8.521776	-0.082959	0.431931
C	-7.987663	-1.197516	1.133046
C	-6.732251	-1.697201	0.814728
C	-5.940154	-1.099740	-0.213142
H	-6.333581	-2.561329	1.363554
H	-8.574044	-1.671198	1.934990
H	-9.524978	0.294210	0.682531
H	-8.161319	1.408991	-1.096821

H	-6.075721	1.639997	-2.315815	H	-3.121811	4.578711	-3.703754
O	-2.618247	-1.309499	-1.851908	C	-3.711980	2.631725	-2.967896
C	3.975707	2.738510	-3.385025	H	-4.484265	3.057317	-2.314304
C	2.657923	2.597032	-3.844767	C	-3.488346	1.233284	-2.981097
H	2.453836	2.023152	-4.761025	C	-2.452303	0.740156	-3.804243
C	1.590172	3.166461	-3.136896	H	-2.278880	-0.339110	-3.877724
H	0.560210	3.031619	-3.491265	C	-1.637644	1.604234	-4.566929
C	1.839737	3.913247	-1.966084	C	2.914754	-1.488763	0.377060
C	3.165707	4.042867	-1.510847	N	1.965068	-1.989406	-0.102740
H	3.354602	4.595388	-0.580274	H	1.061979	-2.320730	-0.579132
C	4.247371	3.453589	-2.194961	C	5.390213	-1.158114	-0.675467
C	-2.949797	-1.040339	5.140895	O	6.003423	-1.687345	0.328410
C	-3.966271	-1.249799	4.198387	C	5.295299	0.312633	-0.788722
H	-4.671874	-2.084512	4.329616	H	4.630995	0.705366	0.019598
C	-4.082604	-0.414262	3.080442	H	6.291970	0.769453	-0.630146
H	-4.882531	-0.586590	2.346932	H	4.880493	0.660763	-1.745852
C	-3.172418	0.649270	2.887186	Si	6.392832	-1.257318	2.023930
C	-2.126869	0.824076	3.819358	C	4.909983	-1.817271	3.003952
H	-1.414899	1.649958	3.686133	H	5.169108	-1.809318	4.084036
C	-2.005284	-0.003285	4.957426	H	4.041460	-1.151447	2.834153
C	1.593522	-3.397621	3.298059	H	4.622924	-2.848118	2.715895
C	0.544747	-2.520780	3.599026	C	6.757438	0.566802	2.219004
H	0.692925	-1.710653	4.327016	H	5.825421	1.158788	2.121634
C	-0.687471	-2.650483	2.949510	H	7.153866	0.719027	3.245768
H	-1.499549	-1.947953	3.167814	H	7.513729	0.952116	1.507085
C	-0.895330	-3.692041	2.020126	C	7.898728	-2.348653	2.271086
C	0.167037	-4.566490	1.719577	H	8.268630	-2.255119	3.312984
H	0.018597	-5.349721	0.961983	H	7.642065	-3.412662	2.093194
C	1.431777	-4.413809	2.330437	H	8.726360	-2.067722	1.588939
C	-1.897409	2.992089	-4.542970	C	3.707594	-5.565603	2.760879
C	-2.933452	3.495003	-3.745879	C	2.742567	-5.767253	0.638497

C	4.675139	-6.312638	2.121880
H	3.774064	-5.279099	3.819758
H	2.079394	-5.627051	-0.227352
H	5.605544	-6.721498	2.535139
S	4.226669	-6.643654	0.478597
C	2.583891	-5.243125	1.917171
H	2.569945	-3.251776	3.780568
C	5.887833	3.609104	-0.278098
C	6.826862	3.514402	-2.419082
H	5.166337	3.548221	0.547553
C	7.968953	3.562897	-1.647545
H	6.845192	3.491411	-3.517536
H	9.014558	3.589818	-1.978488
H	4.800651	2.263504	-3.939441
C	5.615097	3.540414	-1.641119
S	7.586187	3.641149	0.043376
H	-2.856194	-1.714645	6.005082
C	0.350798	0.690841	5.595872
C	-1.008114	-0.080409	7.338790
H	0.749460	0.923397	4.598211
C	0.155771	0.186796	8.028411
H	-1.924056	-0.443908	7.824824
H	0.348555	0.082783	9.103312
C	-0.911091	0.206449	5.930800
S	1.392245	0.783853	6.970079
C	-0.053720	1.574551	-6.615462
C	0.277899	-0.005327	-4.923265
C	1.031056	0.892033	-7.124052
H	-0.544255	2.403753	-7.143948
H	0.217637	-0.545131	-3.971609
H	1.551352	1.049003	-8.076812
S	1.521707	-0.388782	-6.060796
C	-0.505269	1.061299	-5.347796
H	-1.263065	3.679711	-5.123209
C	4.918742	-2.080386	-1.710706
C	4.992902	-3.479543	-1.484425
C	4.302551	-1.605038	-2.897207
C	4.448872	-4.371680	-2.408545
H	5.438389	-3.852381	-0.551739
C	3.761672	-2.505806	-3.817056
H	4.209949	-0.527286	-3.090117
C	3.820713	-3.887975	-3.569930
H	4.487945	-5.451871	-2.206376
H	3.265379	-2.125926	-4.720918
H	3.357571	-4.590376	-4.279171

30-IDPi **5**-TS2-*R*

P	0.852014	-0.817042	0.918020
N	0.194119	0.549639	0.495024
P	0.013932	1.781277	-0.452507
N	-1.371089	1.709723	-1.313474
S	-1.683325	2.253827	-2.824562
O	-0.730522	3.252505	-3.342691
O	-3.141401	2.462563	-2.949522
C	-1.361840	0.668757	-3.820895
F	-0.082722	0.287980	-3.691375
F	-2.150995	-0.333221	-3.368929
F	-1.630565	0.875412	-5.114417
O	0.007021	3.078026	0.554759
C	-0.115708	4.308324	-0.097891
C	1.026373	4.827641	-0.702902
C	0.903039	6.036203	-1.478951
C	1.988533	6.608852	-2.206756

C	1.817454	7.766539	-2.952933	C	-0.468957	-2.797055	3.936804
C	0.552240	8.407939	-3.013209	F	-0.309944	-3.871707	4.719938
C	-0.529164	7.863869	-2.338018	F	0.329783	-1.813835	4.373643
C	-0.391338	6.670176	-1.568746	F	-1.748385	-2.387034	4.022931
C	-1.510014	6.091943	-0.905846	O	1.915983	-1.222963	-0.281197
C	-1.407029	4.919079	-0.165660	C	2.738214	-2.325073	-0.024213
H	-2.488477	6.591784	-0.976782	C	3.805581	-2.190145	0.870801
H	-1.520174	8.341226	-2.391573	C	4.531263	-3.383671	1.233728
H	0.430000	9.327321	-3.605791	C	5.524150	-3.404297	2.258814
H	2.670064	8.186249	-3.508323	C	6.215351	-4.567803	2.565802
H	2.970761	6.117855	-2.181581	C	5.949028	-5.773498	1.864873
C	2.308301	4.063399	-0.657999	C	4.960358	-5.799207	0.894280
C	2.369958	2.779839	-1.209653	C	4.215157	-4.626241	0.572484
C	3.603412	2.119868	-1.525562	C	3.151189	-4.672651	-0.367866
C	4.770766	2.688000	-1.023206	C	2.384463	-3.552854	-0.660491
C	4.757200	3.897691	-0.272500	H	2.915435	-5.629070	-0.859607
C	5.949699	4.434498	0.297787	H	4.720856	-6.733299	0.362763
C	5.927382	5.623518	1.012284	H	6.511858	-6.687482	2.108386
C	4.705741	6.331185	1.174796	H	6.971410	-4.555565	3.365721
C	3.531382	5.846888	0.614232	H	5.733513	-2.483740	2.820156
C	3.519378	4.622678	-0.118263	C	4.135809	-0.853703	1.450497
H	2.586262	6.392141	0.750478	C	3.136040	-0.094993	2.074614
H	4.689296	7.269113	1.750549	C	3.352117	1.209977	2.640561
H	6.853955	6.021060	1.453887	C	4.666974	1.677445	2.617116
H	6.888912	3.875925	0.165536	C	5.727578	0.968174	1.998294
H	5.737793	2.206823	-1.235073	C	7.049076	1.508916	1.963163
O	1.197186	2.113327	-1.545690	C	8.071213	0.854593	1.293585
N	-0.258539	-1.911042	1.321603	C	7.802598	-0.371824	0.628720
S	-0.045883	-3.286701	2.151437	C	6.534368	-0.935272	0.666300
O	-1.149052	-4.219129	1.792636	C	5.464592	-0.298775	1.364416
O	1.332062	-3.813253	2.238083	H	6.348303	-1.882204	0.143532

H	8.606636	-0.886128	0.079765	C	-0.920389	-4.695356	-2.192338
H	9.084573	1.283694	1.270552	C	0.262055	3.815997	4.200260
H	7.233532	2.468764	2.469956	C	1.454361	4.336209	3.678600
H	4.902596	2.644915	3.082798	H	1.611405	5.425312	3.642216
O	1.878590	-0.668382	2.199231	C	2.432425	3.483990	3.153975
C	-4.834141	3.269600	1.845252	H	3.323300	3.925517	2.686887
C	-3.658360	3.526351	2.557370	C	2.266722	2.076618	3.183708
H	-3.614029	3.316089	3.635841	C	1.062146	1.571580	3.721684
C	-2.530664	4.036629	1.901369	H	0.906642	0.491244	3.792690
H	-1.600106	4.214153	2.456739	C	0.041197	2.421826	4.194849
C	-2.586291	4.326524	0.522596	Si	-5.885475	-1.716279	-2.436445
C	-3.773642	4.057195	-0.189686	C	-5.427207	0.072890	-2.668569
H	-3.777683	4.197169	-1.279759	H	-4.341128	0.264623	-2.634966
C	-4.904345	3.503872	0.452658	H	-5.907911	0.707529	-1.899489
C	3.792065	-1.125129	-4.358095	H	-5.793151	0.413749	-3.659503
C	2.925229	-0.044426	-4.569421	C	-4.857919	-2.924955	-3.442076
H	2.304195	-0.006913	-5.477340	H	-4.927876	-3.961789	-3.054586
C	2.836787	0.990814	-3.630793	H	-5.234140	-2.926410	-4.486805
H	2.143712	1.825777	-3.803549	H	-3.790530	-2.631326	-3.468506
C	3.636971	0.966322	-2.464382	C	-7.726228	-2.021130	-2.611732
C	4.514908	-0.118771	-2.268213	H	-8.037891	-1.886082	-3.667987
H	5.117364	-0.162960	-1.350486	H	-8.001918	-3.048488	-2.299780
C	4.592723	-1.180770	-3.195461	H	-8.295281	-1.295895	-1.995078
C	-0.980032	-3.853803	-3.327592	O	-5.686159	-2.098974	-0.708080
C	0.021439	-2.905291	-3.568746	C	-4.708586	-2.285351	0.127617
H	-0.048113	-2.242490	-4.443633	C	-3.339138	-2.599312	-0.384259
C	1.103587	-2.775242	-2.688905	H	-2.536867	-2.421785	0.353664
H	1.872192	-2.012548	-2.869366	H	-3.326402	-3.684974	-0.632587
C	1.211487	-3.631849	-1.572609	H	-3.104891	-2.032023	-1.301671
C	0.194893	-4.578168	-1.331735	C	-4.092425	-0.101738	0.538169
H	0.265537	-5.210386	-0.435012	N	-3.320694	0.654413	0.080132

H	-2.531549	1.190560	-0.487461
C	-2.385687	-6.049149	-0.553331
C	-2.892081	-6.166040	-2.835798
C	-3.502566	-6.857200	-0.526827
H	-1.861059	-5.710336	0.352013
H	-2.849748	-6.054792	-3.926079
H	-4.002385	-7.294111	0.346618
C	-7.041811	2.182642	0.114346
C	-6.465737	3.646168	-1.611146
H	-7.015089	1.579203	1.029534
C	-7.636254	3.119302	-2.111312
H	-5.873747	4.399542	-2.147049
H	-8.137733	3.353276	-3.058304
C	5.041955	-3.668617	-3.111209
C	6.805442	-2.292059	-2.436039
H	4.051009	-4.017242	-3.427887
C	7.368195	-3.537053	-2.241585
H	7.337312	-1.348445	-2.253987
H	8.377330	-3.782154	-1.888178
C	-2.036129	2.291447	5.667779
C	-1.904057	0.771523	3.893365
H	-1.790898	3.071193	6.399435
C	-3.131215	0.433204	4.420086
H	-1.488282	0.301804	2.991765
H	-3.836328	-0.326168	4.058343
H	-0.523988	4.491964	4.570959
H	3.859945	-1.937330	-5.097602
H	-5.708942	2.868309	2.378044
H	-1.842820	-3.918799	-4.007541
S	-4.125276	-7.144646	-2.120472
S	-8.331666	1.964320	-1.018202
S	-3.522756	1.411303	5.798396
S	6.261916	-4.804569	-2.657797
C	-6.108715	3.122686	-0.316765
C	-2.024678	-5.631620	-1.886823
C	-1.258687	1.844687	4.605031
C	5.456530	-2.353670	-2.931800
C	-5.125872	-2.653244	1.498601
C	-6.464219	-2.430127	1.900944
C	-4.209533	-3.228227	2.405639
C	-6.872641	-2.761968	3.194860
H	-7.166645	-1.976940	1.187109
C	-4.629148	-3.564275	3.700674
H	-3.165975	-3.418953	2.116711
C	-5.953811	-3.328181	4.099950
H	-7.911418	-2.576154	3.507494
H	-3.903687	-4.002335	4.401503
H	-6.275385	-3.583670	5.121491

30-IDPi **2**-TS2-*S*

P	-1.069202	-0.934564	-1.420404
N	-1.651706	0.194472	-0.476296
P	-1.437200	1.137234	0.760301
N	0.066301	1.386124	1.374112
S	0.714581	0.369611	2.469092
O	2.190578	0.372270	2.336443
O	-0.013585	-0.911834	2.591308
C	0.421911	1.264107	4.121468
F	1.055583	0.582882	5.090353
F	0.918331	2.513646	4.077556
F	-0.883054	1.325305	4.410147
O	-2.022855	2.599576	0.303104
C	-2.478025	3.478417	1.289037

C	-3.684206	3.198501	1.940513	S	1.042675	-0.681952	-3.390791
C	-4.122509	4.085654	2.990565	O	0.075516	-0.339162	-4.458314
C	-5.278395	3.830467	3.789902	O	2.334378	0.055409	-3.345663
C	-5.665406	4.709146	4.791829	C	1.575328	-2.440204	-3.835777
C	-4.915425	5.886765	5.051608	F	0.533225	-3.279788	-3.816233
C	-3.770250	6.149241	4.316283	F	2.112139	-2.433291	-5.065812
C	-3.336992	5.259390	3.287731	F	2.510639	-2.879481	-2.968318
C	-2.128368	5.489864	2.574583	O	-1.351067	-2.351517	-0.626607
C	-1.670780	4.618676	1.591220	C	-1.403670	-3.551339	-1.326310
H	-1.527372	6.380554	2.815214	C	-2.486429	-3.787037	-2.189136
H	-3.165855	7.046853	4.521371	C	-2.483859	-4.993274	-2.978851
H	-5.236384	6.579467	5.844436	C	-3.431460	-5.239477	-4.017626
H	-6.559357	4.486421	5.394179	C	-3.409663	-6.423872	-4.740596
H	-5.864628	2.918880	3.612296	C	-2.441757	-7.424839	-4.458938
C	-4.428508	1.939515	1.646222	C	-1.488761	-7.204891	-3.476848
C	-3.771032	0.717728	1.814373	C	-1.468908	-5.986651	-2.732121
C	-4.424683	-0.553725	1.752015	C	-0.452697	-5.721039	-1.776703
C	-5.760146	-0.552731	1.354140	C	-0.354705	-4.505885	-1.098238
C	-6.456415	0.647018	1.039893	H	0.302128	-6.499176	-1.586467
C	-7.796382	0.618189	0.547848	H	-0.716448	-7.961424	-3.265837
C	-8.463632	1.790295	0.224415	H	-2.440448	-8.364503	-5.032099
C	-7.816234	3.044425	0.391497	H	-4.145491	-6.585270	-5.543060
C	-6.518788	3.108476	0.880979	H	-4.180084	-4.470154	-4.252161
C	-5.800618	1.921448	1.214349	C	-3.615433	-2.812843	-2.283046
H	-6.020413	4.081536	0.996033	C	-3.352666	-1.466363	-2.550051
H	-8.344924	3.971965	0.124158	C	-4.355518	-0.443572	-2.629166
H	-9.492522	1.752754	-0.164662	C	-5.674511	-0.862065	-2.455911
H	-8.283727	-0.360910	0.417757	C	-6.017362	-2.204816	-2.143002
H	-6.296300	-1.511177	1.278255	C	-7.373100	-2.570720	-1.881317
O	-2.395892	0.743183	2.045973	C	-7.704405	-3.858856	-1.491042
N	0.459425	-0.803792	-1.903054	C	-6.683698	-4.835960	-1.348774

C	-5.359368	-4.518994	-1.618304	H	2.942721	2.938999	2.398769
C	-4.982805	-3.205944	-2.034658	C	4.537874	5.507453	3.897790
H	-4.582613	-5.285332	-1.493806	H	2.663893	6.398275	3.260582
H	-6.941963	-5.853002	-1.016928	H	4.060801	7.073325	2.377960
H	-8.752055	-4.123660	-1.281883	H	3.021956	4.082884	4.541050
H	-8.150700	-1.797044	-1.981339	H	4.657348	3.371145	4.509535
H	-6.486214	-0.123265	-2.533104	H	4.512456	6.059526	4.859745
O	-2.026721	-1.102682	-2.742697	H	5.598209	5.483475	3.562693
C	6.341974	5.458654	-0.821202	H	7.440687	5.484539	-0.893268
C	5.562713	5.638573	-1.979620	C	0.520305	-6.844220	4.305930
H	6.056696	5.802843	-2.949995	C	-0.096604	-7.268050	3.115171
C	4.159527	5.609674	-1.905711	H	0.210171	-8.216822	2.648551
H	3.547930	5.741065	-2.811883	C	-1.084851	-6.477236	2.507443
C	3.549235	5.400337	-0.657826	H	-1.545556	-6.786660	1.556395
C	2.134731	5.289276	-0.288597	C	-1.448050	-5.263389	3.113059
C	0.971481	5.309008	-1.073654	C	-2.356547	-4.209906	2.652184
H	1.018173	5.502408	-2.155392	C	-3.188927	-4.137859	1.524513
C	-0.267990	5.064999	-0.466363	H	-3.283909	-4.988551	0.832974
H	-1.180476	5.056687	-1.077756	C	-3.874490	-2.945556	1.267106
C	-0.358914	4.846691	0.926948	H	-4.503929	-2.864749	0.372576
C	0.814777	4.862800	1.716487	C	-3.717281	-1.812190	2.096531
H	0.737151	4.661914	2.795367	C	-2.908998	-1.910192	3.256574
C	2.055083	5.062004	1.107495	H	-2.791675	-1.036434	3.914358
C	4.332327	5.222759	0.513871	C	-2.255713	-3.109380	3.542374
C	5.729211	5.250327	0.430161	C	-0.838261	-4.835278	4.323687
H	6.348279	5.104256	1.328433	C	0.154060	-5.627644	4.914853
C	3.440871	5.022946	1.738057	H	0.667296	-5.304537	5.832455
C	3.748121	3.685779	2.516898	C	-1.407241	-3.482229	4.753471
C	3.650716	6.143713	2.819783	C	-2.294598	-3.609965	6.036319
C	3.984253	4.079959	3.985209	C	-0.349828	-2.451726	5.227452
H	4.659763	3.225411	2.092029	C	-1.297445	-3.614226	7.204885

H	-2.956600	-4.498156	6.001923
H	-2.947027	-2.711799	6.083761
C	-0.119156	-2.716009	6.737094
H	-0.767413	-1.438706	5.074301
H	0.571766	-2.498308	4.619036
H	-1.761386	-3.262365	8.148461
H	-0.946447	-4.649336	7.394906
H	-0.082072	-1.758380	7.295138
H	0.856547	-3.211433	6.920111
H	1.308025	-7.463229	4.762401
C	7.203480	-3.510522	2.484134
C	6.276929	-2.908109	3.354810
H	6.633532	-2.414825	4.272103
C	4.903623	-2.922829	3.058396
H	4.176140	-2.446370	3.734389
C	4.470835	-3.552169	1.878786
C	3.129183	-3.705221	1.307976
C	1.880308	-3.239107	1.742680
H	1.778202	-2.648732	2.662882
C	0.741982	-3.494946	0.968240
H	-0.225588	-3.114531	1.314442
C	0.828453	-4.224157	-0.243021
C	2.099023	-4.702098	-0.661180
H	2.186803	-5.233703	-1.620661
C	3.236865	-4.437931	0.099470
C	5.402757	-4.173364	1.005125
C	6.769257	-4.147436	1.304779
H	7.503405	-4.619235	0.631998
C	4.689824	-4.814209	-0.183920
C	5.226368	-4.300405	-1.573381
C	4.900720	-6.375063	-0.254743
C	5.400562	-5.549616	-2.451369
H	6.212700	-3.813773	-1.420085
H	4.553886	-3.545760	-2.020574
C	5.839979	-6.620175	-1.445002
H	3.919982	-6.855364	-0.451318
H	5.273794	-6.781685	0.705480
H	4.426549	-5.836513	-2.905084
H	6.114853	-5.395401	-3.285745
H	5.779127	-7.656097	-1.836729
H	6.897700	-6.444945	-1.148431
H	8.276848	-3.485432	2.727206
C	-1.310865	7.346245	-3.713676
C	-2.442513	7.466931	-2.885294
H	-2.721659	8.453239	-2.484031
C	-3.214813	6.338480	-2.564290
H	-4.095847	6.431755	-1.910425
C	-2.835315	5.086547	-3.079399
C	-3.410566	3.751902	-2.888369
C	-4.513082	3.317227	-2.137129
H	-5.127851	4.026536	-1.562602
C	-4.804459	1.950236	-2.092062
H	-5.636103	1.607516	-1.461990
C	-4.020072	0.995772	-2.789108
C	-2.923984	1.452008	-3.567173
H	-2.299076	0.736302	-4.118335
C	-2.623717	2.815154	-3.605052
C	-1.693853	4.961923	-3.915703
C	-0.934526	6.093430	-4.235955
H	-0.048950	6.008669	-4.886054
C	-1.470234	3.509671	-4.322440
C	-1.475154	3.310660	-5.886673

C	-0.066295	2.956895	-3.874640
C	-0.244953	2.447348	-6.212280
H	-1.385586	4.303855	-6.373568
H	-2.429588	2.868387	-6.234286
C	0.781182	2.861486	-5.150552
H	-0.204606	1.942478	-3.457946
H	0.387336	3.569677	-3.073757
H	-0.475022	1.370985	-6.076846
H	0.109365	2.586433	-7.254506
H	1.609231	2.133978	-5.039758
H	1.227834	3.849690	-5.403126
H	-0.713753	8.239423	-3.953882
Si	5.769253	1.011021	-3.413897
C	4.505035	2.322729	-3.806514
H	3.489282	1.966796	-3.550973
H	4.720061	3.251688	-3.242560
H	4.533851	2.547275	-4.893410
C	5.432515	-0.693717	-4.117157
H	5.994084	-1.480136	-3.572937
H	5.762029	-0.715724	-5.177048
H	4.350983	-0.925230	-4.088655
C	7.528153	1.578001	-3.750367
H	7.688552	1.713180	-4.840032
H	8.272426	0.842050	-3.384414
H	7.722259	2.550487	-3.253482
O	5.838096	0.924331	-1.642185
C	5.143974	0.515408	-0.629003
C	4.227515	-0.659638	-0.764972
H	4.864116	-1.571061	-0.718728
H	3.682043	-0.640707	-1.725117
H	3.492575	-0.723230	0.056128
C	3.284204	1.948365	-0.624826
N	2.199776	2.037507	-0.183699
H	1.280276	1.927061	0.356111
C	5.687718	0.920429	0.687379
C	6.745878	1.860846	0.740637
C	5.204371	0.353202	1.887620
C	7.316956	2.209170	1.967225
H	7.099658	2.316848	-0.193689
C	5.779049	0.710186	3.113400
H	4.364734	-0.350743	1.877894
C	6.837026	1.631174	3.158080
H	8.138232	2.941025	2.000491
H	5.385210	0.267738	4.039984
H	7.284285	1.911243	4.124329

30-IDPi **2**-TS2-*R*

P	-0.171474	1.585014	-0.772905
N	0.362612	0.850195	0.504497
P	1.218096	-0.244943	1.248399
N	0.515833	-1.651240	1.576883
S	0.009329	-2.275106	2.956862
O	-0.897195	-3.421445	2.667657
O	-0.402328	-1.299168	3.997182
C	1.512120	-3.140079	3.730756
F	1.150636	-3.631676	4.923959
F	1.929363	-4.157561	2.954962
F	2.523649	-2.278766	3.899635
O	2.581417	-0.466363	0.347942
C	3.571967	-1.247476	0.941296
C	4.377970	-0.663296	1.926322
C	5.324616	-1.503788	2.618438
C	6.095546	-1.054363	3.731831

C	7.010246	-1.891504	4.355780
C	7.198554	-3.223399	3.899875
C	6.434885	-3.705238	2.848195
C	5.470412	-2.877166	2.198661
C	4.639697	-3.391000	1.167146
C	3.666664	-2.617932	0.538274
H	4.766377	-4.441949	0.865272
H	6.549902	-4.744743	2.503027
H	7.934938	-3.874985	4.394405
H	7.589064	-1.520303	5.215182
H	5.951588	-0.030882	4.103576
C	4.197133	0.778235	2.271069
C	2.922995	1.253532	2.609739
C	2.643254	2.619600	2.955562
C	3.738511	3.482795	3.004152
C	5.052235	3.075372	2.652589
C	6.137465	4.004100	2.663288
C	7.402191	3.634086	2.232394
C	7.629929	2.308299	1.775677
C	6.604242	1.372754	1.788935
C	5.293018	1.715169	2.238437
H	6.800593	0.350543	1.441096
H	8.627136	2.014670	1.413125
H	8.226118	4.363859	2.236459
H	5.939421	5.031968	3.004195
H	3.583767	4.529576	3.304399
O	1.876010	0.341645	2.641709
N	-1.290177	0.814738	-1.685038
S	-1.105374	0.149967	-3.162521
O	0.290459	-0.178103	-3.528400
O	-2.173449	-0.856313	-3.349378
C	-1.550836	1.567109	-4.354270
F	-0.945735	2.706946	-3.992271
F	-1.151679	1.227670	-5.584917
F	-2.882659	1.766602	-4.371204
O	-0.853889	2.951027	-0.168436
C	-1.092753	4.011545	-1.042797
C	0.001965	4.743395	-1.506470
C	-0.224633	5.789387	-2.475163
C	0.834065	6.514692	-3.101231
C	0.568755	7.509674	-4.031738
C	-0.767951	7.831112	-4.387160
C	-1.818444	7.126821	-3.820483
C	-1.579869	6.087917	-2.871364
C	-2.650887	5.326675	-2.329460
C	-2.442270	4.284586	-1.431115
H	-3.681353	5.565972	-2.634913
H	-2.859455	7.350144	-4.102504
H	-0.965686	8.627039	-5.121198
H	1.404592	8.049702	-4.502095
H	1.874801	6.272020	-2.848349
C	1.392262	4.375782	-1.108119
C	1.879988	3.100394	-1.408379
C	3.272194	2.754285	-1.376863
C	4.129355	3.708100	-0.822945
C	3.669792	4.962937	-0.337164
C	4.559819	5.897301	0.273271
C	4.101094	7.119856	0.740370
C	2.728179	7.463133	0.606163
C	1.841910	6.589034	-0.008348
C	2.283367	5.323078	-0.496185
H	0.779642	6.855404	-0.107339

H	2.365194	8.428116	0.991413
H	4.797895	7.825811	1.217786
H	5.618682	5.616851	0.377699
H	5.209536	3.502703	-0.786512
O	0.967529	2.133870	-1.828968
C	-2.356104	-6.891443	-3.470572
C	-2.192780	-5.689169	-4.183672
H	-2.820151	-5.488560	-5.066102
C	-1.236677	-4.741019	-3.779802
H	-1.121197	-3.790140	-4.322603
C	-0.437522	-5.022199	-2.657911
C	0.654913	-4.261036	-2.043301
C	1.229750	-3.024710	-2.379062
H	0.877957	-2.439130	-3.239824
C	2.245328	-2.503869	-1.567435
H	2.680947	-1.530051	-1.825225
C	2.698605	-3.203972	-0.421003
C	2.151181	-4.477627	-0.132669
H	2.486627	-5.019145	0.762666
C	1.134570	-4.995391	-0.931302
C	-0.617252	-6.224195	-1.920139
C	-1.577500	-7.157913	-2.325489
H	-1.719082	-8.097864	-1.767423
C	0.358689	-6.294180	-0.749061
C	-0.357693	-6.367755	0.652621
C	1.281840	-7.571368	-0.771461
C	0.439053	-7.391084	1.474333
H	-1.392131	-6.745252	0.505086
H	-0.425023	-5.373389	1.135437
C	0.846862	-8.432793	0.425427
H	2.335451	-7.250380	-0.638391
H	1.227880	-8.098884	-1.743963
H	1.341218	-6.910631	1.911441
H	-0.143433	-7.809548	2.320419
H	1.640839	-9.128396	0.766337
H	-0.035683	-9.054185	0.154598
H	-3.102227	-7.628571	-3.805721
C	-5.495352	4.298472	4.420577
C	-4.937063	5.476256	3.891317
H	-5.567129	6.369866	3.763869
C	-3.578106	5.524068	3.540065
H	-3.136775	6.451492	3.143033
C	-2.789915	4.372950	3.708810
C	-1.362140	4.141962	3.461688
C	-0.355920	4.961239	2.921547
H	-0.574453	5.986600	2.584549
C	0.935735	4.441674	2.775144
H	1.703549	5.064169	2.294724
C	1.262700	3.120746	3.182306
C	0.241783	2.314923	3.746409
H	0.452535	1.289244	4.074078
C	-1.054354	2.815178	3.850995
C	-3.359365	3.167207	4.208578
C	-4.709014	3.140743	4.584815
H	-5.156589	2.230835	5.012355
C	-2.314650	2.055560	4.226315
C	-2.291993	1.194891	5.520339
C	-2.604961	0.971332	3.134427
C	-3.225567	-0.024722	5.262682
H	-2.581184	1.793072	6.407325
H	-1.255597	0.841124	5.686463
C	-3.643682	0.053170	3.773548

H	-1.667726	0.406664	2.970384	C	-7.549395	1.428794	-4.504949
H	-2.905003	1.406392	2.164542	H	-6.828506	3.150441	-3.395136
H	-2.666442	-0.963105	5.446853	H	-8.352254	2.511141	-2.725948
H	-4.106041	-0.032904	5.938377	H	-5.424581	1.495692	-4.978925
H	-3.667369	-0.936881	3.279268	H	-6.166388	-0.069169	-5.401498
H	-4.662728	0.481503	3.663857	H	-7.826991	2.072178	-5.364877
H	-6.557105	4.281285	4.711368	H	-8.346697	0.659458	-4.403849
C	-9.077222	-0.717664	-0.104539	H	-9.950999	-1.381897	-0.190799
C	-8.577343	-0.381198	1.167361	C	8.038648	-3.232922	-4.852539
H	-9.065220	-0.784820	2.068625	C	6.888086	-3.128385	-5.655168
C	-7.468455	0.472705	1.297294	H	6.813408	-3.717120	-6.582430
H	-7.074277	0.733304	2.291731	C	5.838775	-2.271885	-5.285164
C	-6.868729	0.987093	0.135392	H	4.941576	-2.179663	-5.916515
C	-5.728325	1.893001	-0.038209	C	5.955332	-1.527926	-4.099138
C	-4.890120	2.517297	0.898398	C	5.055480	-0.535647	-3.503487
H	-5.061001	2.407390	1.978584	C	3.824093	-0.017724	-3.935402
C	-3.808335	3.281685	0.441466	H	3.335365	-0.393391	-4.846921
H	-3.132428	3.751559	1.168539	C	3.217329	1.010473	-3.203724
C	-3.581082	3.467854	-0.940760	H	2.266664	1.418104	-3.565544
C	-4.467179	2.882467	-1.875673	C	3.813148	1.530763	-2.025364
H	-4.286378	3.026491	-2.950249	C	5.024638	0.952260	-1.566766
C	-5.519397	2.084808	-1.425870	H	5.476159	1.309874	-0.629448
C	-7.358787	0.634969	-1.150754	C	5.643774	-0.056623	-2.305980
C	-8.466632	-0.211532	-1.270089	C	7.107123	-1.646265	-3.273362
H	-8.864480	-0.480790	-2.261657	C	8.153360	-2.493963	-3.658815
C	-6.533969	1.301782	-2.249141	H	9.063539	-2.586007	-3.048208
C	-5.864155	0.262520	-3.230888	C	6.954893	-0.780095	-2.023404
C	-7.393640	2.216076	-3.196344	C	6.890390	-1.655160	-0.736532
C	-6.181998	0.750518	-4.654508	C	8.161635	0.149647	-1.712176
H	-6.320569	-0.734180	-3.063544	C	8.350933	-1.903399	-0.325123
H	-4.777521	0.157765	-3.046045	H	6.304090	-2.579466	-0.894967

H	6.358065	-1.075725	0.043384	H	-7.551592	-2.643432	-1.741934
C	9.153907	-0.684573	-0.863662	O	-5.475009	-3.686600	0.095521
H	7.785584	1.009770	-1.120622	C	-4.508512	-3.605146	0.951843
H	8.608787	0.566712	-2.636285	C	-3.158301	-4.144818	0.620747
H	8.443561	-2.031625	0.772705	H	-3.162140	-5.226491	0.881826
H	8.722059	-2.844700	-0.779366	H	-2.929941	-4.055620	-0.455443
H	9.577528	-0.073309	-0.040167	H	-2.349081	-3.671906	1.209107
H	10.020617	-1.017899	-1.470390	C	-3.735123	-1.315364	0.368034
H	8.859590	-3.899072	-5.159877	N	-3.087427	-0.543668	-0.235645
Si	-5.585735	-4.153633	-1.628065	H	-2.405141	0.066170	-0.823867
C	-4.378643	-3.147308	-2.632442	C	-4.912239	-3.326723	2.338383
H	-3.331993	-3.497302	-2.555733	C	-4.018414	-3.541031	3.415692
H	-4.398431	-2.077735	-2.353109	C	-6.227384	-2.868639	2.598112
H	-4.664816	-3.217201	-3.702837	C	-4.453628	-3.332765	4.730755
C	-5.276899	-6.003391	-1.638689	H	-2.972682	-3.830217	3.236465
H	-5.929393	-6.522136	-0.907580	C	-6.641420	-2.639625	3.911808
H	-5.503477	-6.406225	-2.647678	H	-6.903150	-2.671483	1.754192
H	-4.217614	-6.246014	-1.424713	C	-5.759154	-2.881759	4.982111
C	-7.372431	-3.715086	-1.963533	H	-3.754803	-3.499238	5.563514
H	-7.624405	-3.898642	-3.028076	H	-7.658103	-2.265969	4.107484
H	-8.059864	-4.315906	-1.334986	H	-6.087833	-2.701174	6.017199

11. References

1. Xu, Y. *et al.* Deacylative transformations of ketones via aromatization-promoted C–C bond activation. *Nature* **567**, 373–378 (2019).

2. Penafiel, I., Pastor, I. M., Yus, M., Esteruelas, M. A. & Olivan, M. Preparation, hydrogen bonds, and catalytic activity in metal-promoted addition of arylboronic acids to enones of a rhodium complex containing an NHC ligand with an alcohol function. *Organometallics* **31**, 6154–6161 (2012).

3. Kaib, P. S., Schreyer, L., Lee, S., Properzi, R. & List, B. Extremely Active Organocatalysts Enable a Highly Enantioselective Addition of Allyltrimethylsilane to Aldehydes. *Angew. Chem. Int. Ed.* **55**, 13200–13203 (2016).

4. Gatzenmeier, T. *et al.* Scalable and Highly Diastereo-and Enantioselective Catalytic Diels–Alder Reaction of α, β-Unsaturated Methyl Esters. *J. Am. Chem. Soc*. **140**, 12671–12676 (2018).

5. Fulmer, G. R. *et al.* NMR chemical shifts of trace impurities: common laboratory solvents, organics, and gases in deuterated solvents relevant to the organometallic chemist. *Organometallics* **29**, 2176–2179 (2010).

6. Hoffman, R. E. Standardization of chemical shifts of TMS and solvent signals in NMR solvents. *Magn. Reson. Chem.* **44**, 606–616 (2006).

7. Chen, F. X. *et al.* Enantioselective cyanosilylation of ketones by a catalytic double-activation method with an aluminium complex and an N-oxide. *Chem. Eur. J.* **10**, 4790–4797 (2004).

8. Zeng, X.-P. *et al.* Activation of Chiral (salen) AlCl Complex by Phosphorane for Highly Enantioselective Cyanosilylation of Ketones and Enones. *J. Am. Chem. Soc*. **138**, 416–425 (2015).

9. Xiong, Y. *et al.* Enantioselective cyanosilylation of ketones catalyzed by a nitrogen-containing bifunctional catalyst. *Adv. Synth. Catal.* **348**, 538–544 (2006).

10. Hamashima, Y., Kanai, M. & Shibasaki, M. Catalytic Enantioselective Cyanosilylation of Ketones. *J. Am. Chem. Soc*. **122**, 7412–7413 (2000).

11. Hatano, M., Yamakawa, K., Kawai, T., Horibe, T., & Ishihara, K. Enantioselective Cyanosilylation of Ketones with Lithium (I) Dicyanotrimethylsilicate (IV) Catalyzed by a Chiral Lithium (I) Phosphoryl Phenoxide. *Angew. Chem. Int. Ed.* **55**, 4021–4025 (2016).

12. Fuerst, D. E. & Jacobsen, E. N. Thiourea-Catalyzed Enantioselective Cyanosilylation of Ketones. *J. Am. Chem. Soc*. **127**, 8964–8965 (2005).

13. Kanda, T., Naraoka, A. & Naka, H. Catalytic Transfer Hydration of Cyanohydrins to α-Hydroxyamides. *J. Am. Chem. Soc*. **141**, 825–830 (2018).

14. Hayashi, M., Yoshiga, T., Nakatani, K., Ono, K. & Oguni, N. Reduction of α-trialkylsiloxy nitriles with diisobutylaluminium hydride (DIBAH): a facile preparation of α-trialkylsiloxy aldehydes and their derivatives. *Tetrahedron* **50**, 2821–2830 (1994).

15. Ryu, D. H. & Corey, E. Enantioselective Cyanosilylation of Ketones Catalyzed by a Chiral Oxazaborolidinium Ion. *J. Am. Chem. Soc*. **127**, 5384–5387 (2005).

16. Tan, L. *et al.* Practical enantioselective synthesis of a COX-2 specific inhibitor. *Tetrahedron* **58**, 7403–7410 (2002).

17. Nielsen, C. D.-T. & Burés, J. Visual kinetic analysis. *Chem. Sci.* **10**, 348–353 (2019).

18. Burés, J. A Simple Graphical Method to Determine the Order in Catalyst. *Angew. Chem. Int. Ed.* **55**, 2028–2031 (2016).

19. Burés, J. What is the Order of a Reaction? *Topics in Catalysis* **60**, 631–633 (2017).

20. Burés, J., Armstrong, A. & Blackmond, D. G. Kinetic correlation between aldehyde/enamine stereoisomers in reactions between aldehydes with α-stereocenters and chiral pyrrolidine-based catalysts. *Chem. Sci.* **3**, 1273–1277 (2012).

21. Burés, J. Variable Time Normalization Analysis: General Graphical Elucidation of Reaction Orders from Concentration Profiles. *Angew. Chem. Int. Ed.* **55**, 16084–16087 (2016).

22. Zhang, Z., Klussmann, M. & List, B. Kinetic Study of Disulfonimide Catalyzed Cyanosilylation of Aldehyde Using a Method of Progress Rates. *Synlett* **31**, 1593–1597 (2020).

23. Lee, S., Kaib, P. S. & List, B. N-Triflylphosphorimidoyl Trichloride: A Versatile Reagent for the Synthesis of Strong Chiral Brønsted Acids. *Synlett* **28**, 1478–1480 (2017).

24. Liu, L. *et al.* Catalytic Asymmetric [4+2]-Cycloaddition of Dienes with Aldehydes. *J. Am. Chem. Soc.* **139**, 13656–13659 (2017).

25. Jolit, A. *et al.* Catalytic enantioselective nazarov cyclization. *Eur. J. Org. Chem.* **2017**, 6067–6076 (2017).

26. Yepes, D., Neese, F., List, B. & Bistoni, G. Unveiling the Delicate Balance of Steric and Dispersion Interactions in Organocatalysis Using High-level Computational Methods. *J. Am. Chem. Soc.* **142**, 3613–3625 (2020).

27. Frisch, M. *et al.* Gaussian 09 (Gaussian, Inc., Wallingford, CT, 2009).

28. Xtb, Version 6.3; University Bonn: 2020; please refer to xtb@thch.uni-bonn.de.

29. Bannwarth, C., Ehlert, S. & Grimme, S. GFN2-xTB—An accurate and broadly parametrized self-consistent tight-binding quantum chemical method with multipole electrostatics and density-dependent dispersion contributions. *J. Chem. Theory Comput.* **15**, 1652–1671 (2019).

30. T. Lu, *molclus program, http://www.keinsci.com/research/molclus.html*.

31. Perdew, J. P., Burke, K. & Ernzerhof, M. Fluid vesicles in shear flow. *Phys. Rev. Lett.* **77**, 3865–3868 (1996).

32. Grimme, S. Semiempirical GGA-type density functional constructed with a long-range dispersion correction. *J. Comput. Chem.* **27**, 1787–1799 (2006).

33. Weigend, F. & Ahlrichs, R. Balanced basis sets of split valence, triple zeta valence and quadruple zeta valence quality for H to Rn: Design and assessment of accuracy. *Phys. Chem. Chem. Phys.* **7**, 3297–3305 (2005).

34. Tomasi, J., Mennucci, B. & Cammi, R. Quantum mechanical continuum solvation models. *Chem. Rev.* **105**, 2999–3094 (2005).

35. Falivene, L. *et al.* SambVca 2. A web tool for analyzing catalytic pockets with topographic steric maps. *Organometallics* **35**, 2286–2293 (2016).

36. Humphrey, W., Dalke, A. & Schulten, K. VMD: visual molecular dynamics. *J. Mol. Graph.* **14**, 33–38 (1996).

37. Aggarwal, V. K. & Daly, A. M. Extension of ring closing metathesis methodology to the synthesis of carbocyclic methyl and silyl enol ethers. *Chem. Commun*, 2490–2491 (2002).

12. Copies of NMR spectra

(*S*)-2-methyl-2-((trimethylsilyl)oxy)butanenitrile 6

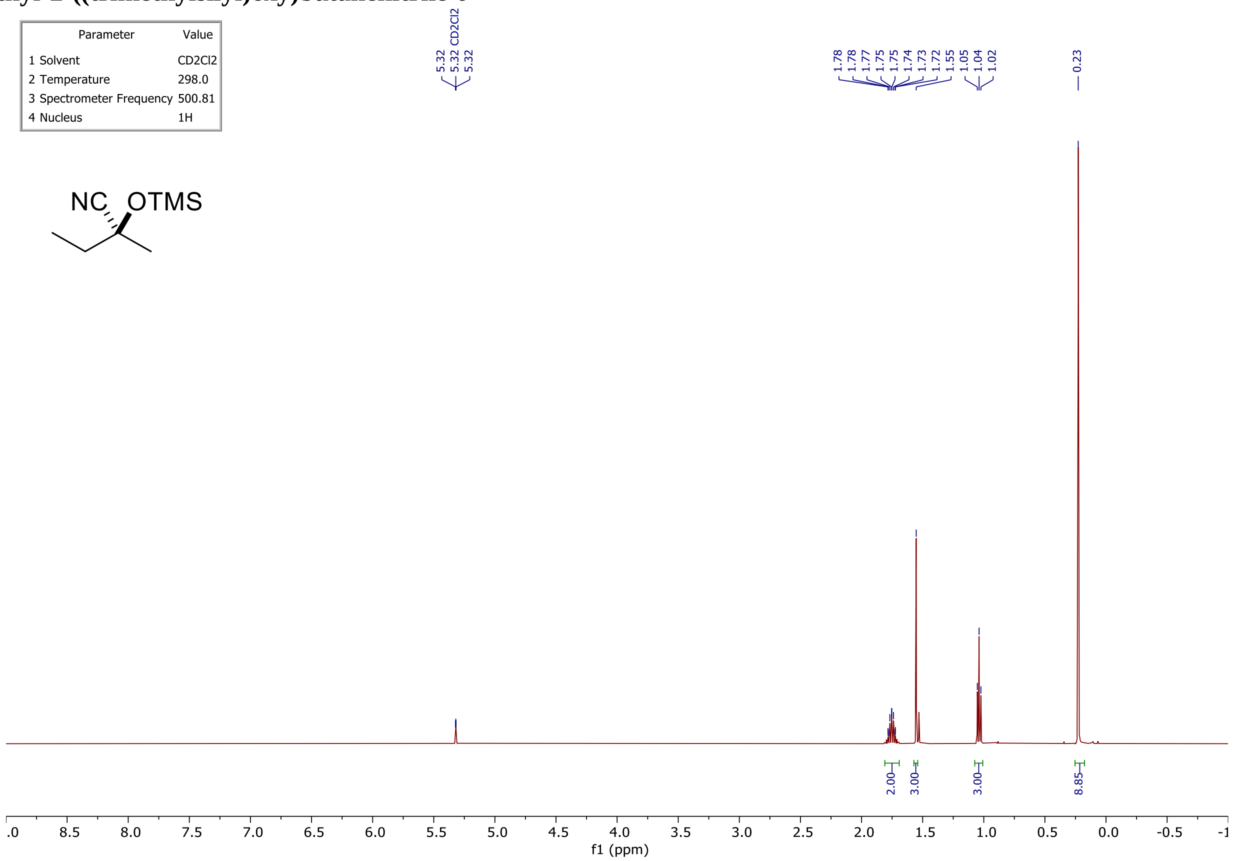

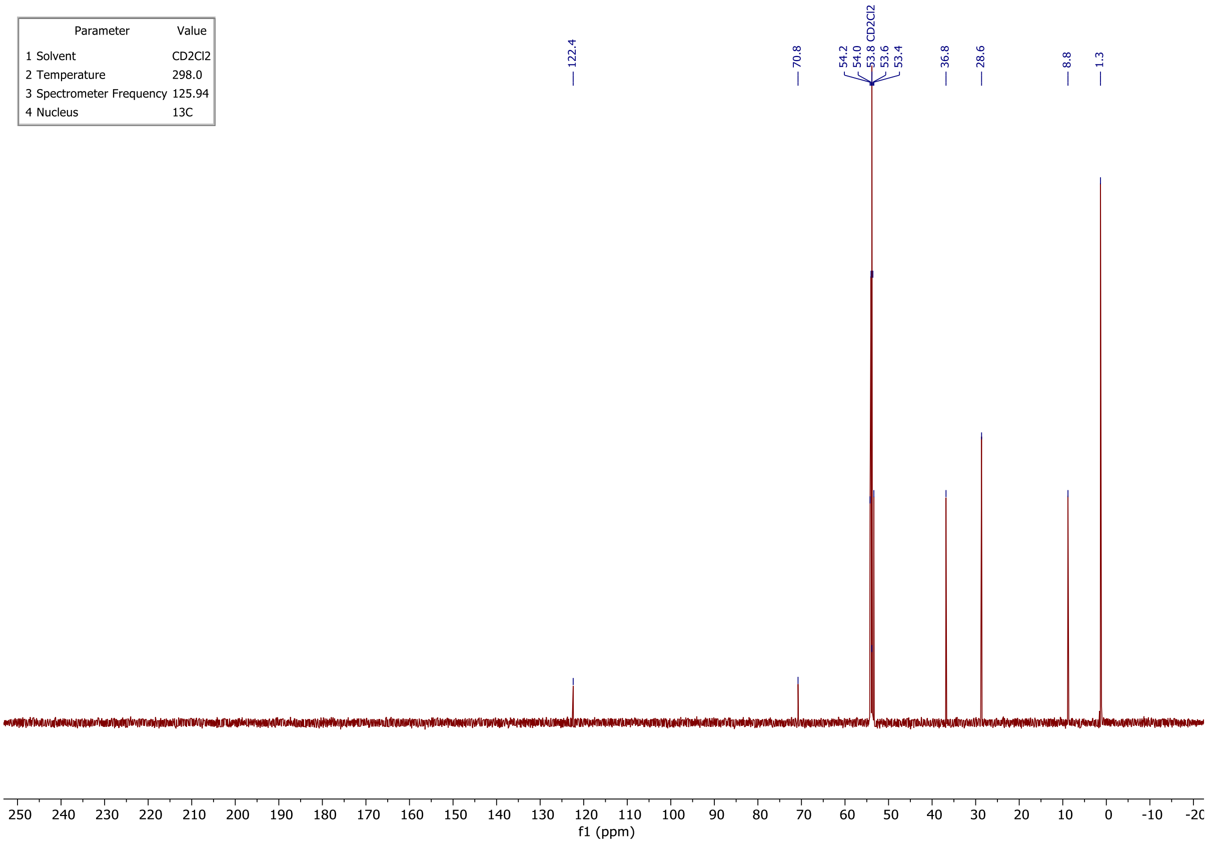

2-methyl-2-((trimethylsilyl)oxy)pentanenitrile 7

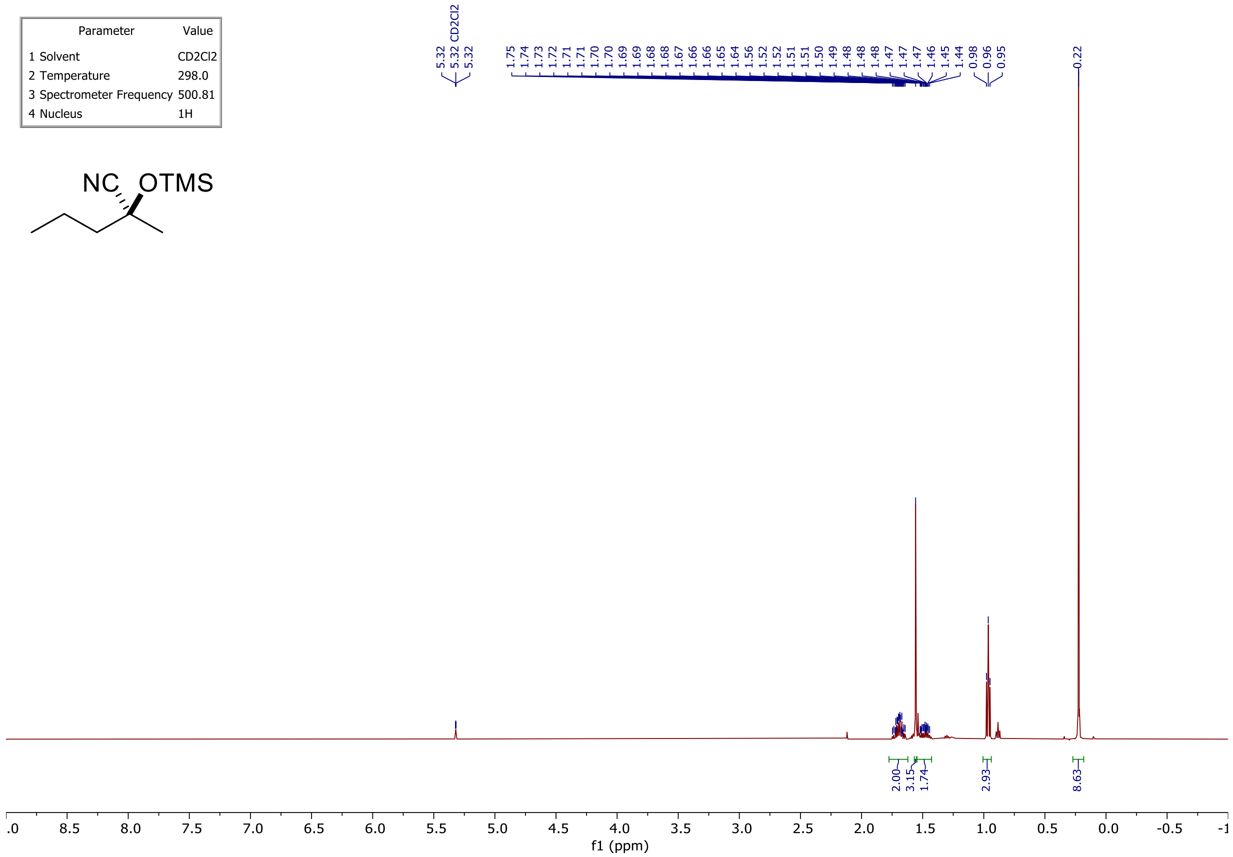

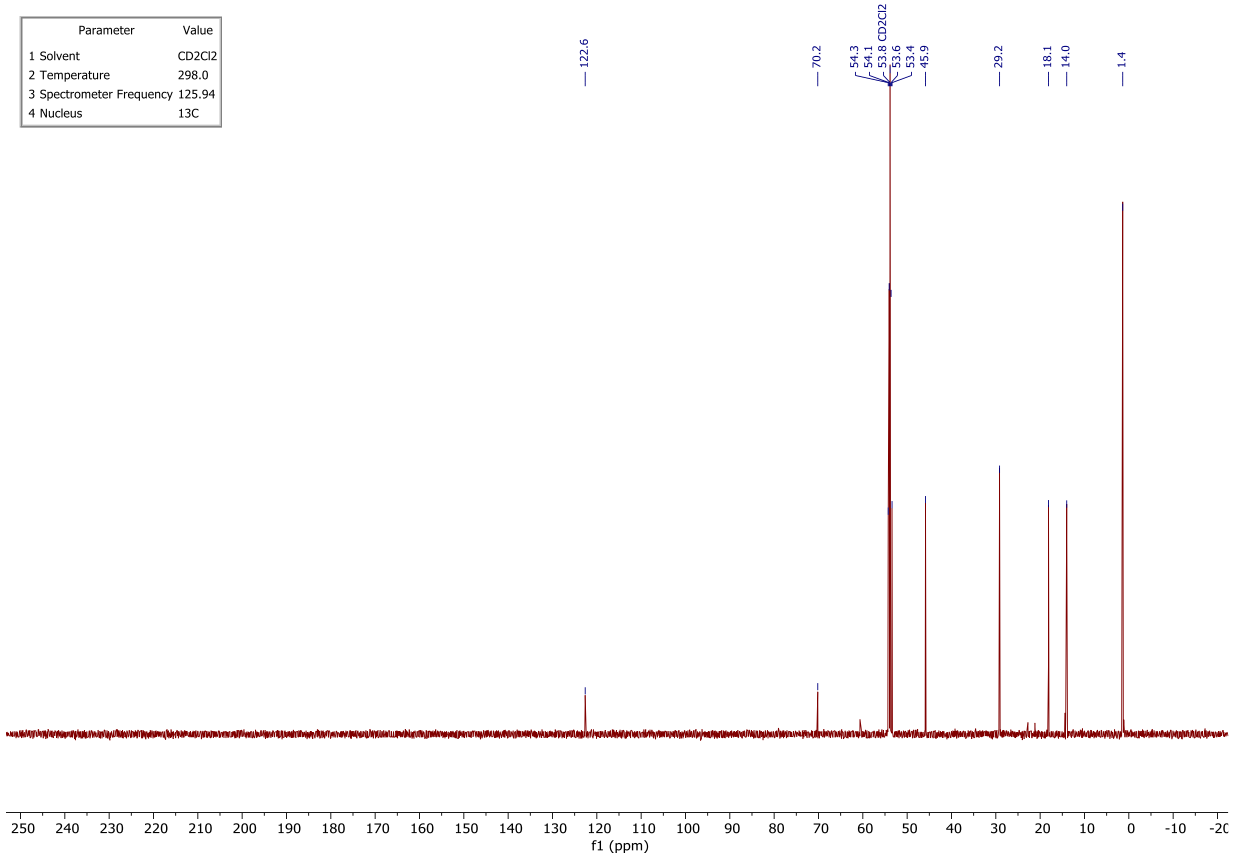

2-methyl-2-((trimethylsilyl)oxy)hexanenitrile 8

Parameter	Value
1 Solvent	CD2Cl2
2 Temperature	297.9
3 Spectrometer Frequency	500.81
4 Nucleus	1H

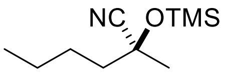

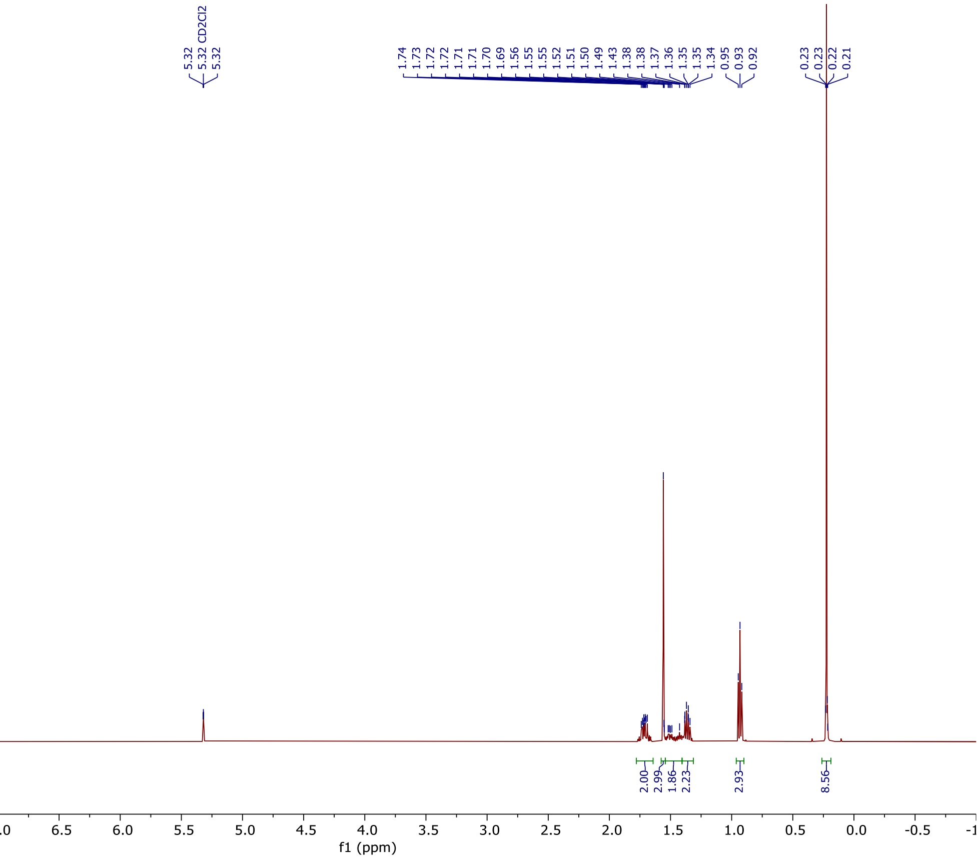

Parameter	Value
1 Solvent	CD2Cl2
2 Temperature	298.0
3 Spectrometer Frequency	125.94
4 Nucleus	13C

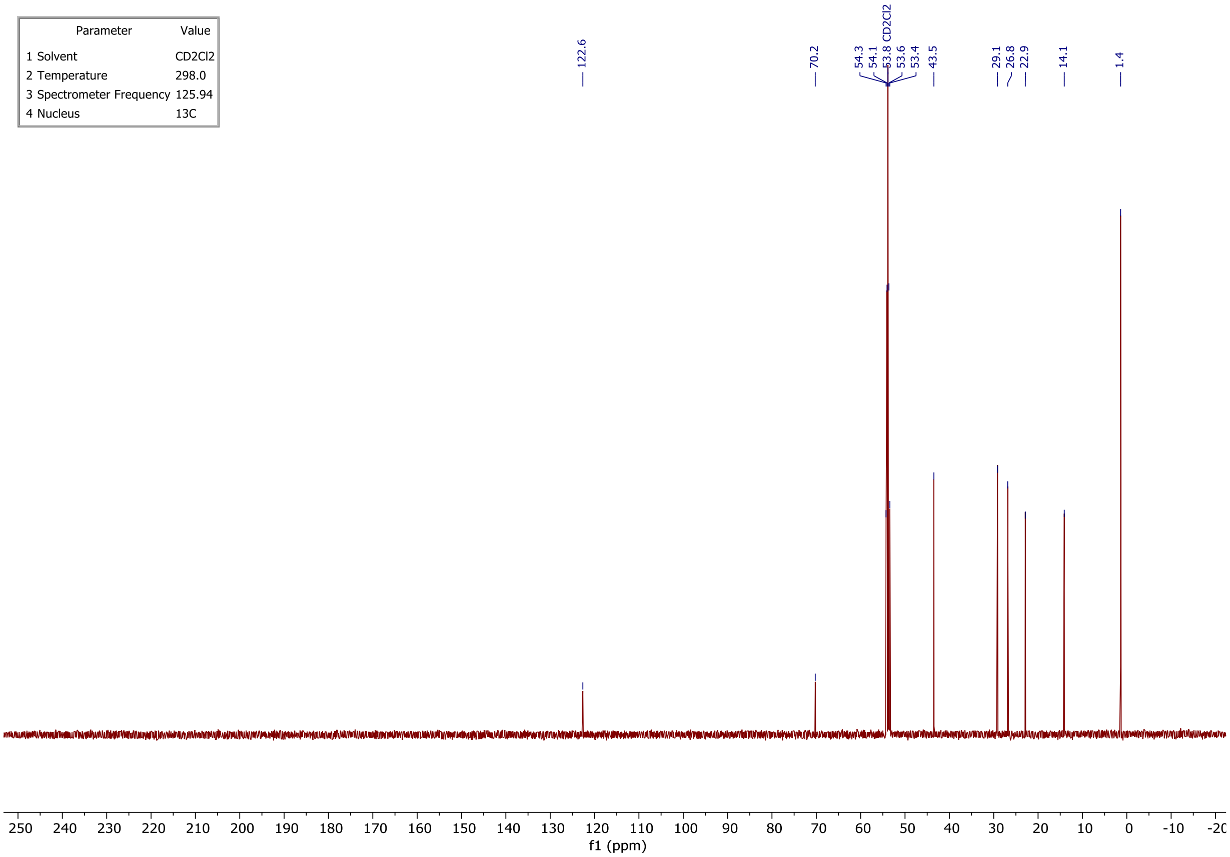

2-methyl-2-((trimethylsilyl)oxy)heptanenitrile 9

Parameter	Value
1 Solvent	CD2Cl2
2 Temperature	297.9
3 Spectrometer Frequency	500.81
4 Nucleus	1H

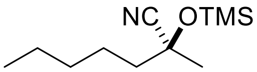

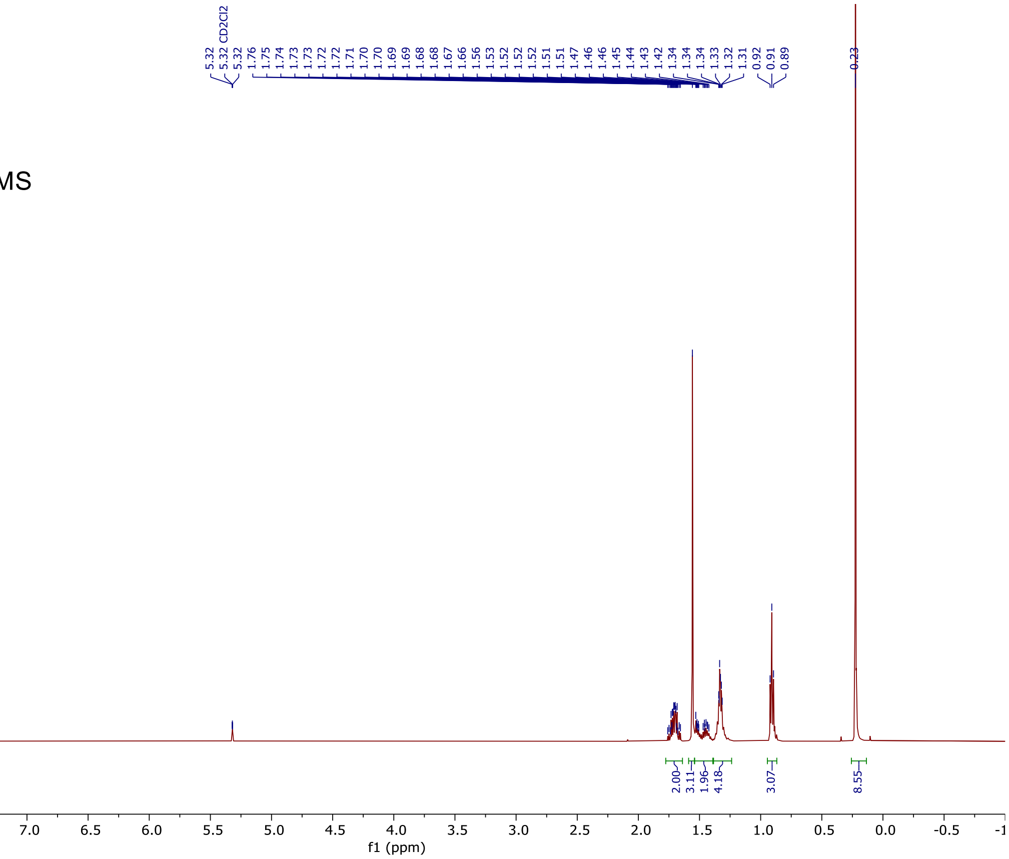

Parameter	Value
1 Solvent	CD2Cl2
2 Temperature	298.0
3 Spectrometer Frequency	125.94
4 Nucleus	13C

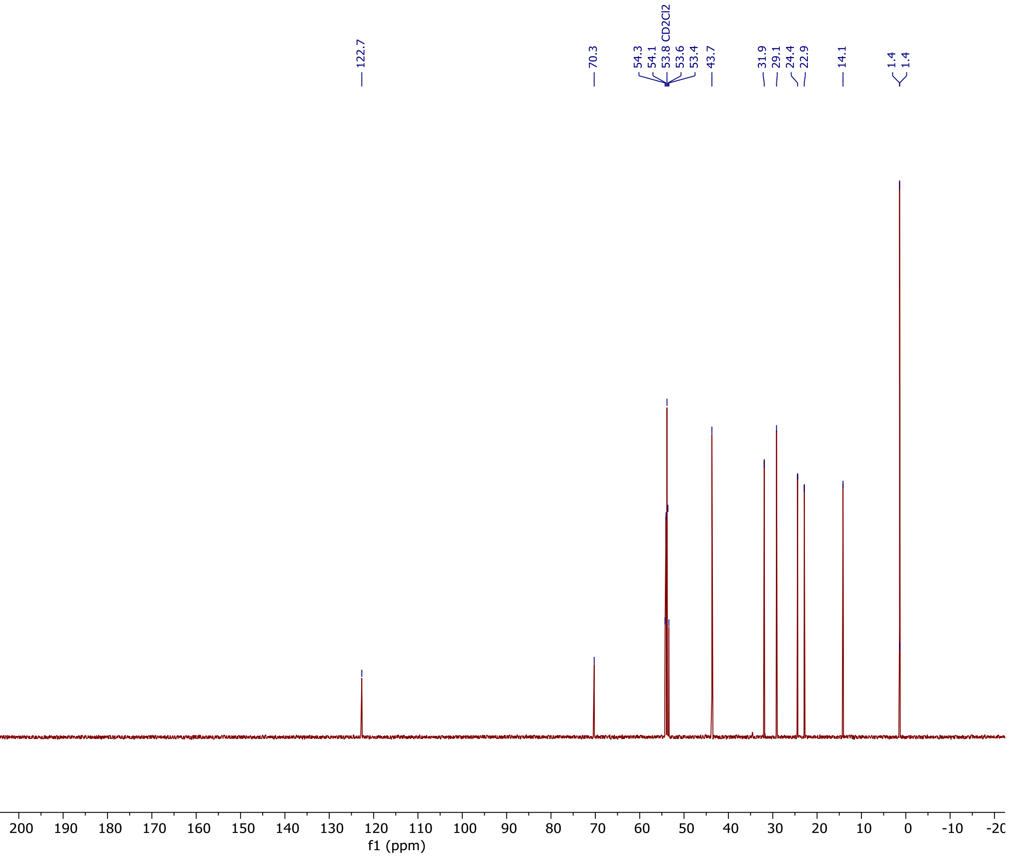

2,4-dimethyl-2-((trimethylsilyl)oxy)pentanenitrile 10

Parameter	Value
1 Solvent	CD2Cl2
2 Temperature	297.9
3 Spectrometer Frequency	500.81
4 Nucleus	1H

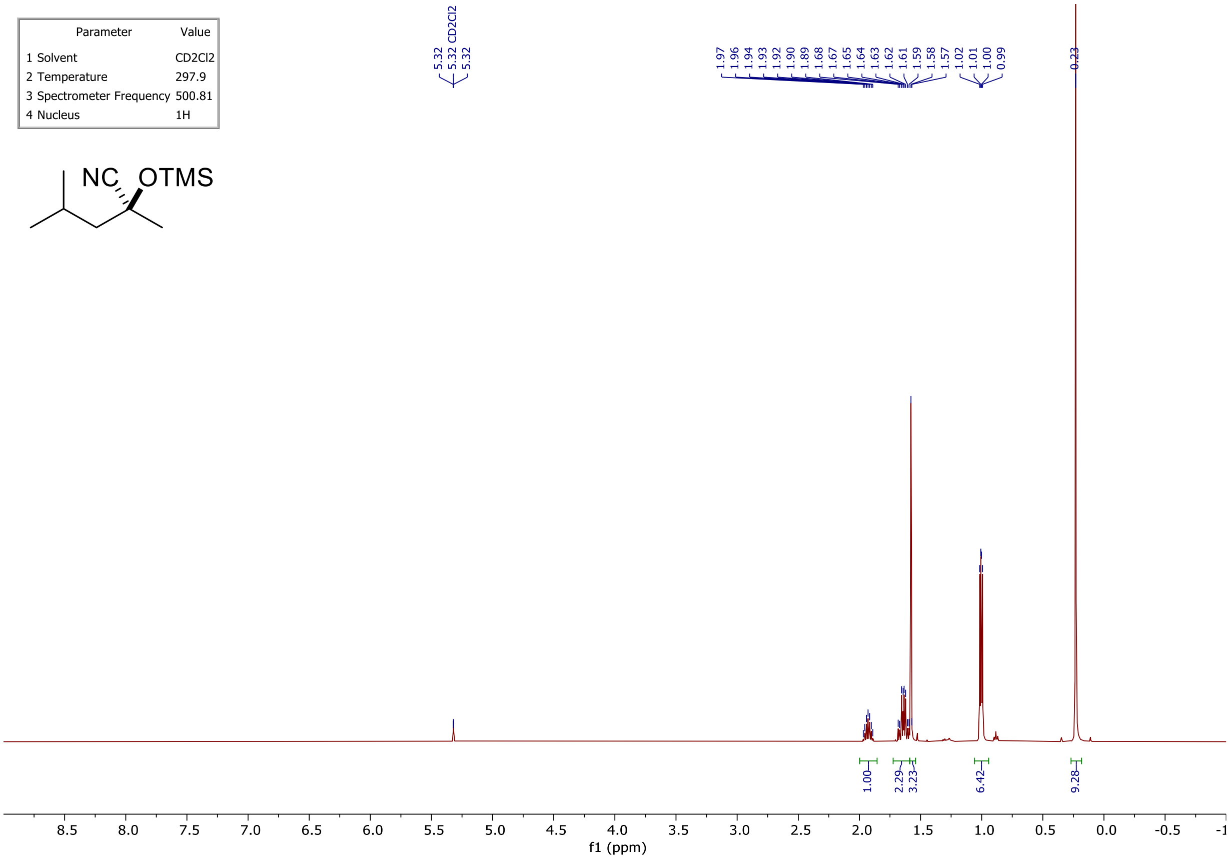

Parameter	Value
1 Solvent	CD2Cl2
2 Temperature	298.0
3 Spectrometer Frequency	125.94
4 Nucleus	13C

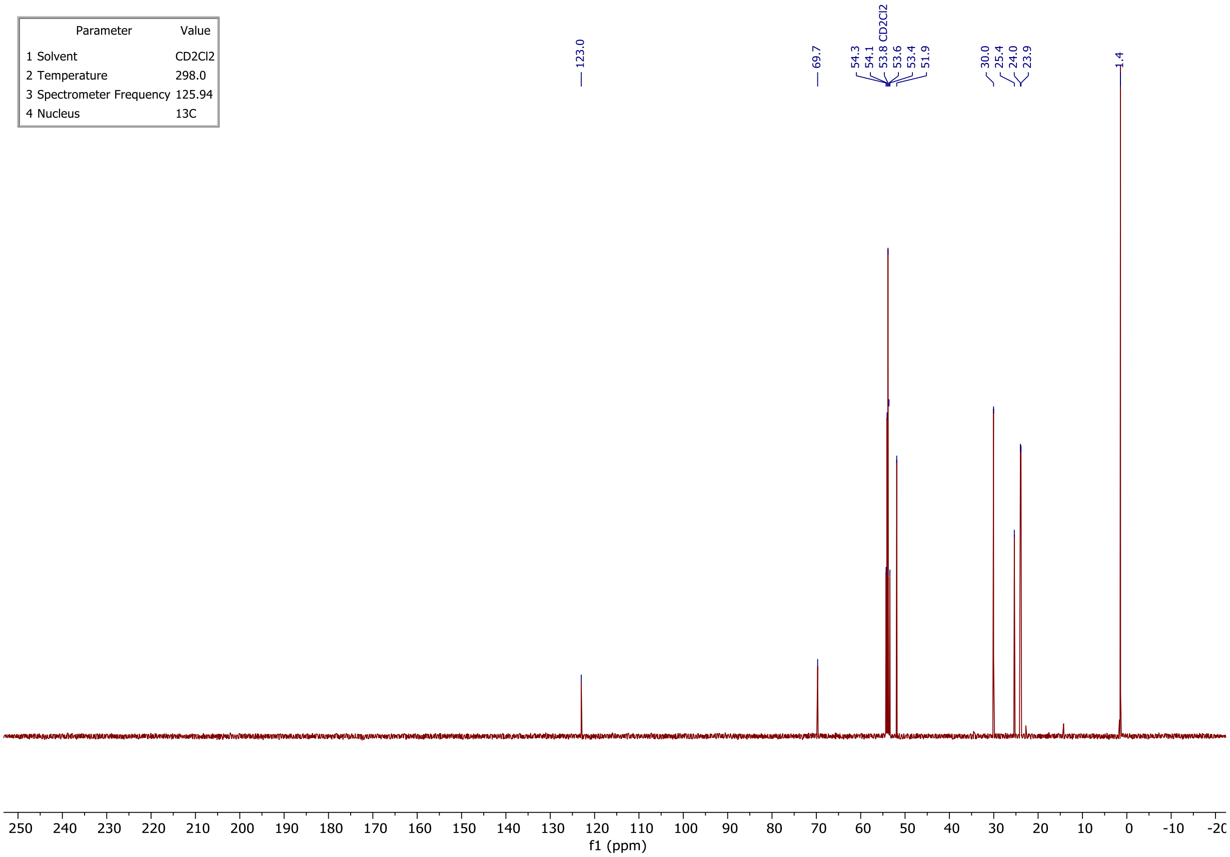

2,5-dimethyl-2-((trimethylsilyl)oxy)hexanenitrile 11

	Parameter	Value
1	Solvent	CD2Cl2
2	Temperature	297.9
3	Spectrometer Frequency	500.81
4	Nucleus	1H

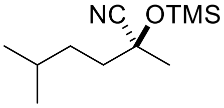

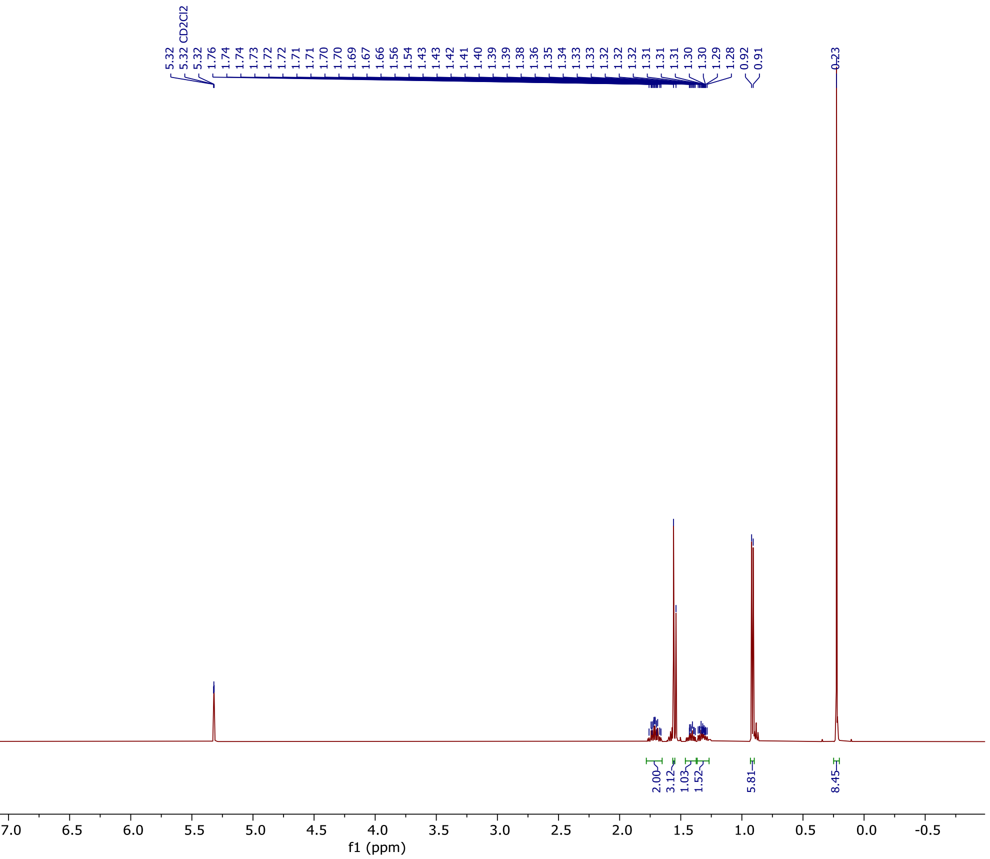

	Parameter	Value
1	Solvent	CD2Cl2
2	Temperature	298.0
3	Spectrometer Frequency	125.94
4	Nucleus	13C

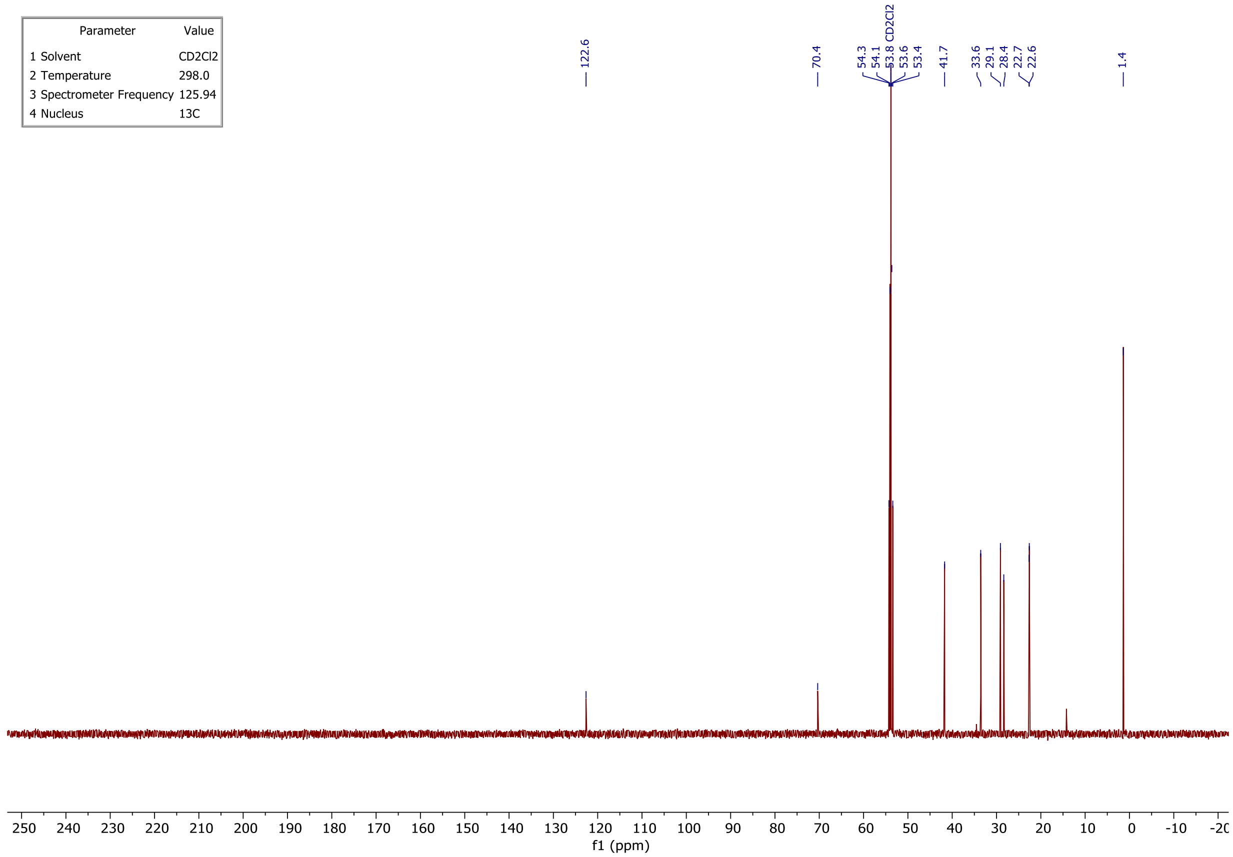

3-cyclohexyl-2-methyl-2-((trimethylsilyl)oxy)propanenitrile 12

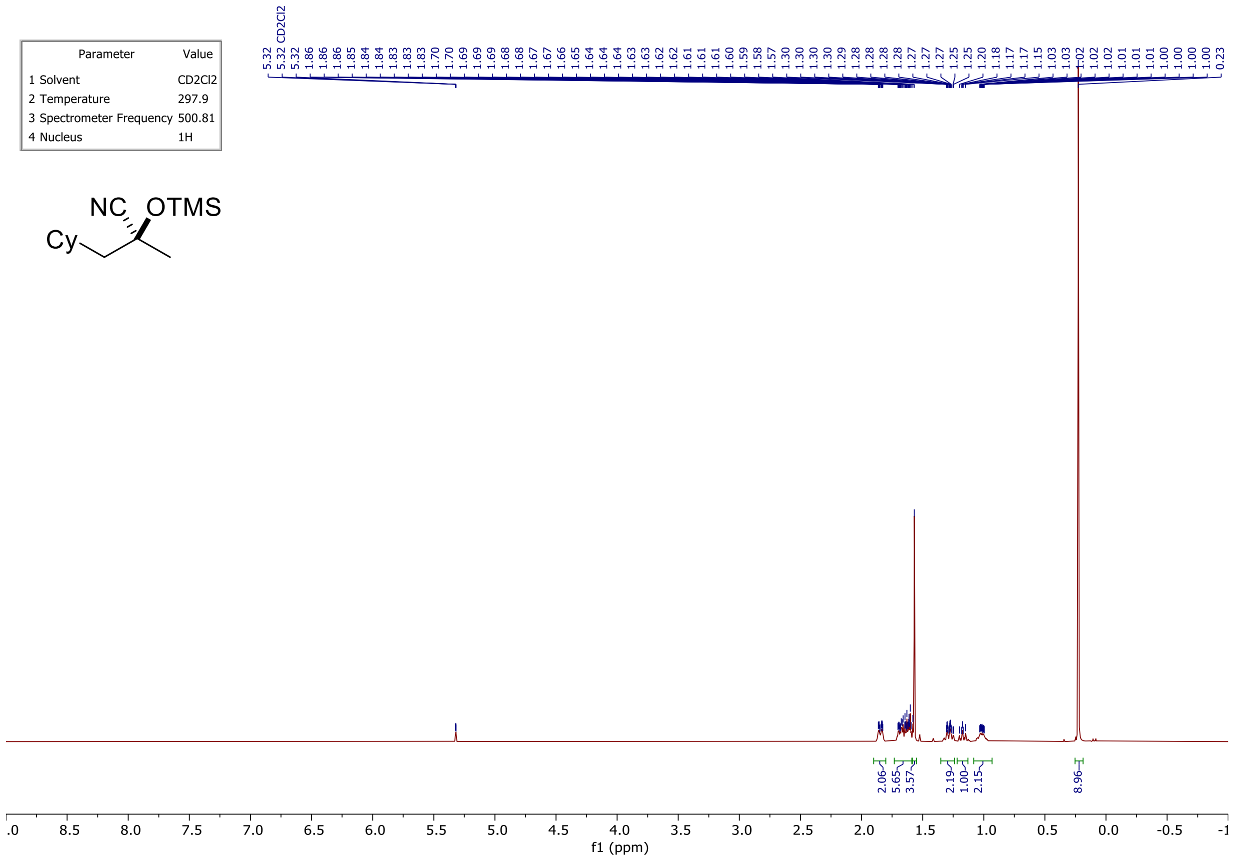

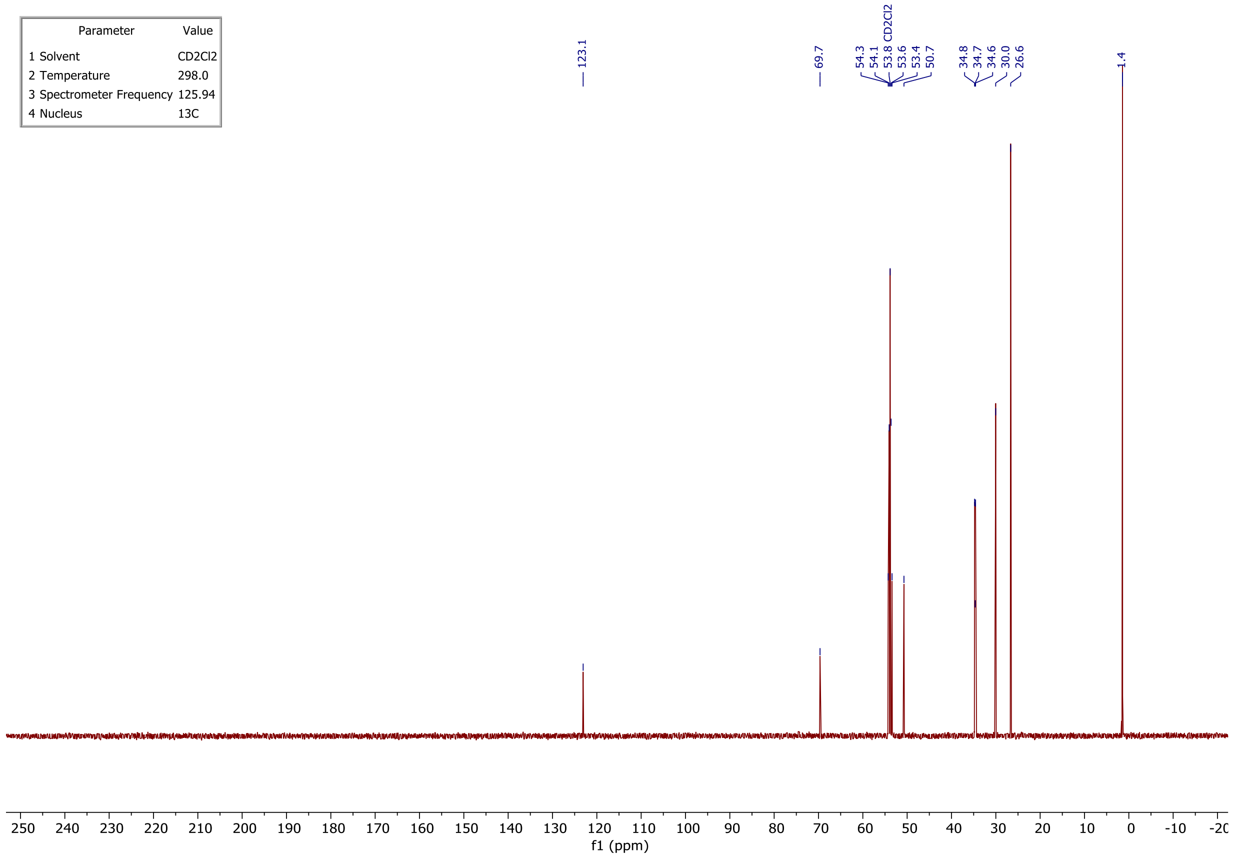

5-chloro-2-methyl-2-((trimethylsilyl)oxy)pentanenitrile 13

Parameter	Value
1 Solvent	CD2Cl2
2 Temperature	297.8
3 Spectrometer Frequency	500.81
4 Nucleus	1H

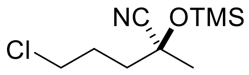

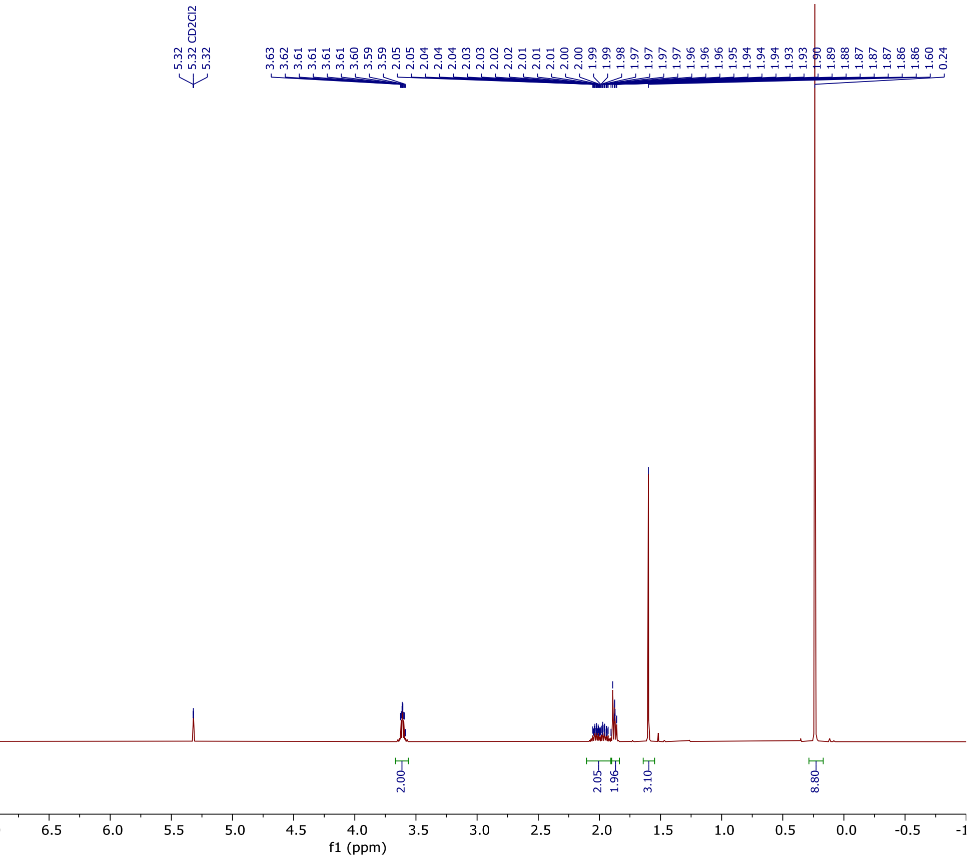

Parameter	Value
1 Solvent	CD2Cl2
2 Temperature	298.0
3 Spectrometer Frequency	125.94
4 Nucleus	13C

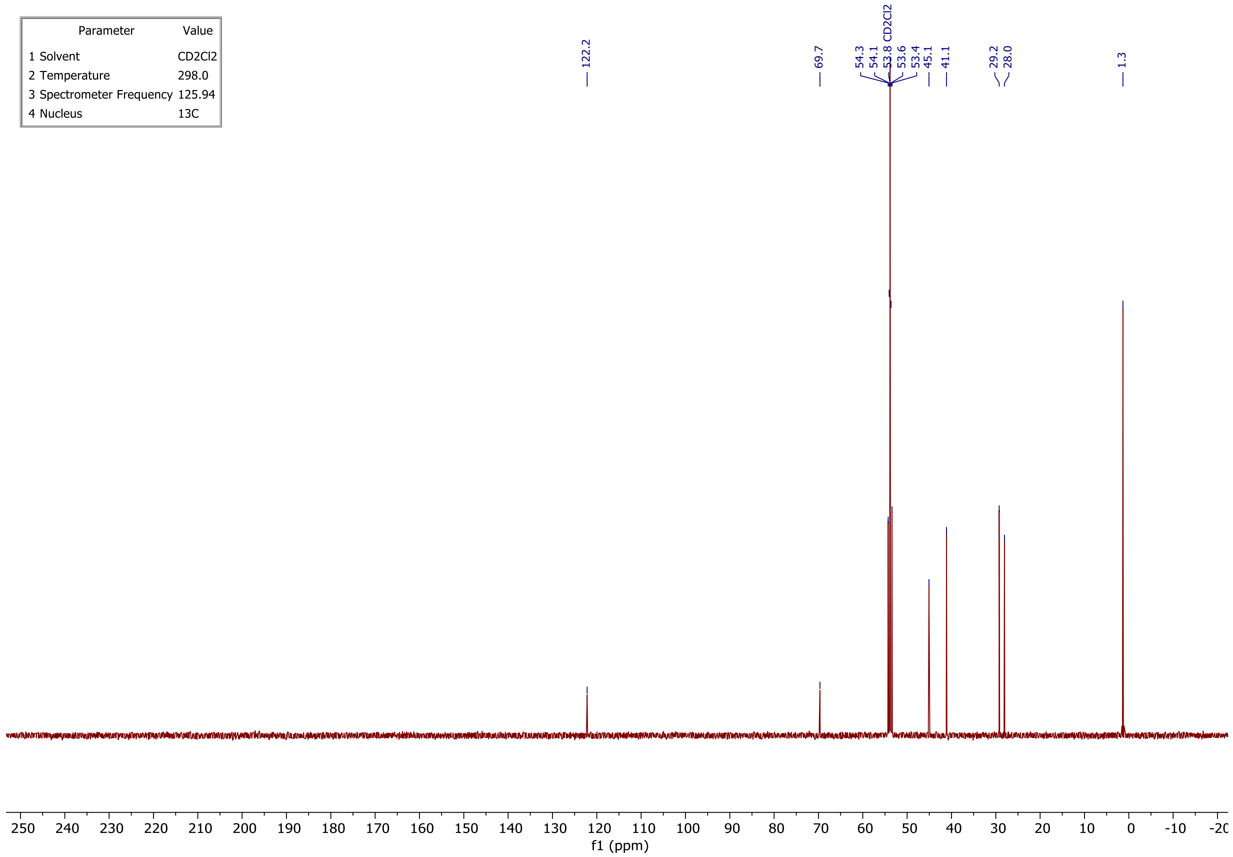

6-chloro-2-methyl-2-((trimethylsilyl)oxy)hexanenitrile 14

Parameter	Value
1 Solvent	CD2Cl2
2 Temperature	297.9
3 Spectrometer Frequency	500.81
4 Nucleus	1H

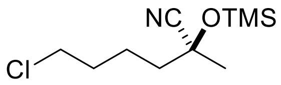

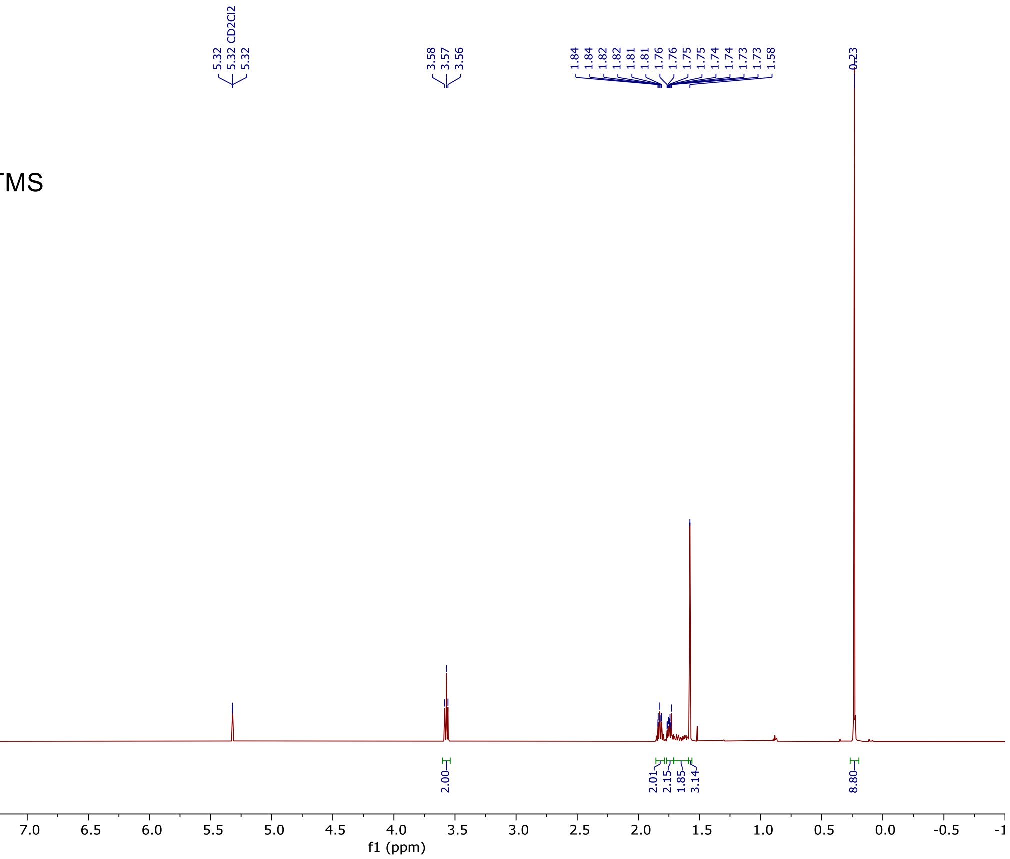

Parameter	Value
1 Solvent	CD2Cl2
2 Temperature	298.0
3 Spectrometer Frequency	125.94
4 Nucleus	13C

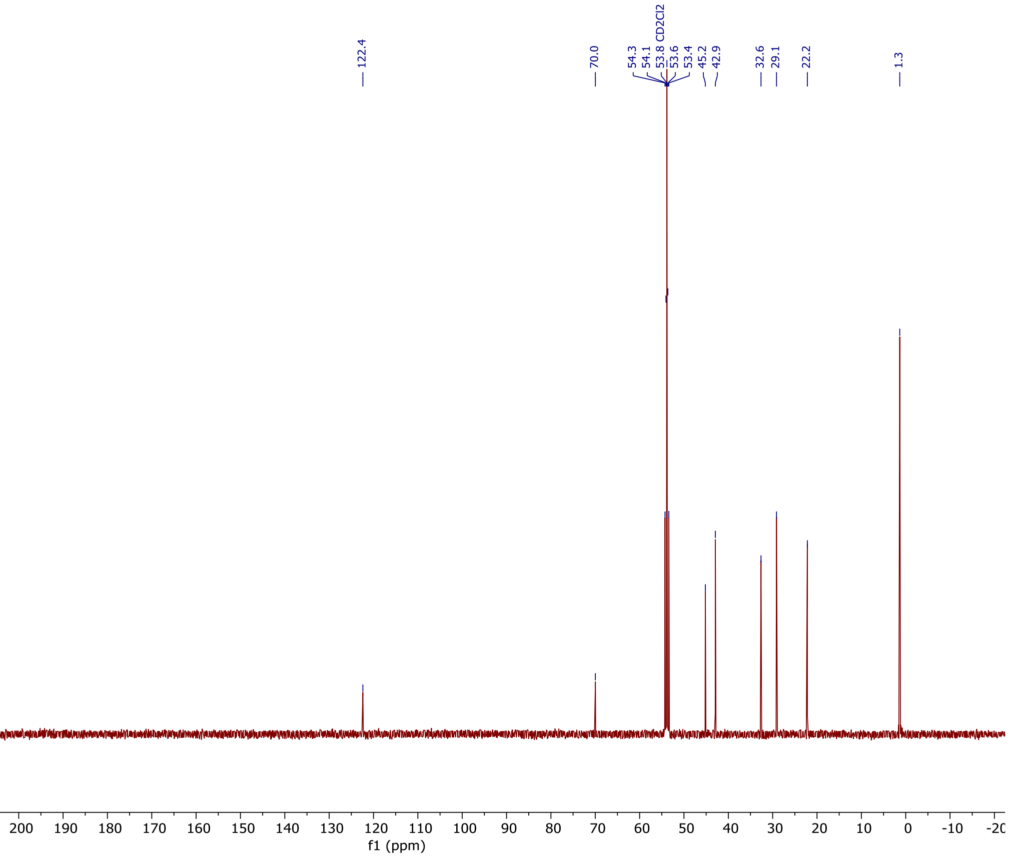

2,6-dimethyl-2-((trimethylsilyl)oxy)hept-5-enenitrile 15

Parameter	Value
1 Solvent	CD2Cl2
2 Temperature	297.9
3 Spectrometer Frequency	500.81
4 Nucleus	1H

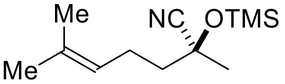

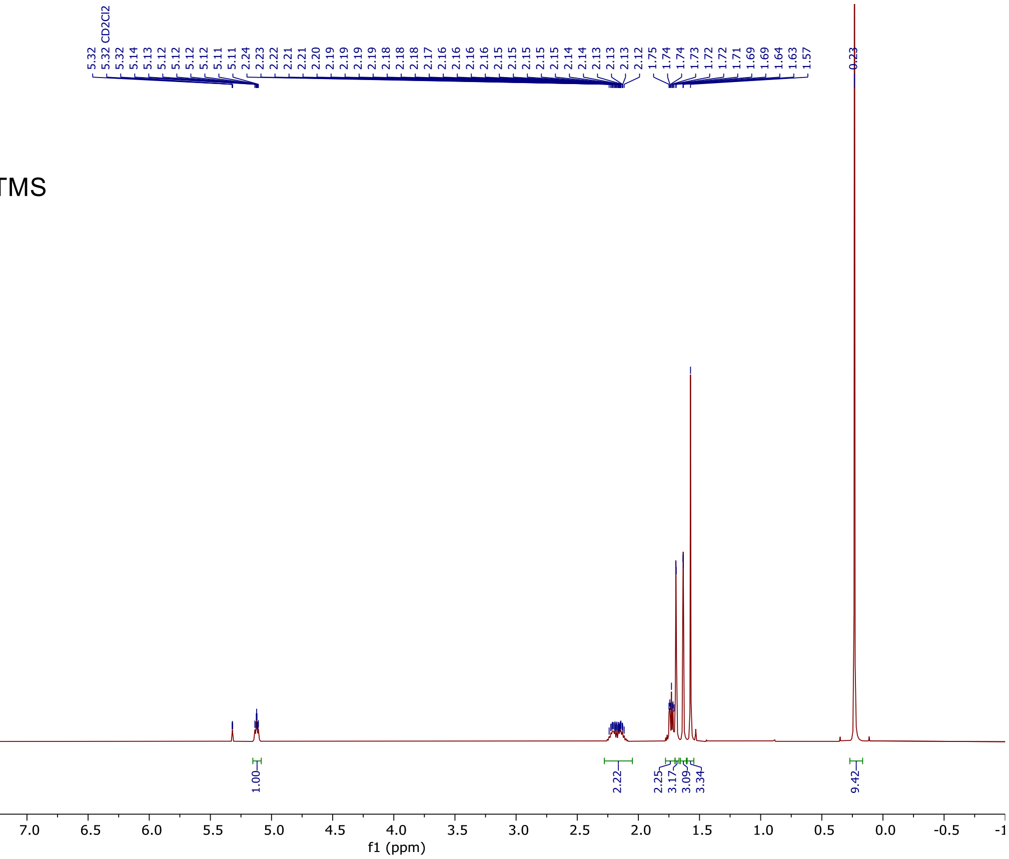

Parameter	Value
1 Solvent	CD2Cl2
2 Temperature	298.0
3 Spectrometer Frequency	125.94
4 Nucleus	13C

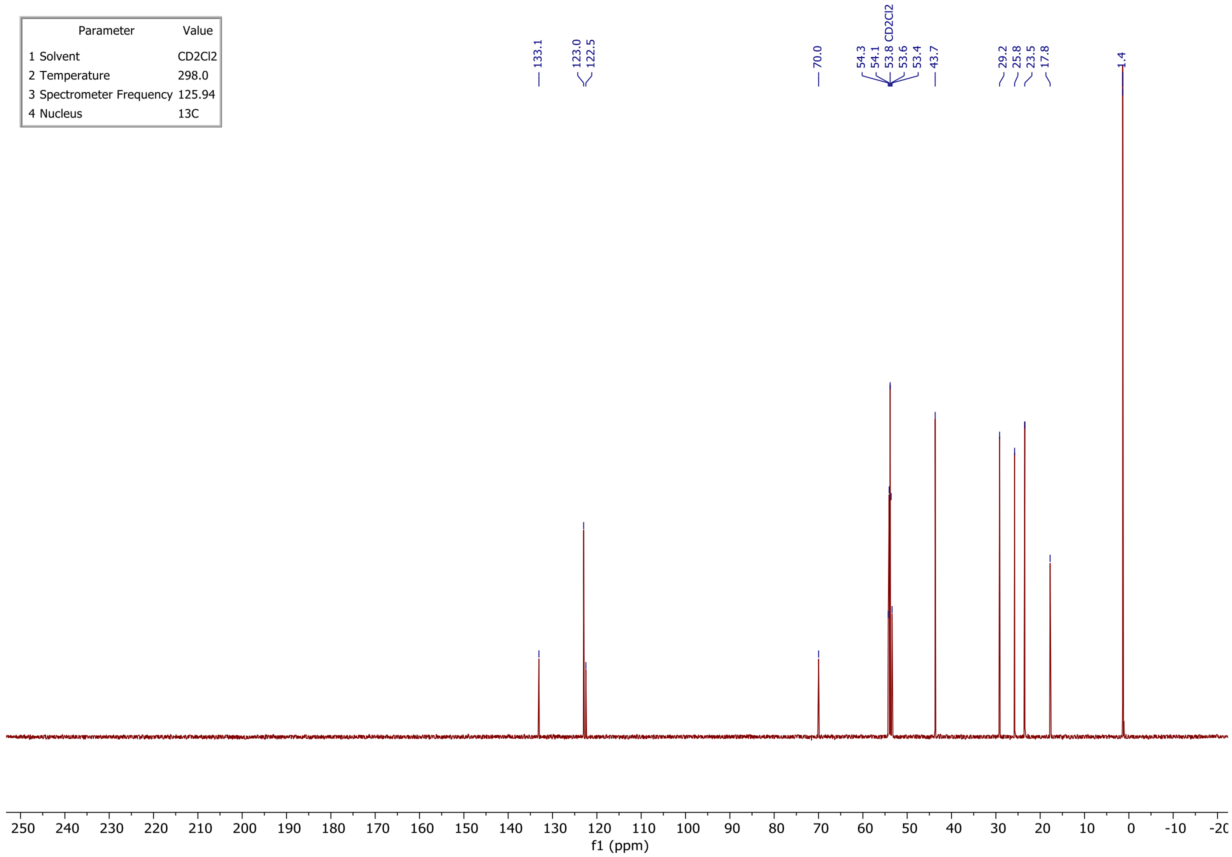

4-((*tert*-butyldimethylsilyl)oxy)-2-methyl-2-((trimethylsilyl)oxy)butanenitrile 16

Parameter	Value
1 Solvent	CD2Cl2
2 Temperature	297.9
3 Spectrometer Frequency	500.81
4 Nucleus	1H

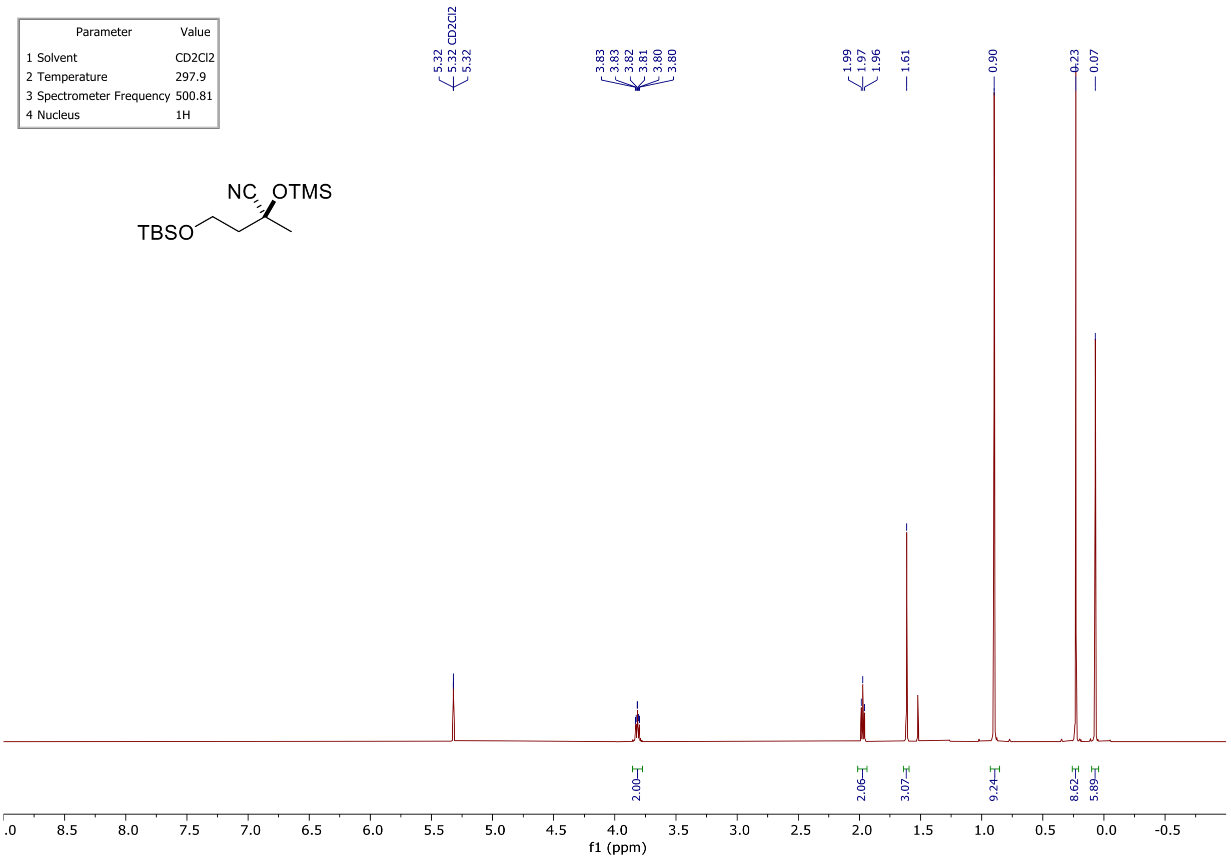

Parameter	Value
1 Solvent	CD2Cl2
2 Temperature	298.0
3 Spectrometer Frequency	125.94
4 Nucleus	13C

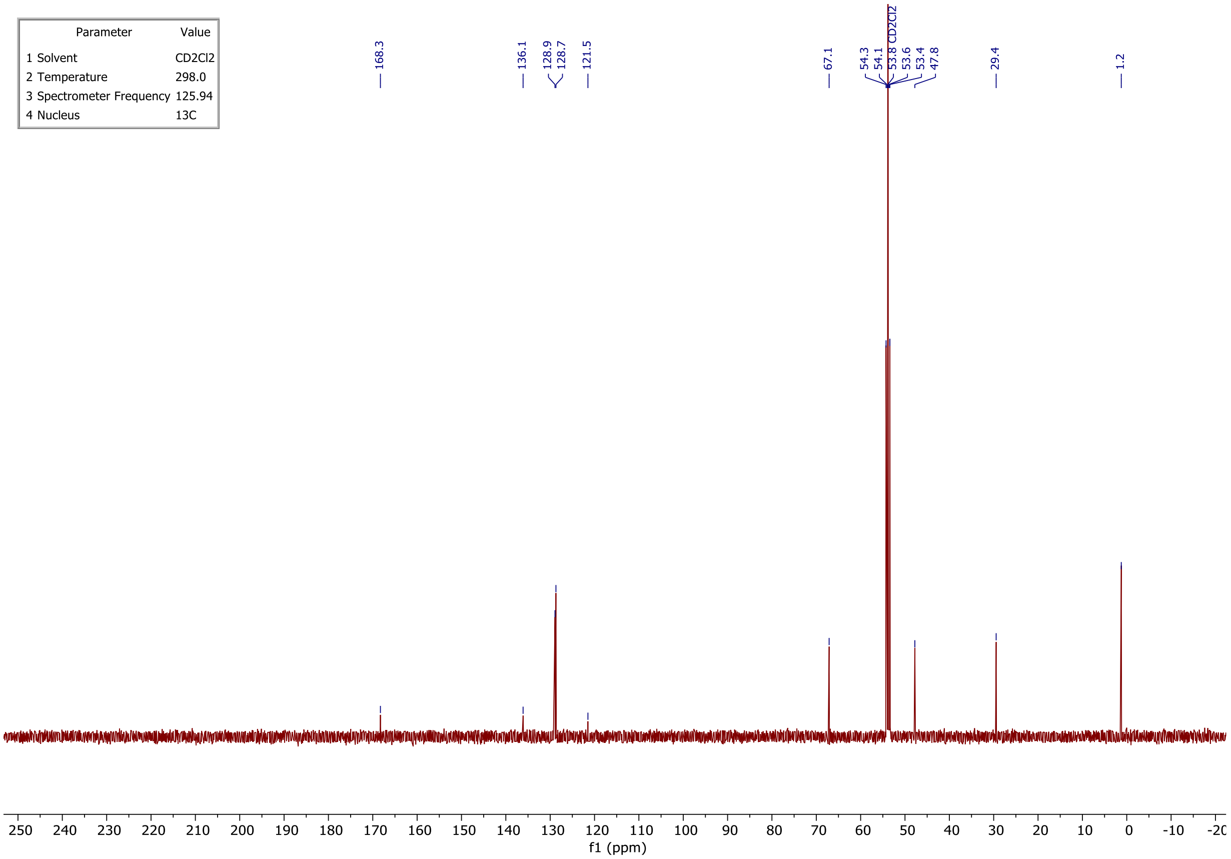

3,3-dimethyl-1-((trimethylsilyl)oxy)cyclohexane-1-carbonitrile 17

Parameter	Value
1 Solvent	CD2Cl2
2 Temperature	298.0
3 Spectrometer Frequency	500.81
4 Nucleus	1H

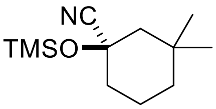

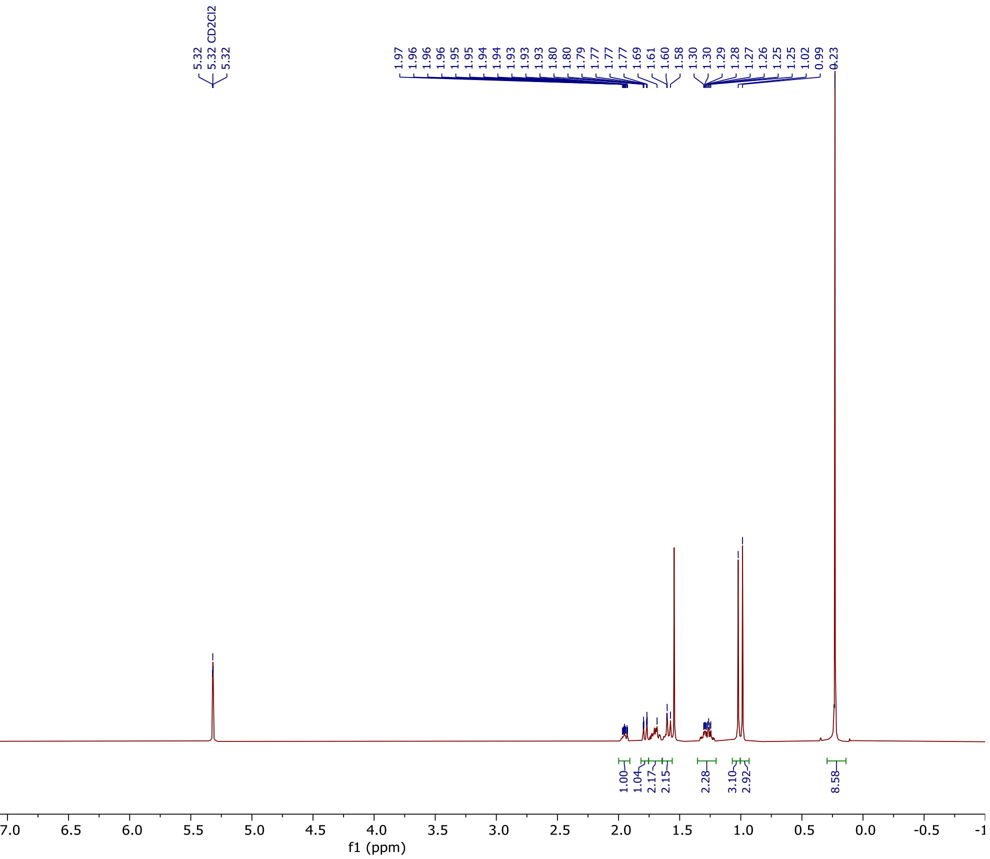

Parameter	Value
1 Solvent	CD2Cl2
2 Temperature	298.0
3 Spectrometer Frequency	125.94
4 Nucleus	13C

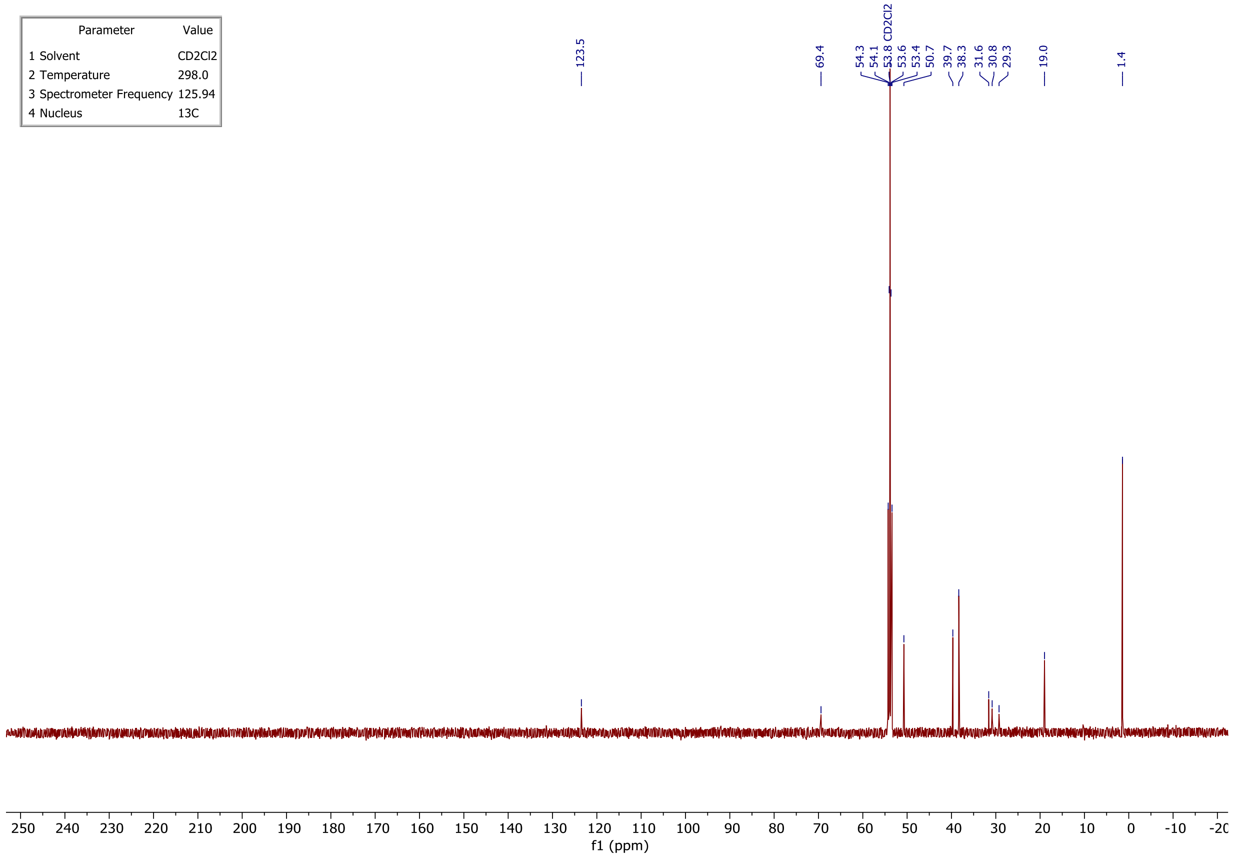

3-oxocyclohexane-1-carbonitrile 18

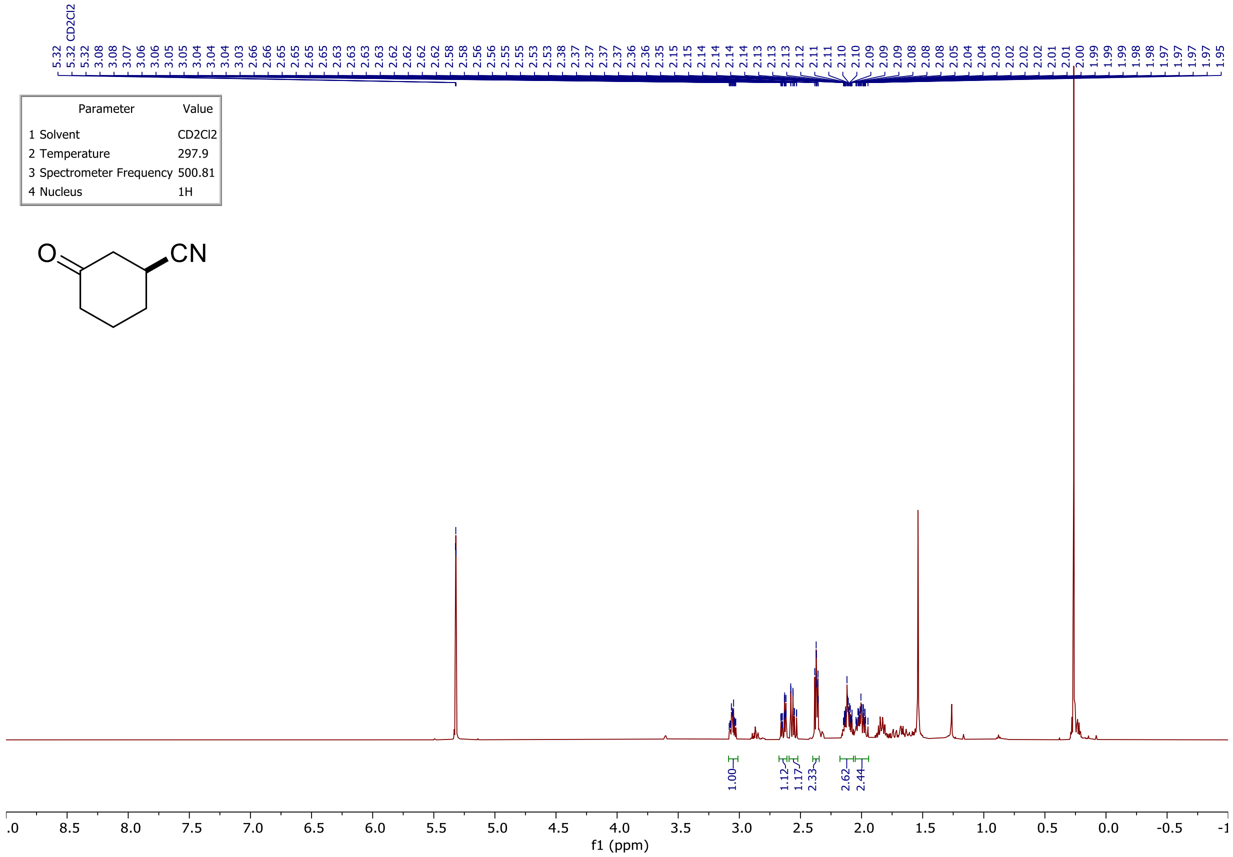

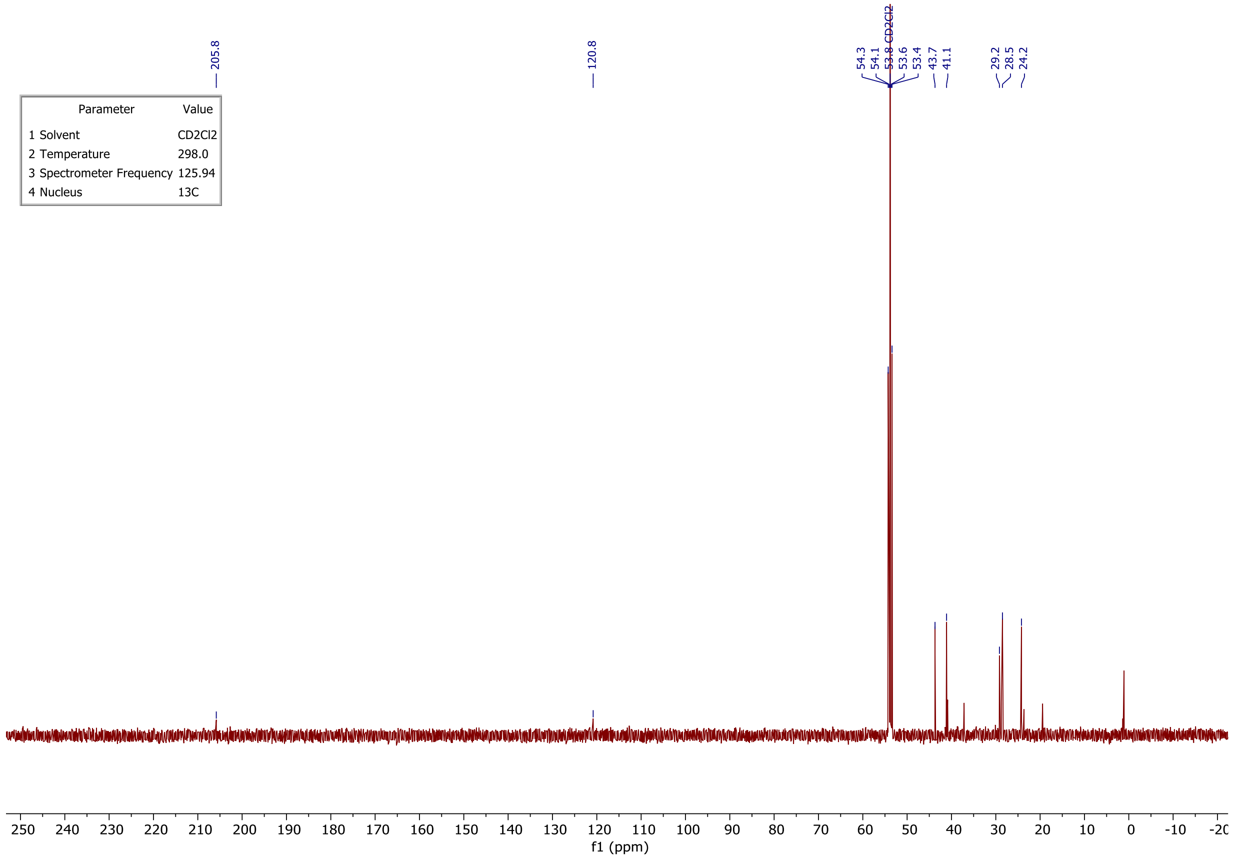

This sample is a mixture of the desired product and bis-adduct. From ^{1}H NMR analysis, the ratio of the two product was 4:1. The GC traces and MS spectra are shown below:

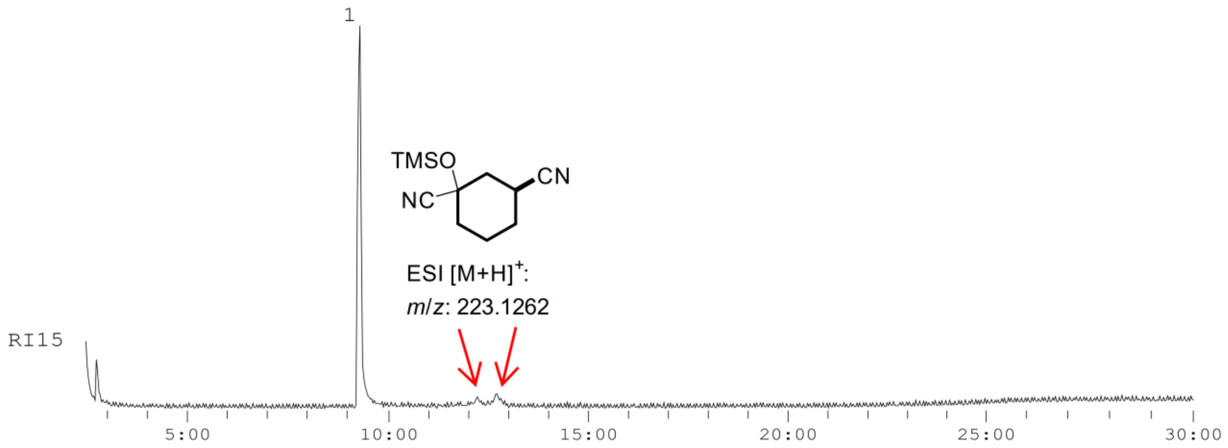

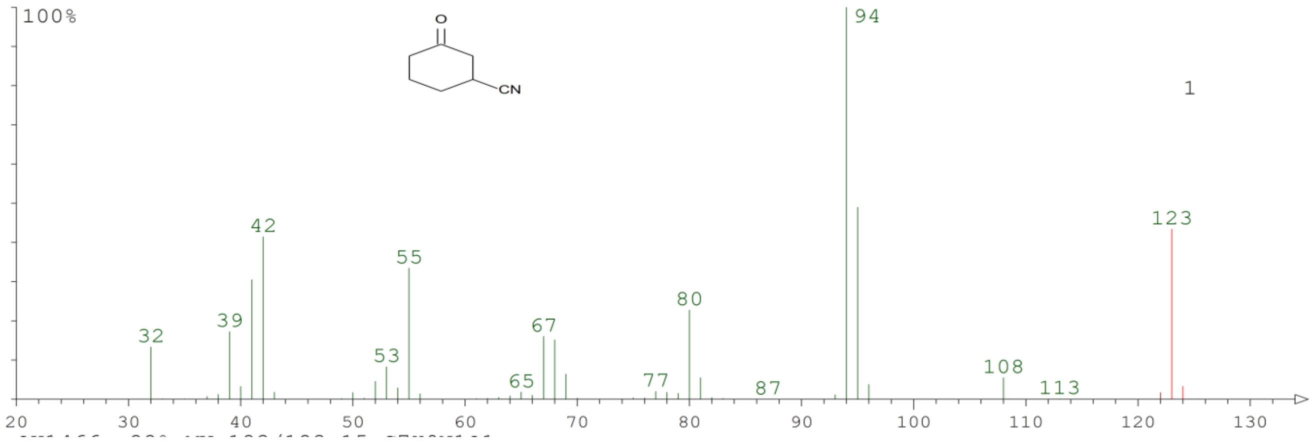

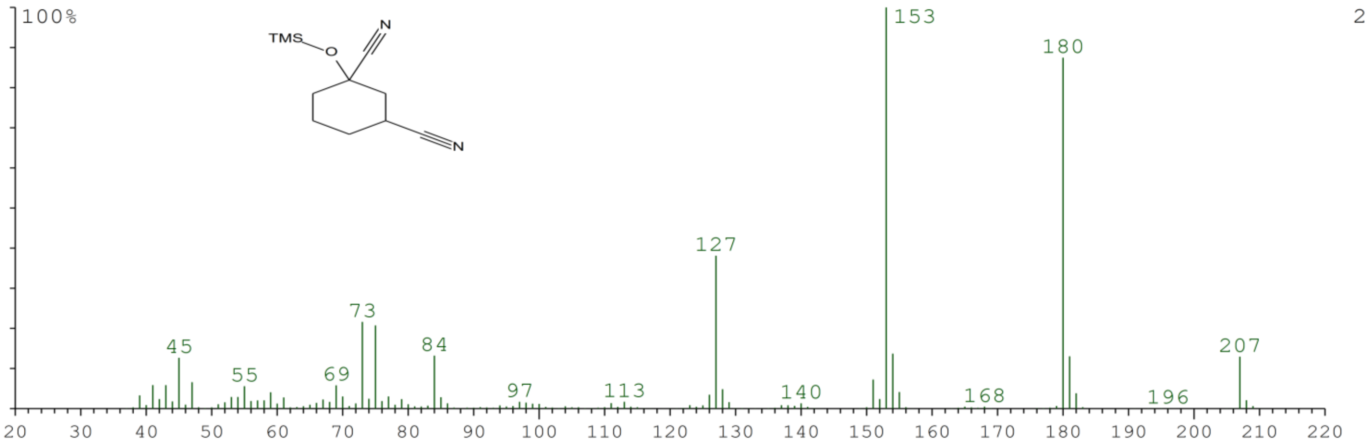

2-methyl-3-phenyl-2-((trimethylsilyl)oxy)propanenitrile 19

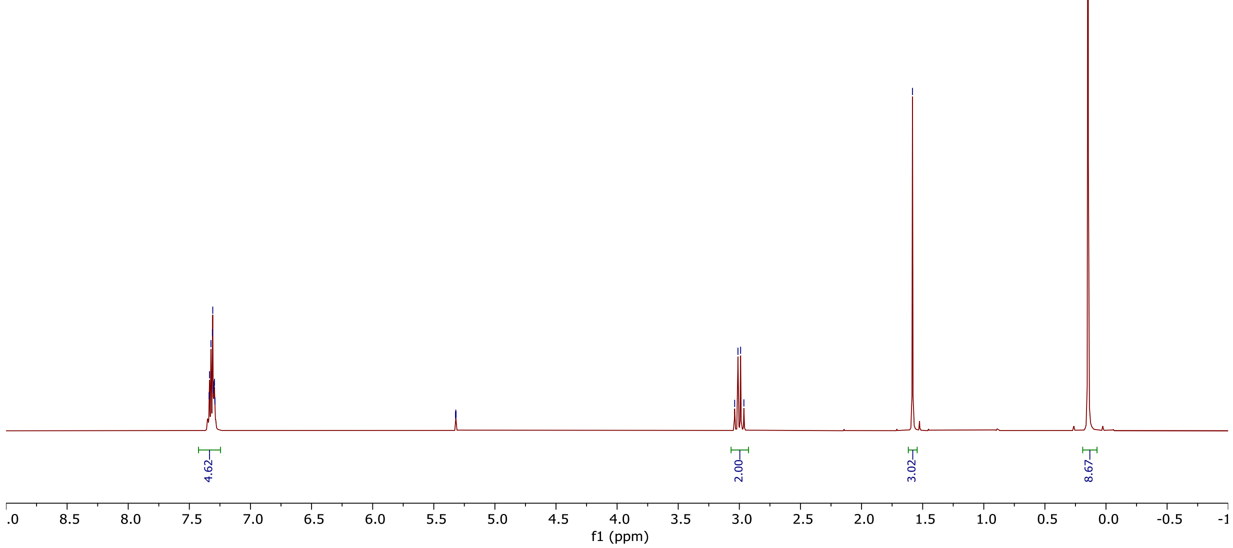

Parameter	Value
1 Solvent	CD2Cl2
2 Temperature	297.9
3 Spectrometer Frequency	500.81
4 Nucleus	1H

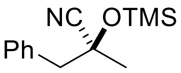

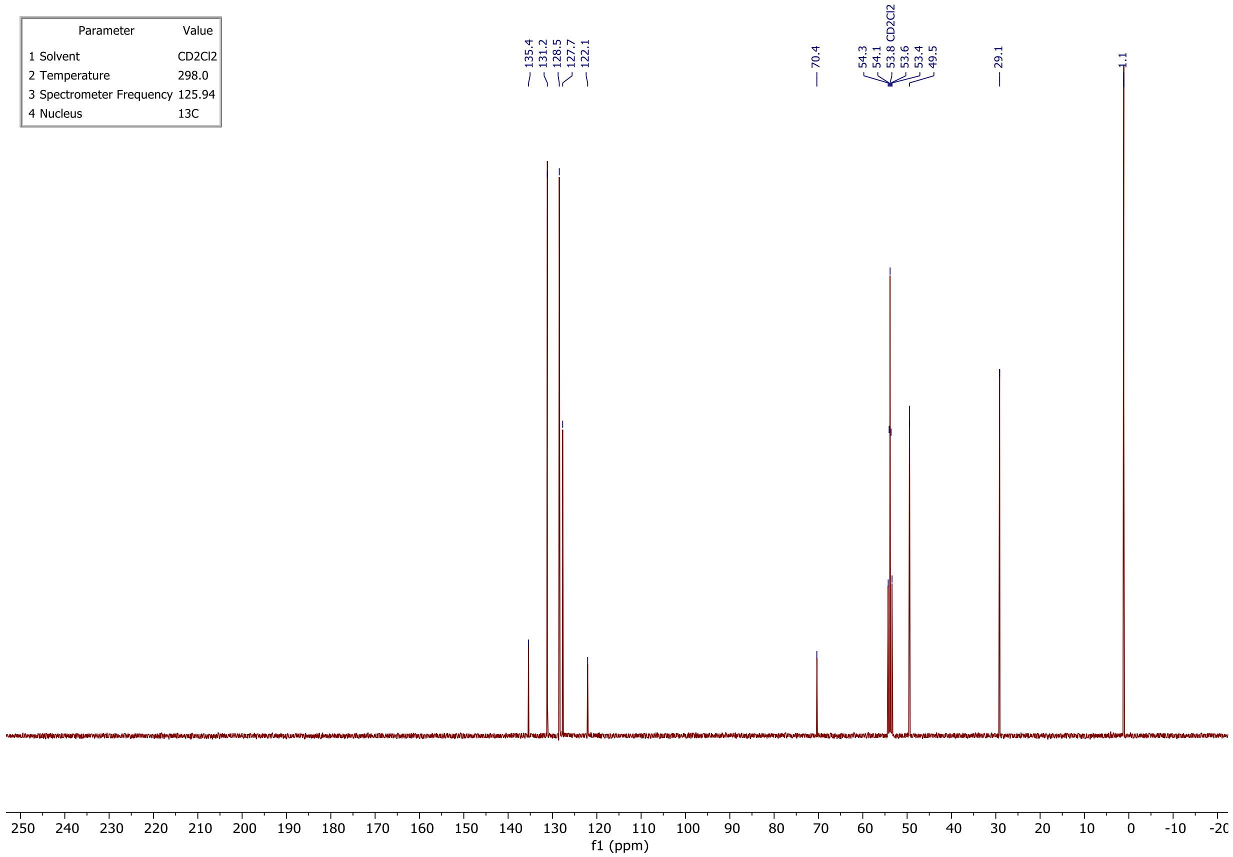

Parameter	Value
1 Solvent	CD2Cl2
2 Temperature	298.0
3 Spectrometer Frequency	125.94
4 Nucleus	13C

2-methyl-3-(p-tolyl)-2-((trimethylsilyl)oxy)propanenitrile 20

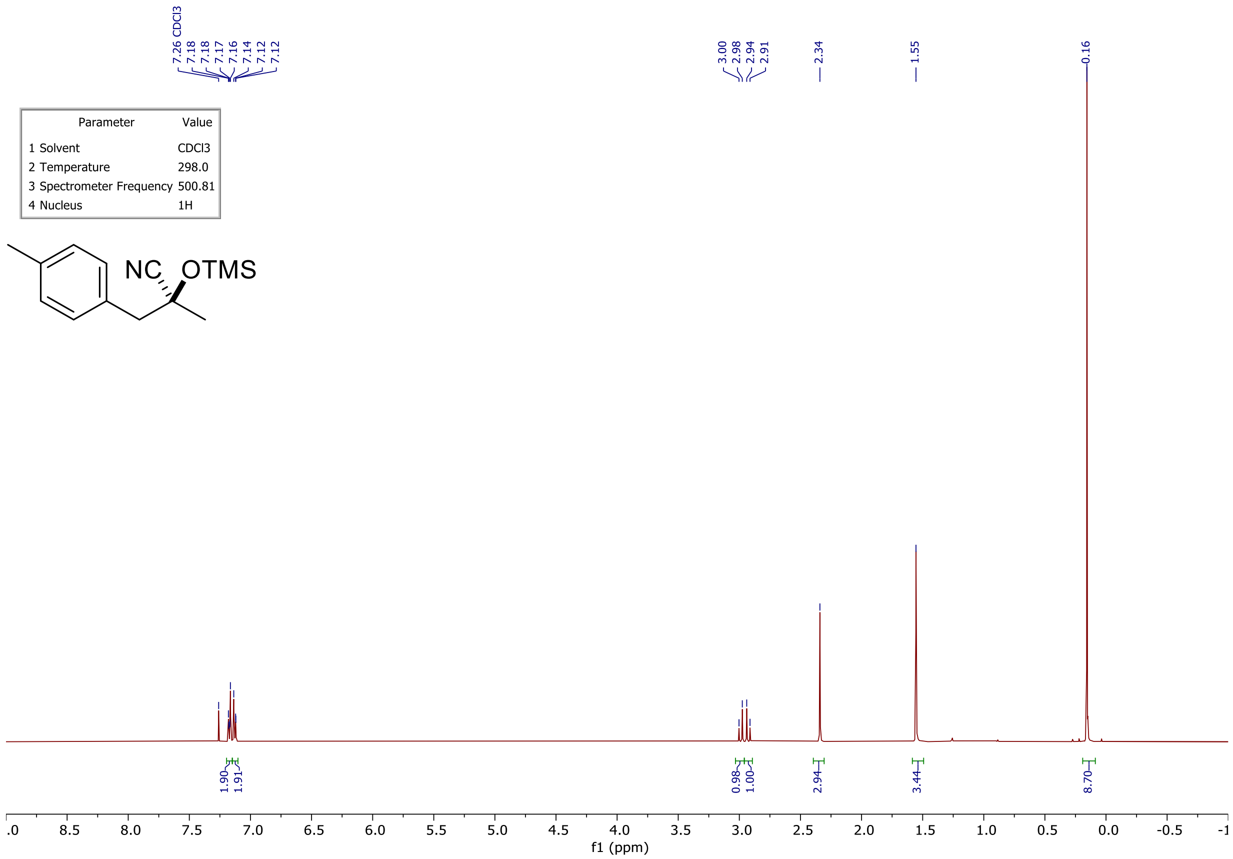

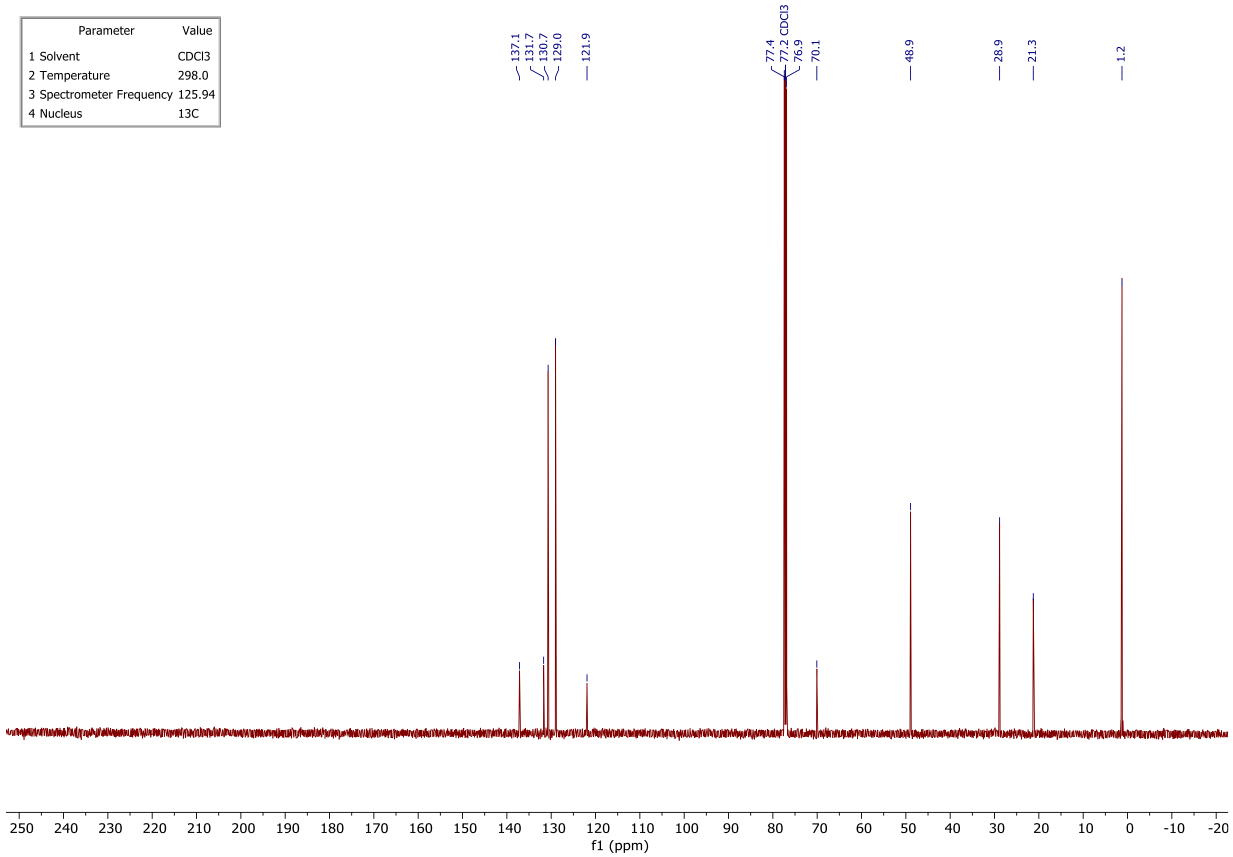

3-(4-methoxyphenyl)-2-methyl-2-((trimethylsilyl)oxy)propanenitrile 21

Parameter	Value
1 Solvent	CD2Cl2
2 Temperature	297.8
3 Spectrometer Frequency	500.81
4 Nucleus	1H

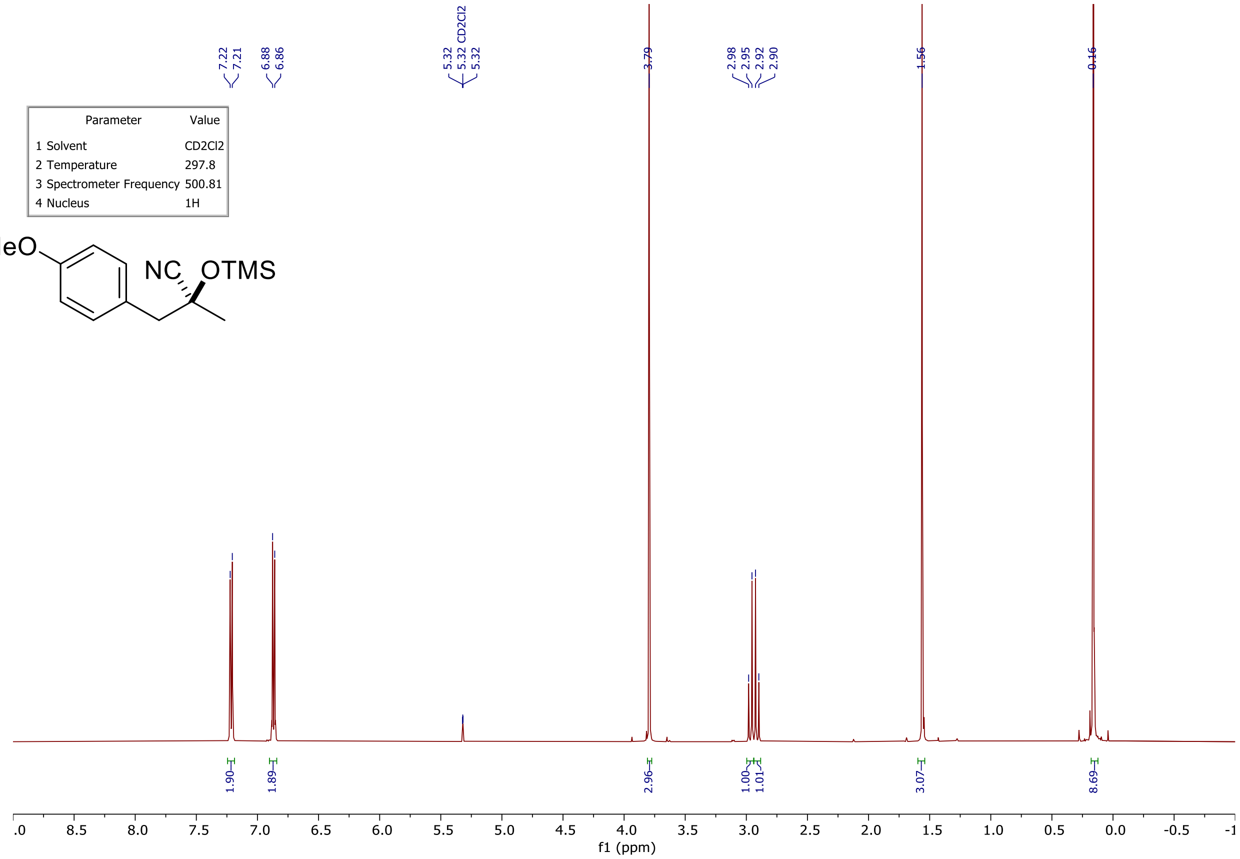

Parameter	Value
1 Solvent	CD2Cl2
2 Temperature	298.0
3 Spectrometer Frequency	125.94
4 Nucleus	13C

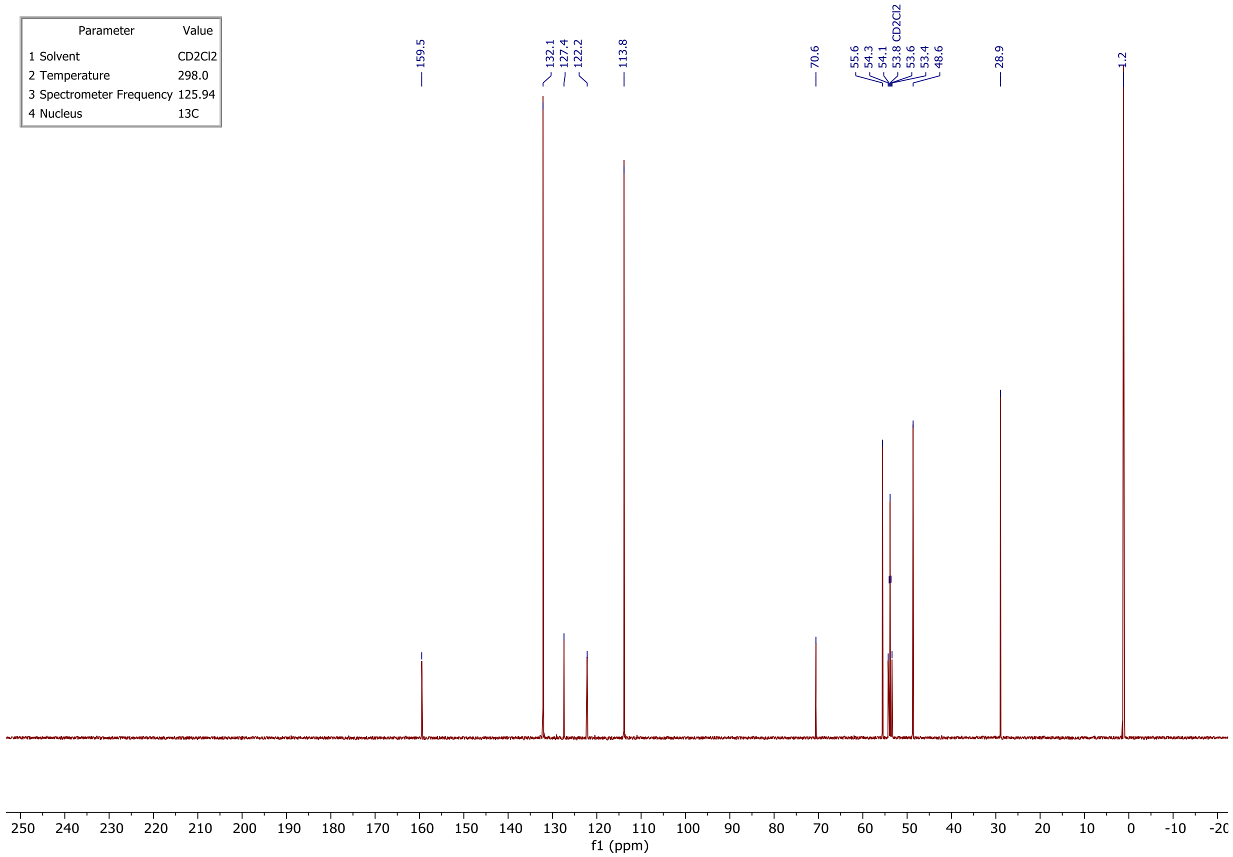

2-methyl-3-(naphthalen-2-yl)-2-((trimethylsilyl)oxy)propanenitrile 22

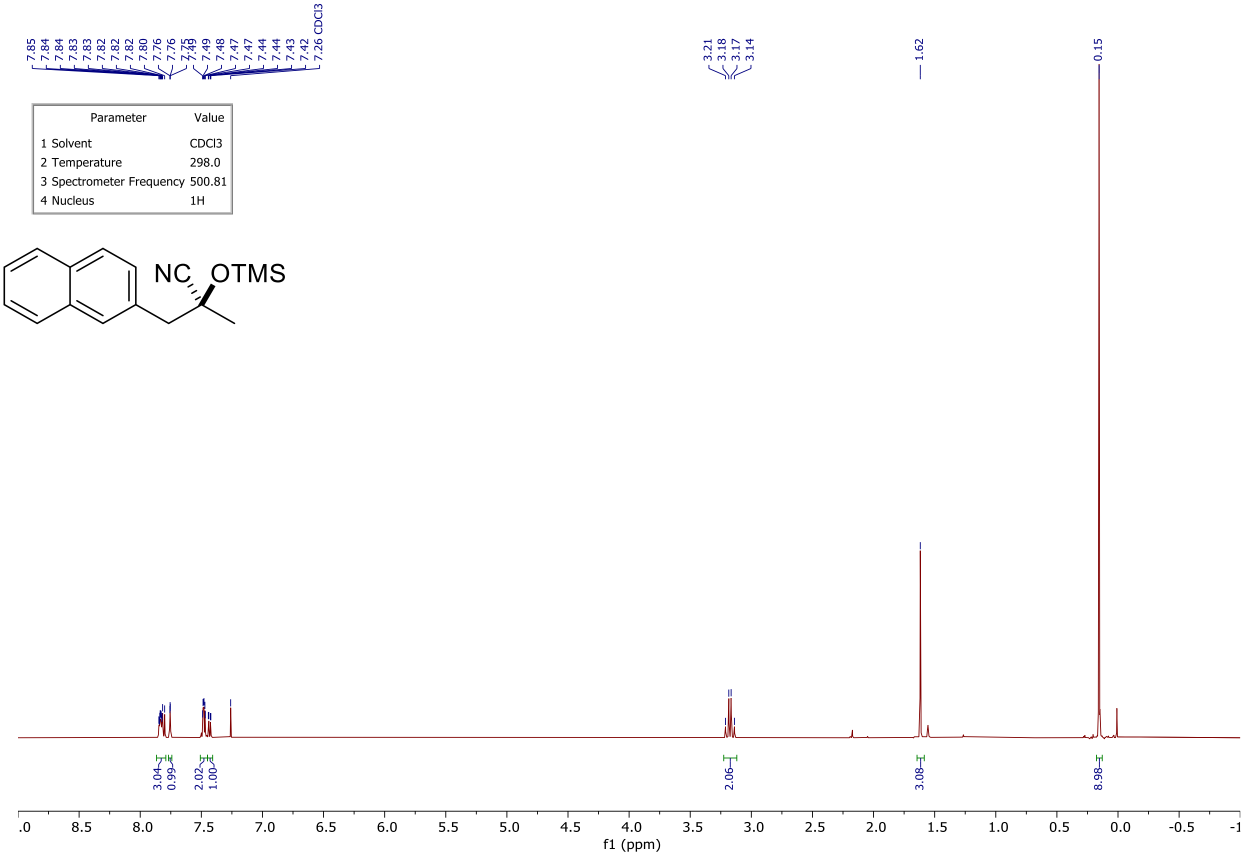

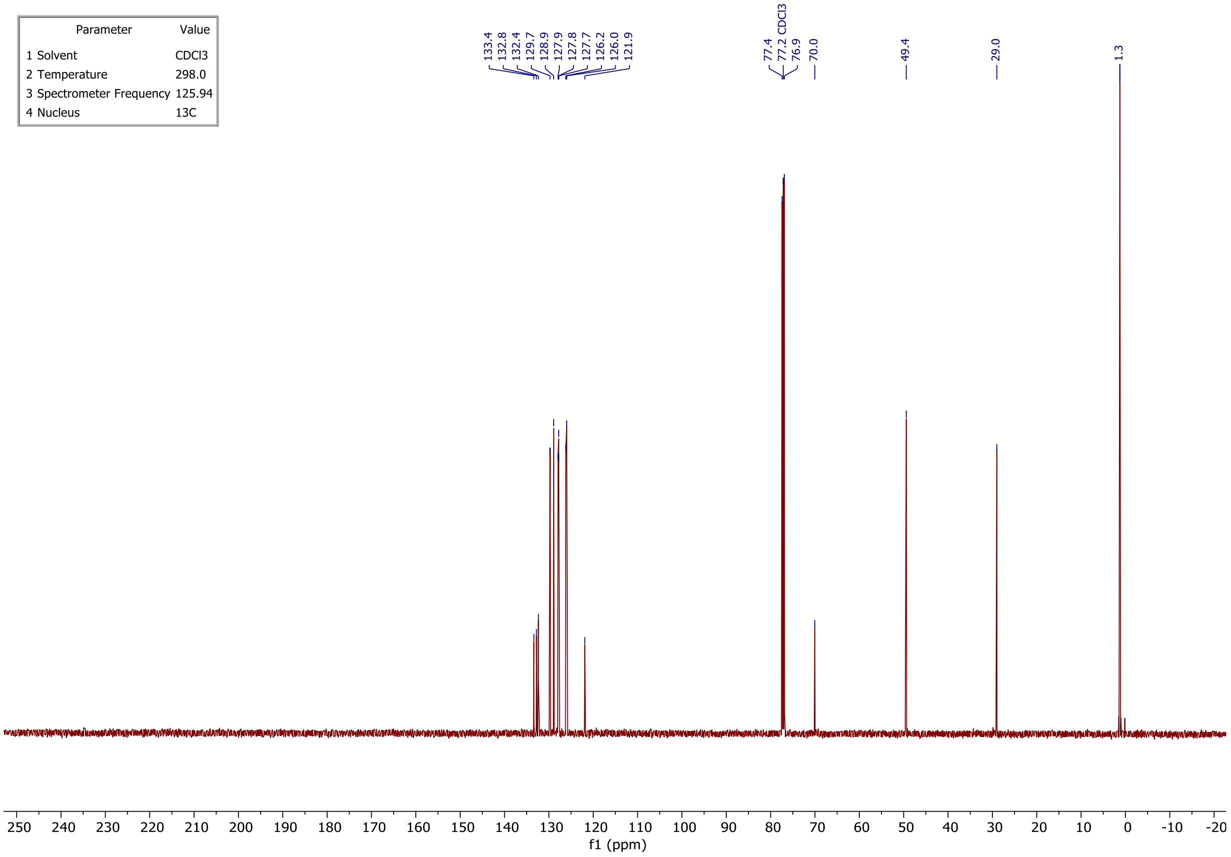

2-methyl-4-phenyl-2-((trimethylsilyl)oxy)butanenitrile 23

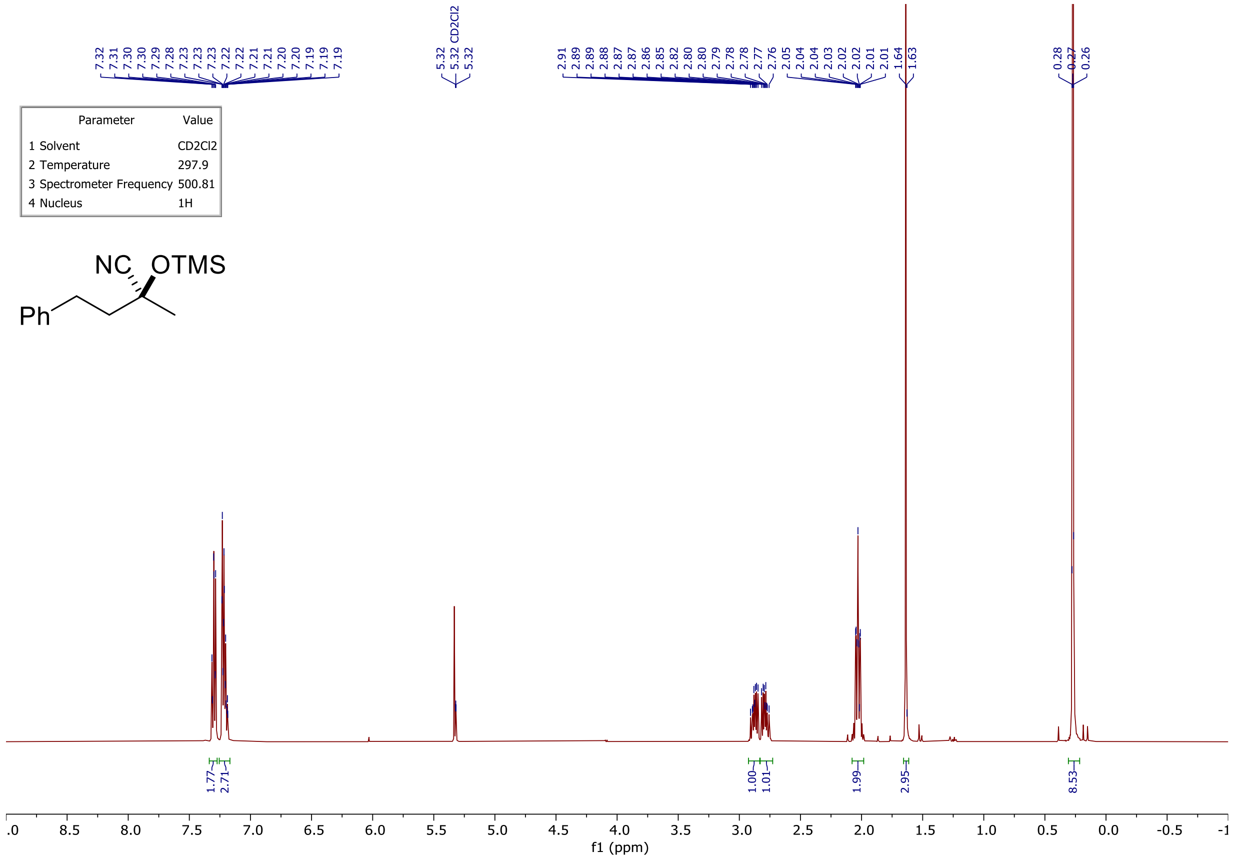

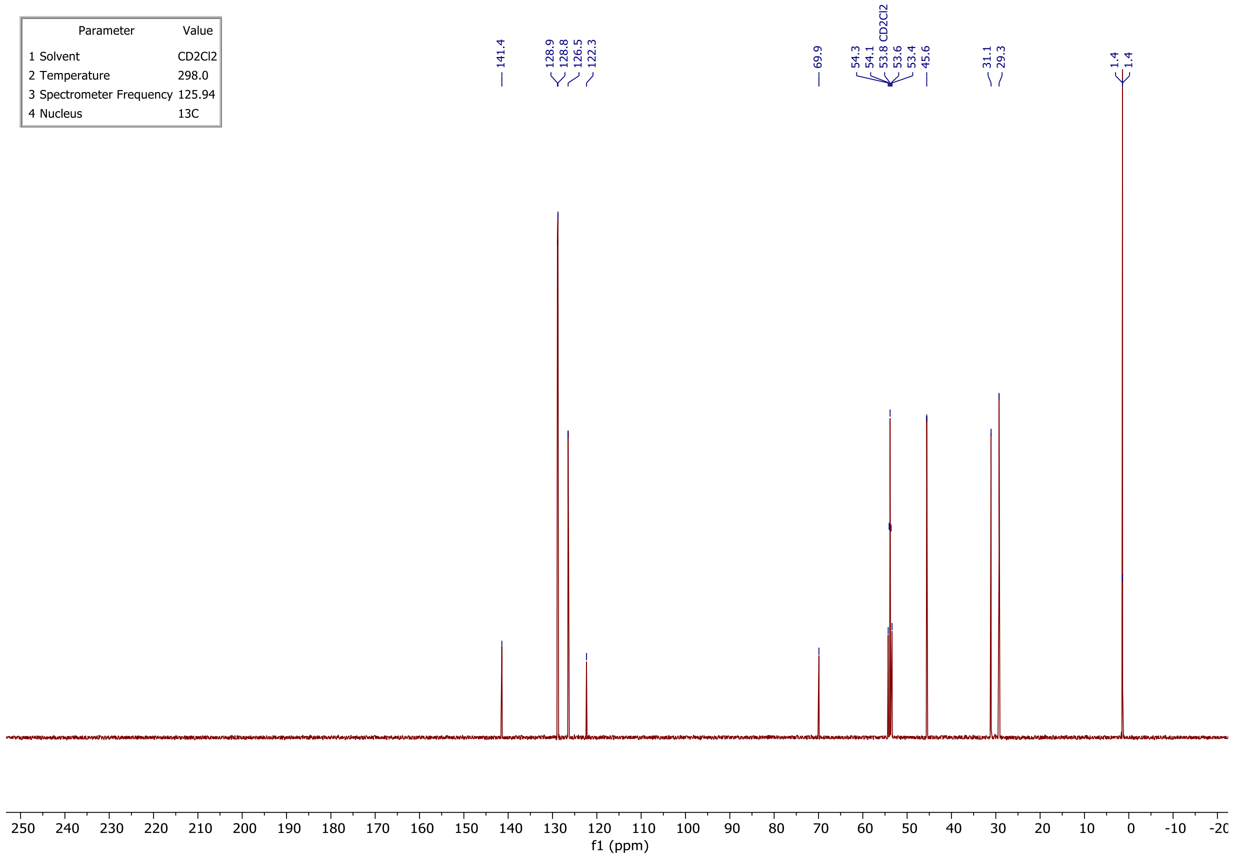

2-methyl-4-(p-tolyl)-2-((trimethylsilyl)oxy)butanenitrile 24

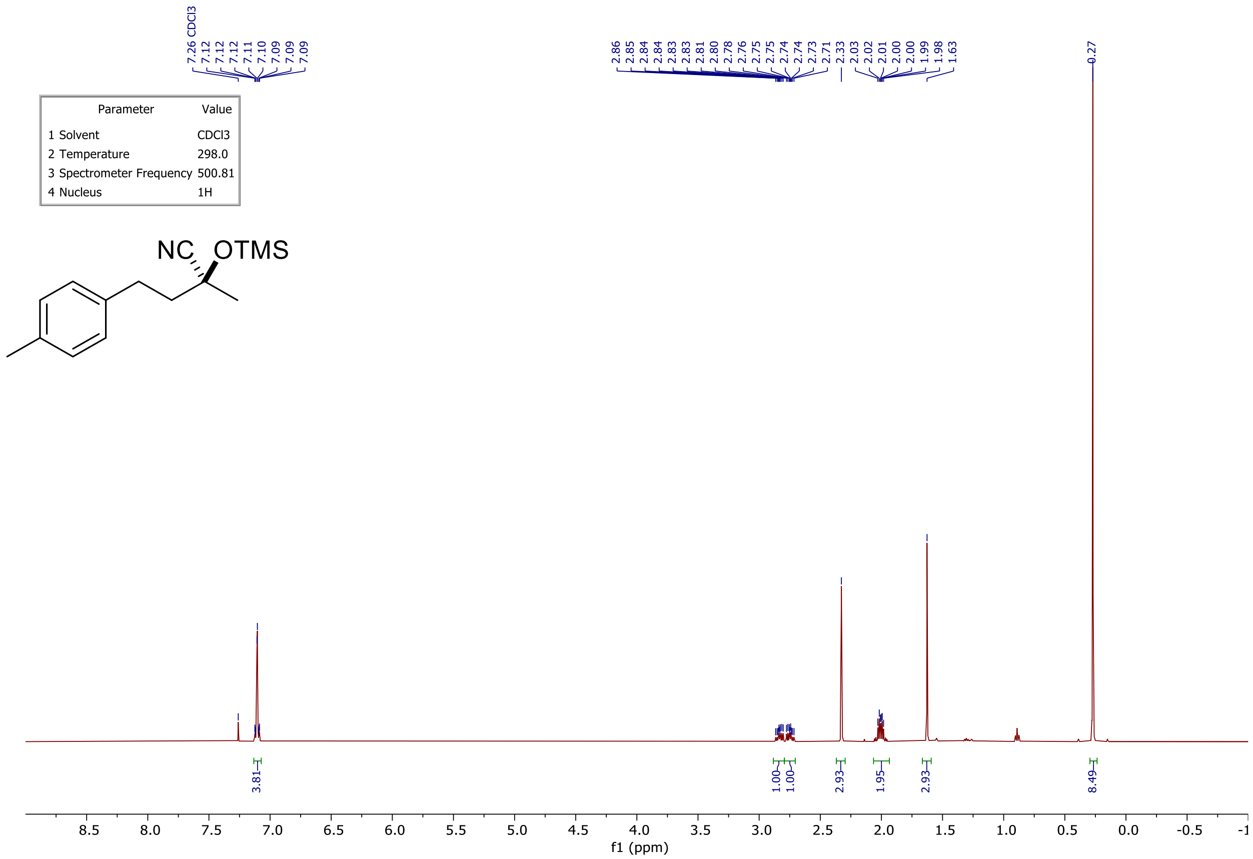

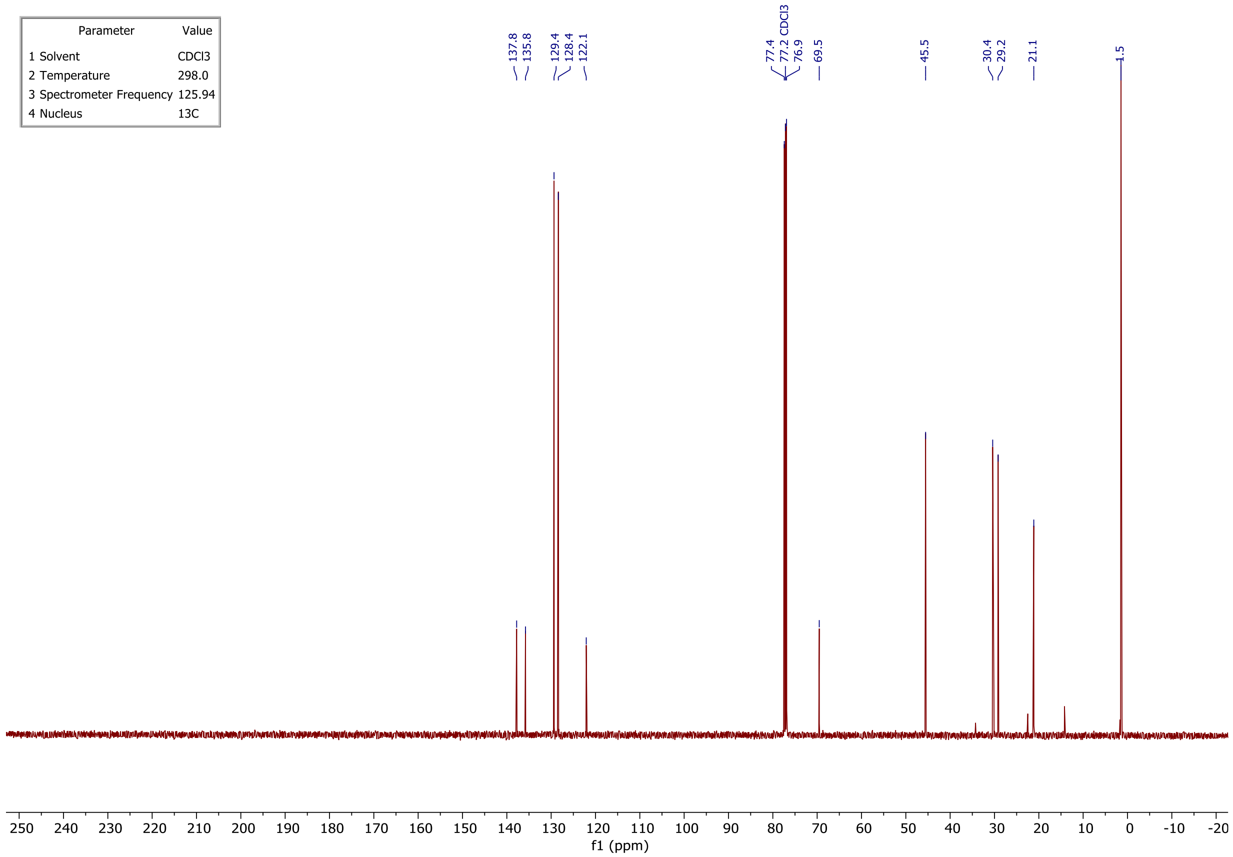

4-(4-methoxyphenyl)-2-methyl-2-((trimethylsilyl)oxy)butanenitrile 25

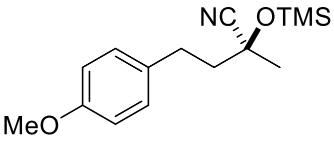

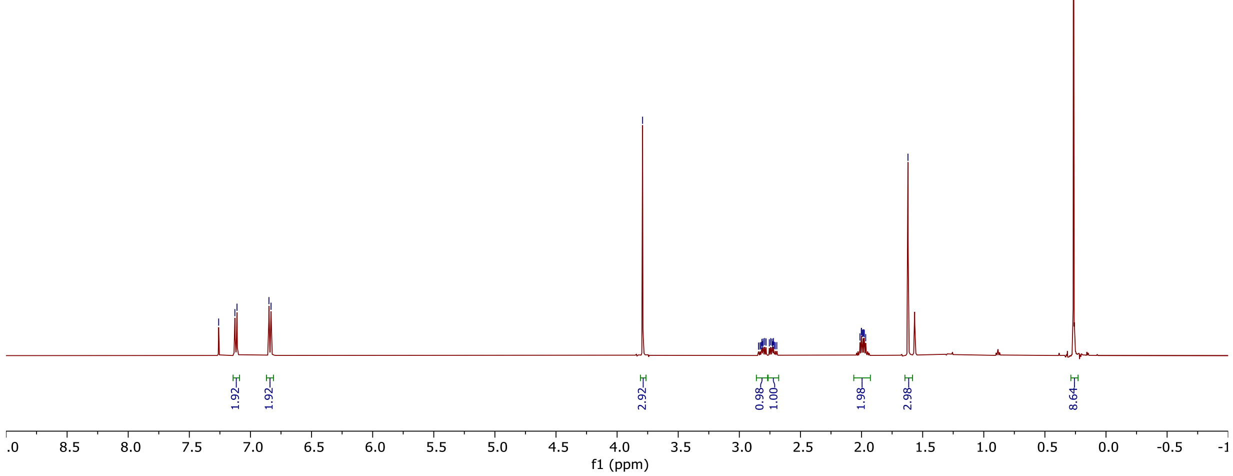

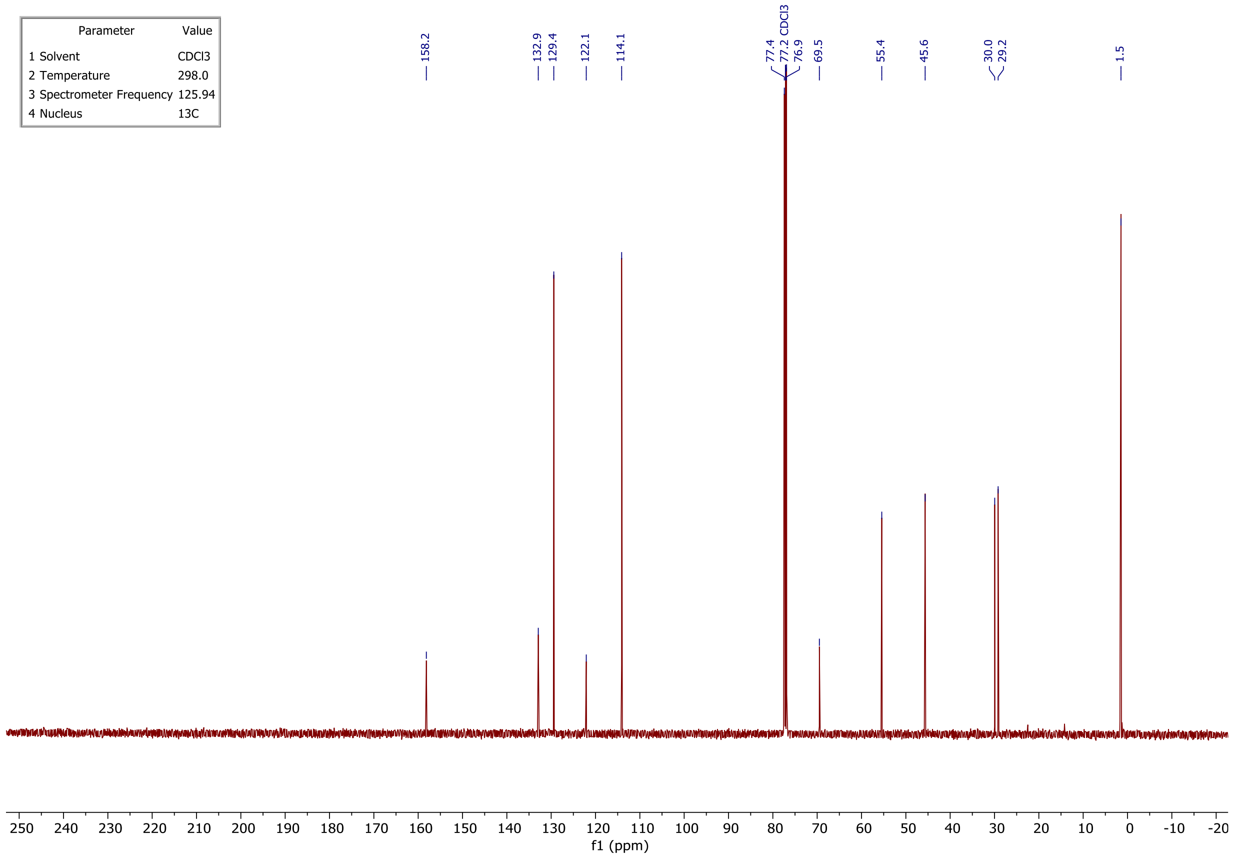

4-(4-chlorophenyl)-2-methyl-2-((trimethylsilyl)oxy)butanenitrile 26

Parameter	Value
1 Solvent	CDCl3
2 Temperature	298.0
3 Spectrometer Frequency	500.81
4 Nucleus	1H

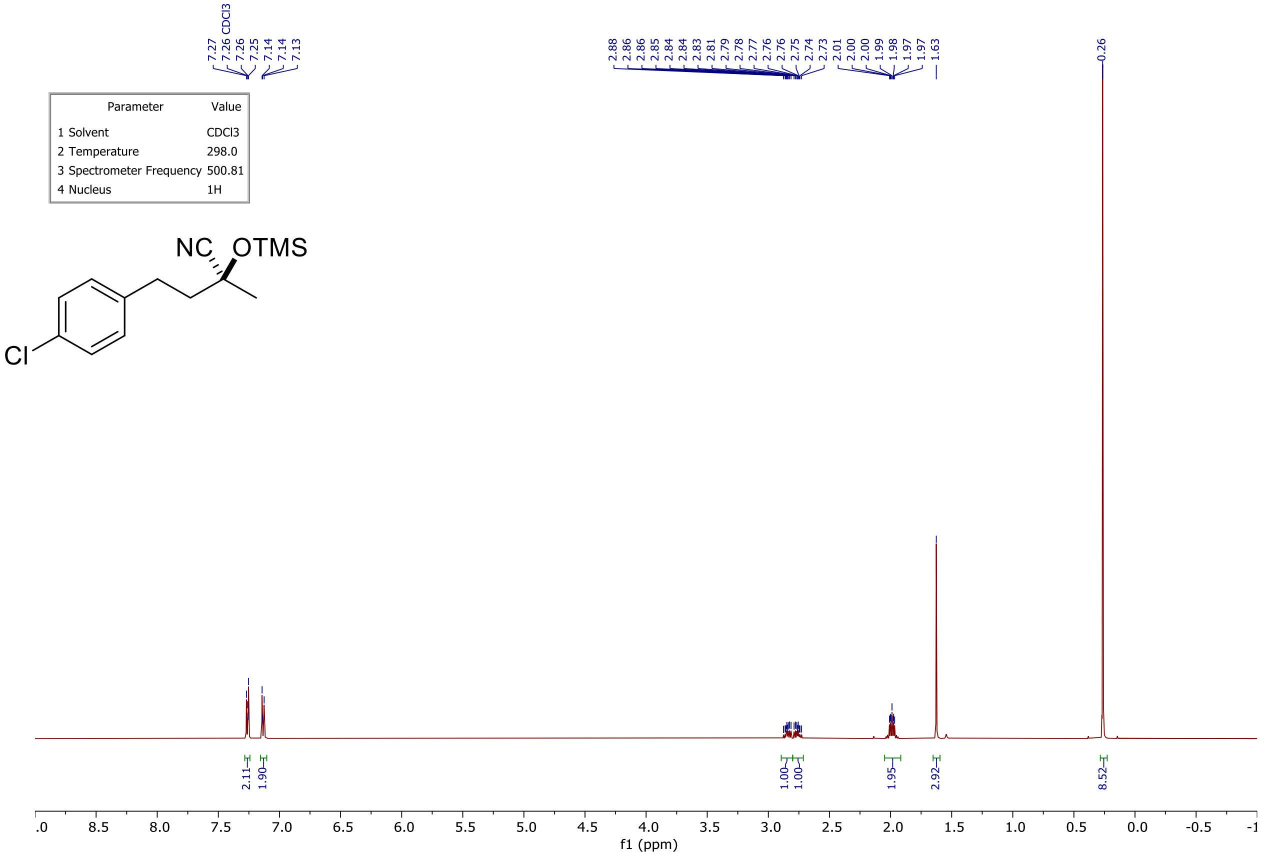

Parameter	Value
1 Solvent	CDCl3
2 Temperature	298.0
3 Spectrometer Frequency	125.94
4 Nucleus	13C

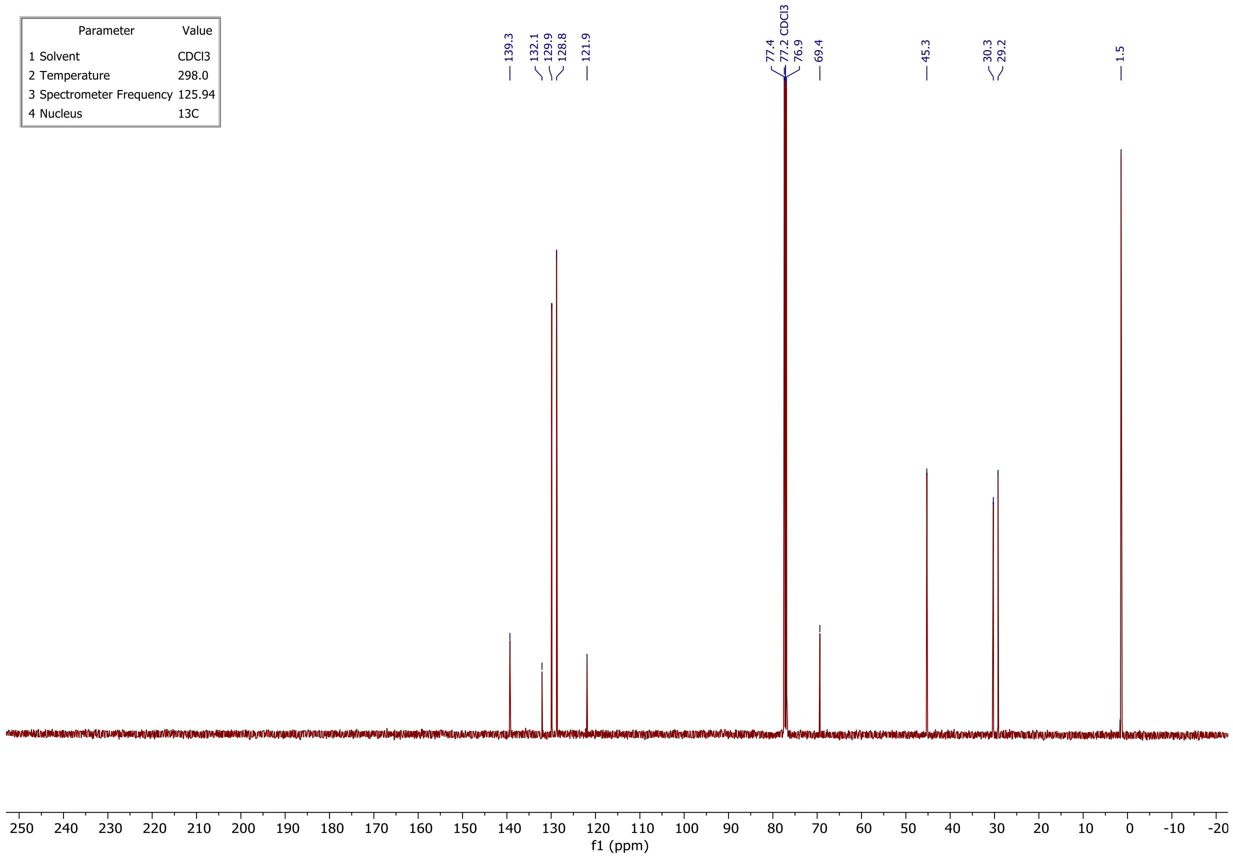

4-(4-bromophenyl)-2-methyl-2-((trimethylsilyl)oxy)butanenitrile 27

Parameter	Value
1 Solvent	CDCl3
2 Temperature	298.0
3 Spectrometer Frequency	500.81
4 Nucleus	1H

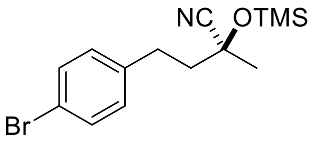

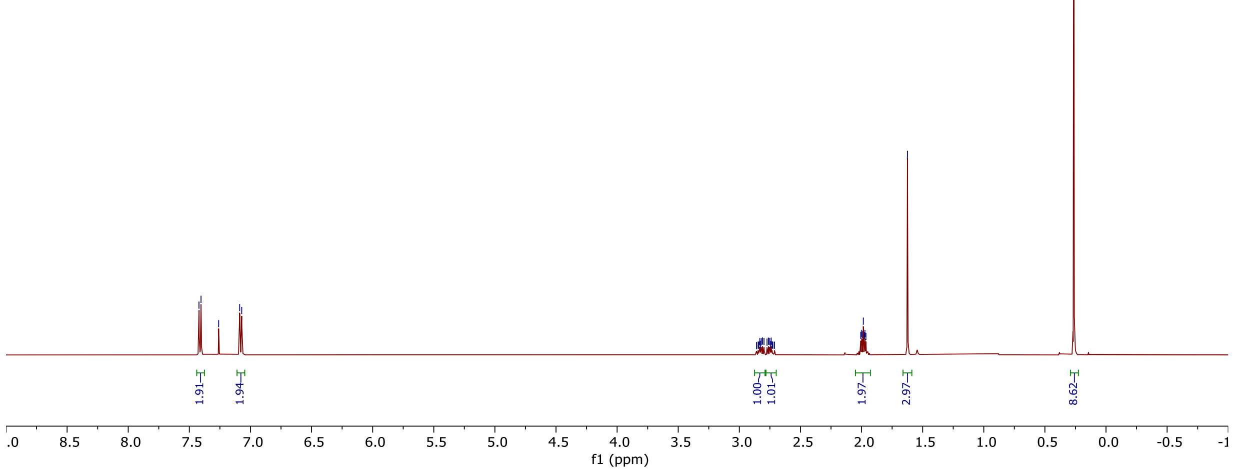

Parameter	Value
1 Solvent	CDCl3
2 Temperature	298.0
3 Spectrometer Frequency	125.94
4 Nucleus	13C

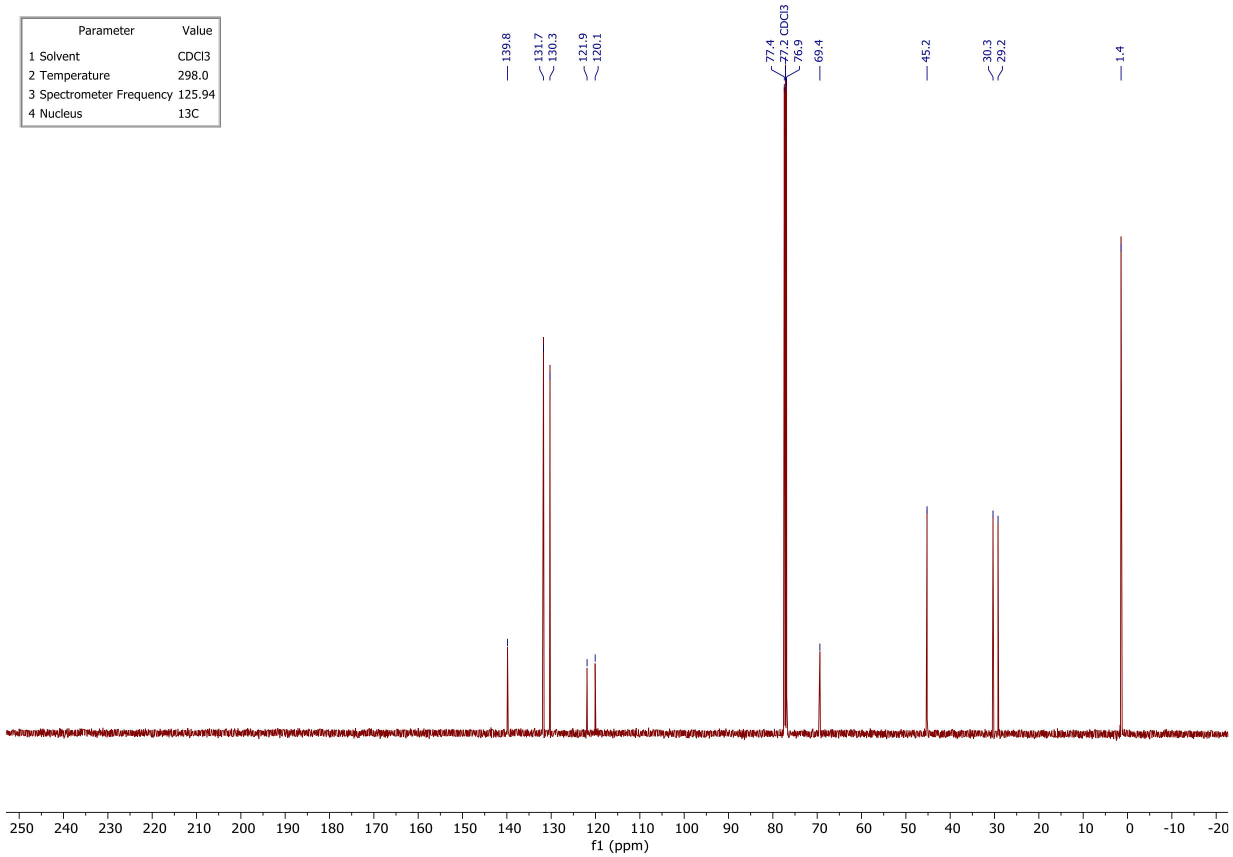

2-methyl-4-(naphthalen-1-yl)-2-((trimethylsilyl)oxy)butanenitrile 28

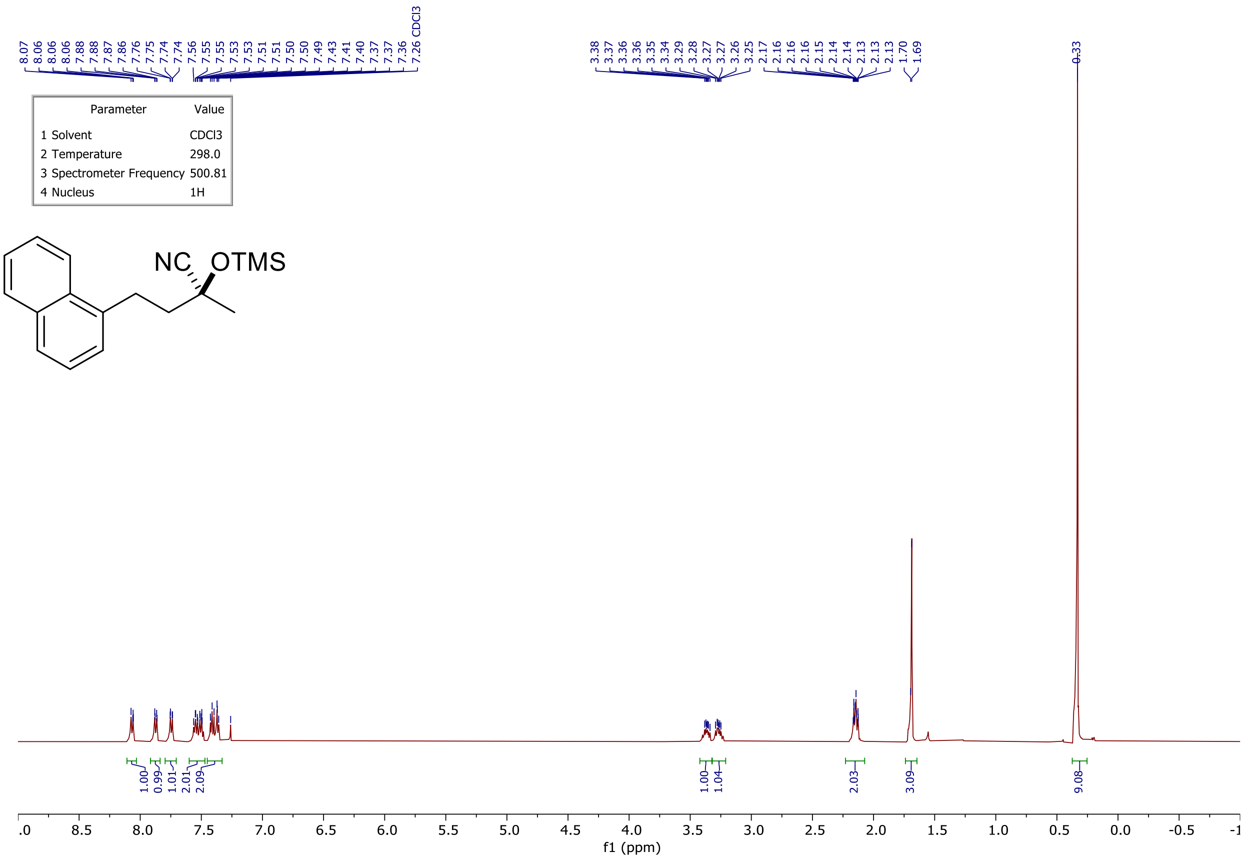

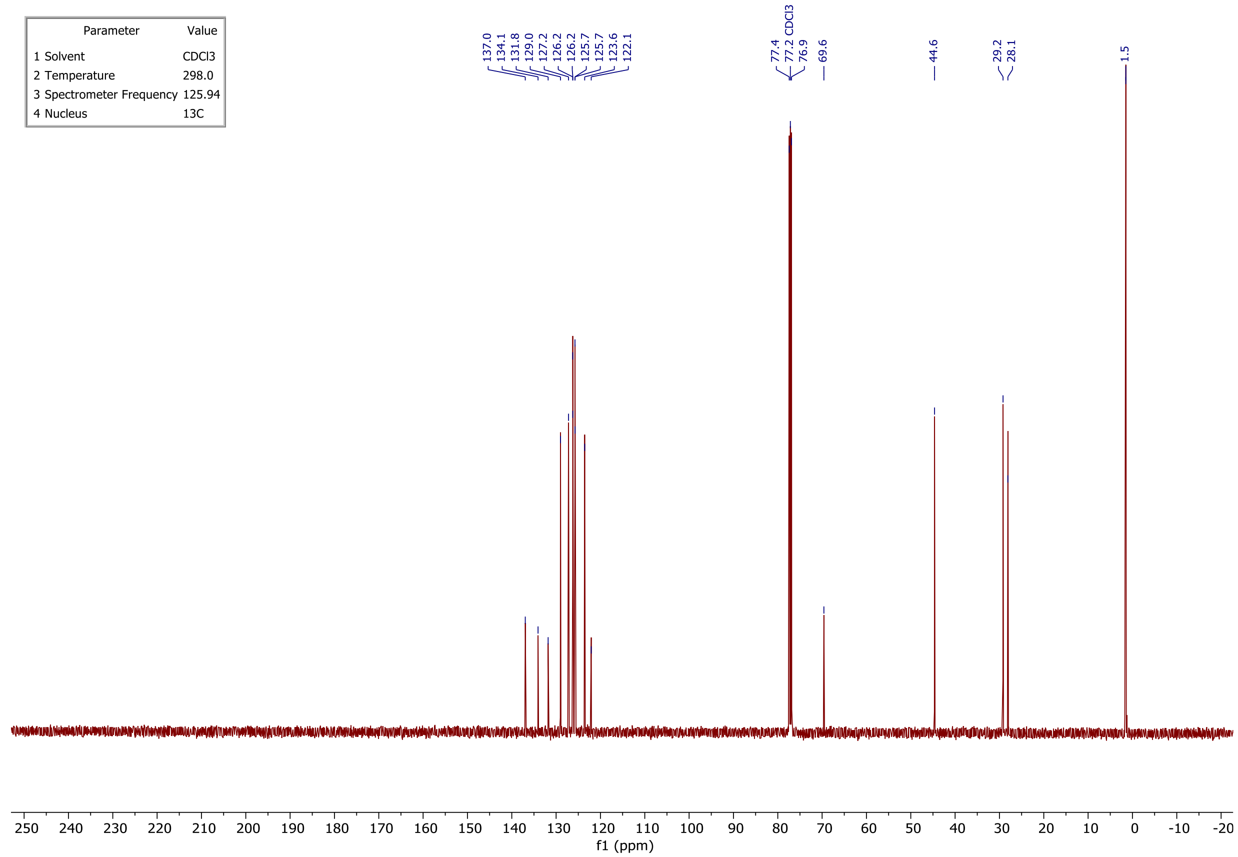

2-methyl-4-(naphthalen-2-yl)-2-((trimethylsilyl)oxy)butanenitrile 29

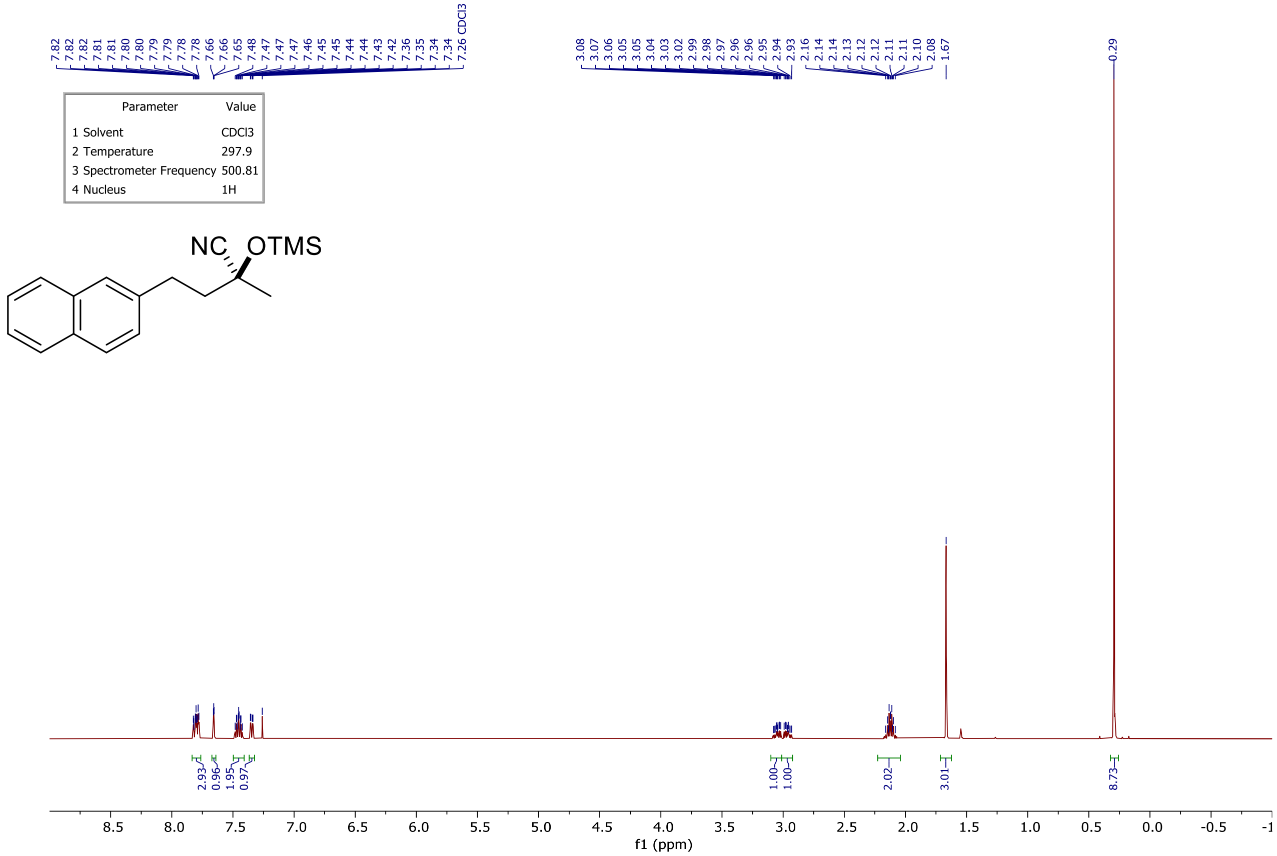

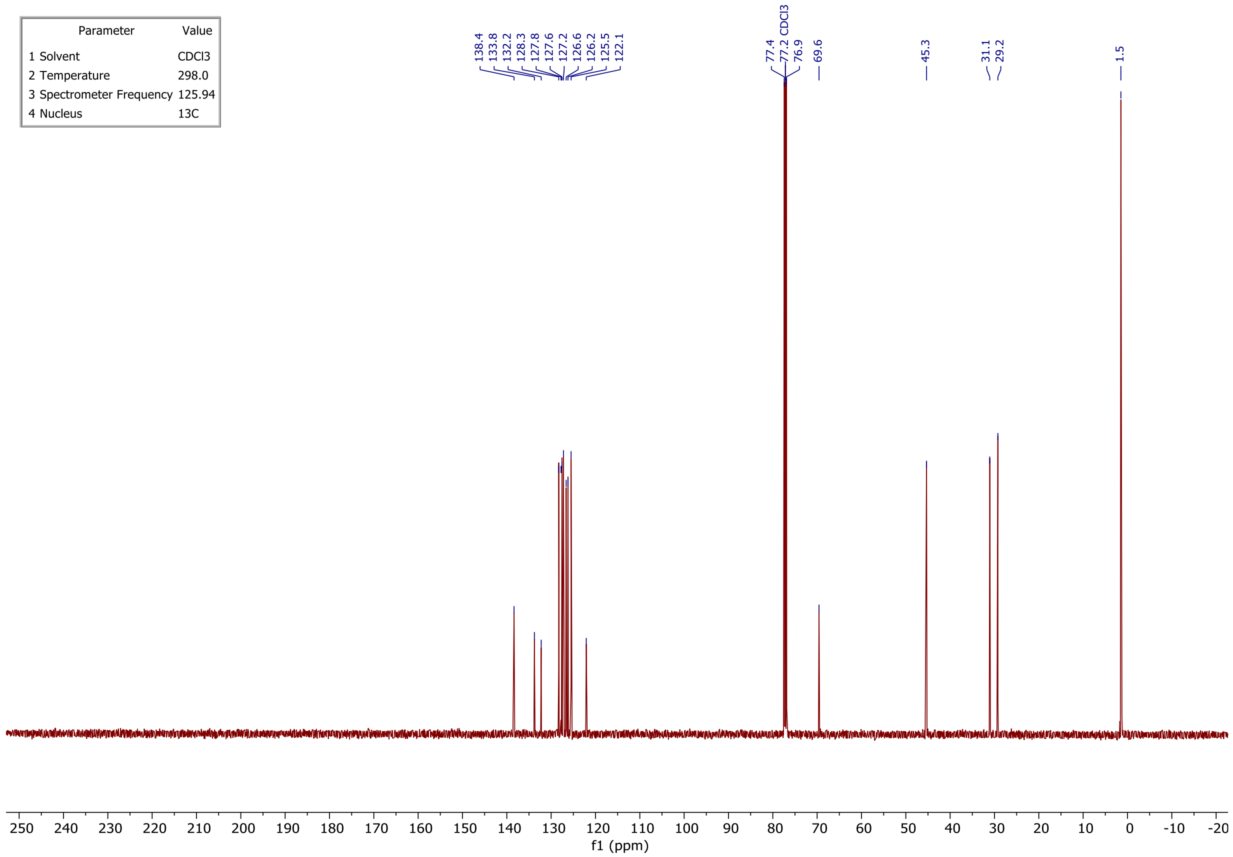

(*S*)-2-phenyl-2-((trimethylsilyl)oxy)propanenitrile 30

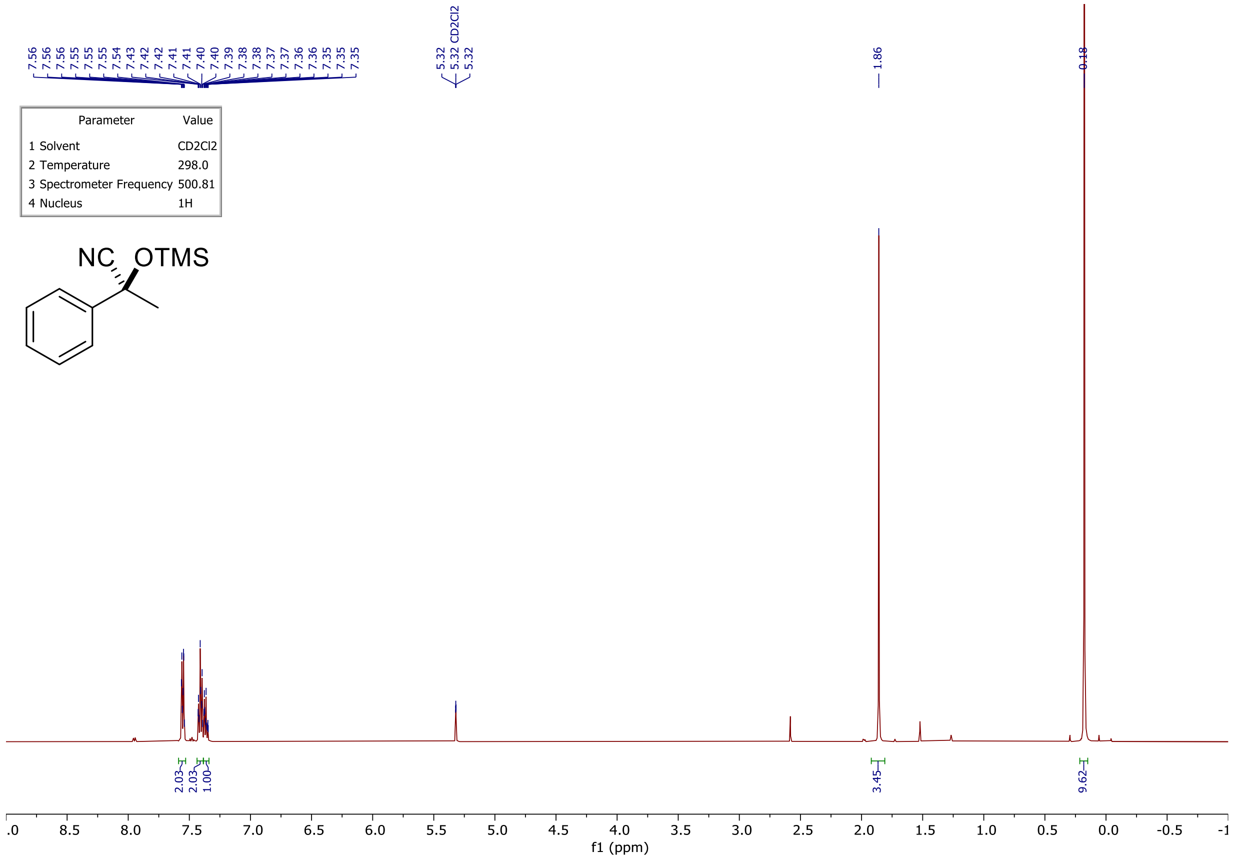

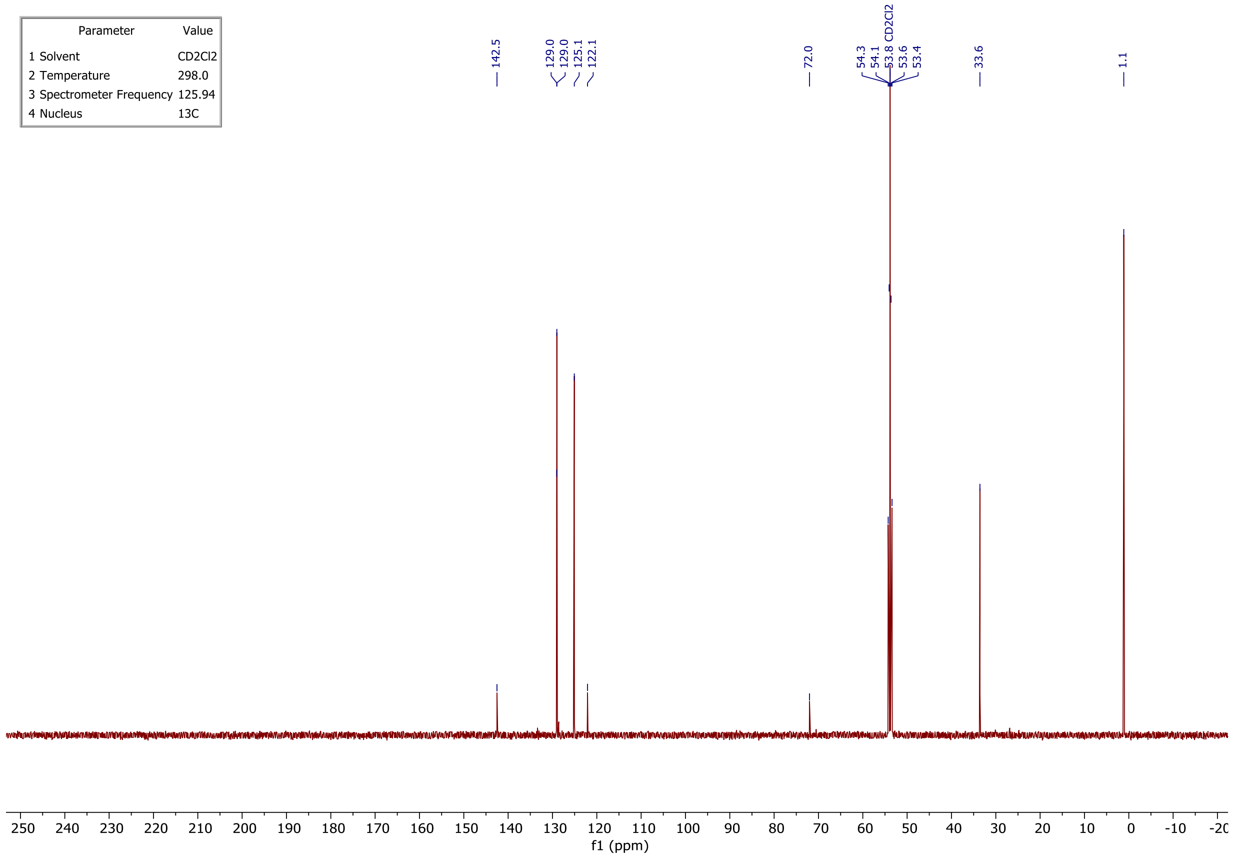

2-(p-tolyl)-2-((trimethylsilyl)oxy)propanenitrile 31

Parameter	Value
1 Solvent	CD2Cl2
2 Temperature	297.9
3 Spectrometer Frequency	500.81
4 Nucleus	1H

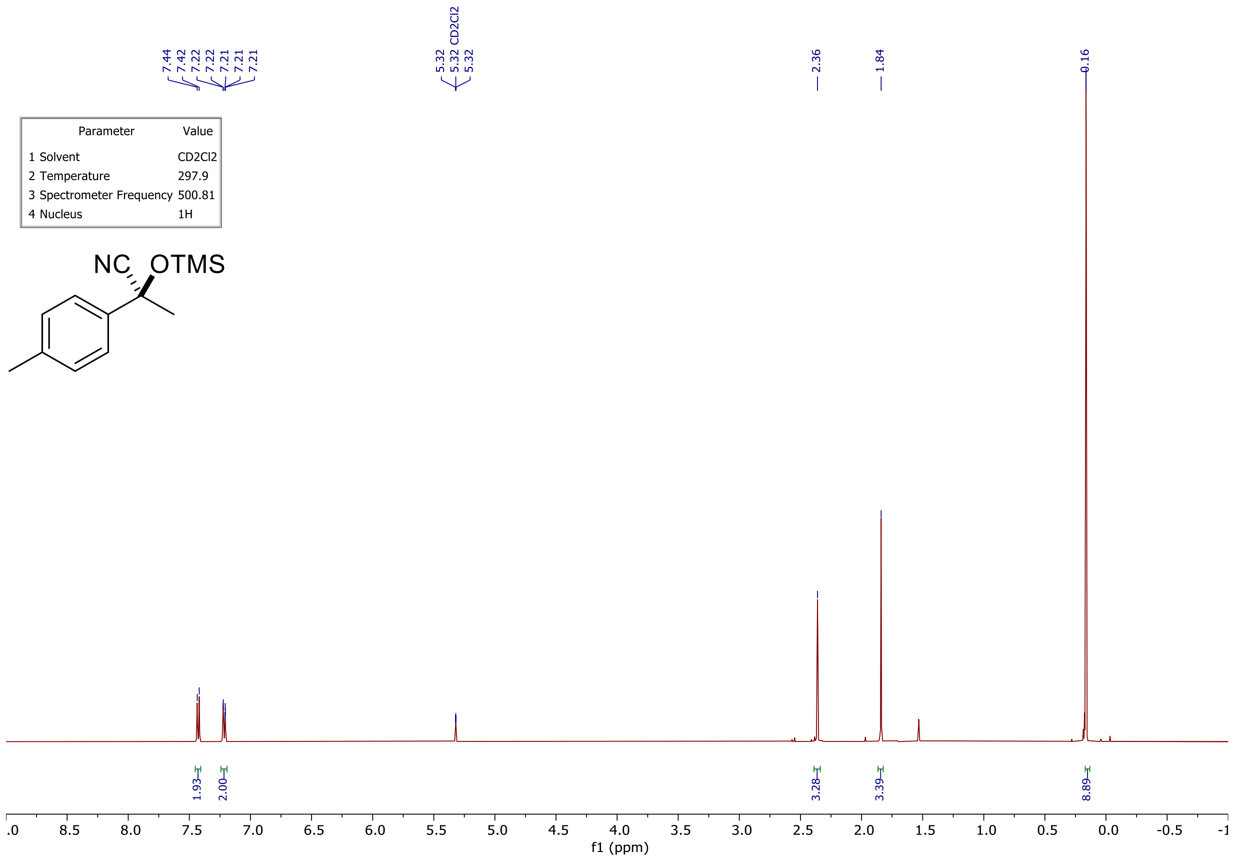

Parameter	Value
1 Solvent	CD2Cl2
2 Temperature	298.0
3 Spectrometer Frequency	125.94
4 Nucleus	13C

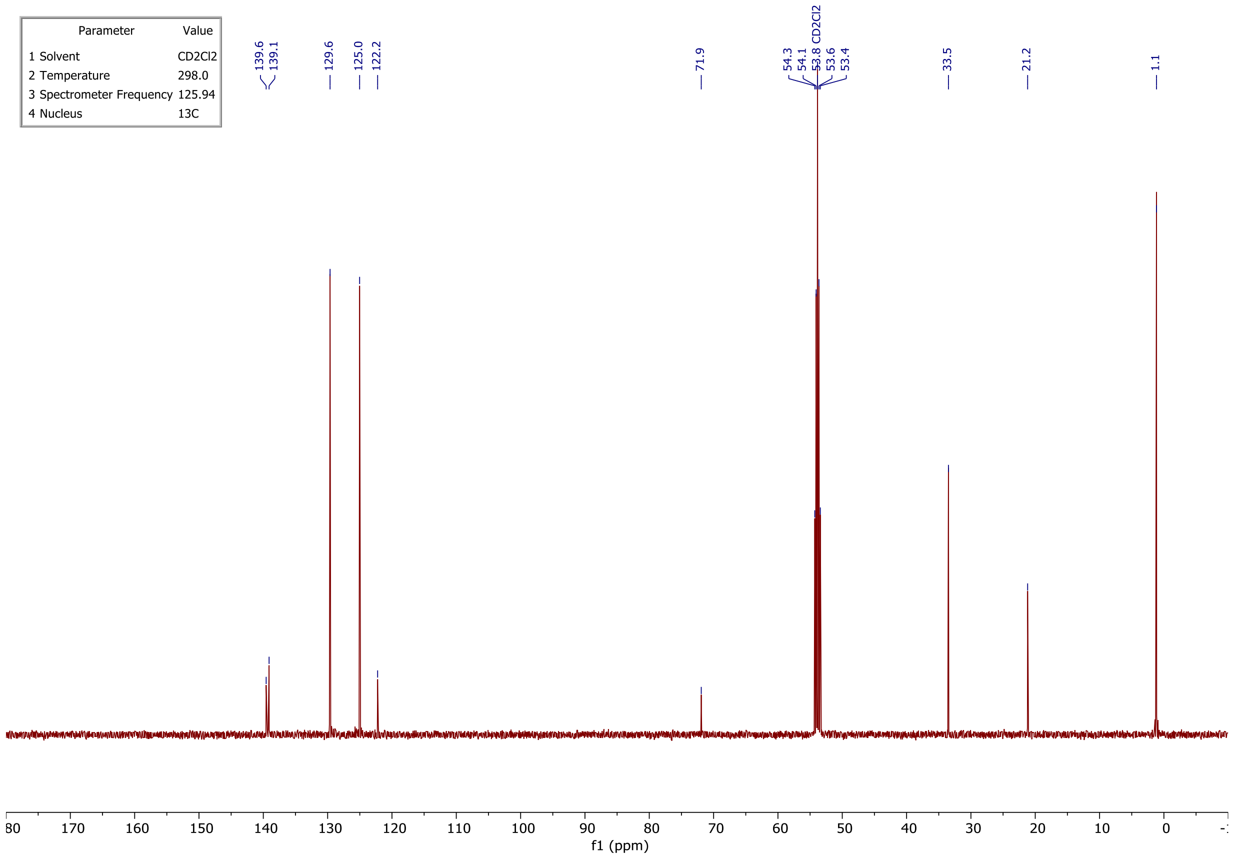

2-(4-fluorophenyl)-2-((trimethylsilyl)oxy)propanenitrile 32

Parameter	Value
1 Solvent	CDCl3
2 Temperature	298.0
3 Spectrometer Frequency	500.81
4 Nucleus	1H

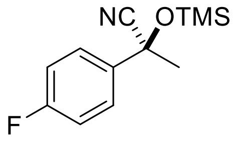

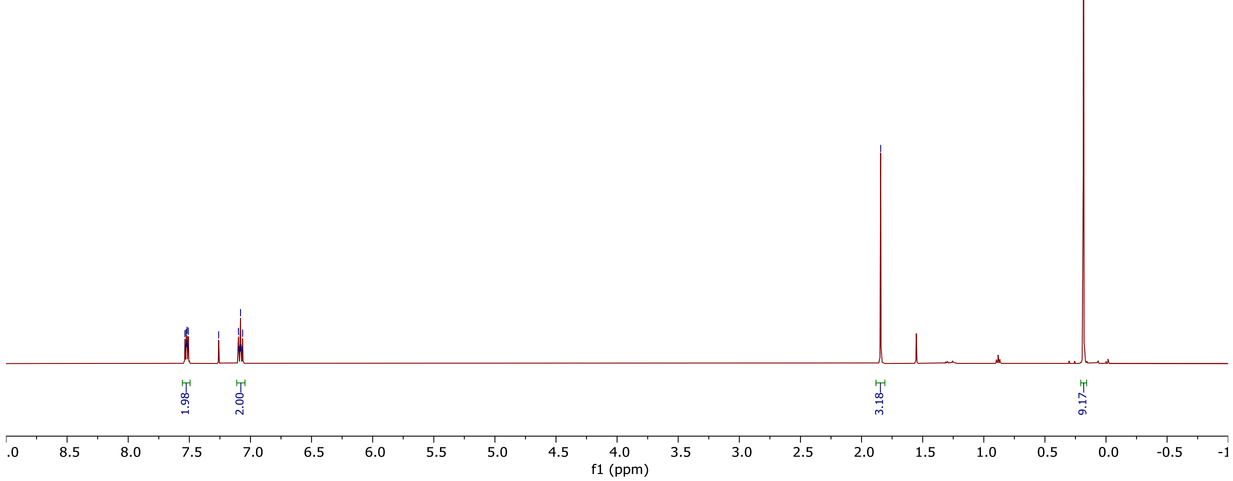

Parameter	Value
1 Solvent	CDCl3
2 Temperature	298.0
3 Spectrometer Frequency	125.94
4 Nucleus	13C

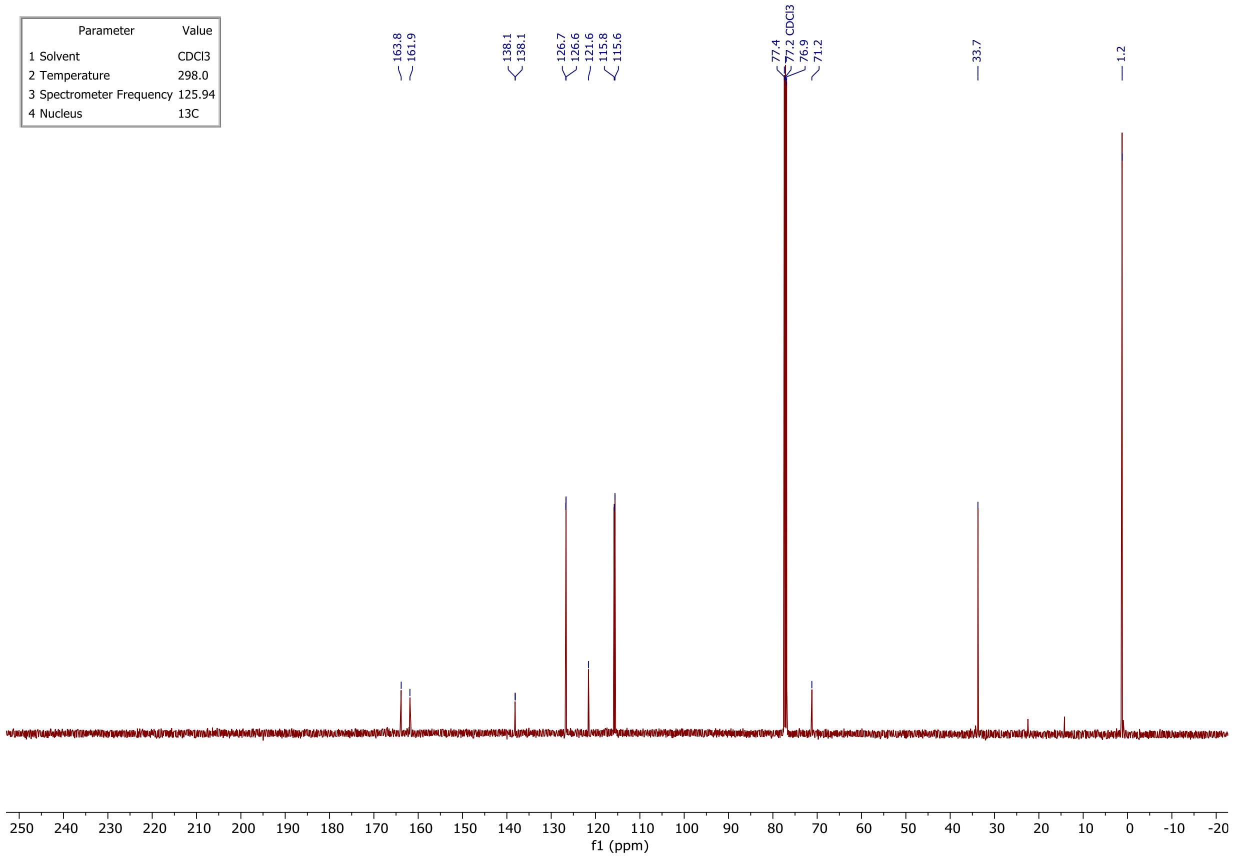

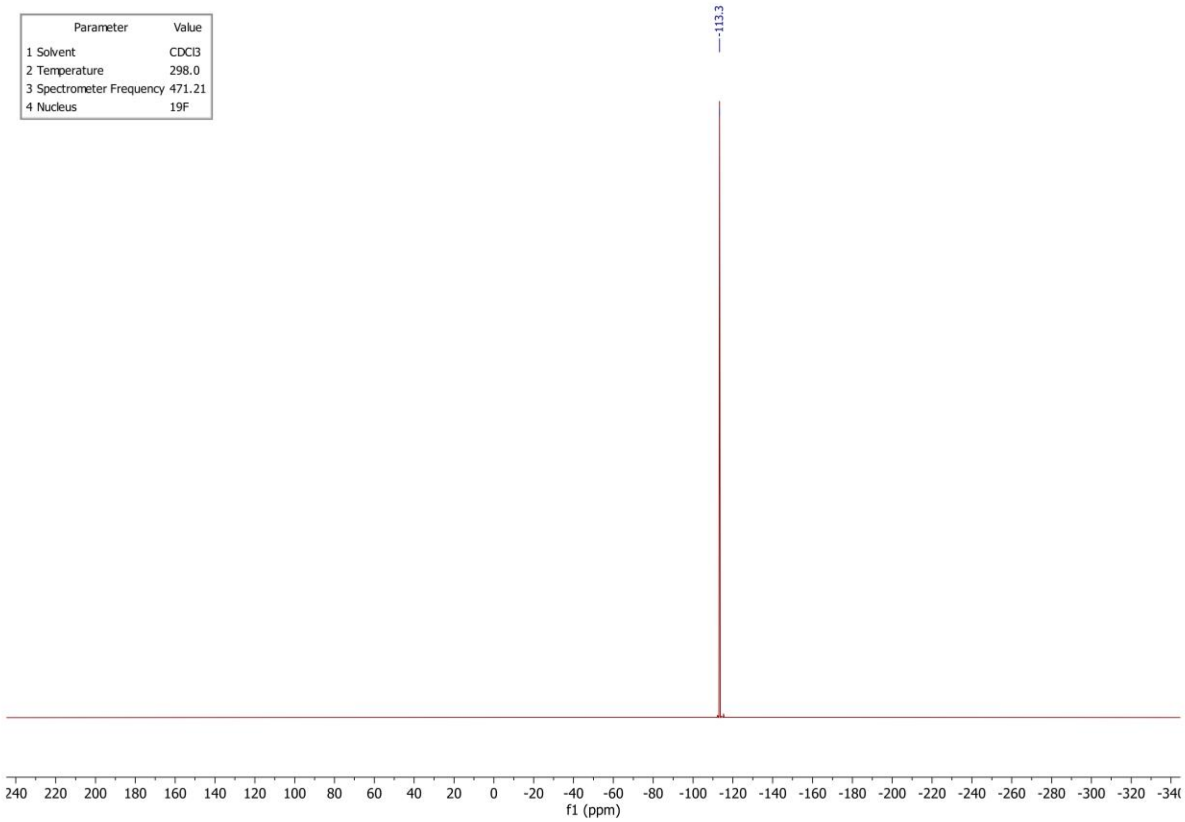

2-(4-chlorophenyl)-2-((trimethylsilyl)oxy)propanenitrile 33

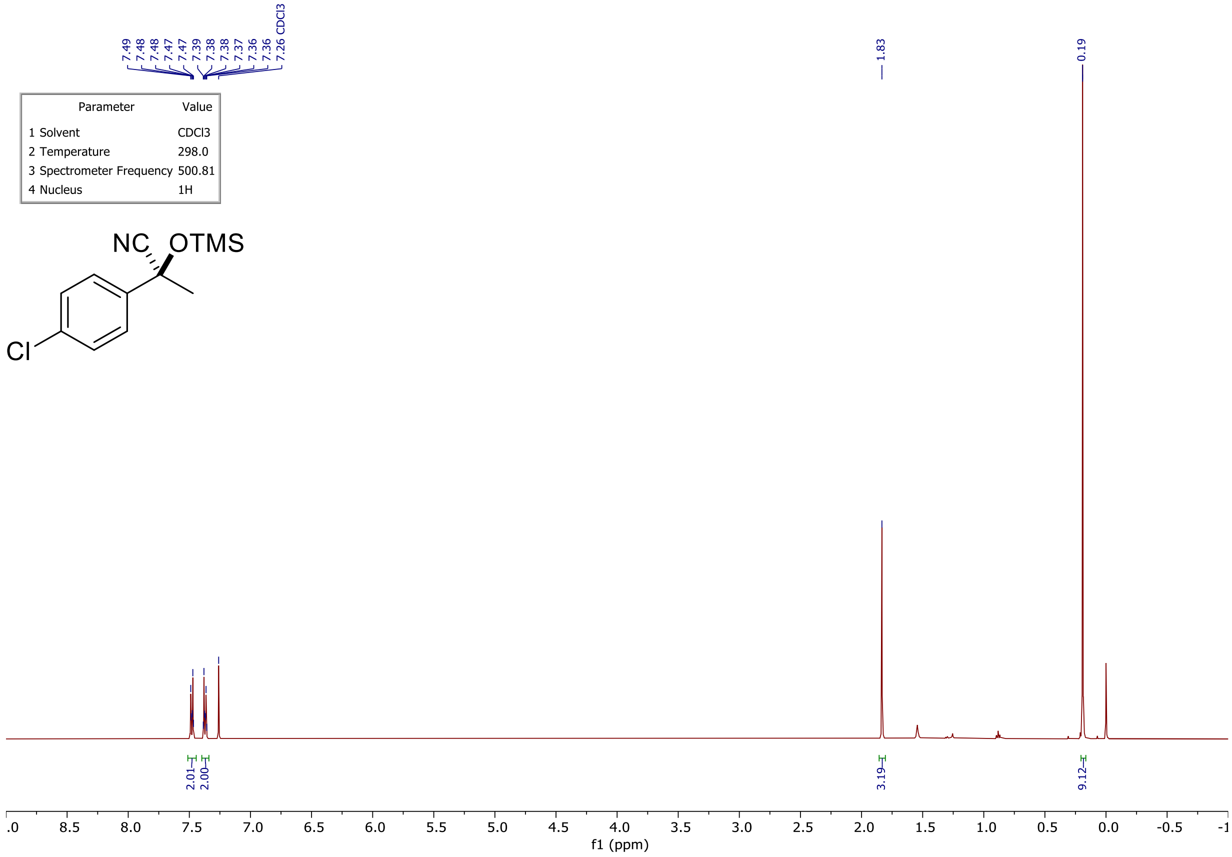

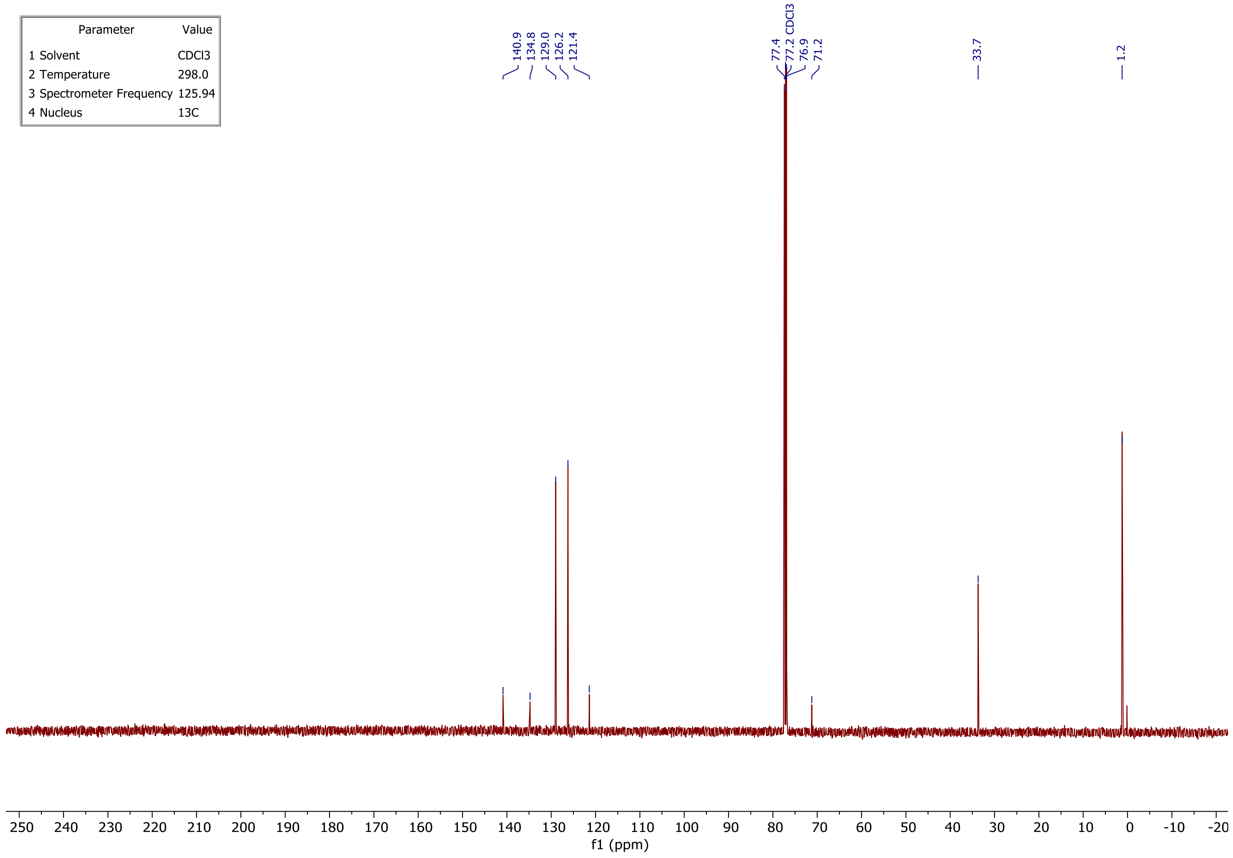

2-(4-bromophenyl)-2-((trimethylsilyl)oxy)propanenitrile 34

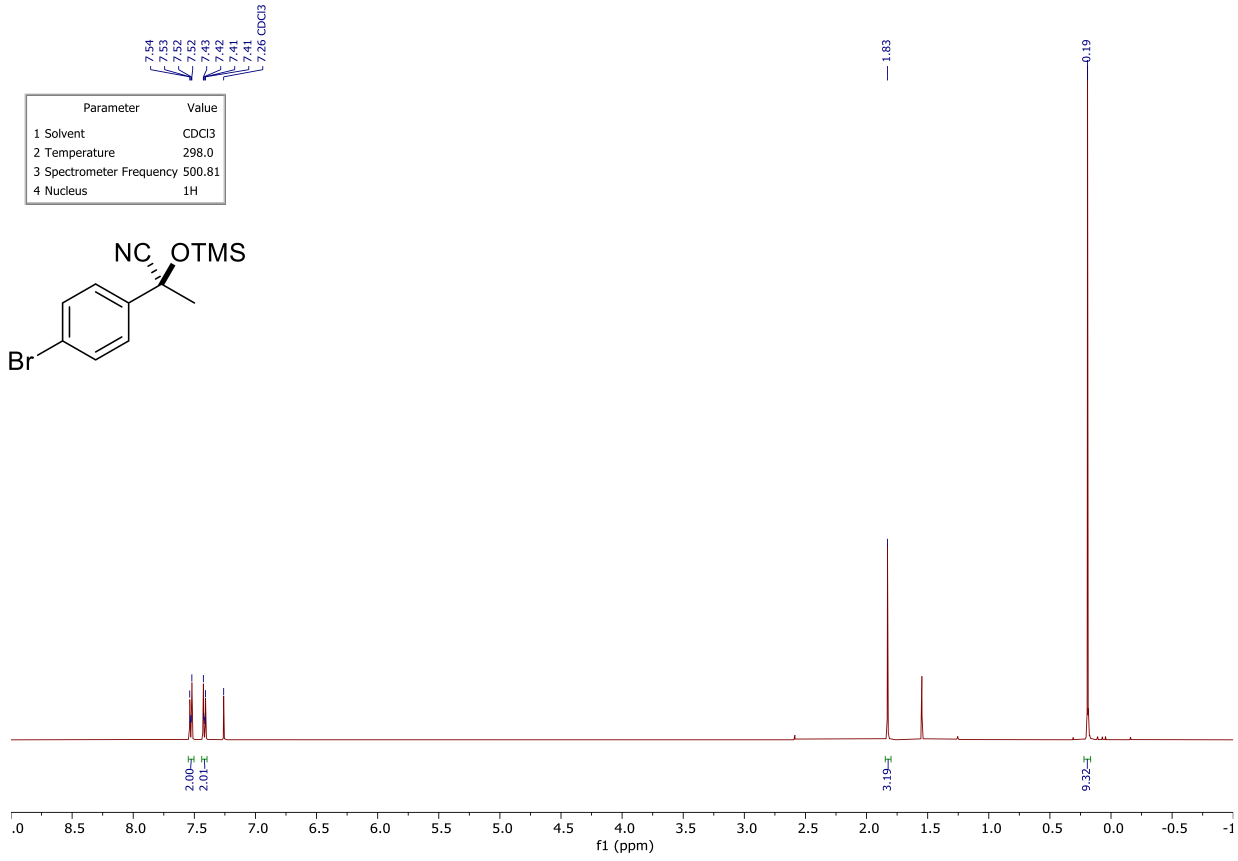

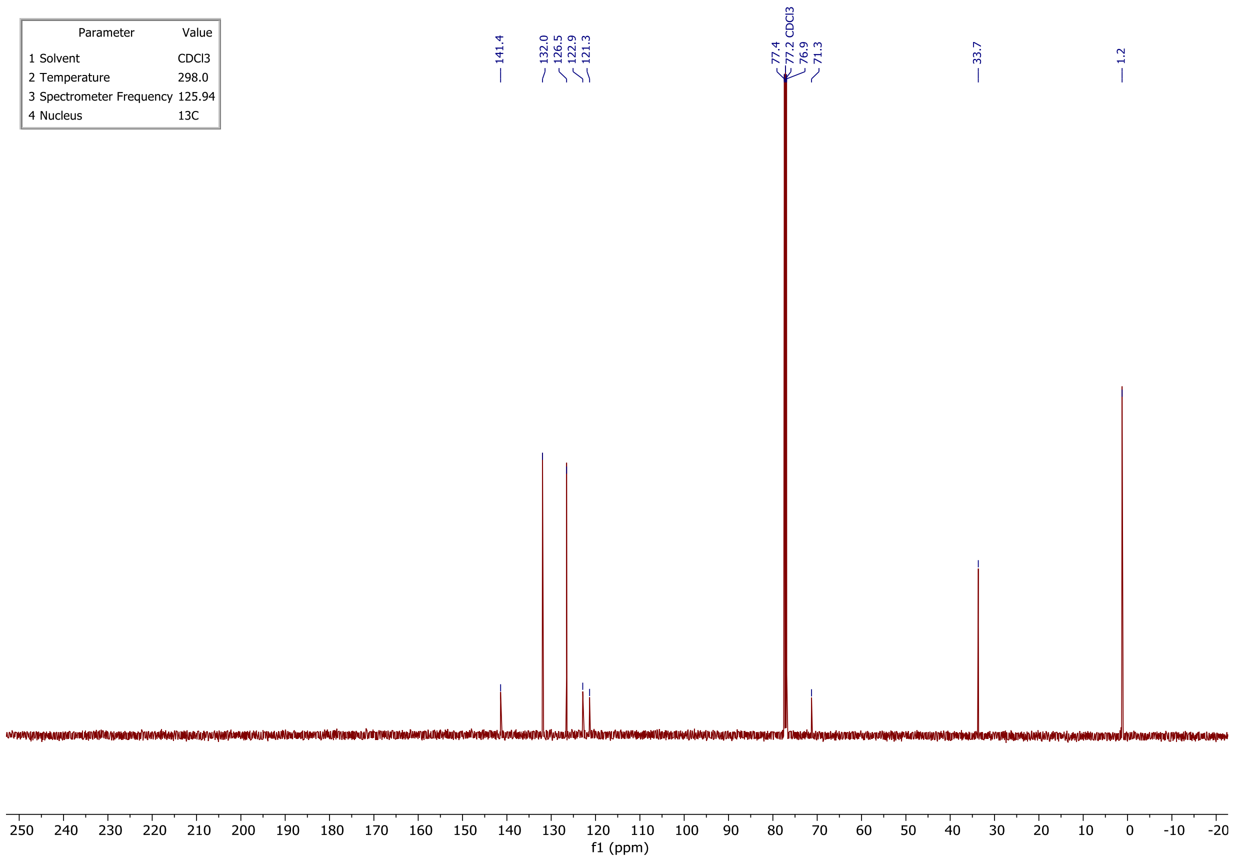

2-(m-tolyl)-2-((trimethylsilyl)oxy)propanenitrile 35

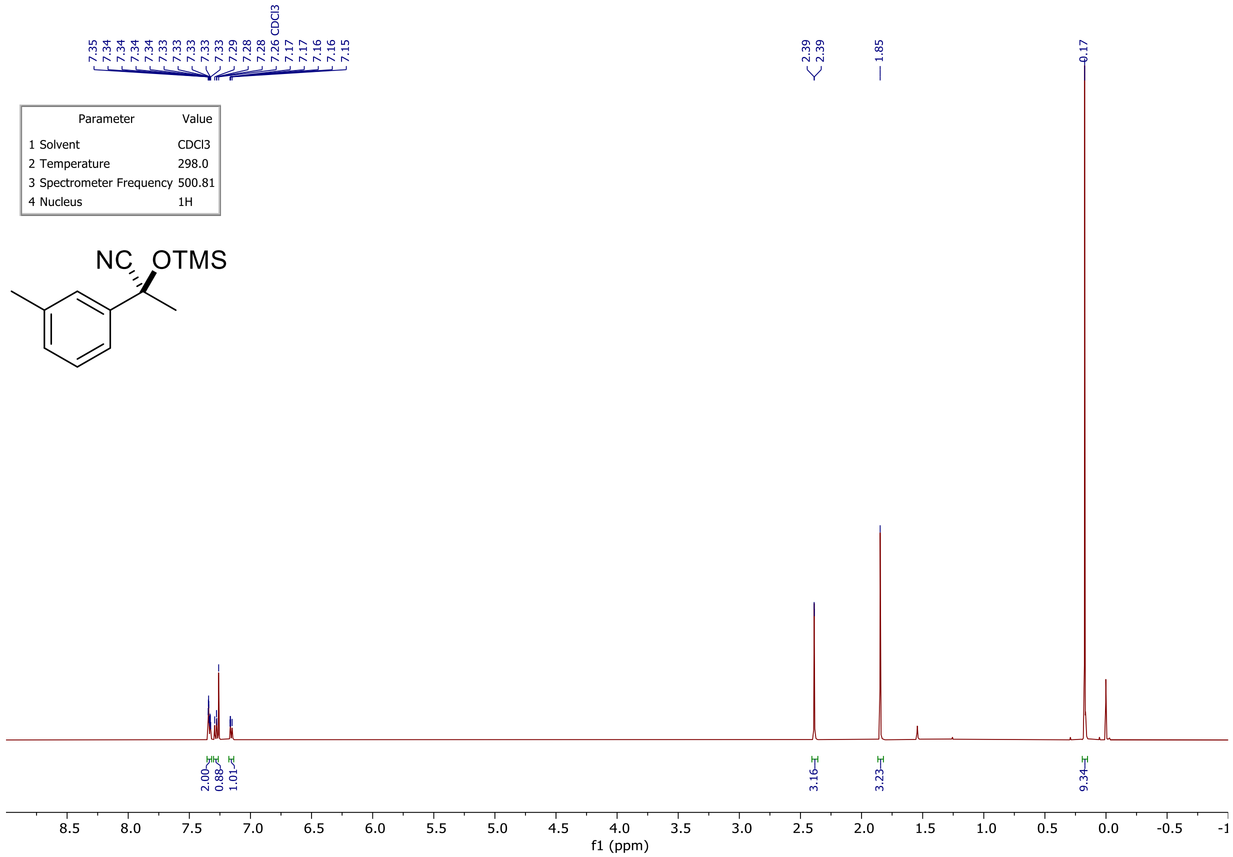

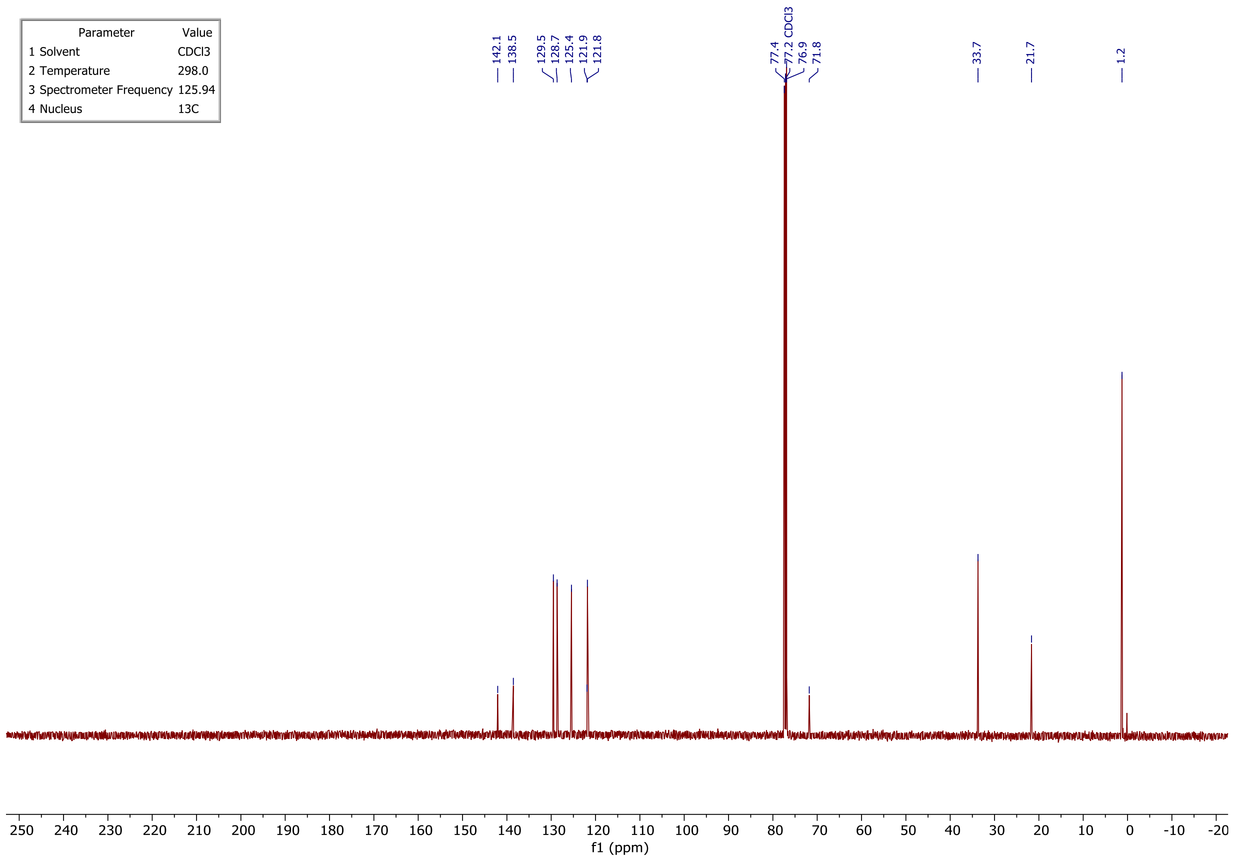

2-(3-methoxyphenyl)-2-((trimethylsilyl)oxy)propanenitrile 36

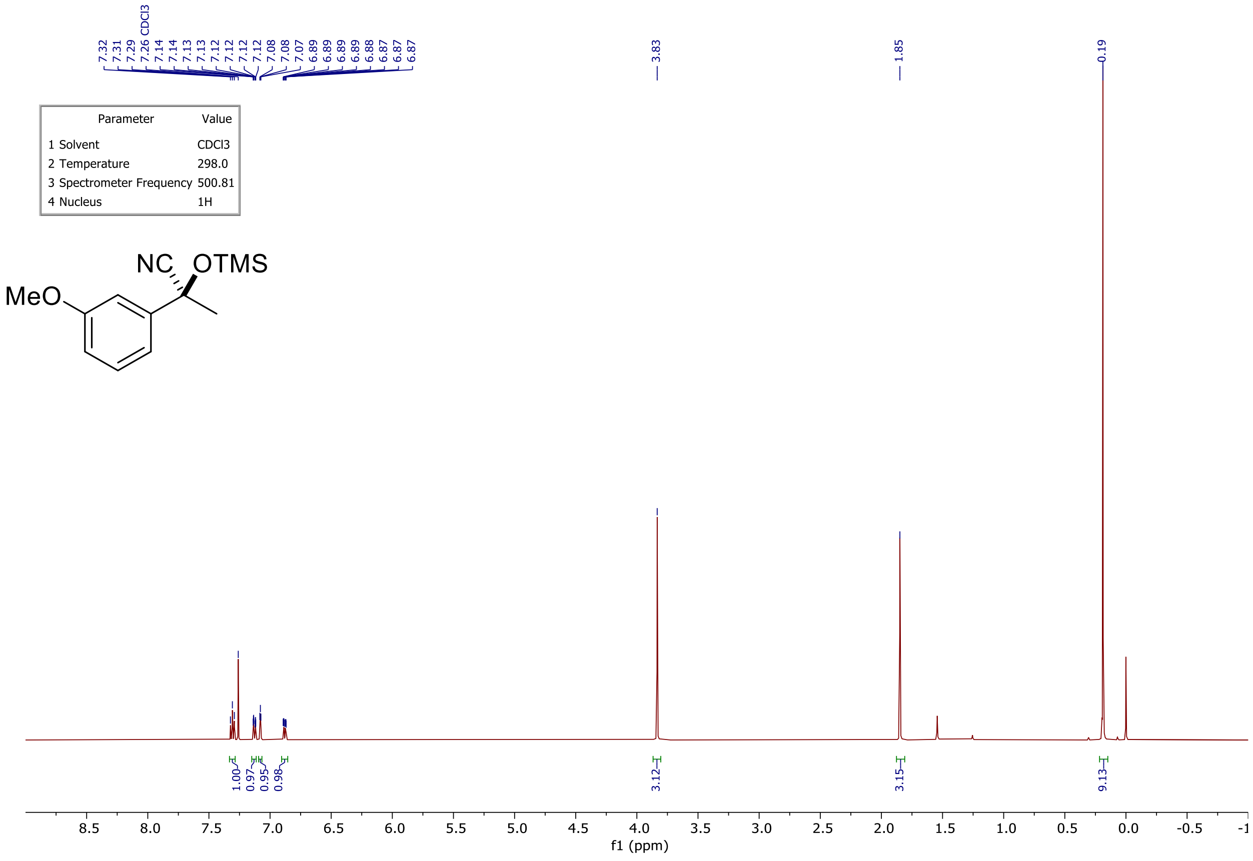

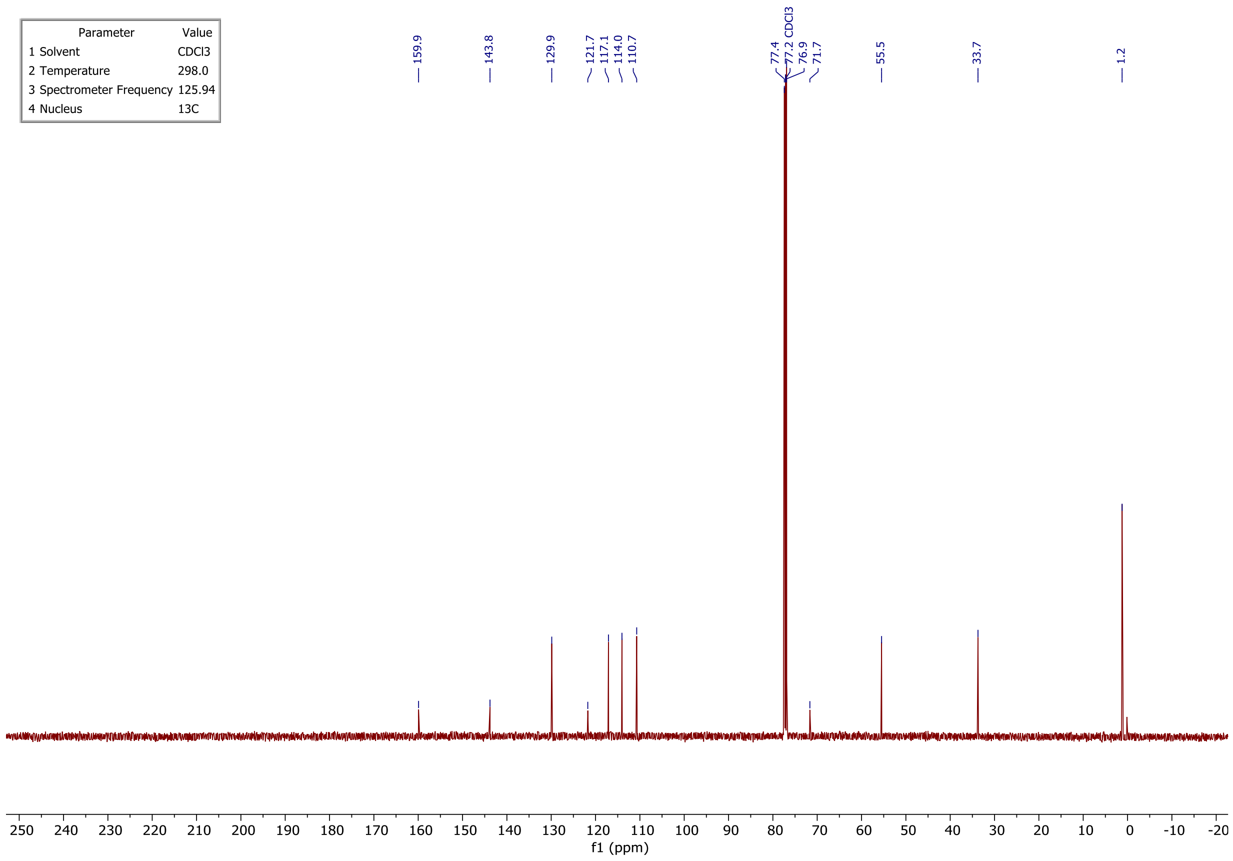

2-(3-chlorophenyl)-2-((trimethylsilyl)oxy)propanenitrile 37

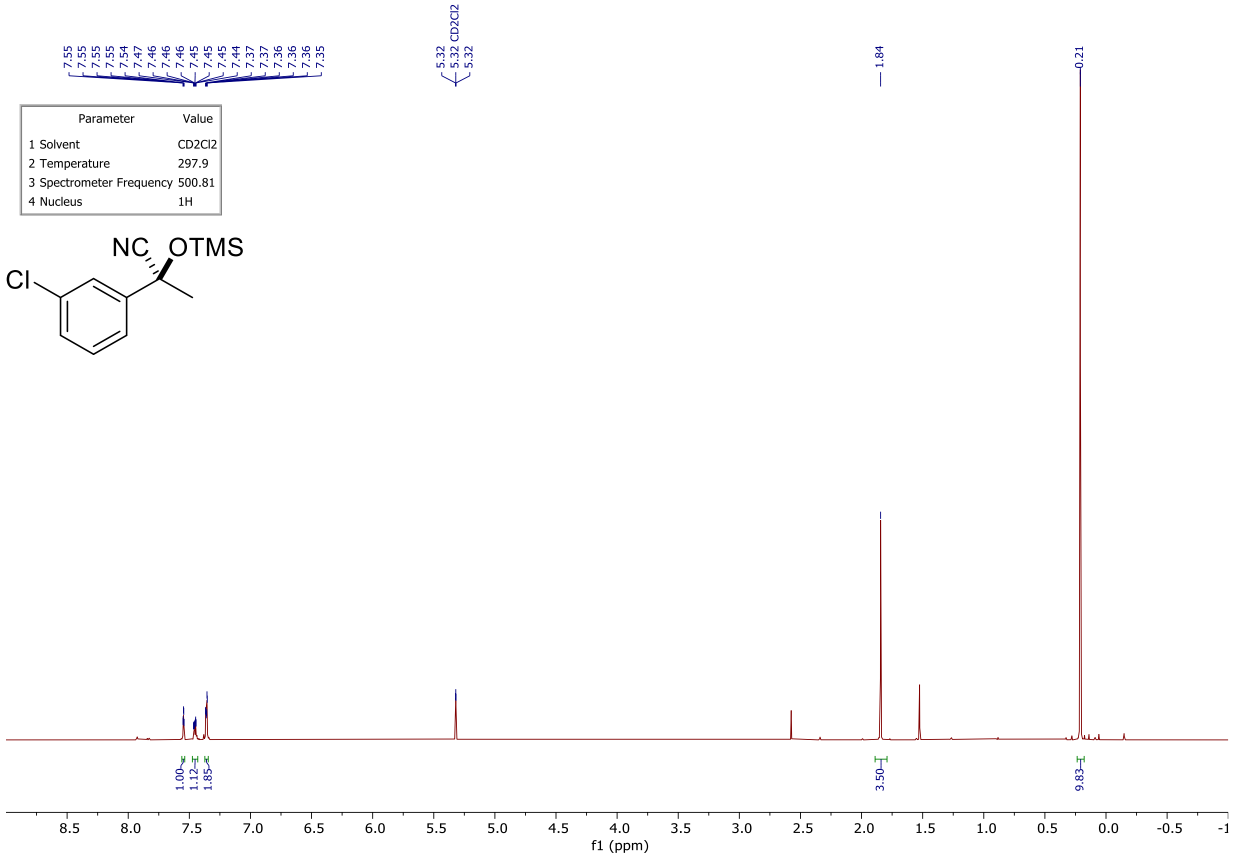

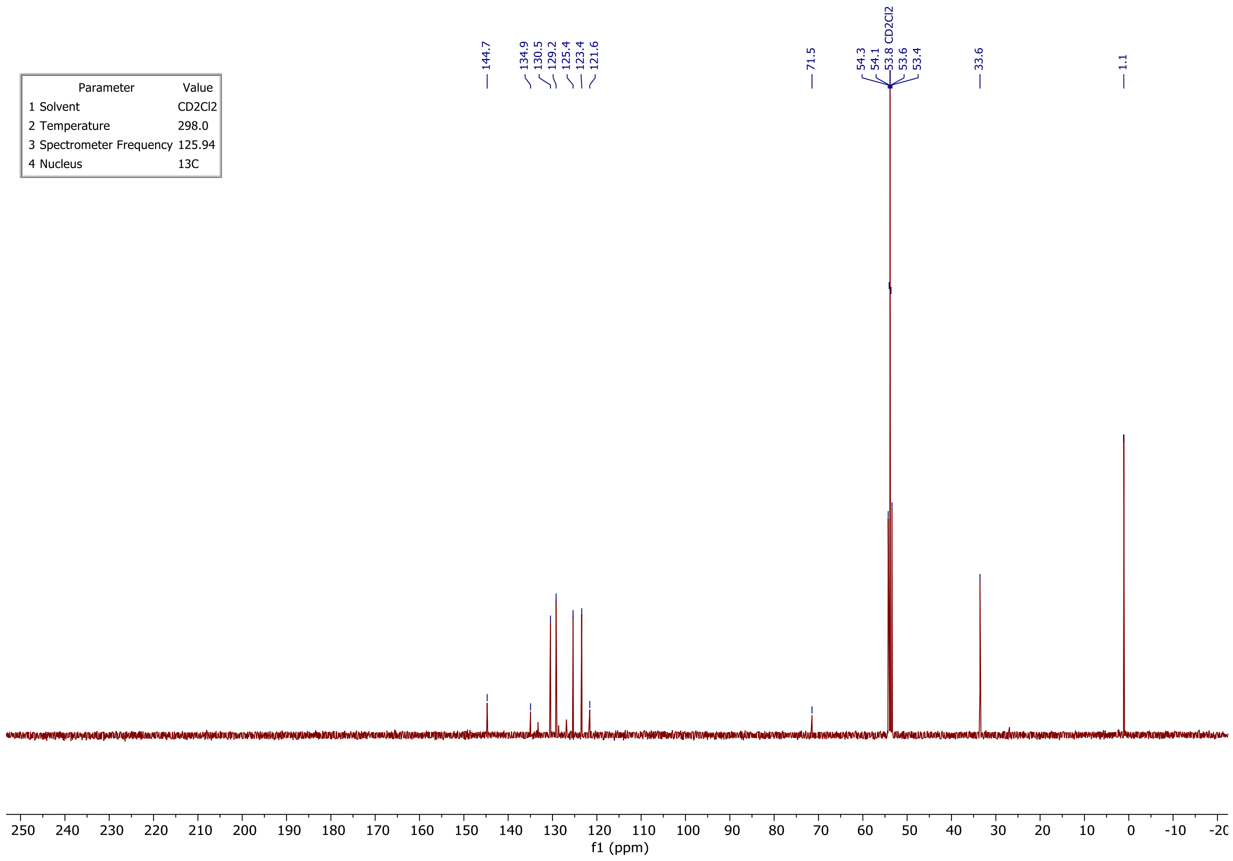

2-(3-(trifluoromethyl)phenyl)-2-((trimethylsilyl)oxy)propanenitrile 38

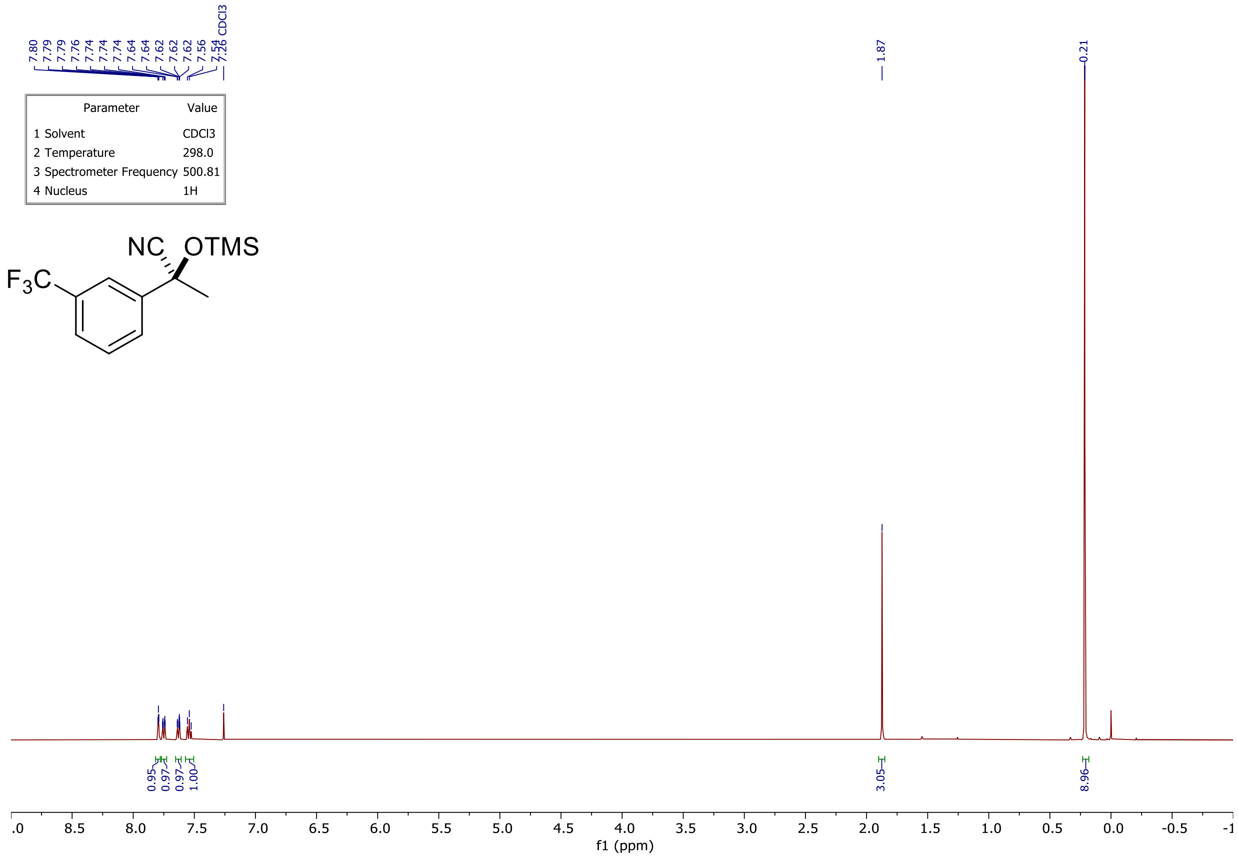

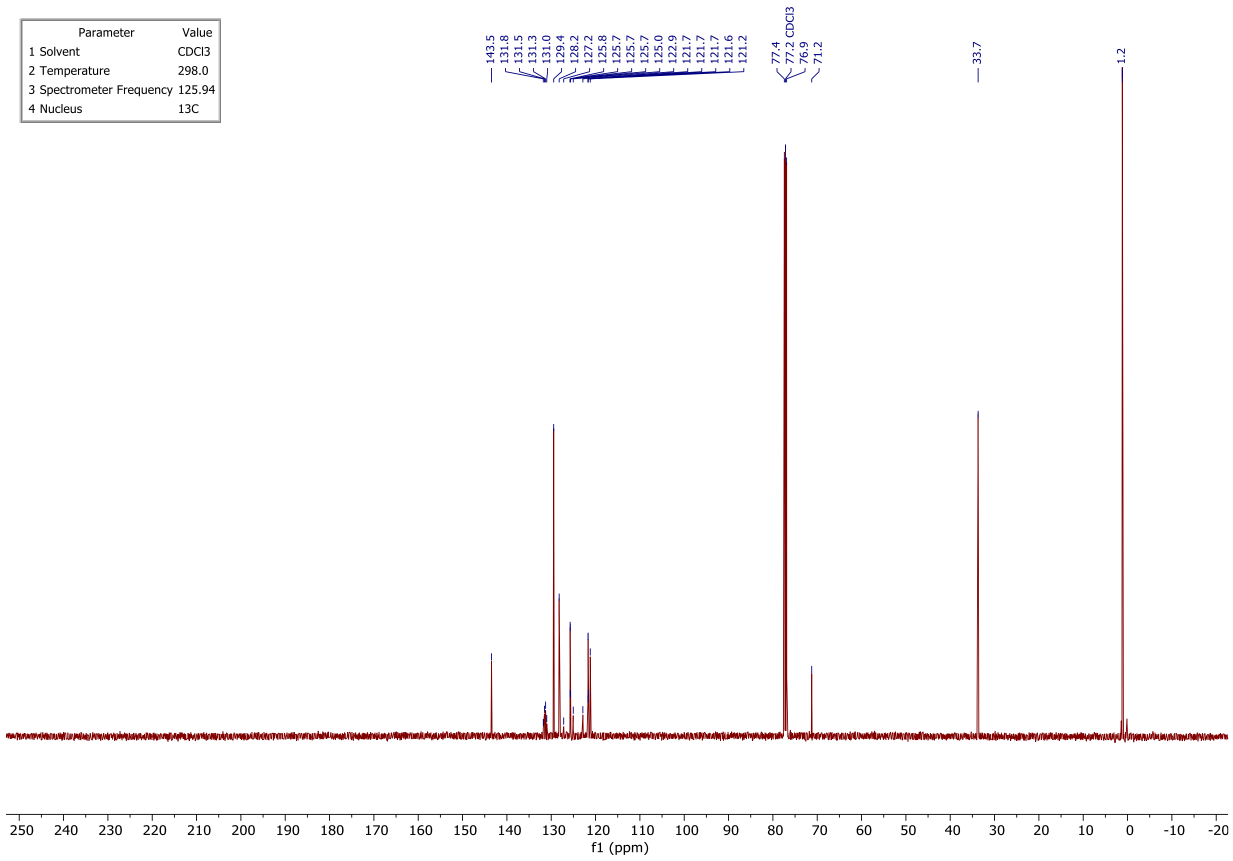
Parameter Value
1 Solvent CDCl3
2 Temperature 298.0
3 Spectrometer Frequency 125.94
4 Nucleus 13C
143.5
131.8
131.5
131.3
131.0
129.4
128.2
127.2
125.8
125.7
125.7
125.7
125.0
122.9
121.7
121.7
121.7
121.6
121.2
77.4
77.2 CDCl3
76.9
71.2
33.7
1.2
f1 (ppm)

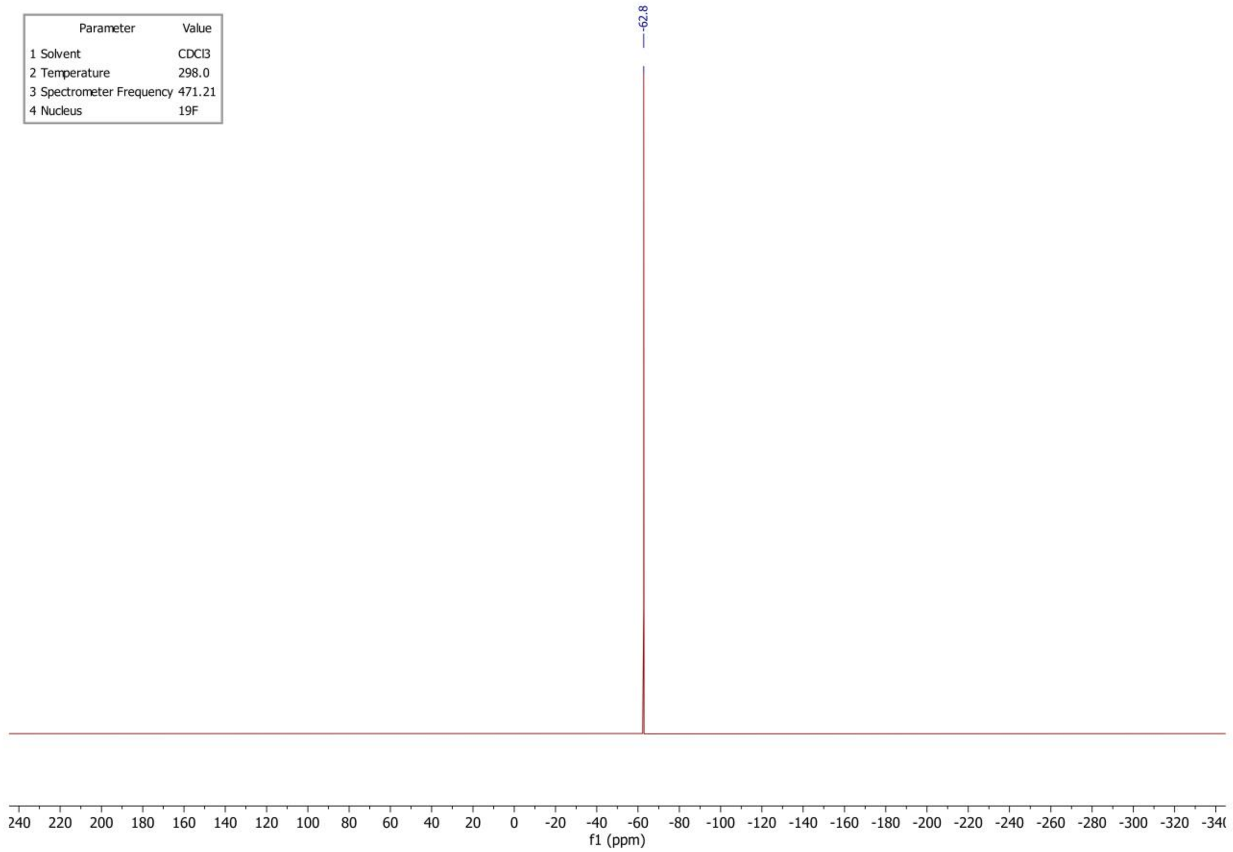
Parameter Value
1 Solvent CDCl3
2 Temperature 298.0
3 Spectrometer Frequency 471.21
4 Nucleus 19F
-62.8
f1 (ppm)

2-(2-chlorophenyl)-2-((trimethylsilyl)oxy)propanenitrile 39

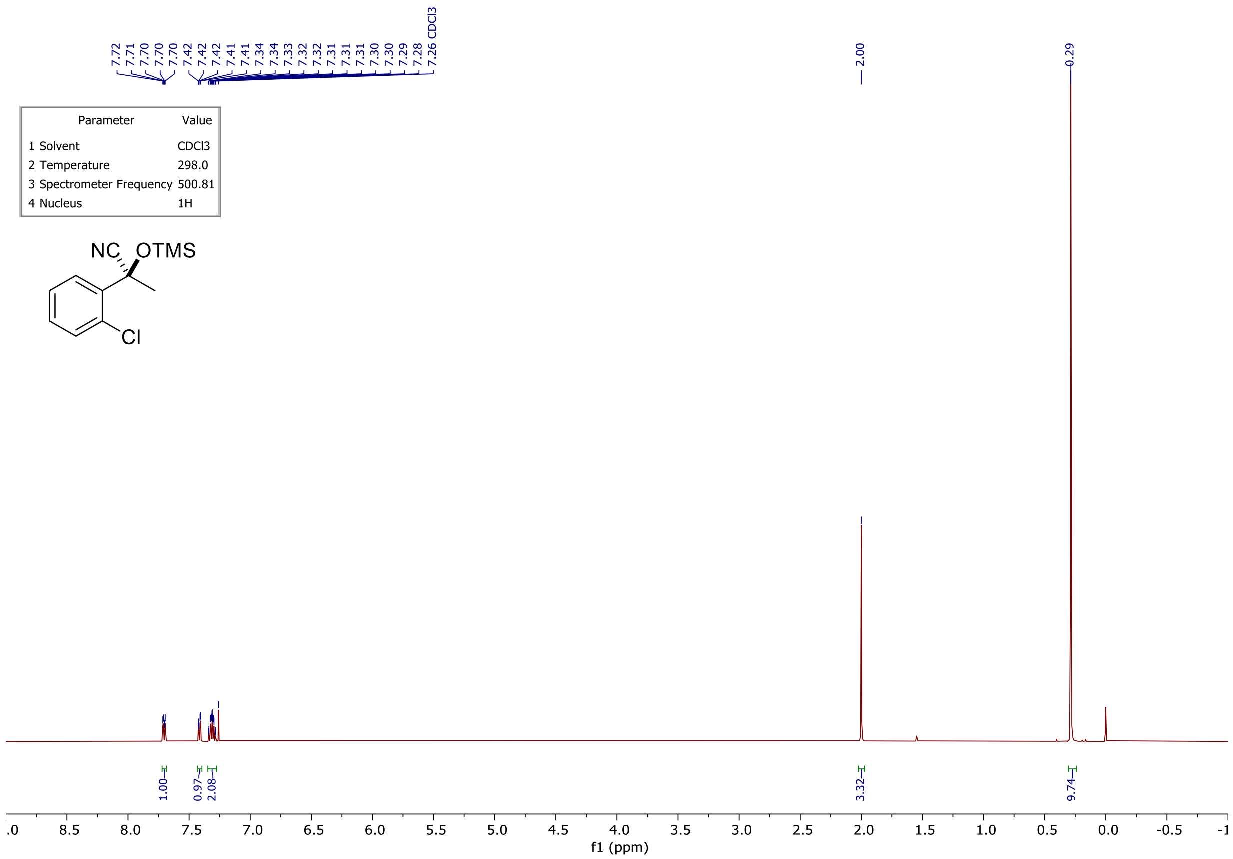

Parameter	Value
1 Solvent	CDCl3
2 Temperature	298.0
3 Spectrometer Frequency	500.81
4 Nucleus	1H

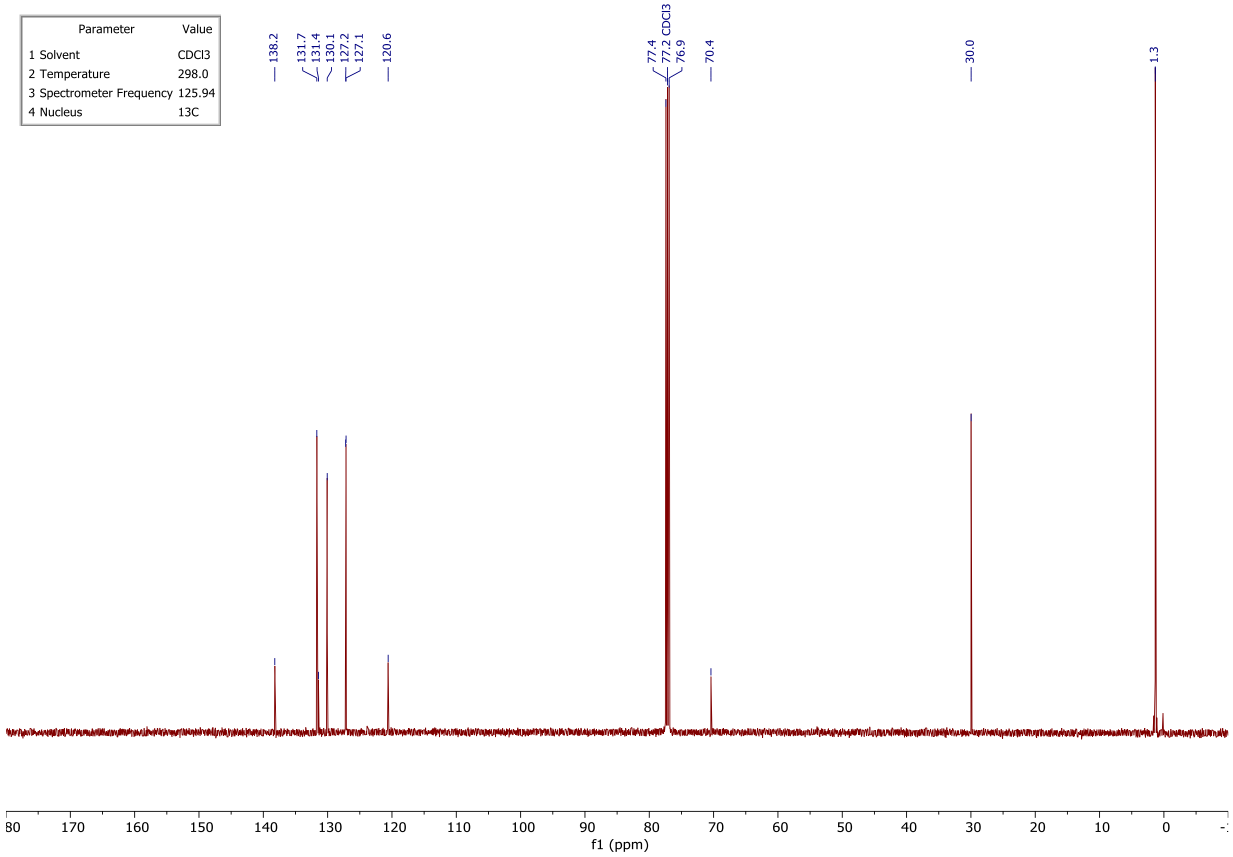

Parameter	Value
1 Solvent	CDCl3
2 Temperature	298.0
3 Spectrometer Frequency	125.94
4 Nucleus	13C

2-(3,5-dimethylphenyl)-2-((trimethylsilyl)oxy)propanenitrile 40

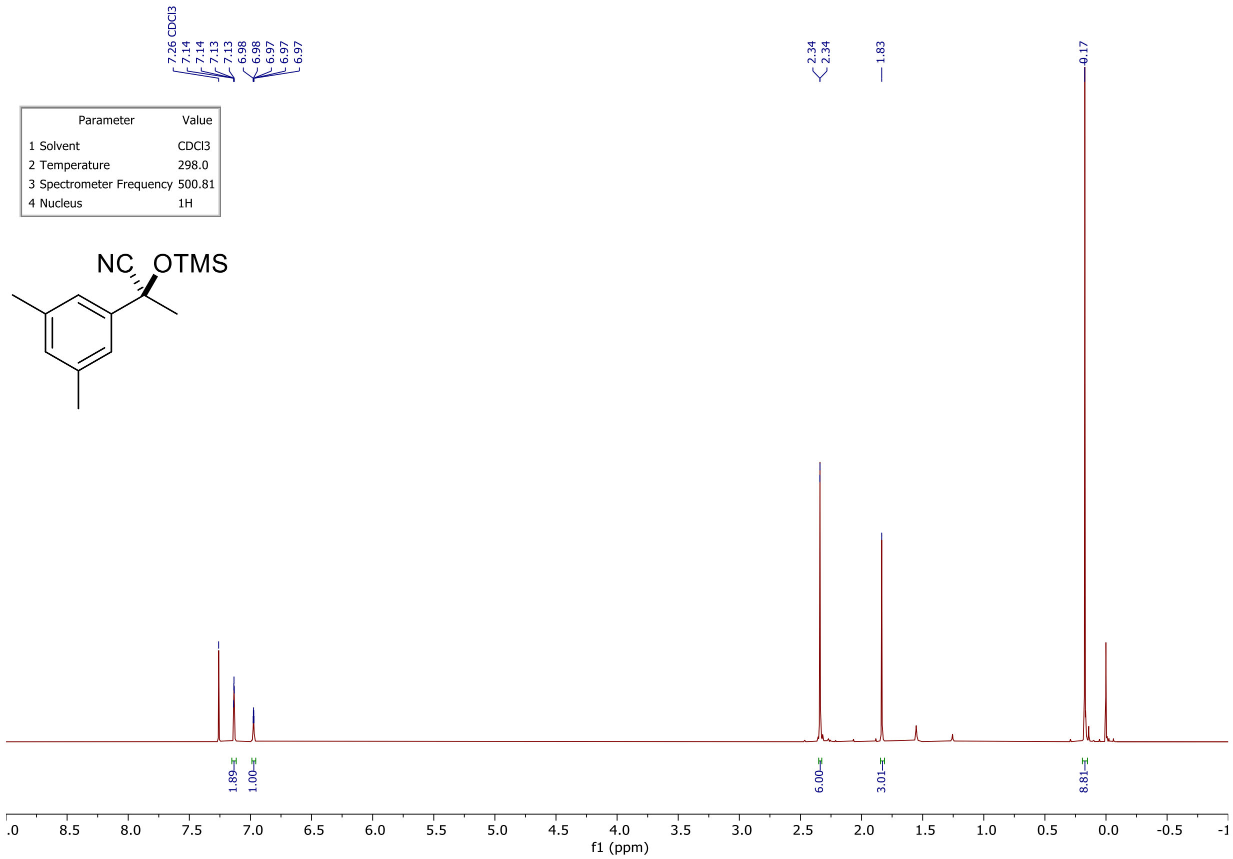

Parameter	Value
1 Solvent	CDCl3
2 Temperature	298.0
3 Spectrometer Frequency	500.81
4 Nucleus	1H

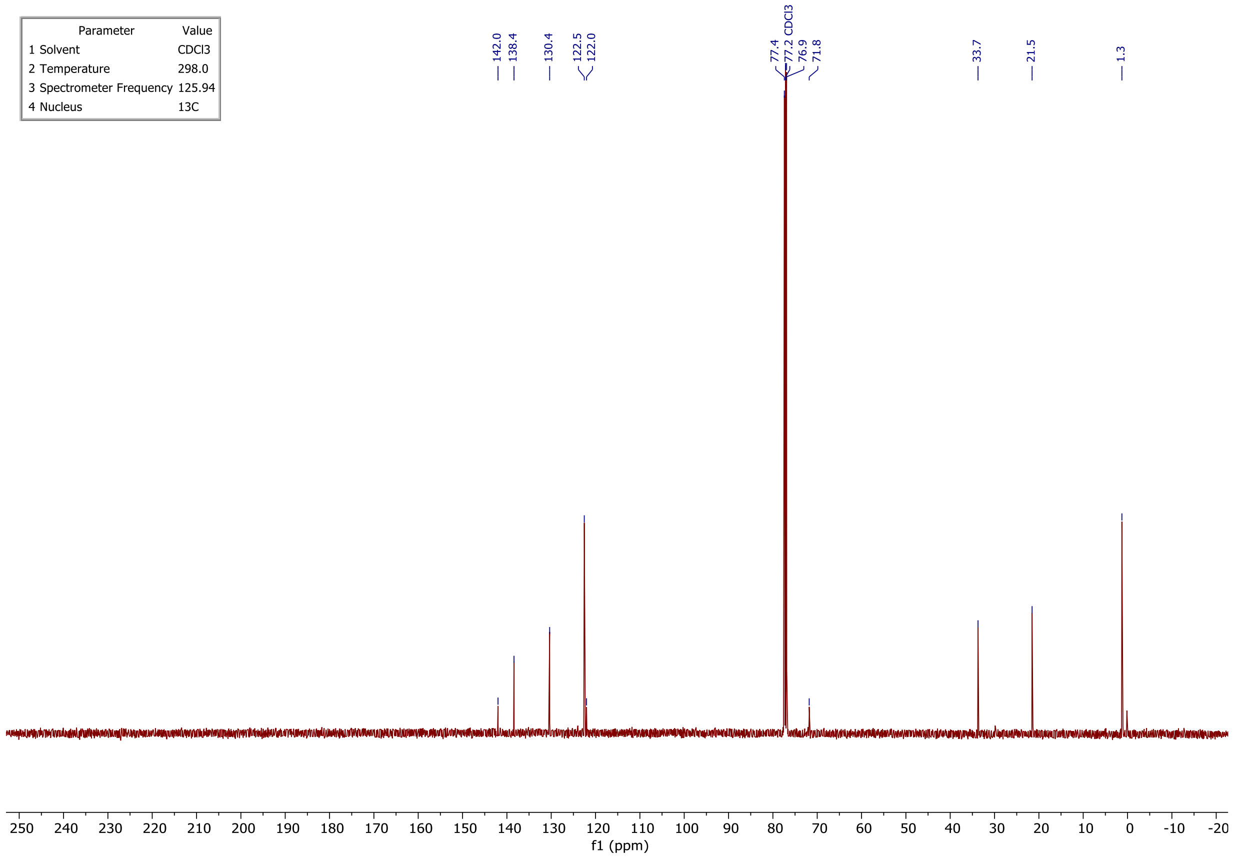

Parameter	Value
1 Solvent	CDCl3
2 Temperature	298.0
3 Spectrometer Frequency	125.94
4 Nucleus	13C

2-(naphthalen-2-yl)-2-((trimethylsilyl)oxy)propanenitrile 41

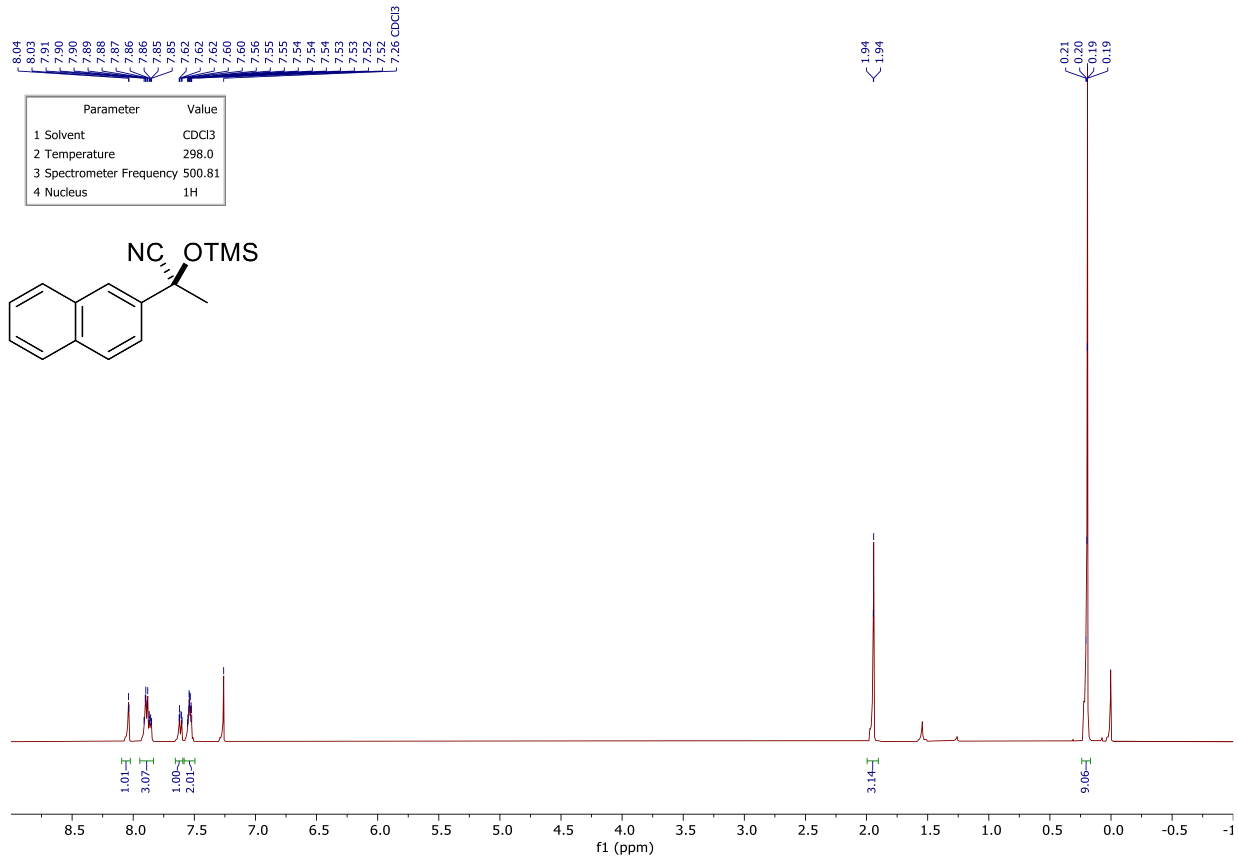

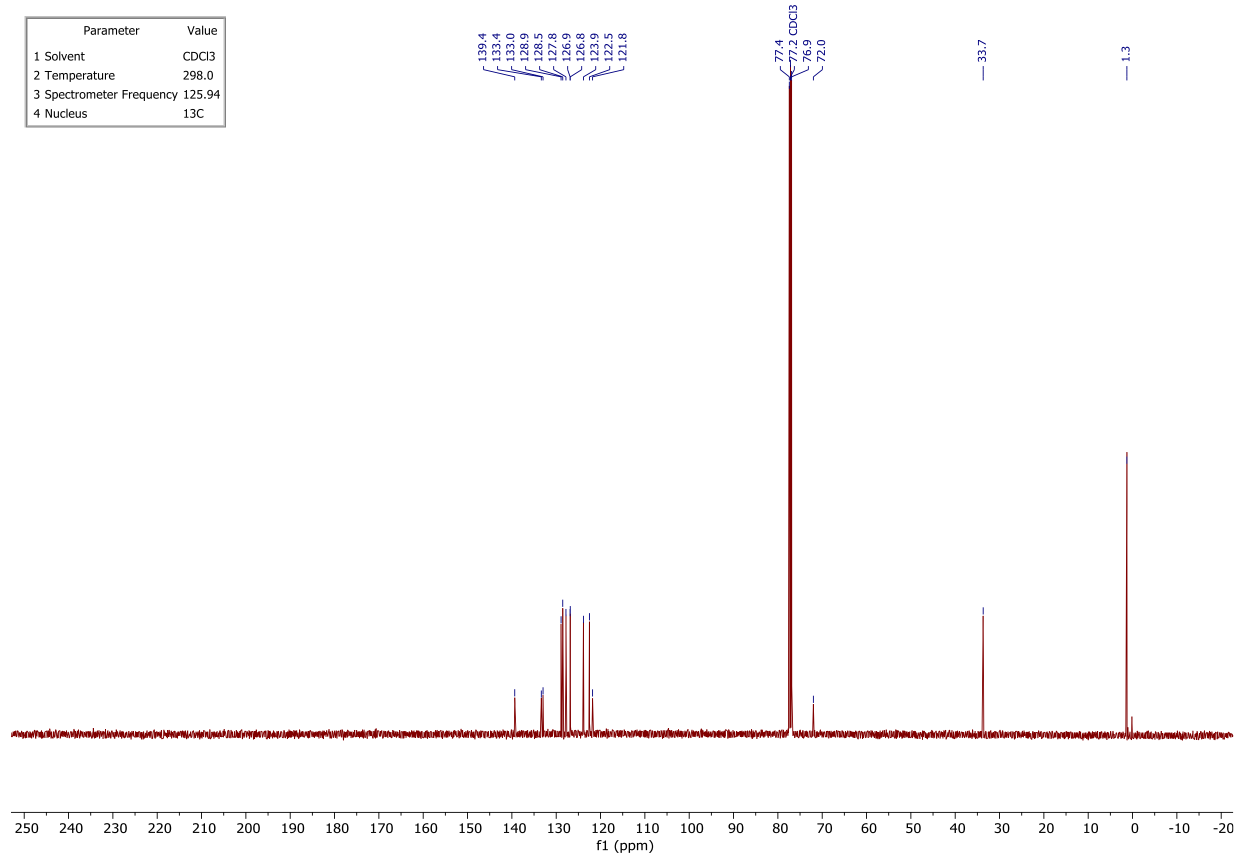

2-methyl-3-(thiophen-2-yl)-2-((trimethylsilyl)oxy)propanenitrile 42

Parameter	Value
1 Solvent	CD2Cl2
2 Temperature	298.0
3 Spectrometer Frequency	500.81
4 Nucleus	1H

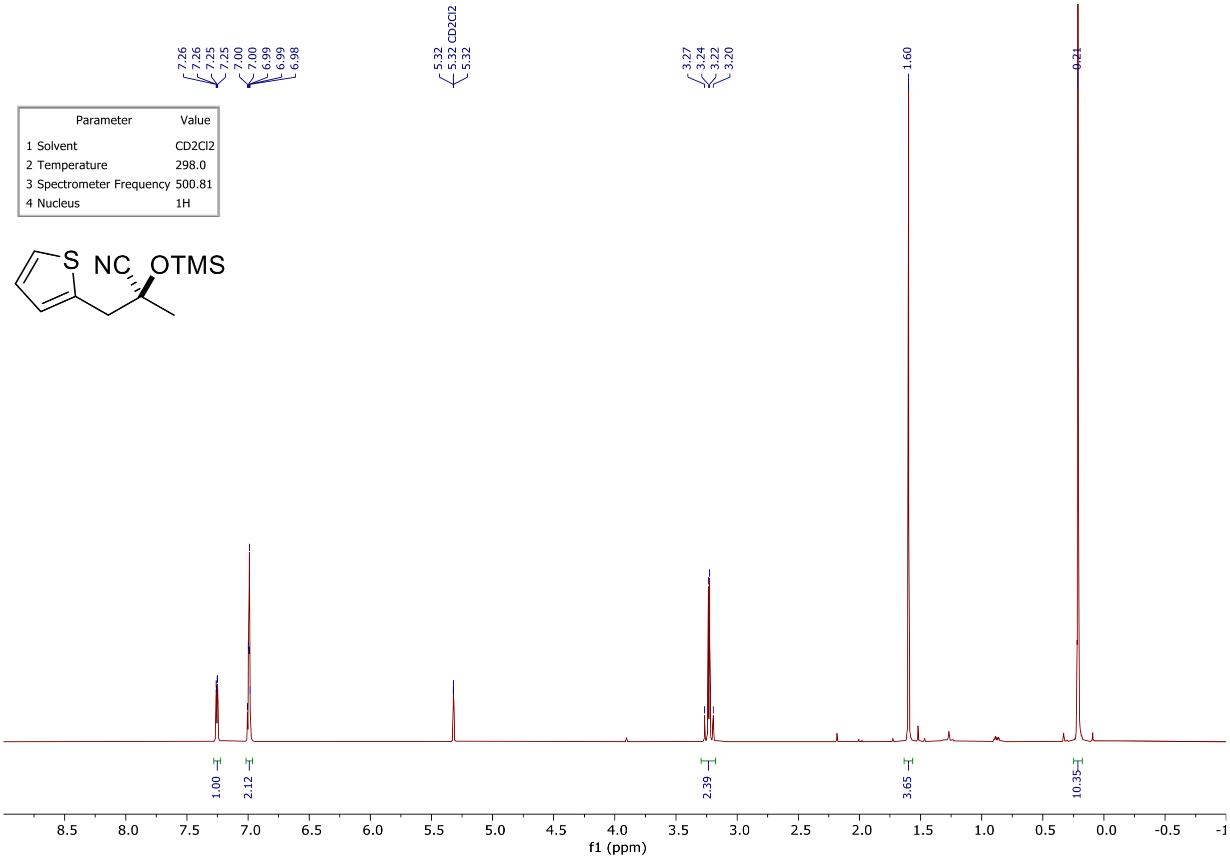

Parameter	Value
1 Solvent	CD2Cl2
2 Temperature	298.0
3 Spectrometer Frequency	125.94
4 Nucleus	13C

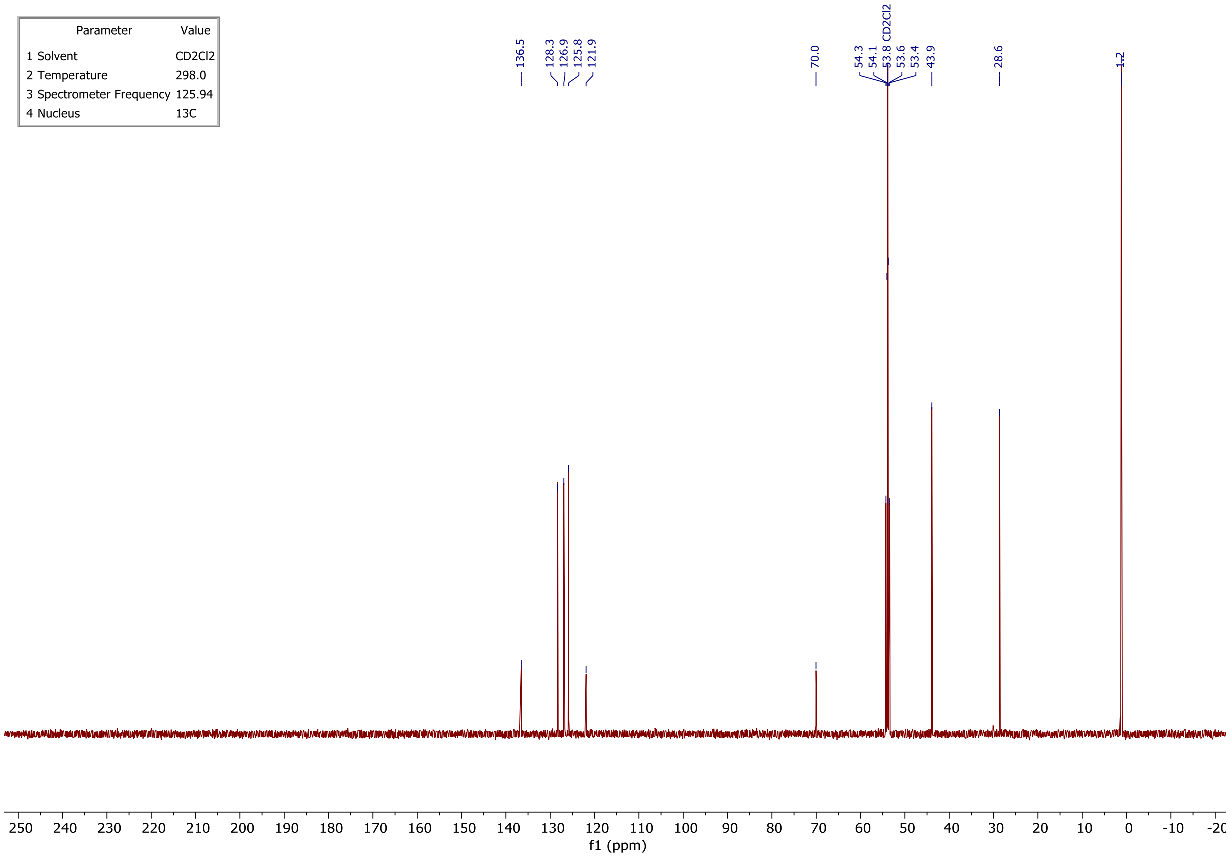

3-(furan-2-yl)-2-methyl-2-((trimethylsilyl)oxy)propanenitrile 43

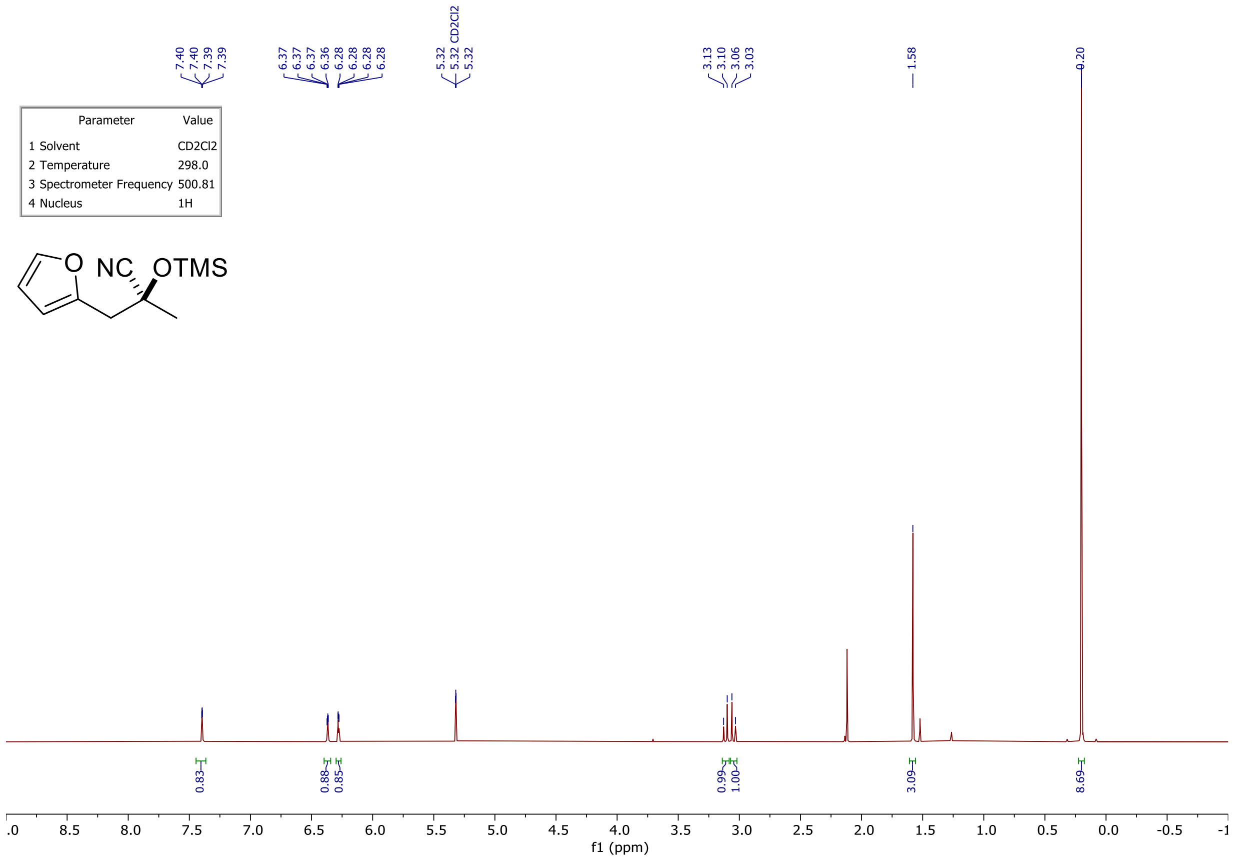

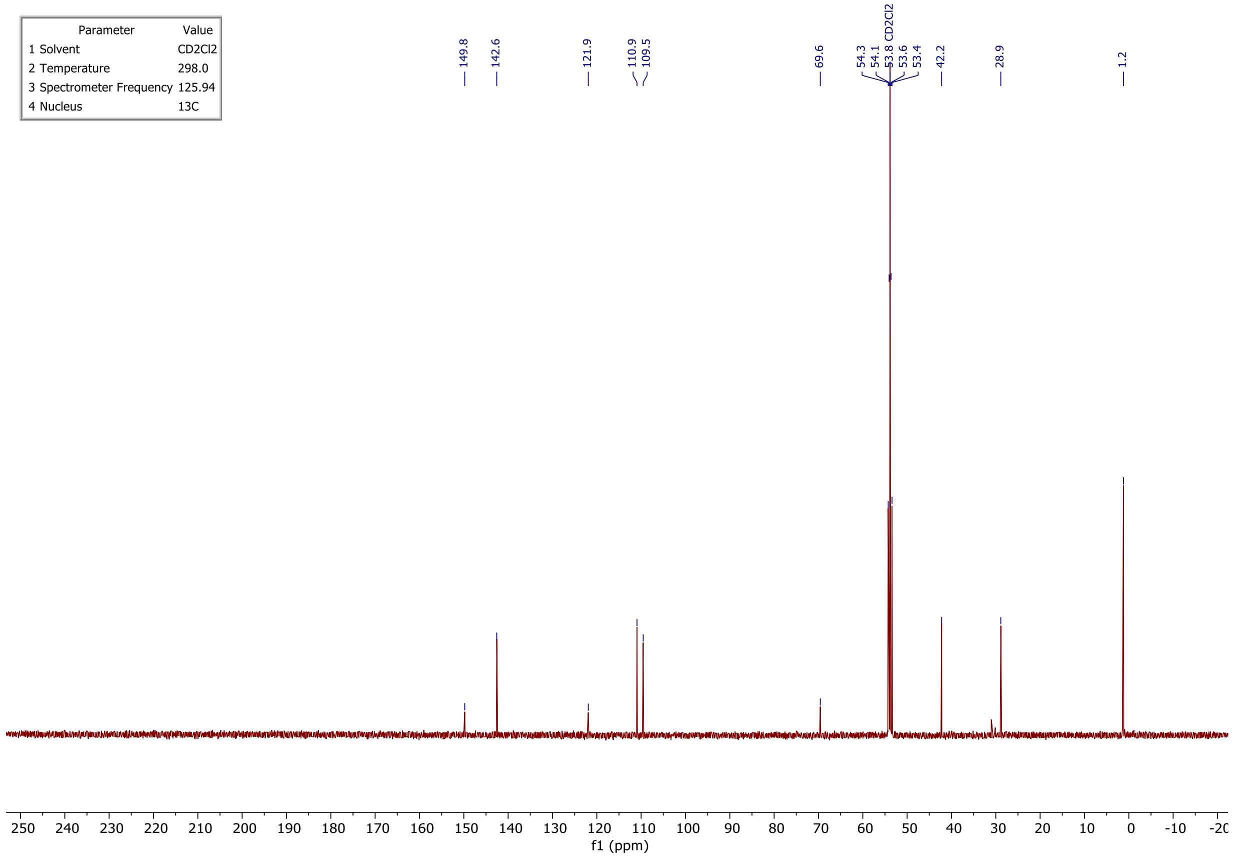

2-methyl-4-(thiophen-2-yl)-2-((trimethylsilyl)oxy)butanenitrile 44

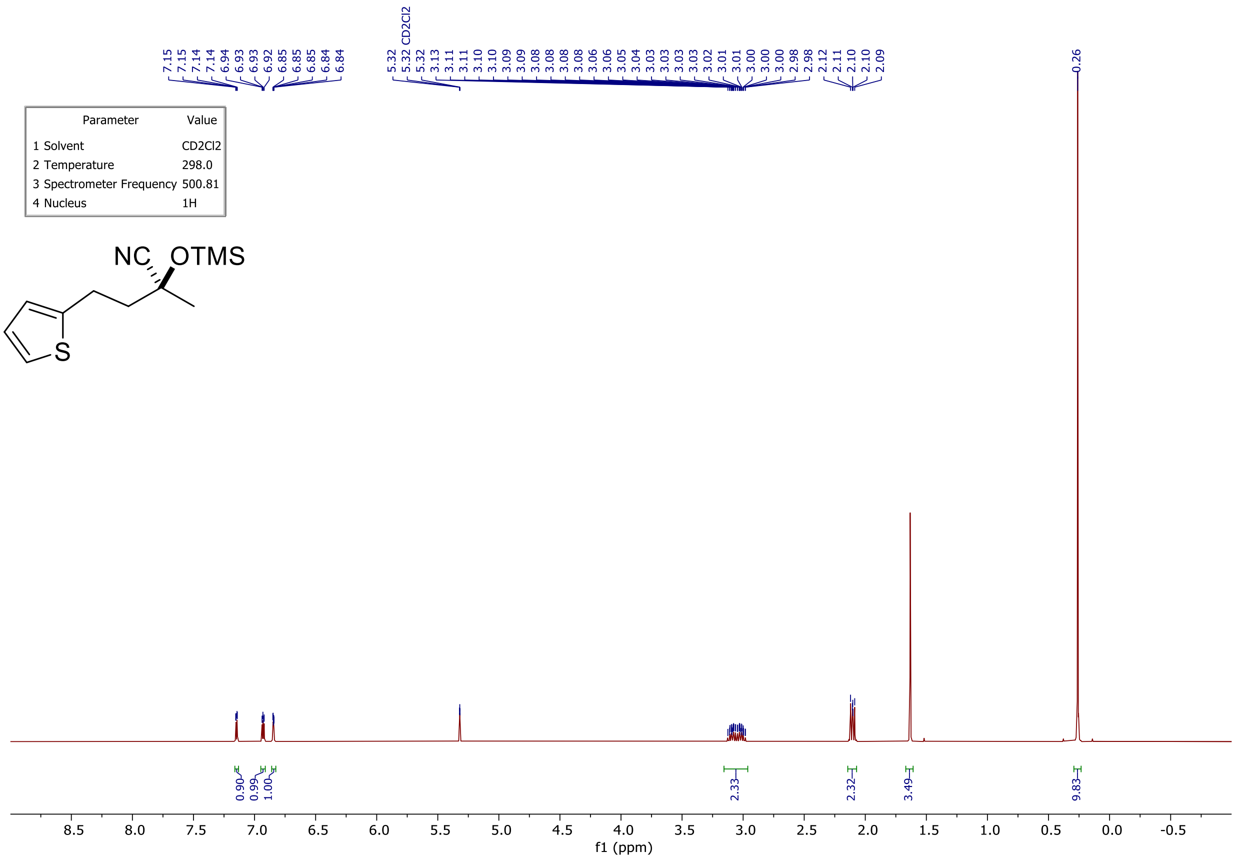

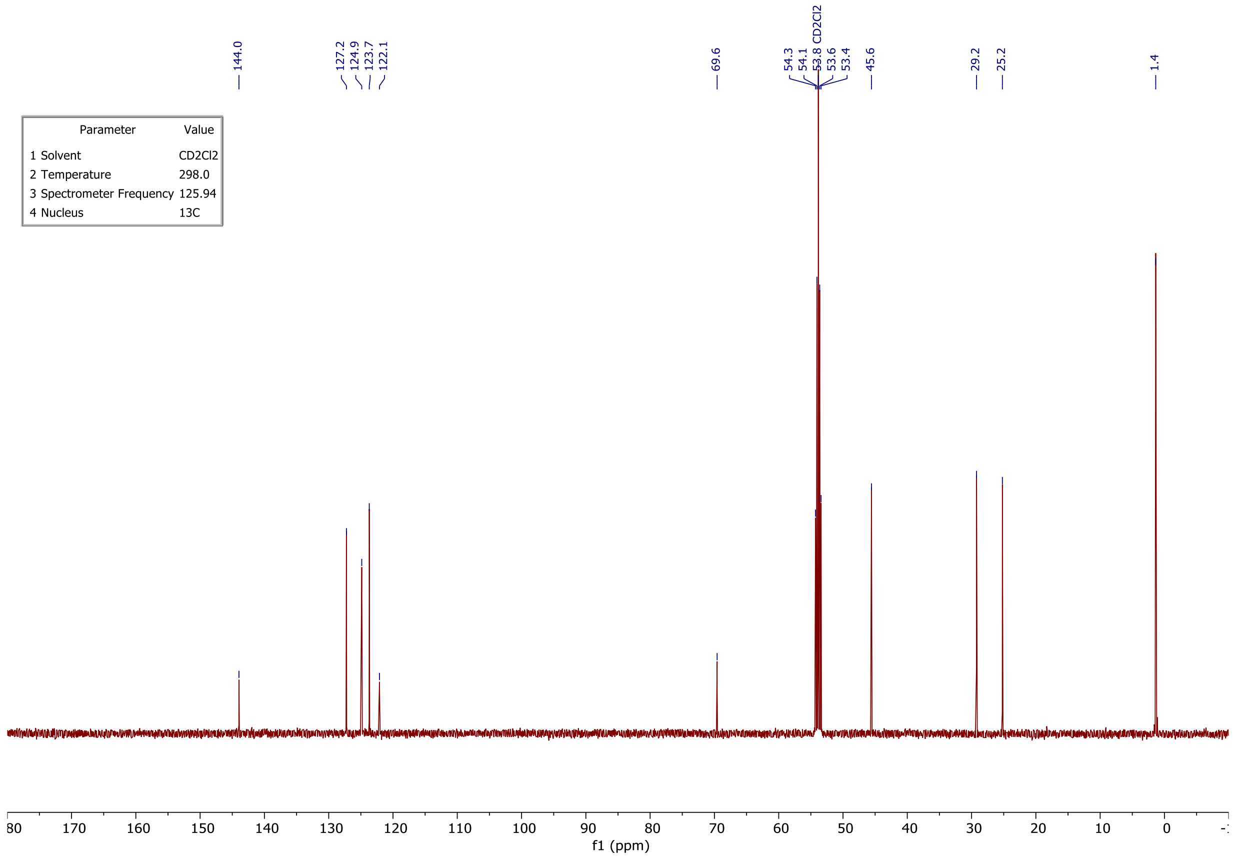

(3*S*,5*S*,8*R*,9*S*,10*S*,13*S*,14*S*,17*S*)-10,13-dimethyl-3,17-bis((trimethylsilyl)oxy)hexadecahydro-1*H*-cyclopenta[*a*]phenanthrene-3-carbonitrile 45

User Report
ZHH-ZC-201-02

NMR data supports the following structure:

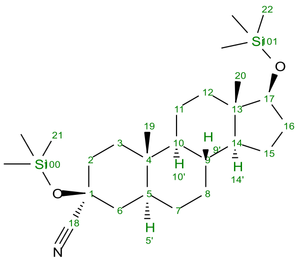

Atom	J	δ (ppm)	COSY	HSQC	HMBC	NOESY
1 C		72.669			2', 3', 3", 6'	
2 C		36.036		2', 2"	3', 6'	
H'		1.722	2", 3', 3"	2	1, 3, 6, 18	2", 19
H"		2.005	2', 3', 3"	2	18	2'
3 C		36.176		3', 3"	2', 19	
H'		1.248	2', 2", 3", 19	3	1, 2, 4, 19	3"
H"		1.755	2', 2", 3'	3	1, 19	3'
4 C		35.810			3', 6', 10', 19	
5 C		44.056		5'	6', 6", 19	
5' H	13.10(6')	1.411	6', 6", 7', 7"	5		10'
6 C		42.021		6', 6"	2'	
H'	13.10(6"), 13.10(5')	1.590	5', 6"	6	1, 2, 4, 5, 18	6", 19
H"	13.10(6')	1.755	5', 6'	6	5, 7, 18	6'
7 C		28.369		7', 7"	6", 8', 8"	
H'		1.294	5'	7	8	
H"		1.258	5'	7	8	
8 C		31.743		8', 8"	7', 7", 9', 10', 14'	
H'		0.920	9'	8	7, 14	8"
H"		1.685	9'	8	7, 14	8'
9 C		35.922		9'	10', 15'	
9' H		1.392	8', 8", 10', 14'	9	8, 10	19, 20
10 C		54.520		10'	9', 11', 11", 12', 12", 19	
10' H		0.740	9', 11', 11"	10	4, 8, 9, 11, 14, 19	14',5'
11 C		21.222		11', 11"	10', 12', 12"	
H'		1.288	10', 11", 12', 12"	11	10, 12	11", 19, 20
H"		1.543	10', 11', 12', 12"	11	10, 12	11'

Atom	J	δ (ppm)	COSY	HSQC	HMBC	NOESY
12 C		37.383		12', 12"	11', 11", 14', 17, 20	
H'		0.987	11', 11", 12", 20	12	10, 11, 13, 14, 17, 20	12", 17
H"		1.727	11', 11", 12'	12	10, 11, 13, 14, 17	12'
13 C		43.443			12', 12", 14', 16', 17, 20	
14 C		50.988		14'	8', 8", 10', 12', 12", 15', 15", 16', 16", 20	
14' H		0.930	9', 15', 15"	14	8, 12, 13, 15, 17, 20	10', 17
15 C		23.820		15', 15"	14', 16', 16"	
H'		1.224	14', 15", 16', 16"	15	9, 14, 16, 17	15", 16"
H"		1.550	14', 15', 16', 16"	15	14, 16, 17	15'
16 C		31.229		16', 16"	15', 15", 17	
H'		1.875	15', 15", 16", 17	16	13, 14, 15, 17	16", 17
H"		1.409	15', 15", 16', 17	16	14, 15, 17	15', 16'
17 C		82.098		17	12', 12", 14', 15', 15", 16', 16", 20	
H		3.553	16', 16"	17	12, 13, 16, 20,101	12', 14', 16'
18 C		122.555			2', 2", 6', 6"	
19 C		12.354		19	3', 3", 10'	
H3		0.816	3'	19	3, 4, 5, 10	2', 6', 9', 11'
20 C		11.561			12', 14', 17	
H3		0.683	12'		12, 13, 14, 17	9', 11'
21 C		1.642		21	21	100
H3		0.225		21	21	
22 C		0.251		22	22	
H3		0.064		22	22	101
100 Si		17.402				21
101 Si		14.782				101

Remarks:

The relative stereochemistry of the is supported by observed NOESY cross-peaks as given in the table on the right and did not change during the reaction.

The relative stereochemistry at C1 was determined with the help of a J-HMBC (see next page).

P-ID: ML00xxx
Measured on: 03/11/2021
CHIFFRE:ZHH-ZC-201-02
ELNA#:7184
Client: Hui Zhou
Group: List
Spectroscopist: Leutzsch
Analysed on: 13/12/2021
Analysed by: Leutzsch
Amount: 10.0 mg
Solvent: CD2Cl2
Reference: 1H+13C on solvent,other nuclei w/ xiref
Temperature: 298 K
Spectrometer: av600neo
Probe: cryoBBO
Experiments: 1H-zg30, 13C-zgpg30, [13C, 1H]-hsqcedetgpsisp2.3, [13C, 1H]-hmbcetgpl3nd, [1H, 1H]-cosygpppqf, [1H, 1H]-noesygpphpp, 29Si-ineptrd, [29Si, 1H]-hmbcgpndqf, [13C, 1H]-hmbcetgpjcl2nd

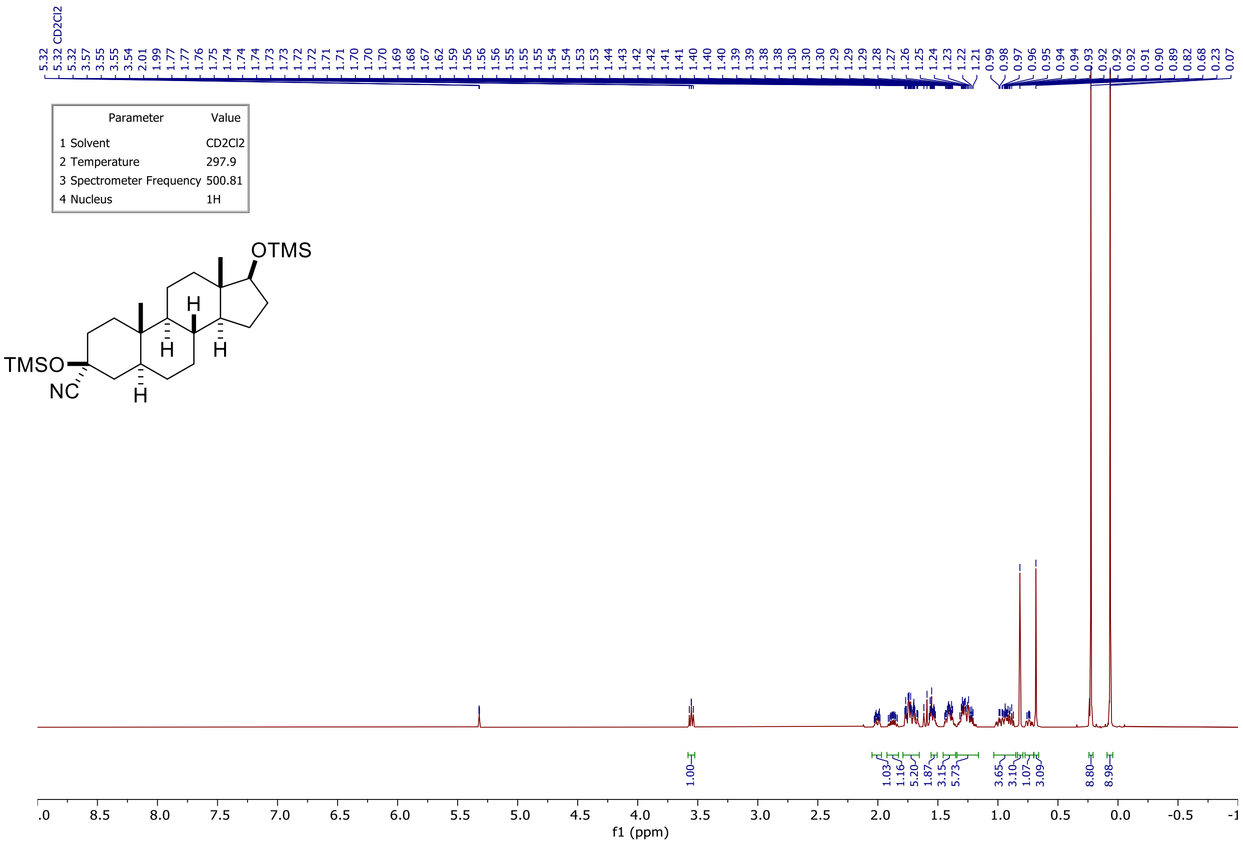

Parameter	Value
1 Solvent	CD2Cl2
2 Temperature	297.9
3 Spectrometer Frequency	125.94
4 Nucleus	13C

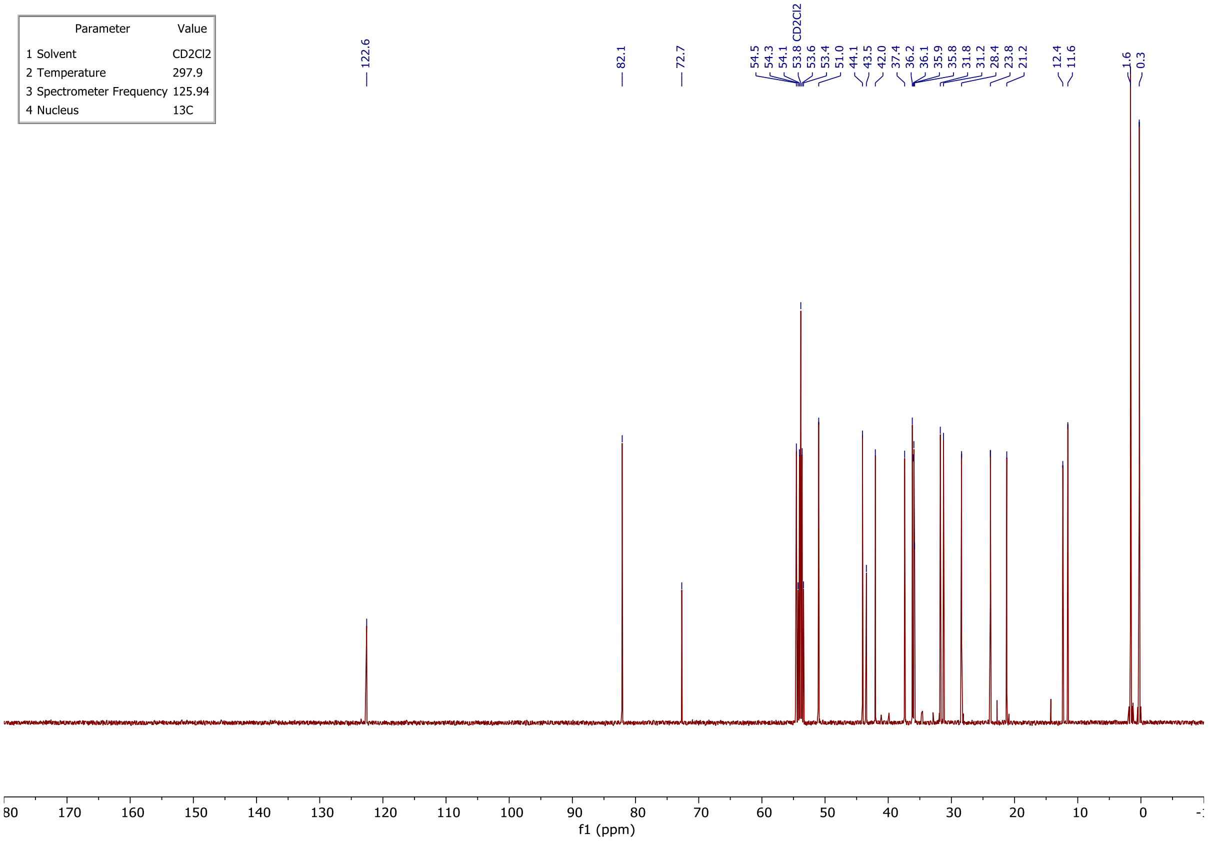

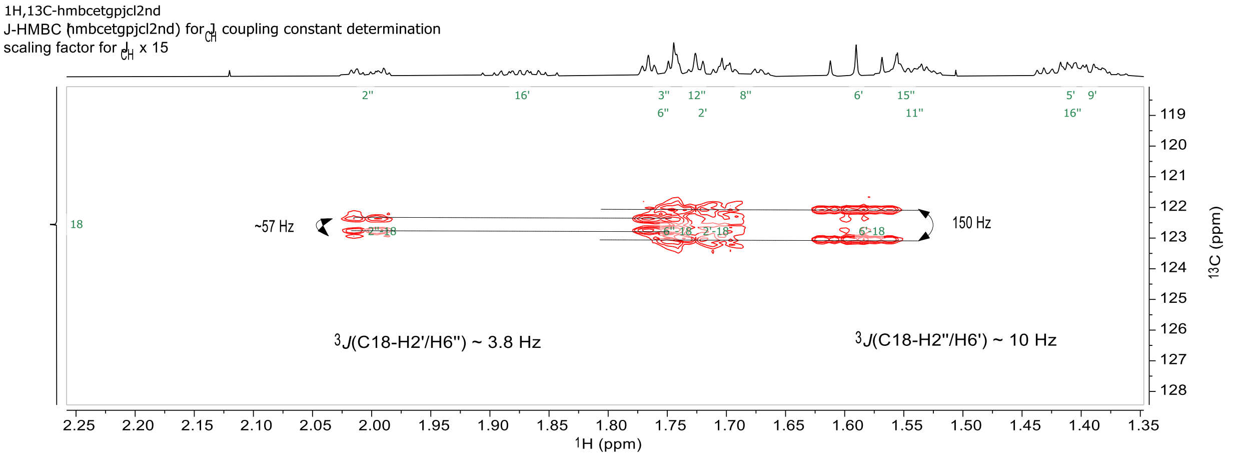

Possibilities

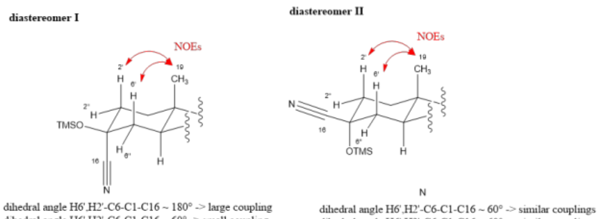

dihedral angle H6',H2'-C6-C1-C16 ~ 180° -> large coupling
dihedral angle H6',H2'-C6-C1-C16 ~ 60° -> small coupling

dihedral angle H6',H2'-C6-C1-C16 ~ 60° -> similar couplings
dihedral angle H6',H2'-C6-C1-C16 ~ 60° -> similar couplings

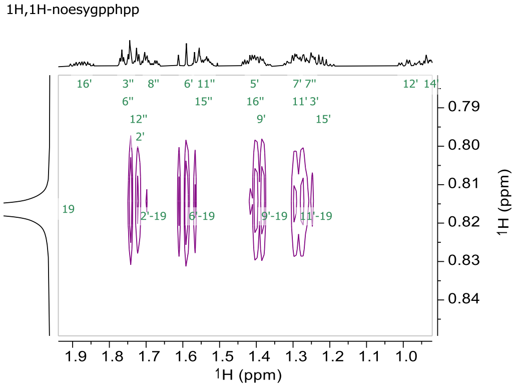

The observed data fits diastereomer I better as 2 significantly different J_{CH}-values of the axial and equatorial protons to C16 are observed.

1H NMR (600 MHz, CD_2Cl_2) δ 3.55 (t, J = 8.4 Hz, 1H), 2.01 (m, 1H), 1.87 (dddd, J = 13.4, 9.5, 8.8, 5.9 Hz, 1H), 1.73 (m, 5H), 1.56 (m, 3H), 1.40 (m, 3H), 1.28 (m, 5H), 0.94 (m, 3H), 0.82 (m, 3H), 0.74 (dd, J = 10.6, 4.2 Hz, 1H), 0.68 (d, J = 0.6 Hz, 3H), 0.23 (s, 9H), 0.06 (s, 9H).

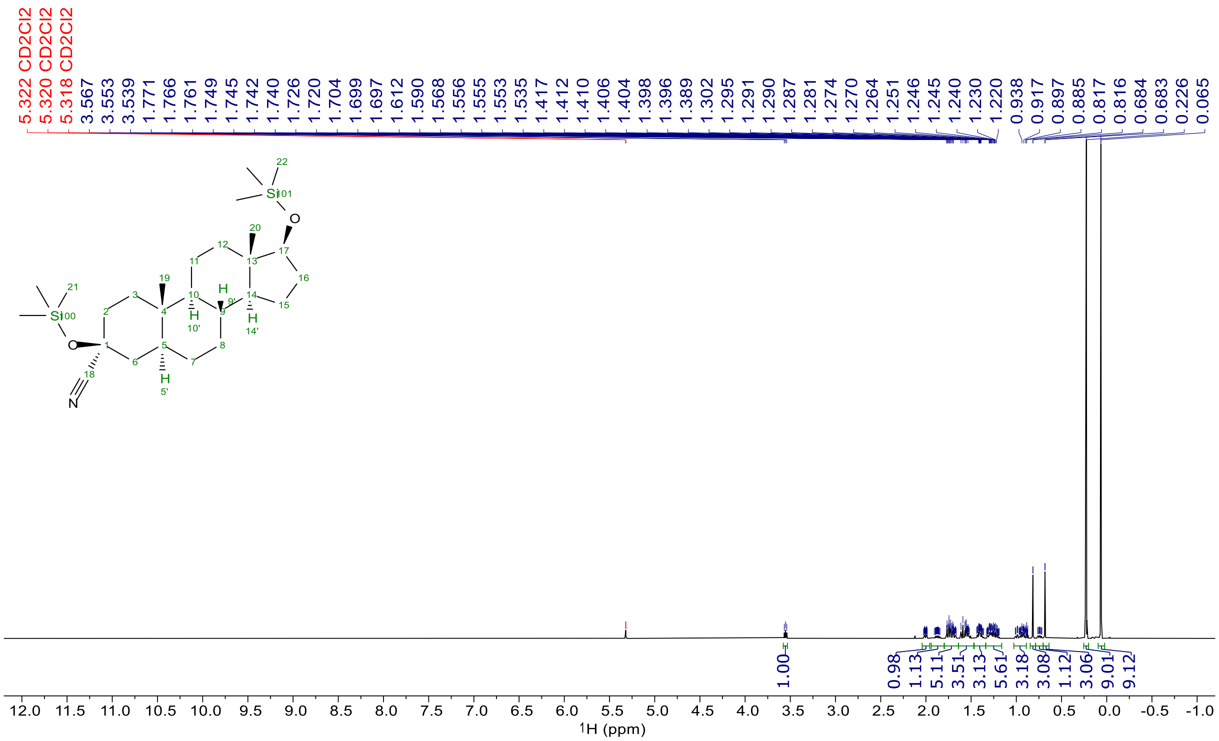

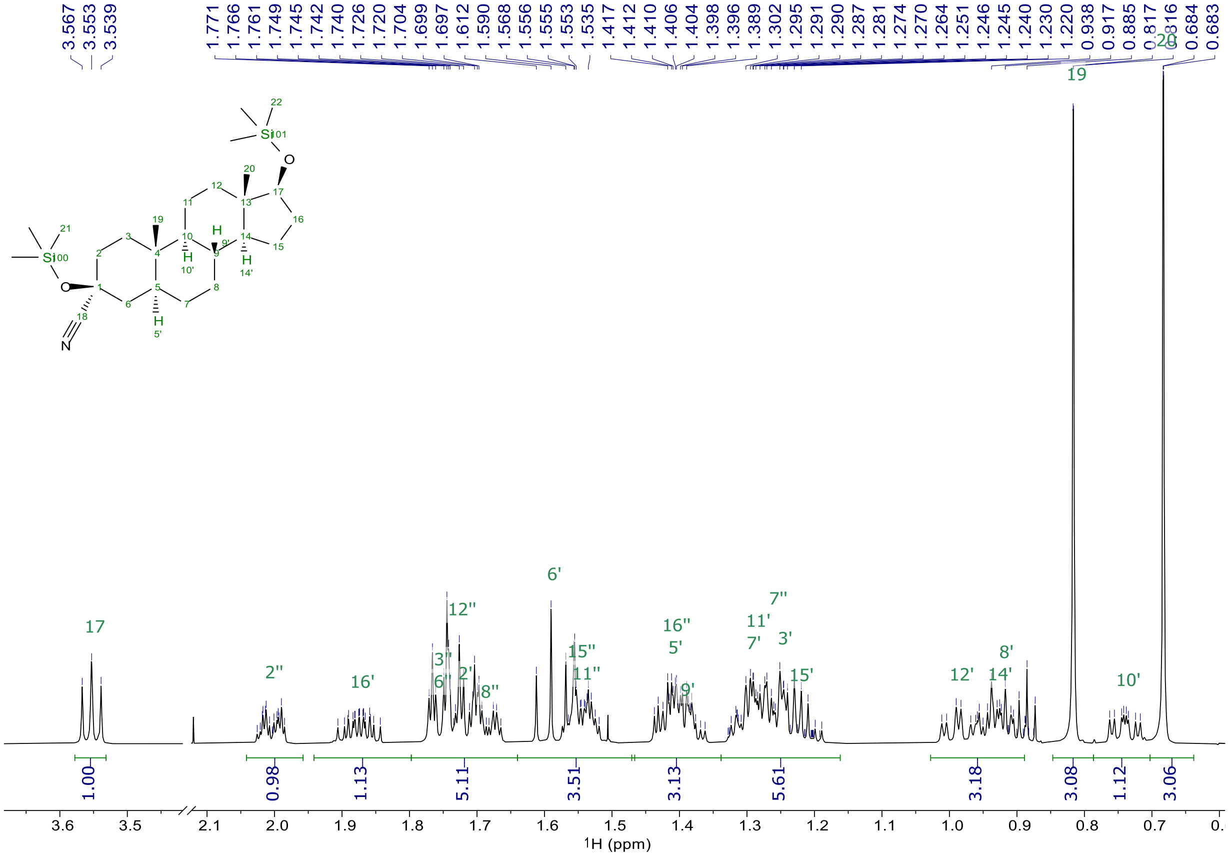

^{13}C NMR (151 MHz, CD_2Cl_2) δ 122.6, 82.1, 72.7, 54.5, 51.0, 44.1, 43.4, 42.0, 37.4, 36.2, 36.0, 35.9, 35.8, 31.7, 31.2, 28.4, 23.8, 21.2, 12.4, 11.6, 1.6, 0.3.

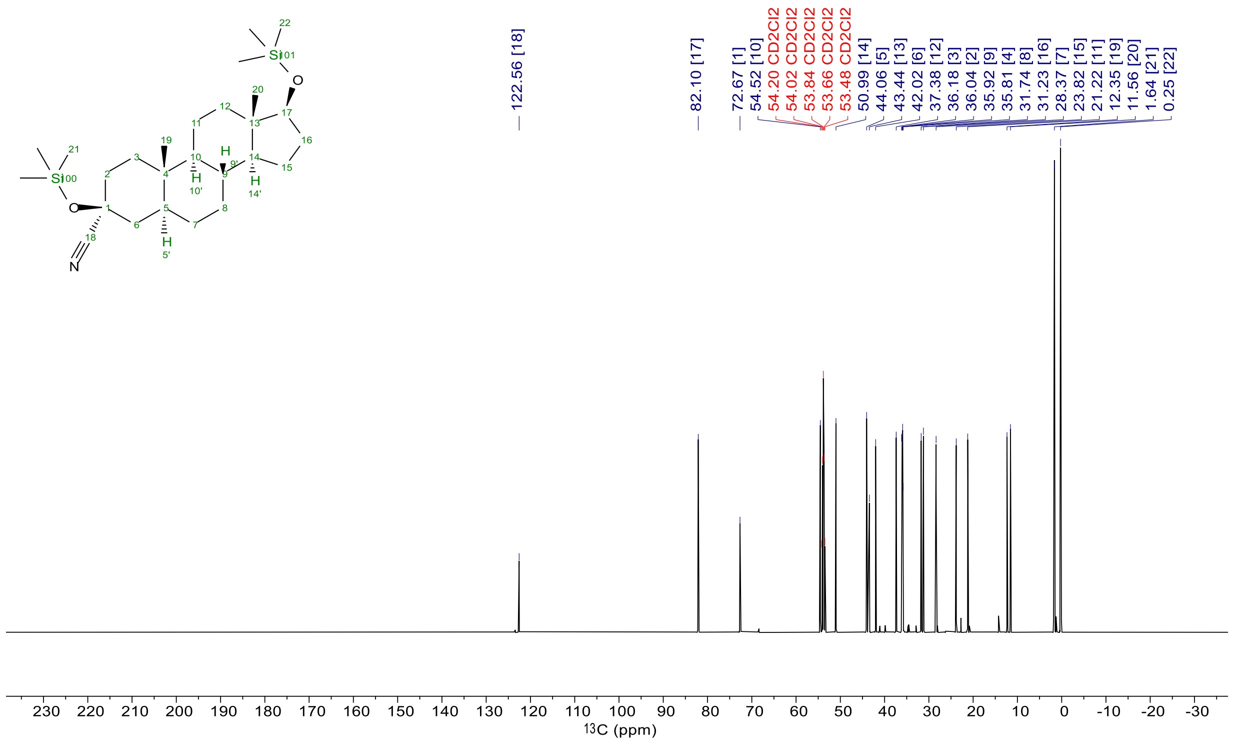

1H,13C-hsqcedetgpsisp2.3

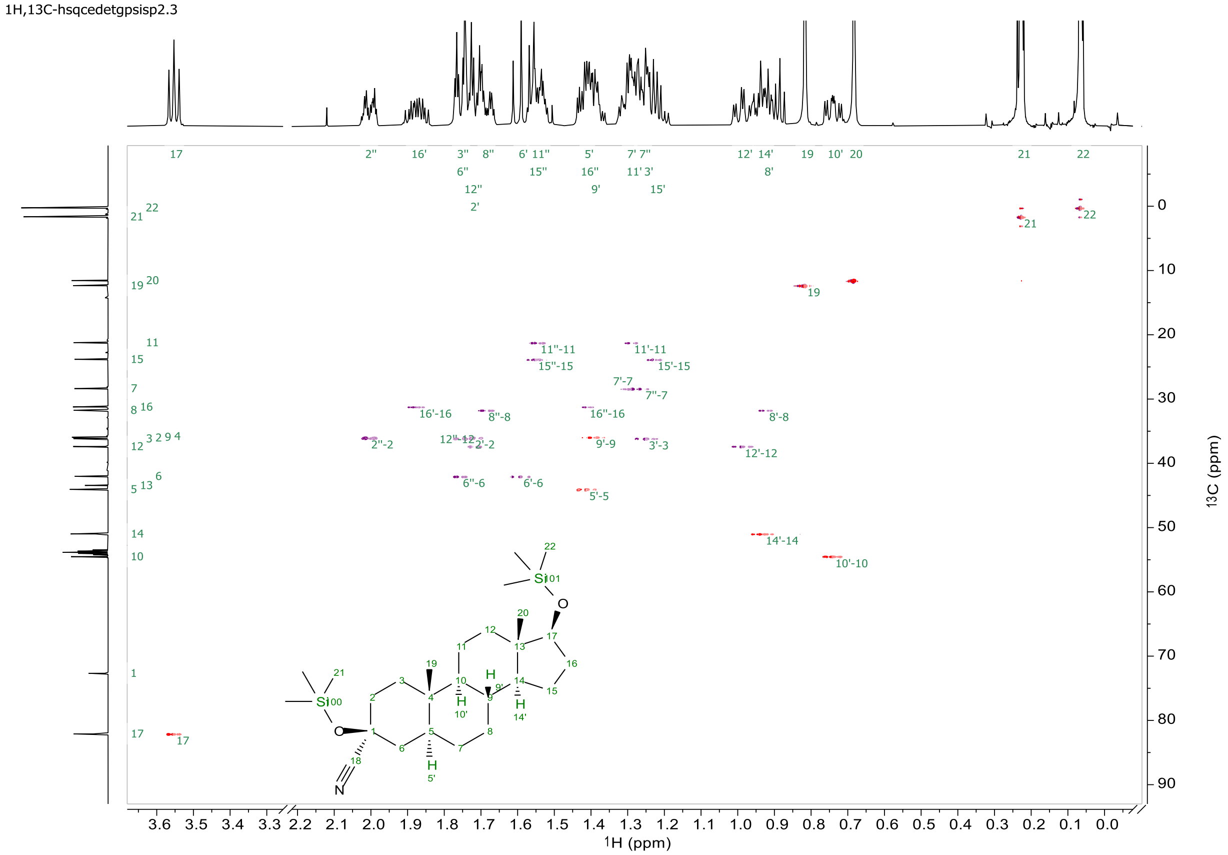

1H,13C-hmbcetgpl3nd

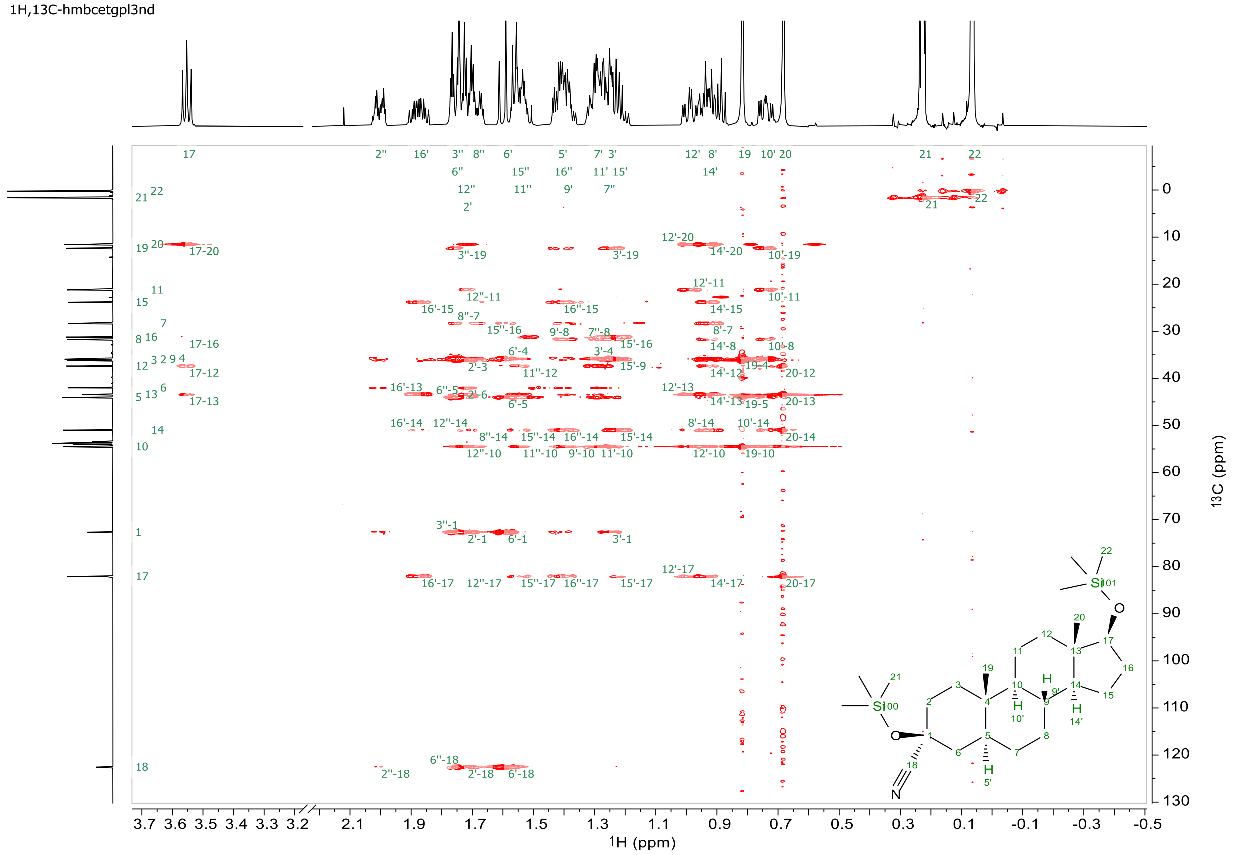

1H,1H-cosygpppqf

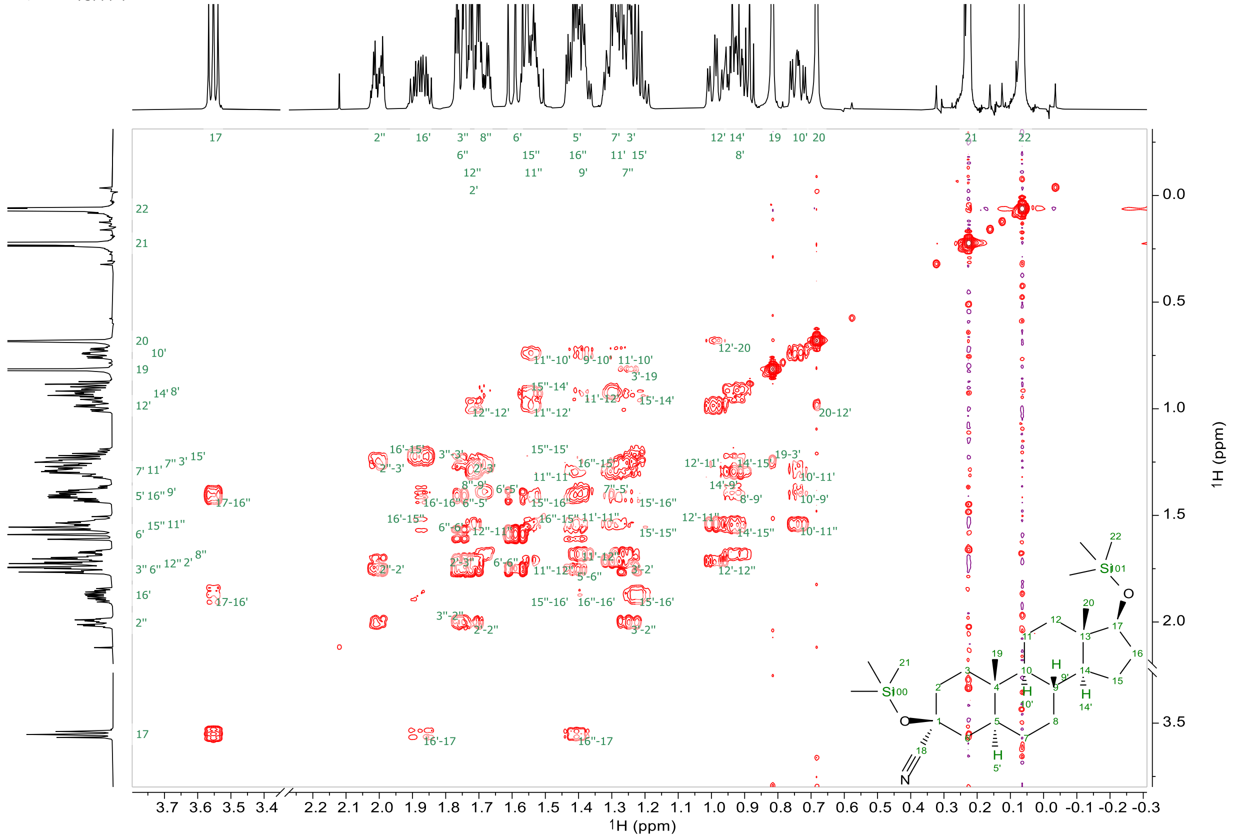

1H,1H-noesygpphpp

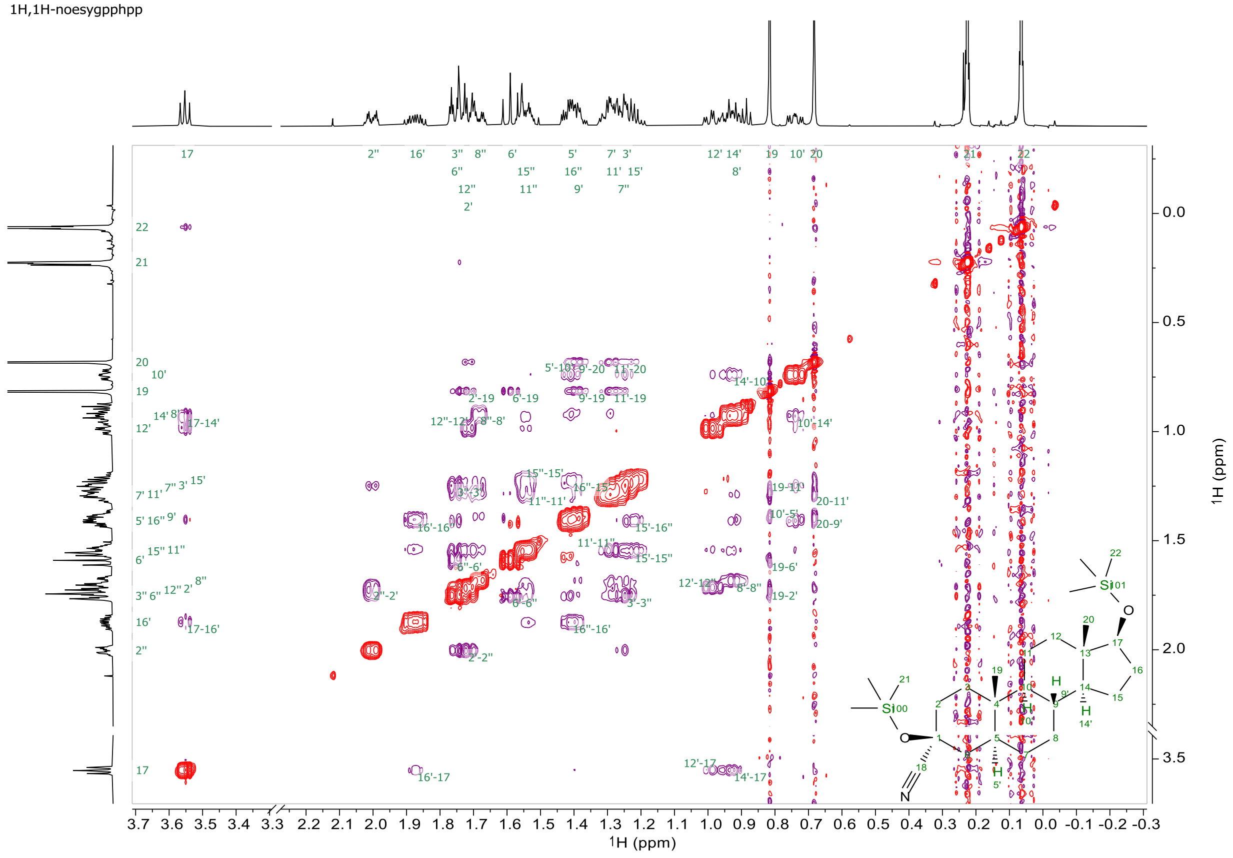

29Si,-ineptrd

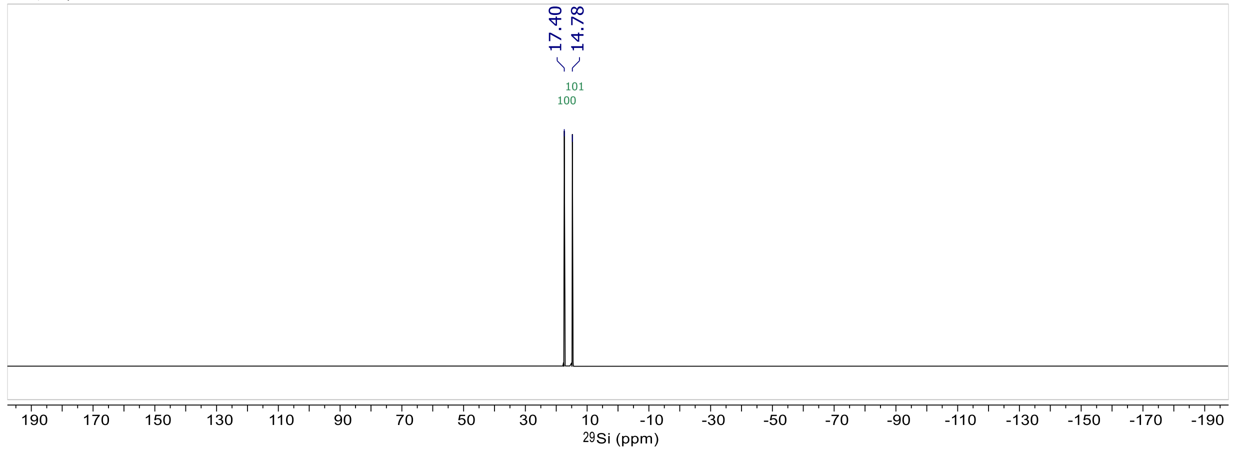

1H,29Si-hmbcgpndqf

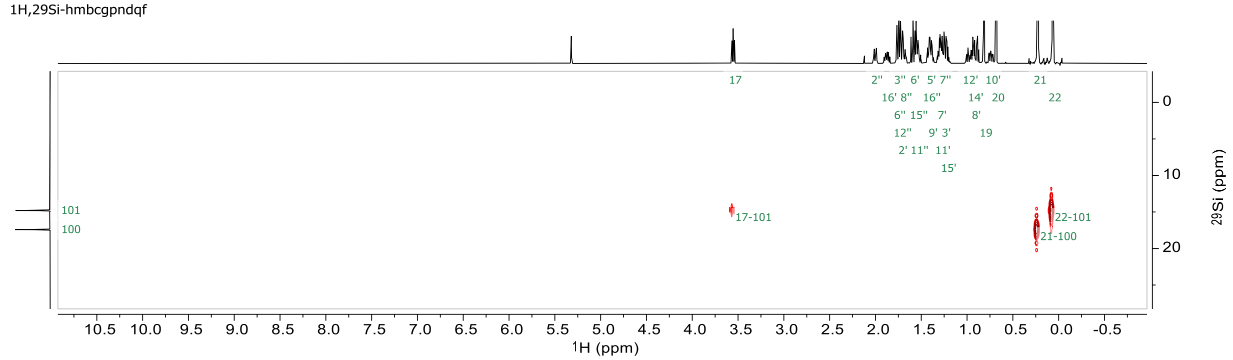

4-bromo-N-(2-hydroxy-2-methyl-4-phenylbutyl)benzenesulfonamide derivatized from 46

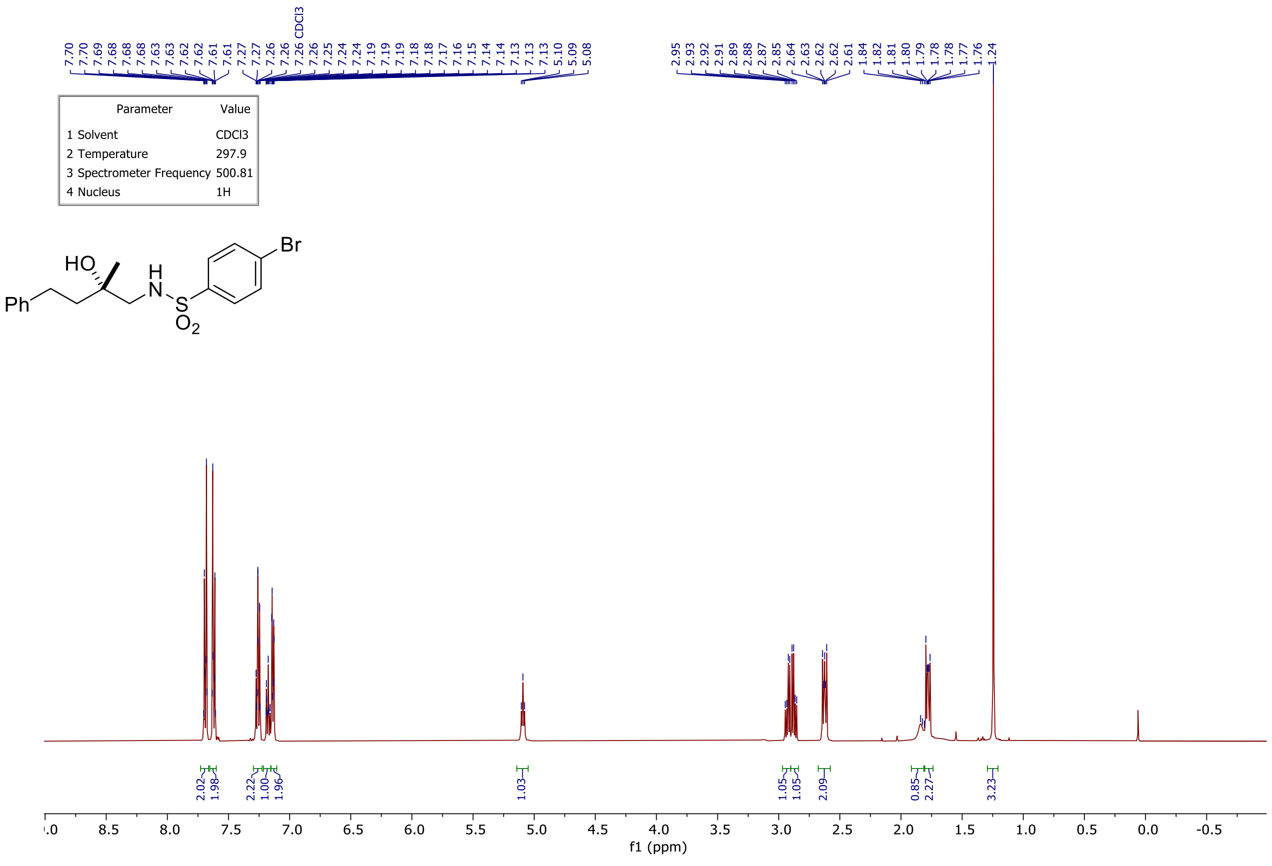

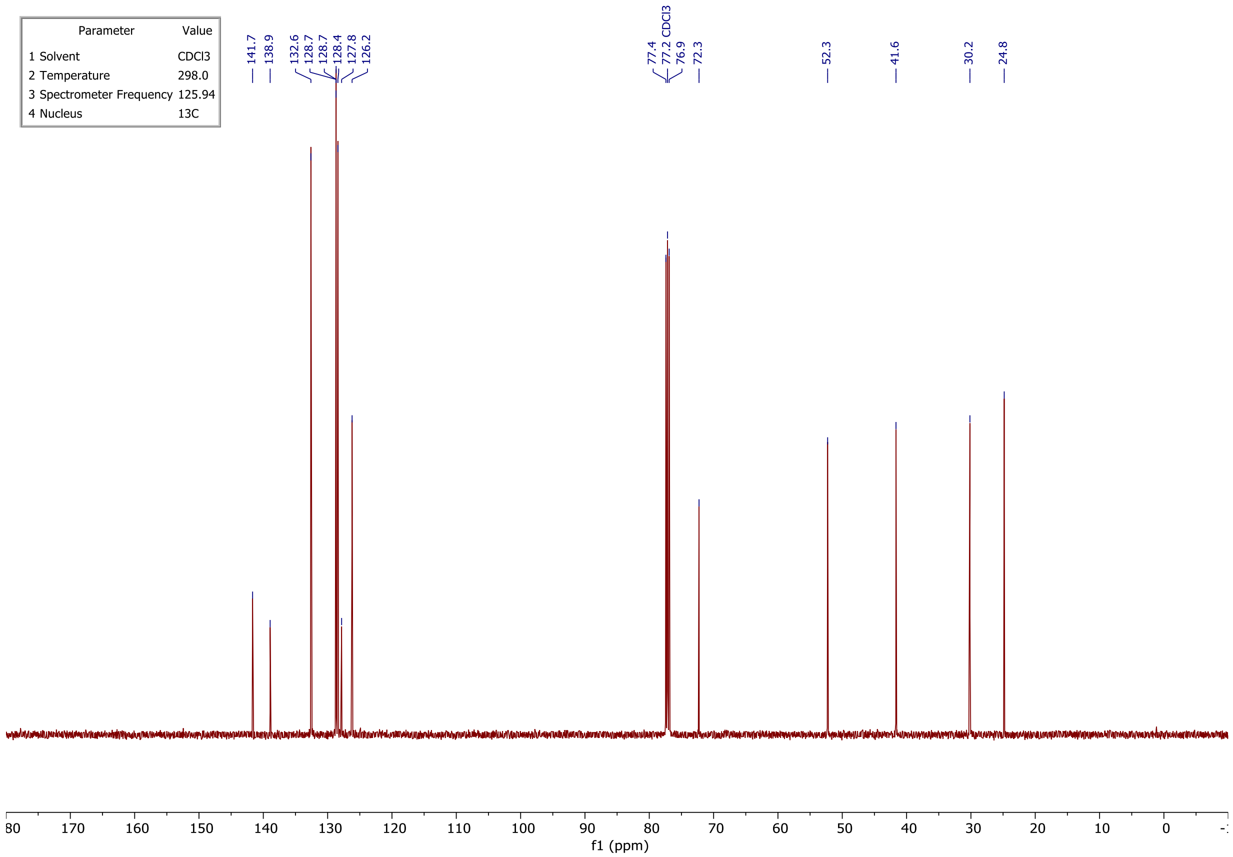

2-hydroxy-2-methyl-4-phenylbutanenitrile 47

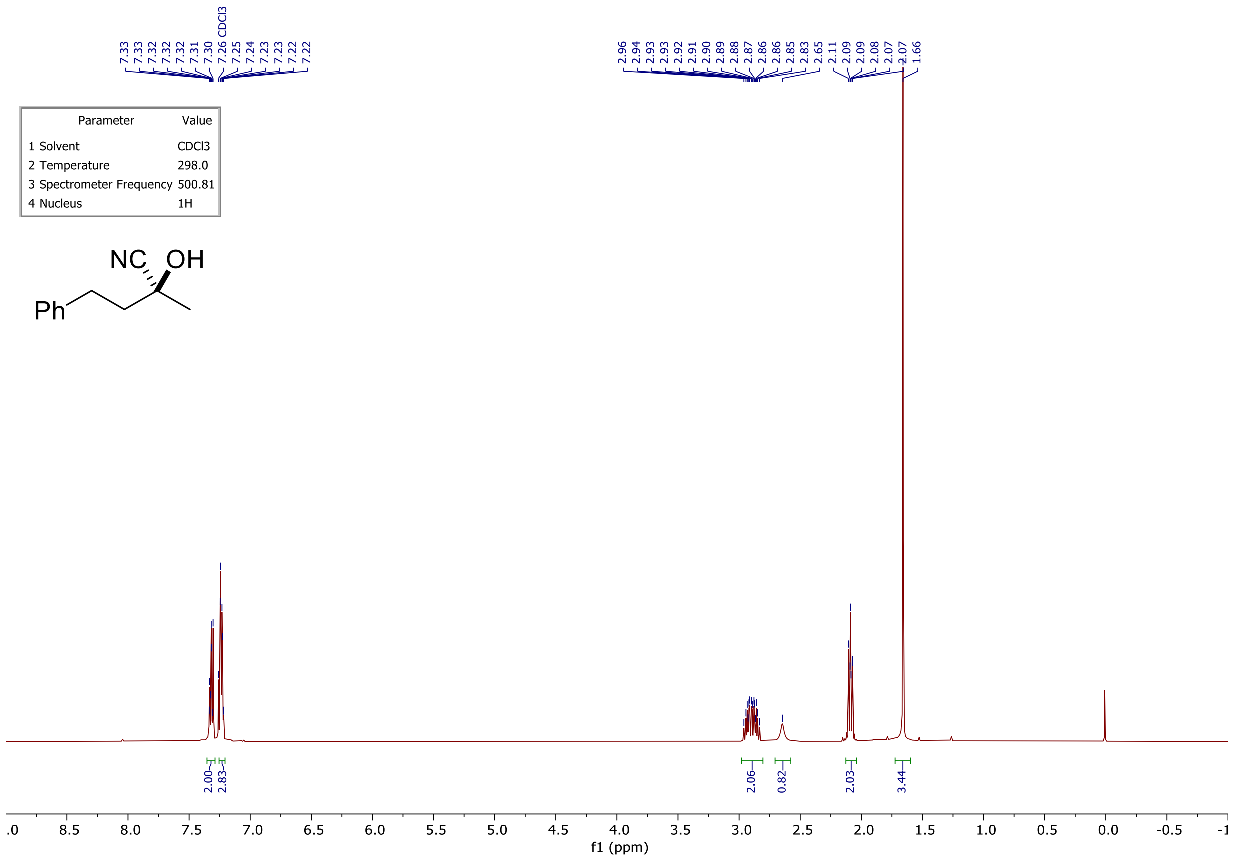

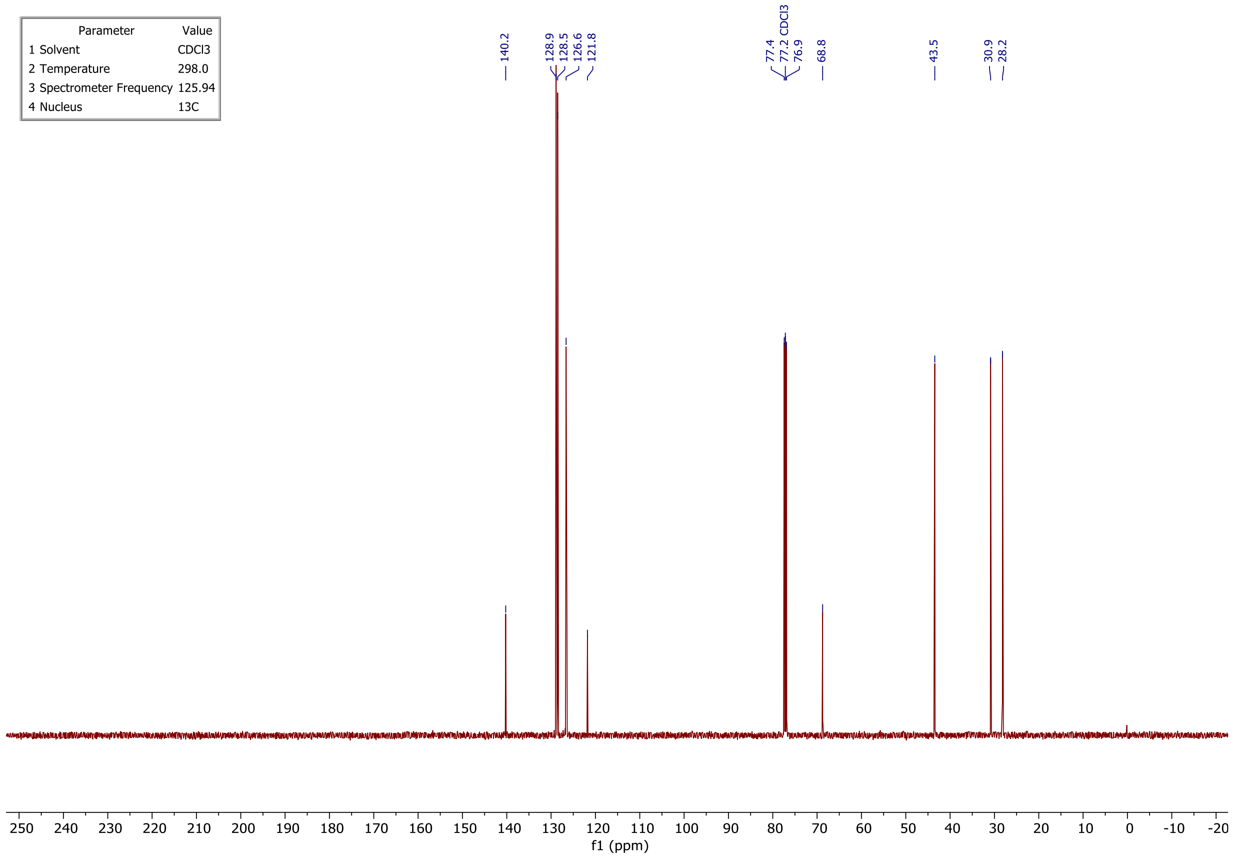

2-cyano-4-phenylbutan-2-yl acetate derivatized from 47

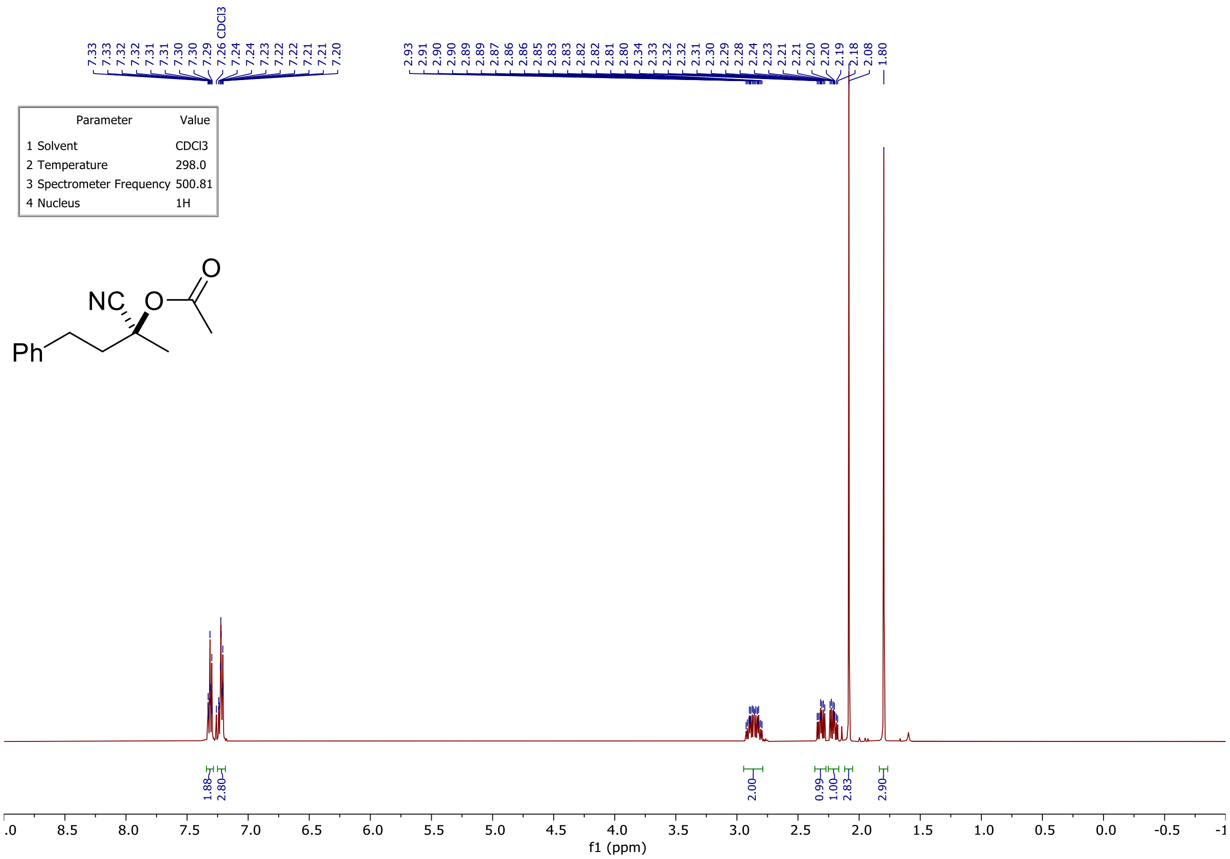

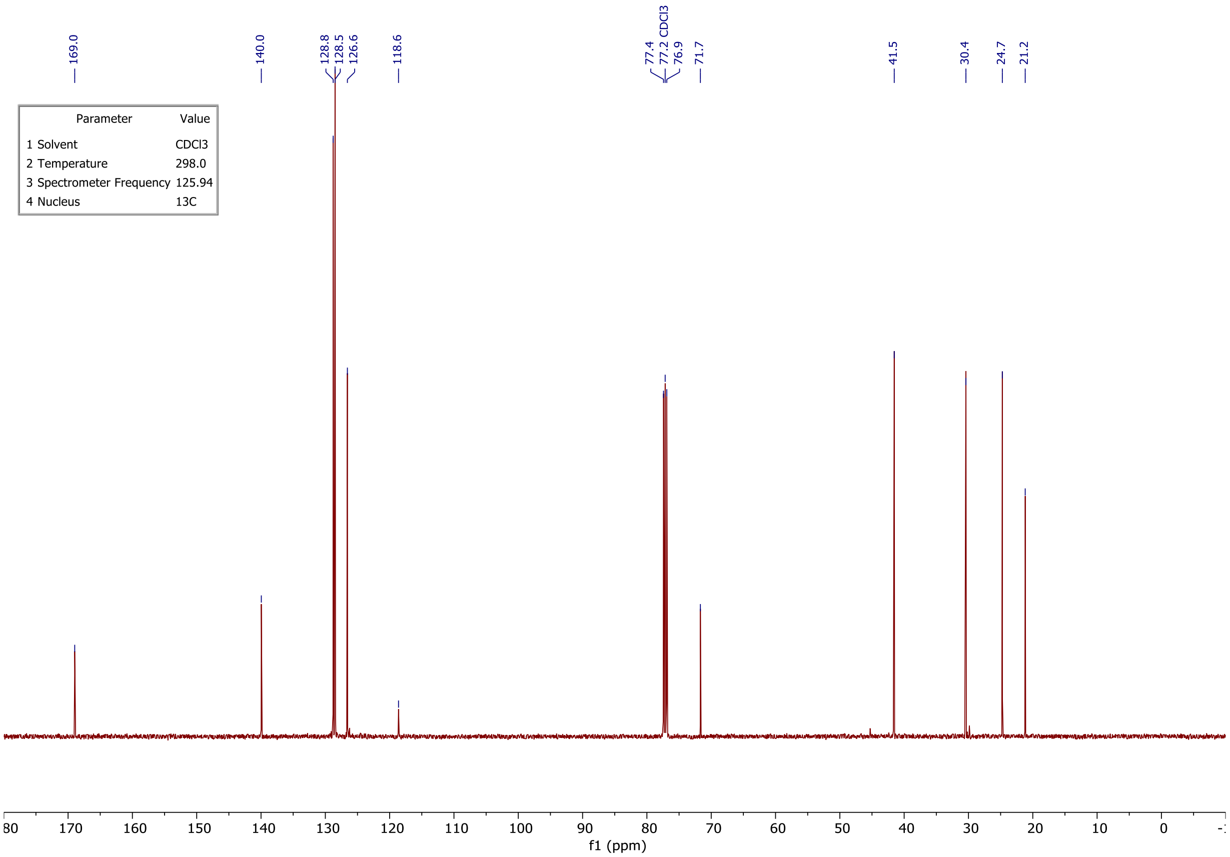

2-methyl-4-phenyl-2-((trimethylsilyl)oxy)butanal 48

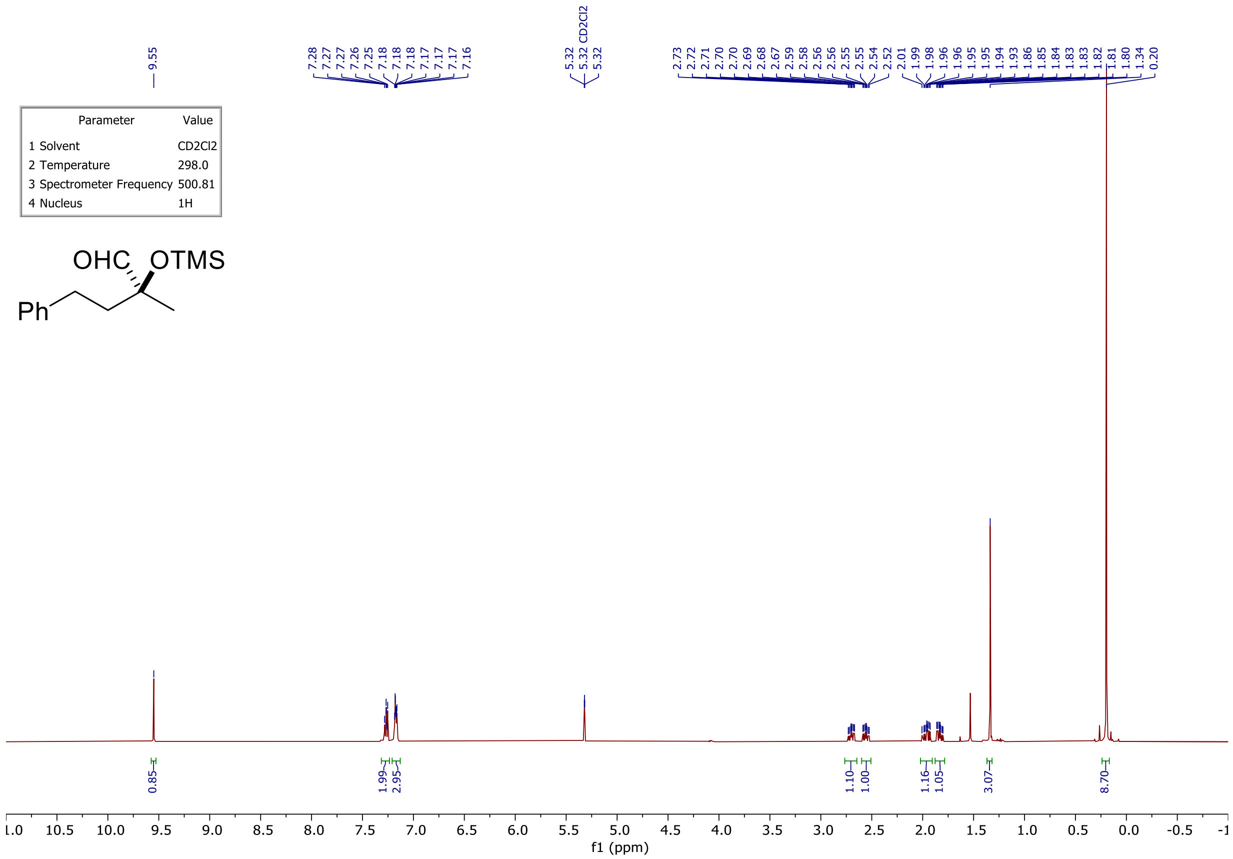

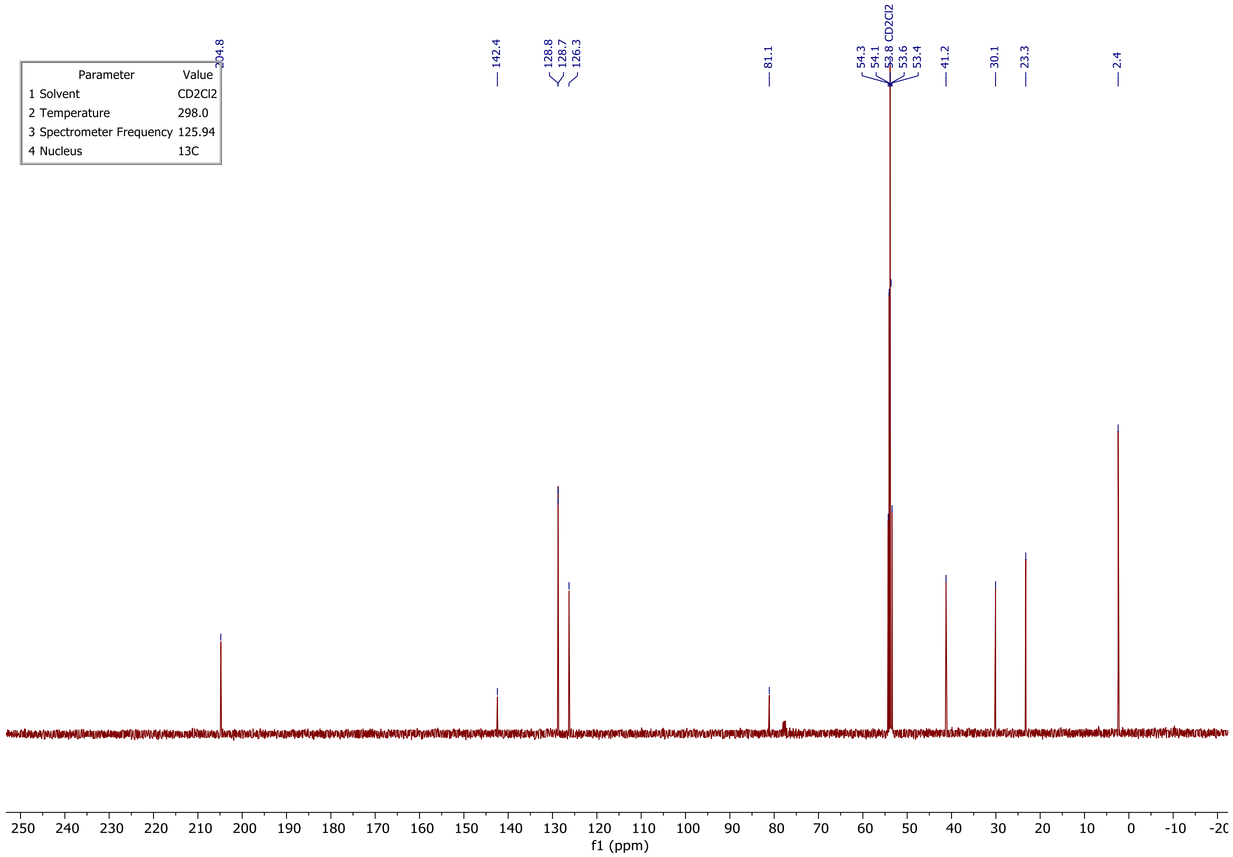

5-methyl-5-phenethyloxazolidin-2-one 49

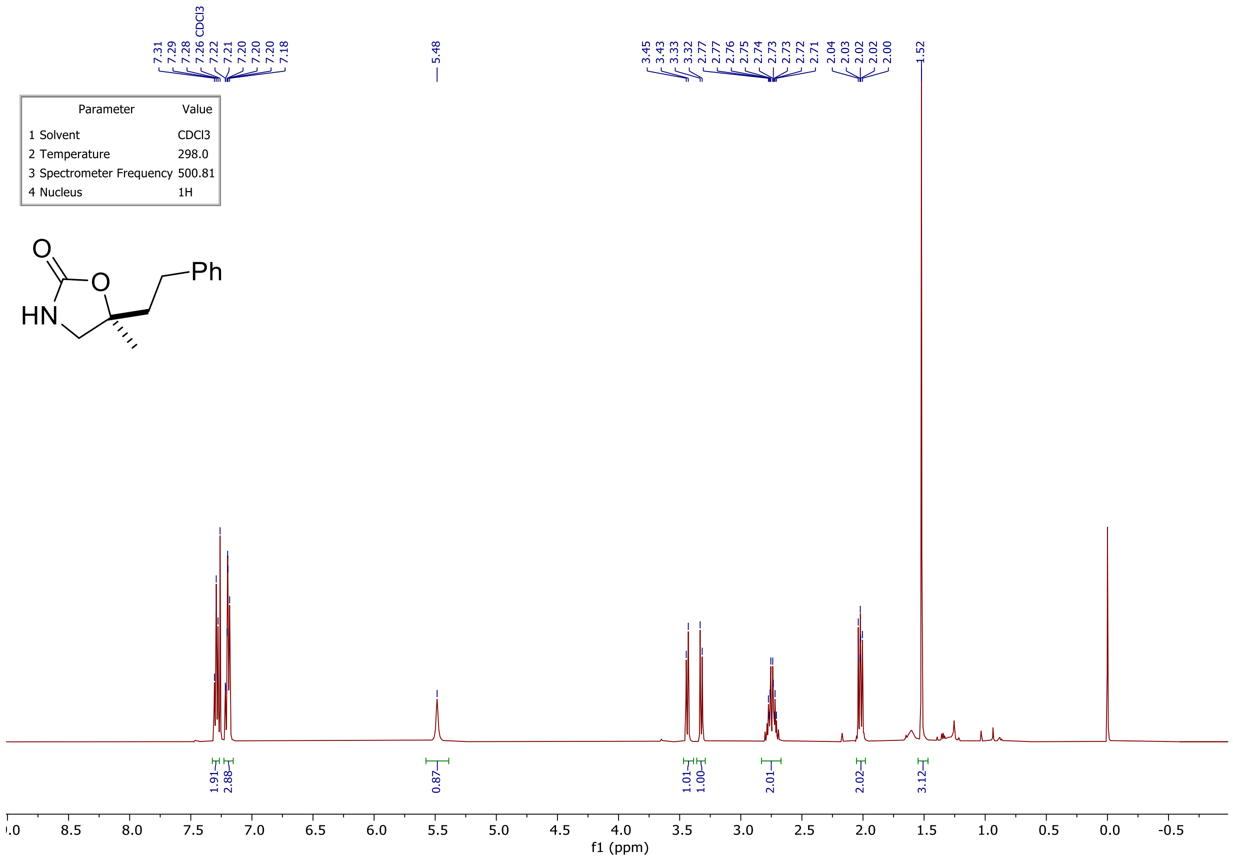

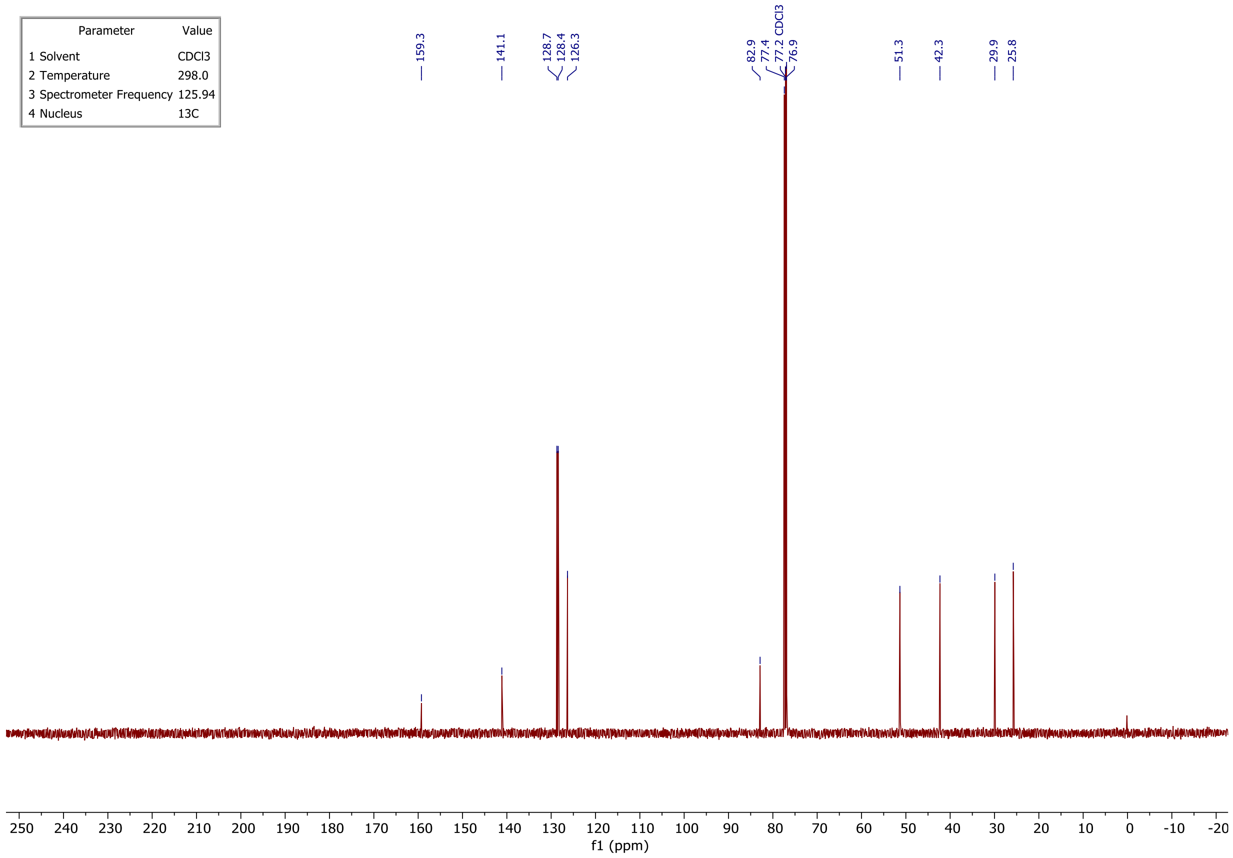

(*S*)-2-hydroxy-2-methylbutanoic acid 50

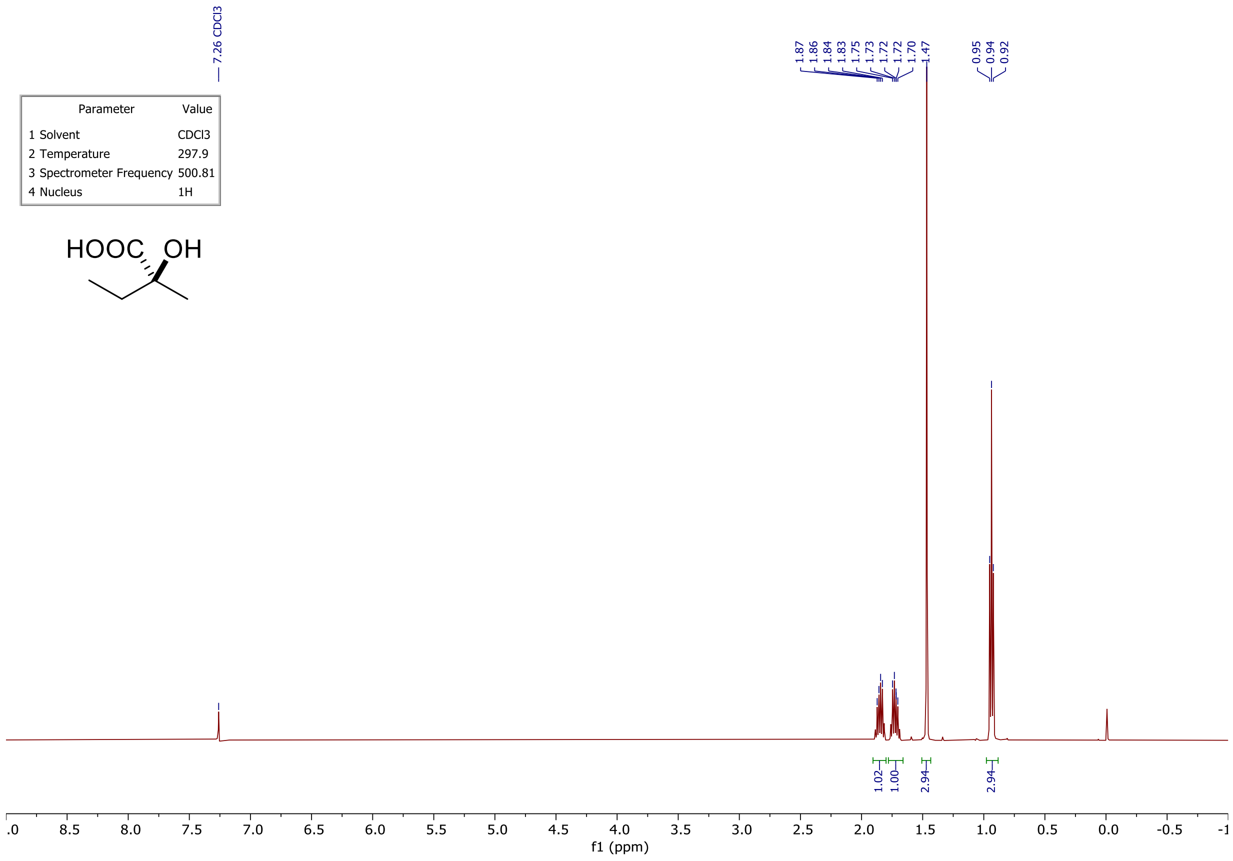

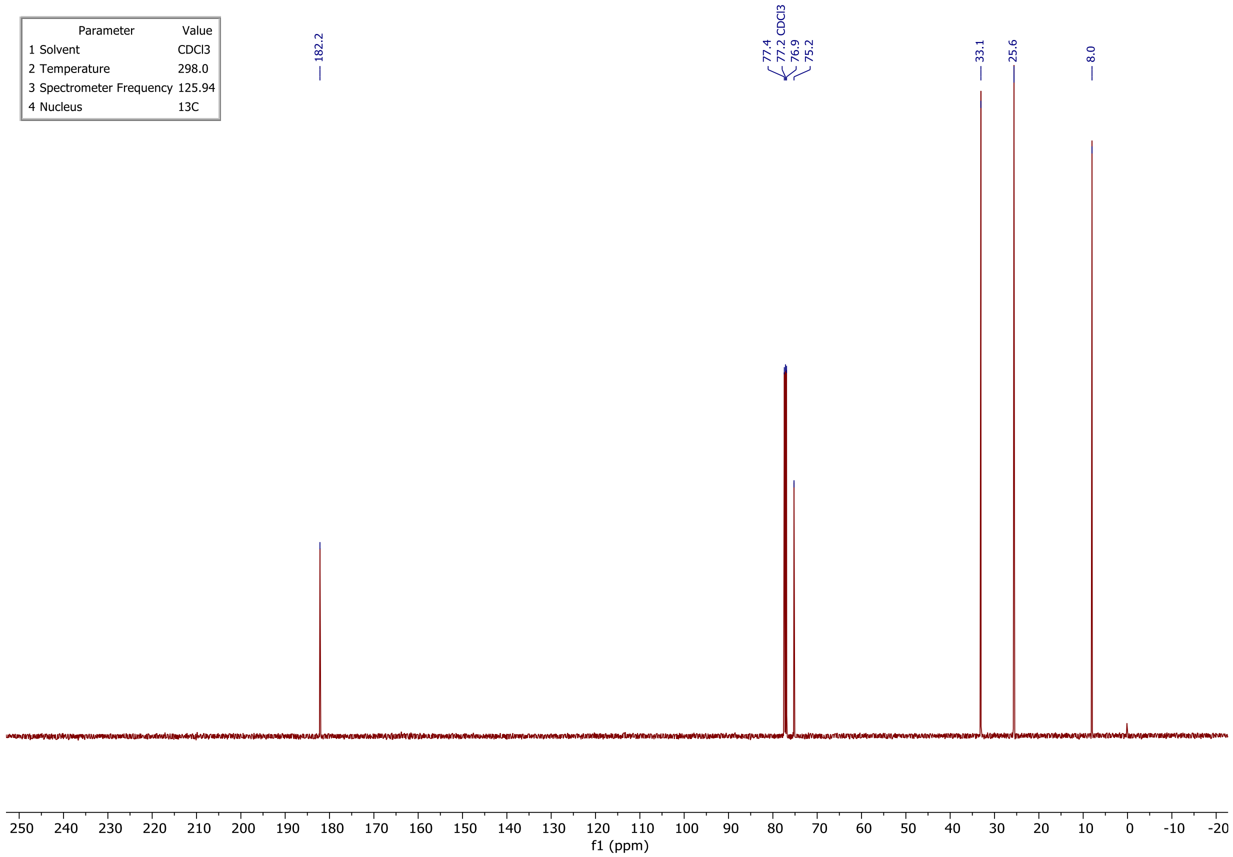

Spectrum of product **53** and **54** from the reaction of ketone **52** and TMSCN catalyzed by DSI-I.

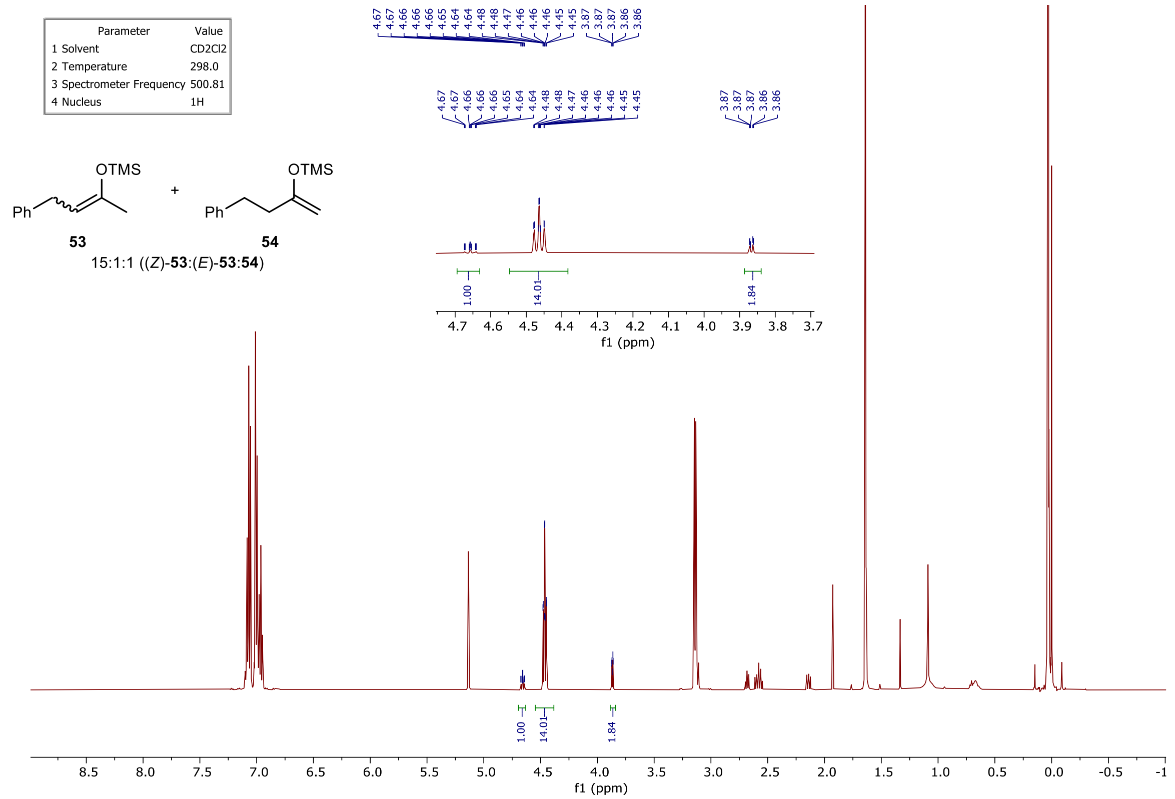

Spectrum of product **53** and **54** from reaction reported previously.[37]

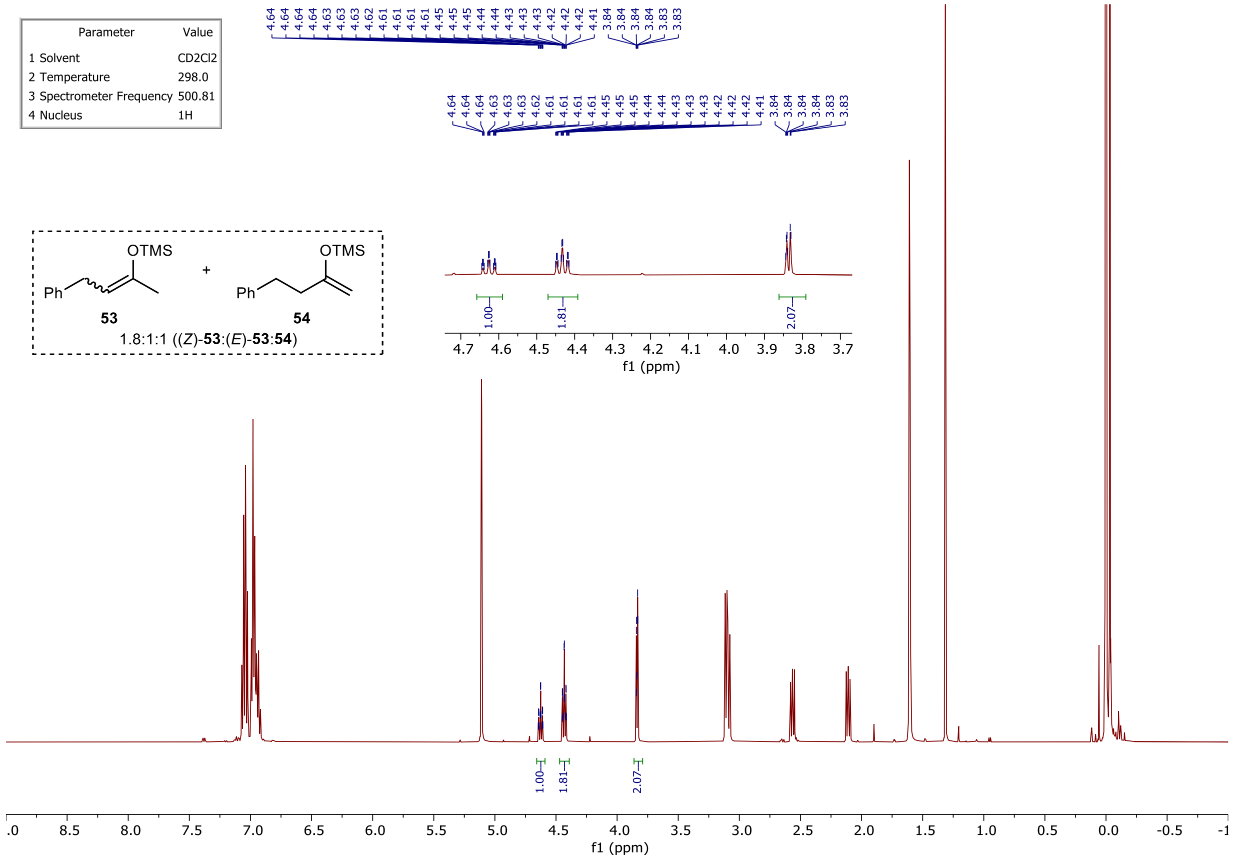

3-bromo-4'-(trifluoromethyl)-1,1'-biphenyl

Parameter	Value
1 Solvent	CDCl3
2 Temperature	298.0
3 Spectrometer Frequency	500.81
4 Nucleus	1H

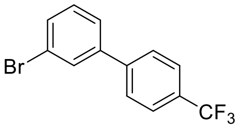

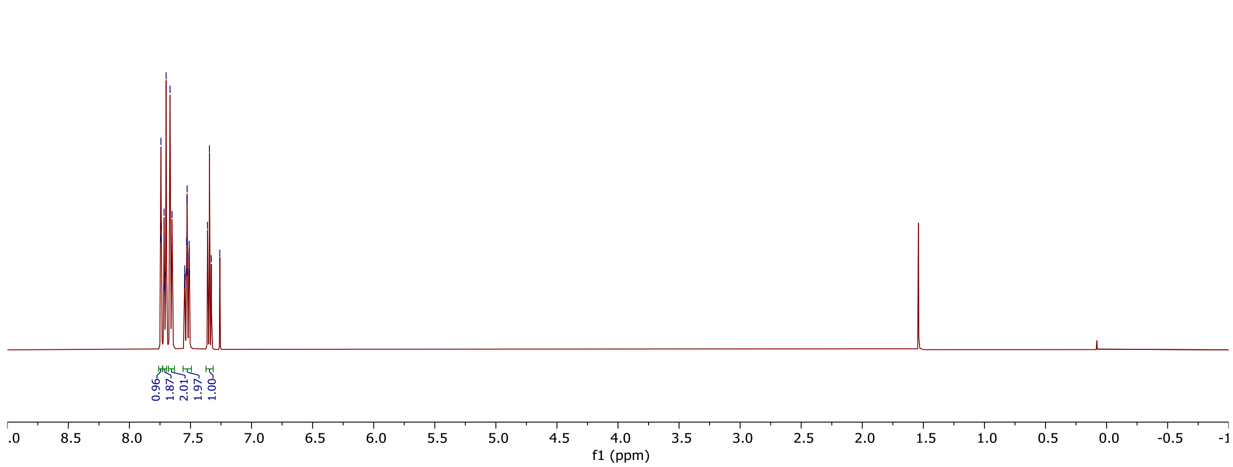

Parameter	Value
1 Solvent	CDCl3
2 Temperature	298.0
3 Spectrometer Frequency	125.94
4 Nucleus	13C

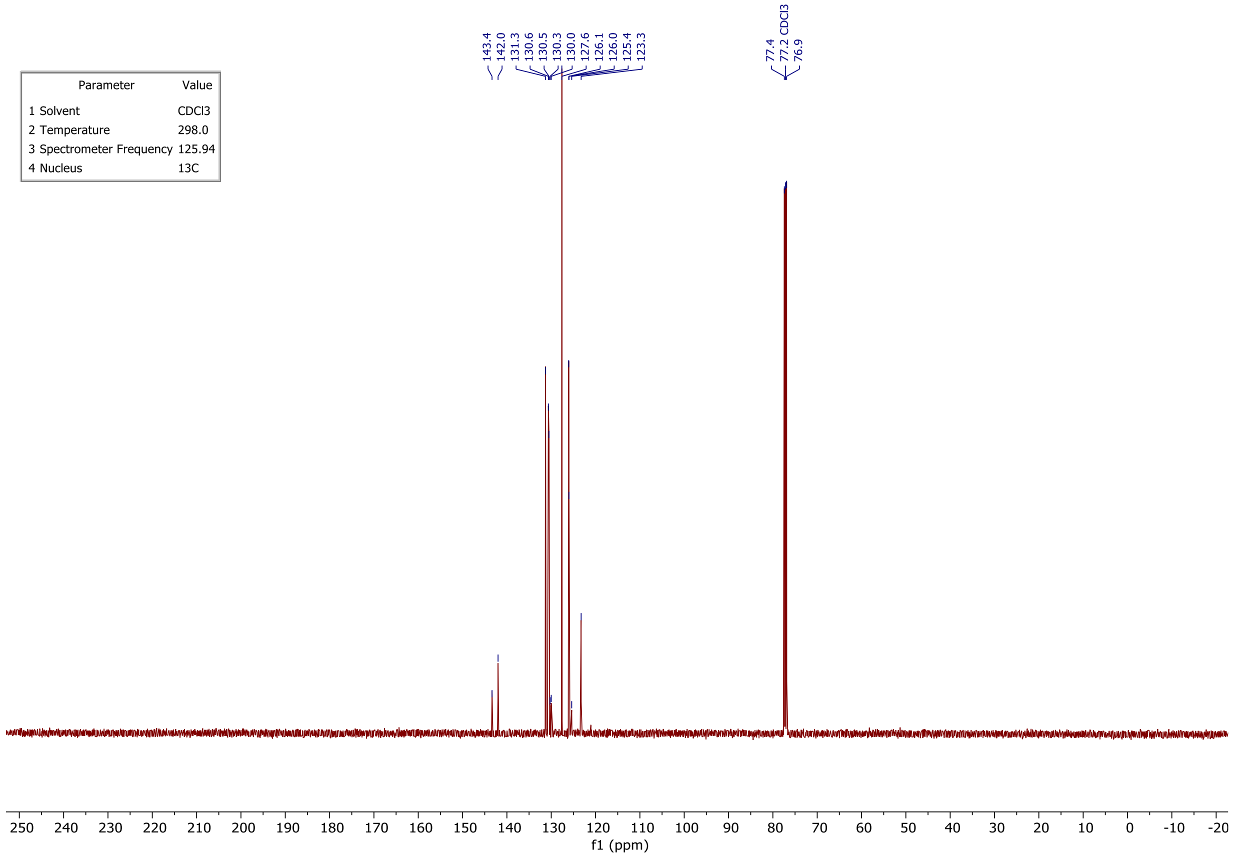

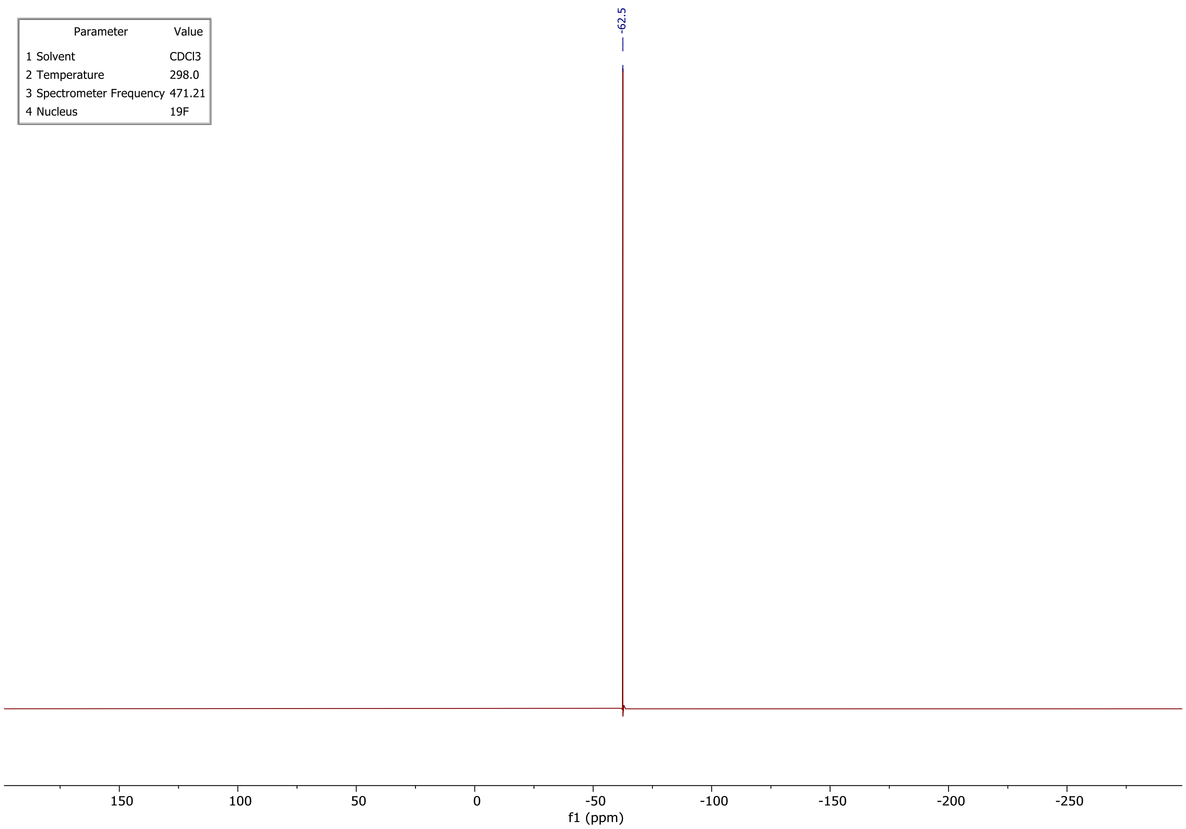

(*S*)-2,2'-bis(methoxymethoxy)-3,3'-bis(4'-(trifluoromethyl)-[1,1'-biphenyl]-3-yl)-1,1'-binaphthalene

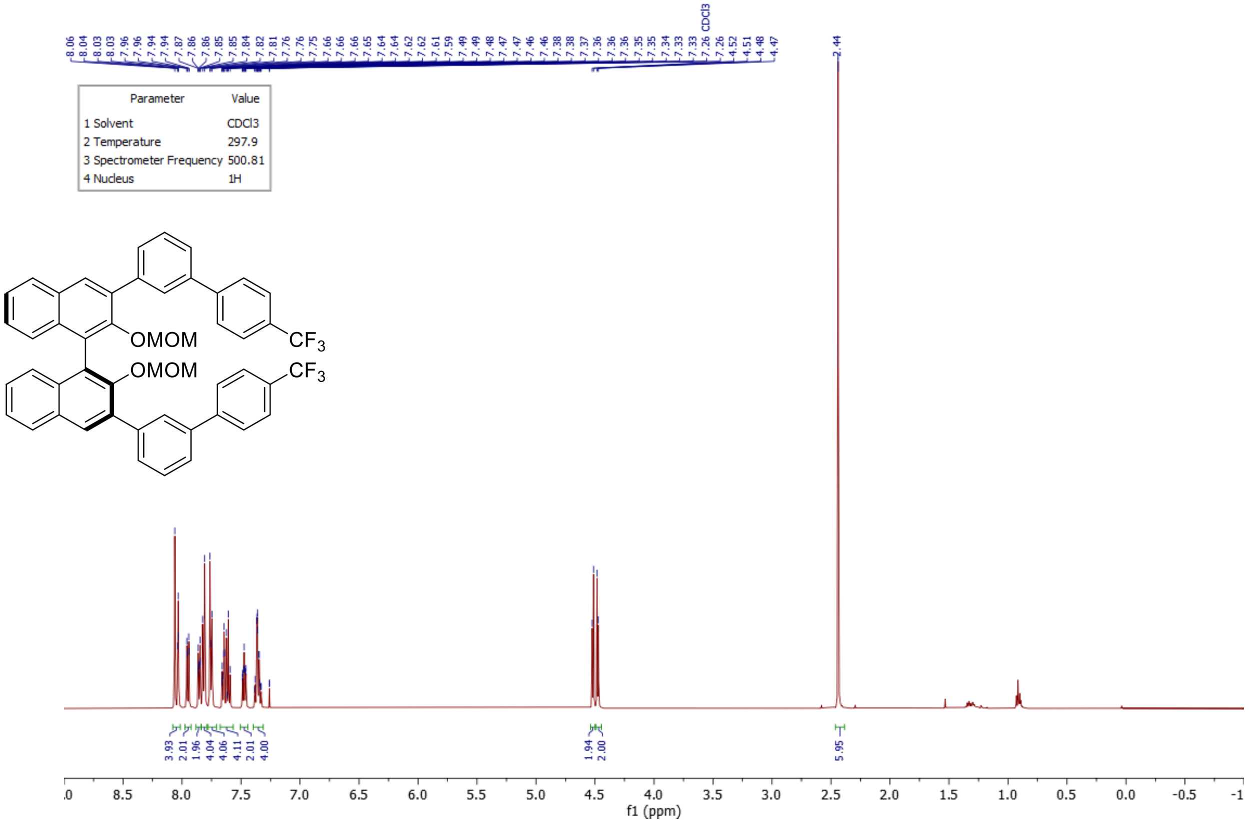

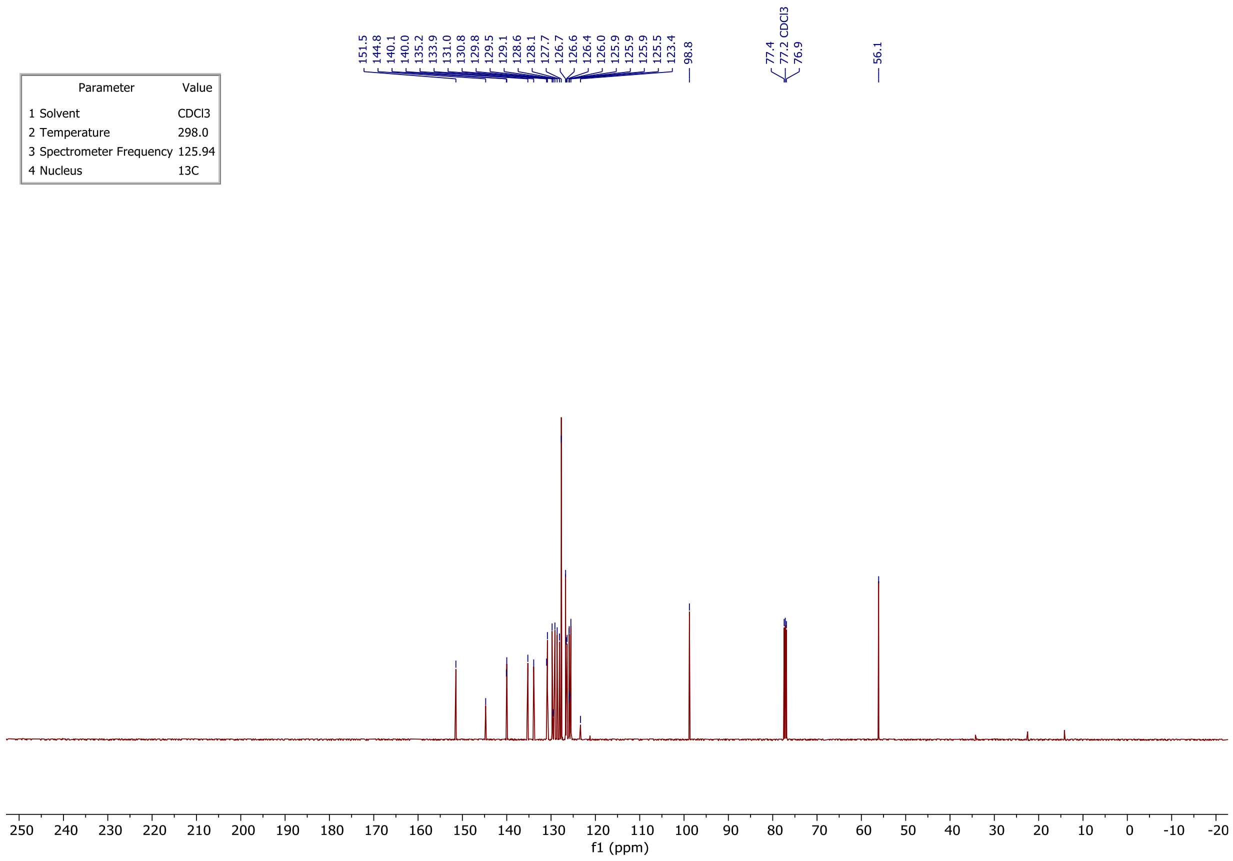
Parameter Value
1 Solvent CDCl3
2 Temperature 298.0
3 Spectrometer Frequency 125.94
4 Nucleus 13C
151.5
144.8
140.1
140.0
135.2
133.9
131.0
130.8
129.8
129.5
129.1
128.6
128.1
127.7
126.7
126.6
126.4
126.0
125.9
125.9
125.9
125.5
123.4
98.8
77.4
77.2 CDCl3
76.9
56.1
f1 (ppm)

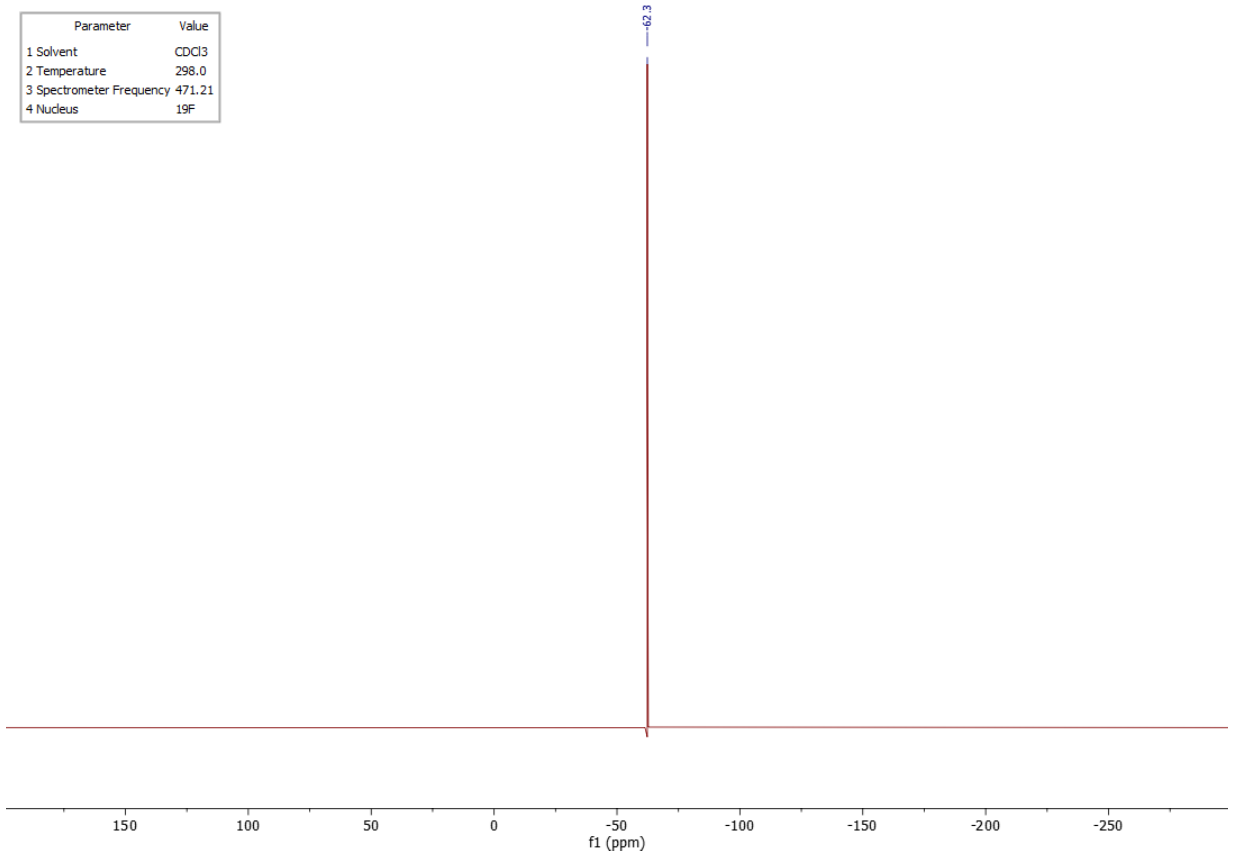
Parameter Value
1 Solvent CDCl3
2 Temperature 298.0
3 Spectrometer Frequency 471.21
4 Nucleus 19F
-62.3
f1 (ppm)

(*S*)-3,3'-bis(4'-(trifluoromethyl)-[1,1'-biphenyl]-3-yl)-[1,1'-binaphthalene]-2,2'-diol

Parameter	Value
1 Solvent	CDCl3
2 Temperature	297.9
3 Spectrometer Frequency	500.81
4 Nucleus	1H

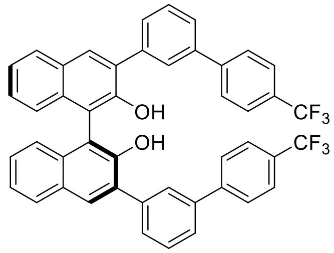

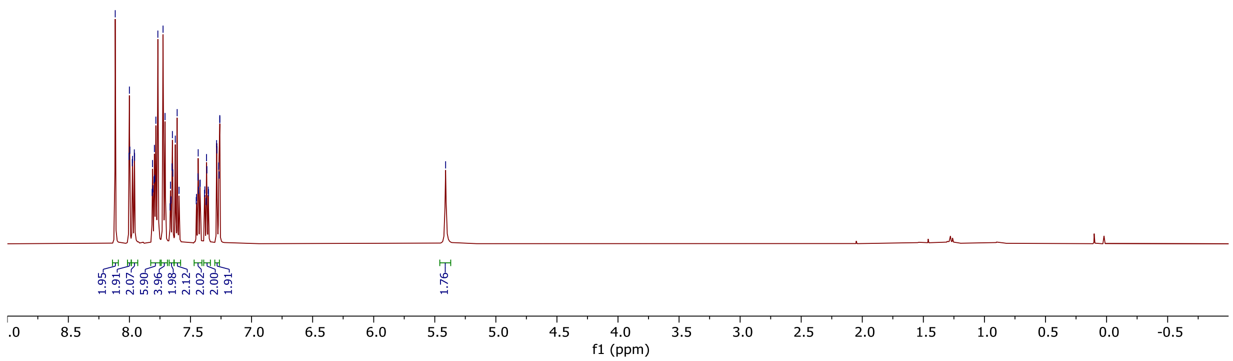

Parameter	Value
1 Solvent	CD2Cl2
2 Temperature	298.0
3 Spectrometer Frequency	125.94
4 Nucleus	13C

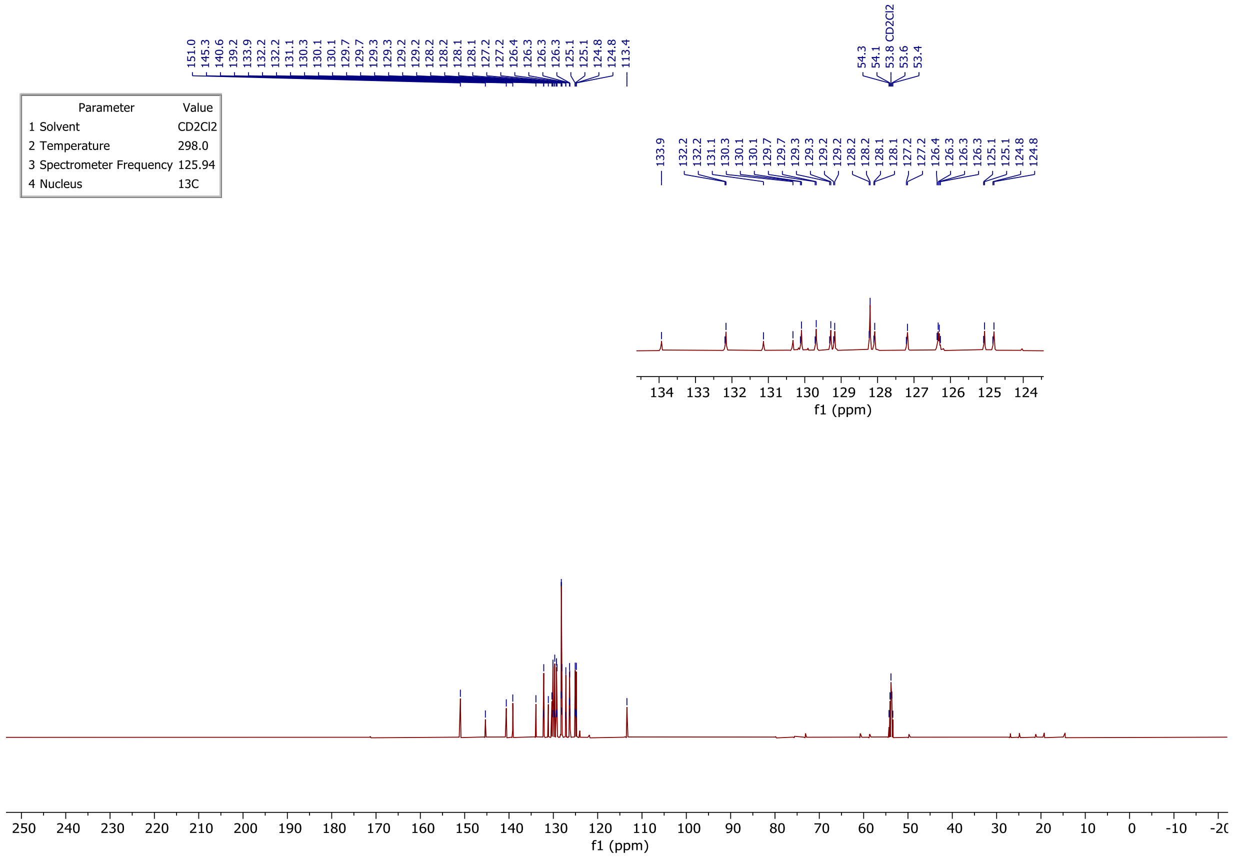

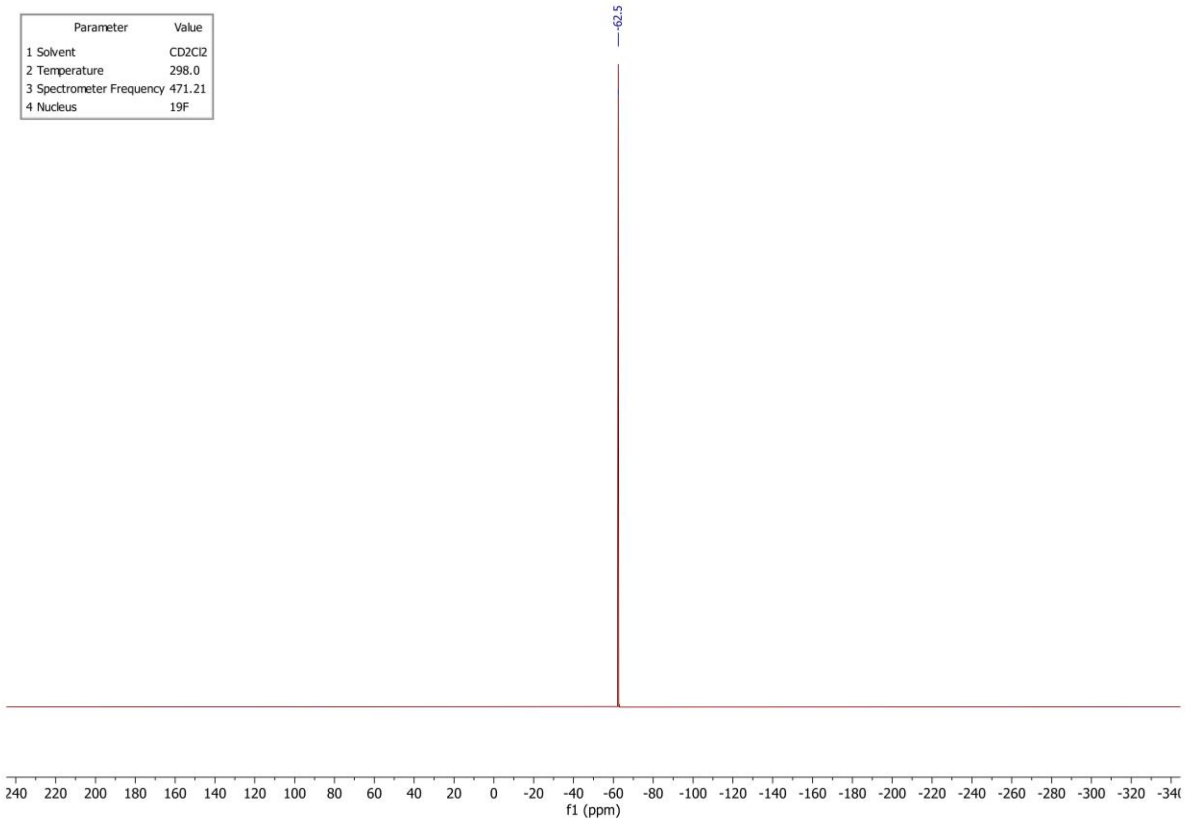

3-(3-bromophenyl)thiophene

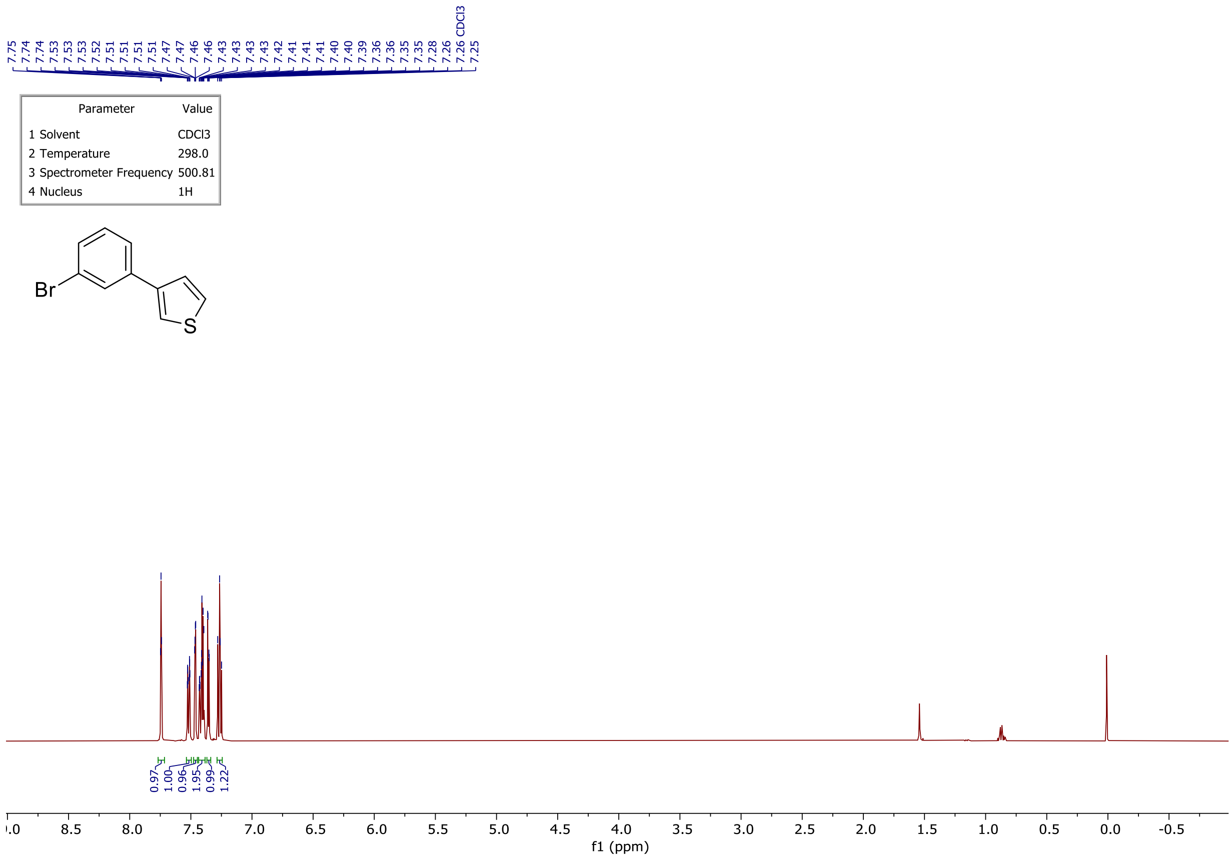

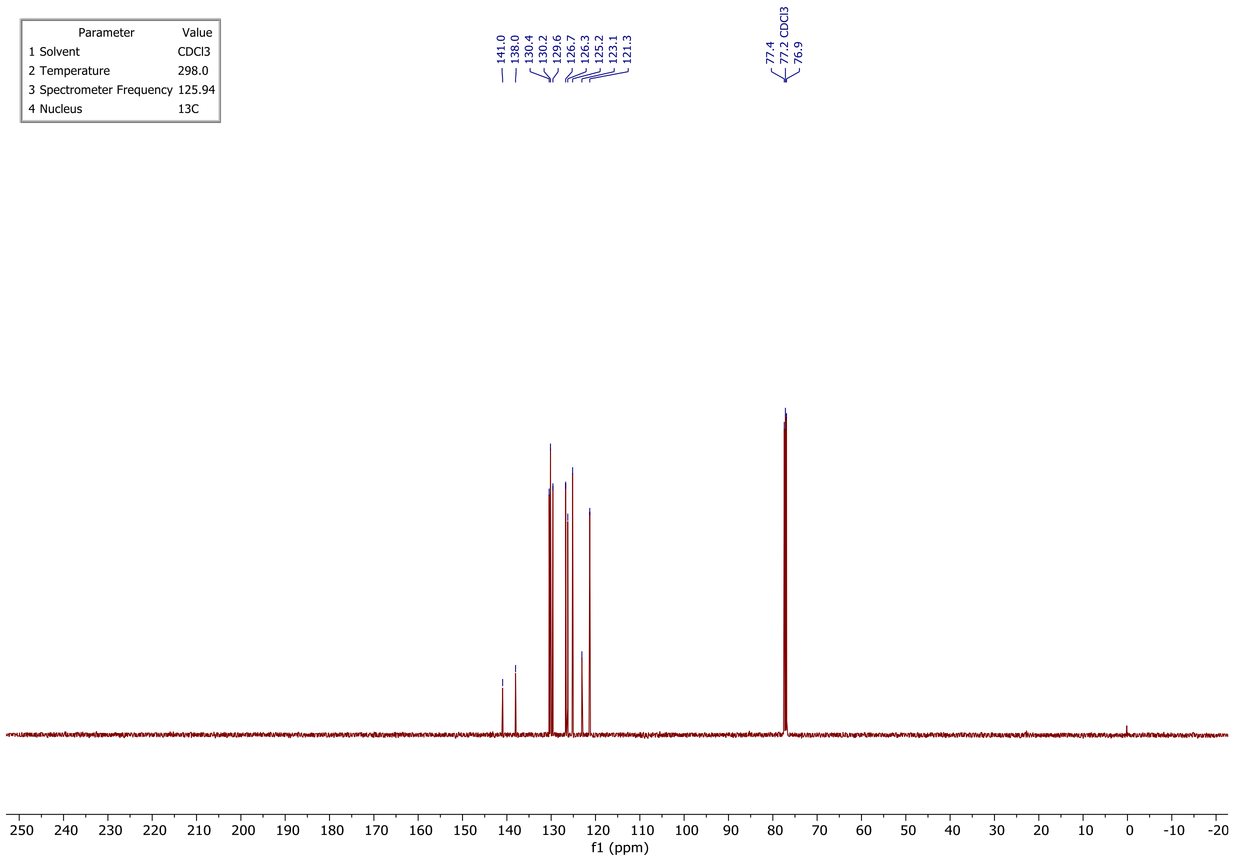

(*S*)-3,3'-((2,2'-bis(methoxymethoxy)-[1,1'-binaphthalene]-3,3'-diyl)bis(3,1-phenylene))dithiophene

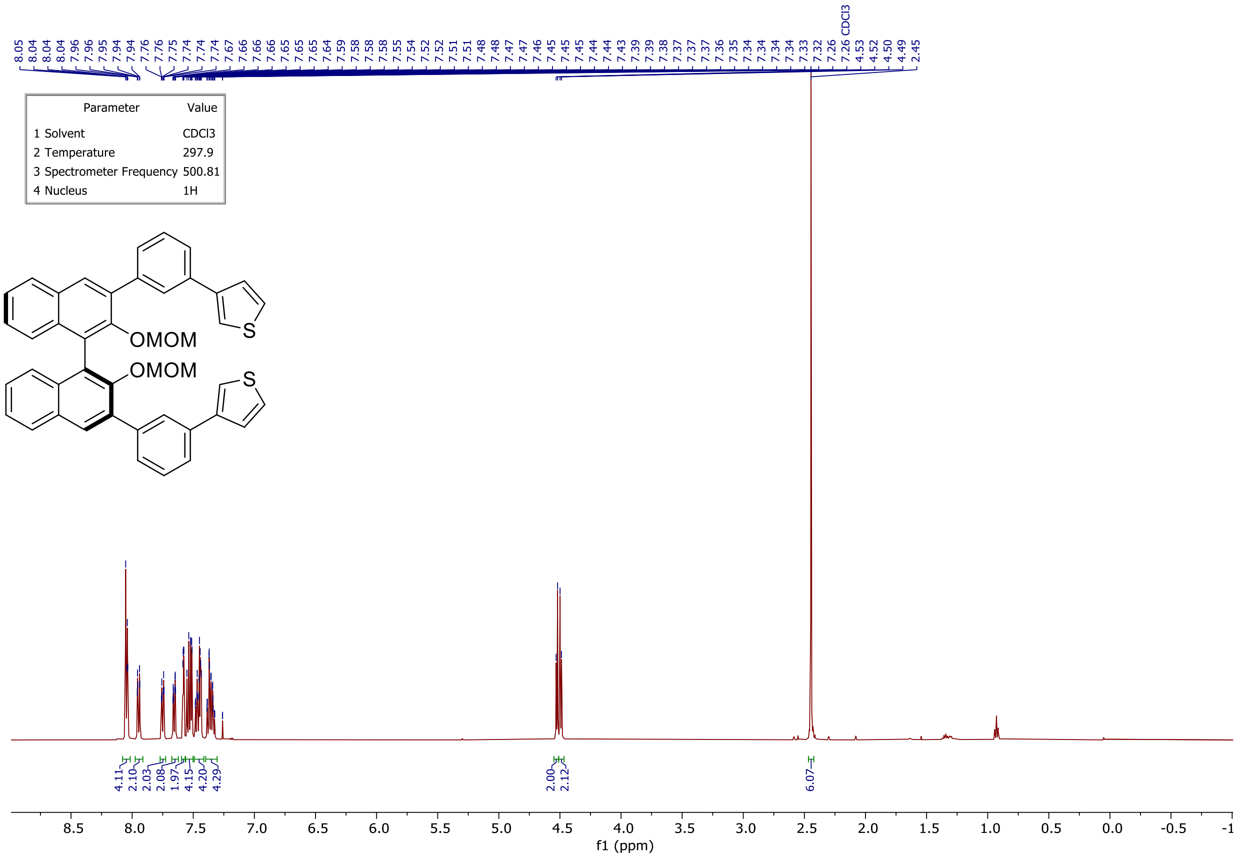

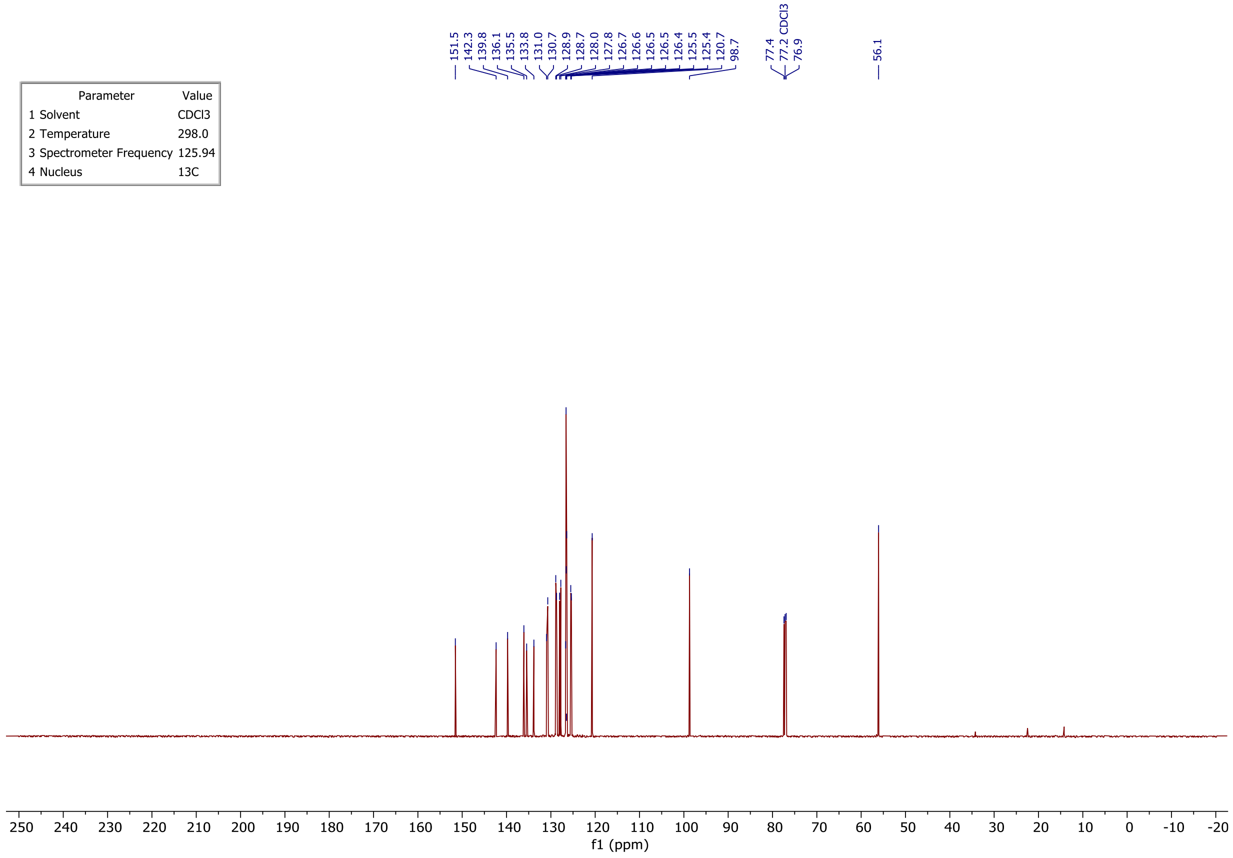

Parameter	Value
1 Solvent	CDCl3
2 Temperature	298.0
3 Spectrometer Frequency	125.94
4 Nucleus	13C

(*S*)-3,3'-bis(3-(thiophen-3-yl)phenyl)-[1,1'-binaphthalene]-2,2'-diol

Parameter	Value
1 Solvent	CDCl3
2 Temperature	297.9
3 Spectrometer Frequency	500.81
4 Nucleus	1H

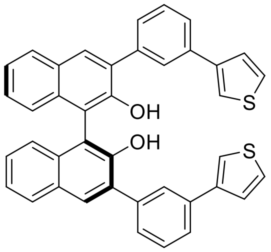

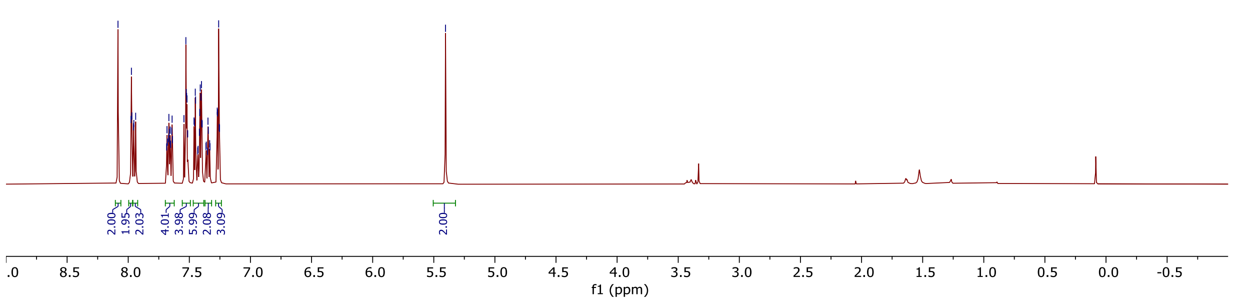

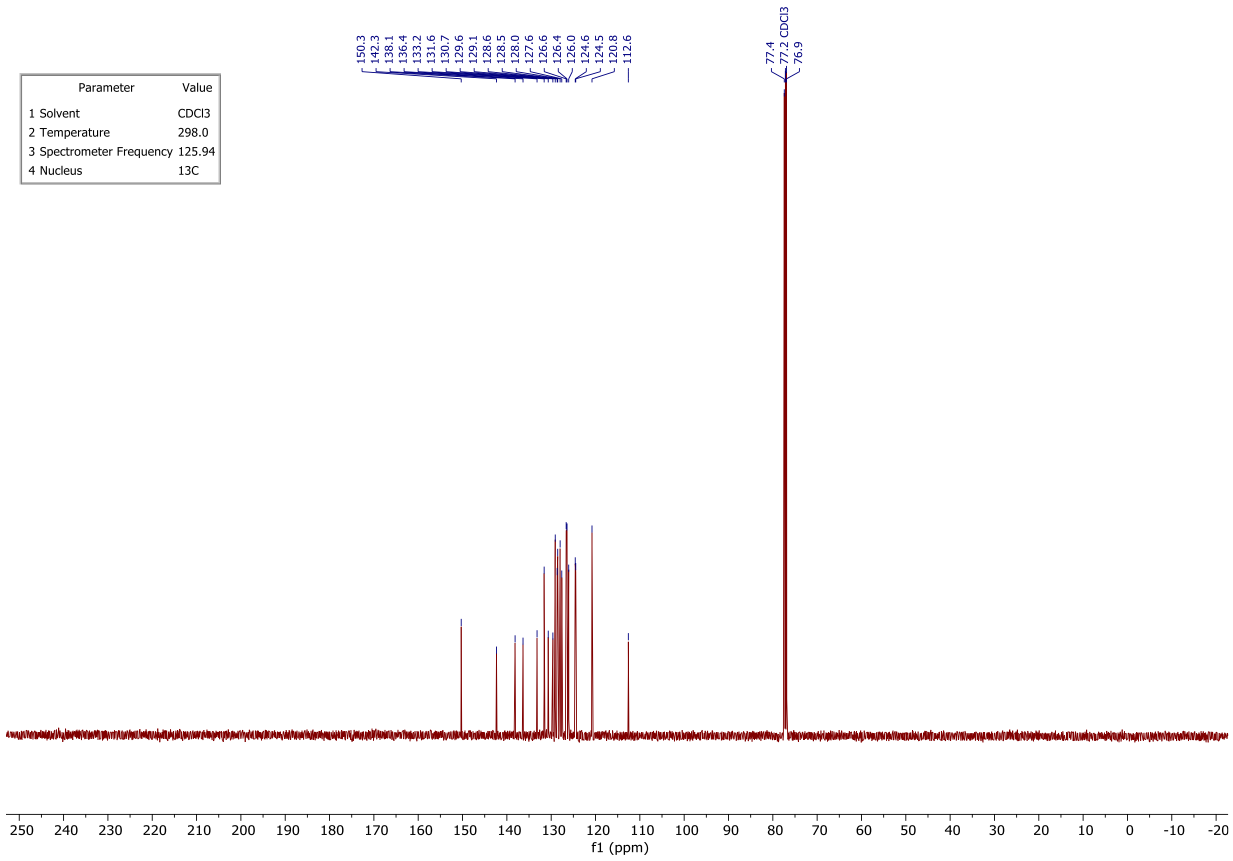

(*S*,*S*)-IDPi-**3**

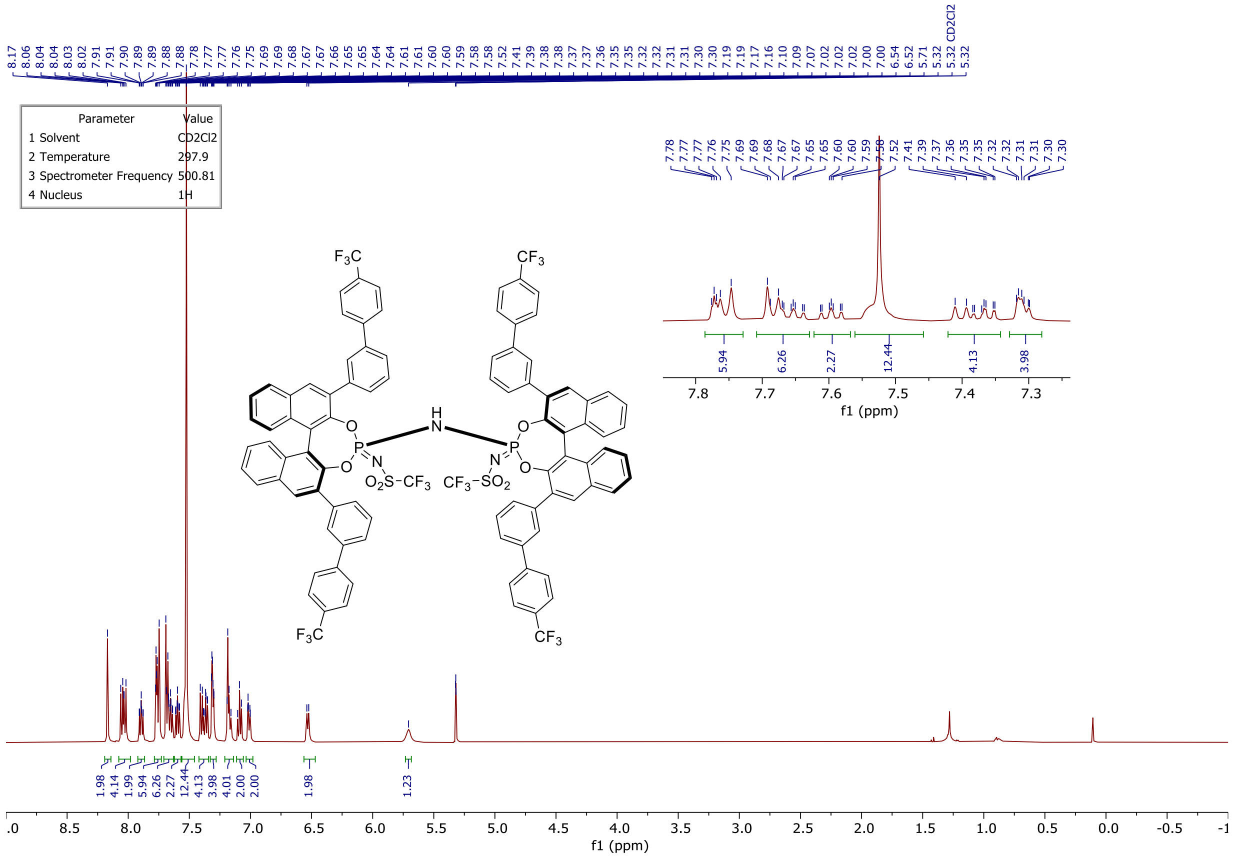

Parameter	Value
1 Solvent	CD2Cl2
2 Temperature	298.0
3 Spectrometer Frequency	125.94
4 Nucleus	13C

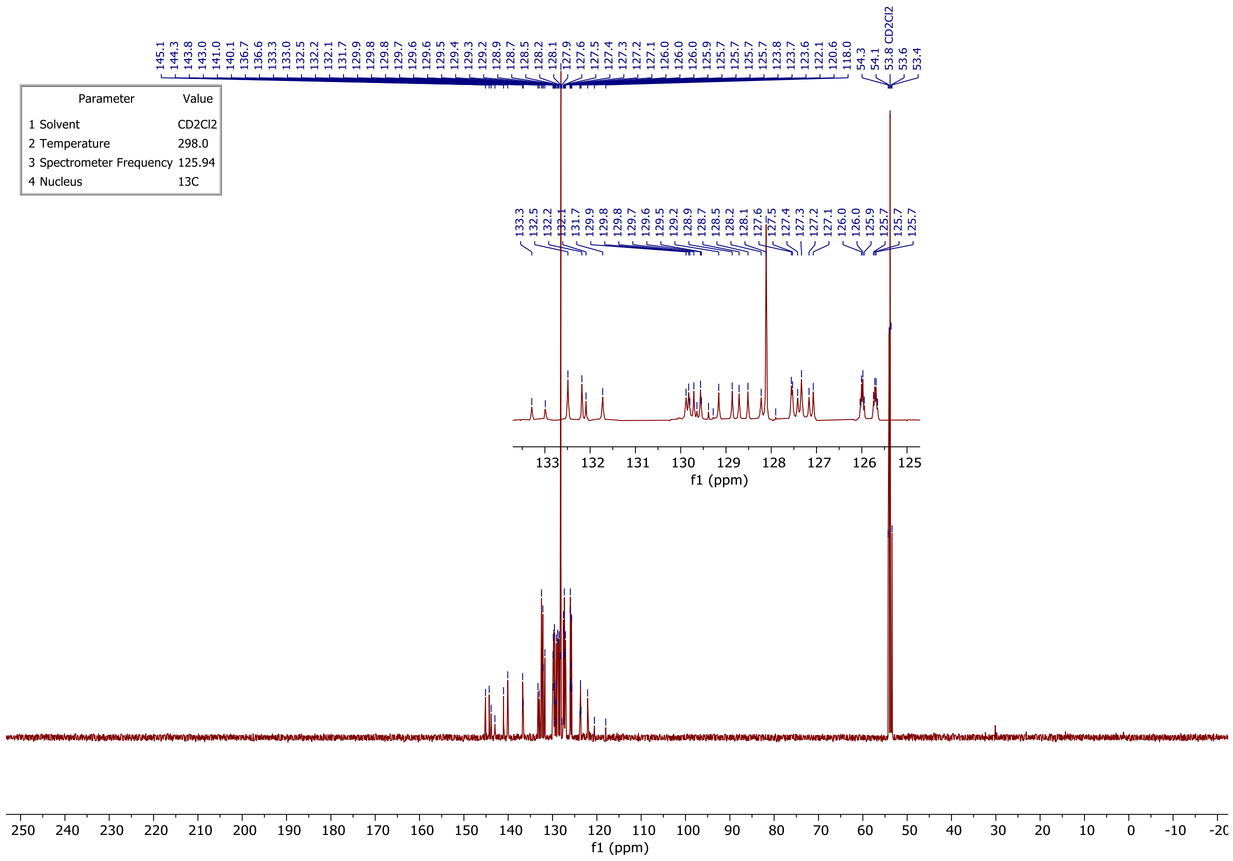

Parameter	Value
1 Solvent	CD2Cl2
2 Temperature	298.0
3 Spectrometer Frequency	471.21
4 Nucleus	19F

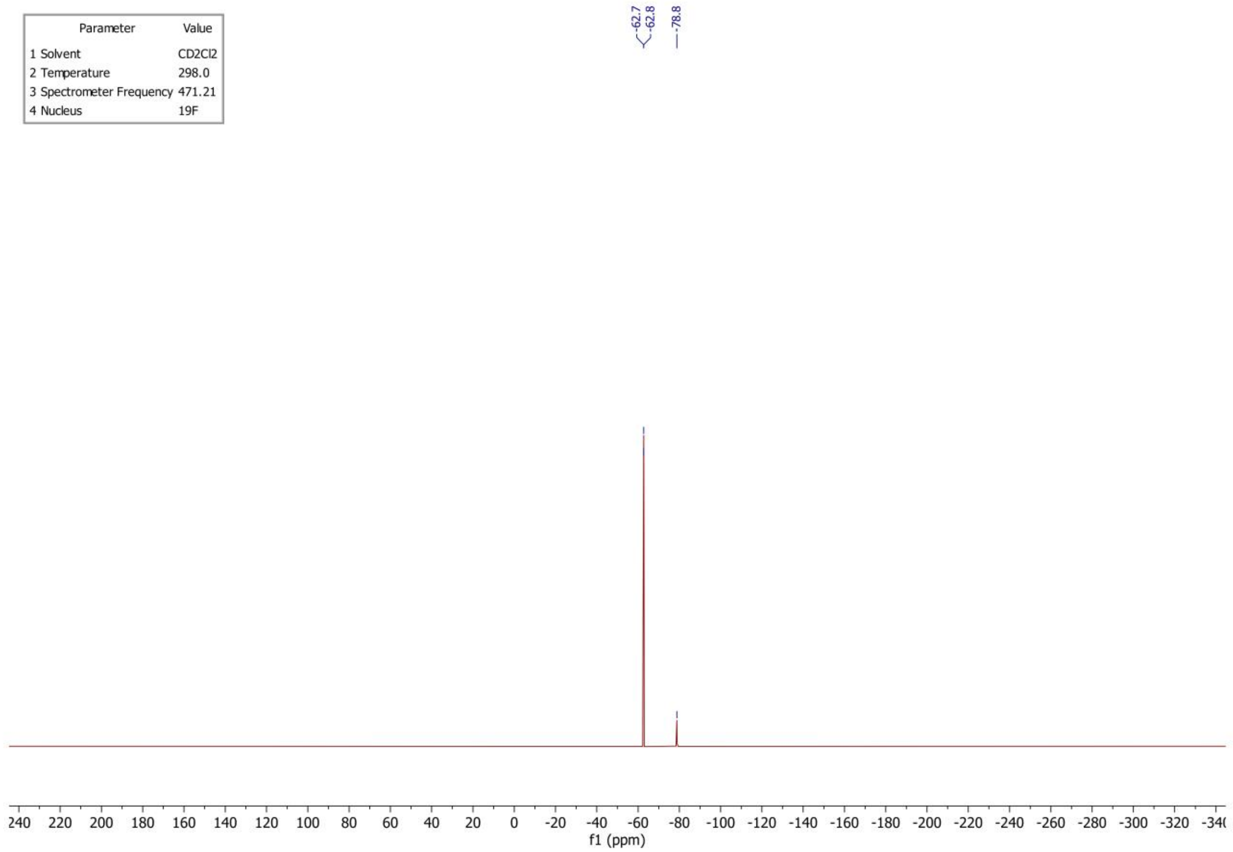

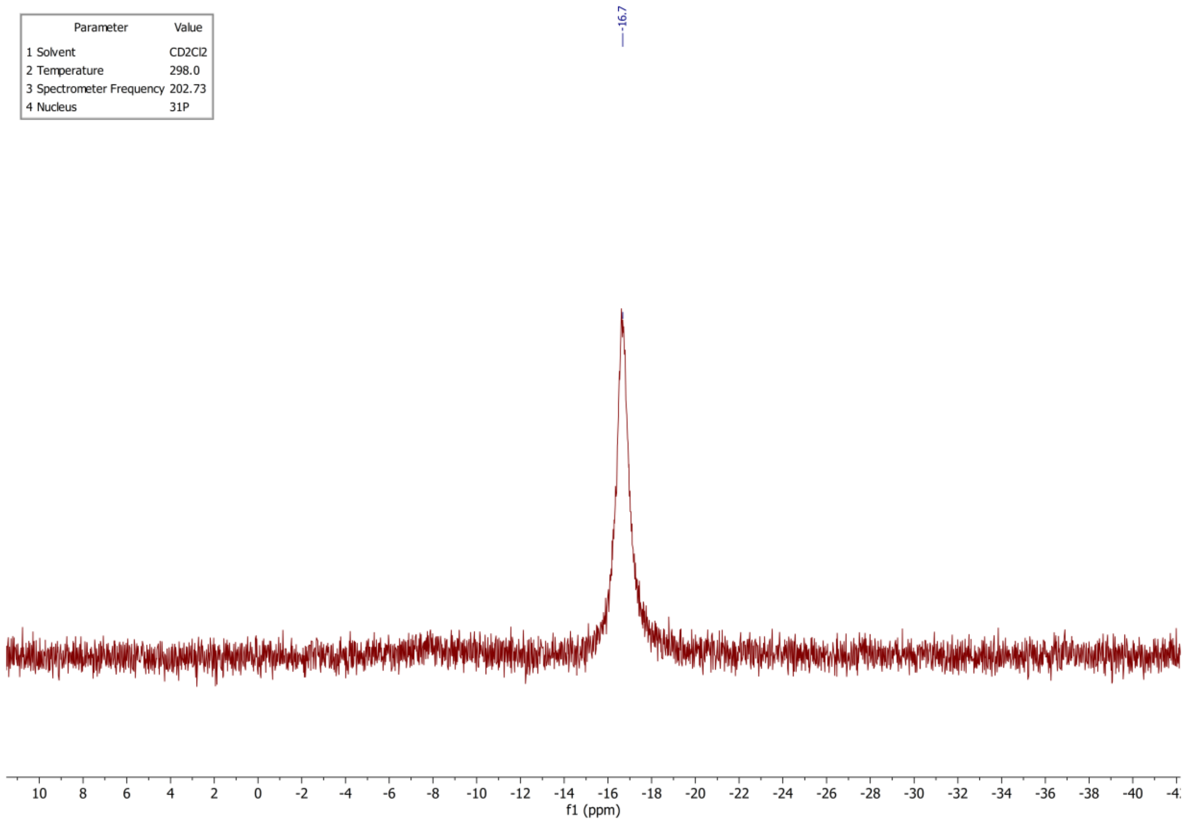

Parameter Value
1 Solvent CD2Cl2
2 Temperature 298.0
3 Spectrometer Frequency 202.73
4 Nucleus 31P
-16.7
f1 (ppm)

(*S*,*S*)-IDPi-**4**

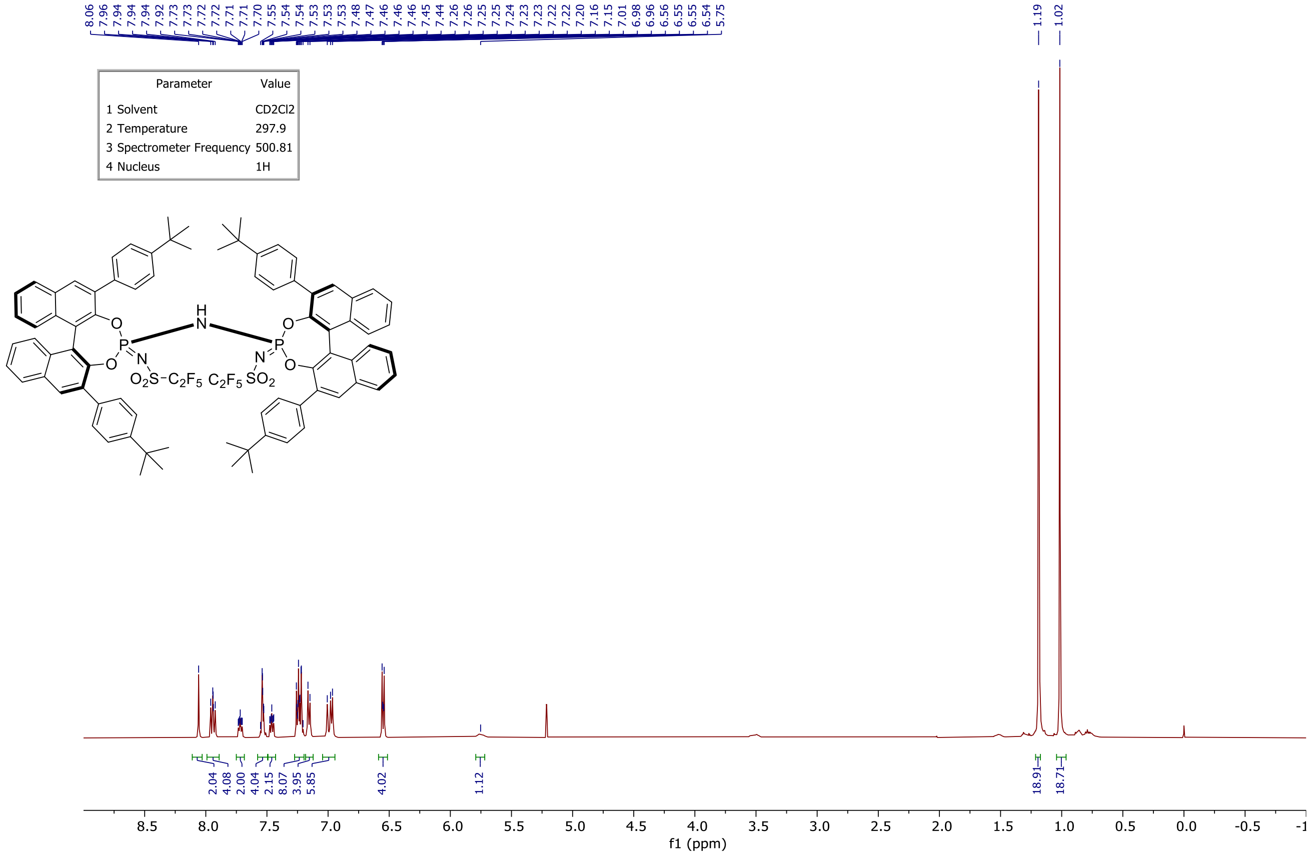

8.06 7.96 7.94 7.94 7.92 7.73 7.72 7.72 7.71 7.70 7.55 7.54 7.53 7.53 7.53 7.48 7.47 7.46 7.46 7.45 7.44 7.26 7.25 7.24 7.23 7.23 7.22 7.22 7.20 7.16 7.15 7.01 6.98 6.96 6.56 6.55 6.55 6.54 5.75
1.19
1.02
Parameter Value
1 Solvent CD2Cl2
2 Temperature 297.9
3 Spectrometer Frequency 500.81
4 Nucleus 1H
2.04 4.08 2.00 4.04 2.15 8.07 3.95 5.85 4.02 1.12 18.91 18.71
f1 (ppm)

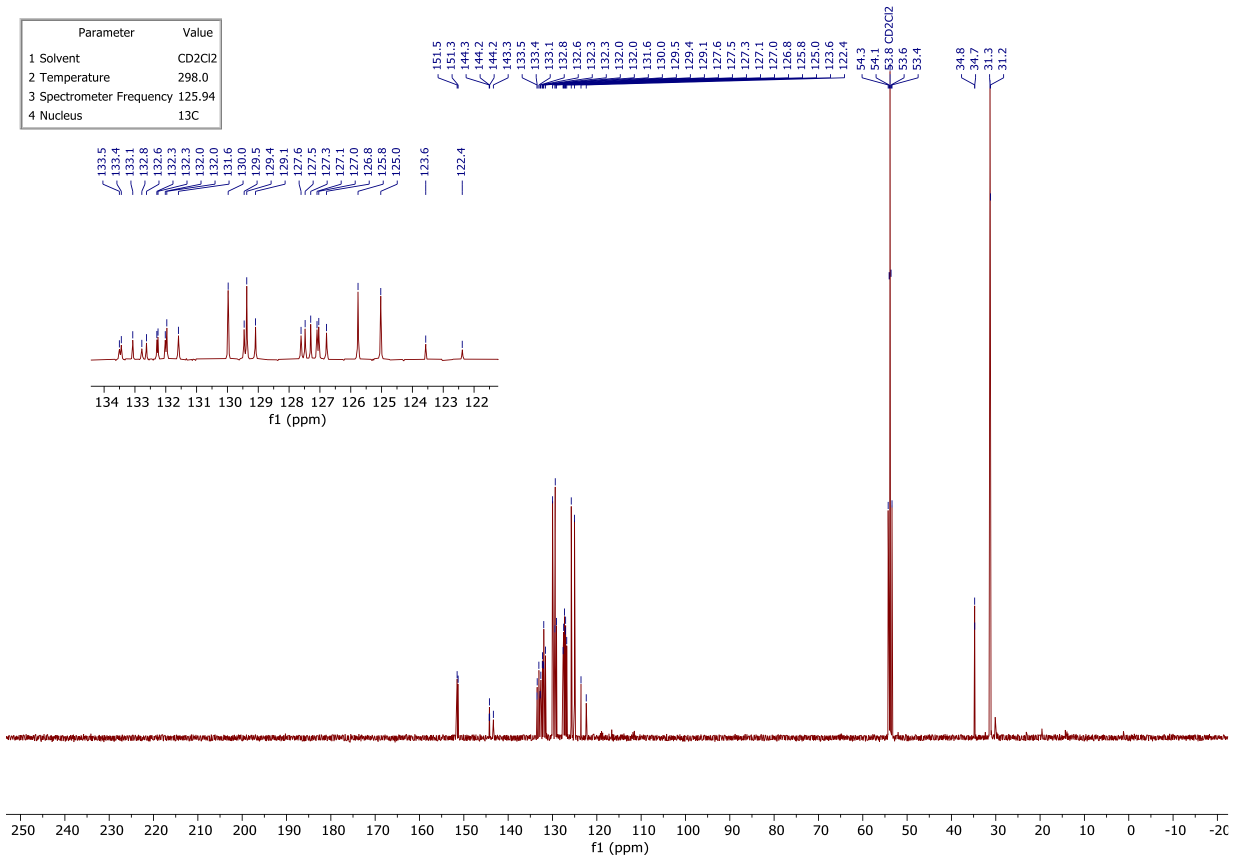
Parameter Value
1 Solvent CD2Cl2
2 Temperature 298.0
3 Spectrometer Frequency 125.94
4 Nucleus 13C
151.5
151.3
144.3
144.2
144.2
143.3
133.5
133.4
133.1
132.8
132.6
132.3
132.3
132.0
132.0
131.6
130.0
129.5
129.4
129.1
127.6
127.5
127.3
127.1
127.0
126.8
125.8
125.0
123.6
122.4
54.3
54.1
53.8 CD2Cl2
53.6
53.4
34.8
34.7
31.3
31.2
f1 (ppm)

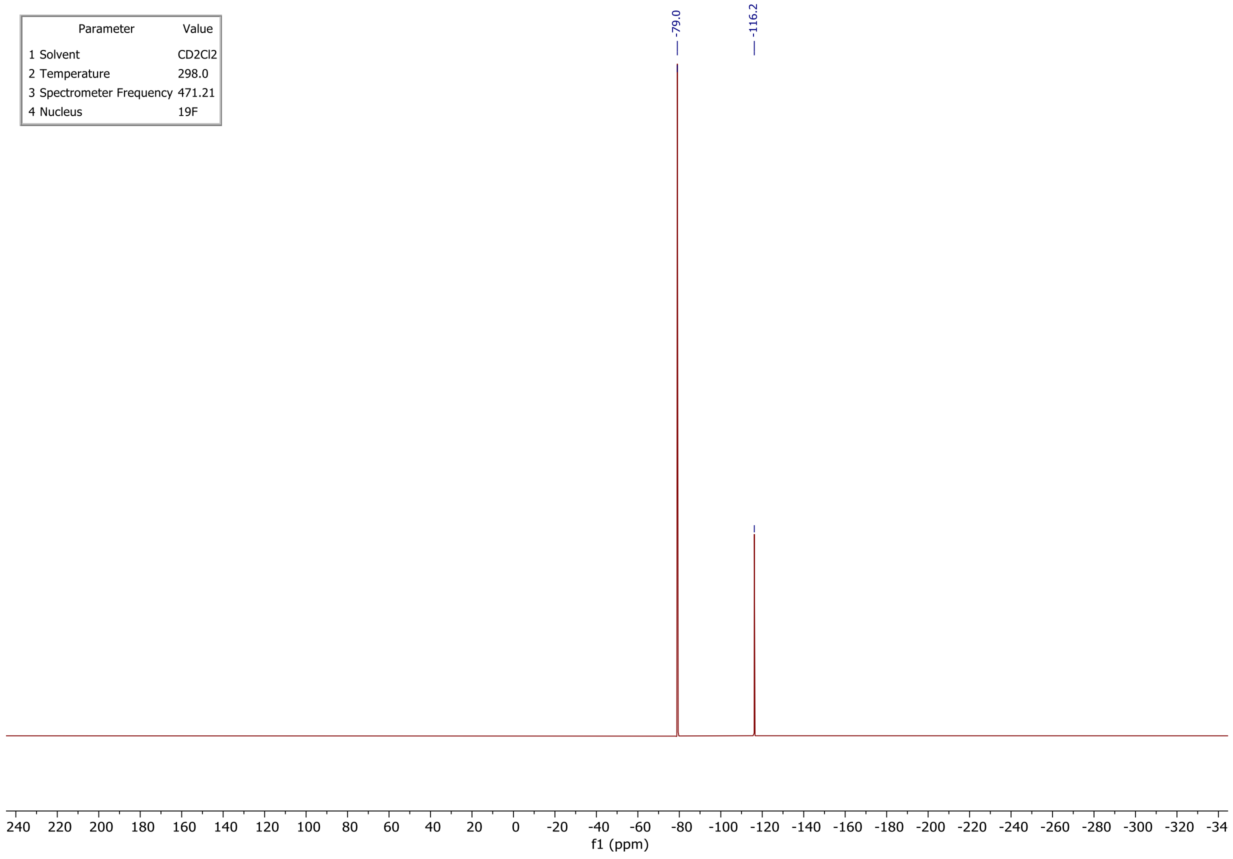
Parameter Value
1 Solvent CD2Cl2
2 Temperature 298.0
3 Spectrometer Frequency 471.21
4 Nucleus 19F
-79.0
-116.2
f1 (ppm)

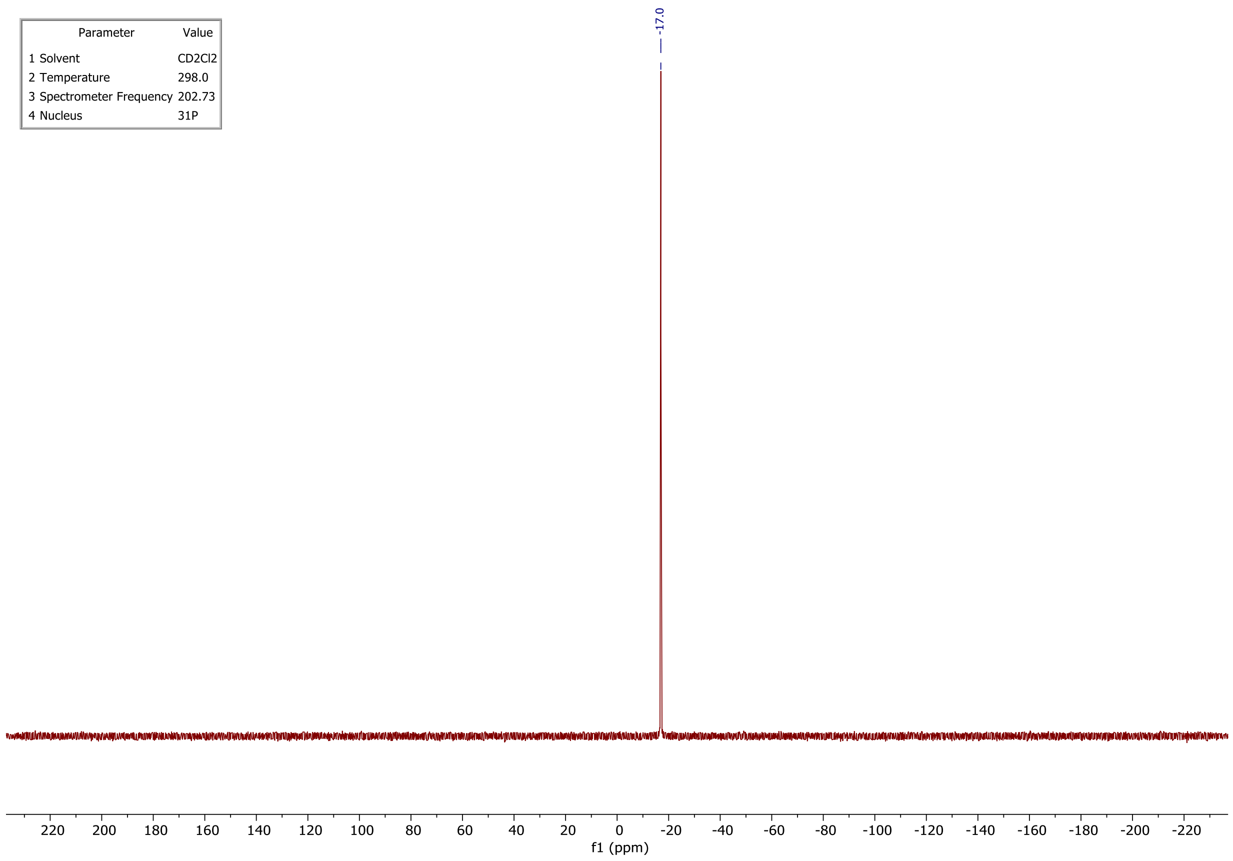

Parameter Value
1 Solvent CD2Cl2
2 Temperature 298.0
3 Spectrometer Frequency 202.73
4 Nucleus 31P
-17.0
f1 (ppm)

(*S*,*S*)-IDPi-**5**

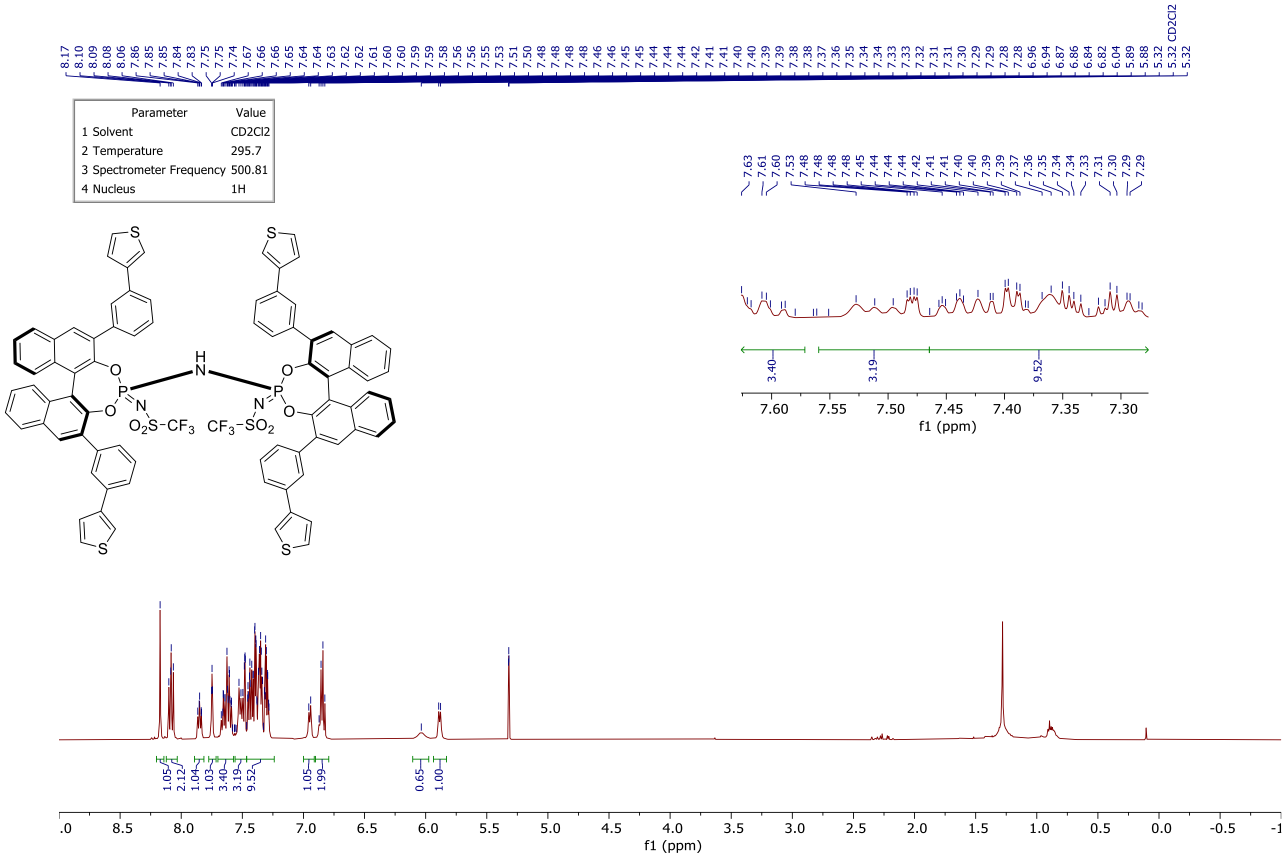

8.17 8.10 8.09 8.08 8.06 7.86 7.85 7.85 7.84 7.83 7.75 7.75 7.74 7.67 7.66 7.66 7.65 7.64 7.64 7.63 7.62 7.62 7.61 7.60 7.59 7.59 7.58 7.56 7.56 7.55 7.53 7.53 7.51 7.50 7.48 7.48 7.48 7.46 7.46 7.45 7.45 7.44 7.44 7.44 7.42 7.41 7.41 7.40 7.40 7.39 7.39 7.38 7.38 7.37 7.36 7.35 7.34 7.34 7.33 7.33 7.32 7.31 7.31 7.30 7.29 7.29 7.28 7.28 6.96 6.94 6.87 6.86 6.84 6.82 6.04 5.89 5.88 5.32 5.32 CD2Cl2 5.32
Parameter Value
1 Solvent CD2Cl2
2 Temperature 295.7
3 Spectrometer Frequency 500.81
4 Nucleus 1H
H
N
P
O
S
CF3
O2S
SO2
3.40
3.19
9.52
f1 (ppm)
1.05 2.12 1.04 1.03 3.40 3.19 9.52 1.05 1.99 0.65 1.00
f1 (ppm)

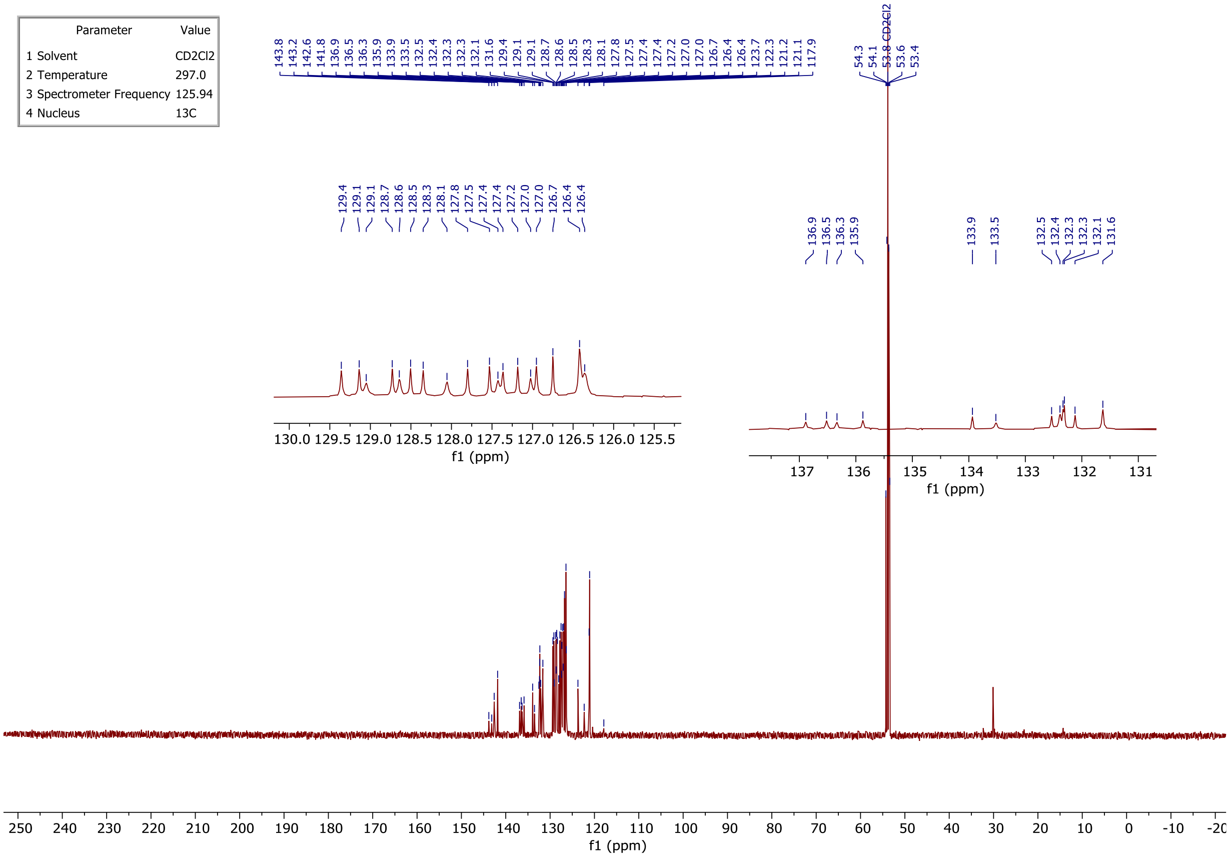
Parameter Value
1 Solvent CD2Cl2
2 Temperature 297.0
3 Spectrometer Frequency 125.94
4 Nucleus 13C
143.8 143.2 142.6 141.8 136.9 136.5 136.3 135.9 133.9 133.5 132.5 132.4 132.3 132.3 132.1 131.6 129.4 129.1 129.1 128.7 128.6 128.5 128.3 128.1 127.8 127.5 127.4 127.4 127.2 127.0 126.7 126.4 126.4 123.7 122.3 121.2 121.1 117.9
54.3 54.1 53.8 CD2Cl2 53.6 53.4
129.4 129.1 129.1 128.7 128.6 128.5 128.3 128.1 127.8 127.5 127.4 127.4 127.2 127.0 127.0 126.7 126.4 126.4
136.9 136.5 136.3 135.9 133.9 133.5 132.5 132.4 132.3 132.3 132.1 131.6
f1 (ppm)

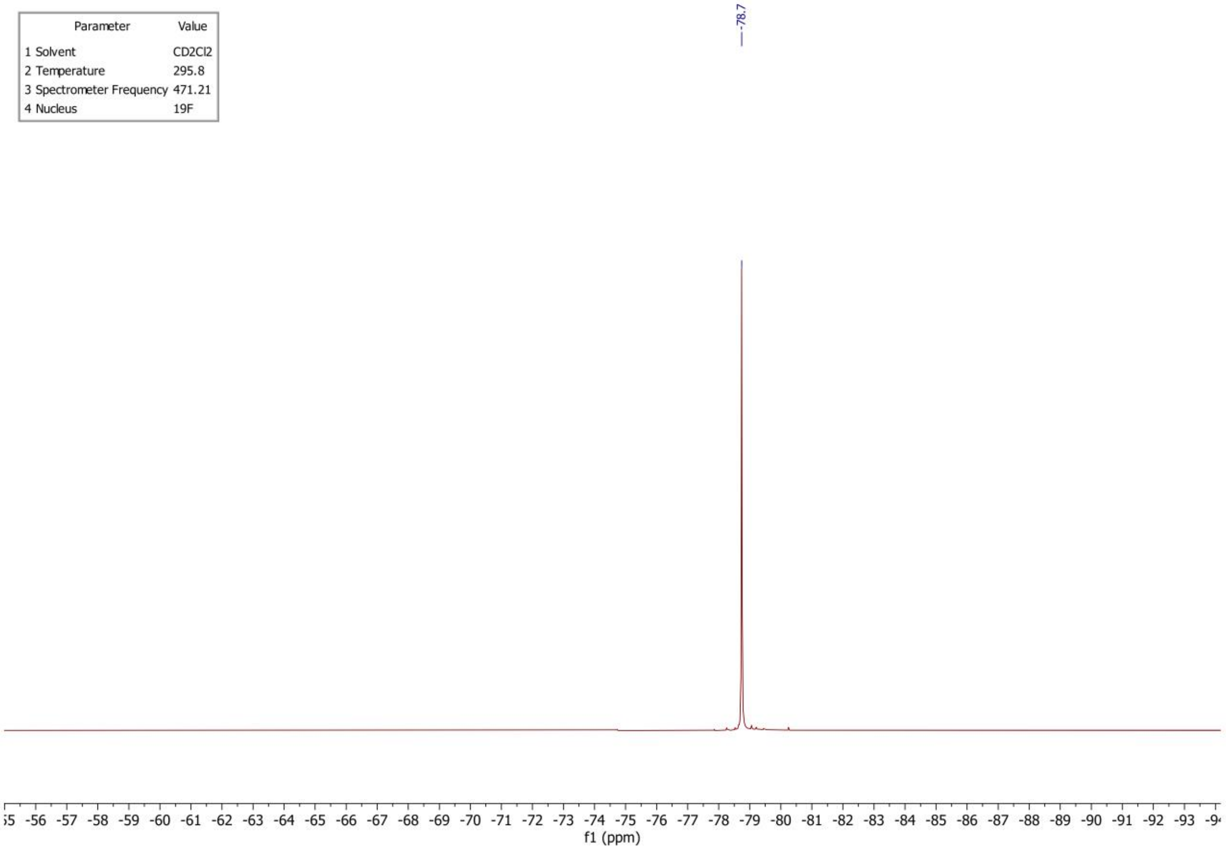
Parameter Value
1 Solvent CD2Cl2
2 Temperature 295.8
3 Spectrometer Frequency 471.21
4 Nucleus 19F
-78.7
f1 (ppm)

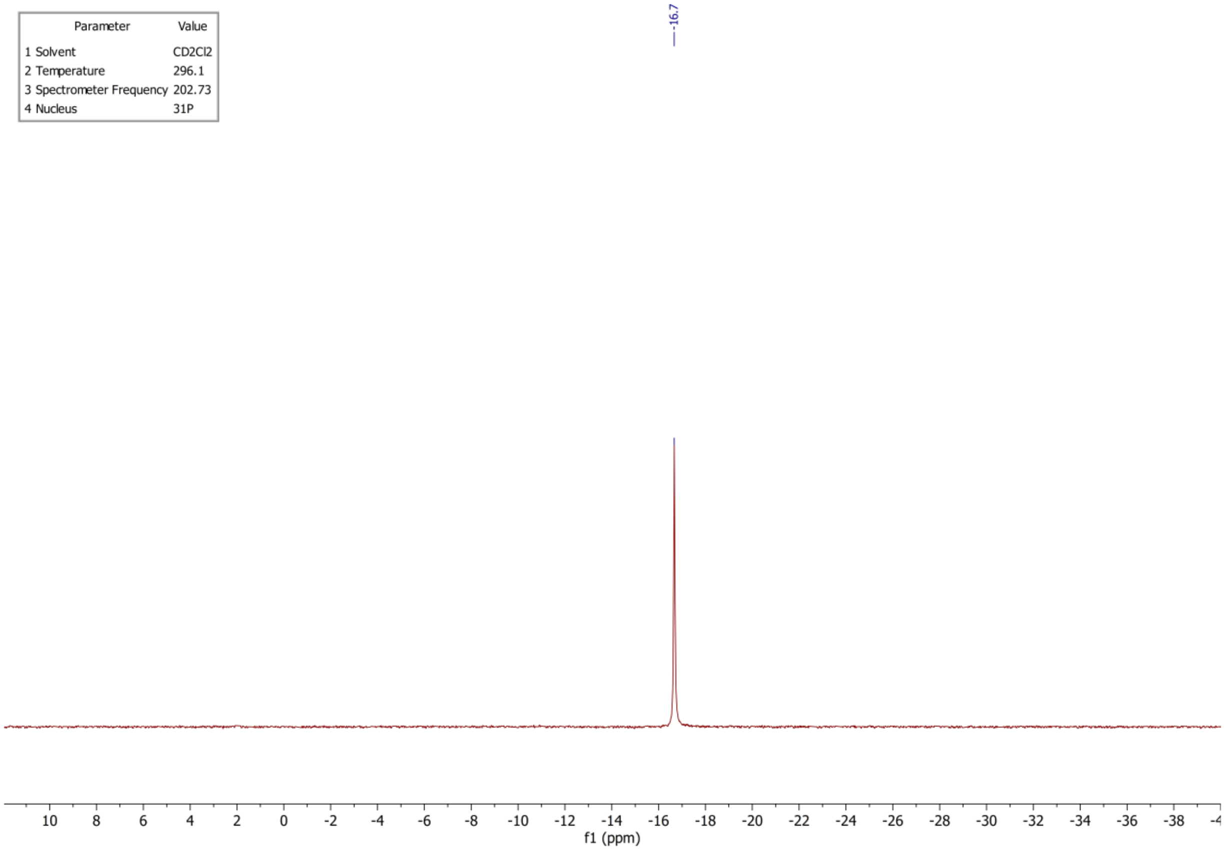
Parameter Value
1 Solvent CD2Cl2
2 Temperature 296.1
3 Spectrometer Frequency 202.73
4 Nucleus 31P
-16.7
10 8 6 4 2 0 -2 -4 -6 -8 -10 -12 -14 -16 -18 -20 -22 -24 -26 -28 -30 -32 -34 -36 -38
f1 (ppm)

13. Copies of HPLC and GC traces

GC (30.0 m BGB 176, injection temperature: 220 °C, 40 °C iso 35 min, 0.6 bar H_2).

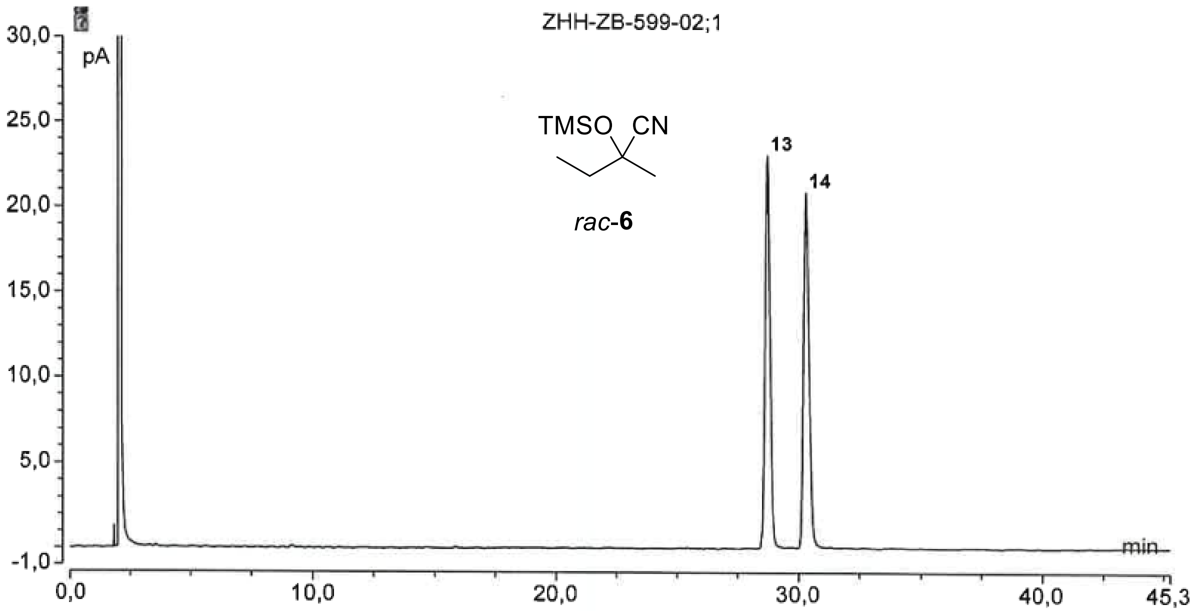

Peak #	t_R/min	% peak area
1	28.7	50.00
2	30.3	50.00
Total		100

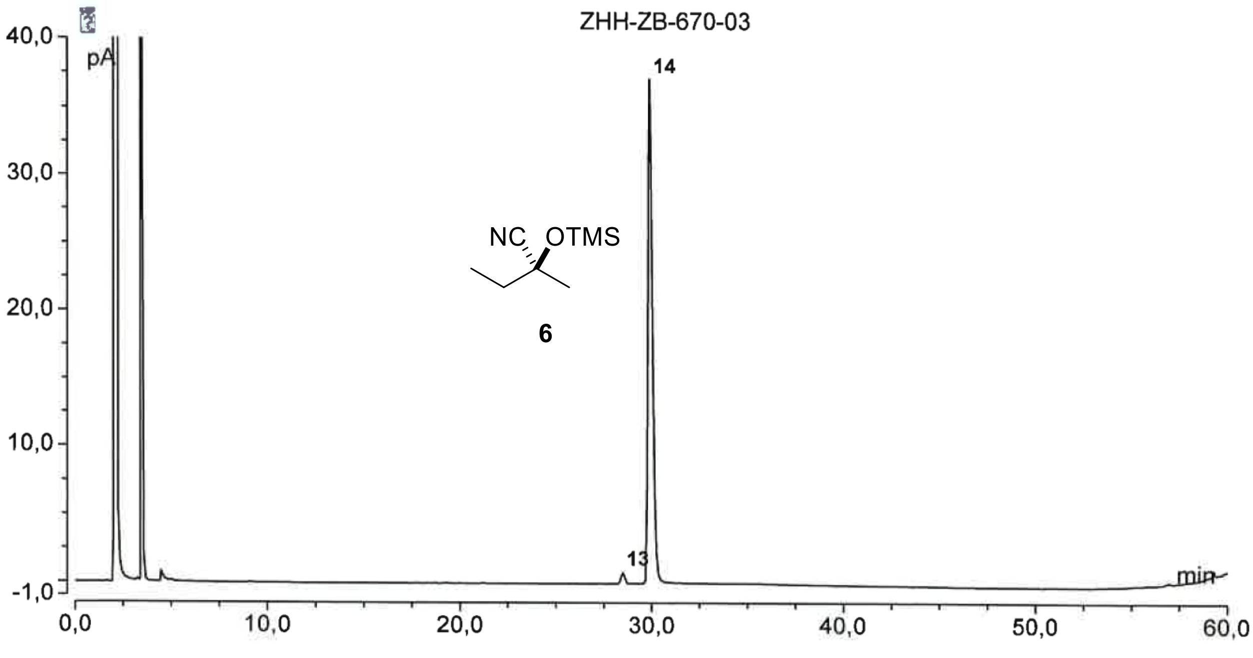

Peak #	t_R/min	% peak area
1	28.5	1.91
2	29.9	98.09
Total		100

GC spectrum of product from gram-scale synthesis, the e.r. = 95:5:

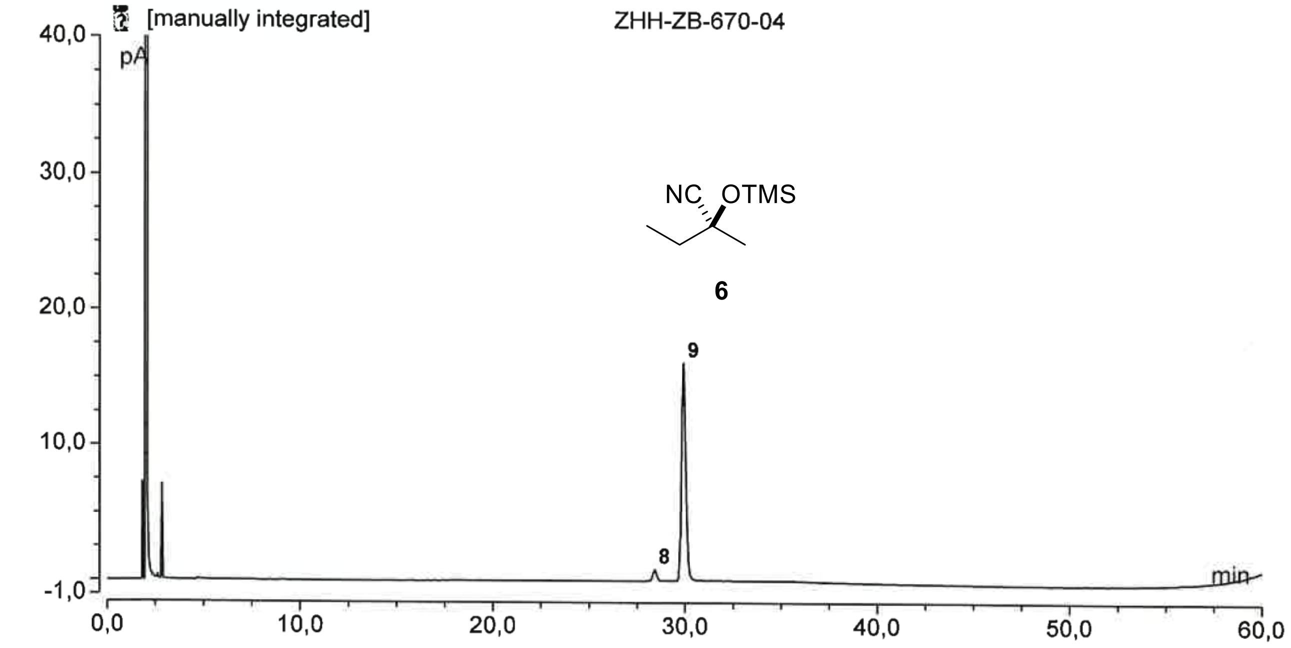

Peak #	t_R/min	% peak area
1	28.4	4.55
2	29.9	95.45
Total		100

GC (30.0 m BGB 176, injection temperature: 220 °C, 60 °C iso 60 min, 0.5 bar H_2)

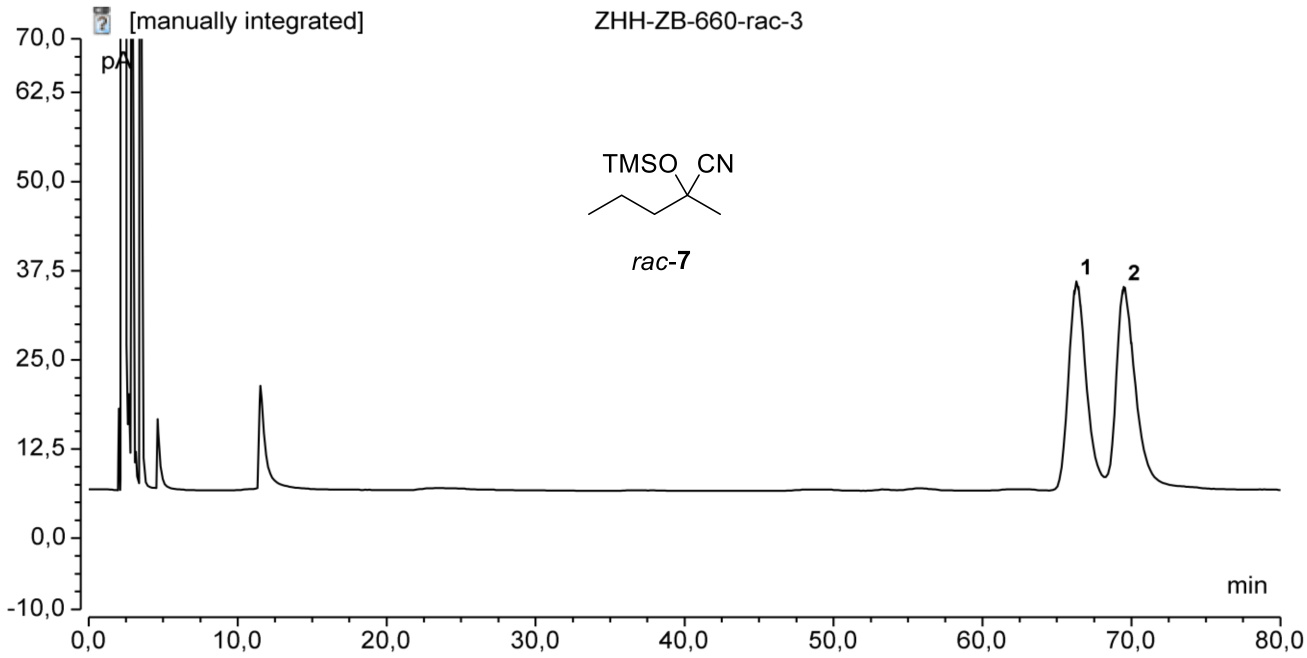

Peak #	t_R/min	% peak area
1	66.3	49.24
2	69.5	50.76
Total		100

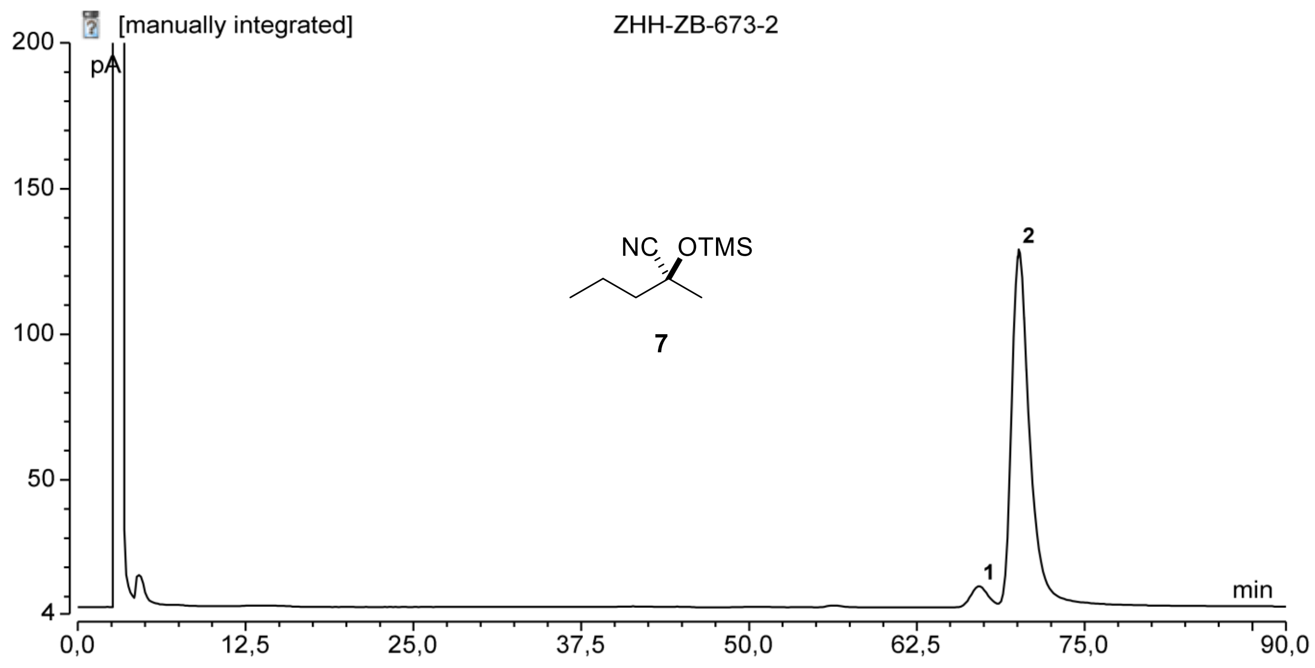

Peak #	t_R/min	% peak area
1	67.2	5.11
2	70.1	94.89
Total		100

GC (30.0 m BGB 176, injection temperature: 220 °C, 80 °C iso 140 min, 0.5 bar H_2)

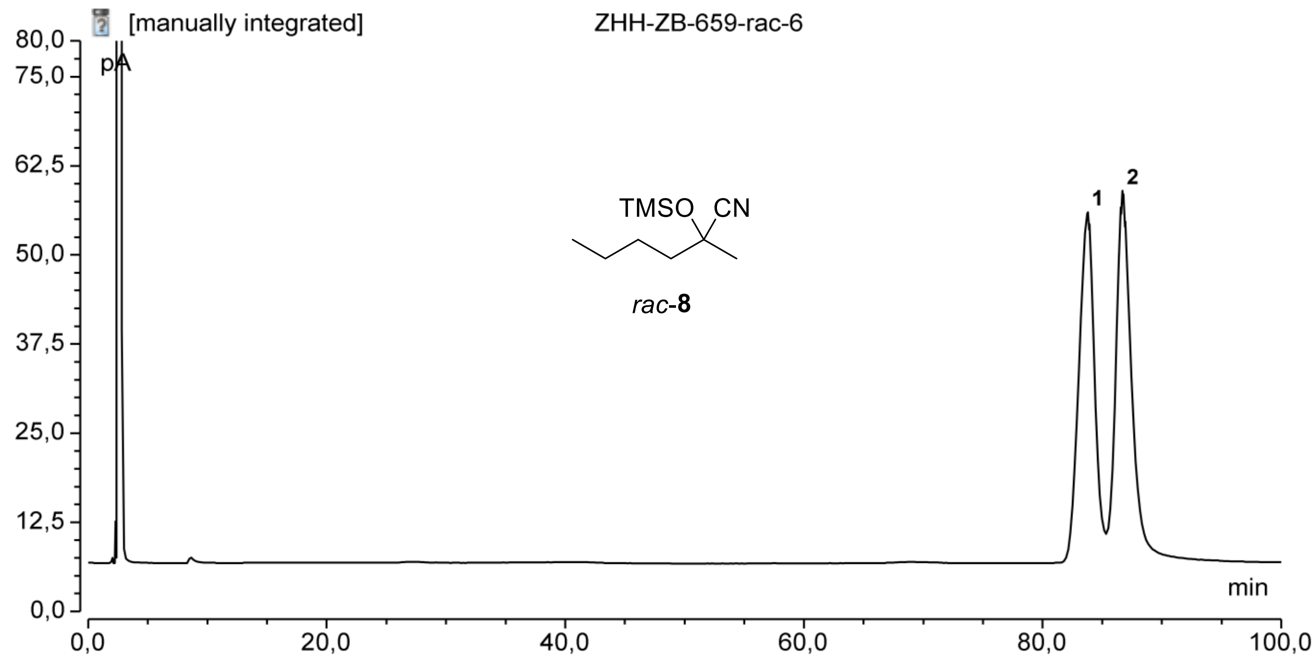

Peak #	t_R/min	% peak area
1	83.8	50.56
2	86.7	49.44
Total		100

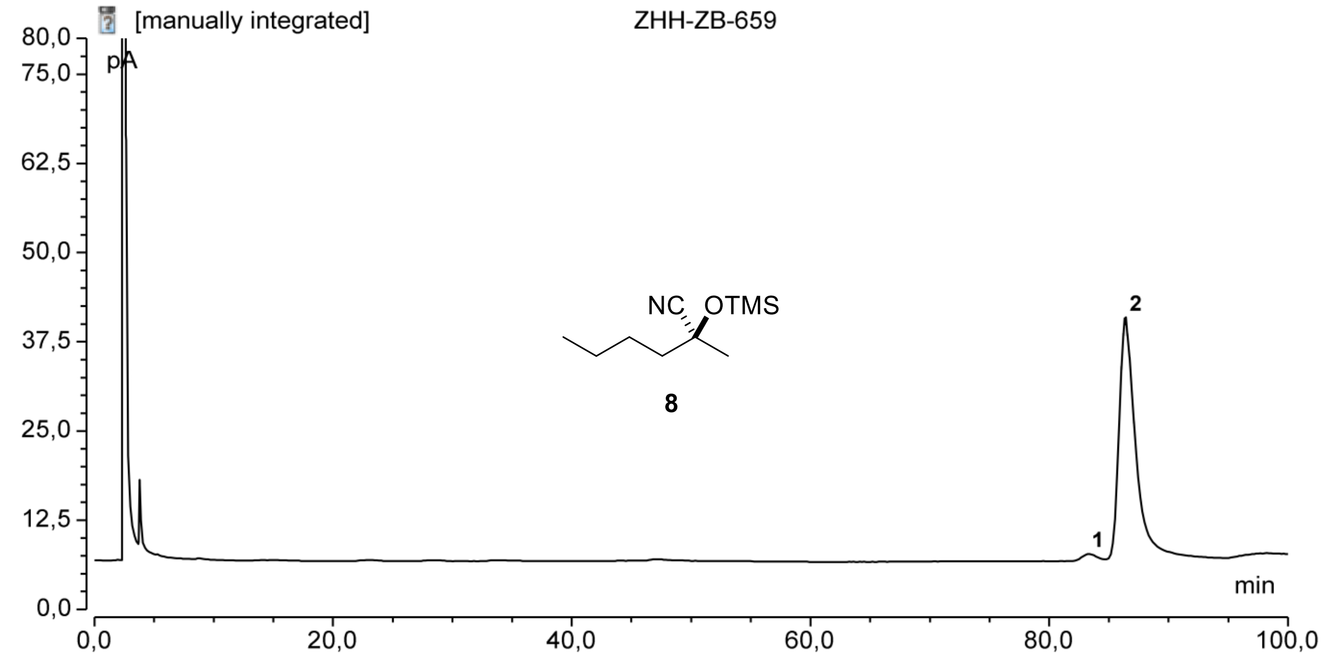

Peak #	t_R/min	% peak area
1	83.3	1.73
2	86.4	98.27
Total		100

GC (30.0 m BGB 176, injection temperature: 220 °C, 80 °C iso 80 min, 0.5 bar H_2)

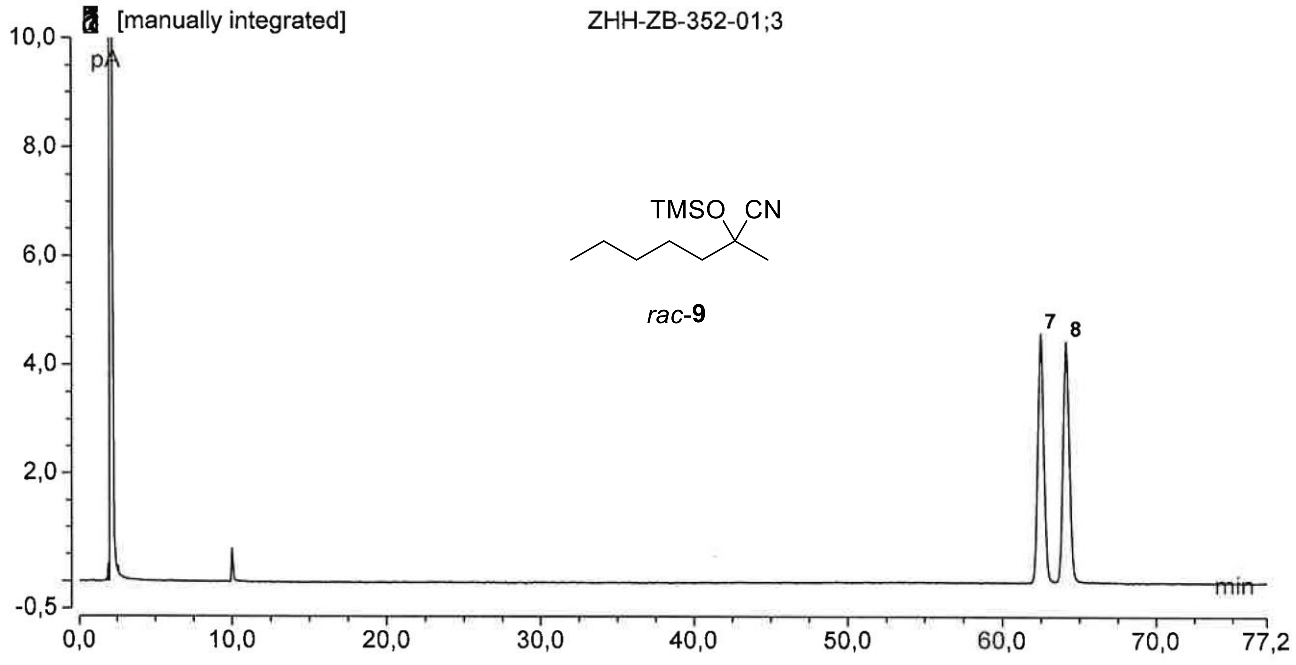

Peak #	t_R/min	% peak area
1	62.5	50.00
2	64.2	50.00
Total		100

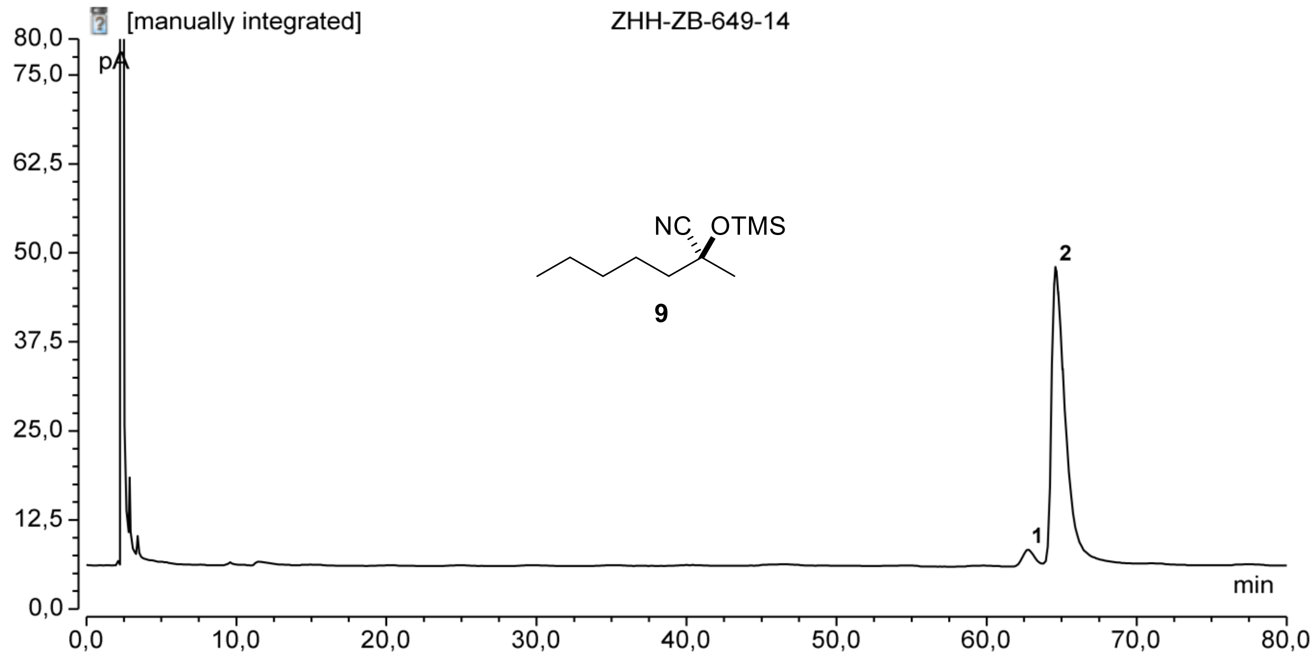

Peak #	t_R/min	% peak area
1	62.7	4.46
2	64.6	95.54
Total		100

GC (30.0 m G-TA, injection temperature: 220 °C, 65 °C iso 20 min, 6 °C/min, 180 °C iso 3 min, 0.5 bar H_2)

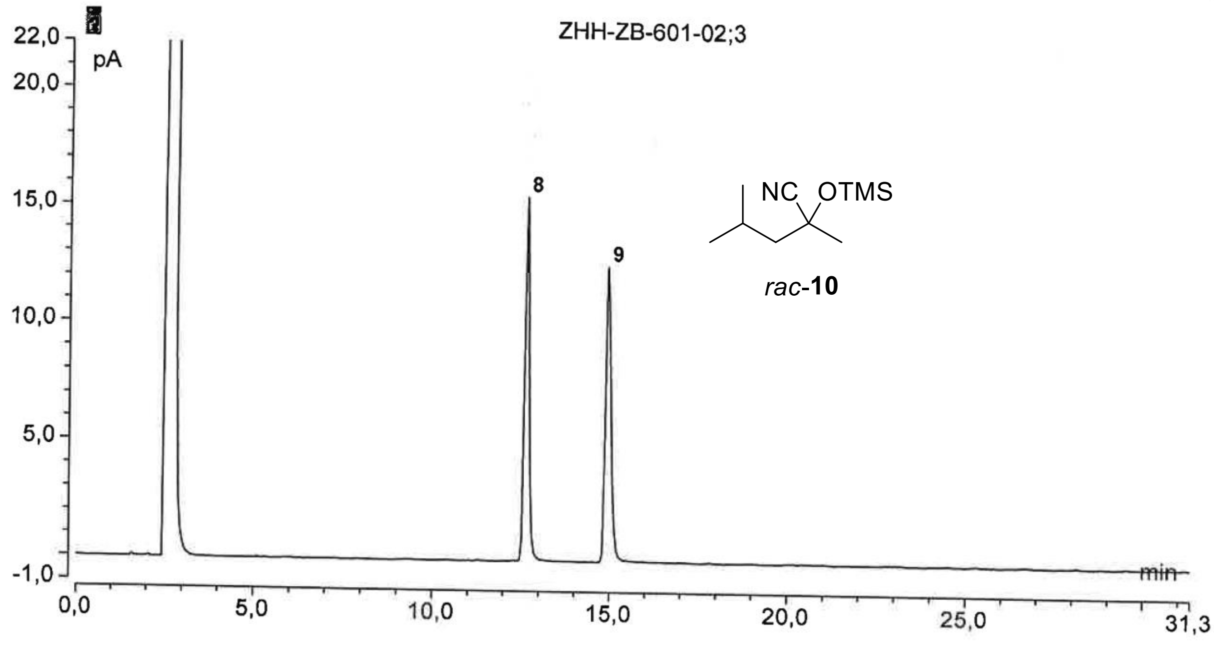

Peak #	t_R/min	% peak area
1	12.6	50.00
2	14.9	50.00
Total		100

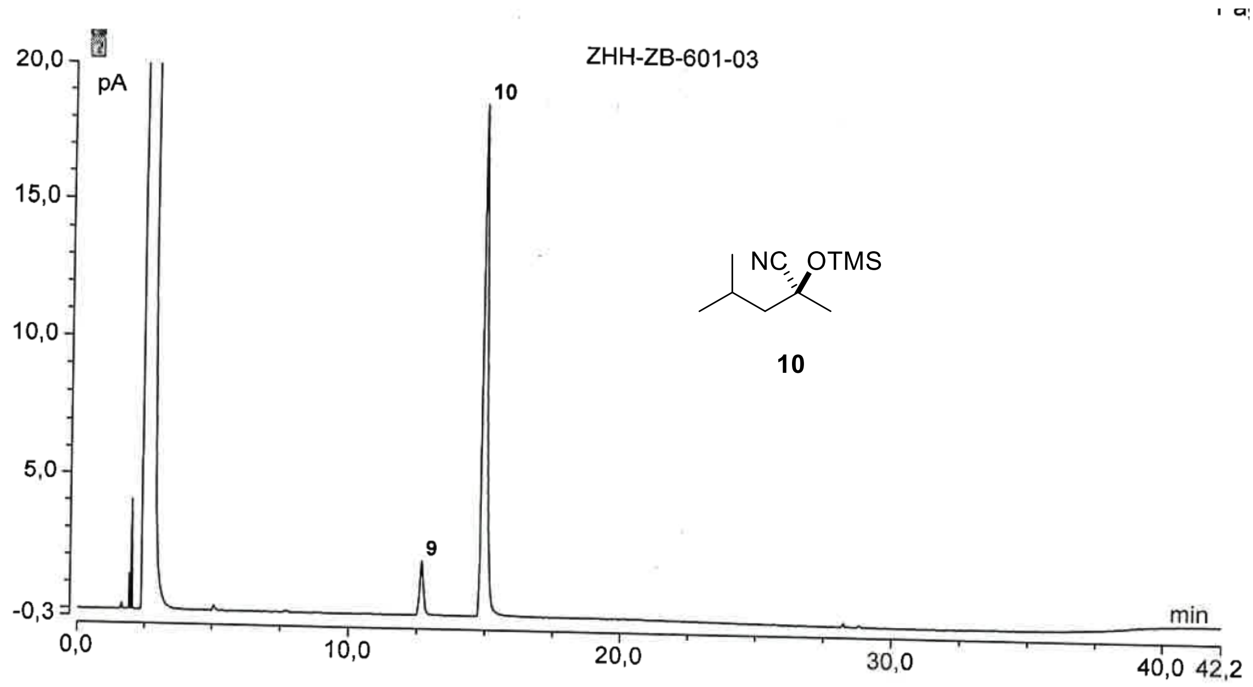

Peak #	t_R/min	% peak area
1	12.7	7.47
2	14.9	92.53
Total		100

GC (25.0 m Hydrodex-gamma TBDAc-CD, injection temperature: 220 °C, 65 °C iso 40 min, 8 °C/min, 230 °C iso 3 min, 0.5 bar H_2)

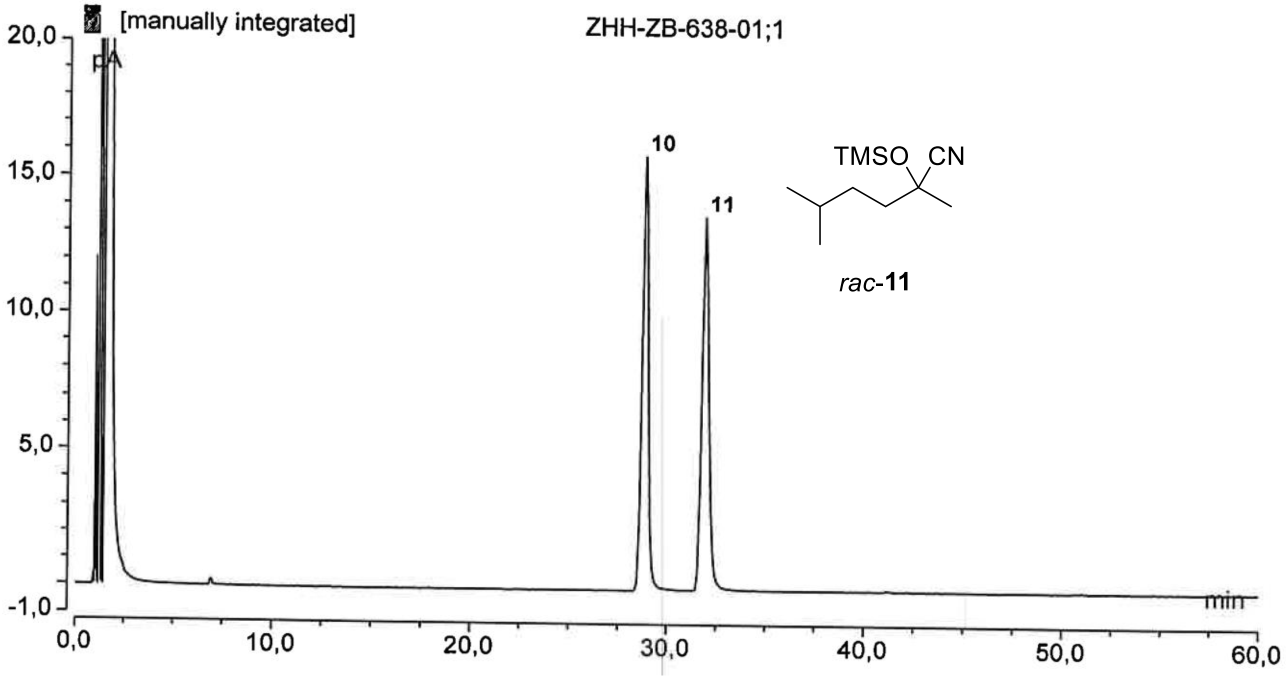

Peak #	t_R/min	% peak area
1	28.8	49.93
2	31.9	50.07
Total		100

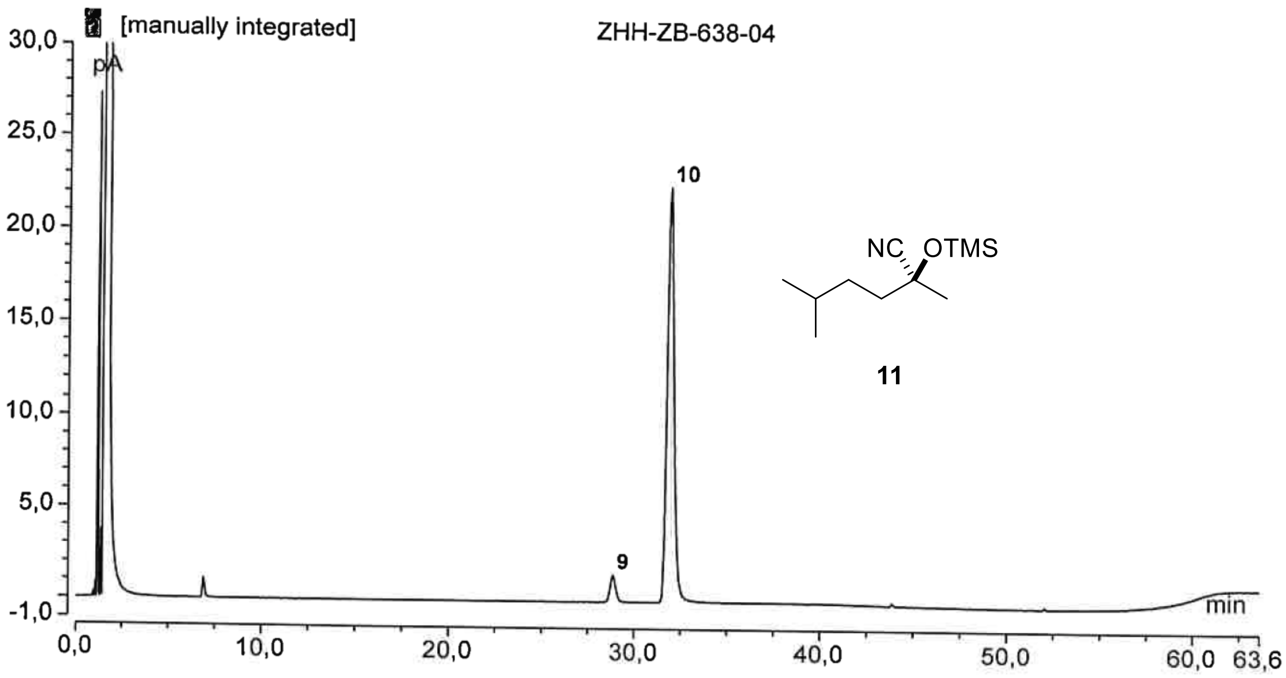

Peak #	t_R/min	% peak area
1	28.9	5.01
2	31.9	94.99
Total		100

GC (30.0 m BGB 176, injection temperature: 220 °C, 90 °C iso 100 min, 6 °C/min, 230 °C iso 3 min, 0.5 bar H_2)

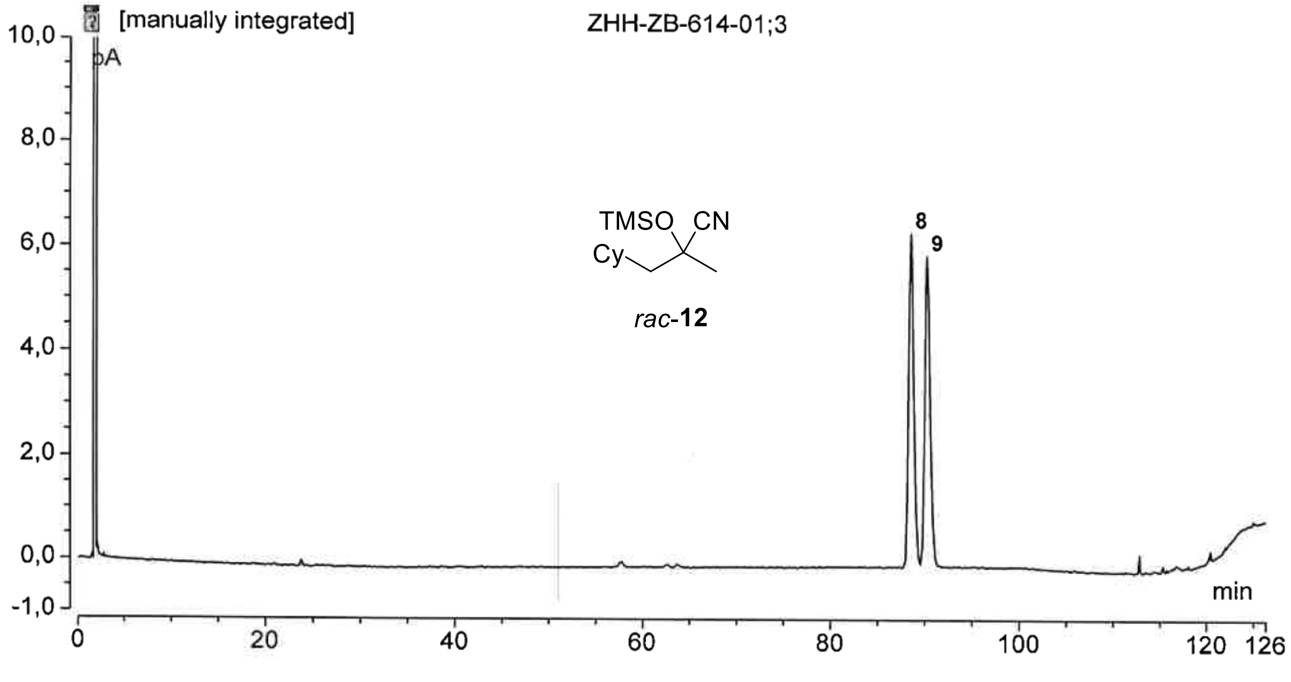

Peak #	t_R/min	% peak area
1	88.6	49.95
2	90.3	50.05
Total		100

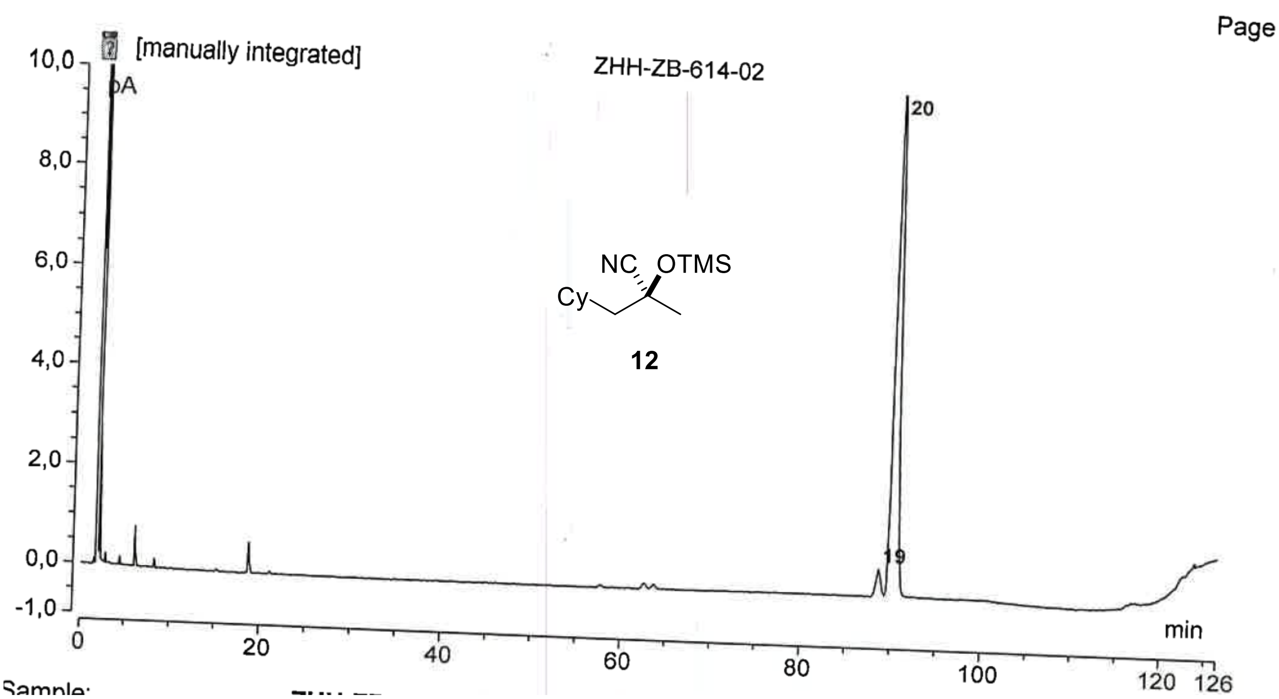

Peak #	t_R/min	% peak area
1	88.7	4.30
2	90.1	95.70
Total		100

GC (30.0 m G-TA, injection temperature: 220 °C, 75 °C iso 80 min, 0.5 bar H_2)

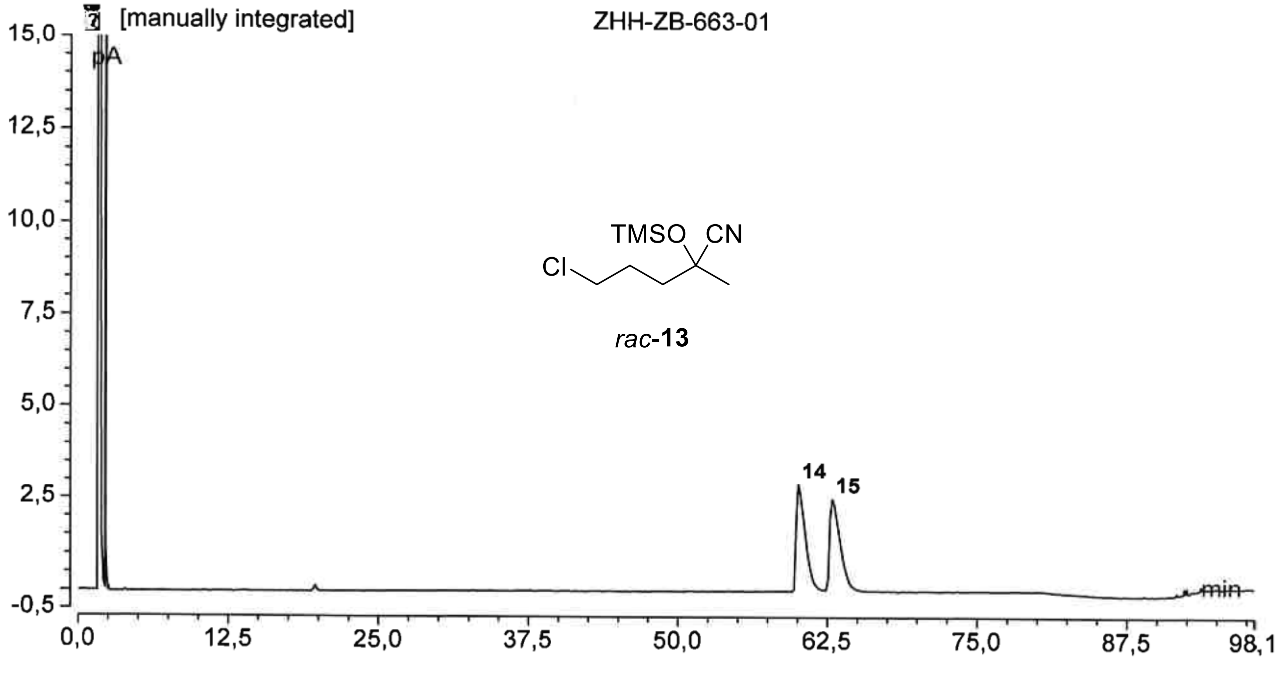

Peak #	t_R/min	% peak area
1	60.0	49.62
2	62.9	50.38
Total		100

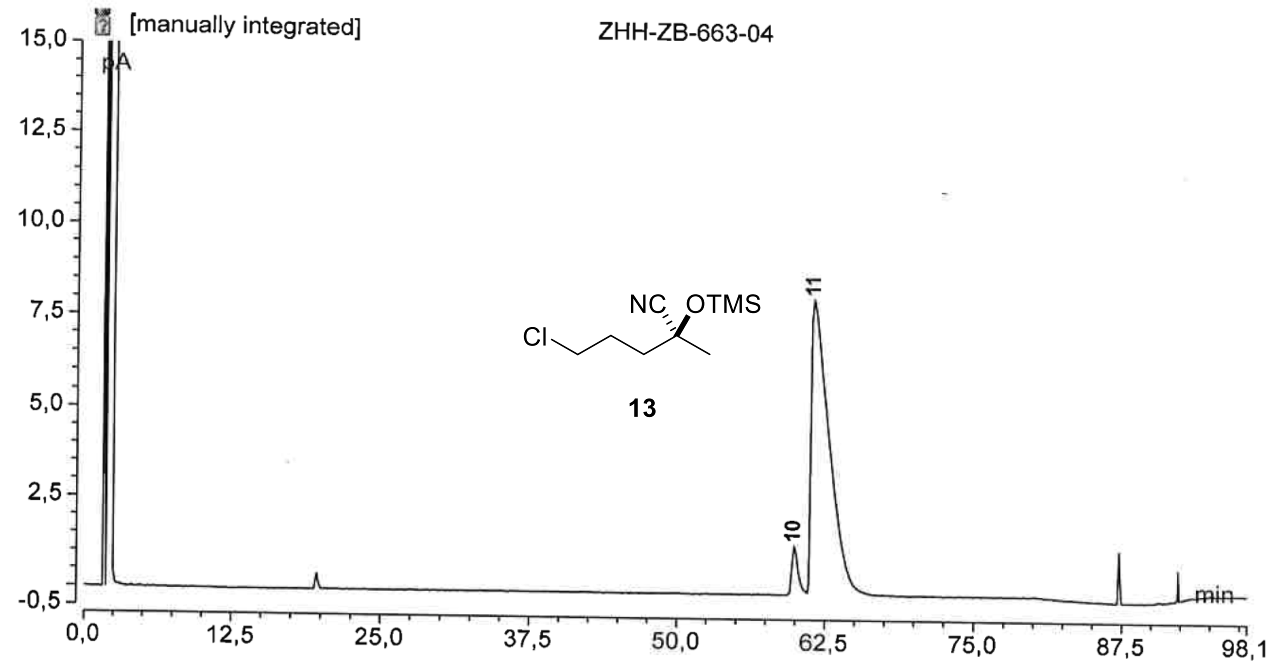

Peak #	t_R/min	% peak area
1	60.0	5.36
2	61.5	94.64
Total		100

GC (30.0 m G-TA, injection temperature: 220 °C, 100 °C iso 30 min, 8 °C/min, 180 °C iso 3 min, 0.5 bar H_2)

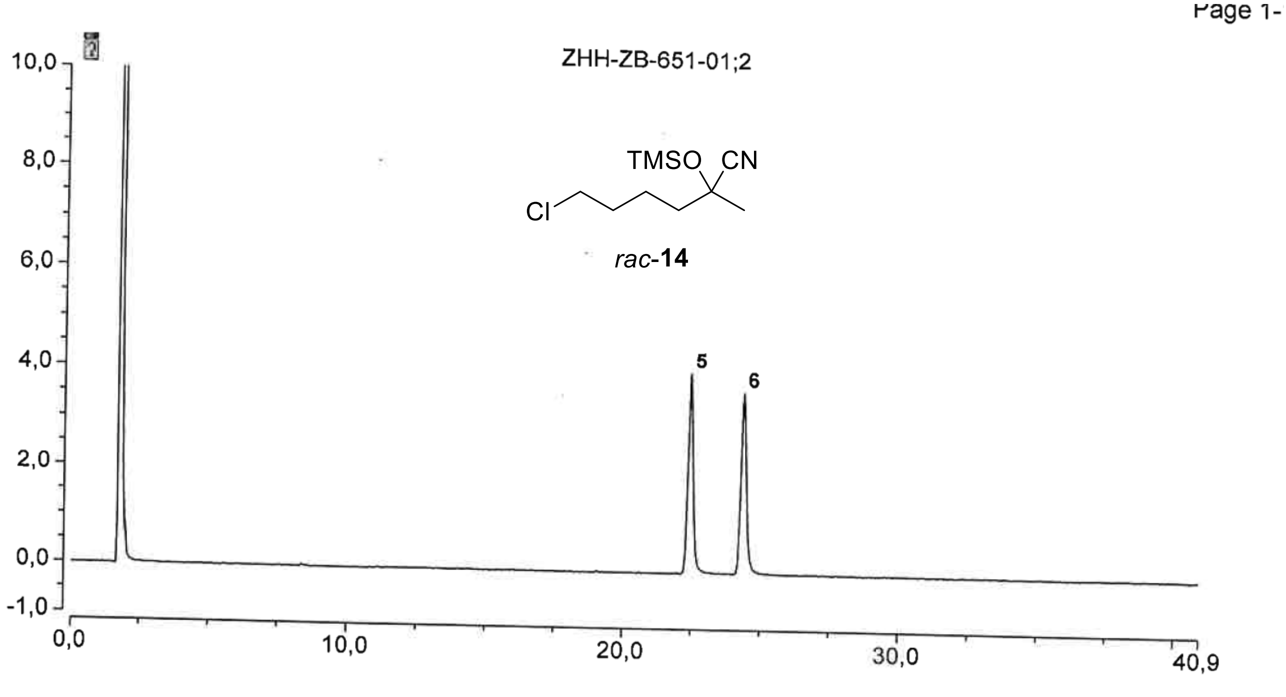

Peak #	t_R/min	% peak area
1	22.4	50.02
2	24.4	49.98
Total		100

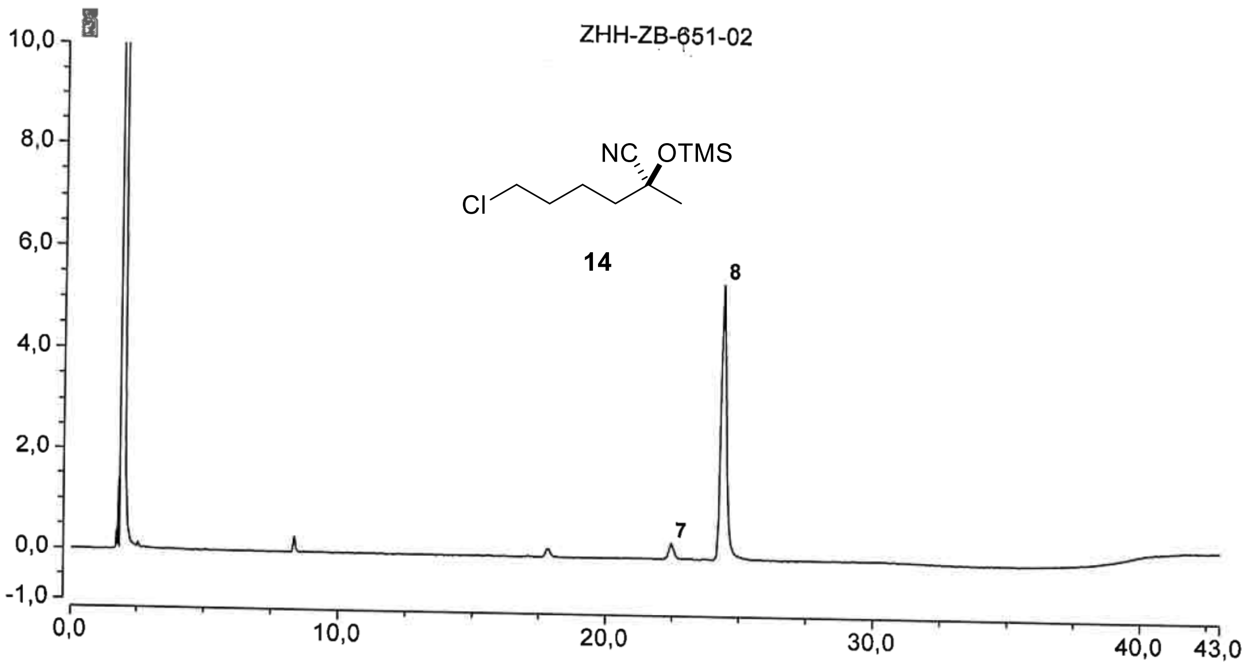

Peak #	t_R/min	% peak area
1	22.5	4.86
2	24.4	95.14
Total		100

GC (30.0 m BGB 176, injection temperature: 220 °C, 85 °C iso 55 min, 8 °C/min, 220 °C iso 3 min, 0.5 bar H_2)

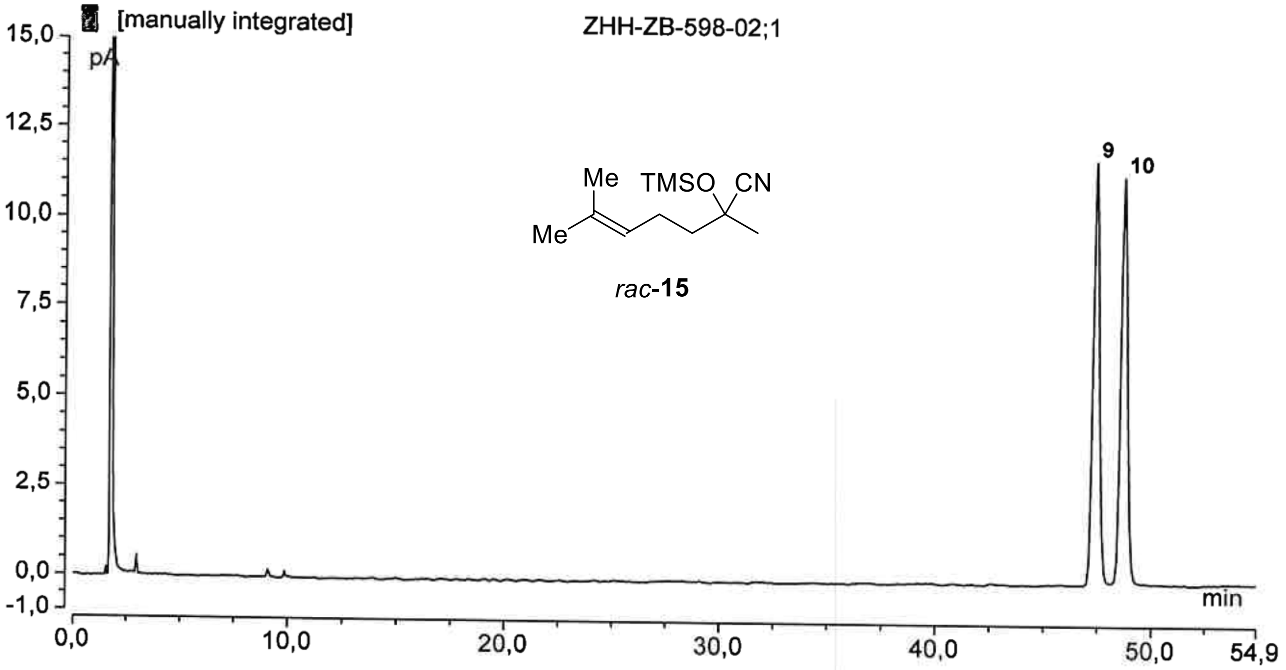

Peak #	t_R/min	% peak area
1	47.4	50.05
2	48.7	49.95
Total		100

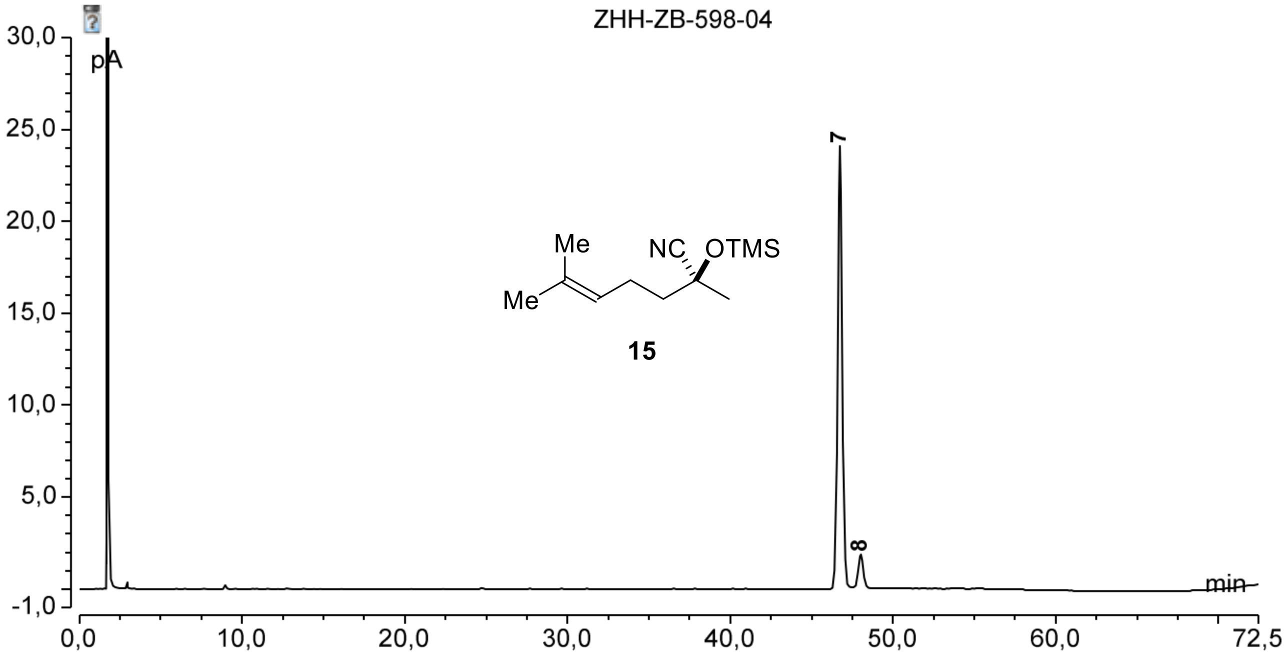

Peak #	t_R/min	% peak area
1	46.7	92.56
2	48.0	7.44
Total		100

GC (25.0 m Hydrodex-gamma TBDAc-CD, injection temperature: 220 °C, 95 °C iso 60 min, 8 °C/min, 230 °C iso 3 min, 0.5 bar H_2)

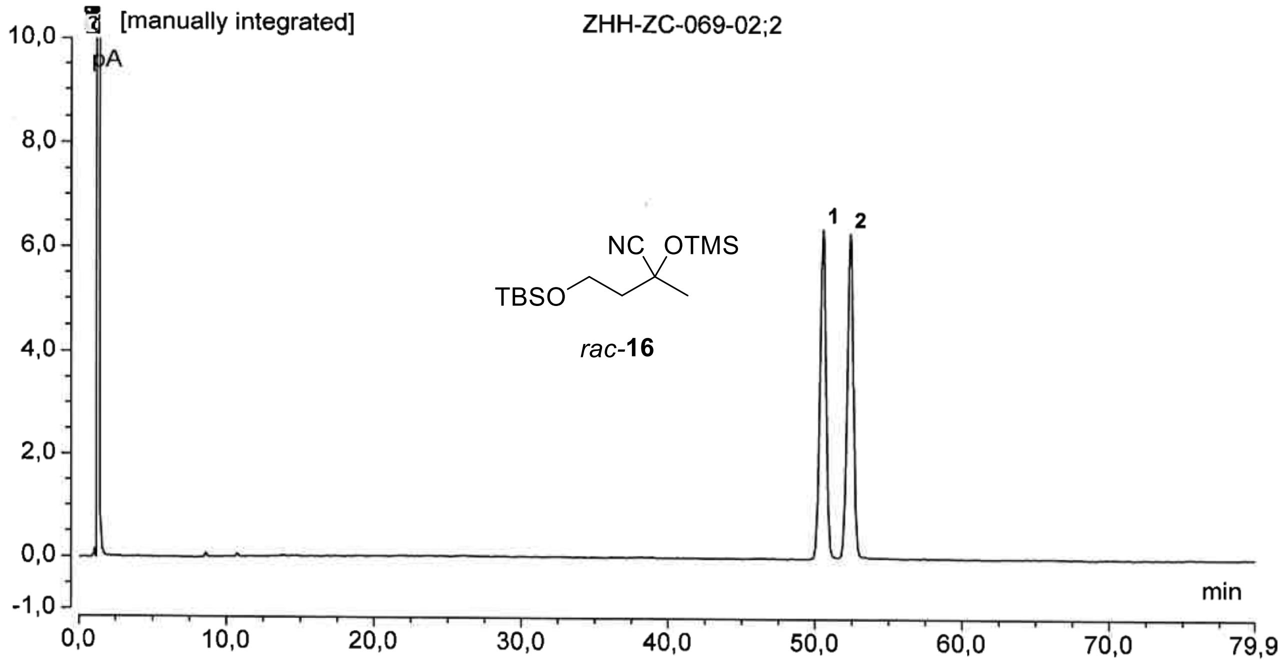

Peak #	t_R/min	% peak area
1	50.6	49.84
2	52.4	50.16
Total		100

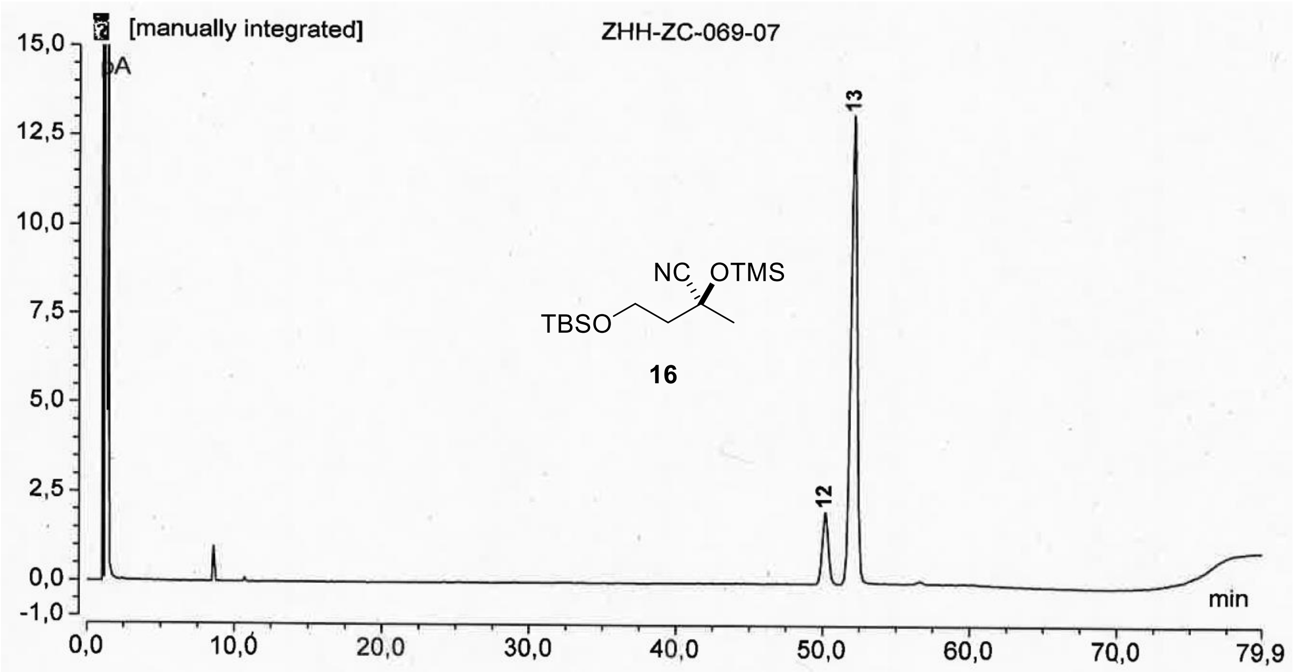

Peak #	t_R/min	% peak area
1	50.2	12.72
2	52.2	87.28
Total		100

GC (30.0 m BGB 176, injection temperature: 220 °C, 70 °C iso 120 min, 25 °C/min, 220 °C iso 2 min, 0.5 bar H_2)

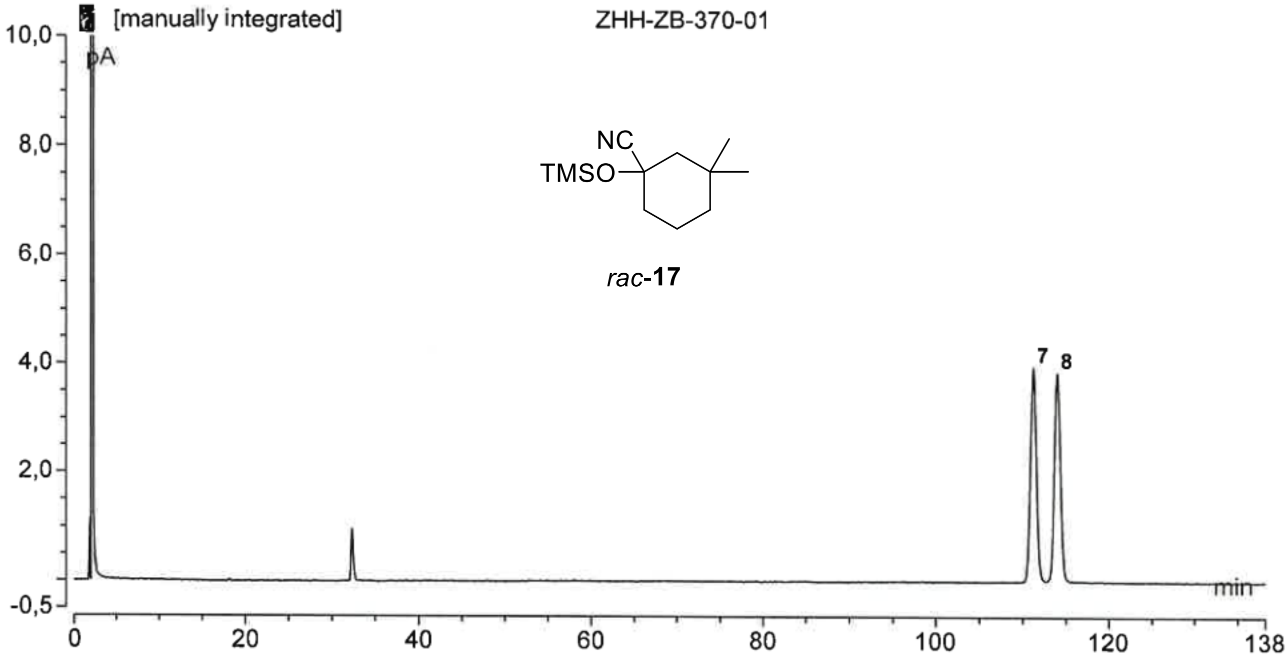

Peak #	t_R/min	% peak area
1	111.2	50.07
2	114.0	49.93
Total		100

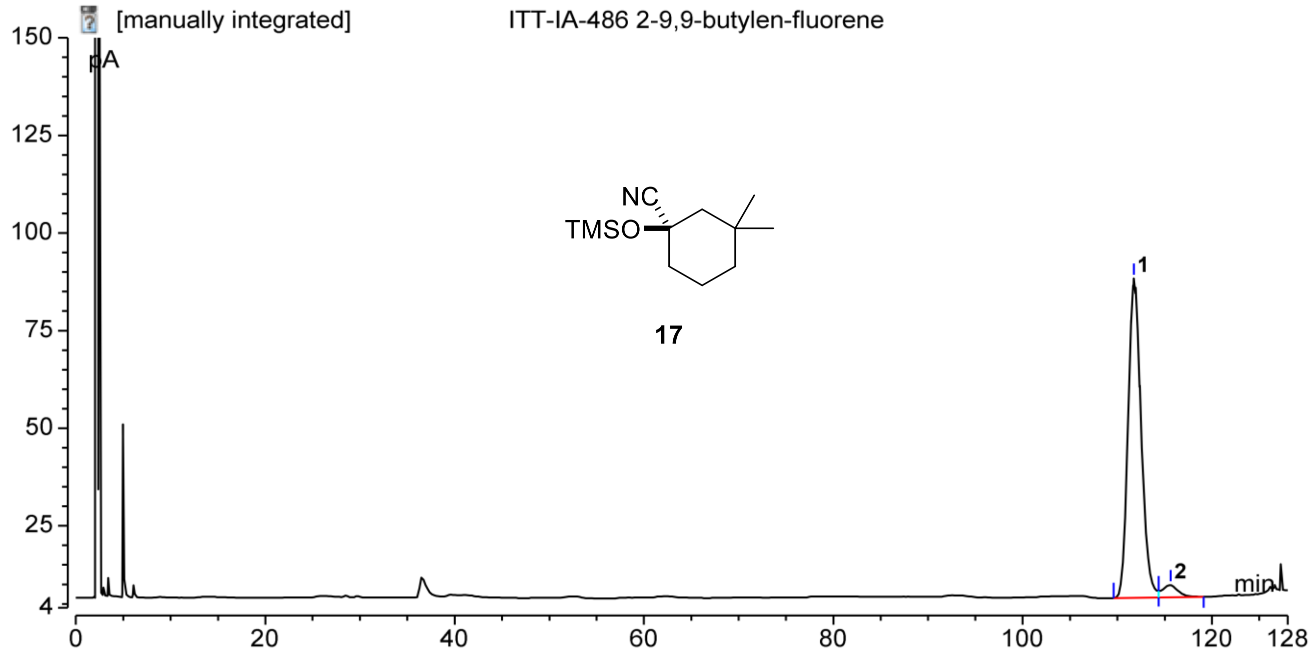

Peak #	t_R/min	% peak area
1	111.7	95.60
2	115.6	4.40
Total		100

GC (30.0 m BGB 176, injection temperature: 220 °C, 110 °C iso 65 min, 8 °C/min, 240 °C iso 3 min, 0.5 bar H_2)

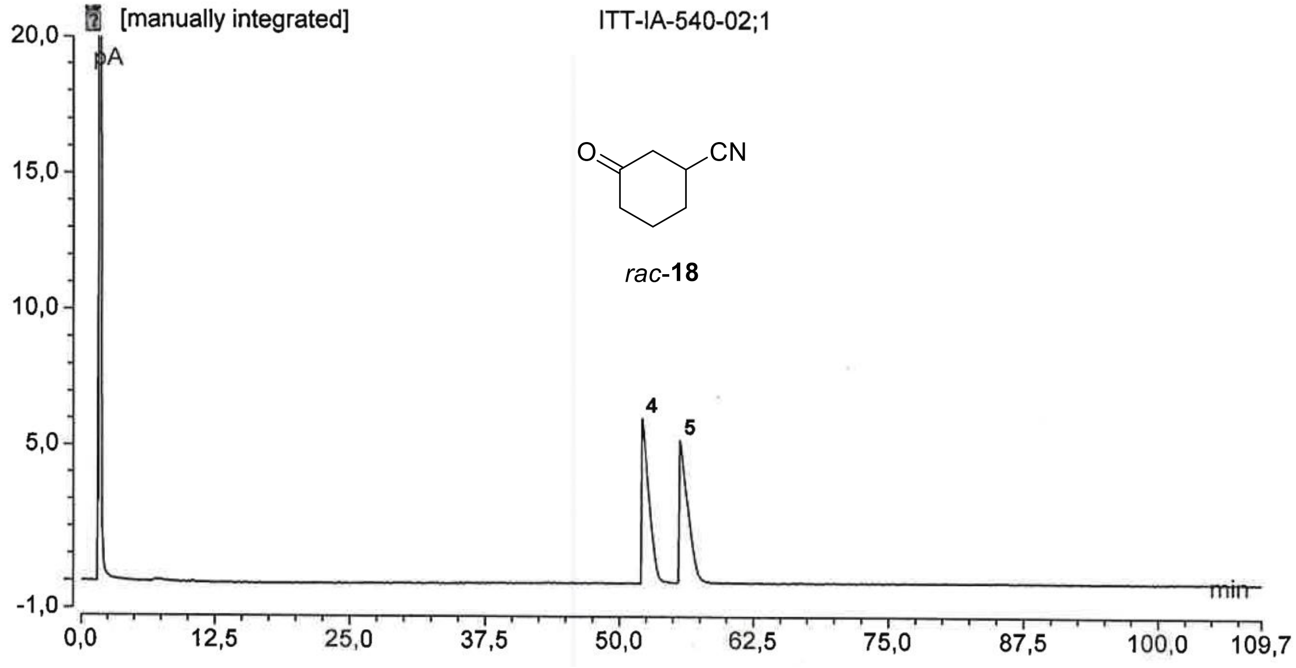

Peak #	t_R/min	% peak area
1	52.2	50.24
2	55.7	49.76
Total		100

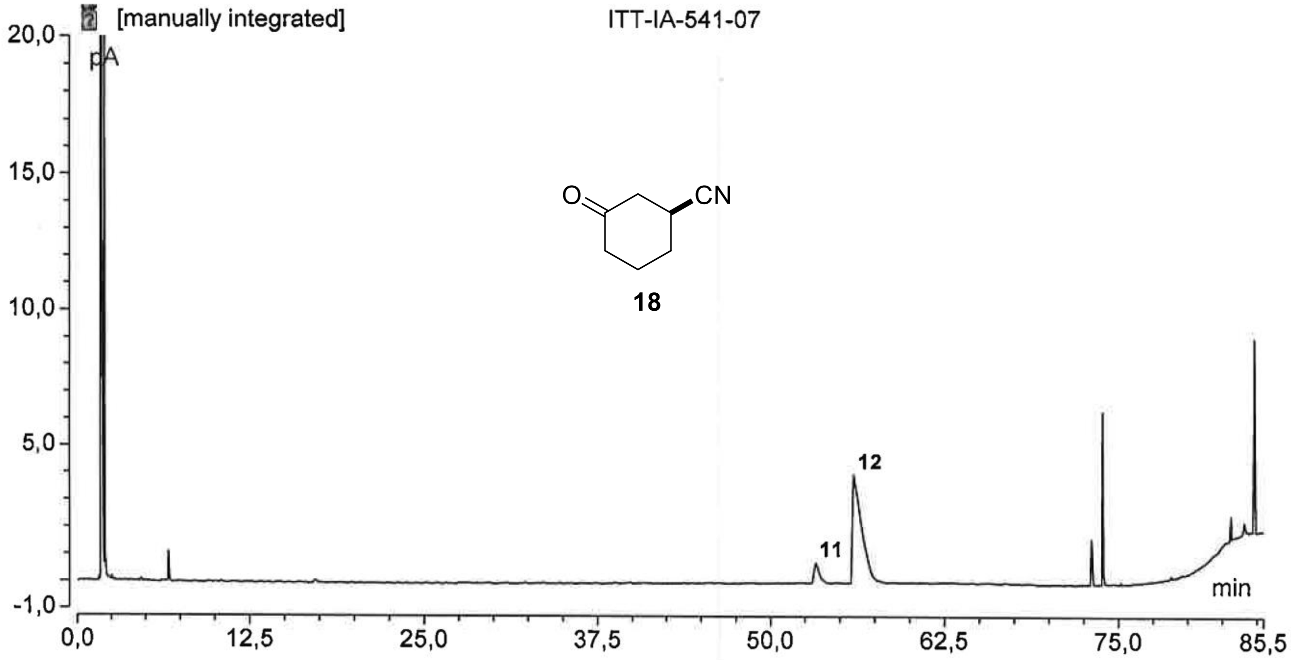

Peak #	t_R/min	% peak area
1	53.3	9.20
2	56.0	90.80
Total		100

HPLC (OJ-3R, MeOH: Water = 75:25, 1.0 mL/min, 298 K, 220 nm)

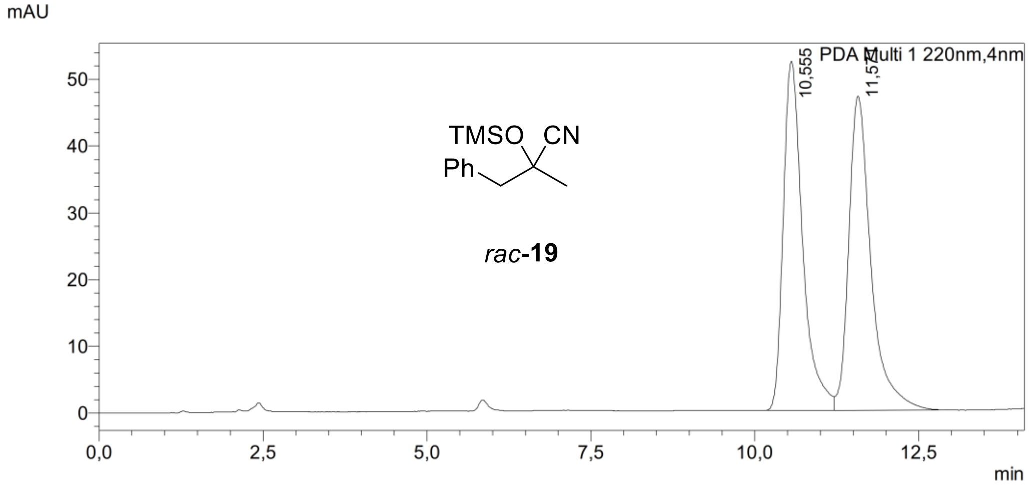

Peak #	t_R/min	% peak area
1	10.6	49.25
2	11.6	50.75
Total		100

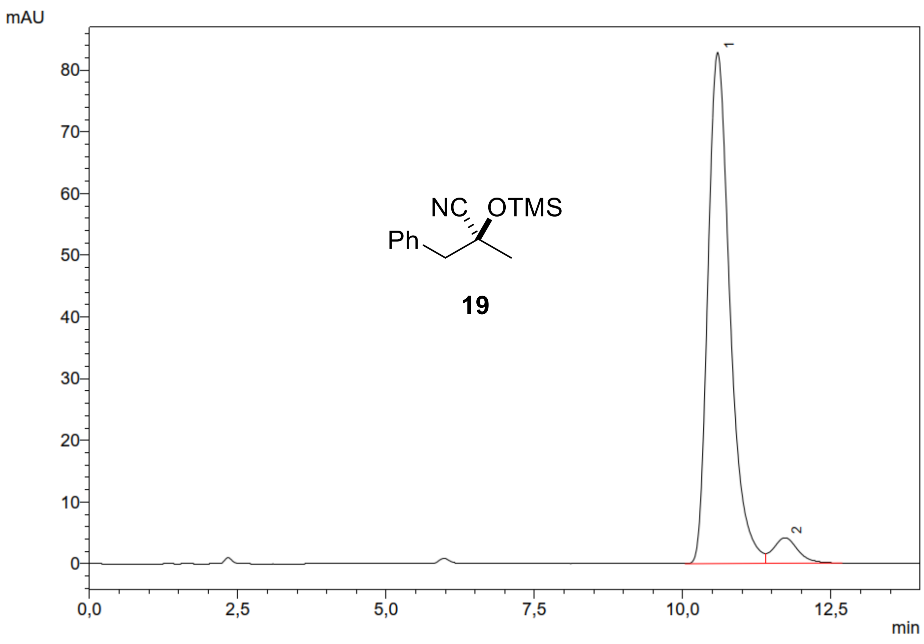

Peak #	t_R/min	% peak area
1	10.6	94.69
2	11.7	5.31
Total		100

HPLC (OD-3, 100% heptane, 0.5 mL/min, 298 K, 220 nm)

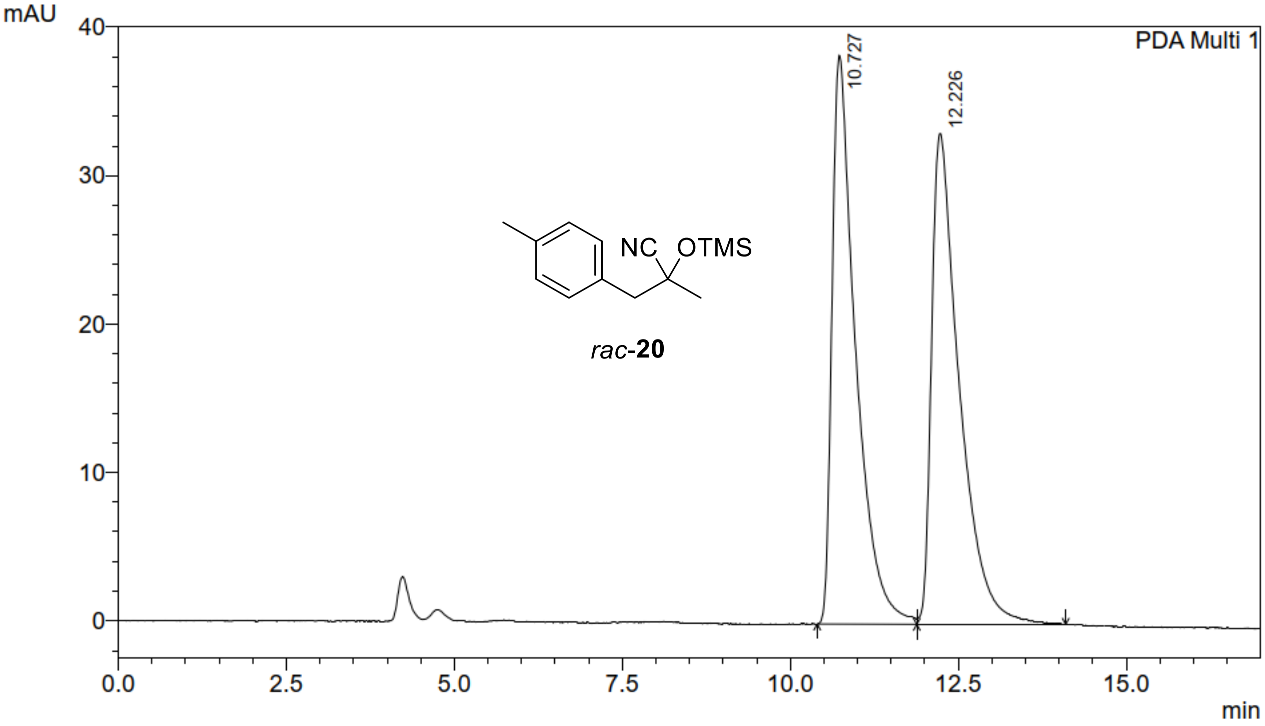

Peak #	t_R/min	% peak area
1	10.7	49.65
2	12.2	50.35
Total		100

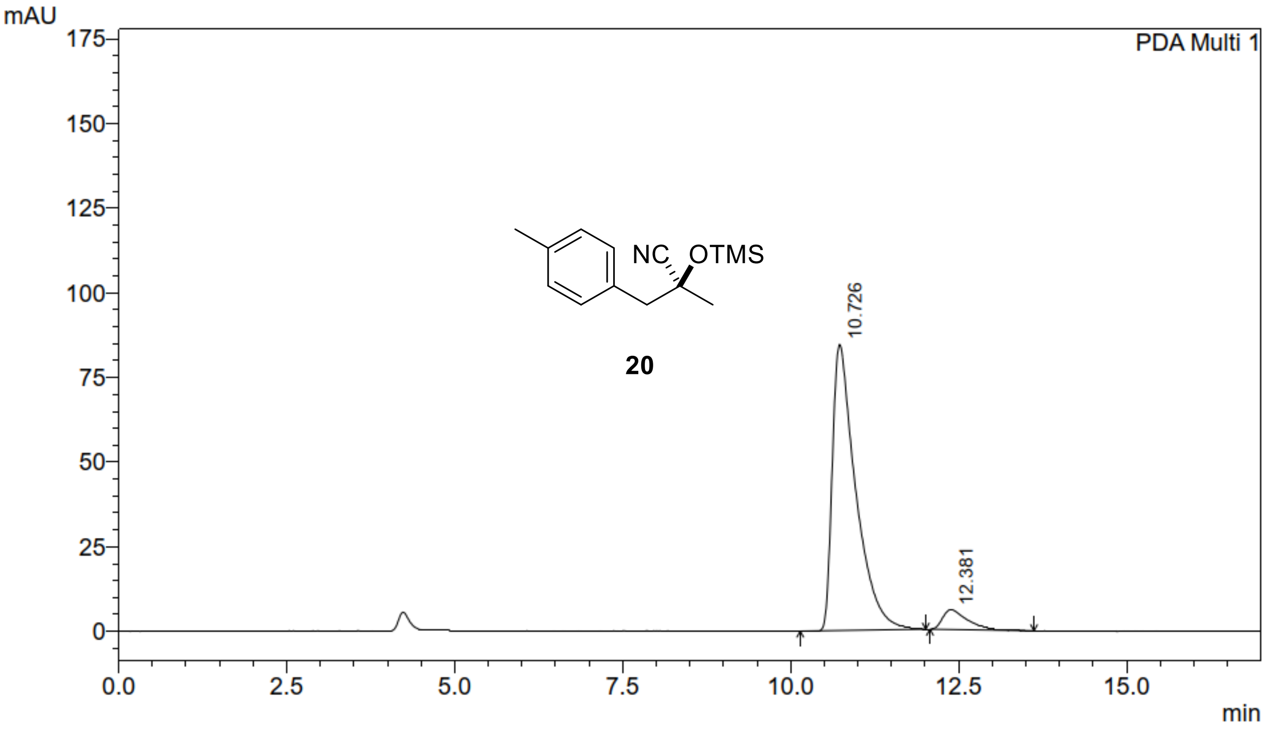

Peak #	t_R/min	% peak area
1	10.7	93.13
2	12.4	6.87
Total		100

HPLC (OJ-3R, MeOH: Water = 75:25, 1.0 mL/min, 298 K, 220 nm)

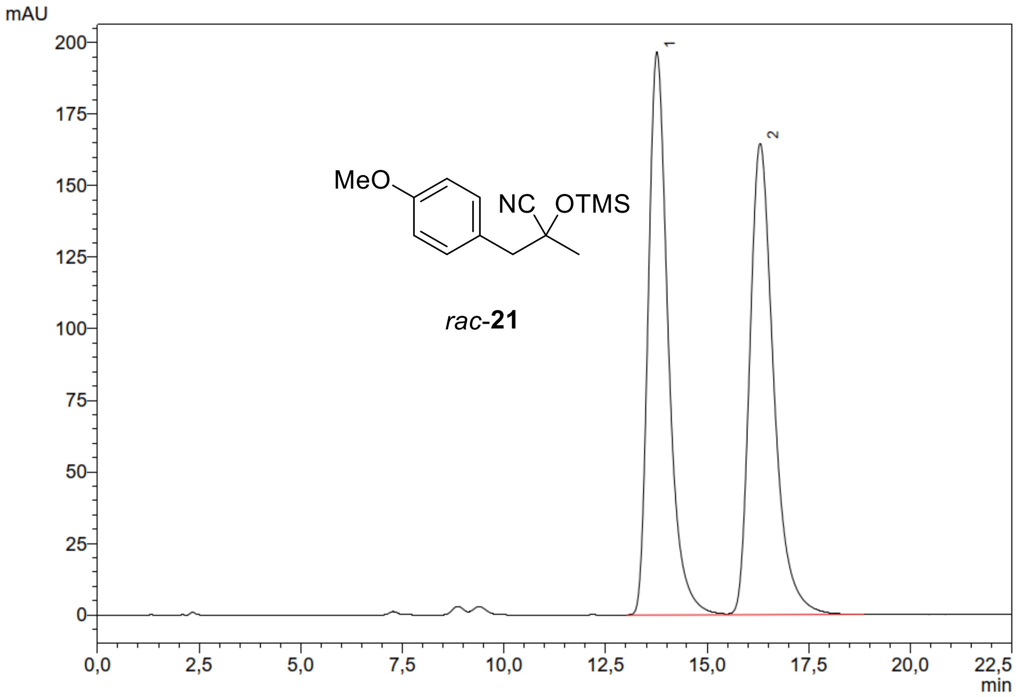

Peak #	t_R/min	% peak area
1	13.8	49.97

2	16.3	50.03
Total		100

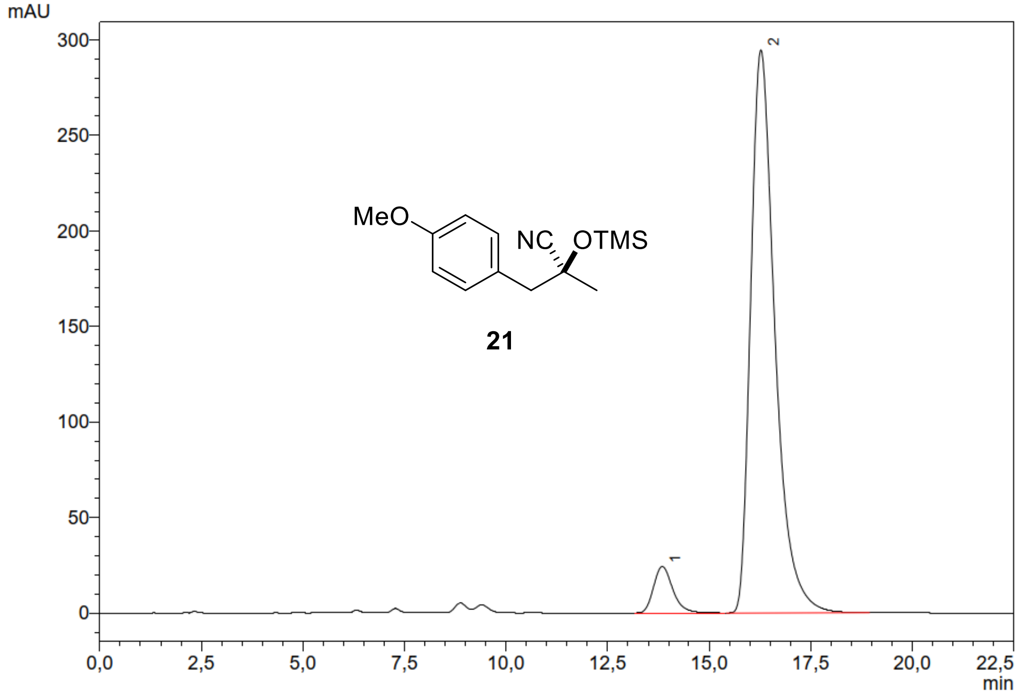

Peak #	t_R/min	% peak area
1	13.8	6.22
2	16.3	93.78
Total		100

HPLC (OJ-3, isopropanol: heptane = 0.5:99.5, 0.5 mL/min, 298 K, 254 nm)

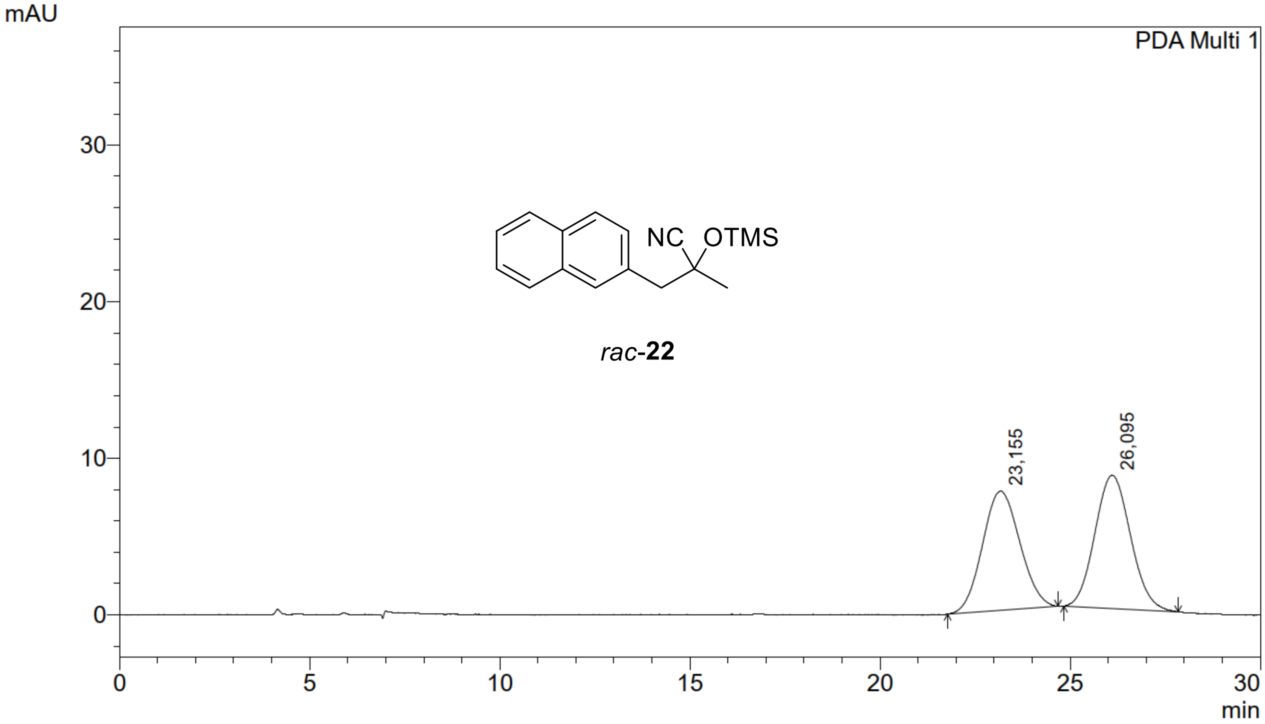

Peak #	t_R/min	% peak area
1	23.2	48.41
2	26.1	51.59
Total		100

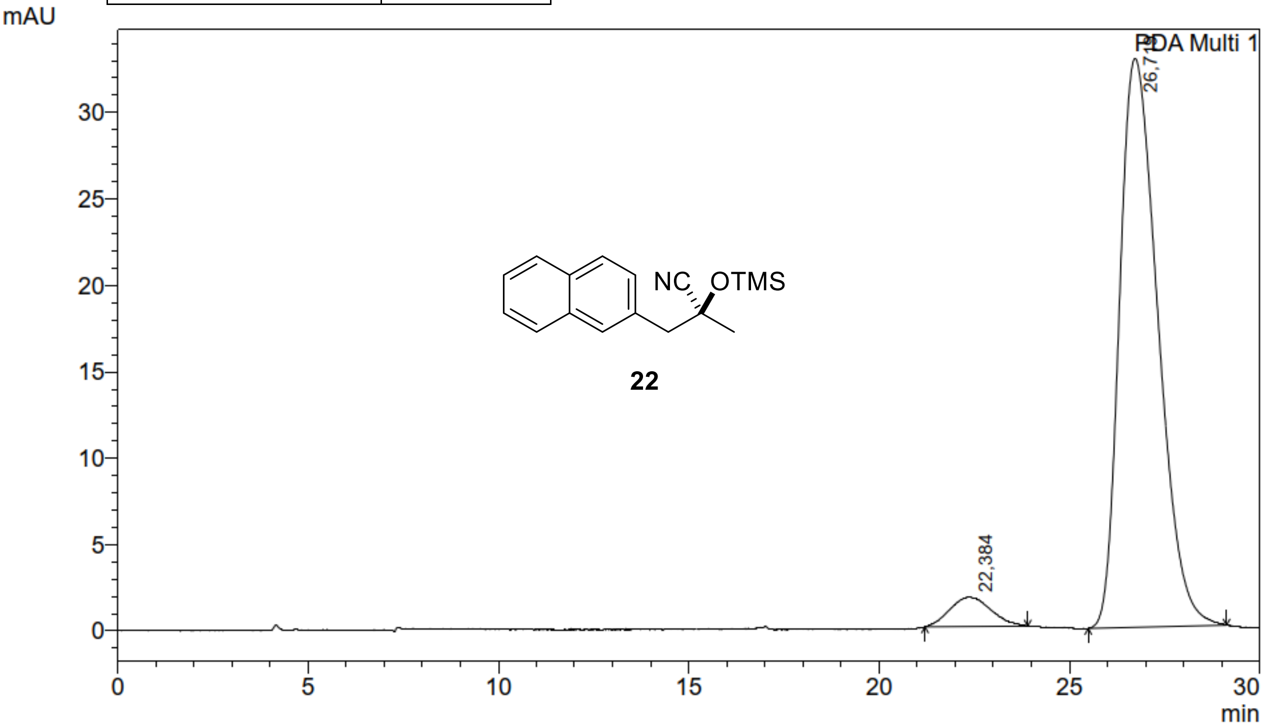

Peak #	t_R/min	% peak area
1	22.4	5.26
2	26.7	94.74
Total		100

HPLC (IA, 100% heptane, 0.5 mL/min, 298 K, 220 nm)

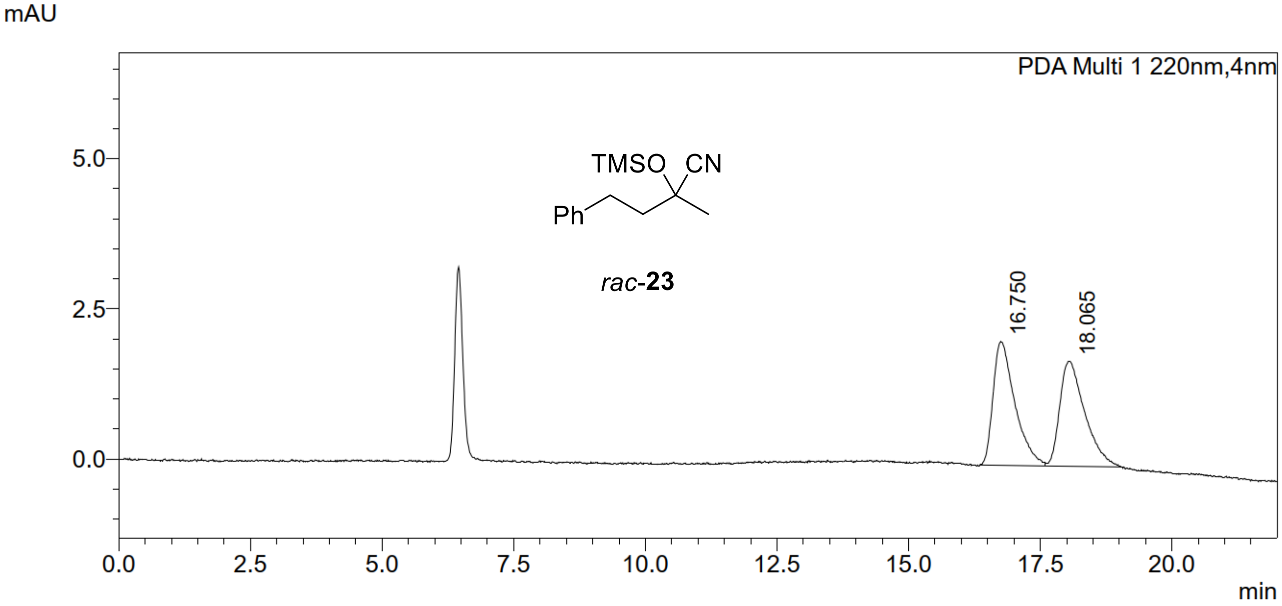

Peak #	t_R/min	% peak area
1	16.8	50.68
2	18.1	49.32
Total		100

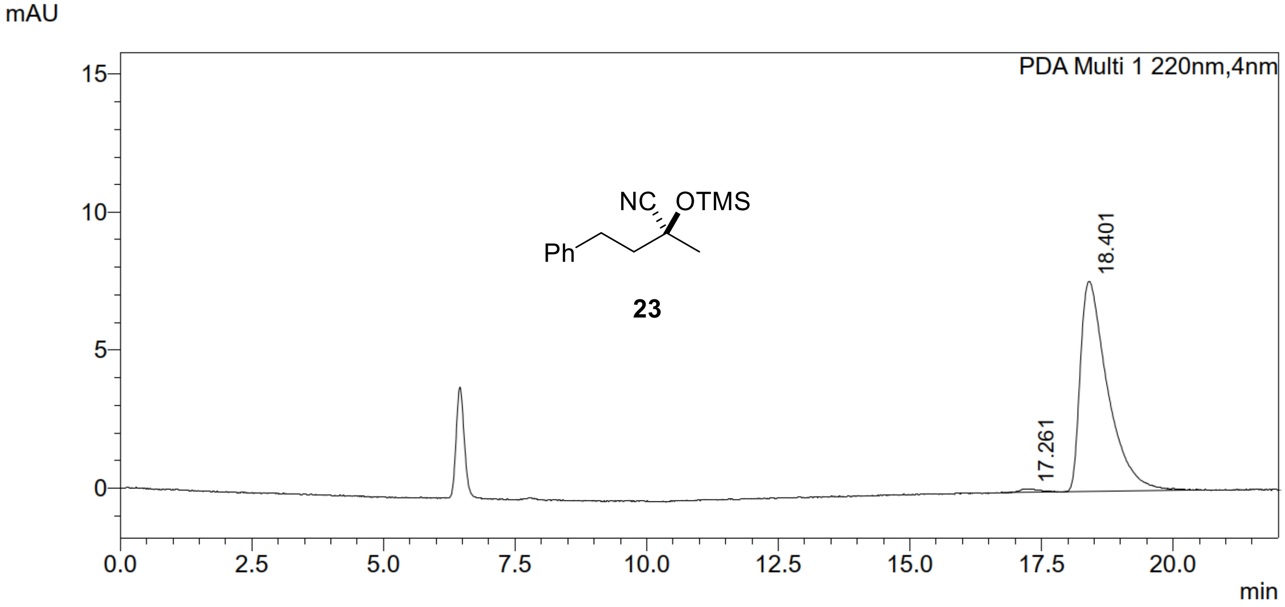

Peak #	t_R/min	% peak area
1	17.3	0.96
2	18.4	99.04
Total		100

For reaction of enol silanes with HCN, the e.r. = 96:4, the HPLC spectrum was shown below:

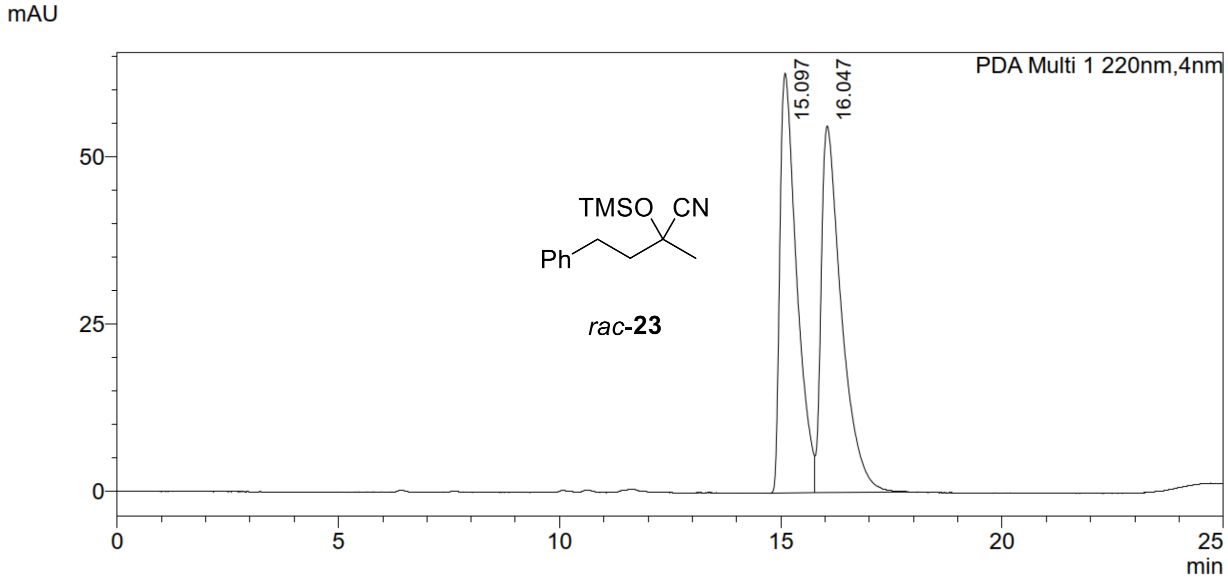

Peak #	t_R/min	% peak area
1	15.1	48.57
2	16.0	51.43
Total		100

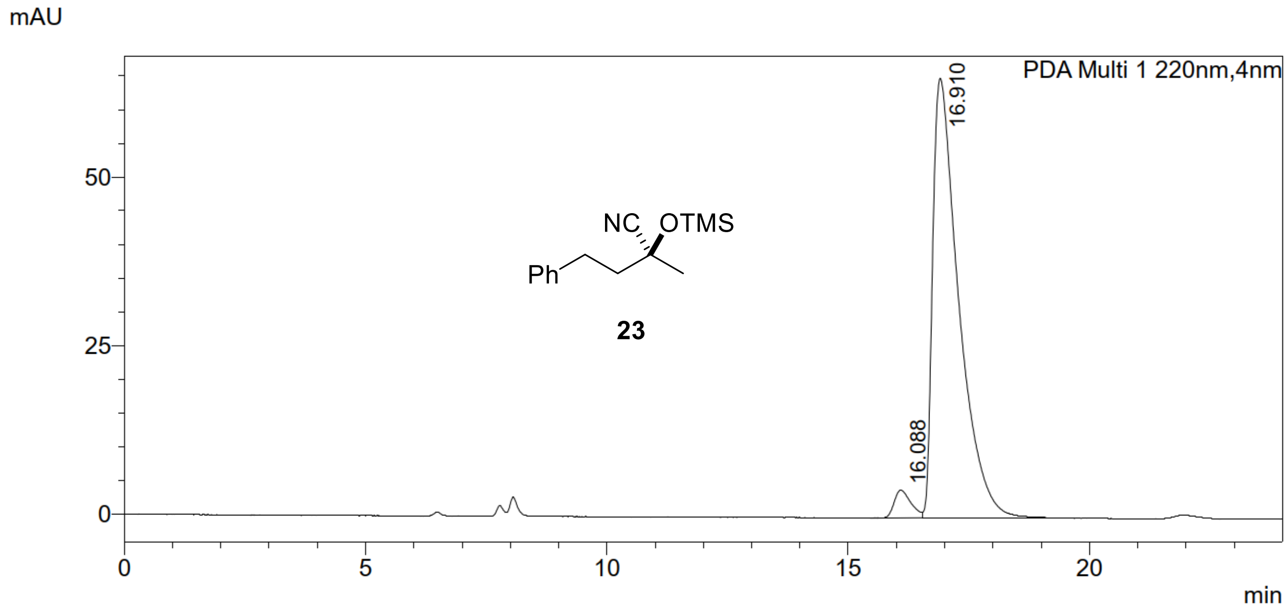

Peak #	t_R/min	% peak area
1	16.1	3.93
2	16.9	96.07
Total		100

HPLC spectra of product from gram-scale synthesis, the e.r. = 95:5:

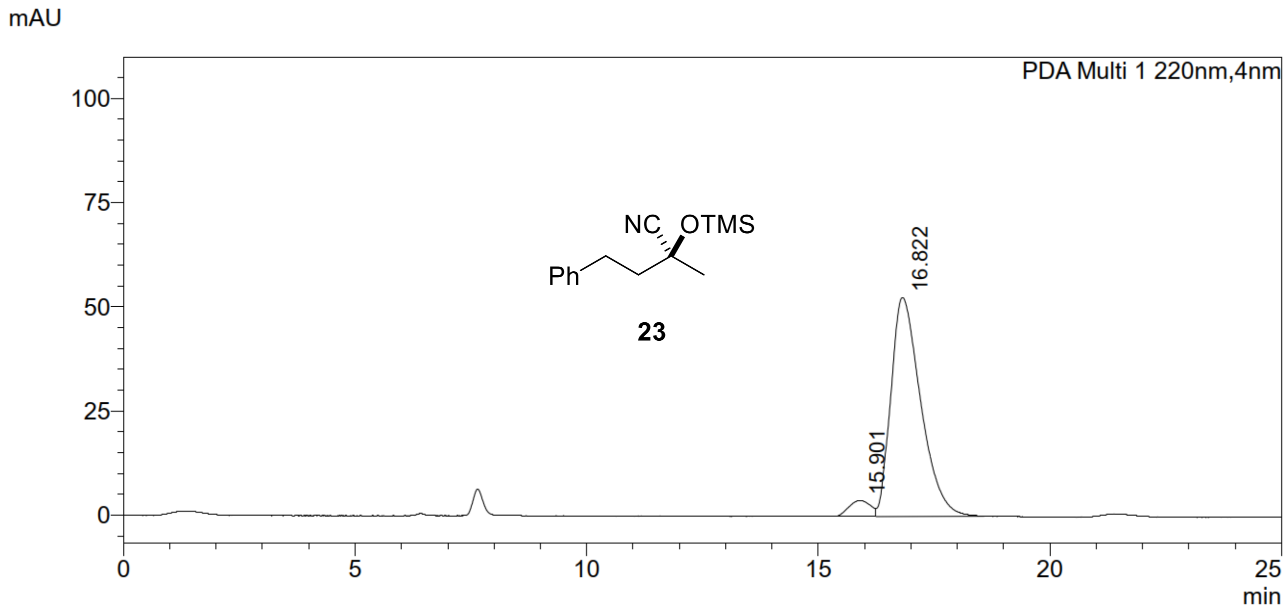

Peak #	t_R/min	% peak area
1	15.9	5.10
2	16.8	94.90
Total		100

For reaction in Et_2O at –20 °C, the e.r. = 92:8, the HPLC spectrum was shown below. New optimization was performed because the old method didn’t show baseline separation in this case: HPLC (IB N3, MeOH: Water = 75:25, 1.0 mL/min, 298 K, 220 nm)

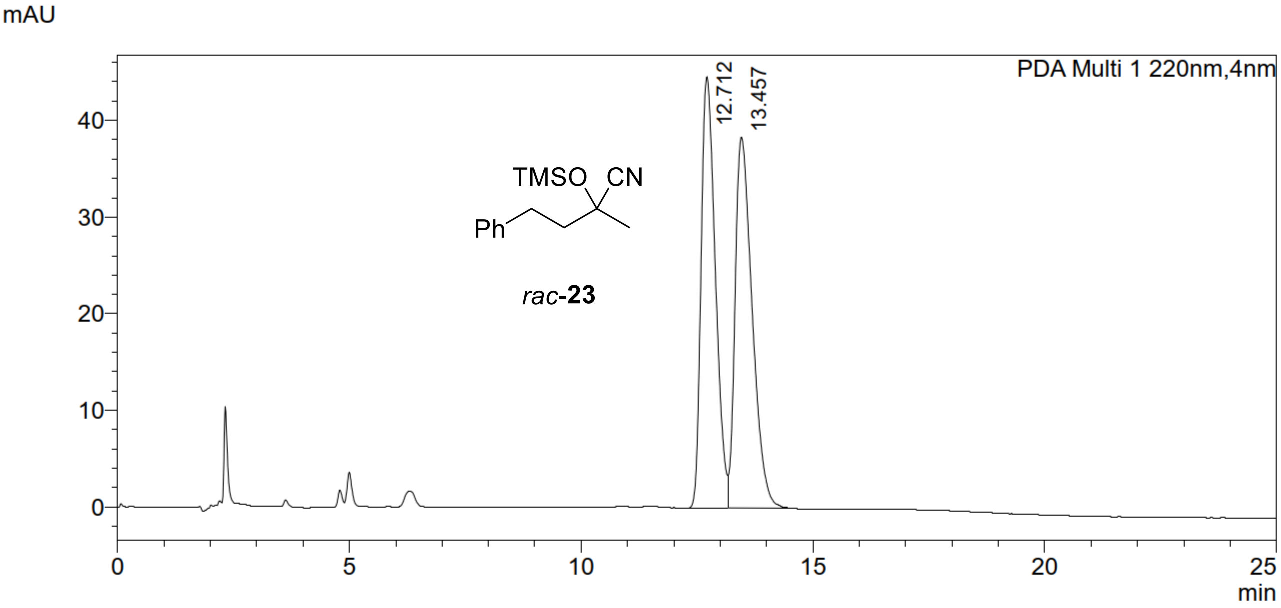

Peak #	t_R/min	% peak area
1	12.7	49.27
2	13.5	50.73
Total		100

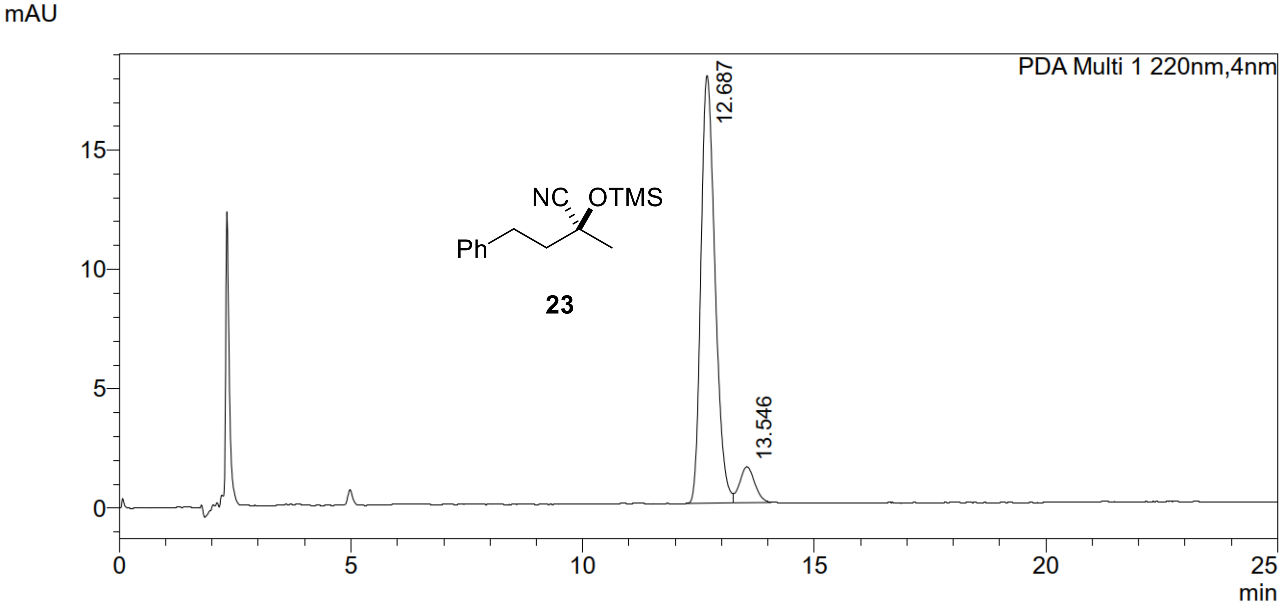

Peak #	t_R/min	% peak area
1	12.7	91.79
2	13.5	8.21
Total		100

HPLC (IA, 100% heptane, 0.5 mL/min, 298 K, 220 nm)

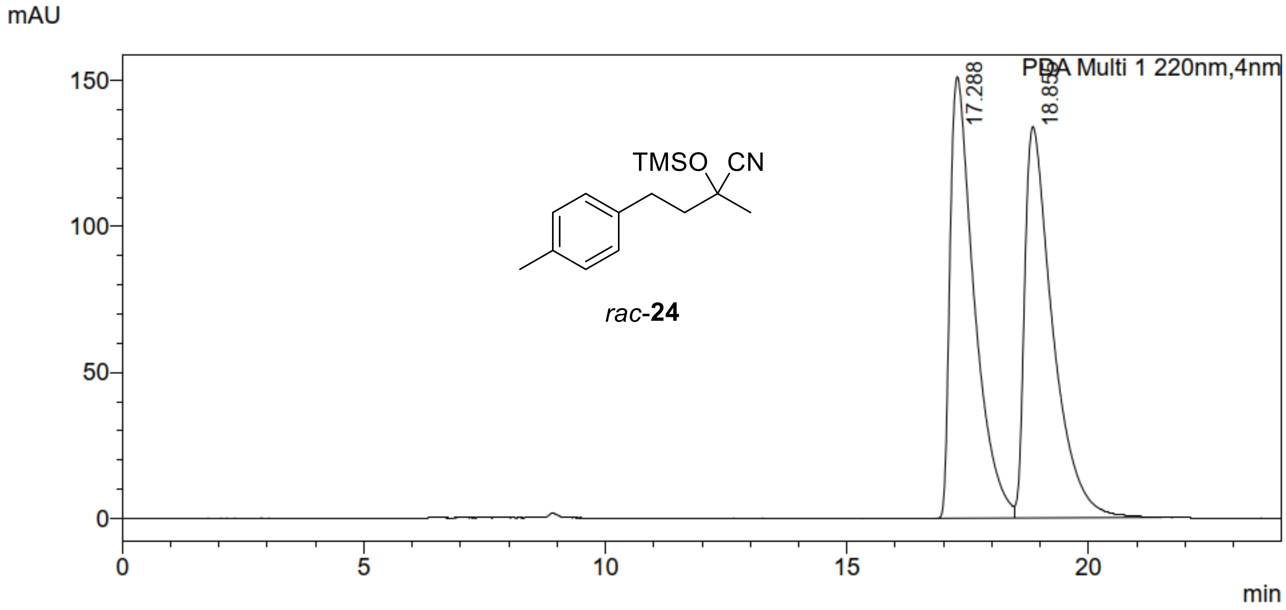

Peak #	t_R/min	% peak area
1	17.3	49.35
2	18.8	50.65
Total		100

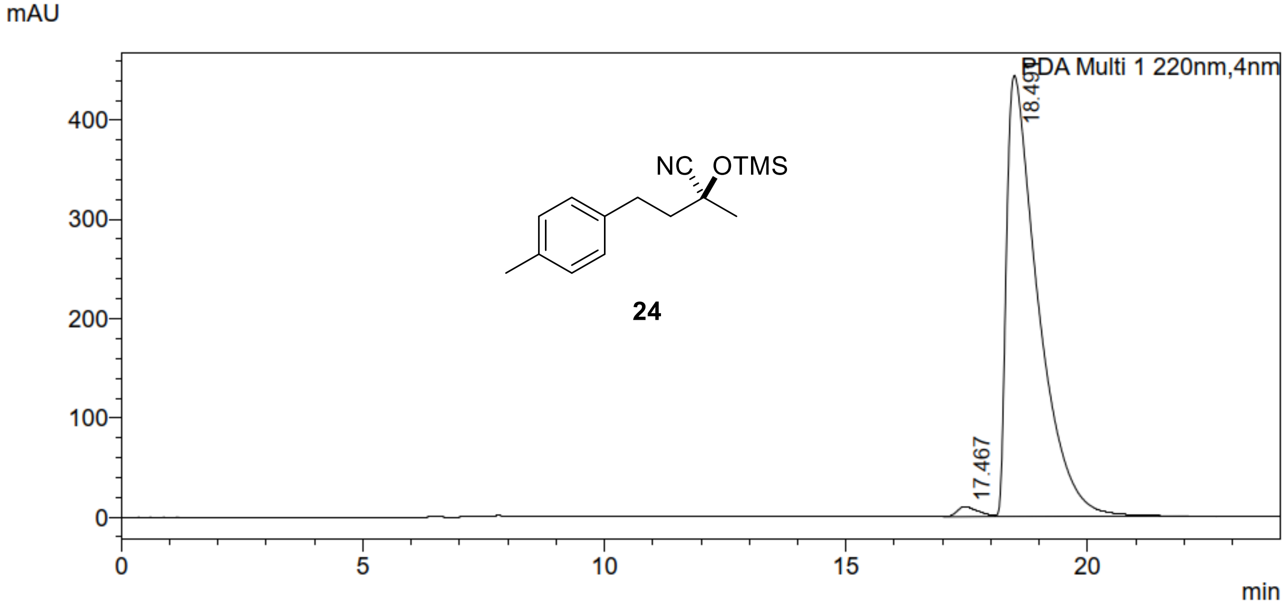

Peak #	t_R/min	% peak area
1	17.5	1.48
2	18.5	98.52
Total		100

HPLC (AS-3, isopropanol: heptane = 0.5:99.5, 0.5 mL/min, 298 K, 254 nm)

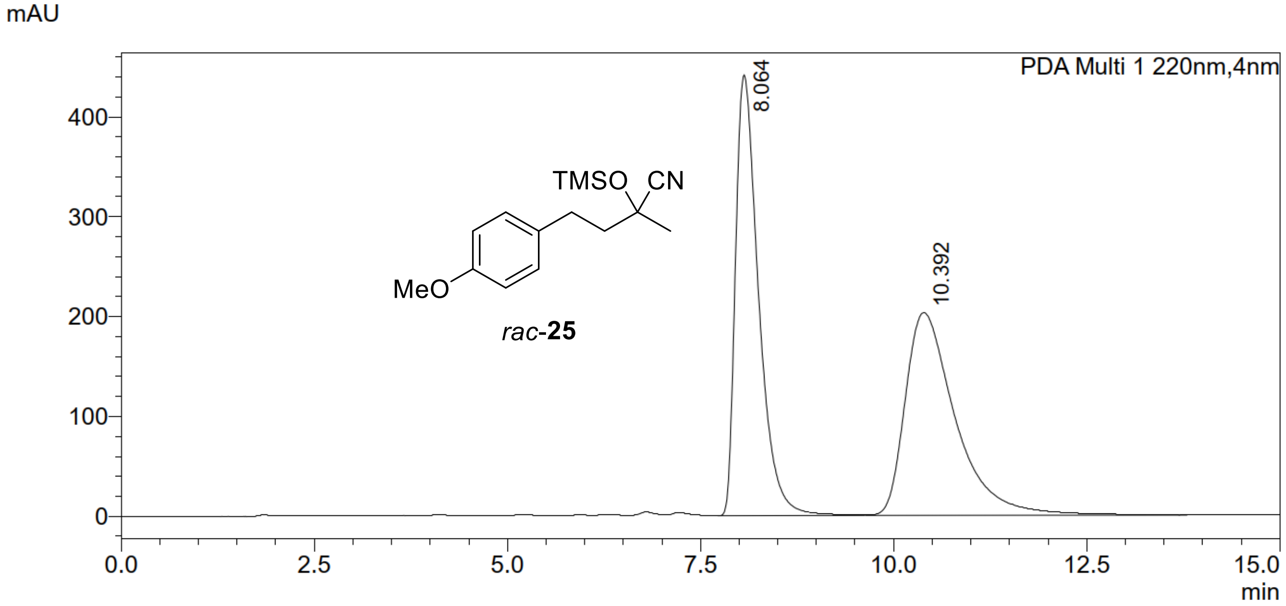

Peak #	t_R/min	% peak area
1	8.1	49.94
2	10.4	50.06
Total		100

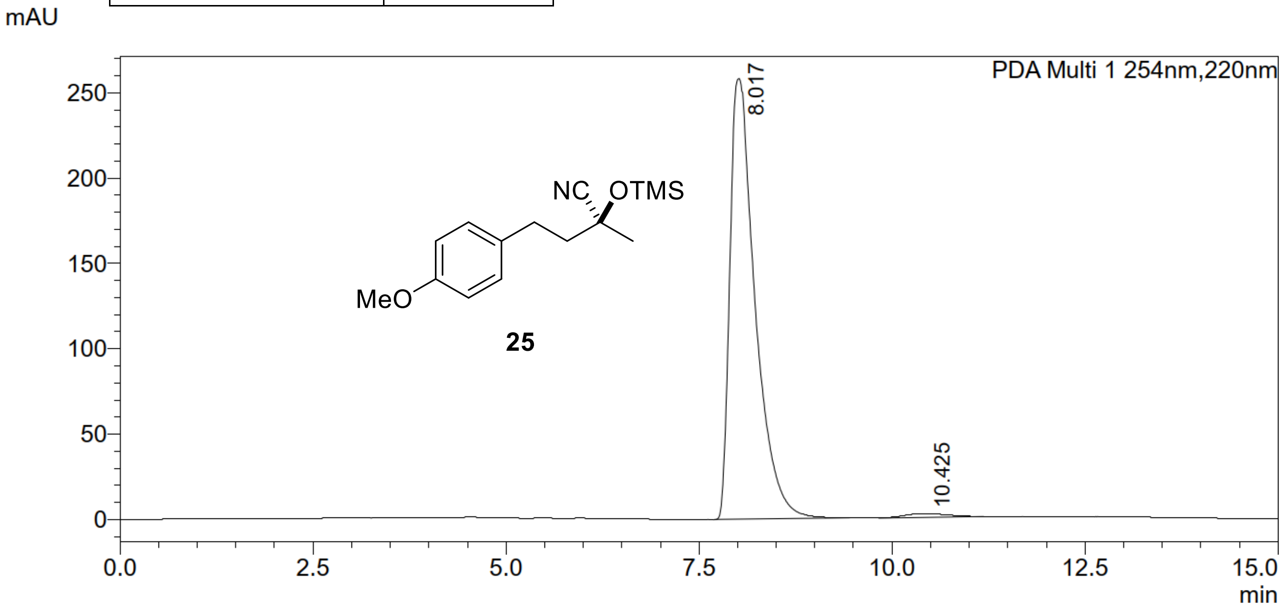

Peak #	t_R/min	% peak area
1	8.0	98.58
2	10.4	1.42
Total		100

HPLC (OD-3, 100% heptane, 0.5 mL/min, 298 K, 220 nm)

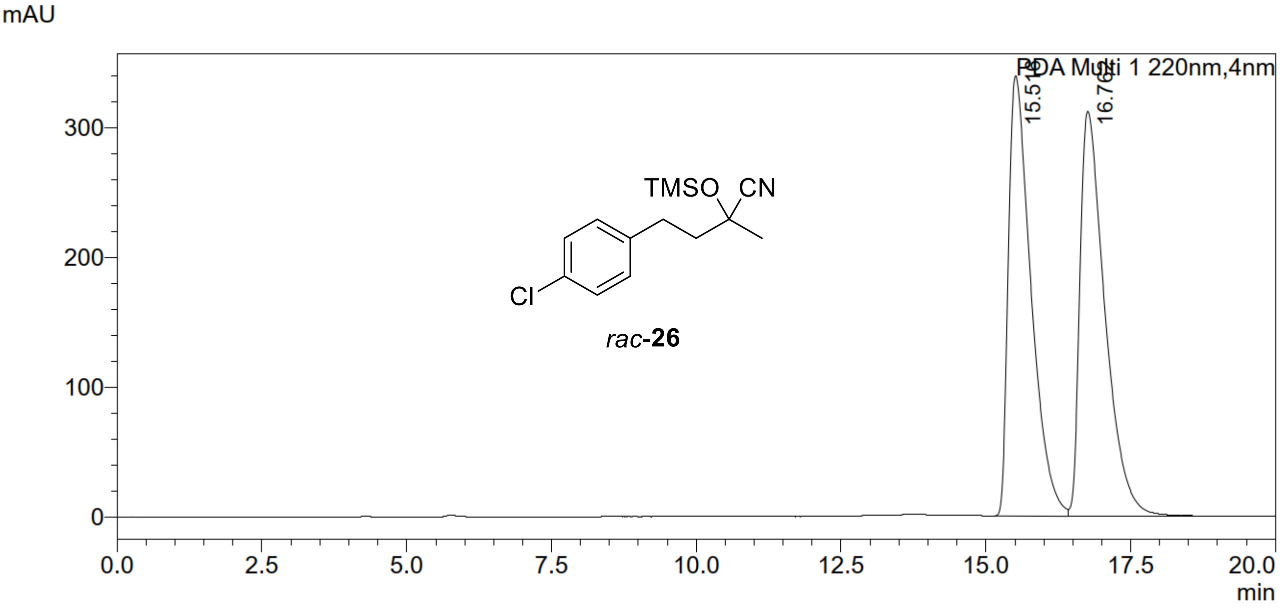

Peak #	t_R/min	% peak area
1	15.5	49.57
2	16.8	50.43
Total		100

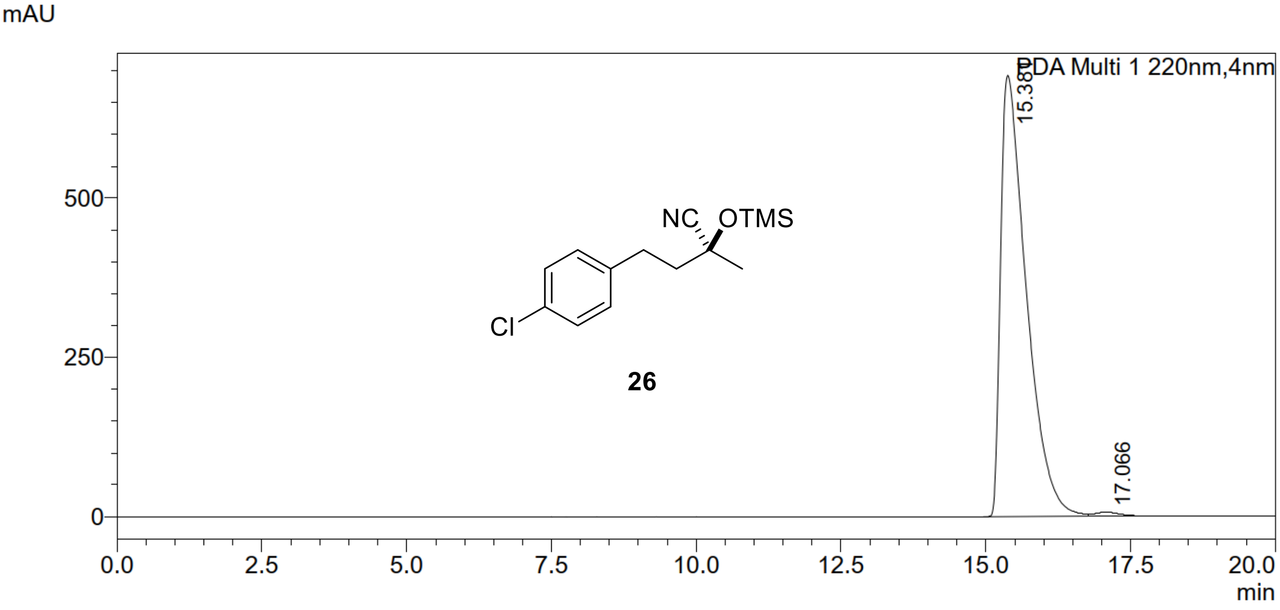

Peak #	t_R/min	% peak area
1	15.4	99.14
2	17.1	0.86
Total		100

HPLC (OD-3, 100% heptane, 0.5 mL/min, 298 K, 220 nm)

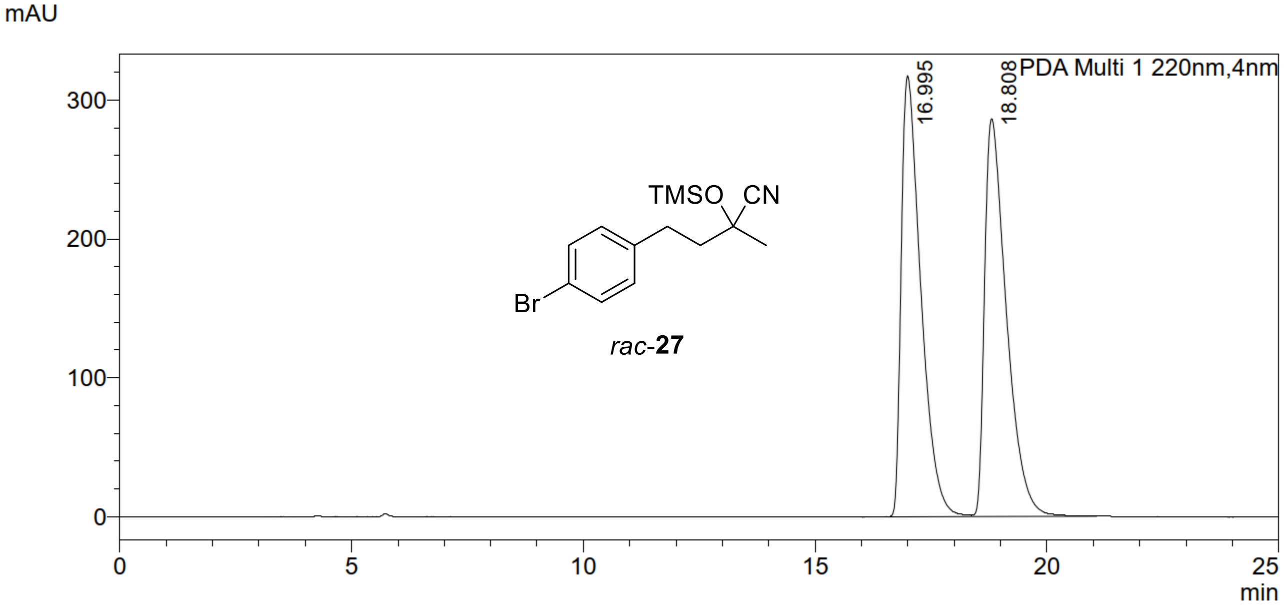

Peak #	t_R/min	% peak area
1	17.0	49.90
2	18.8	50.10
Total		100

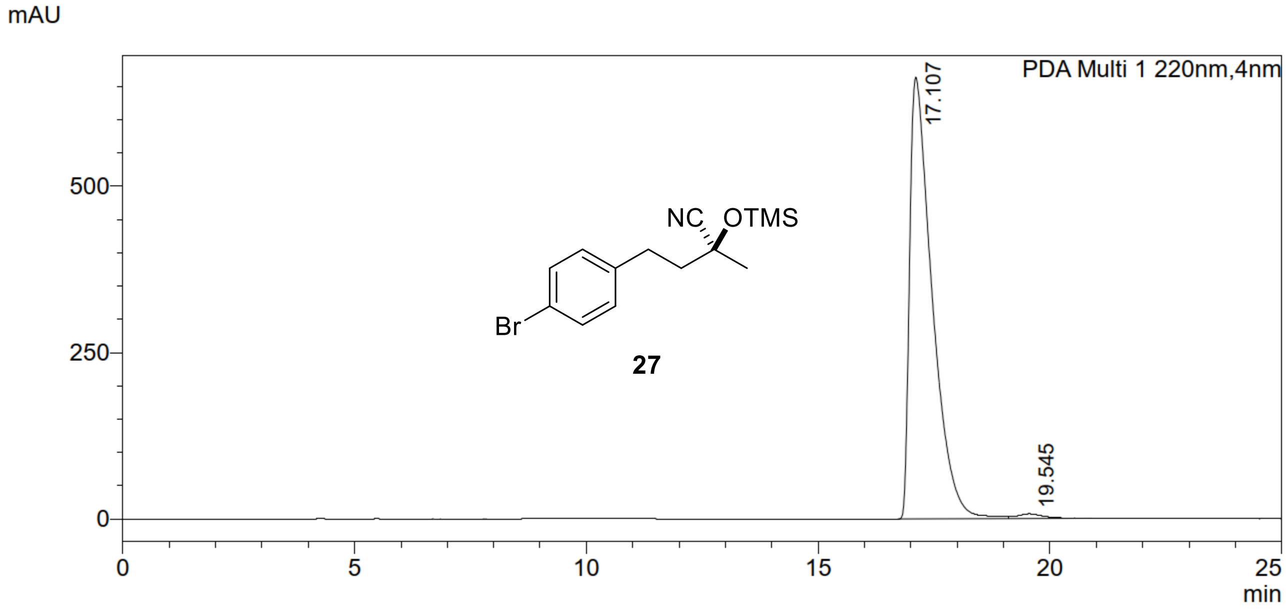

Peak #	t_R/min	% peak area
1	17.1	98.73
2	19.5	1.27
Total		100

HPLC (OJ-3, isopropanol: heptane = 0.5:99.5, 0.5 mL/min, 298 K, 220 nm)

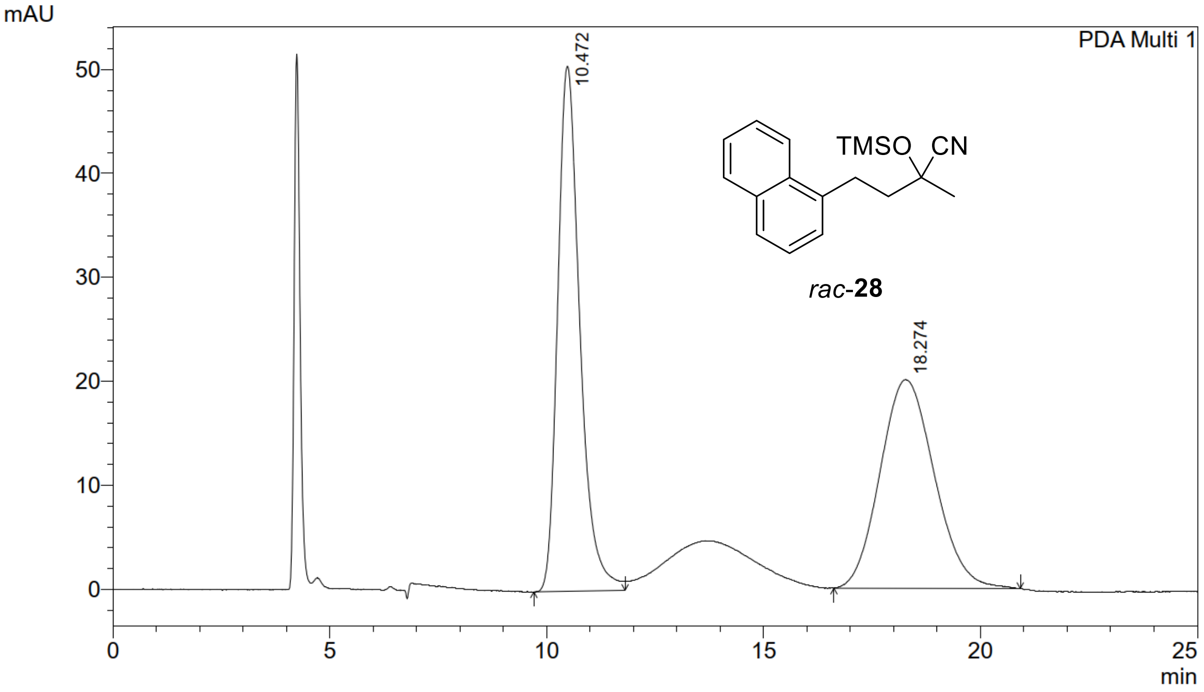

Peak #	t_R/min	% peak area
1	10.5	50.80
2	18.3	49.20
Total		100

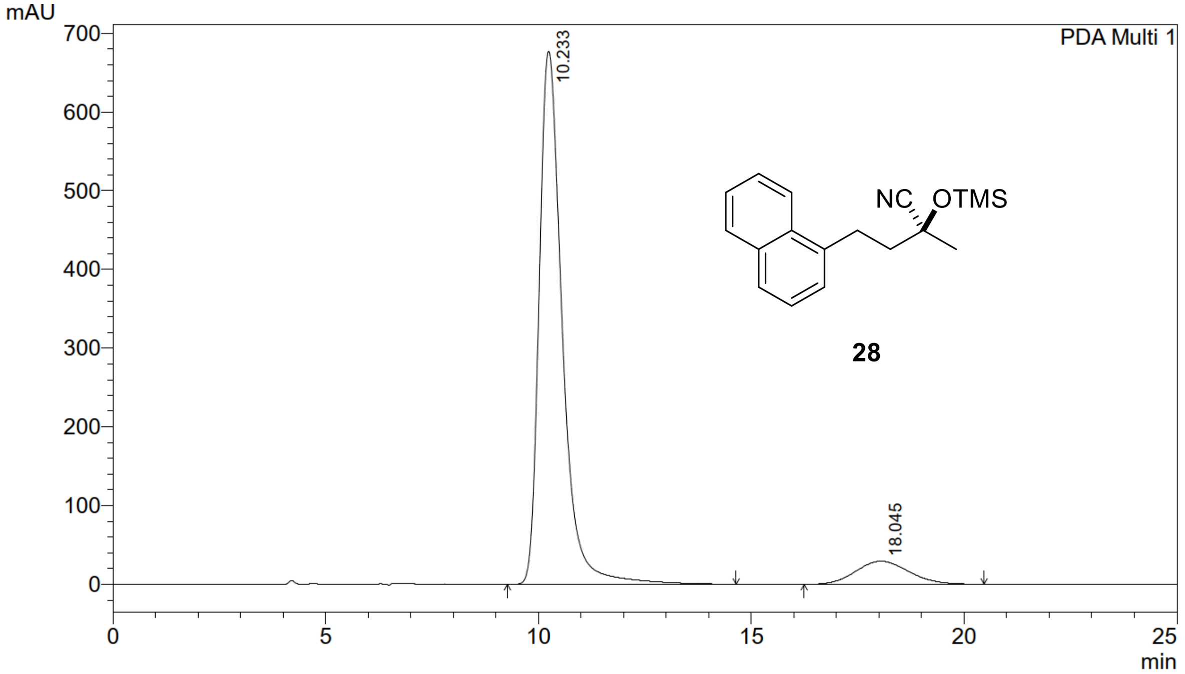

Peak #	t_R/min	% peak area
1	10.2	90.85
2	18.0	9.15
Total		100

HPLC (IB-3, isopropanol: heptane = 0.5:99.5, 0.5 mL/min, 298 K, 220 nm)

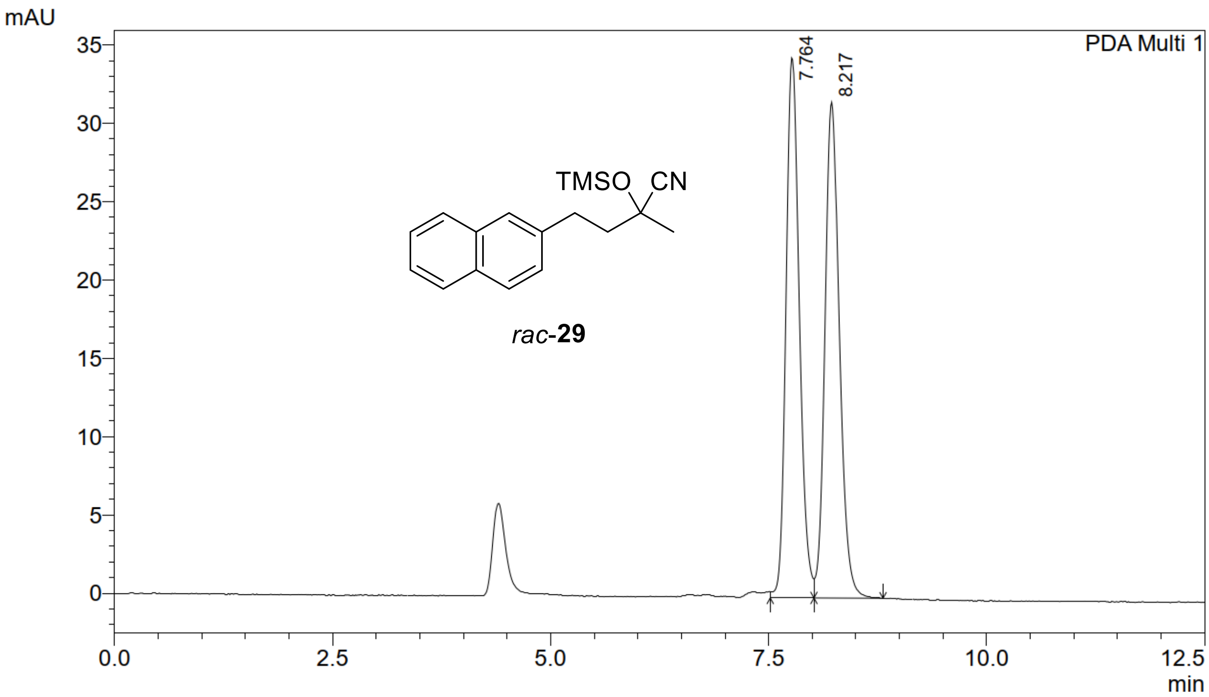

Peak #	t_R/min	% peak area
1	7.8	50.01
2	8.2	49.99
Total		100

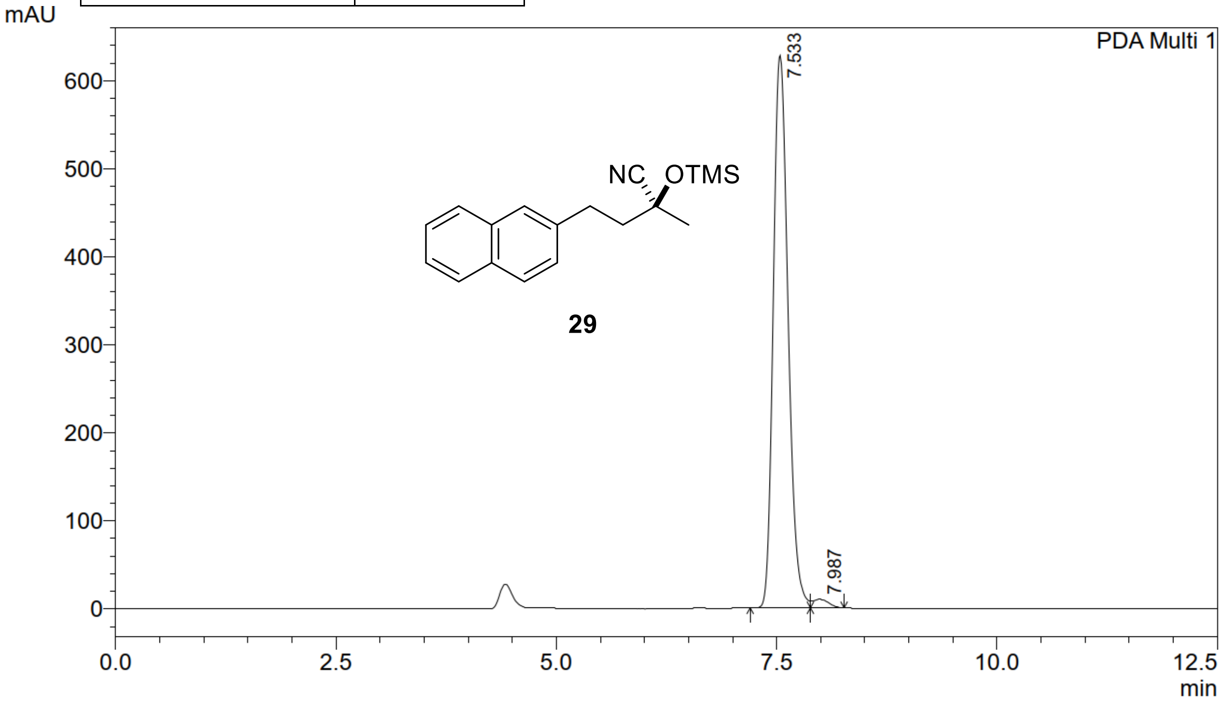

Peak #	t_R/min	% peak area
1	7.5	98.34
2	8.0	1.66
Total		100

HPLC (OD-3, 100% heptane, 0.5 mL/min, 298 K, 220 nm)

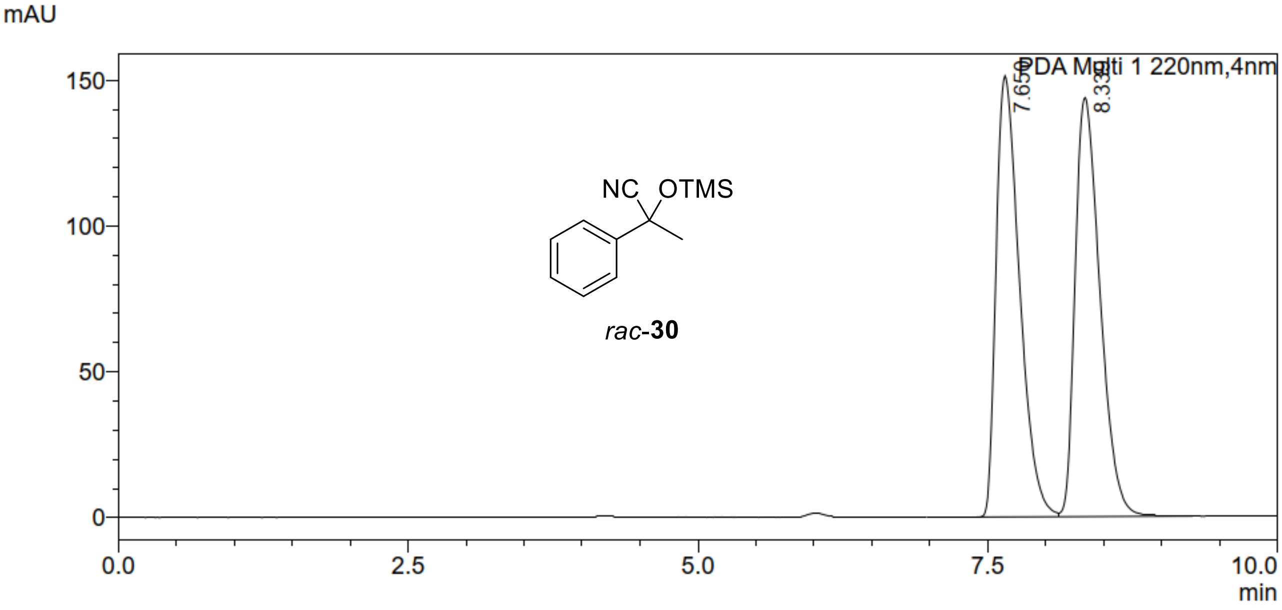

Peak #	t_R/min	% peak area
1	7.6	49.78
2	8.3	50.22
Total		100

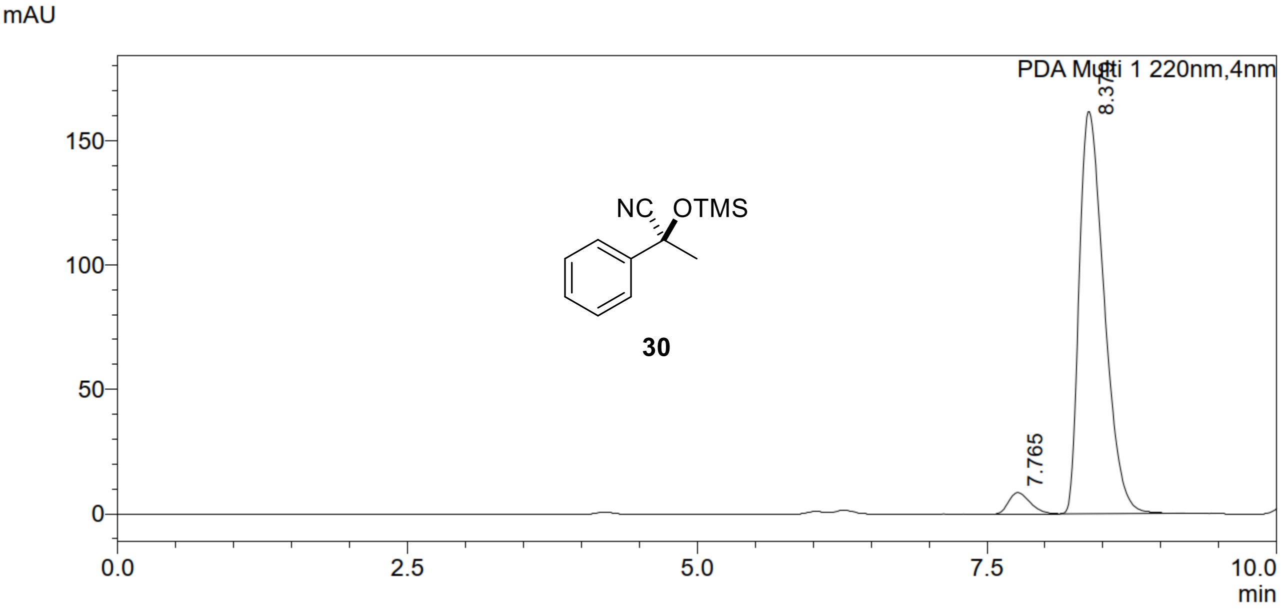

Peak #	t_R/min	% peak area
1	7.8	4.36
2	8.4	95.64
Total		100

HPLC (OJ-3R, MeOH: Water = 75:25, 1.0 mL/min, 298 K, 220 nm)

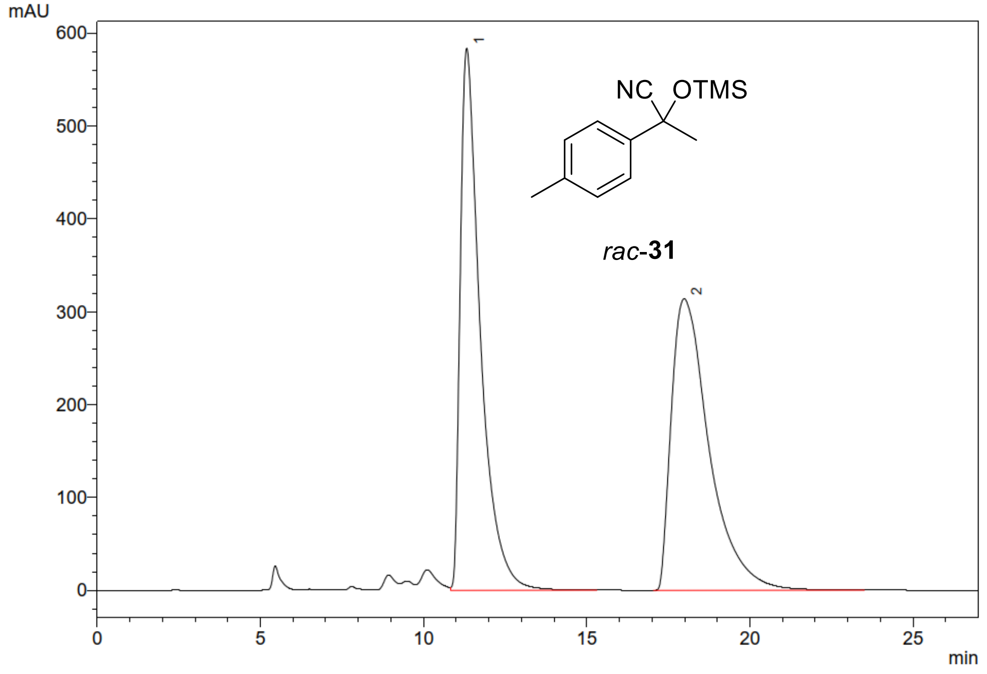

Peak #	t_R/min	% peak area
1	11.3	49.64
2	18.0	50.36
Total		100

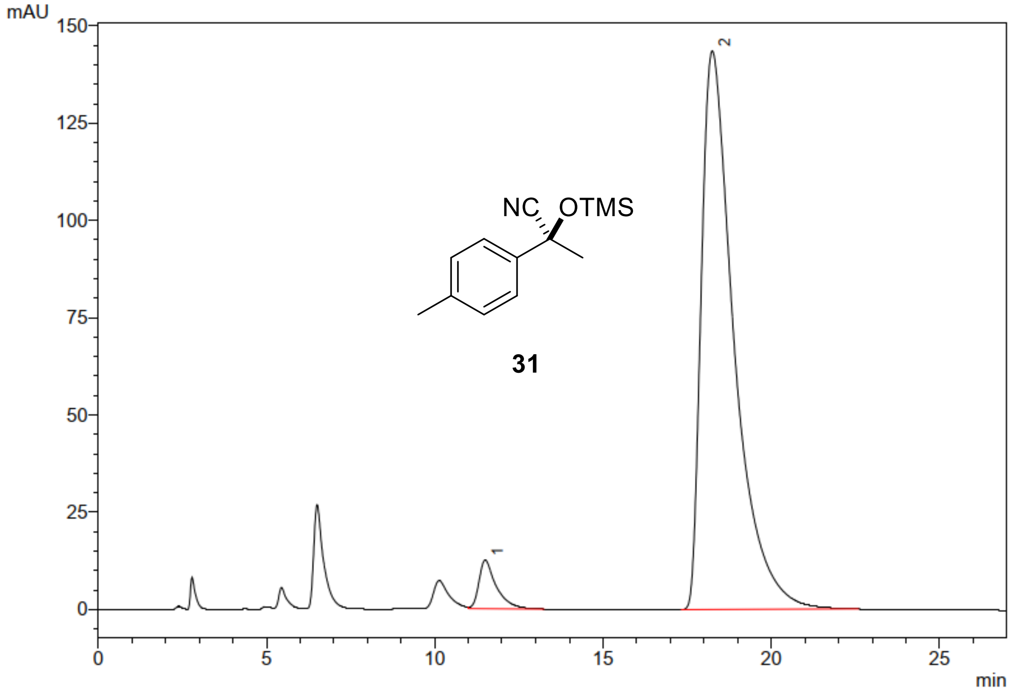

Peak #	t_R/min	% peak area
1	11.5	4.44
2	18.2	95.56
Total		100

HPLC (OJ-3R, MeOH: Water = 75:25, 1.0 mL/min, 298 K, 220 nm)

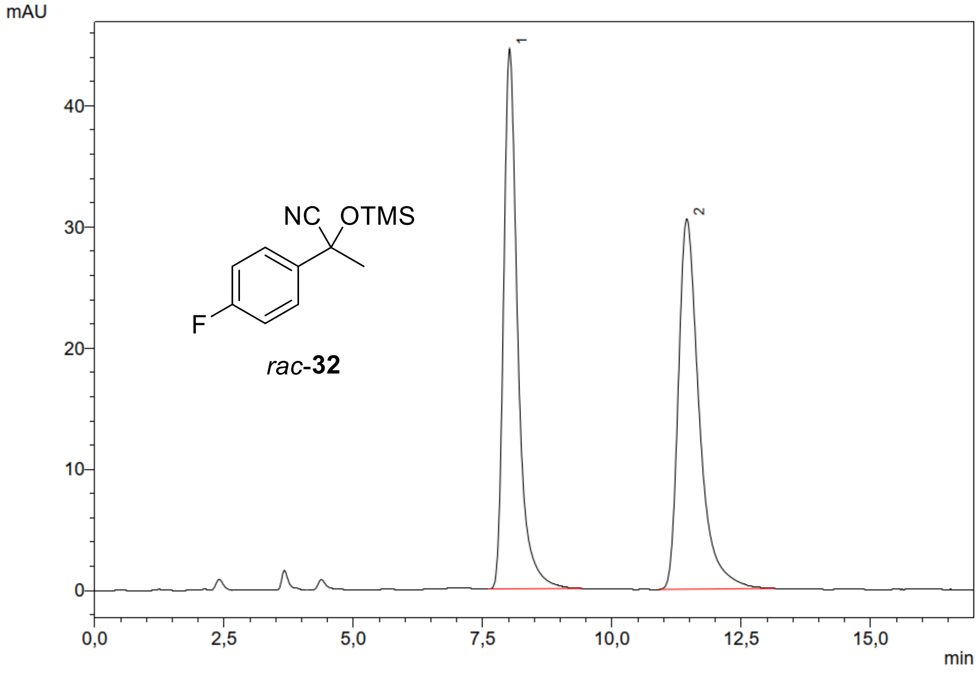

Peak #	t_R/min	% peak area
1	8.0	49.88
2	11.4	50.12
Total		100

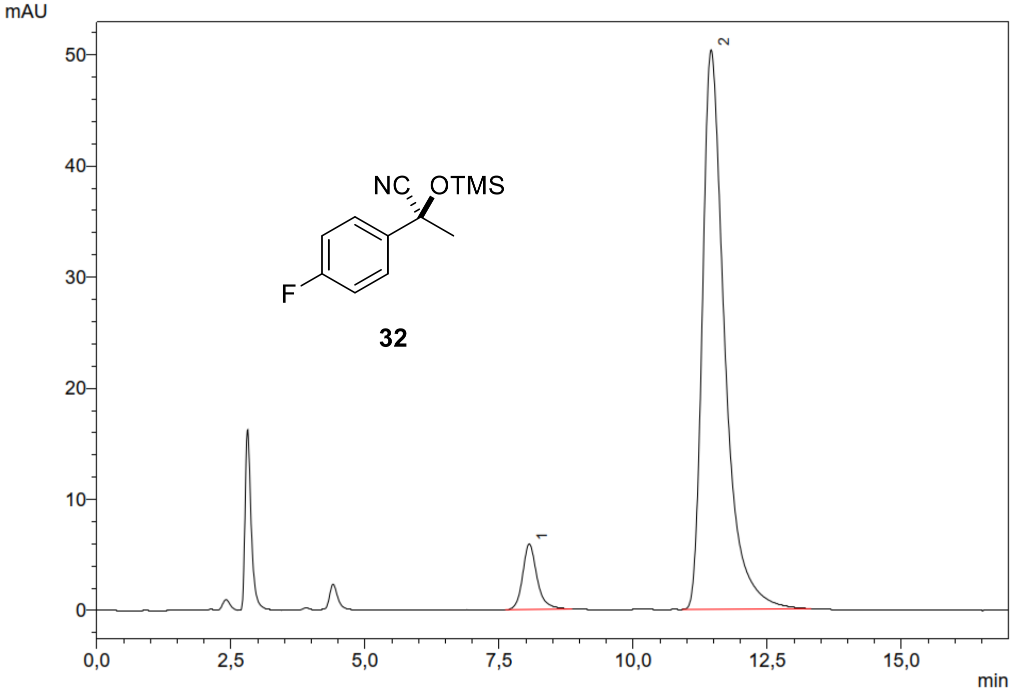

Peak #	t_R/min	% peak area
1	8.1	7.13
2	11.4	92.87
Total		100

HPLC (OJ-3R, MeOH: Water = 75:25, 1.0 mL/min, 298 K, 220 nm)

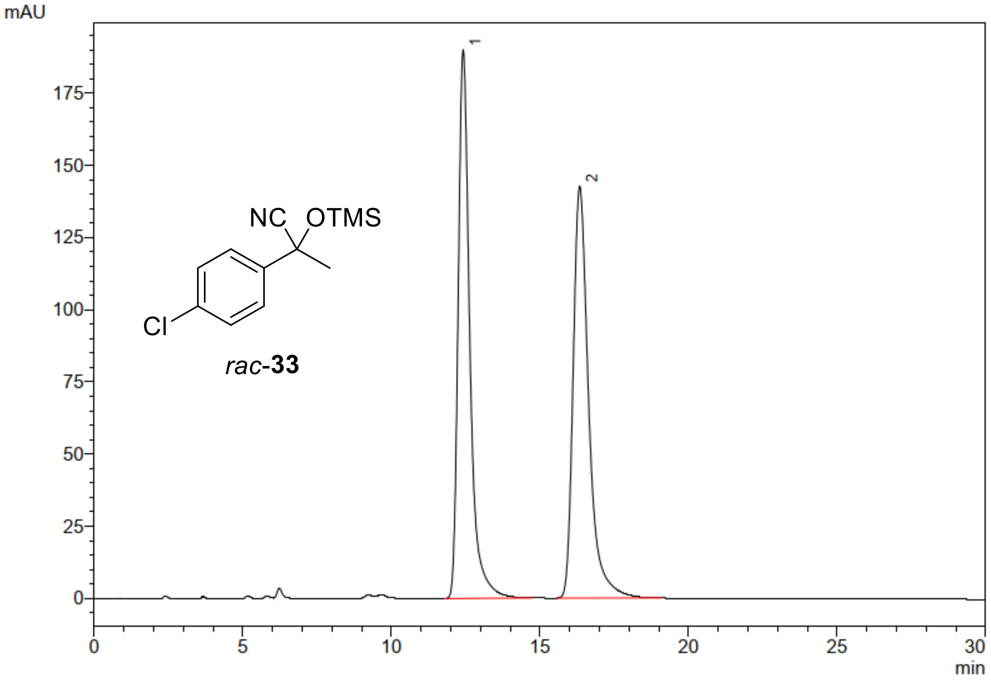

Peak #	t_R/min	% peak area
1	12.4	50.00
2	16.4	50.00
Total		100

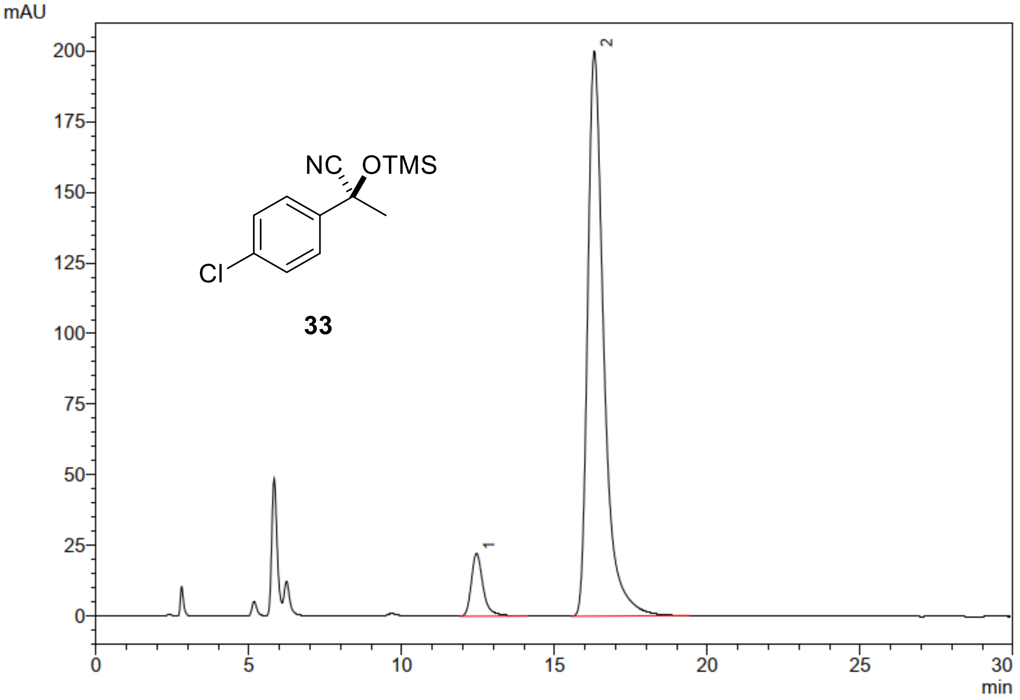

Peak #	t_R/min	% peak area
1	12.5	7.52
2	16.3	92.48
Total		100

HPLC (OJ-3R, MeOH: Water = 75:25, 1.0 mL/min, 298 K, 220 nm)

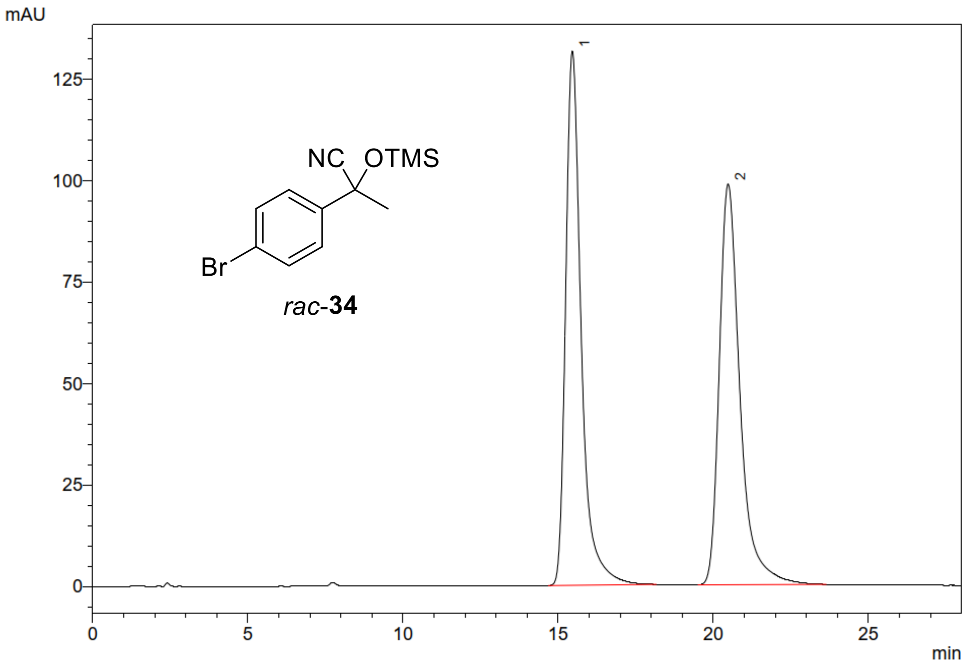

Peak #	t_R/min	% peak area
1	15.5	50.08
2	20.5	49.92
Total		100

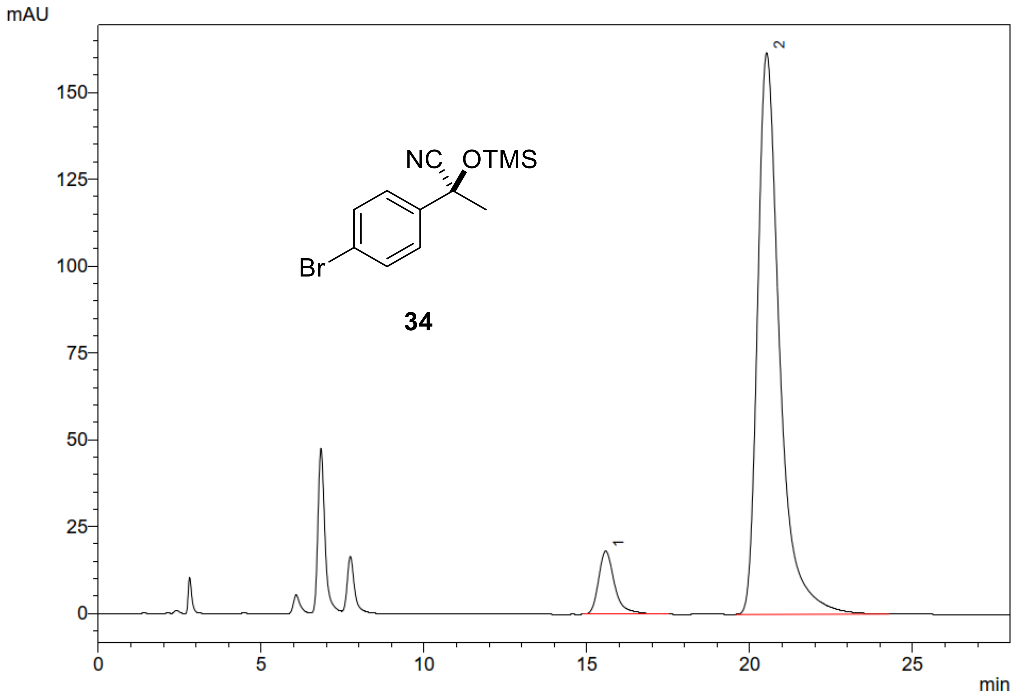

Peak #	t_R/min	% peak area
1	15.6	7.63
2	20.5	92.37
Total		100

HPLC (OJ-3R, MeOH: Water = 75:25, 1.0 mL/min, 298 K, 220 nm)

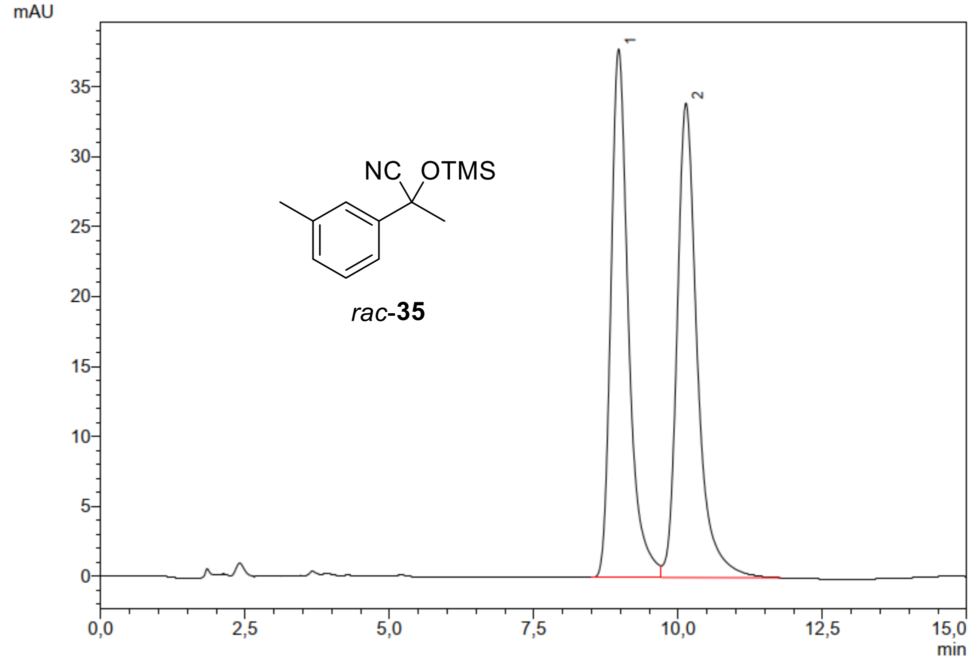

Peak #	t_R/min	% peak area
1	9.0	49.28
2	10.1	50.72
Total		100

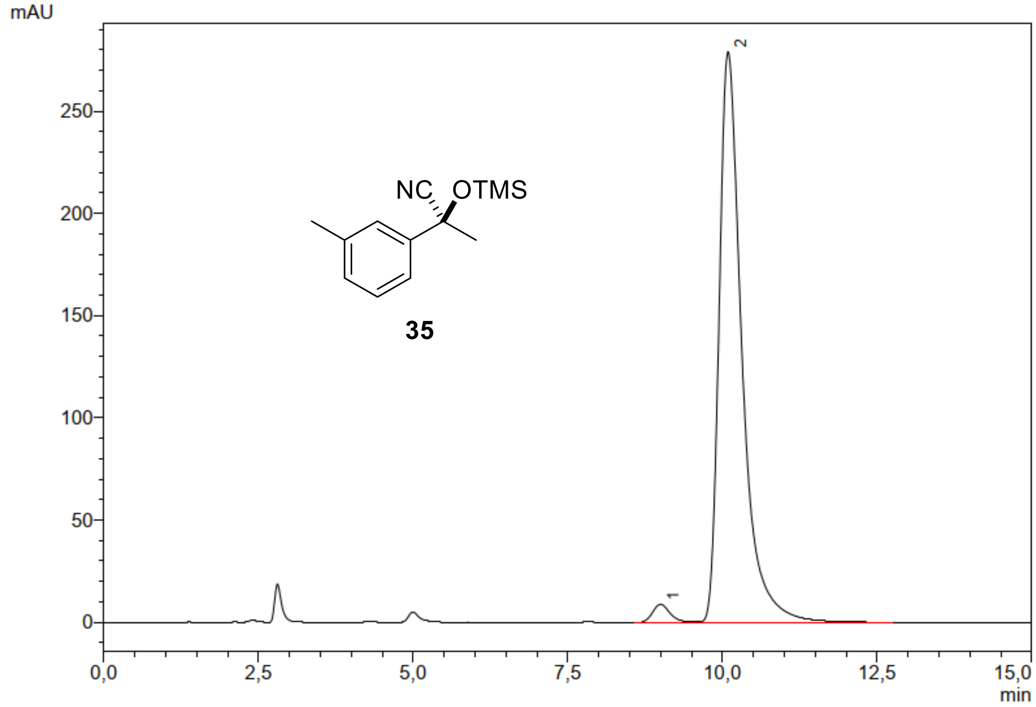

Peak #	t_R/min	% peak area
1	9.0	2.45
2	10.1	97.55
Total		100

HPLC (OD-3, 100% heptane, 0.5 mL/min, 298 K, 220 nm)

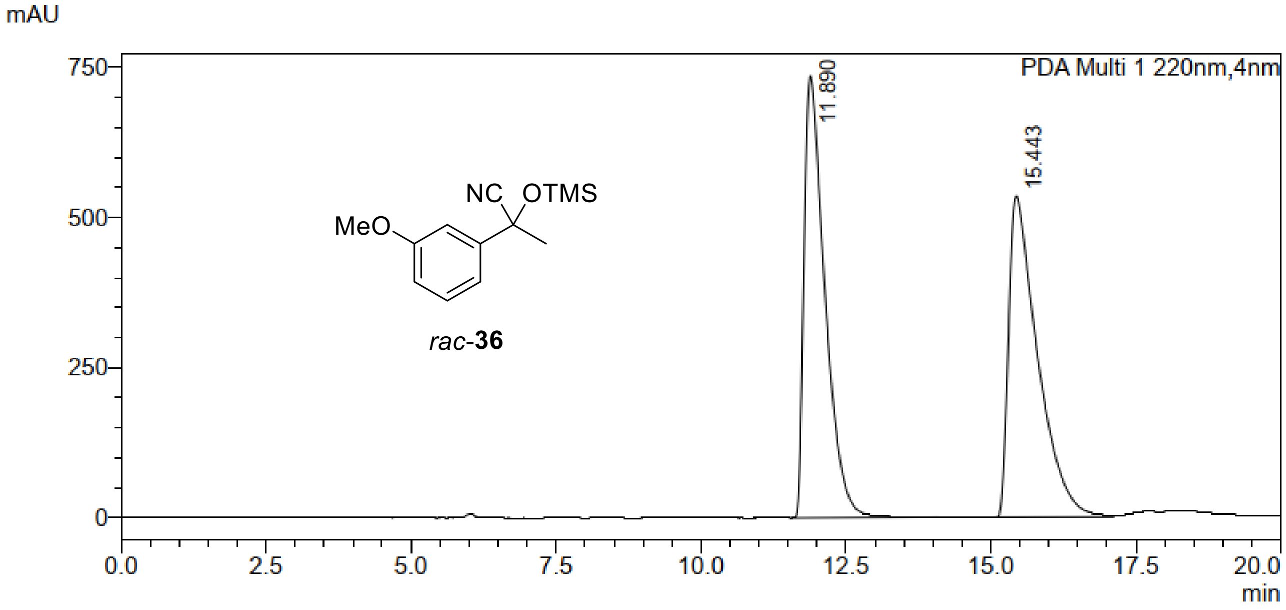

Peak #	t_R/min	% peak area
1	11.9	49.70
2	15.4	50.30
Total		100

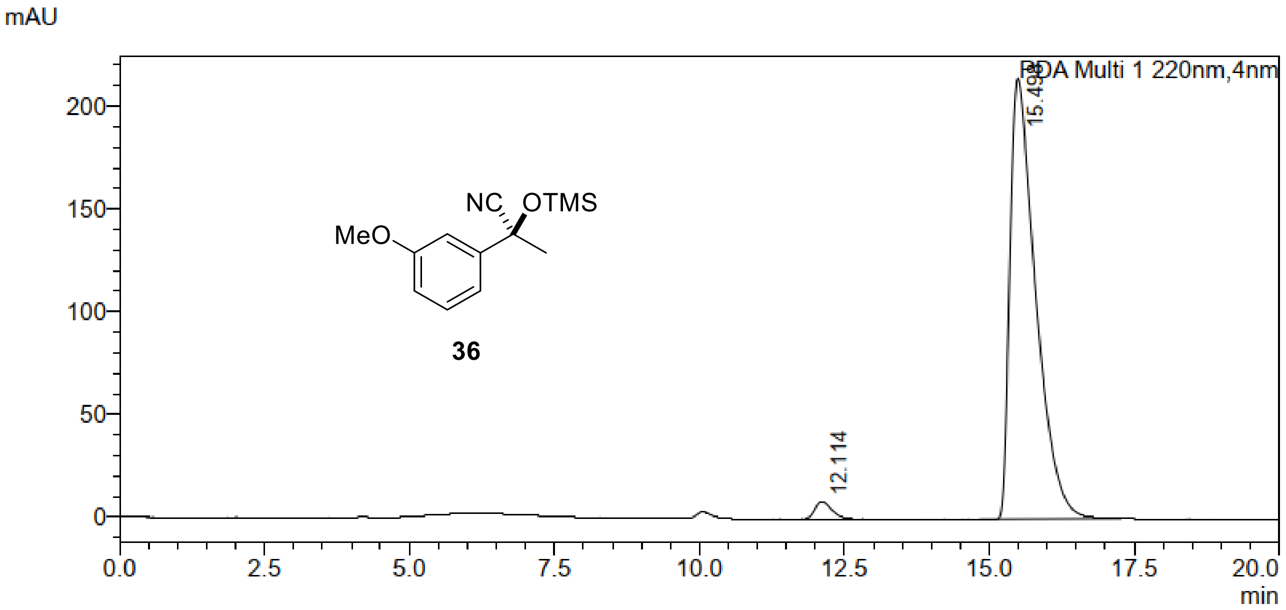

Peak #	t_R/min	% peak area
1	12.1	2.60
2	15.5	97.40
Total		100

HPLC (OD-3, 100% heptane, 0.5 mL/min, 298 K, 220 nm)

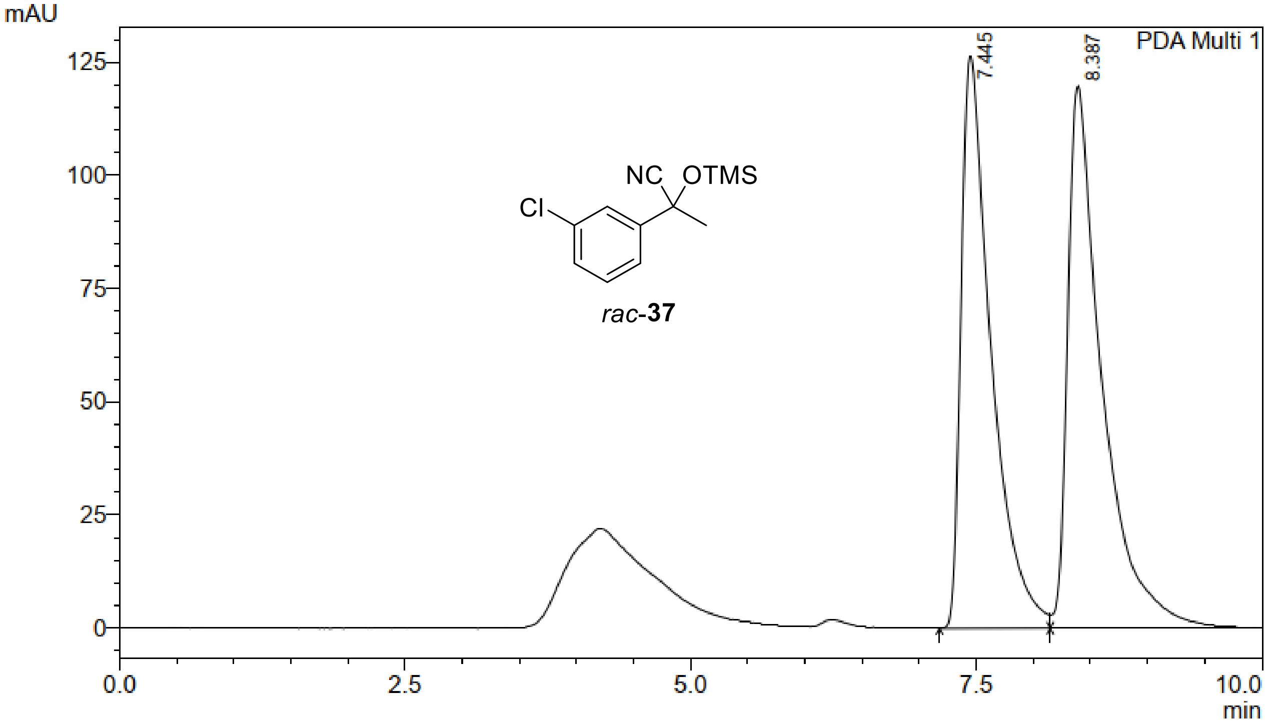

Peak #	t_R/min	% peak area
1	7.4	48.04
2	8.4	51.96
Total		100

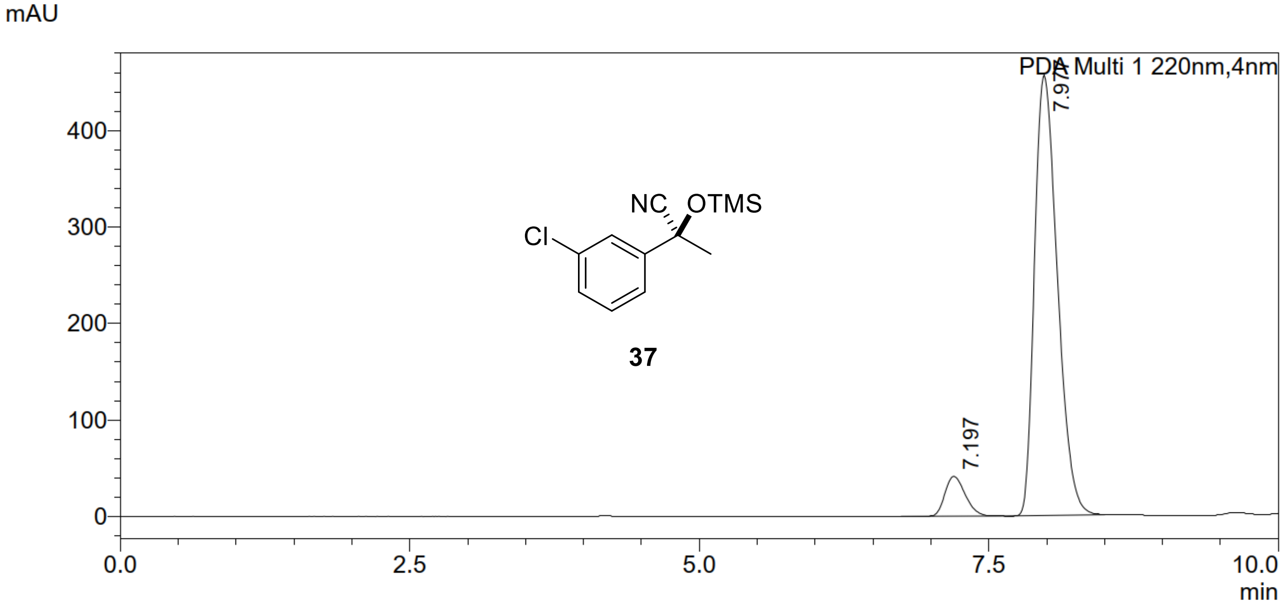

Peak #	t_R/min	% peak area
1	7.2	7.47
2	8.0	92.53
Total		100

HPLC (OD-3, 100% heptane, 0.5 mL/min, 298 K, 220 nm)

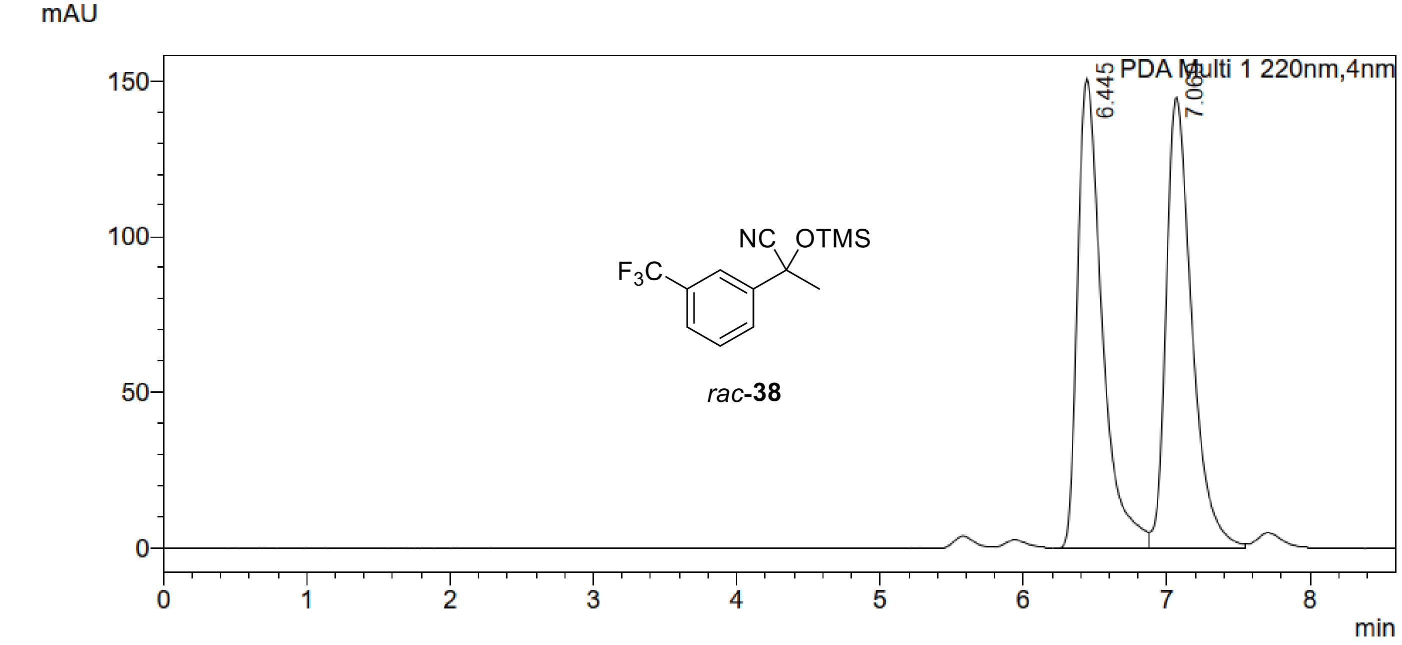

Peak #	t_R/min	% peak area
1	6.4	49.74
2	7.1	50.26
Total		100

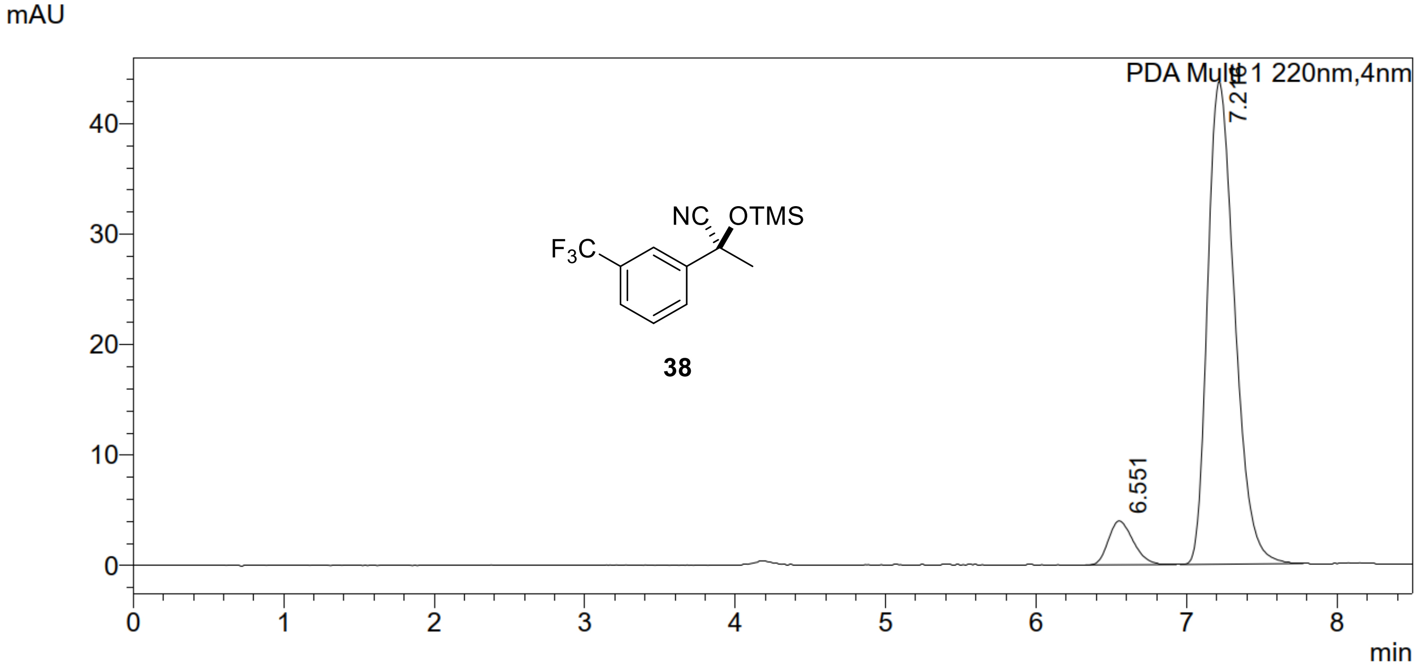

Peak #	t_R/min	% peak area
1	6.6	7.74
2	7.2	92.26
Total		100

HPLC (OD-3, 100% heptane, 0.5 mL/min, 298 K, 220 nm)

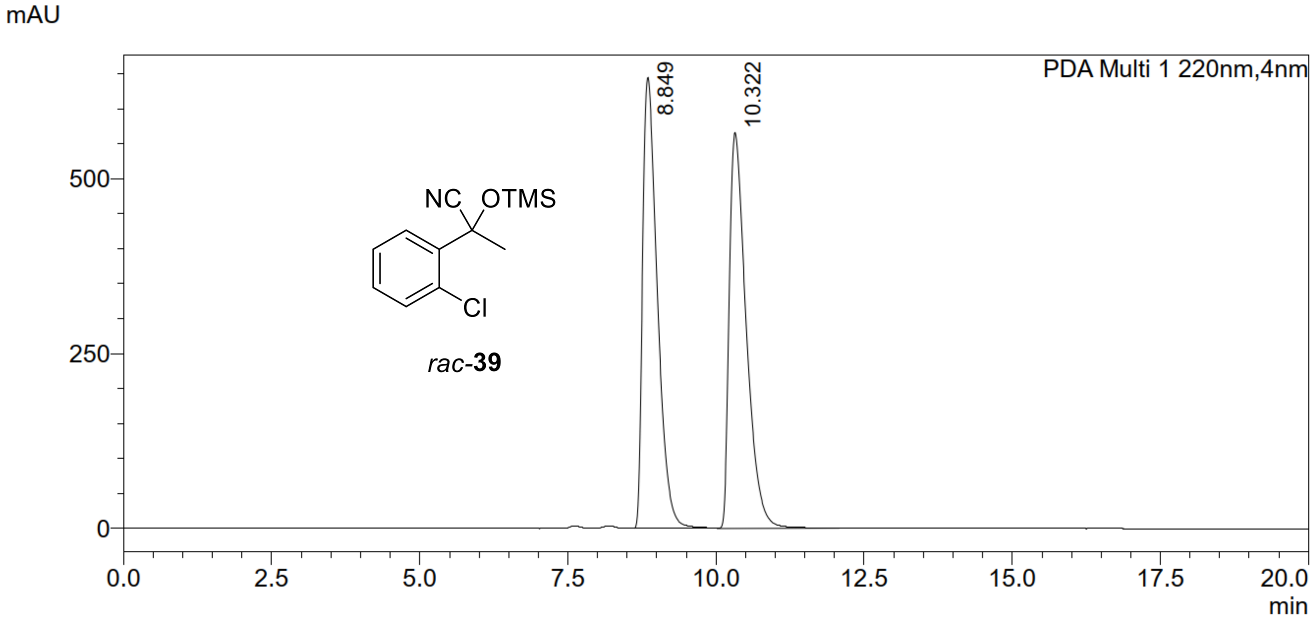

Peak #	t_R/min	% peak area
1	8.8	49.75
2	10.3	50.25
Total		100

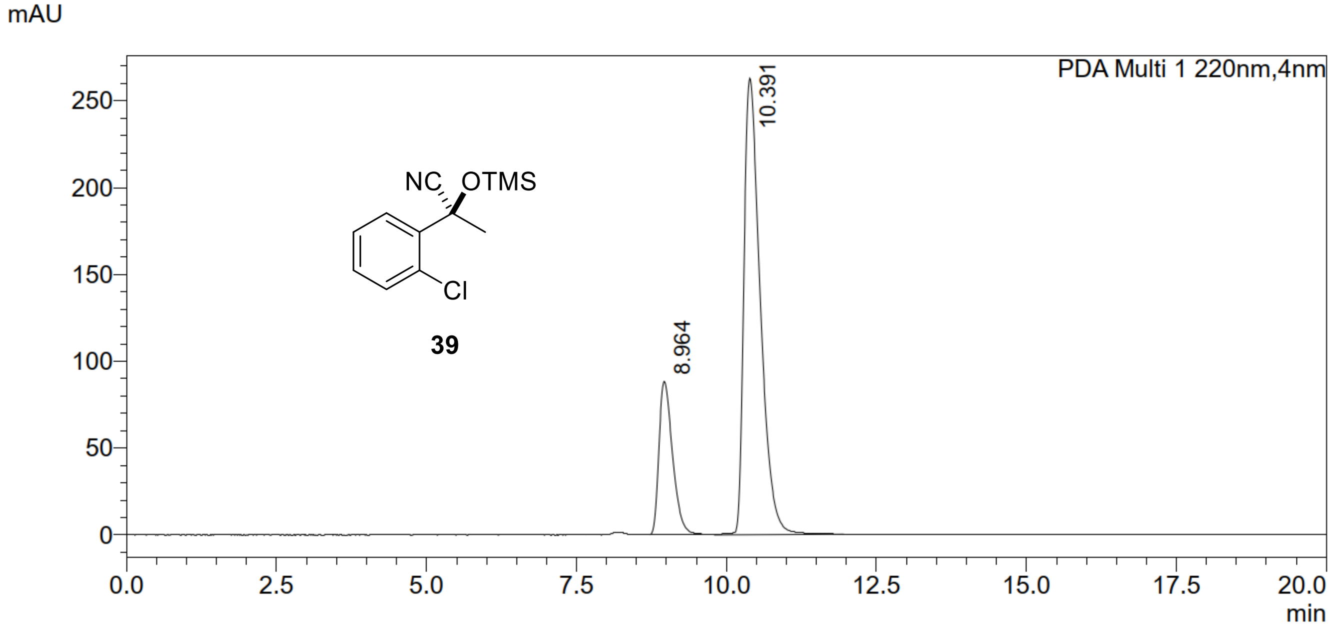

Peak #	t_R/min	% peak area
1	9.0	21.93
2	10.4	78.07
Total		100

GC (30.0 m G-TA, injection temperature: 220 °C, 80 °C iso 110 min, 8 °C/min, 180 °C iso 3 min, 0.5 bar H_2)

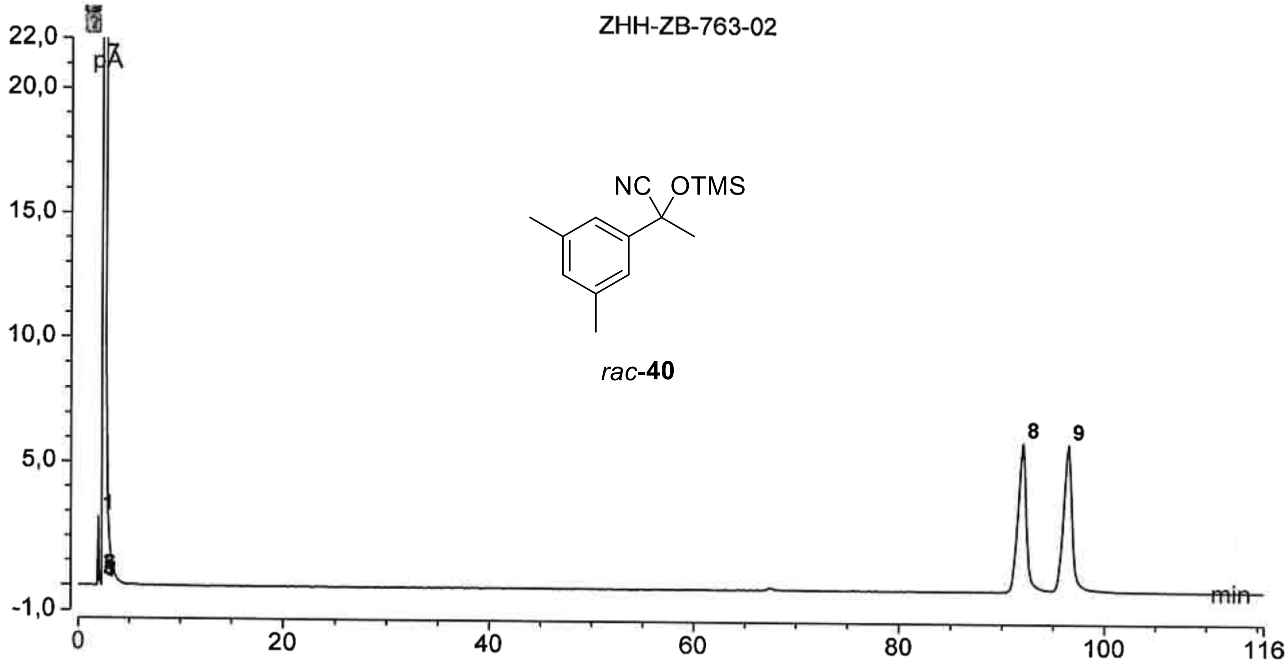

Peak #	t_R/min	% peak area
1	92.0	49.79
2	96.5	50.21
Total		100

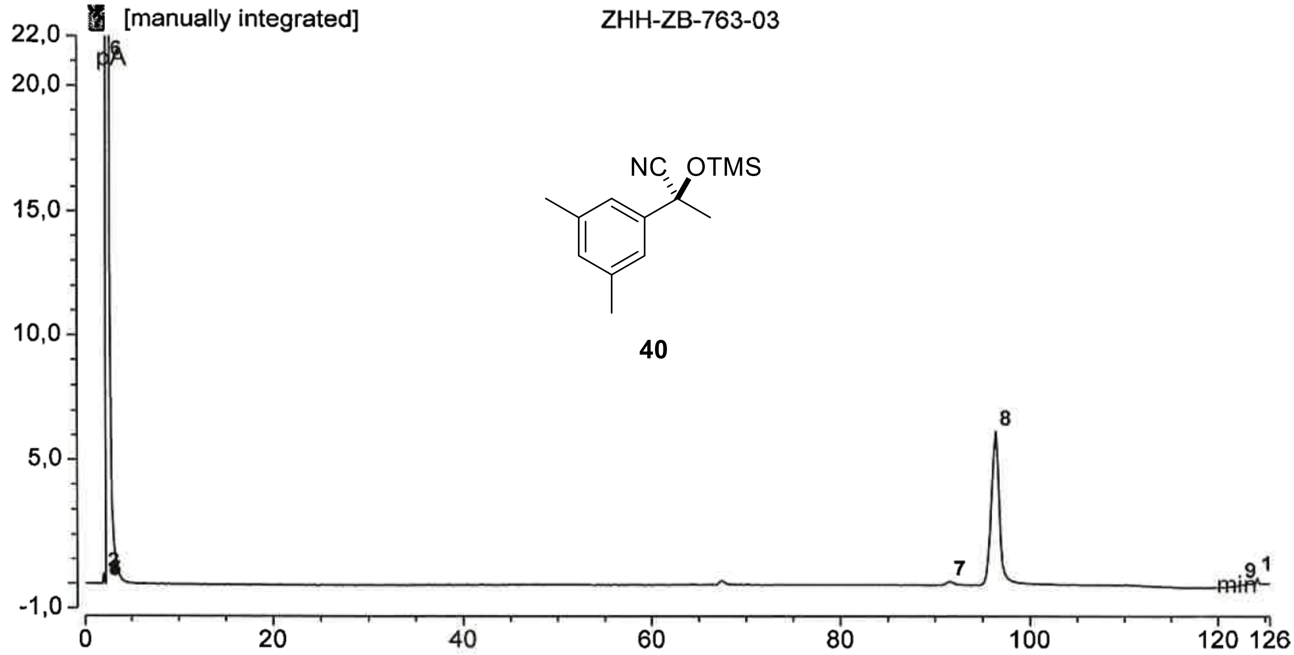

Peak #	t_R/min	% peak area
1	91.6	1.92
2	96.5	98.08
Total		100

HPLC (OD-3, 100% heptane, 0.5 mL/min, 298 K, 254 nm)

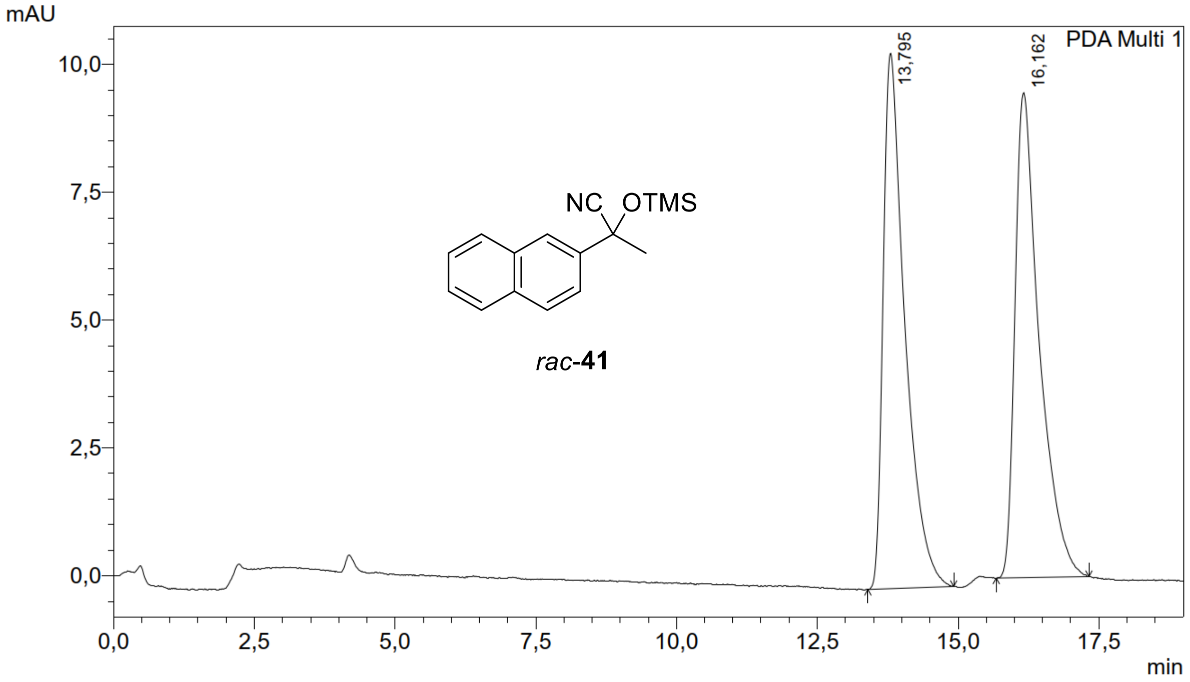

Peak #	t_R/min	% peak area
1	13.8	49.78
2	16.2	50.22
Total		100

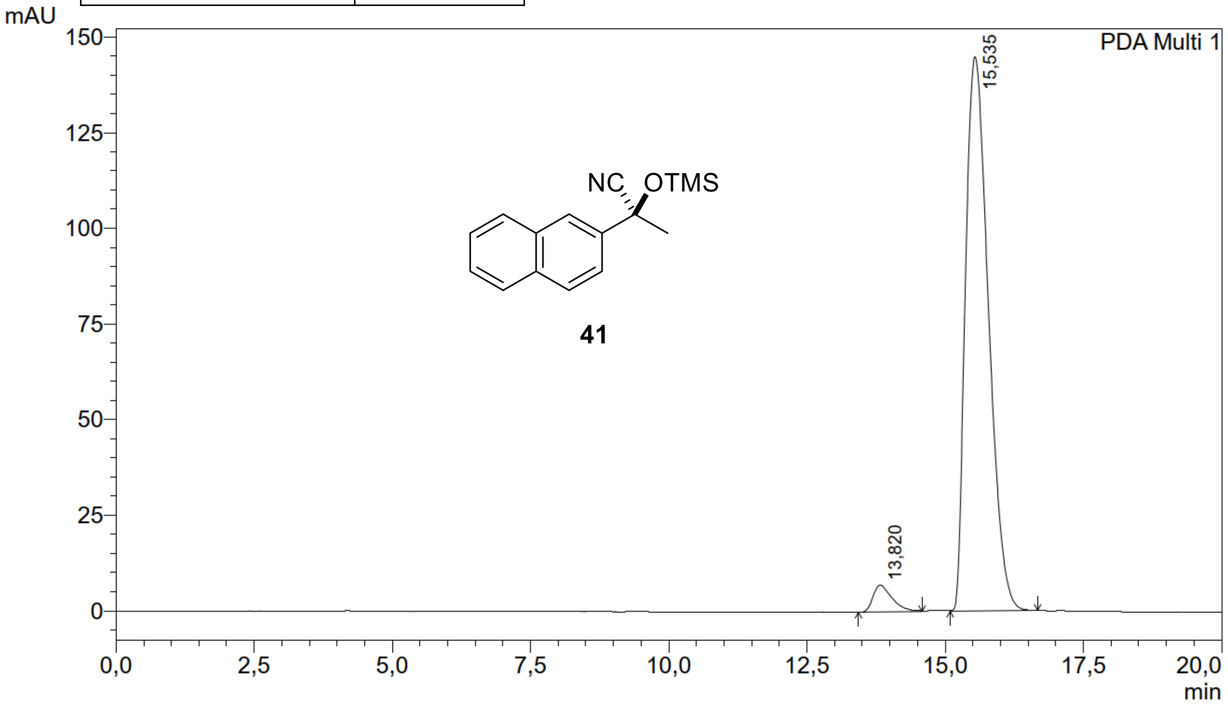

Peak #	t_R/min	% peak area
1	13.8	3.90
2	15.5	96.10
Total		100

GC (30.0 m G-TA, injection temperature: 220 °C, 90 °C iso 60 min, 8 °C/min, 180 °C iso 3 min, 0.5 bar H_2)

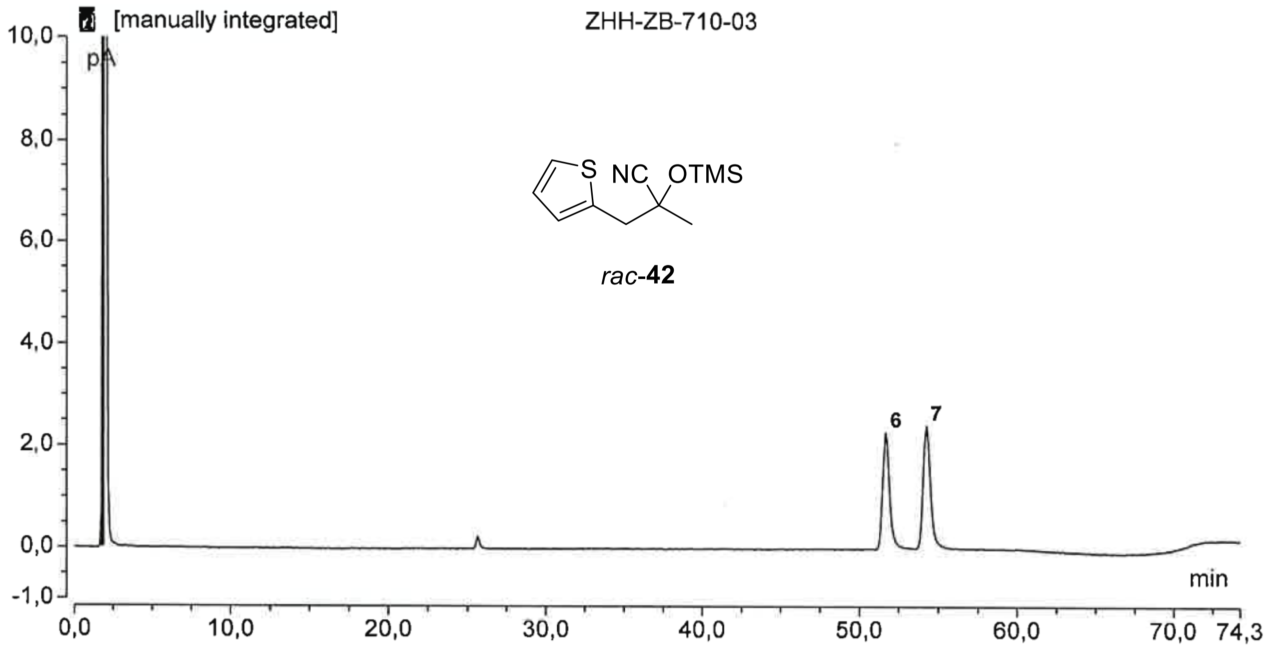

Peak #	t_R/min	% peak area
1	51.7	47.01
2	54.3	52.99
Total		100

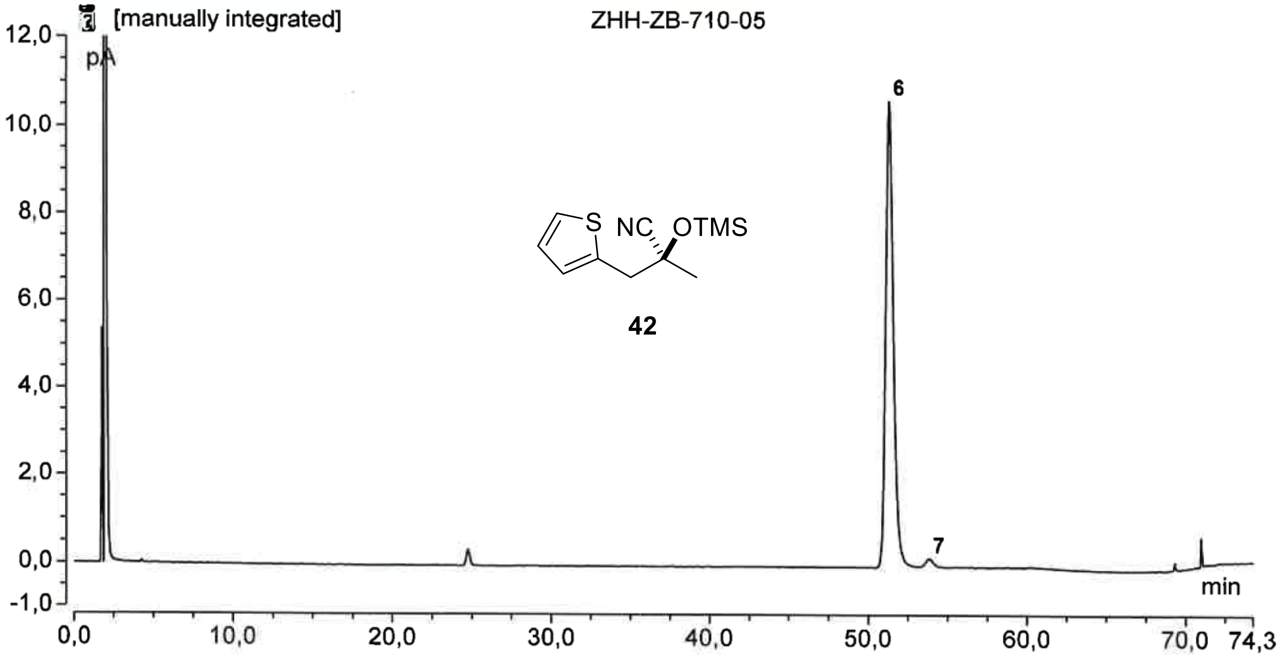

Peak #	t_R/min	% peak area
1	51.3	98.43
2	53.8	1.57
Total		100

GC (30.0 m G-TA, injection temperature: 220 °C, 70 °C iso 62 min, 8 °C/min, 180 °C iso 3 min, 0.5 bar H_2)

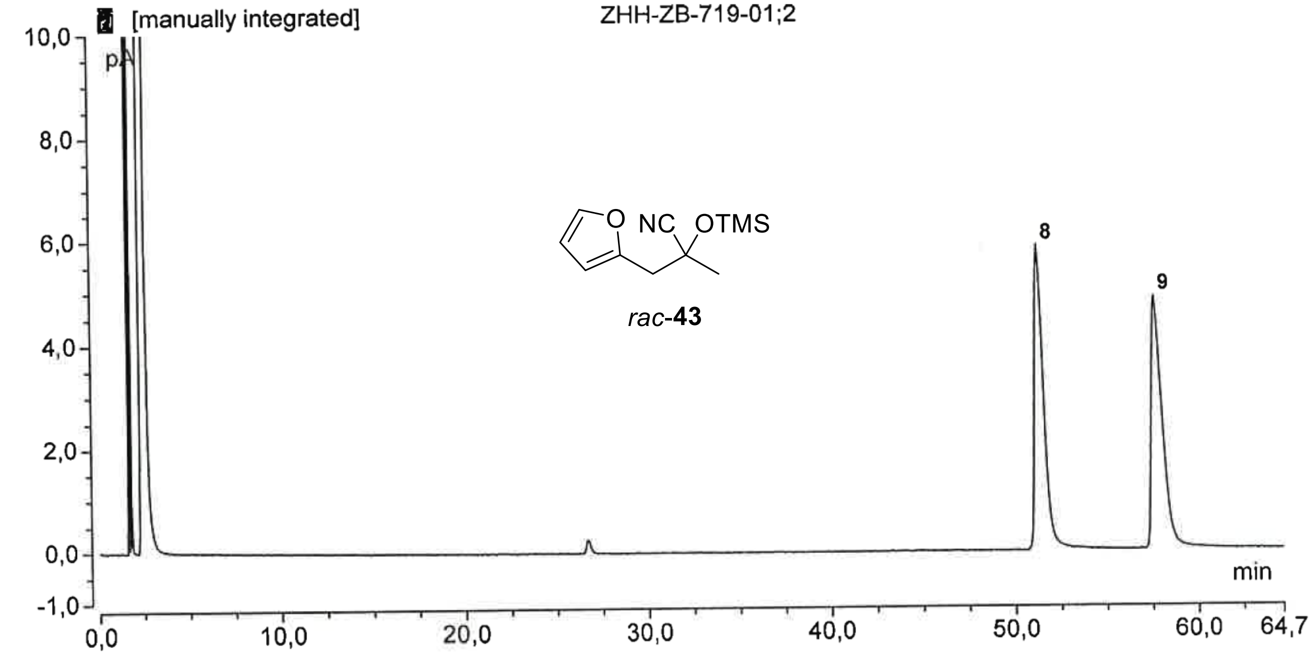

Peak #	t_R/min	% peak area
1	51.3	50.06
2	57.7	49.94
Total		100

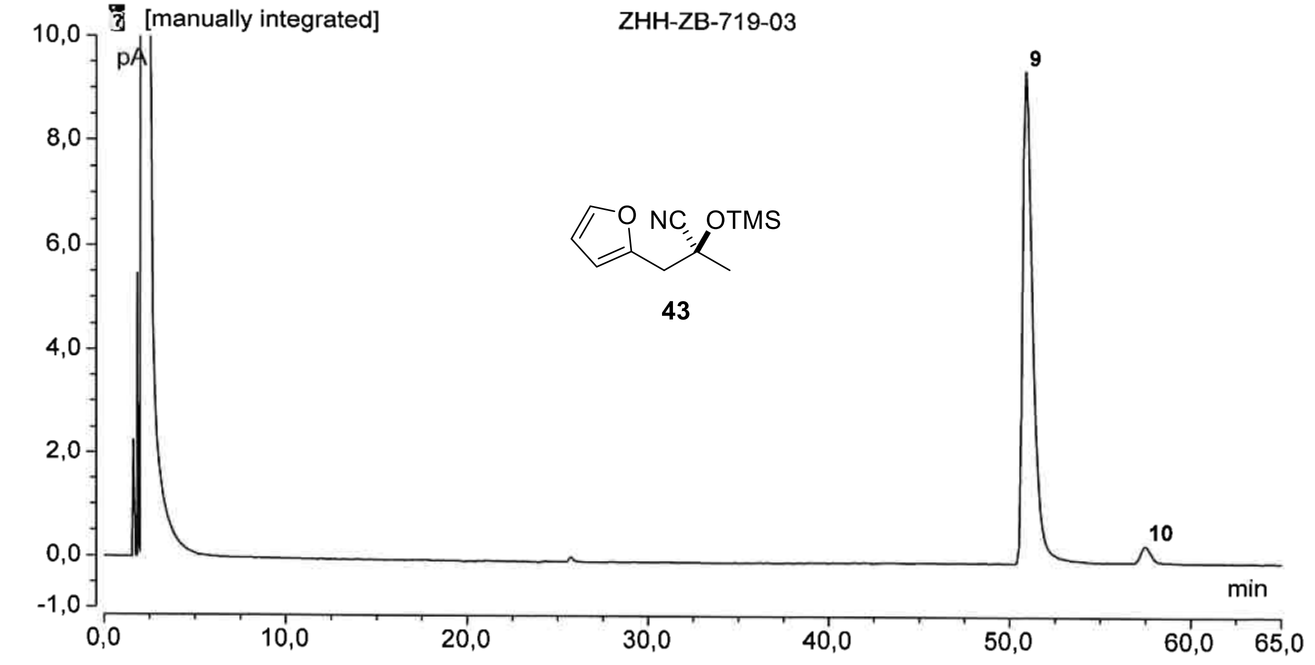

Peak #	t_R/min	% peak area
1	50.9	96.69
2	57.5	3.31
Total		100

HPLC (IA, 100% heptane, 0.5 mL/min, 298 K, 220 nm)

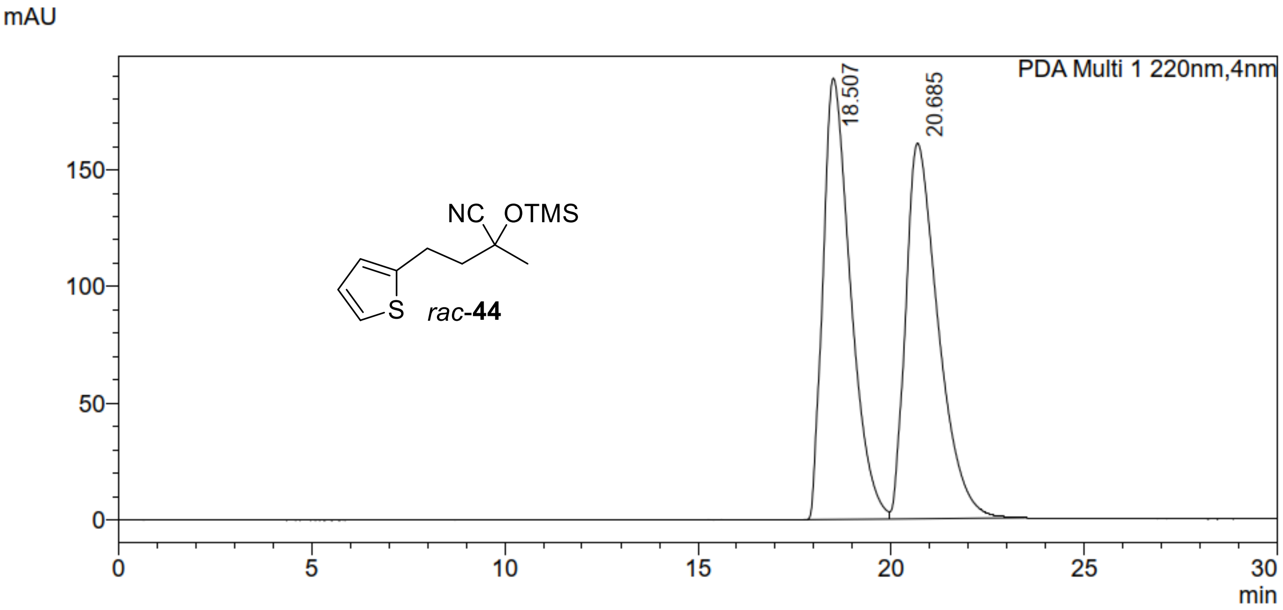

Peak #	t_R/min	% peak area
1	18.5	49.82
2	20.7	50.18
Total		100

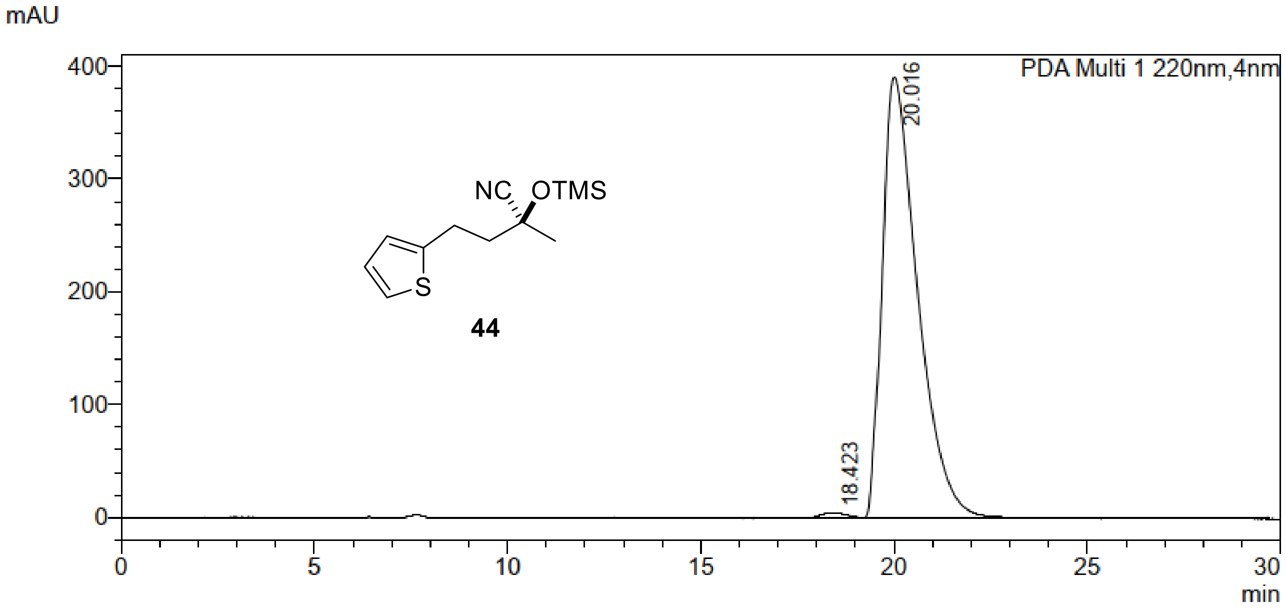

Peak #	t_R/min	% peak area
1	18.4	0.89
2	20.0	99.11
Total		100

*Note: the derivatives were made from product **23**, which was synthesized in gram scale (95:5 e.r.)*

HPLC (IC-3, isopropanol: heptane = 15:85, 1.0 mL/min, 298 K, 254 nm)

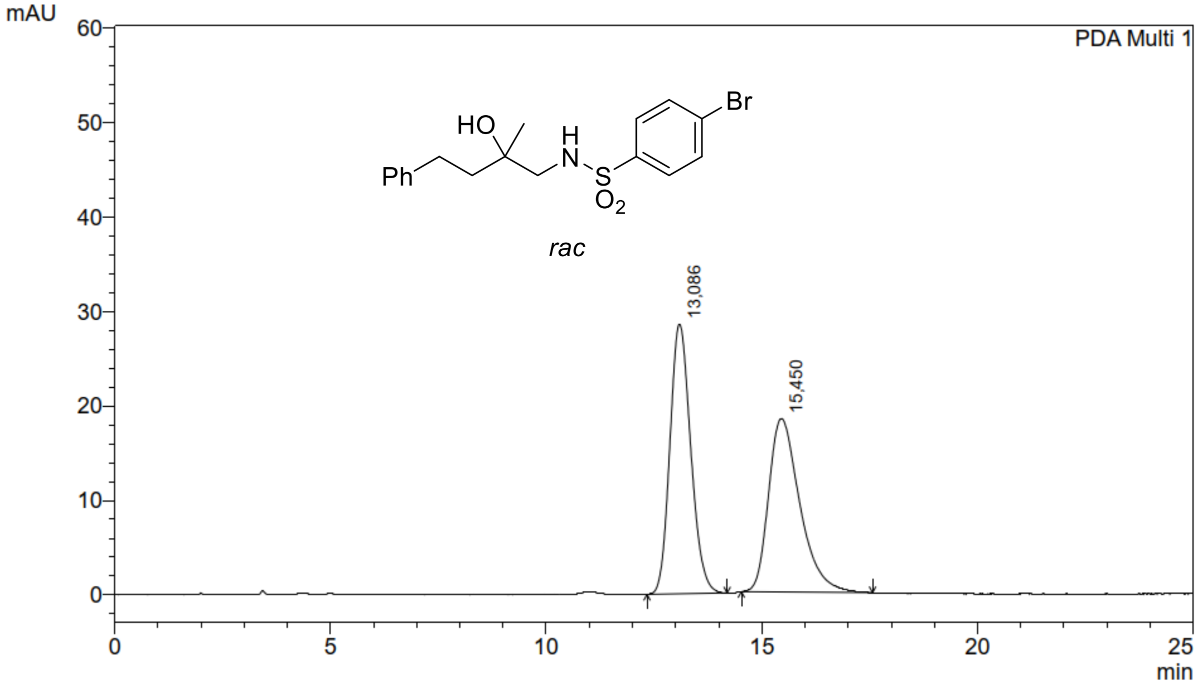

Peak #	t_R/min	% peak area
1	13.1	50.90
2	15.4	49.10
Total		100

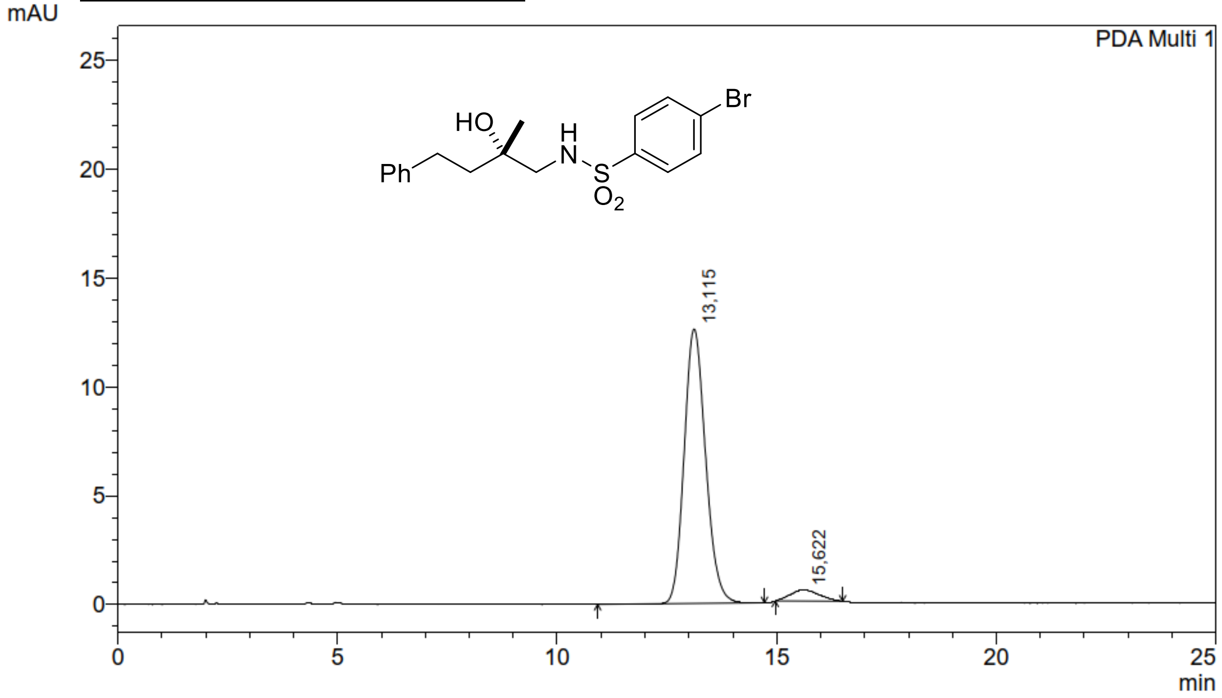

Peak #	t_R/min	% peak area
1	13.1	94.74
2	15.6	5.26
Total		100

HPLC (IE-3, isopropanol: heptane = 1:99, 1.0 mL/min, 298 K, 220 nm)

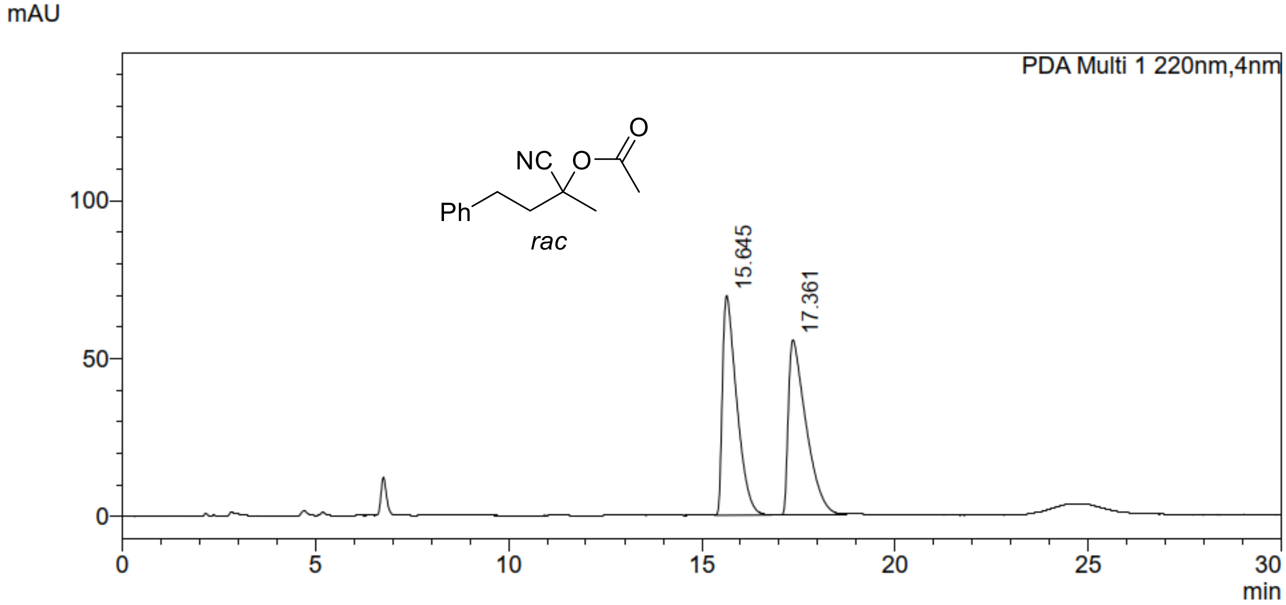

Peak #	t_R/min	% peak area
1	15.6	50.06
2	17.4	49.94
Total		100

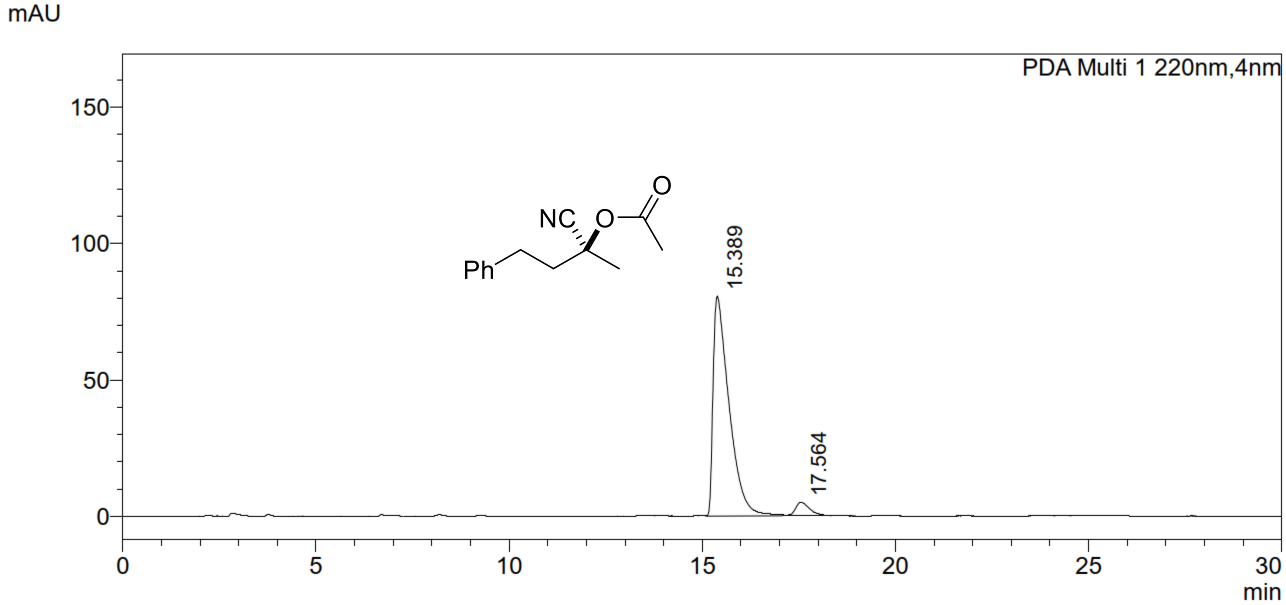

Peak #	t_R/min	% peak area
1	15.4	94.60
2	17.6	5.40
Total		100

HPLC (OD-3, isopropanol: heptane = 0.5:99.5, 1.0 mL/min, 298 K, 190 nm)

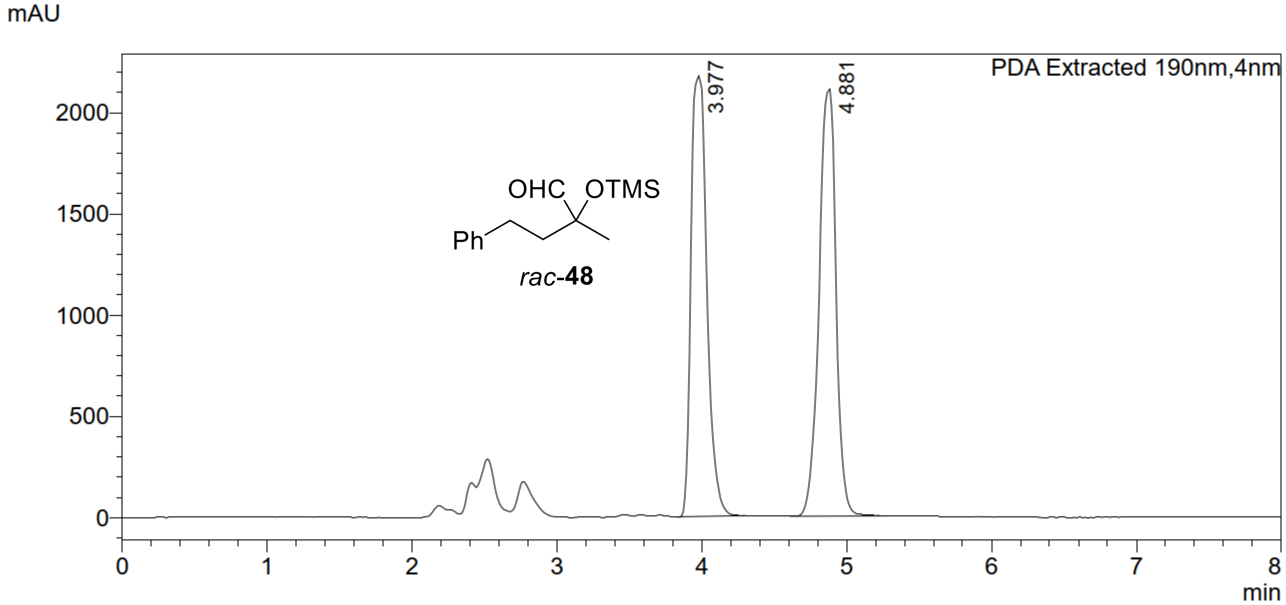

Peak #	t_R/min	% peak area
1	4.0	50.80
2	4.9	49.20
Total		100

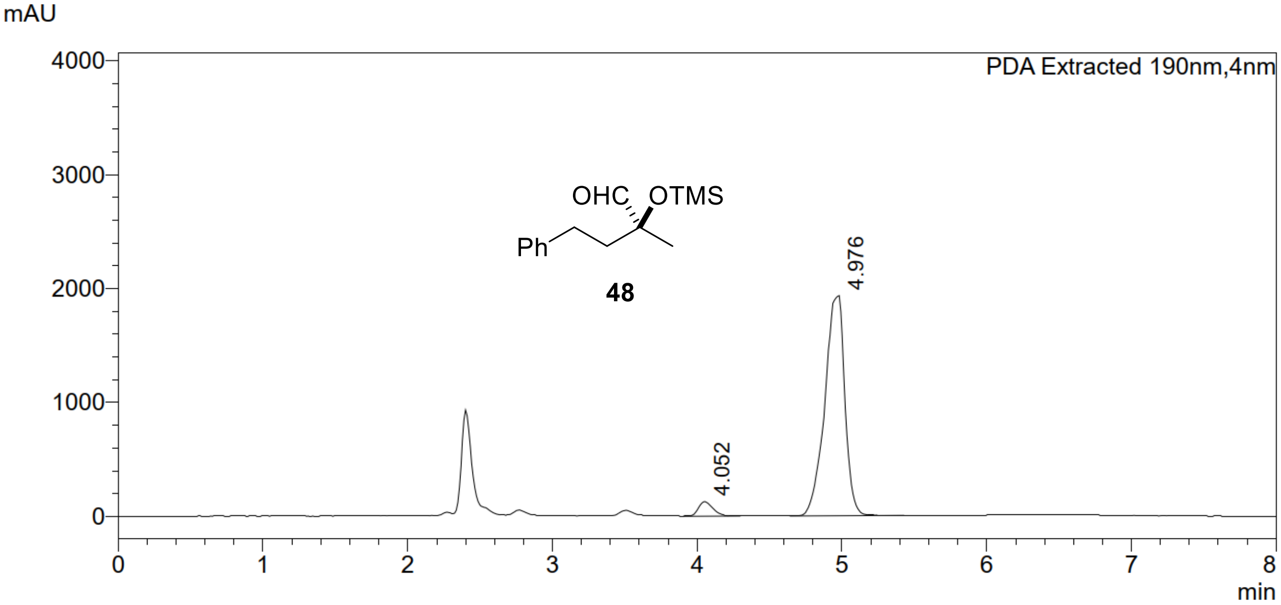

Peak #	t_R/min	% peak area
1	4.1	4.62
2	5.0	95.38
Total		100

HPLC (AD-3, isopropanol: heptane = 5:95, 1.0 mL/min, 298 K, 190 nm)

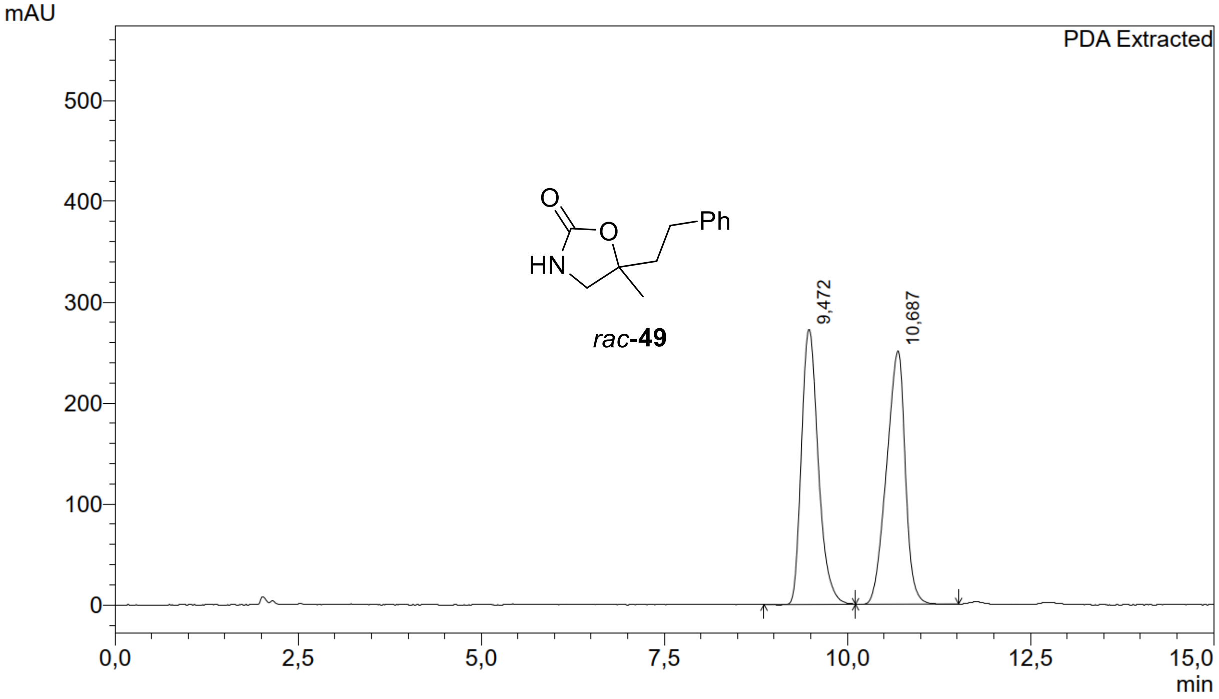

Peak #	t_R/min	% peak area
1	9.5	49.48
2	10.7	50.52
Total		100

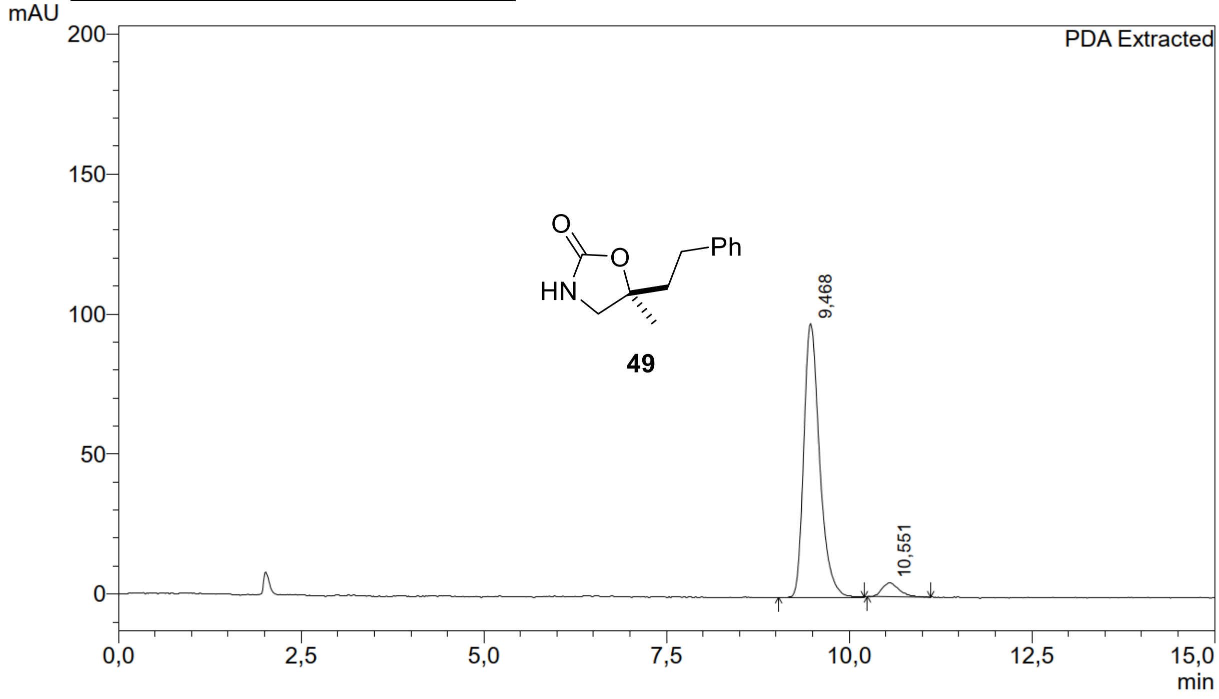

Peak #	t_R/min	% peak area
1	9.5	94.88
2	10.6	5.12
Total		100

HPLC (IA, isopropanol: heptane:TFA = 2:98:0.1, 1.0 mL/min, 298 K, 220 nm)

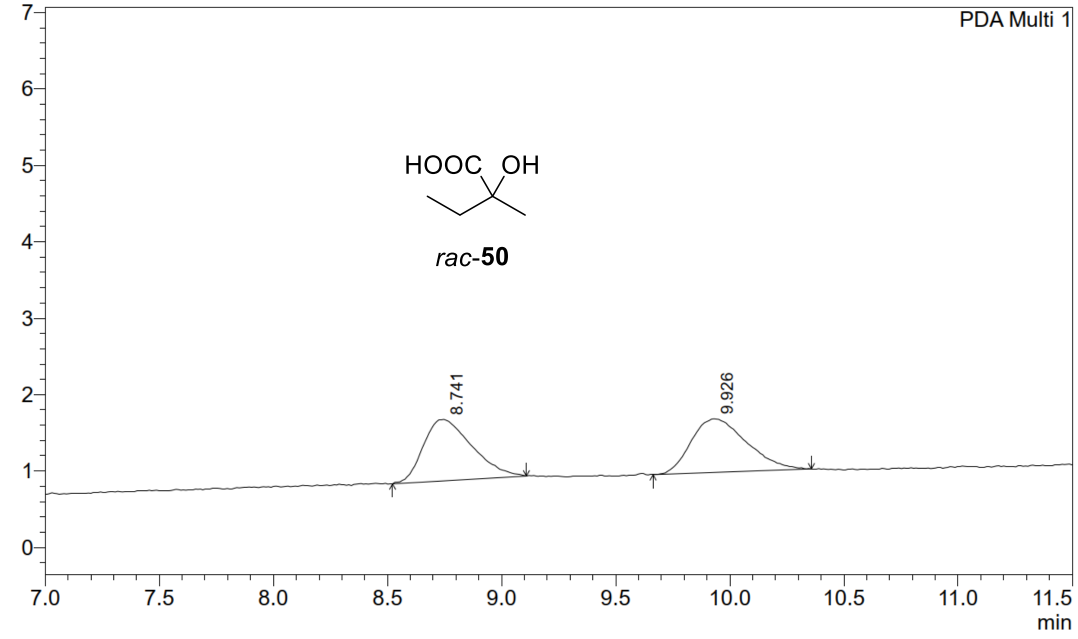

Peak #	t_R/min	% peak area
1	8.7	50.02
2	9.9	49.98
Total		100

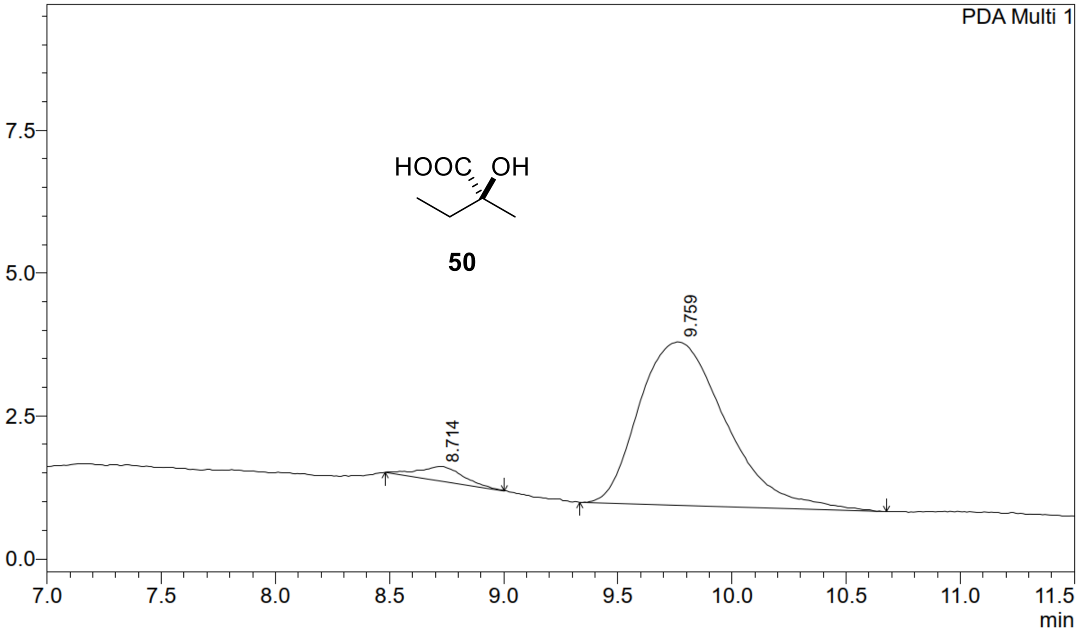

Peak #	t_R/min	% peak area
1	8.7	4.55
2	9.8	95.45
Total		100
